# Proceedings of Réanimation 2020, the French Intensive Care Society International Congress

**DOI:** 10.1186/s13613-020-0623-7

**Published:** 2020-02-11

## Acknowledgements

This abstract book was edited and corrected by the members of the Congress Committee of the French Intensive Care Society:

- Frédéric Pène (Paris)
- Hafid Ait-Oufella (Paris)
- Pierre Asfar (Angers)
- Cécile Aubron (Brest)
- Emmanuel Canet (Nantes)
- Guillaume Carteaux (Créteil)
- Stephan Ehrmann (Tours)
- Jean-Pierre Frat (Poitiers)
- Guillaume Geri (Boulogne-Billancourt)
- Julie Helms (Strasbourg)
- Alexandre Demoule (Paris)
- Saad Nseir (Lille)
- Mehdi Oualha (Paris)

## Oral Communications

### COK-1 Expiratory muscle weakness quantified by maximal expiratory pressure may be insufficient in predicting critical outcomes in mechanically ventilated patients

#### Yann Combret^1^, Guillaume Prieur^1^, Francis-Edouard Gravier^2^, Tristan Bonnevie^2^, Olivier Contal^3^, Bouchra Lamia^1^, Clement Medrinal^1^

##### ^1^Groupe Hospitalier du Havre, Le Havre, France; ^2^ADIR Association, Rouen, Fs; ^3^Haute Ecole de Santé Vaud, Lausanne, Switzerland

###### **Correspondence:** Yann Combret (yann.combret@gmail.com)

*Ann. Intensive Care* 2020, **10 (Suppl 1):**COK-1

**Rationale:** Expiratory muscles has recently been stated as the «neglected component» in mechanically ventilated patient. Several authors stated these muscles importance in cough capacity, contractile efficiency of the diaphragm or reduction of hyperinflation. However, few studies reported potential factors leading to expiratory muscle weakness and its importance on weaning success or survival after mechanical ventilation.

**Patients and methods:** This study is a secondary analysis of our previously described cohort of 124 patients ventilated for at least 24 h assessed for respiratory muscles function. Maximal expiratory pressure (MEP) measurement was carried out during spontaneous breathing trial using a manometer with an unidirectional valve. MEP diagnostic accuracy to predict ICU-AW (ICU acquired weakness), weaning success and sursvival within 30 days were assessed using expiratory muscle strength as absolute values (cmH_2_O), as %predicted values and as %lower limit of normal.

**Results:** Due to the paucity of data reporting threshold value for expiratory muscle weakness, we considered our median value (47 cmH_2_O (IQR 44)) as the threshold value for expiratory muscle weakness group (MEP ≤ 47 cmH_2_O) and normal expiratory muscle group (MEP > 47 cmH_2_O). Patients with low MEP received more catecholamines (p = 0.04) and a higher duration of mechanical ventilation (p = 0.001). Inversely, higher body mass index was associated with higher MEP. Patients with low MEP presented more ICU-AW compared to normal MEP patients (64% vs. 35%; p = 0.003). No other outcomes were different between groups. MEP was statistically able to predict ICU-AW but area under (AUC) receiving operating curves showed weak predictive ability (AUC: 0.66 (95% IC 0.55–0.77; p < 0.01) for a threshold value ≤ 49 cmH_2_O. Expiratory muscle weakness was unable to predict critical outcomes when adjusting MEP to the %predicted or lower limit of normal.

**Discussion:** Possible explanation is that contrary to inspiratory muscle weakness, cough inefficacy after weaning from mechanical ventilation could be managed with cough supplementation techniques (*i.e.* mechanical in-exsufflation).

**Conclusion:** In our cohort, MEP was not associated with mechanical ventilation weaning or death. Despite our results, different clinical techniques for quantifying expiratory muscle weakness may provide more beneficial results.

**Compliance with ethics regulations**: Yes

**Table 1:** Patients’ outcomes

Outcomes	Low MEP N = 63	Normal MEP N = 61	Risk Ratio [95% IC]	*p* value
Extubation failure, n (%)	14 (22)	8 (13)	1.32 (0.91–1.93)	0.18
Death within 30 days, n (%)	10 (16)	5 (8)	1.37 (0.91–2.06)	0.19
ICU-AW, n (%*)	32 (64*)	17 (35*)	1.82 (1.19–2.77)	0.003
Readmission in ICU within 30 days, n (%)	1 (2)	1 (2)	0.98 (0.24–3.98)	0.98

CI: Confidence interval; ICU-AW: Intensive care unit acquired weakness; MEP: Maximal expiratory pressure; *99/124 has been assessed for ICU-AW (MRC score)

### COK-2 Consequences of ICU acquired weakness: a systematic review and meta-analysis

#### Clement Medrinal^1^, Yann Combret^2^, Roger Hilfiker^3^, Nadine Aroichane^4^, Tristan Bonnevie^5^, Francis-Edouard Gravier^5^, Guillaume Prieur^1^, Olivier Contal^6^, Bouchra Lamia^7^

##### ^1^Le Havre Hospital, ICU Department. Normandie University, UNIROUEN, UPRES EA3830-GRHV, Institute for Research and Innovation in Biomedicine (IRIB), Le Havre, France; ^2^Le Havre Hospital, ICU Department. Research and Clinical Experimentation Institute (IREC), Pulmonology, ORL and Dermatology, Louvain Catholic University, Brussels, Le Havre, France; ^3^University of Applied Sciences and Arts Western Switzerland Valais (HES-SO Valais-Wallis), School of Health Sciences, Leukerbad, Leukerbad, Switzerland; ^4^IFMK de Rouen, Rouen, France; ^5^ADIR Association Normandie University, UNIROUEN, UPRES EA3830-GRHV, Institute for Research and Innovation in Biomedicine (IRIB), Bois Guillaume, France; ^6^University of Applied Sciences and Arts Western Switzerland (HES-SO), Lausanne, Switzerland; ^7^Le Havre Hospital, Pulmonology Department. Normandie University, UNIROUEN, UPRES EA3830-GRHV, Institute for Research and Innovation in Biomedicine (IRIB), Le Havre, France

###### **Correspondence:** Clement Medrinal (medrinal.clement.mk@gmail.com)

*Ann. Intensive Care* 2020, **10 (Suppl 1):**COK-2

**Rationale:** It is increasingly recognized that identification of muscle weakness in ICU should be as early as possible. Unfortunately, volitional tests can be assessed in a minority of patients who are awake and cooperative. Some studies proposed alternative assessments conductable without patient cooperation such as magnetic phrenic nerve stimulation or muscular ultrasonography. However, clinical relevance of these tests are not determined and further correlation with muscle strength and functional outcomes is needed in a large representative population of critically ill patients. We undertook a systematic review and meta-analysis to estimate the clinical relevance and association between the different ICU-Aw diagnostic tests and critical outcomes such as mortality or weaning failure.

**Patients and methods:** We built our systematic review using the Preferred Reported Items for Systematic Review. Literature research was conducted in five databases (PubMed, EMBASE, CINAHL, Cochrane library, Science Direct). Search terms combined keywords relative to three domains: ICU-Aw, diagnostic test of ICU-Aw and outcomes. All observational studies published between January 2000 and December 2018 were included. Randomised controlled trial were excluded. Reviewers abstracted study data by using a standardized form and assessed quality by using Quality in Prognosis Studies tool and the Quality Assessment of Diagnostic Accuracy Studies criteria.

**Results:** 60 studies were analyzed including 4382 patients. 23 studies (1390 patients) performed diaphragm ultrasound, 6 (292 patients) performed transdiaphragmatic twitch pressure and 13 (709 patients) performed maximal inspiratory pressure (MIP). 14 studies (1656 patients) performed MRC score and 4 (335 patients) performed Handgrip test. Overall, ICU-Aw prevalence was 47%. ICU-Aw significantly increased the odds of global mortality (OR to 3.2 95% IC (2.3–4.4); p < 0.0001). The best predictive capacity of global mortality was obtained using the transdiaphragmatic twich pressure with a pooled sensitivity of 0.87 95% IC (0.76–0.93) and a pooled specificity of 0.36 95% IC (0.27–0.43) and an AUC of 0.74 95% IC (0.70–0.78). ICU-Aw was also associated with weaning failure (OR to 3.06 95% IC (2.57–3.63)s; p < 0.001). Diaphragm thickening fraction showed the best predictive capacity for weaning failure with a pooled sensitivity to 0.76 95% IC (0.67–0.83) and a pooled specificity to 0.86 95% IC (0.78–0.92) and an AUC to 0.86 95% IC (0.83–0.89).

**Conclusion:** ICU-Aw is highly prevalent and is a serious problem associated with a higher mortality and a higher mechanical ventilation weaning failure. Respiratory muscle evaluation should be the most important muscular assessment in ICU patients.

**Compliance with ethics regulations:** Yes

### COK-3 Accuracy of diaphragm ultrasound to predict weaning outcome during spontaneous breathing trial: a meta-analysis

#### Aymeric Le Neindre^1^, Francois Philippart^1^, Marta Luperto^2^, Johan Wormser^1^, Johanna Morel^3^, Serge Aho^2^, Silvia Mongodi^4^, Francesco Mojoli^4^, Belaid Bouhemad^2^

##### ^1^Groupe Hospitalier Paris Saint-Joseph, Paris, France; ^2^CHU de Dijon, Dijon, France; ^3^CHU Henri Mondor, Créteil, France; ^4^Fondazione IRCCS Policlinico San Matteo, Pavia, Italy

###### **Correspondence:** Aymeric Le Neindre (aymeric.leneindre@gmail.com)

*Ann. Intensive Care* 2020, **10 (Suppl 1):**COK-3

**Rationale:** Many studies have demonstrated the potential of diaphragm ultrasound (DUS) to predict weaning outcome but no comprehensive conclusion has been reached. The aim of this meta-analysis was to assess diagnostic accuracy of DUS for the prediction of weaning failure in critically ill patients.

**Patients and methods:** PubMed, Science direct, Cochrane Library, CENTRAL and MEDLINE were searched. Three investigators independently selected studies meeting inclusion criteria, extracted data and performed bias analysis using the Quality Assessment of Diagnostic Accuracy Studies-2 instrument. The bivariate model was used to estimate the pooled results of sensitivity, specificity and diagnostic odds ratio. Sources of heterogeneity were explored and Bayesian analysis was used to confirm the estimates.

**Results:** Twenty-three studies with a total were selected and included in the meta-analysis. The pooled sensitivity, specificity and Diagnostic Odd ratio (DOR) of Diaphragm Thickening Fraction were 0.72, 0.88 and 18.82. The Diaphragm Excursion results was lower with pooled sensitivity, specificity and DOR of 0.73, 0.81 and 12.0, respectively. Important heterogeneity was observed among the studies. High risk of bias in selection of patients and conditions of measure was highlighted. A Bayesian analysis confirms the global estimation of diagnostic performance.

**Conclusion:** Diaphragm ultrasound provides good diagnostic performance to predict weaning outcome. Nevertheless, heterogeneity in studies and recent results suggest that DUS may currently be used to explain weaning failure rather than predict weaning outcome.

**Compliance with ethics regulations:** NA

### CO-01 Risk factors for candidemia: a prospective matched case–control study

#### Julien Poissy^1^, Lauro Damonti^2^, Anne Bignon^3^, Nina Khanna^4^, Matthias Von Kietzell^5^, Katia Boggian^5^, Dionysios Neofytos^6^, Fanny Vuotto^7^, Valerie Coiteux^8^, Florent Artru^9^, Stefan Zimmerli^2^, Jean-Luc Pagani^10^, Thierry Calandra^11^, Boualem Sendid^12^, Daniel Poulain^12^, Christian Van Delden^6^, Frédéric Lamoth^13^, Oscar Marchetti^11^, Pierre-Yves Bochud^11^

##### ^1^Intensive Care Department, University hospital of Lille, Lille, France; ^2^Department of Infectious Diseases, Inselspital, Bern University Hospital, University of Bern, Bern, Switzerland; ^3^Surgical Intensive Care Unit. University hospital of Lille, Lille, France; ^4^Division of Infectious Diseases and Hospital Epidemiology, University Hospital of Basel, Basel, Switzerland; ^5^Department of Infectious Diseases, Cantonal Hospital of Saint Gallen, Saint Gallen, Switzerland; ^6^Infectious diseases, University Hospital of Geneva, Geneva, Switzerland; ^7^Infectious Diseases Department, University hospital of Lille, Lille, France; ^8^Hematological Disorders Department, University Hospital of Lille, Lille, France; ^9^Digestive Intensive Care Department, University Hospital of Lille, Lille, France; ^10^Intensive Care Department, University hospital of Lausanne, Lausanne, Switzerland; ^11^Infectious Diseases Department, University Hospital of Lausanne, Lausanne, Switzerland; ^12^Laboratory of Mycology and Parasitology, University Hospital of Lille, Lille, France; ^13^Microbiology Institute, University Hospital of Lausanne, Lausanne, Switzerland

###### **Correspondence:** Julien Poissy (julien_poissy@hotmail.fr)

*Ann. Intensive Care* 2020, **10 (Suppl 1):**CO-01

**Rationale:** Candidemia is an opportunistic infection associated with high morbidity and mortality in hospitalized patients, both inside and outside intensive care units (ICUs). Identification of patients at risk for preemptive approach and early detection is crucial. Prospective control-matched studies and comparison between ICU and non-ICU patients are lacking in this field. We aim to identify and compare specific risk factors in ICU and non-ICU patients.

**Patients and methods:** This was a prospective multicenter matched case–control study assessing risk factors for candidemia and death in candidemic patients, both outside and inside ICUs, from 6 teaching hospitals. Controls were matched to cases based on age, hospitalization ward, hospitalization duration and, when applicable, type of surgery. Risk factors were analyzed by univariate and multivariate conditional regression models, as a basis for a new scoring system to predict candidemia.

**Results:** The study included 192 cases and 411 matched controls. 44% were hospitalized inside ICU and 56% outside. Independent risk factors for candidemia within the ICU population included total parenteral nutrition (TPN) (OR = 6.75, p < 0.001), acute kidney injury (OR = 4.77, p < 0.001), heart disease (OR = 3.78, p = 0.006), previous septic shock (OR = 2.39, p = 0.02) and exposure to aminoglycosides (OR = 2.28, p = 0.05). Independent risk factors for candidemia within the non-ICU population included central venous catheter (CVC) (OR = 9.77, p < 0.001), TPN (OR = 3.29, p = 0.003), exposure to glycopeptides (OR = 3.31, p = 0.04), and to nitroimidazoles (OR = 3.12, p = 0.04).

The weighted scores and their ROC curves are presented in Fig. 1. The weighted ICU-score was as follows: TPN, + 2.5; AKI, + 1.5; heart disease, + 1.5; previous septic shock, + 1.0; aminoglycosides, + 1.0. AUC of the ROC curve was 0.768. The optimal cut-off was ≥ 4 (sensitivity = 69%, specificity = 70%). The best cut-off to optimize specificity was ≥ 5 (sensitivity = 43%, specificity = 88%). The weighted non-ICU score was as follows: CVC, + 2.5; nitroimidazole: + 1.0; TPN, + 1.0; Glycopeptide: + 1.0. AUC of the ROC curve was 0.717. The optimal cut-off was ≥ 2 (sensitivity = 83%, specificity = 50%). The best cut-off to optimize specificity was ≥ 4 (sensitivity = 51%, specificity = 81%).

Independent factors for death in candidemic ICU patients were septic shock (OR = 4.09, p = 0.003), acute kidney injury (OR = 3.45, p = 0.02), the number of antibiotics to which patients were exposed before candidemia (OR = 1.37 per unit, p = 0.02). For non-ICU patients, acute kidney injury (OR = 11.9, p = 0.002) and septic shock (OR = 8.70, p = 0.002) were the only variables significantly associated with death.

**Conclusion:** Risk factors for candidemia differ between ICU and non-ICU settings, including different patterns of antibiotic exposure, leading to different weighted scores predictive of candidemia, with better performances for ICU patients.

**Compliance with ethics regulations:** Yes

**Fig. 1:**
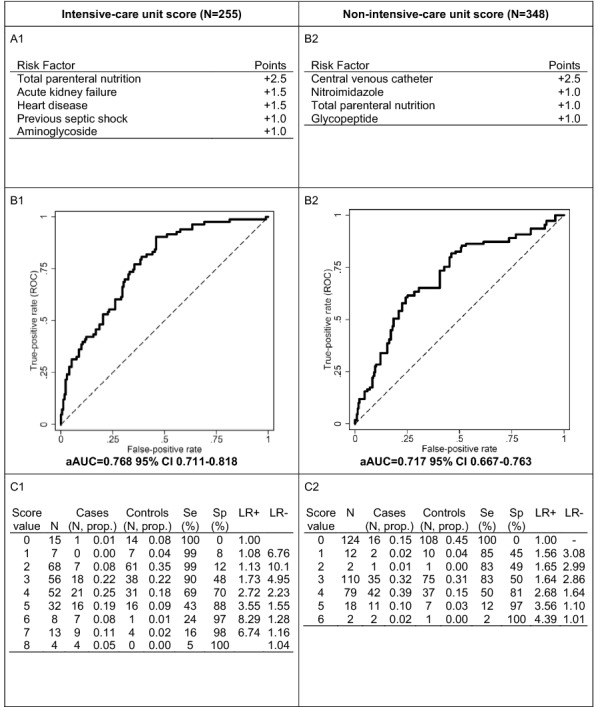
Risk scores for candidemia

### CO-02 A stronger signal of ART-123 efficacy in patients with sepsis associated coagulopathy enrolled into the SCARLET Trial in France

#### Bruno François^1^, Maud Fiancette^2^, Julie Helms^3^, Emmanuelle Mercier^4^, Jean-Baptiste Lascarrou^5^, Xavier Wittebole^6^, Amanda Radford^7^, Toshihiko Kayanoki^7^, Kosuke Tanaka^7^, David Fineberg^7^, Jean-Louis Vincent^8^

##### ^1^Réanimation polyvalente/Inserm CIC 1435/Inserm UMR 1092, CHU Dupuytren, Limoges, France; ^2^Médecine Intensive Réanimation, Centre Hospitalier Départemental Vendée, La Roche-Sur-Yon, France; ^3^Université de Strasbourg (UNISTRA), Faculté de Médecine; Hôpitaux universitaires de Strasbourg, Médecine Intensive-Réanimation, Nouvel Hôpital Civil, Strasbourg, France; ^4^Médecine Intensive Réanimation/CRICS-TRIGGERSEP, CHRU de Tours, Tours, France; ^5^Service de Médecine Intensive Réanimation, CHU de Nantes, Nantes, France; ^6^Department of Critical Care Medicine, Clinique universitaire St Luc, Université Catholique de Louvain, Brussels, Belgium; ^7^Asahi Kasei Pharma America, Waltham, UNITED STATES; ^8^Department of Intensive Care, Erasme Hospital, Universite Libre de Bruxelles, Brussels, Belgium

###### **Correspondence:** Bruno François (b.francois@unilim.fr)

*Ann. Intensive Care* 2020, **10 (Suppl 1):**CO-02

**Rationale:** To assess the effect of recombinant human soluble thrombomodulin (ART-123) treatment on 28-day all-cause mortality in patients with sepsis associated coagulopathy (SAC) enrolled in France, the highest enrolling country, as compared to the global population of patients in the randomized, double-blinded, placebo-controlled, SCARLET phase 3 study (NCT01598831), and especially to evaluate factors that may have contributed to greater mortality reduction in the French patients.

**Patients and methods:** Among the global patient population with SAC defined as an international normalized ratio (INR) > 1.40, platelet count > 30– < 150 × 109 or > 30% decrease in 24 h, and with concomitant cardiovascular and/or respiratory failure, patients in France were analyzed. All subjects were treated with 0.06 mg/kg/day of intravenous ART-123 (n = 75/395) or placebo (n = 74/405) for up to 6 days in addition to standard of care.

**Results:** In the SCARLET trial, ART-123 did not significantly reduce 28-day all-cause mortality (800 patients from 149 sites in 26 countries) with an absolute risk reduction (ARR) of 2.55% (P = 0.32) in ART-123 treated patients with SAC, and 5.4% in those patients who maintained protocol specified coagulopathy (INR > 1.40, platelet count > 30 × 109) at baseline prior to study drug dosing. In France, there were 149/800 (18.6%) patients enrolled at 16/149 (10.7%) sites. Considering all randomized and dosed patients in France as compared to those patients that maintained protocol specified coagulopathy, the 28-day ARR for ART-123 was higher in France than for the global population. Patients enrolled in France were more likely to have the protocol specified coagulopathy criteria at baseline than in other countries. They also had overall more severe baseline disease as measured by the number of patients with at least 3 organ dysfunctions (cardiovascular, respiratory, renal, and hepatic) and a higher APACHE II score than the other patients. However, they were less likely to be treated by renal replacement therapy (RRT) or to receive heparin at baseline. In France there was a greater proportion of enrollment from individual sites enrolling 6 patients or more.

**Conclusion:** The trend towards a higher difference of mortality benefit in patients treated with ART-123 and enrolled in France may be attributed to better patient selection, and a higher rate of patients meeting the protocol specified coagulopathy criteria at baseline by primarily high enrolling research sites.

**Compliance with ethics regulations:** Yes

### CO-03 Use and impact of aminoglycoside empirical therapy in extended spectrum beta-lactamase enterobacteriaceae bloodstream infections in intensive care unit

#### Lucie Benetazzo, Pierre-Yves Delannoy, Olivier Leroy, Olivier Robineau, Agnes Meybeck

##### CH Tourcoing, Tourcoing, France

###### **Correspondence:** Lucie Benetazzo (lucie.benetazzo@gmail.com)

*Ann. Intensive Care* 2020, **10 (Suppl 1):**CO-03

**Rationale:** Aminoglycosides are prescribed in severe infections for bactericidal activity and broadening of the spectrum. As the prevalence of extended-spectrum beta-lactamase-producing enterobacteriaceae (ESBLE) increases, aminoglycosides may be interesting for their treatment. However, their use is limited by their toxicity, especially renal. Our study evaluated the impact of an aminoglycoside in empirical treatment of ESBLE bloodstream infection in intensive care unit (ICU).

**Patients and methods:** Between January 2011 and September 2017, patients treated for ESBLE bacteraemia in the ICU of 5 French hospitals from Hauts de France were included in a retrospective observational cohort study. In order to evaluate the impact of the empirical prescription of an aminoglycoside, a bivariate and multivariate analysis were performed. The primary endpoint was mortality on day 30. Secondary endpoints were empirical antibiotic therapy adequacy and renal failure rates.

**Results:** Three hundred and seven patients were included, 169 received an aminoglycoside as initial treatment. The death rate at day 30 was 40%. We did not find any difference in mortality between aminoglycoside and non-aminoglycoside group (43.4% vs. 39.3%, p = 0.545). Renal impairment occurred in aminoglycoside and non-aminoglycoside groups in 20.7% and 23.9%, respectively (p = 0.59). The adequacy rate of empirical antibiotic therapy was higher in the aminoglycoside group (91.7% vs. 77%, p = 0.001). An age greater than 70 years, a history of transplantation, or the nosocomial origin of bacteraemia were associated with mortality at day 30. Maintenance of amines more than 48 h after bacteraemia, occurrence of ARDS or acute renal failure also increased mortality on day 30.

**Conclusion:** Our study did not show any impact of aminoglycoside empirical prescription for the treatment of ESBLE bacteraemia even if it led to an increase in the adequacy rate of empirical therapy. We did not find any renal toxicity caused by aminoglycosides.

**Compliance with ethics regulations:** Yes

### CO-04 Impact of a restrictive antibiotic policy on the emergence of extended-spectrum ß-lactamase producing Enterobacteriaceae (ESBL-E) in the ICU. A quasi-experimental observational study

#### Christophe Le Terrier^1^, Marco Vinetti^1^, Régine Richard^1^, Bruno Jarrige^2^, Sébastien Breurec^3^, Michel Carles^1^, Guillaume Thiery^1^

##### ^1^CHU Guadeloupe Intensive Care Unit, Pointe-À-Pitre, France; ^2^CHU Guadeloupe Hospital Infection Control Department, Pointe-À-Pitre, France; ^3^CHU Guadeloupe Microbiology unit, Pointe-À-Pitre, France

###### **Correspondence:** Christophe Le Terrier (christophe.clt@orange.fr)

*Ann. Intensive Care* 2020, **10 (Suppl 1):**CO-04

**Rationale:** Massive consumption of antibiotics in the intensive care unit (ICU) is a major determinant of extended-spectrum beta-lactamase–producing Enterobacteriaceae (ESBL-E) spreading. We evaluated whether a stewardship program including restrictive antibiotic policy in the ICU would reduce ESBL-E emergence without worsening patients’ outcomes.

**Patients and methods:** We conducted an observational quasi-experimental pre-post intervention study of all consecutive patients with length of stay (LOS) superior to 48 h in the medical-surgical ICU of University Hospital of Guadeloupe. From January 1, 2014 to December 31, 2014, a liberal strategy was used including a broad-spectrum antibiotic as initial empirical treatment in case of sepsis or suspected infection, followed by de-escalation after 48–72 h. From January 1, 2015 to December 31, 2015, a restrictive strategy was adopted which consisted of limitation of broad-spectrum antibiotics, avoidance of antibiotics targeting anaerobic microbiota and shortening of antibiotic duration. In addition, antibiotic therapy was initiated only after microbiological identification, except in cases of septic shock, acute respiratory distress syndrome and meningitis, where an empiric therapy was started immediately after the microbiological samples were taken. Our primary outcome was the incidence of ICU-acquired ESBL-E and the main secondary outcome were all-cause ICU mortality and the rate of ESBL-E infections.

**Results:** 1009 and 1067 patients were admitted to ICU during the liberal and the restrictive strategy period of the study respectively. Among them, 767 and 826 patients were hospitalized > 48 h and were enrolled in the study (Table 1). During the restrictive strategy period, less patients were treated with antibiotic therapy (41 vs 52%; p < 0.001), treatment duration was shorter (5 vs 6 days; p = 0.01) and antibiotics targeting anaerobic pathogens were significantly less administrated (87.1% vs 37.5%; p < 0.0001). The rate of ICU-acquired ESBL-E carriage was significantly lower during the restrictive strategy period (18.9% vs 11.1%; p < 0.0001). Similarly, ICU-acquired ESBL-E infection rate and ICU mortality were lower during the restrictive strategy period. In multivariate analysis, the length of stay in the ICU, the number of antibiotic adminsitered and the restrictive strategy period were independently associated with a lower rate of ESBL-E acquisition.

**Conclusion**: In a large cohort of consecutive ICU patients, a stewardship program including a restrictive antibiotic strategy has proven effective in terms of reduction of antibiotic consumption, especially broad spectrum antibiotics and those targeting anaerobic microbiota. This strategy was associated with a lower rate of ESBL-E acquisition without worsening patients’ outcomes.

**Compliance with ethics regulations:** Yes.

**Table 1:** Demographic characteristics, outcomes and antibiotics used

	2014	2015	p
N patients	1009	1067	
N patients with ICU stay > 48 h	767 (76.0%)	826 (77.4%)	0.45
Age (years)	55.5 (19.9%)	54.7 (19.6%)	0.38
SAPS II mean ± SD	42 ± 24	40 ± 25	0.01
All patient’s ICU length of stay > 48 h	n = 767	n = 826	
SAPS II mean ± SD	42 ± 21	40 ± 21	0.08
Sepsis (community or acquired)	374 (48.8%)	320 (38.7%)	< 0.0001
Catecholamines administrated in septic patients	170 (22.2%)	151 (18.3%)	0.06
Primary outcome			
ICU-acquired ESBL-E	145 (18.9%)	92 (11.1%)	< 0.0001
Secondary outcomes			
ESBL-E infections	60 (7.8%)	37 (4.5%)	0.005
All-cause ICU mortality	215 (28.0%)	184 (22.3%)	0.01
All-cause hospital mortality	256 (33.4%)	226 (27.4%)	0.01
ICU length of stay, days median ± IQR	6 [4–12]	5 [4–10]	0.02
Patients who did not receive antibiotic therapy	332 (43.3%)	440 (53.5%)	< 0.0001
Number of patients receiving antibiotics	n = 435	n = 386	< 0.0001
Antibiotics administrated			
Amoxicillin	29 (6.7%)	67 (17.6%)	< 0.0001
Amoxicillin/ clavulanic acid	122 (88.0%)	69 (17.9%)	< 0.0001
Piperacillin/tazobactam	169 (38.9%)	18 (4.7%)	< 0.0001
Cefotaxime/Ceftriaxone (C3G)	196 (45.1%)	162 (42%)	0.37
Cefoxitin (cephamycin)	6 (1.4%)	34 (8.8%)	< 0.0001
Ceftazidime	19 (4.4%)	40 (10.4%)	< 0.0001
Cefepim	7 (1.6%)	18 (4.3%)	0.01
Carbapenem	54 (12.4%)	14 (4.7%)	< 0.0001
Antibiotics targeting anaerobic pathogens*	379 (87.1%)	171 (37.5%)	< 0.0001

*Abbreviations:* SAPS II: Simplified Acute Physiology Score II; ICU: Intensive care unit; ESBL-E: extended-spectrum beta-lactamase-producing *Enterobacteriaceae;* SD: standard deviation

C1G: 1^st^ generation cephalosporin; C2G: 2^nd^ generation cephalosporin; C3G: 3^rd^ generation cephalosporin

^*^Amoxicillin/Clavulanic acid, Piperacillin/Tazobactam, Carbapenem, Cefoxitin, Clindamycin, Metronidazole

# when not specified, results are n (%). In bold, *p* values < 0.05.

### CO-05 Awake venoarterial extracorporeal membrane oxygenation in cardiogenic shock: a propensity score matched analysis

#### Santiago Montero^1^, Florent Huang^2^, Juliette Chommeloux^2^, Nicolas Brechot^2^, Pierre Demondion^3^, Guillaume Franchineau^2^, Guillaume Hekimian^2^, Romain Persichini^4^, Charles-Edouard Luyt^2^, Guillaume Lebreton^3^, Alain Combes^2^, Matthieu Schmidt^2^

##### ^1^Hospital Germans Trias i Pujol, Acute Cardiovascular Care Unit, Cardiology, Universitat Autònoma de Barcelona, Barcelona, Spain; ^2^Assistance Publique–Hôpitaux de Paris, Pitié–Salpêtrière Hospital, Medical Intensive Care Unit, 75651 Paris Cedex 13, France; ^3^Assistance Publique–Hôpitaux de Paris, Pitié–Salpêtrière Hospital, Thoracic and Cardiovascular department, 75651 Paris Cedex 13, France; ^4^Medical–Surgical Intensive Care Unit, CHU de La Réunion, Felix-Guyon Hospital, Saint Denis, La Réunion, France

###### **Correspondence:** Santiago Montero (monteroaradas@gmail.com)

*Ann. Intensive Care* 2020, **10 (Suppl 1):**CO-05

**Rationale:** Venoarterial extracorporeal membrane oxygenation (VA-ECMO) is the first-line therapy for refractory cardiogenic shock (CS), but its applicability is undermined by the high morbidity associated with its complications, especially those related to mechanical ventilation (MV). We aimed at assessing the impact on survival of keeping patients awake during the VA-ECMO run in the context of refractory CS.

**Patients and methods:** A 7-year database of CS-patients supported with peripheral VA-ECMO was used to perform a propensity score (PS) matched analysis in order to balance their clinical profile. Patients were classified as “awake and partially awake” or “non-awake” if mechanical ventilation was present ≤ 50% or > 50% of the ECMO run. Primary outcomes were 60-day and 1-year mortality, and secondary outcomes included rates of ventilator-associated pneumonia (VAP) and ECMO-related complications. A multivariate logistic regression analysis was performed to identify if respiratory status at cannulation was independently associated with 60-day mortality.

**Results:** Out of 231 patients included, 91 (39%) were “awake and partially awake” and 140 (61%) “non-awake”. After PS matching adjustment, the “awake and partially awake” group had significantly better 60-day (19% vs 46%, p < 0.006; 95% CI OR 0.36 [0.17–0.79], p = 0.01) and 1-year survival (32% vs 57%, p < 0.018; 95% CI OR 0.43 [0.22–0.82], p = 0.01) compared to the “non-awake” group, as well as reduced rates of VAP (34% vs 64%, p = 0.004) and less antibiotic and sedative drugs consumption. However, MV at ECMO cannulation was not independently related to 60-day mortality.

**Discussion:** Previous clinical series have suggested better survival and less VAP rates in ECLS-supported patients, but the small number of patients and the inclusion of non-arousable patients with likely neurological impairment hampered the interpretation and validity of this strategy. Although the likelihood of remaining awake seem to increase in vigil patients at cannulation, we did not find an independent association between this fact and 60-day mortality. Therefore, it is likely that the earliest possible extubation represents the main message in this setting, allowing to longer time on spontaneous breathing whilst on VA-ECMO support. Beyond better outcome, physical rehabilitation, communication with relatives and, specifically, interactive information of the medical decisions, are key potential benefits from a non-intubation or an early-extubation management in these patients. Indeed, the avoidance of relevant complications (mainly VAP) may additionally contribute to these advantages.

**Conclusion:** An “awake and partially awake” VA-ECMO strategy in CS is safe and is associated with improved short- and long-term survival compared to mechanically ventilated patients.

**Compliance with ethics regulations:** Yes.

**Fig. 1:**
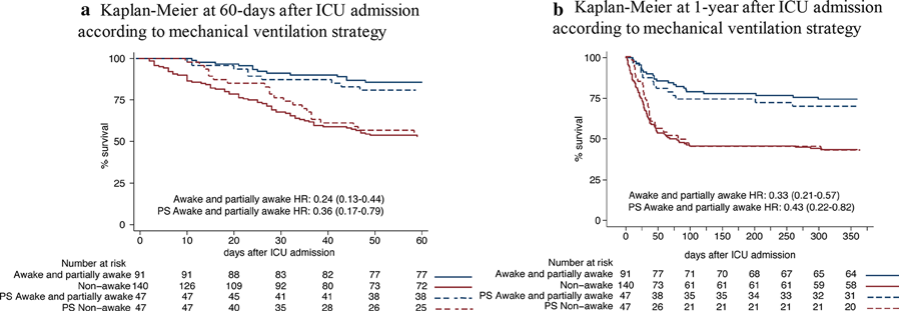
Kaplan-Meier curves at 60-day and 1-year according to mechanical ventilation strategy

### CO-06 Impact of pulmonary hypertension on post heart transplant outcome

#### Cyrielle Desnos^1^, Guillaume Coutance^1^, Mathieu Kerneis^1^, Amandine Baptiste^2^, Guillaume Lebreton^3^, Charles-Edouard Luyt^4^, Alain Combes^4^, Shaida Varnous^1^, Nicolas Brechot^4^

##### ^1^Cardiology department, La Pitié Salpêtrière hospital, Paris, France; ^2^Biostatistics and public health department, La Pitié Salpêtrière hospital, Paris, France; ^3^Cardio-thoracic surgery department, La Pitié Salpêtrière hospital, Paris, France; ^4^Intensive care unit, Cardiology institute, La Pitié Salpêtrière hospital, Paris, France

###### **Correspondence:** Cyrielle Desnos (cyrielle.desnos@wanadoo.f)

*Ann. Intensive Care* 2020, **10 (Suppl 1):**CO-06

**Rationale:** Pulmonary hypertension (PH) is associated with a higher risk of early right ventricular graft failure after heart transplantation, and poorer short and long-term outcomes. However, improvements in short term extra corporeal life support in the past decades allows an easier supply of early graft failure. Thereby, we aimed to compare the outcome of patients undergoing heart transplantation with or without elevated pulmonary vascular resistances (PVR), in an expert center for mechanical circulatory support.

**Patients and methods:** We conducted a retrospective monocentric cohort study including all consecutive patients receiving a heart transplant in our center from 2011 to 2017, with an assessment by right heart catheterization in the year before transplantation.

**Results:** Among the 304 patients included, 129 (42%) had low PVR (PVR ≤ 2.5 WU), and 175 (58%) had high PVR (PVR > 2.5 WU) before transplant. 82% of patients with high PVR and 81% of patients with low PVR were alive at 1 year follow-up (p = 0.6). Patients with high PVR were more likely to require immediate post-transplant circulatory support by veno-arterial extracorporeal membrane oxygenation (VA-ECMO) (58% vs 44%, p = 0.02), as well as a pulmonary vasodilator treatment by Sildenafil (14% vs 7%, p = 0.045). PVR were not associated with 1-year post-transplant mortality in multivariate analysis. At 1-year follow-up, right and left ventricular graft function were preserved for all survivors, and did not differ between high and low PVR groups. Even in the subgroup of the 24 patients transplanted with PVR > 5WU, unresponsive to vasodilator challenge, 1-year survival was 92%, with preserved right and left ventricular function.

**Conclusion:** PH was not associated with a poor outcome in our cohort, even when it was severe and unresponsive to vasodilator challenge. However, the rate of VA-ECMO support immediately after heart transplant was higher in patients with high PVR. Cardiac transplantation as first line strategy may be a valuable option in patients with elevated pre-transplant PVR.

**Compliance with ethics regulations:** Yes.

### CO-07 Hemodynamic and microcirculation evaluation of Vasopressin versus Norepinephrine in a porcine model of refractory cardiac arrest resuscitated by venous-arterial ECMO

#### Thomas Klein^1^, Caroline Fritz^1^, Daniel Grandmougin^2^, Yihua Liu^2^, Sophie Orlowski^3^, Tran N’guyen^4^, Eliane Albuisson^5^, Bruno Lévy^1^

##### ^1^Service de Réanimation Médicale Brabois, CHRU Nancy, Pôle Cardio-Médico-Chirurgical, INSERM U1116 Vandoeuvre-Les-Nancy, France; ^2^Service de Chirurgie Cardiaque Brabois, CHRU Nancy, Pôle Cardio-Médico-Chirurgicale, INSERM U1116, Vandoeuvre-Les-Nancy, France; ^3^Service de Biochimie, Pôle Laboratoires, CHRU Nancy Central, Nancy, France; ^4^Ecole de chirurgie, Faculte de Médecine, Université de Lorraine, Vandoeuvre-Les-Nancy, France; ^5^Plateforme d’aide à la recherche clinique (PARC-UMDS), Hôpital de Brabois, CHRU Nancy, Vandoeuvre-Les-Nancy, France

###### **Correspondence:** Thomas Klein (thomas090@outlook.fr)

*Ann. Intensive Care* 2020, **10 (Suppl 1):**CO-07

**Rationale:** Venoarterial extracorporeal membrane oxygenation (VA-ECMO) is used to support tissue perfusion during extracorporeal cardiopulmonary resuscitation (e-CPR). Shock, resuscitation and the extracorporeal circuit may trigger a capillary leakage and a vasoplegic shock. Currently, in these situations, high doses of Norepinephrine (NE) are required. Because high NE doses may have significant cardiovascular side effects, alternative options to support arterial blood pressure are needed. In recent years, several approaches to decrease the administration of high NE doses have been tested, one of them is the administration of Vasopressin (AVP). Randomized trials have shown that AVP infusion increases arterial pressure and systemic vascular resistance, decreases catecholamine requirements in patients with or at high risk of vasoplegic syndrome and attenuates vascular dysfunction. Currently, no data are available for the study of the effects of AVP in shock state in post refractory cardiac arrest.

**Patients and methods:** 20 pigs were randomized into two groups, in order to receive AVP or NE. A refractory cardiac arrest of ischemic origin was surgically created and VA-ECMO was started after a 30 min period of cardio-pulmonary resuscitation. Then, resuscitation lasted 6 h in each randomization group. The evolution of the consequences of the shock was evaluated by lactatemia and microcirculation (SDF and NIRS) at baseline hour, H0 (when ECMO starts), H3 and H6. Renal and hepatic functions were assessed.

**Results:** Experimental conditions were met for 16 animals (AVP, n = 8; NE, n = 8). The groups were comparable on the shock impact and its severity. No significant differences were found between populations for ECMO flow and MAP. There was a significant difference on fluid volume resuscitation amount (14000 [11.250–15.250] mL in the NE group versus 3500 [1750–4000] mL in the AVP group, p < 0.05) (Fig. 1). No significant difference between the NE and AVP groups for lactate clearance between H0 and H6 (25.6 [− 7.31 to 35.34]% vs 47.84 [13.42–82.73]%, p = 0.686). We did not find any significant for sublingual microcirculation indices and NIRS values. Renal and liver function evolution were similar in the two groups during the protocol.

**Conclusion:** AVP administration in refractory cardiac arrest resuscitated by VA-ECMO when compared to NE is associated with less fluid volume for similar global and regional hemodynamic effects.

**Compliance with ethics regulations:** Yes.

**Fig. 1:**
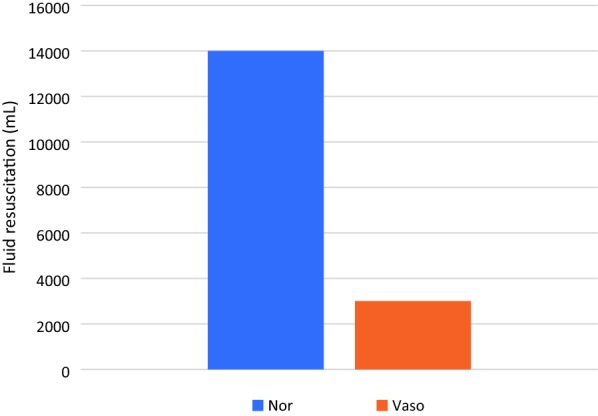
Fluid volume resuscitation in Vasopressin group compared to Norepinephrine group

### CO-08 Veno-arterial Extracorporeal Membrane Oxygenation flow or Dobutamine to increase microcirculation for Refractory Cardiogenic Shock

#### Juliette Chommeloux^1^, Santiago Montero^2^, Guillaume Franchineau^1^, Alain Combes^1^, Matthieu Schmidt^1^

##### ^1^Assistance Publique–Hôpitaux de Paris, Pitié–Salpêtrière Hospital, Medical Intensive Care Unit, 75651 Paris Cedex 13, France; ^2^Acute and intensive cardiovascular care unit, department of cardiology, hospital de la Santa Creu i Sant Pau, Barcelona, Spain

###### **Correspondence:** Juliette Chommeloux (juliette.chommeloux@gmail.com)

*Ann. Intensive Care* 2020, **10 (Suppl 1):**CO-08

**Rationale:** Venoarterial extracorporeal membrane oxygenation (VA-ECMO) is an effective technique to support refractory cardiogenic shock (CS) and increase macro- and microcirculation. Given that major and persistent microcirculation alterations are associated with worse outcome, we investigated the respective impact of an increase of either the VA-ECMO flow and or dobutamine dose on microcirculation in stabilized patients with refractory CS on VA-ECMO.

**Patients and methods:** Prospective study in academic medical intensive unit. Consecutive patients with ECMO-supported CS instability, who were able to tolerate a stepwise increment of the dobutamine dose and the ECMO flow. Baseline was defined by the lowest VA-ECMO flow and dobutamine 5 µg/kg/min for PAM ≥ 65 mmHg. Starting from the baseline, VA-ECMO flow was progressively increased by 25% (ECMO125%, ECMO150%, ECMO175%, ECMO200%). Back to baseline, a stepwise increase of the dobutamine to 10, 15 and 20 µg/kg/min (DOBU10, DOBU15, DOBU20) was performed. Macro- and microcirculatory evaluations were made after 30 min in each condition.

**Results:** Fourteen patients (median age 52 [40–61] years; SAPS II 68 [52–76]) were included. Acute myocardial infarction was the main cause of cardiogenic shock (64%). Macro- and microcirculation were assessed 2 [2–5] days after ECMO start. The increment of the dobutamine dose did not modify microcirculation parameters. De Backer score tended to be reduced (p = 0.08), with a significant mean arterial pressure (MAP) increase during the ECMO flow increment. These findings were not different between patients successfully weaned-off ECMO (n = 6) and those who did not.

**Conclusion:** When macro and microcirculation are already restored on ECMO-supported refractory CS, increasing dobutamine (above 5 µg/kg/min) or ECMO flow did not further improve microcirculation. For now, ECMO flow and dobutamine should be set as the minimum flow to get a MAP ≥ 65 mmHg and lactate < 2.5 mmol/L, and the minimal dose to maintain aortic valve opened, respectively.

**Compliance with ethics regulations:** Yes.

**Fig. 1:**
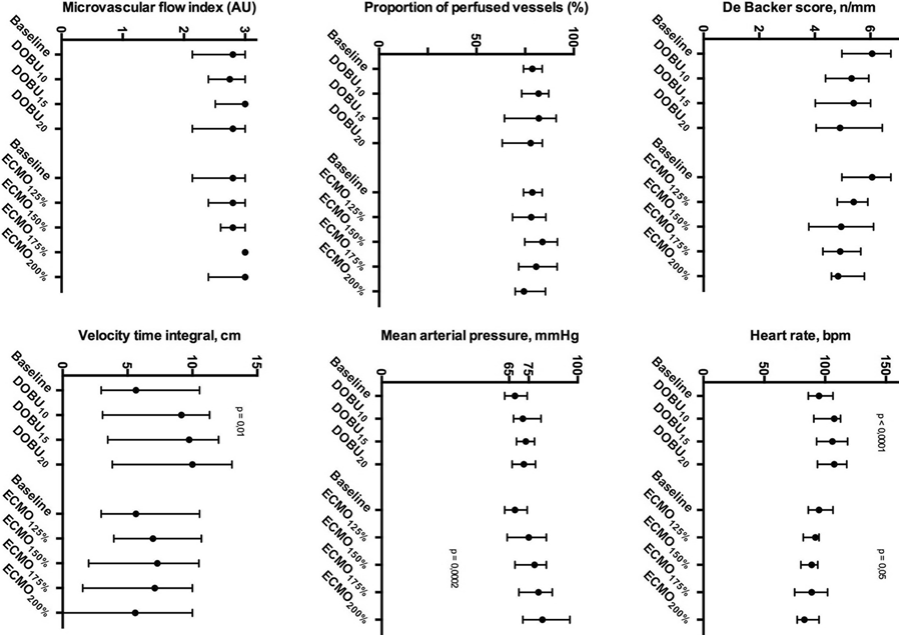
Bar plots of macro- and microcirculation-parameters’ trends during a progressive increment of either the dobutamine dose or the ECMO flow. The point corresponds to the median, the T-bars represent the 25th and 75th percentile, p shows the effect of a progressive increment of either the dobutamine or the ECMO flow

### CO-09 Weaning from noninvasive ventilation and high flow nasal cannula in patients with severe bronchiolitis

#### Julie Cassibba^1^, Anne Ego^2^, Isabelle Pin^1^, Guillaume Mortamet^3^

##### Service de pédiatrie, CHU de Grenoble, Grenoble, France; ^2^Département de santé publique, CHU de Grenoble, Grenoble, France; ^3^Service de réanimation pédiatrique, CHU de Grenoble, Grenoble, France

###### **Correspondence:** Julie Cassibba (jcassibba@chu-grenoble.fr)

*Ann. Intensive Care* 2020, **10 (Suppl 1):**CO-09

**Rationale:** Non Invasive Ventilation (NIV) and High Flow Nasal Cannula (HFNC) are the first-line therapies for the most severe patients with acute bronchiolitis. Unlike invasive mechanical ventilation, there is no consensus with regards to weaning from NIV/HFNC. The main objective of this study is to describe the weaning practices from NIV/HFNC in patients with acute bronchiolitis.

**Patients and methods:** A single-center prospective study. Patients younger than 6 months with severe bronchiolitis and supported by NIV or HFNC were included. NIV/HFNC was discontinued according to the local practices and no protocol existed. Exceptt the principal investigator, the attending team was blinded to the study. Weaning failure was defined as the need to reinstate NIV/HFNC in the 48 h after discontinuation. Ethical approval was not necessary for this study in accordance with the French data protection autority methodology reference number MR-004.

**Results:** A total of 95 patients (median age 47 days, 53 (56%) males) were included. Respectively, 72 (76%) and 23 patients (24%) were supported by NIV and HFNC at admission (Fig. 1). Regarding the mode of NIV, a bilevel mode was used in 46 patients (48%) (Fig. 1). In patients supported by HFNC, the ventilatory support was discontinued progressively by decreasing air flow in 9 patients (39%) while it was stopped abruptly in 5 (22%). In patients supported by NIV, the respiratory support was stopped abruptly in 5 (19%) of them while HFNC was used as a weaning method for 17 (65%) patients. A total of 22 (23%) patients experienced a weaning failure. Patients supported by NIV/HFNC who experienced a prompt weaning had a lower Pediatric Intensive Care Unit (PICU) length of stay as compared to patients in whom HFNC was used as a weaning method (78 ± 27 h versus 112 ± 112 h, p = 0.01). However, the hospital length of stay was similar according to the weaning method (6 ± 3 days versus 7 ± 3 days for prompt and progressive methods respectively, p = 0.07). The duration of the weaning process did not differ according to the bed-availability in PICU.

**Conclusion:** In patients with severe bronchiolitis, a prompt weaning from NIV/HFNC was associated with a lower length of stay in PICU. However, the hospital length of stay was similar according to the weaning method. We suggest that a prompt weaning should be preferred in order to reduce the risk of PICU related complications.

**Compliance with ethics regulations:** Yes.

**Fig. 1:**
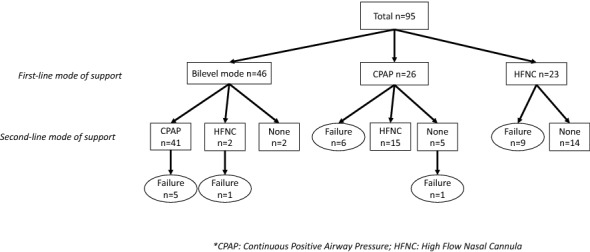
Flow chart of the study

### CO-10 Influenza-associated encephalitis: a French multicentric retrospective study in pediatric intensive care units

#### Pierre CLEUZIOU^1^, Florence RENALDO^1^, Sylvain RENOLLEAU^2^, Isabelle DESGUERRE^2^, Etienne JAVOUHEY^3^, Pierre TISSIERES^4^, Pierre-Louis LEGER^5^, Stéphane DAUGER^1^, Michael LEVY^1^

##### ^1^Hôpital Robert Debré-APHP, Paris, France; ^2^Hôpital Necker Enfants Malades-APHP, Paris, France; ^3^Hôpitaux de Lyon, Lyon, France; ^4^Hôpital du Kremlin-Bicêtre-APHP, Le Kremlin Bicêtre, France; ^5^Chef de service de la réanimaton pédiatrique de l’hôpital Trousseau, Paris, France

###### **Correspondence:** Pierre Cleuziou (pierre.cleu@gmail.com)

*Ann. Intensive Care* 2020, **10 (Suppl 1):**CO-10

**Rationale:** Influenza-associated encephalitis (IAE) is a very rare disease with poorly understood pathophysiology. It has mainly been described in Japan, where the death rate is around 8% and where one-third of the survivors suffer from neurological sequelae. The objective of this study was to describe severe forms of the disease among children hospitalized in pediatric intensive care unit (PICU) and to estimate the death rate in this population.

**Patients and methods:** By using consensus definition criteria, we retrospectively identified children hospitalized between 2010 and 2018 in 12 french PICU for an encephalitis associated with a laboratory-proven acute infection to Influenzae virus. Patients with preexisting neurological chronic disorder or presenting a co-infection potentially responsible of the disease were excluded. We collected data describing clinical presentation, cerebro-spinal fluid (CSF) results, electroencephalographic and MRI findings, therapeutics used in PICU and outcome at discharge.

**Results:** 41 patients were included with 4.7 years old as median age (range 0.8–15.4 years old). Most of the patients were admitted in ICU less than 48 h after the first symptoms (62%, n = 25). The main clinical features were fever (93%, n = 38), vomiting (44%, n = 18), altered consciousness (100%, n = 41), epileptic seizures (88%, n = 36), status epilepticus (54%, n = 22) and motor weakness or pyramidal signs (71%, n = 29). 48% of patients had meningitis (n = 16) and the virus was never found in the CSF (n = 0/13). One-third of children (n = 13) presented MRI lesions compatible with acute necrotizing encephalitis. Regarding therapeutics, 80% of patients required mechanical ventilation, especially for neurologic dysfunction, and the use of specific treatments was very heterogeneous: 68% had oseltamivir, 49% boluses of corticosteroids, 24% intravenous immunoglobulins and 10% plasma exchanges. The median length of stay (LOS) in PICU was 7 days (range 1–87 days) and there were 7 fatalities (17%). Among survivors, 35% had severe neurological sequelae at discharge from the hospital (n = 11). A predisposing mutation in the RANBP2 gene was rarely sought (15%, n = 6) but was positive in one out of two patients.

**Conclusion:** Patients requiring PICU for an IAE still have an extremely severe prognosis with a high mortality rate and frequent neurological sequelae. It appears that patients’ therapeutic management is still heterogeneous because of the lack of consensual guidelines. The research of a predisposing genetic mutation in RANBP2 is not yet part of the systematic etiologic assessment although it is known to be an important risk factor for the severe form of the disease.

**Compliance with ethics regulations:** Yes.

### CO-11 Population pharmacokinetics of Cefazolin in critically ill children infected with methicillin-sensitive *Staphylococcus aureus*

#### Elodie Salvador, Mehdi Oualha, Emmanuelle Bille, Olivier Bustarret, Agathe Béranger, Guillaume Geslain, Florence Moulin, Julie Toubiana, Sihem Benaboud, Sylvain Renolleau, Jean-Marc Tréluyer, Déborah Hirt

##### Necker Hospital, Paris, France

###### **Correspondence:** Elodie Salvador (elodie.salvador@aphp.fr)

*Ann. Intensive Care* 2020, **10 (Suppl 1):**CO-11

**Rationale:** Cefazolin is one of curative treatments for infections with methicillin-sensitive *Staphylococcus aureus* (MSSA) which may occur in critically ill children. Both growth and critical illness may impact of pharmacokinetics (PK) in this vulnerable population. We aim to establish a PK model for cefazolin, using a population approach, and in turn to optimize individual dosing regimens.

**Patients and methods:** We included all children (age < 18 years, bodyweight (BW) > 2.5 kg) receiving cefazolin and infected with MSSA. Cefazolin serum total concentrations were quantified by high-performance liquid chromatography. Data modelling process has been done with a non-linear mixed-effect modeling software MONOLIX. Monte Carlo simulations were used to optimize individual dosing regimens in order to attain the target of 100% [fT (4xMIC)].

**Results:** Thirty-nine patients with a median age of 7 years (0.1–17), a body weight (BW) of 21 kg (2.8–79) and an estimated glomerular filtration rate (eGFR) of 189 mL/min/1.73 m^2^ (66–486) were included. The PK was ascribed a one-compartment model with first-order elimination, where clearance and volume of distribution estimated were 1.4 L/h and 3.3 L respectively, normalized to a median subject of 21 kg and eGFR of 189 mL/min/1.73 m^2^. BW, according to the allometric rules, and eGFR were the significant covariates. Under simulations, continuous infusion with a dose of 100 mg/kg/day was the best scheme to reach the target of 100% [fT (4xMIC)] (Fig. 1). A dose of 150 mg/kg per day by continuous infusion is more appropriate for children with BW < 10 kg or eGFR > 200 mL/min, while also limiting side effects.

**Conclusion:** In critically ill children infected with MSSA, BW with allometric scaling and eGFR were the main influential covariates on cefazolin PK parameters. Current and recommended dosing regimens of cefazolin may be not sufficient to reach the target of 100% [fT (4xMIC)] in all children. Continuous infusion with a dosing of 100 to 150 mg/kg/day seems to be the best scheme to achieve the target of 100% [fT (4xMIC)] in children with normal and augmented renal function, respectively.

**Compliance with ethics regulations:** Yes.

**Fig. 1:**
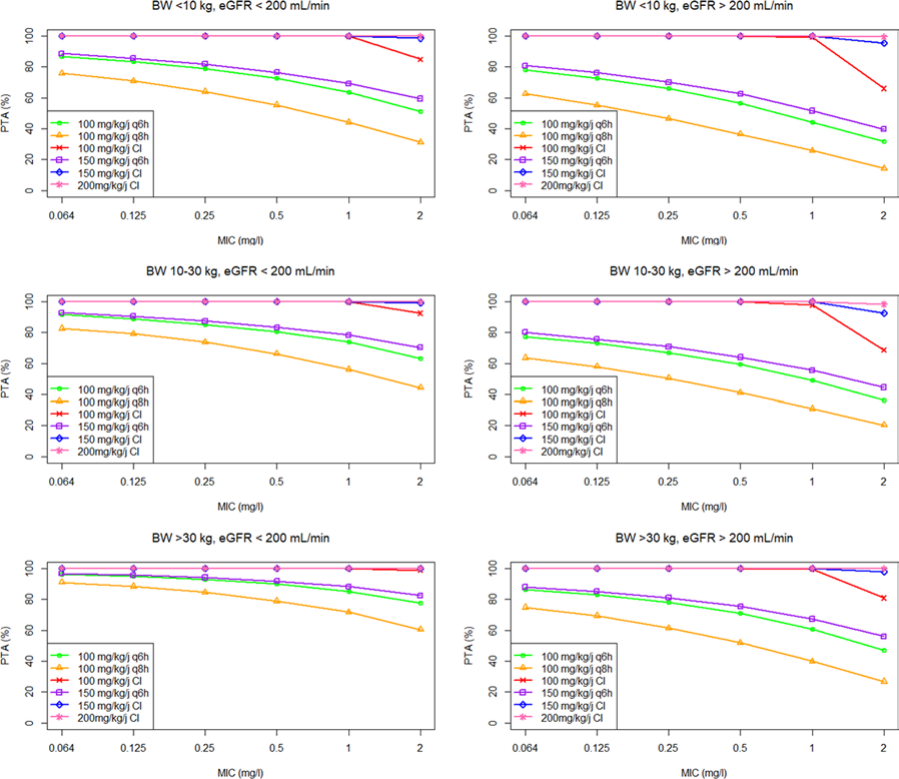
Probability of target attainment obtained on 39,000 simulations from study population for a target defined as 100% 〖fT〗_ (4× MIC.) BW, body weight (kg); eGFR, estimated glomerular filtration (mL/min); PTA, probability of target attainment (%); MIC, minimum inhibitory concentration (mg/L); q6h, administration every 6 h; q8h, administration every 8 h; CI, continuous infusion

### CO-12 Factors influencing unplanned extubations in a pediatric intensive care unit: a 9-year prospective study

#### Guillaume Geslain, Jeanne Mayeur, Michael Levy, Géraldine Poncelet, Fleur Le Bourgeois, Arielle Maroni, Camille Dollat, Jérôme Naudin, Stéphane Dauger, Maryline Chomton, Anna Deho

##### Pediatric Intensive Care Unit, Assistance Publique-Hôpitaux de Paris, Robert Debre University Hospital, Université de Paris, Paris, France

###### **Correspondence:** Guillaume Geslain (guillaume.geslain@aphp.fr)

*Ann. Intensive Care* 2020, **10 (Suppl 1):**CO-12

**Rationale:** Unplanned extubation (UE) is a potentially serious complication that can contribute to increase patient morbidity, mortality and length of stay in pediatric intensive care unit (PICU). The most frequent risk factors reported in literature are younger age, patient agitation, inadequate tube fixation, copious secretions, procedures performed during medical or paramedical care and nursing workload. The aim of our study is to determine incidence and contributing factors of UE in our PICU.

**Patients and methods:** We conducted a prospective monocentric study from January 1, 2010, to December 31, 2018 in a tertiary PICU. All mechanically ventilated children less than 18 years-old admitted to our PICU were included except for patients with tracheotomy. We monitored all cases of UE occurred during PICU stay. Demographic and clinical data were collected using an event report form including patient characteristics, a description of the extubation circumstances and the outcomes.

**Results:** We reported 102 UE in 9 years for 14,661 days of mechanical ventilation. The overall UE incidence was 6.96 per 1000 ventilation days. Characteristics of patients and UE circumstances are reported in Table 1. Some data on events after UE are missing. Thirteen patients on 82 (15.9%) needed non-invasive ventilation after UE, 4 of them needed reintubation (30.8%). Sixty patients on 97 (61.9%) needed reintubation, and 47 of them (78.3%) required immediate reintubation. Over the study period, the number of UE per year was stable. Presence of physical restraints, nasally inserted endotracheal tubes and cuffed tubes seemed not to prevent patients from self-extubating.

**Discussion:** In North America, the nurse-to-patient ratio in PICU is 1:1, in our unit this ratio is at 1:2.29. A reorganization of the PICU staff for intubated patients should be redesigned in our unit. It would also be interesting to study the kinetic of sedation decreasing and to collect the presence or absence of a withdrawal syndrome for its potential implication in UE.

**Conclusion:** Areas for potential improvement seem to lie in implementation of monitoring and staff education programs. Iterative intubations can cause laryngeal and tracheal trauma and prolong length of mechanical ventilation and PICU stay. Our aim is to implement strategies to reduce the occurrence of UE.

**Compliance with ethics regulations:** Yes.

**Table 1:** Characteristics of patients and circumstances of unplanned extubation

Total number of accidental extubations n = 102	
Age < 2 years	68/102 (67.1%)
Use of sedation	80/100 (80.0%)
Patients described as agitated	35/96 (36.5%)
Presence of physical restraints	90/98 (91.8%)
Nasally inserted endotracheal tube	47/101 (46.5%)
Cuffed endotracheal tube	53/92 (57.6%)
Unknown circumstances (no staff at bedside)	55/102 (53.9%)
Re-intubation rate	59/97 (60.8%)
Median nurse to patient ratio	1:2.29

### CO-13 Score for the risk of acquisition of pressure ulcer in the ICU: Data from the Pressure Study

#### Philippe Michel^1^, Gwenaëlle Jacq^1^, Gregoire Muller^1^, Guillaume Decormeille^1^, Atika Youssoufa^1^, Laurent Poiroux^1^, Brigitte Barrois^2^, Nadia Aissaoui^1^, Saber Davide Barbar^1^, Florence Boissier^1^, David Grimaldi^1^, Sami Hraiech^1^, Nicholas Heming^1^, Bertrand Hermann^1^, Jean-FrançOis Llitjos^1^, Lamia Besbes^1^, Jean-Baptiste Lascarrou^1^, Gaël Piton^1^

##### ^1^CERC, Paris, France; ^2^Association PERSE, Paris, France

###### **Correspondence:** Philippe Michel (philippe@docteur-michel.fr)

*Ann. Intensive Care* 2020, **10 (Suppl 1):**CO-13

**Rationale:** Critically ill patients are at risk of developing pressure ulcers (PUs) during their ICU stay. Existing scores of PU have not been validated in the context of ICU. We aimed to create a score evaluating the risk of acquisition of PU being specific of the ICU.

**Patients and methods:** Data from a one-day point prevalence study performed in June 2017 in 1228 patients in 86 ICUs in France (The Pressure Study). On the same day, the presence or absence of PUs in all hospitalized patients of participating ICUs, data on the ICUs, and the characteristics of patients and of PUs had been evaluated. Factors having been significantly associated with acquisition of PU in the ICU by univariate analysis were selected. Quantitative variables were dichotomized at a threshold identified by the ROC curve analysis. The variables were included in a multiple logistic regression analysis for the acquisition of PU in the ICU. The independent variables being the most clinically relevant were included in a score, with a weighting of 1 point for each variable.

**Results:** After exclusion of the IGS2 score, 6 variables were independently associated with acquisition of PU in the ICU: having been confined to bed before ICU admission, presence of motor neurological disorder, body weight ≥ 90 kg, use of high-dose steroids, length of ICU stay > 10 days and need for artificial nutrition (AUC = 0.78). The AUC of a model limited to the four first described variables was correct (AUC = 0.73). A “BCD Weight” score (Bed, Corticosteroids, motor Deficit, body WEIGHT) ranging from 0 to 4 was created. The prevalence of acquired PU was 4.6%, 12.9%, 26.0%, 48.8%, and 100%, among patients presenting with a score 0, 1, 2, 3, and 4, respectively (Fig. 1). Comparatively, the prevalence of acquired PU was 0%, 3.7%, 9.3% and 26.9% among patients classified as no risk, low risk, moderate risk, and high risk with the usual scale evaluating the risk of PU.

**Conclusion:** We identified an easy to remember score addressing the risk of PU in the context of ICU. This “BCD Weight” score, calculable from ICU admission and throughout the stay, might help clinicians to evaluate the risk of acquisition of PU in their patients.

**Compliance with ethics regulations:** Yes.

**Fig. 1:**
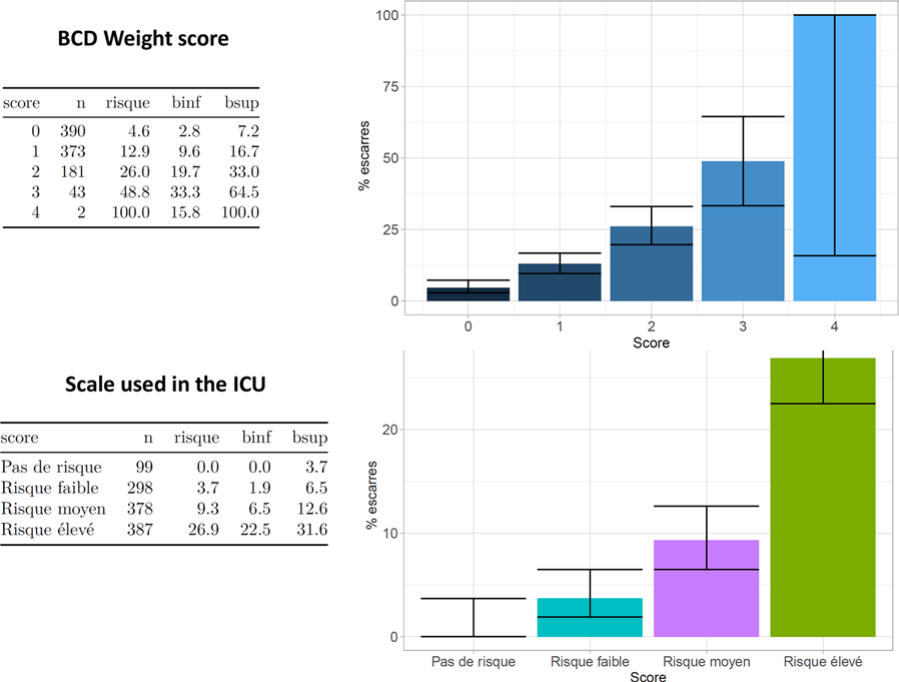
Comparison of a score specific of the ICU with usual scale for the acquisition of PU

### CO-14 Effect of short incitement to follow social media to improve knowledge of scientific literature by intensive care trainees

#### Jean-Baptiste Lascarrou^1^, Stephan Ehrmann^2^, Maelle Martin^1^, Pierre Potier^1^, Jean-Marie Castillo^3^, Jean Reignier^1^, Emmanuel Canet^1^

##### ^1^Médecine Intensive Réanimation, Nantes, France; ^2^Médecine Intensive Réanimation, Tours, France; ^3^General Practionner, Nantes, France

###### **Correspondence:** Jean-Baptiste Lascarrou (jeanbaptiste.lascarrou@chu-nantes.fr)

*Ann. Intensive Care* 2020, **10 (Suppl 1):**CO-14

**Rationale:** Improving knowledge of recent medical literature by intensive care trainees is a major target for medical education and the first step to improve patient’s care. We test the hypothesis that following major Twitter feeds dedicated to intensive care will improve knowledge of recent studies published in medical journals by intensive care trainees.

**Patients and methods:** We included French intensive care trainees (ICT) between March 2019 and October 2019 in an educational randomized trial. All ICT were informed and they consent to the trial. After initial online interrogation, ICT were separated between those already on Twitter© or not (Group 1: ICT already on Twitter©). All ICT not already on Twitter were randomized on 2 groups: Group 2 (short information and incitation to open a Twitter account and to follow critical care journal feeds) or group 3 (control group). ICT were interrogated on their recent medical literature knowledge at 3 and 6 month on 5 trials published in pre-selected journals.

**Results:** During the study period, on the 969 French ICT contacted, 77 agree to participate: 16 were already on Twitter, 31 were randomized to Twitter incitation and 30 to control group. At 3 month, there were 62 who answered electronic questionnaire. Self-declaration of article knowledge was not different between 3 groups (P = 0.85). Knowledge of primary outcome of each trial was not significantly better in 3 groups (P = 0.09). In per-protocol analysis of ICT on Twitter or not, knowledge of article and primary outcome were also not significantly different (respectively P = 0.57 and P = 0.17).

**Conclusion:** Short incitation to open a Twitter account and follow major medical journals with specific focus on cardiac arrest did not improve knowledge of medical literature by intensive care trainees at 3 month. Further trials are needed to better imply intensive care trainees in scientific medical literature.

**Compliance with ethics regulations:** Yes.

**Table 1:** Results at 3 month

	Group 1 Already on Twitter (n = 16)	Group 2 Randomized Twitter (n = 31)	Group 3 Randomized Control (n = 30)	P
Number or respondents	10	24	28	0.29
Opening of a Twitter account	–	21	1	–
Knowledge of #FOAMed resource Sci-Hub Unpaywall Kopernio PubMed Central	10 0 2 5	22 0 0 13	28 0 0 14	0.29 - 0.02 0.94
Self-declaration of article knowledge	4 [2–5] 3.6 ± 1.4	4[3–5] 3.5 ± 1.3	4 [2.5–4] 3.4 ± 1.3	0.85
Global score of article knowledge (1 point for each good answer to primary outcome)	4 [3–5] 3.9 ± 0.9	4 [2.5–4] 3.5 ± 1.2	3 [2–4] 3.1 ± 0.9	0.09
At least one abstract read	9	21	23	0.89
At least one article read	8	10	15	0.11
At least one article discussed	9	20	20	0.45
At least one article discussed on Twitter	0	0	0	–

### CO-15 Construction of a stress scale specific to intensive care units: the PS-ICU scale

#### Alicia Fournier^1^, Florent Lheureux^2^, Maria Cruz Martin Delgado^3^, Maria Grazia Bocci^4^, Alessia Prestifilippo^5^, Amélie Anota^6^, Pierre Aslanian^7^, Guillaume Besch^8^, Jean-Michel Constantin^9^, Jean-Pierre Quenot^10^, Belaid Bouhemad^11^, Gilles Capellier^12^, Alexandra Laurent^1^

##### ^1^Department of psychology, University of Burgundy, Dijon, France; ^2^Department of psychology, University of Franche-Comté, Besançon, France; ^3^Hospital Universitario Torrejón en Torrejón de Ardoz, Madrid, Spain; ^4^Department of anesthesiology and intensive care medicine, Fondazione Policlinico Universitario Agostino Gemelli—IRCCS, Roma, Italy; ^5^Psychologist, Private practice, Roma, Italy; ^6^Methodology and quality of life unit in oncology, Centre hospitalier régional universitaire de Besançon, Besançon, France; ^7^Department of intensive care medicine, CHUM, Montréal, Canada; ^8^Surgical intensive care unit, Centre hospitalier régional universitaire de Besançon, Besançon, France; ^9^Anesthesiology critical care and perioperatrive medicine, Hôpital La Pitié Salpêtrière, Paris, France; ^10^Department of intensive care medicine, Centre hospitalier régional universitaire de Dijon, Dijon, France; ^11^Surgical intensive care unit, Centre hospitalier régional universitaire de Dijon, Dijon, France; ^12^Department of intensive care medicine–samu 25, Centre hospitalier régional universitaire de Besançon, Besançon, France

###### **Correspondence:** Alicia Fournier (alicia.fournier@u-bourgogne.fr)

*Ann. Intensive Care* 2020, **10 (Suppl 1):**CO-15

**Rationale:** The intensive care units (ICU) are fertile ground for the emergence of professional stressors. Assessed by individuals as situations that weaken or are beyond their resources, work-related stressors impact the mental and physical health of workers and the quality and safety of care. Currently, many tools are used to assess caregiver stress in ICU, but do not consider the specificity of this work. The objective of this international and multicentric study was to develop a perceived stress scale specific to ICU.

**Patients and methods:** Interviews were conducted with 166 caregivers (84 nurses and 81 physicians) in four countries (France, Italy, Spain, Canada). These interviews were recorded, transcribed and then a thematic analysis was carried out to identify stress factors. A first version of the scale was pre-tested with 70 caregivers (30 physicians and 40 nurses) in the same countries. Finally, we carried out qualitative and quantitative analyses select the most relevant items.

**Results:** We identified 99 stressors specific to the ICU that were grouped into eight main themes: stress in relation to 1) the patient, 2) the task to be performed, 3) the institutional context, 4) the team, 5) the organization of the unit, 6) the personal dimensions, 7) the patient’s family, 8) the working conditions. Following the pre-test, 50 items were selected to constitute the PS-ICU scale.

**Conclusion:** Our results highlight specific items related to vital risk/emergency management and ethically and morally problematic situations. These dimensions will be discussed and compared against existing scales (e.g., JCQ). The PS-ICU scale will allow to better identify and measure stressors in ICU. This scale will contribute to the development of targeted actions in terms of prevention, training and support for professionals. The creation of an internationally validated tool will make it possible to develop comparative studies on cultural and organizational factors.

**Compliance with ethics regulations:** Yes.

### CO-16 Are Intensive Care Residents more exposed to Anxiety/Depression?

#### Mehdi Marzouk^1^, Manel Lahmar^2^, Zeineb Hammouda^2^, Islem Ouanes^2^, Fahmi Dachraoui^2^, Lamia Besbes^2^, Fekri Abroug^2^

##### ^1^Service de Réanimation Centre Hospitalier de Béthune, Beuvry, France; ^2^Service de Réanimation Polyvalente. CHU F.Bourguiba, Monastir, Tunisia

###### **Correspondence:** Mehdi Marzouk (docmehdi.marzouk@gmail.com)

*Ann. Intensive Care* 2020, **10 (Suppl 1):**CO-16

**Rationale:** When compared to general population, moods disorders are more prevalent among health care workers and especially young doctors. Whether certain specialties are more exposed than others given the burden of workload and specific aspects is not known. The aim of this study is to assess the prevalence of anxiety and depressive symptoms among Tunisian young residents and verify whether they are more frequent in specialties with high workload such as Intensive Care Medicine.

**Patients and methods:** We conducted a cross-sectional survey in all Tunisian medical residents brought together between 14 and 22 December 2015 to choose their next 6-month rotation. The items of the Hospital Anxiety and Depression (HAD) questionnaire were employed to capture the prevalence of anxiety and/or depression among the residents. The statistical relationships between anxiety and depression (HAD score) and work-related data were explored by Poisson regression. In particular we compared a group of specialties including Intensive care, Anesthesiology, and Emergency medicine (Acute care group), to the rest of specialties.

**Results:** 1700 out of 2200 (77%) medical residents answered the questionnaire. Among these, residents who started the first semester of a new curriculum (n = 320) were not included. 243 (17.6%) were in the acute care group.

Overall, 73.5% of the participating residents had either definite (44.2%) or probable (29.3%) anxiety, while 65% had definite (33.5%) or probable (31.5%) depression symptoms. In the acute care group, these proportions were not substantially higher: 48.6% and 27.6% for definite and probable anxiety, respectively; and 37% and 31.7% for definite and probable depression, respectively. Total HAD score was significantly associated with the resident’s age (OR = 1.01, 95% CI 1.004 to 1.02, p = 0.001); female gender; and the heavy burden of work imposed on a weekly or monthly basis, as reflected by the number of hours worked per week (0.3% increase per worked hour per week), and the number of night shifts per month (1.5% increase per night shift).

**Conclusion:** Anxiety/Depression symptoms are not more frequent in Intensive Care, Anesthesiology, or Emergency medicine residents. Rather, these symptoms are related to the socio-demographic situation of residents, and the workload characteristics in general.

**Compliance with ethics regulations:** NA.

### CO-17 Outcome of adult sickle cell patients admitted in ICU: national retrospective study in French ICUs

#### Maïté Agbakou^1^, Noelle Brule^2^, Morgane Pere^3^, Emmanuel Canet^2^, Jean Reignier^2^, Jean-Baptiste Lascarrou^2^

##### ^1^Service de médecine intensive réanimation, CHU Nantes, Nantes, France; ^2^CHU Nantes, Service De Médecine Intensive Réanimation, Chu Nantes, France; ^3^Plateforme de Méthodologie et Bistastistiques, direction de la recherche, CHU Nantes, Nantes, France

###### **Correspondence:** Maïté Agbakou (maite.agbakou@gmail.com)

*Ann. Intensive Care* 2020, **10 (Suppl 1):**CO-17

**Rationale:** Sickle cell disease (SCD) is associated with high morbidity and mortality, and most of deaths in sickle cell patients occur in the ICU. The purpose of this styudy was to describe clinical characteristics of adult sickle cell patients requiring ICU admission, and identify prognostic factors associated with an adverse outcome defined by death in the ICU or need for vital support.

**Patients and methods:** This multicentric observational retrospective study included all adult patients with SCD, admitted in 16 French university hospital ICUs, from January 1st 2015 to December 31th 2017. Only the first episode on the study period was analyzed for each patient. Main outcome was the occurrence of an adverse outcome, defined by death in ICU or need for vital support. Predictors of adverse outcome were assessed by Cox regression model.

**Results:** Four hundred and eighty-eight patients were included during the study period. Reasons for ICU admission were mainly SCD related, with acute chest syndrome being the first one (47.5%). Adverse outcome occurred in 81 (16.6%) patients, with 9.4% patients requiring invasive mechanical ventilation, 5.9% non-invasive mechanical ventilation, 6.6% vasopressor support, 3.7% renal replacement therapy and 1.6% ECMO (arterio-veinous or veno-venous). Those patients had more often high blood pressure, chronic kidney failure and pulmonary hypertension than those without adverse outcome. Sixteen (3.3%) patients died in the ICU, mainly of multi-organ failure following sickle cell crisis or sepsis. In multivariable analysis, independent predictors of adverse outcome were mean arterial pressure (OR 0.98 IC 95% (0.96–1), p = 0.034), respiratory rate (OR 1.09 IC 95% (1.01–1.47), p = 0.035), hemoglobin level (OR 1.22 IC 95% (1.01–1.47)) and creatinine clearance (OR 0.98 IC 95% (0.97–0.98) p < 0.0001) in ICU, and blood exchange transfusion before ICU admission (OR 5.75 IC 95% (1.32–25.03), p = 0.02).

**Conclusion:** This multicentric study confirmed well-known predictors of adverse outcome, and identified for the first-time low mean arterial blood pressure in ICU, and blood exchange transfusion before ICU admission as independent predictors of adverse outcome in SCD. This last finding interrogates the need for systematic referral to ICU of SCD patients requiring blood exchange transfusion.

**Compliance with ethics regulations:** Yes.

### CO-18 In-Hospital Mortality-Associated Factors of Thrombotic Antiphospholipid Syndrome Patients Requiring Intensive Care Unit Admission

#### Marc Pineton De Chambrun^1^, Romaric Larcher^2^, Frédéric Pene^3^, Laurent Argaud^4^, Julien Mayaux^5^, Rémi Coudroy^6^, Elie Azoulay^7^, Yacine Tandjaoui-Lambiotte^8^, Stanislas Faguer^9^, Charles-Edouard Luyt^1^, Alain Combes^1^, Zahir Amoura^10^

##### ^1^Sorbonne Université, APHP, Hôpital La Pitié–Salpêtrière, Institut de Cardiométabolisme et Nutrition (ICAN), Service de Médecine Intensive-Réanimation, Paris, France; ^2^Service de Médecine Intensive-Réanimation, Hôpital Lapeyronie, Centre Hospitalier Universitaire (CHU) de Montpellier PhyMedExp, Université de Montpellier, INSERM, CNRS, Montpellier, France; ^3^Service de Médecine Intensive-Réanimation, Hôpital Cochin, Hôpitaux Universitaires Paris Centre, APHP & Université Paris Descartes, Paris, France; ^4^Service de Médecine Intensive-Réanimation, Hôpital Edouard-Herriot, Hospices Civils de Lyon, Lyon, France; ^5^APHP, Hôpital La Pitié–Salpêtrière, Service de Pneumologie, Médecine Intensive et Réanimation Médicale, Département R3S, Paris, France; ^6^Service de Médecine Intensive-Réanimation, INSERM CIC1402, Groupe ALIVE, Université de Poitiers, CHU de Poitiers, Poitiers, France; ^7^Service de Médecine Intensive-Réanimation, Hôpital Saint-Louis, APHP, Paris, France; ^8^Service de Réanimation Médico-Chirurgicale, Hôpital Avicenne, APHP, HUPSSD, Bobigny, France; ^9^Département de Néphrologie et Transplantation d’Organes, Unité de Réanimation, Centre de Référence des Maladies Rénales Rares, Hôpital Rangueil, CHU de Toulouse, Toulouse, France; ^10^Sorbonne Université, Assistance Publique-Hôpitaux de Paris (APHP), Hôpital La Pitié–Salpêtrière, Institut E3M, Service de Médecine Interne 2, Centre de Référence National Lupus Systémique, Syndrome des Anticorps Anti-phospholipides et Autres Maladies Auto-Immunes Systémiques Rares, Paris, France

###### **Correspondence:** Marc Pineton de Chambrun (marc.dechambrun@gmail.com)

*Ann. Intensive Care* 2020, **10 (Suppl 1):**CO-18

**Rationale:** The antiphospholipid syndrome (APS) is a systemic autoimmune disease defined by thrombotic events that can require ICU admission because of organ dysfunction related to macrovascular and/or microvascular thrombosis. Critically ill thrombotic APS patients were studied to gain insight into their prognoses and in-hospital mortality-associated factors.

**Patients and methods:** This French national, multicenter, retrospective study included all APS patients with any new thrombotic manifestation (s) admitted to 24 ICUs (January 2000-September 2018).

**Results:** During the study period, 134 patients (male/female ratio, 0.4) with 152 APS episodes were admitted to the ICU (at mean age 46.0 ± 15.1 years). In-hospital mortality of their 134 last episodes was 35/134 (26.1%). The Cox multivariable model retained (HR [95% CI]): age ≥ 40 years (11.4 [3.1–41.5]; P < .0001), mechanical ventilation (11.0 [3.3–37]; P < .0001), renal replacement therapy (2.9 [1.3–6.3]; P = .007) and in-ICU anticoagulation (0.1 [0.03–0.3]; P < .0001) as independently associated with in-hospital mortality. For the subgroup of “definite/probable CAPS”, the Cox bivariable model including the SAPS II score retained double therapy (corticosteroids + anticoagulant: 0.2 [0.07–0.6]; P = .005) but not triple therapy (corticosteroids + anticoagulant + intravenous immunoglobulins or plasmapheresis: HR 0.3 [0.1–1.1]; P = .07) as independently associated with in-hospital mortality (Fig. 1).

**Discussion:** Triple therapy is the recommended first-line treatment of CAPS. However, herein, it was not significantly associated with better survival in critically ill, thrombotic APS patients. For the subgroup of “definite/probable CAPS” patients, double and triple regimens were associated with survival. But the bivariable analyses including the day-0 SAPS II showed that survival was linked to in-ICU anticoagulation and corticosteroids—not IVIg or plasmapheresis. Our findings indicate that corticosteroids should probably be added to in-ICU anticoagulation to treat “definite/probable CAPS”. Frequent fever and elevated C-reactive protein in all thrombotic APS patients suggest a marked inflammatory state that could explain corticosteroid efficacy. Neither plasmapheresis nor IVIg impacted the prognosis of “definite/probable CAPS”, but that finding could be explained by a lack of power compared to CAPS Registry data.

**Conclusion:** In-ICU anticoagulation was the only APS-specific treatment independently associated with survival for all patients. Double—but not triple—therapy was independently associated with better survival of “definite/probable CAPS” patients. In these patients, double therapy should be used as first-line therapy while the role of triple therapy requires further evaluation.

**Compliance with ethics regulations:** Yes.

**Fig. 1:**
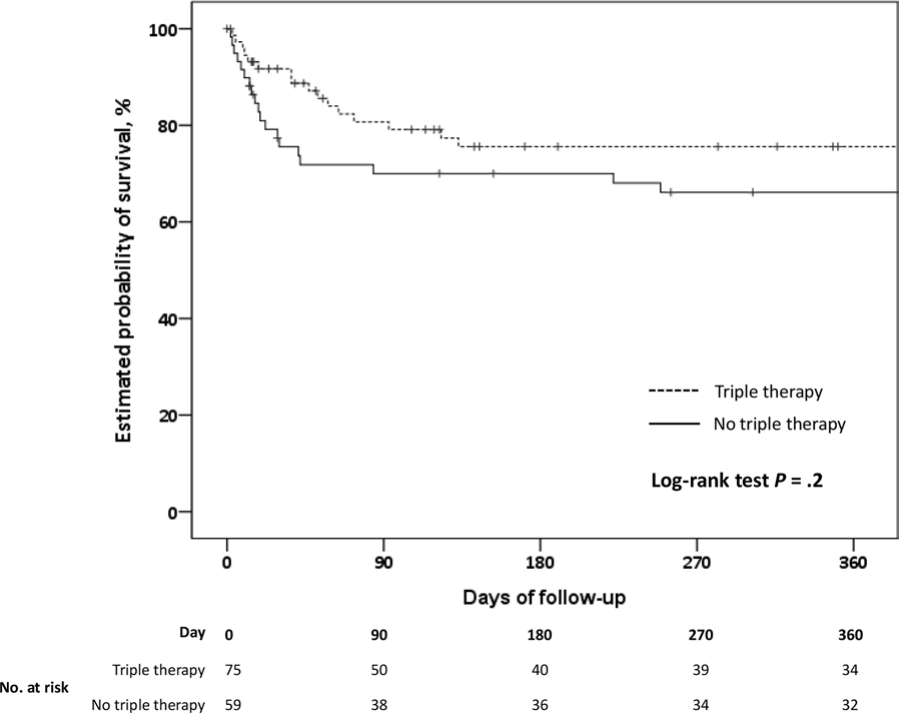
Kaplan-Meier estimated probability of survival for the 134 APS patients’ last episodes requiring intensive care unit admission with comparison according triple therapy use

### CO-19 Coagulation disorders in critically ill HLH patients: a prospective study

#### Sandrine Valade^1^, Michaël Darmon^1^, Amélie Launois^2^, Bérangère Joly^2^, Jehane Fadlallah^3^, Lionel Galicier^3^, Claire Fieschi^3^, Lara Zafrani^1^, Virginie Lemiale^1^, Anne Claire Lepretre^4^, Adrien Mirouse^1^, Jean-Jacques Tudesq^1^, Agnès Veyradier^2^, Elie Azoulay^1^, Eric Mariotte^1^

##### ^1^Saint-Louis Hospital, Medical ICU, Paris, France; ^2^Hematology Department and Research Unit EA3518, Institute of Hematology, French Reference Center for Thrombotic Microangiopathies, Lariboisière Hospital, University Paris Diderot, Paris, France; ^3^Department of Clinical Immunology, Saint-Louis Hospital, Paris, France; ^4^Saint-Louis Hospital, Transfusion Department, Etablissement Français Du Sang, Paris, France

###### **Correspondence:** Sandrine Valade (sandrine.valade@aphp.fr)

*Ann. Intensive Care* 2020, **10 (Suppl 1):**CO-19

**Rationale:** Hemophagocytic lymphohistiocytosis (HLH) is a rare condition that can be severe and lead patients to the ICU. Coagulation disorders are commonly observed during HLH, the most frequently reported being a decreased fibrinogen level. Hemostasis impairment has been associated with increased risk of bleeding and death in previous studies. The main objective of this study was to describe coagulation defects during HLH in order to identify early markers associated with bleeding events.

**Patients and methods:** In this prospective study conducted in the ICU and the hematological wards of one university hospital between April 2015 and December 2018, all the patients with a new diagnosis of HLH were included. Blood samples were retrieved at day 1 and day 7 to explore hemostasis. Coagulation disorders were defined as PT < 50% and/or fibrinogen < 2 g/L. Results are presented as median [interquartile range] and number (percent).

**Results:** During the study period, 47 patients aged 54 years [42–67] were included. Seventy-nine percent required ICU admission, mainly for acute respiratory (n = 14; 30%) or hemodynamic failure (n = 10; 21%). Patients fulfilled 5 [4–5] HLH 2004 criteria and their HScore was 244 [221–276]. Fever was almost constant and histological hemophagocytosis was found in 68% of the patients. HLH etiology was hematological malignancy in 35 patients (74%), infectious disease in 7 patients (15%) and auto-immune disease in 2 patients (4%). Three additional patients had an alternative diagnosis or unknown etiology. Thirty patients (64%) presented coagulation disorders and 11 (23%) experienced a bleeding event. At day 1, fibrinogen level was 2.65 g/L [1.61–5.66], ADAMTS13 activity 22% [12–33], PT 64% [48–72], fibrin degradation products 8.69 [5–31] (N < 6 µg/L), PAI-1 94.1 [45–188] (N = 4–43 ng/mL), tPA 45.2 [30.7–66.6] (N = 2–12 ng/mL). Fifteen patients (32%) required mechanical ventilation and 17 (36%) vasopressors. Etoposide was administered to 72% of the patients. Eighteen (38%) patients died during hospital stay. In multivariate analysis, the occurrence of a severe hemorrhage (OR 3.215 [1.194–8.653], p = 0.021) and SOFA score (OR 1.305 per point [1.146–1.485], p < 0.001) were associated with a higher mortality rate. No early biological marker was associated with bleeding.

**Conclusion:** Coagulation disorders are frequent during HLH. Severe bleedings occur in almost one in four patients and confer an increased risk of death. This is the first prospective study specifically targeting hemostasis disorders in HLH. Investigations on specialized hemostasis function are ongoing, in order to determine the mechanisms leading to coagulopathy.

**Compliance with ethics regulations:** Yes.

**Fig. 1:**
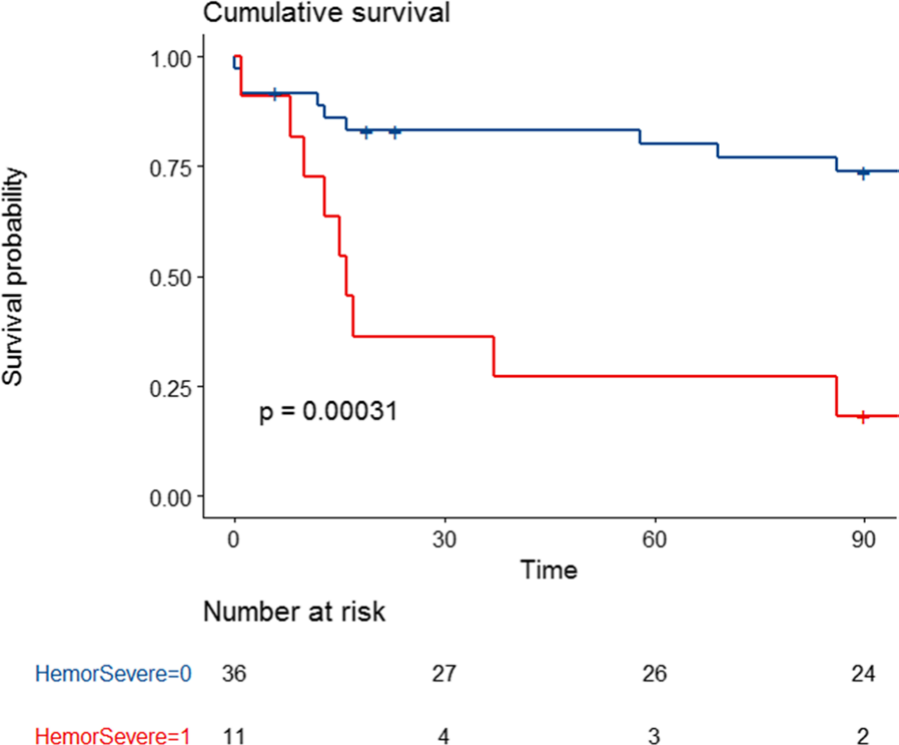
Survival curve according to severe bleeding

### CO-20 Thrombotic thrombocytopenic purpura related neurological manifestations: clusters at presentation and long-term prognosis

#### Adrien Mirouse^1^, Stéphane Legriel^2^, Sylvie Chevret^1^, Agnès Veyradier^1^, Lionel Galicier^1^, Lara Zafrani^1^, Eric Mariotte^1^, Elie Azoulay^1^

##### ^1^Hôpital Saint-Louis, Paris, France; ^2^Centre Hospitalier de Versailles, Le Chesnay, France

###### **Correspondence:** Adrien Mirouse (adrien.mirouse@aphp.fr)

*Ann. Intensive Care* 2020, **10 (Suppl 1):**CO-20

**Rationale:** Thrombotic thrombocytopenic purpura (TTP) is a thrombotic microangiopathy with frequent neurological manifestations. Data are lacking concerning precise description of neurological manifestations, timing of symptoms, correlation between clinical manifestations and neuro-imaging, and recovery after a severe episode of neurological TTP. This study aimed at describing neurological manifestations during TTP episodes and long term neurological outcome.

**Patients and methods:** Prospective study in one French ICU including all adult patients with an acute TTP diagnosis with neurological manifestations. Patients were included if they had thrombocytopenia and microangiopathic hemolytic anemia with signs of visceral ischemia and an ADAMTS13 activity < 10%. Neurological clusters were identified using a non parametric unsupervised cluster analysis. Long term neurological recovery was assessed with Glasgow Outcome Scale (GOS).

**Results:** 108 patients were included from 1997 to 2019. Neurological symptoms were migraine-like symptoms (64%), limb weakness/paresthesia (49%), pyramidal syndrome (39%), confusion or cognitive impairment (34%), obtundation (31%), seizure (19%), visual symptoms (20%), and cerebellar syndrome (18%). Time between neurological symptoms and ICU admission was 7 [3–18] days. A cerebral CT-scan performed in 48 (44%) patients and an MRI in 67 (62%) were abnormal in 9 (19%) and 27 (40%) cases, respectively. Twenty-seven (25%) patients had an electroencephalogram, abnormal in 12 (44%) cases. Three clusters of patients were identified. Cluster 1 included younger patients (37 [27–48] vs. 41 [32–52] and 48 [35–54], p = 0.045), with headaches (75% vs. 27% and 36%, p < 0.0001). Cluster 2 patients presented ataxic gait and cerebellar syndrome (77% vs. 0% and 0%, p < 0.0001), and dizziness (50% vs. 0% and 0%, p < 0.0001). Cluster 3 patients presented confusion (36% vs. 0% and 9%, p < 0.0001), obtundation (58% vs. 0% and 24%, p < 0.0001), and seizure (36% vs. 0% and 14%, p < 0.0001). All patients were treated with plasma exchange therapy. Median ICU length-of-stay was 8 [6–16.5] days. During ICU management, 31 (29%) patients required mechanical ventilation, 18 (17%) vasopressor use, and 16 (15%) renal replacement therapy. Six (6%) patients died in ICU. After a median follow-up of 34 [12–71] months, 100 (93%) patients were alive. Patients from cluster 1 were more frequently GOS 5 compared to cluster 2 and 3 at 3 months (44 [98%] vs. 13 [65%] and 21 [60%], p < 0.0001), 6 months (44 [100%], 15 [68%], and 23 [69%], p < 0.0001), and 1 year (40 [100%] vs. 15 [79%] and 20 [57%], p < 0.0001).

**Conclusion:** Neurological recovery may be delayed after a TTP episode. One year full neurological recovery range from 57% to 100% depending on neurological initial presentation.

**Compliance with ethics regulations:** Yes.

**Table 1:** Patients outcome according to cluster

Outcome	Cluster 1 n = 47	Cluster 2 n = 22	Cluster 3 n = 39	p-value
Month 3 (n = 108)				
Relapse	4 (9%)	0 (0%)	1 (3%)	0.43
Alive	47 (100%)	22 (100%)	37 (95%)	0.0001
GOS 5	44 (98%)	13 (65%)	21 (60%)	< 0.0001
Motor deficiency	0 (0%)	4 (20%)	8 (24%)	0.0006
Cognitive impairment	0 (0%)	6 (30%)	9 (27%)	< 0.0001
Month 6 (n = 105)				
Relapse	2 (5%)	1 (5%)	2 (6%)	1.00
Alive	44 (100%)	22 (100%)	36 (92%)	< 0.0001
GOS 5	44 (100%)	15 (68%)	23 (69%)	< 0.0001
Motor deficiency	0 (0%)	5 (26%)	6 (19%)	0.0008
Cognitive impairment	0 (0%)	4 (21%)	7 (23%)	0.001
Year 1 (n = 94)				
Relapse	4 (10%)	1 (5%)	1 (4%)	0.86
Alive	40 (100%)	19 (100%)	27 (77%)	0.0002
GOS 5	40 (100%)	15 (79%)	20 (57%)	< 0.0001
Motor deficiency	0 (0%)	2 (11%)	4 (15%)	0.021
Cognitive impairment	0 (0%)	2 (11%)	7 (26%)	0.001

### CO-21 Relationship between diaphragm thickening fraction and transdiaphragmatic pressure in healthy and mechanically ventilated patients: a breath by breath analysis

#### Thomas Poulard^1^, Quentin Fossé^2^, Jean-Luc Gennisson^3^, Marie-Cécile Niérat^2^, Jean-Yves Hogrel^1^, Thomas Similowski^2^, Alexandre Demoule^2^, Damien Bachasson^1^, Martin Dres^2^

##### ^1^Institute of Myology, Neuromuscular Investigation Center, Neuromuscular Physiology Laboratory, Paris, France; ^2^AP-HP, Groupe Hospitalier Pitié-Salpêtrière Charles Foix, Service de Pneumologie, Médecine Intensive et Réanimation, (Département “R3S”), Paris, France; ^3^Imagerie par Résonance Magnétique Médicale et Multi-Modalités (IR4M), CNRS UMR8081, Université Paris-Saclay, Orsay, France

###### **Correspondence:** Thomas Poulard (t.poulard@institut-myologie.org)

*Ann. Intensive Care* 2020, **10 (Suppl 1):**CO-21

**Rationale:** Diaphragm thickening fraction (TFdi) measured by ultrasound (US) is widely used in clinical research to evaluate diaphragm function in order to guide clinicians in providing optimal ventilator support. Studies reported TFdi cut-off values that could help in predicting weaning outcome in mechanically ventilated (MV) patients, but surprisingly, very little is known on the relationship between TFdi and the changes in transdiaphragmatic pressure (ΔPdi), the reference method. The present study investigated the relationship between ΔPdi and TFdi in healthy subjects and in MV patients.

**Patients and methods:** Pdi was monitored with gastric and esophageal catheters and US was performed at the zone of apposition of the right hemi-diaphragm. Healthy subjects breathed against an external inspiratory threshold load of 0–50% of maximal inspiratory pressure. MV patients were tested under several ventilator assistances before performing a spontaneous breathing trial. A breath by breath analysis was performed to confront ΔPdi and TFdi for a given breathing cycle. Pearson correlation coefficients (r) were used to determine within-individual and overall relationships between ΔPdi and TFdi.

**Results:** Fifteen healthy volunteers and 22 MV patients were studied. One healthy subject displayed a significant positive correlation between ΔPdi and TFdi (r = 0.32, p < 0.01). Only three of the 22 MV patients presented with a significant positive correlation between ΔPdi and TFdi (mean r = 0.67, 95% CIs [0.41, 0.83] in patients with significant ΔPdi-TFdi correlation, all p < 0.01). Overall relationship between ΔPdi and TFdi in MV patients was weak (R = 0.17, 95% CIs [0.04, 0.29], p < 0.05). Individual relationships between ΔPdi and TFdi and averaged values for every condition tested are presented in Fig. 1.

**Conclusion:** These findings show that TFdi does not generally correlate with Pdi suggesting that TFdi may not be used as a surrogate for Pdi. The explanations for this lack of correlation deserve further studies.

**Compliance with ethics regulations:** Yes.

**Fig. 1:**
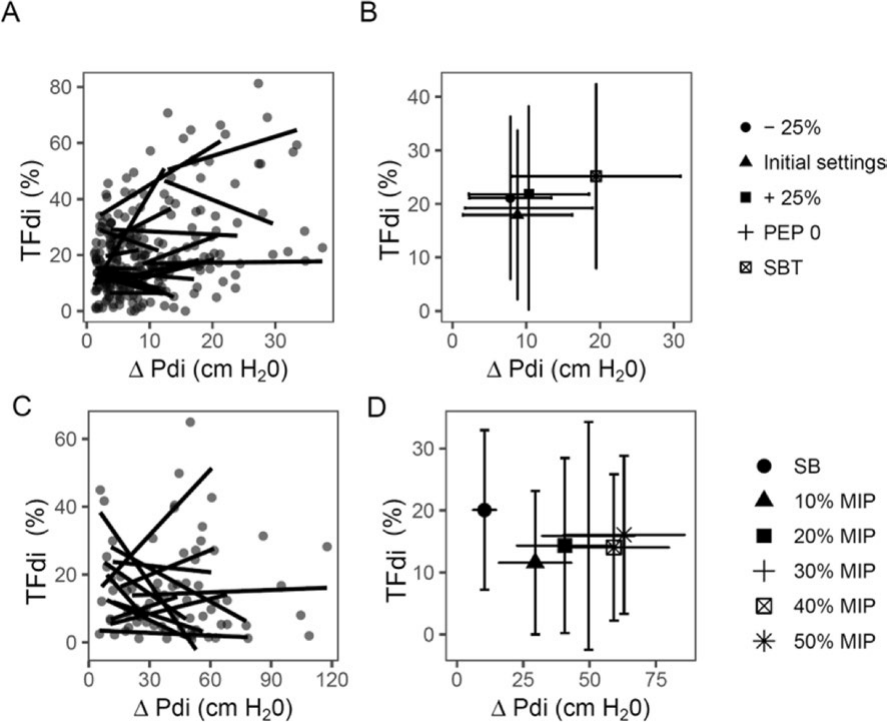
Intra-individual relationships between ΔPdi and TFdi for mechanically ventilated (MV) patients (A) and healthy subjects (C). Relationships between ΔPdi and TFdi when breathing cycles were averaged for all participants during each condition for MV patients (B) and healthy subjects (D). − 25%: initial settings minus 25% inspiratory help, + 25%: initial settings plus 25% more inspiratory help, PEP 0: zero positive end-expiratory pressure, SBT: spontaneous breathing trial. Healthy subjects performed spontaneous breathing (SB) and ventilation against inspiratory threshold at 10, 20, 30, 40 and 50% of maximal inspiratory pressure (MIP)

### CO-22 Accuracy of clinical estimation of the respiratory effort under pressure support ventilation

#### Samuel Tuffet, François Perier, Anne-Fleur Haudebourg, Nicolas De Prost, Keyvan Razazi, Armand Mekontso Dessap, Guillaume Carteaux

##### Réanimation médicale CHU Henri Mondor, Créteil, France

###### **Correspondence:** Samuel Tuffet (samuel.tuffet@hotmail.fr)

*Ann. Intensive Care* 2020, **10 (Suppl 1):**CO-22

**Rationale:** One of the main aims of partial ventilatory support is to maintain the patient’s respiratory effort within a normal range. In clinical routine, the respiratory effort is assessed by clinical judgment, whose accuracy is unknown. In this study, we assessed the accuracy of clinical estimation of the respiratory effort under pressure support ventilation (PSV).

**Patients and methods:** In this prospective monocenter study, patients under mechanical ventilation were included within the first 2 days after switching from assist control ventilation to PSV. Flow, airway pressure, and esophageal pressure were recorded and respiratory effort indices were computed using the FluxMed device (MBMED). Respiratory effort was classified as normal (esophageal pressure time product (PTPes) between 50 and 150 cmH_2_O s min^−1^ (1)), insufficient (PTPes < 50 cmH_2_O s min^−1^) or excessive (PTPes > 150 cmH_2_O s min^−1^). A senior physician, a resident and a nurse were independently asked to clinically evaluate patient’s respiratory effort using a five levels scale: very low, low, normal, high and very high respiratory effort. Data are reported as median [1st–3rd quartile] or number (percentage).

**Results:** Thirty patients, aged 65 years [57–71], with a median SOFA score of 6 [4–9], were included so far. They had spent 9 days [6–12] under mechanical ventilation before inclusion. Median PTPes was 175 cmH2O s min− 1 [98–244]. At the time of assessment, respiratory effort was insufficient in two patients (7%), normal in 10 (33%), and excessive in 18 (60%). Senior physician’s clinical estimation misclassified the respiratory effort in 12 patients (40%). Sensibility/specificity of the senior physician estimation to detect insufficient, normal and excessive respiratory effort were 0.50/0.89, 0.50/0.70 and 0.67/0.75, respectively. Accuracy of the resident and nurse evaluations was consistent with that of senior physician. By univariate analysis, higher SOFA score at inclusion (p = 0.02) and higher age (p = 0.03) were significantly associated with non-detection of excessive respiratory effort.

**Conclusion:** During the 2 days after switching from assist control ventilation to PSV, our preliminary data suggest that: 1/excessive respiratory efforts are frequently observed and 2/the clinical judgment frequently fails to adequately classify the level of respiratory effort, making the excessive efforts underdiagnosed.

**Compliance with ethics regulations:** Yes.

**Reference**
1. The PLeUral pressure working Group (PLUG—Acute Respiratory Failure section of the European Society of Intensive Care Medicine), Mauri T, Yoshida T, Bellani G, Goligher EC, Carteaux G, et al. Esophageal and transpulmonary pressure in the clinical setting: meaning, usefulness and perspectives. Intensive Care Medicine. sept 2016;42 (9):1360–73.

### CO-23 Influence of body mass index on respiratory mechanics in acute respiratory distress syndrome: a multicenter cohort study

#### Rémi Coudroy^1^, Jean-Luc Diehl^2^, Damien Vimpere^3^, Nadia Aissaoui^3^, Romy Younan^3^, Clotilde Bailleul^3^, Amélie Couteau-Chardon^3^, Aymeric Lancelot^3^, Emmanuel Guérot^3^, Chen Lu^4^, Laurent Brochard^4^

##### ^1^Poitiers Hospital, Poitiers, France; ^2^Assitance Publique Hopitaux de Paris, Paris, France; ^3^Georges Pompidou European Hospital, Paris, France; ^4^Interdepartmental Division of Critical Care Medicine, Toronto, Canada

###### **Correspondence:** Rémi Coudroy (r.coudroy@yahoo.fr)

*Ann. Intensive Care* 2020, **10 (Suppl 1):**CO-23

**Rationale:** Overweight and obesity are increasingly prevalent worldwide and account for about 30–40% of patients with acute respiratory distress syndrome (ARDS). How body mass index (BMI) influences respiratory mechanics in ARDS is unclear.

**Patients and methods:** This study is a secondary analysis of 2 cohorts of ARDS according to the Berlin definition: a bicenter Canadian study of 45 ARDS of any BMI enrolled in a prospective study (NCT02457741), and a French monocenter cohort of selected ARDS with a BMI > 40 kg/m2. Airway pressure, flow and esophageal pressure were collected in all patients and we report data at a set positive end-expiratory pressure (PEEP) of 5 cmH2O. Presence of complete airway closure and airway opening pressure were assessed using a low-flow inflation pressure–volume curve. End expiratory lung volume (EELV) was measured using the nitrogen washout/washin technique. The ratio EELV to predicted functional residual capacity was calculated. Patients were sorted in 3 groups according to the World Health Organization overweight classification (BMI < 30, from 30 to < 40, and ≥ 40 kg/m2).

**Results:** Among the 54 patients included, 18 patients (34%) had BMI < 30 kg/m2, 16 (30%) between 30 and 40 kg/m2, and 19 (36%) ≥ 40 kg/m2. The median PaO2/FiO2 was 138 mmHg with a PEEP of 15 cmH2O, and mortality was 32% without difference across groups. Airway closure occurrence increased with BMI (22%, 38% and 58%, p = 0.04). When present, airway opening pressure was 9.6 cmH2O (8.5–13.2) and similar between the 3 groups. With increasing BMI, total PEEP increased from 6.0 to 9.0 cmH2O between groups (p = 0.02). All values of esophageal pressure increased with BMI. End-expiratory esophageal pressure was strongly correlated with BMI (rho = 0.71, p < 0.001), as illustrated in Fig. 1. Consequently end-expiratory transpulmonary pressure decreased from − 2.7 to − 9.3 cm H2O with increasing BMI (p = 0.008). The ratio of EELV to predicted functional residual capacity was negatively correlated with end-expiratory pressure (Rho = − 0.39, p = 0.01), but not with BMI. Driving pressure and elastance of the respiratory system, chest wall and lung were similar across all ranges of BMI. Likewise, EELV was similar between groups.

**Conclusion:** In ARDS, increasing BMI is associated with increased occurrence of airway closure and increased values of esophageal pressure. Conversely, chest wall elastance is not influenced by BMI, as well as lung elastance. Including BMI in interpreting respiratory mechanics in ARDS patients can provide additional information for the clinical management.

**Compliance with ethics regulations:** Yes.

**Fig. 1:**
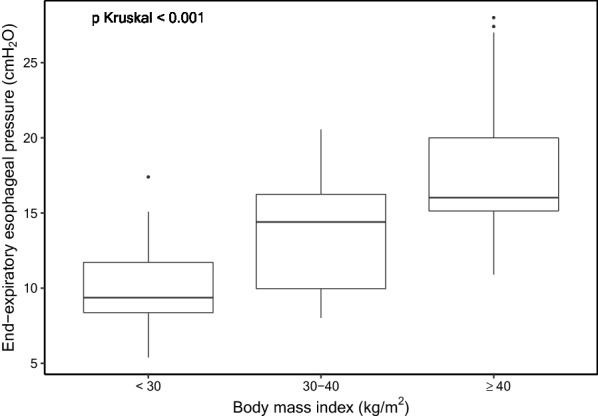
Relationship between body mass index and end-expiratory esophageal pressure measured at a set positive end-expiratory pressure of 5 cmH_2_O

### CO-24 Impact of tidal volume during the “transition period” following neuromuscular blockade cessation in ARDS: an observational study

#### Safaa Nemlaghi^1^, Anne-Fleur Haudebourg^2^, François Perier^2^, Nicolas De Prost^2^, Keyvan Razazi^2^, Guillaume Voiriot^1^, Muriel Fartoukh^1^, Armand Mekontso Dessap^2^, Guillaume Carteaux^2^

##### ^1^Médecine Intensive Réanimation, Hôpital Tenon, Paris, France; ^2^Réanimation Médicale, CHU Henri Mondor, Créteil, France

###### **Correspondence:** Safaa Nemlaghi (safaa.nemlaghi@gmail.com)

*Ann. Intensive Care* 2020, **10 (Suppl 1):**CO-24

**Rationale:** Low tidal volume is the cornerstone of protective ventilation in the initial phase of ARDS (1). Whether such low tidal volume can still be achieved when the patient is allowed to breathe spontaneously under pressure support ventilation (PSV) is unknown. In moderate-to-severe ARDS patients receiving neuromuscular blockade, we assessed the tidal volume and its potential association with the outcome during the “transition period” following neuromuscular blockade.

**Patients and methods:** Retrospective observational study in two university intensive care units. Patients fulfilling moderate-to-severe ARDS criteria less than 72 h after intubation and receiving neuromuscular blockers were included upon entry in the “transition period”. We defined the “transition period” as the 72 h following neuromuscular blockers cessation. Ventilatory and hemodynamic parameters were recorded every 3 h during the “transition period”. Primary outcome was the association between mean tidal volume under pressure support ventilation (PSV) during the “transition period” and the 28-day mortality after adjustment for confounding factors. Data are reported as median [1st–3rd quartile] or number (percentage).

**Results:** One hundred nine patients were included, with a PaO2/FiO2 ratio of 100 mmHg [76–165] at intubation and 194 mmHg [158–244] at inclusion and a SOFA score at 7 [4.5–10]. Patients had been ventilated 2 days [1–3.8] before inclusion. During the “transition period”, 88 patients (80.7%) were switched to PSV. The median duration of PSV was 42 h [27–48]. The mean tidal volume under PSV was significantly lower in survivors than in non survivors at day 28 (7.1 ml/kg [6.3–7.9] vs. 7.8 ml/kg [6.8–9.4] respectively, p = 0.007). By multivariate analysis (Cox proportional hazards regression model), mean tidal volume during PSV remained independently associated with the 28-day mortality after adjusting for SOFA score and immunosuppression. Patients with a mean tidal volume above 8 mL/kg under PSV during the “transition period” had a lower cumulative probability of survival at day 28 as compared with others (Log rank test, p = 0.008) (Fig. 1).

**Conclusion:** In patients with moderate-to-severe ARDS, a higher tidal volume under PSV within the 72 h following neuromuscular blockers cessation is independently associated with the 28-day mortality.**Compliance with ethics regulations:** Yes.

**Fig. 1:**
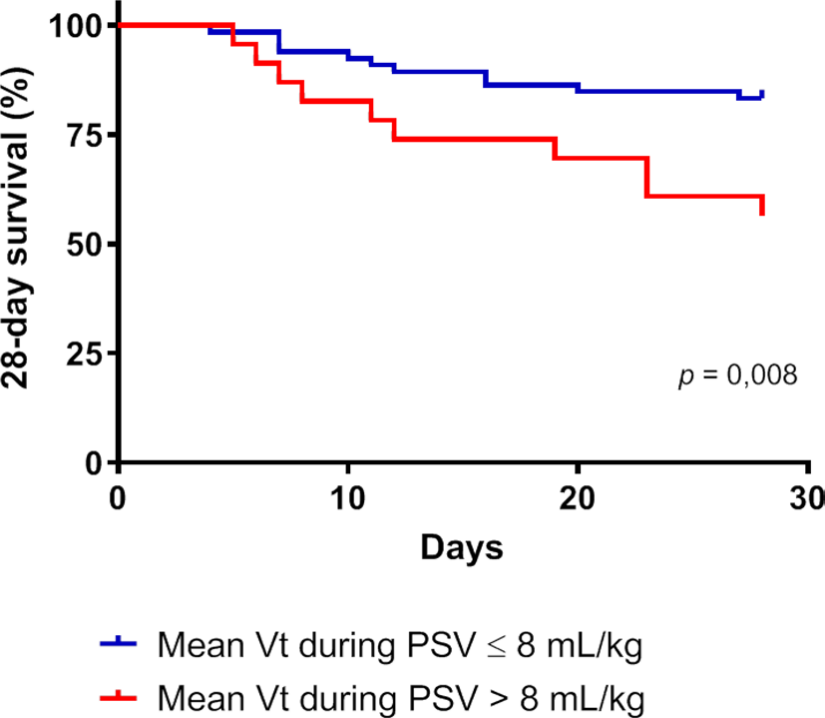
Kaplan-Meier estimate of the cumulative probability of survival according to the mean tidal volume (Vt)—lower of higher than 8 ml/kg—under pressure support ventilation (PSV) during the “transition period”

**Reference**
1. Papazian L, Aubron C, Brochard L, Chiche J-D, Combes A, Dreyfuss D, et al. Formal guidelines: management of acute respiratory distress syndrome. Ann Intensive Care. 2019 Dec;9 (1):69.

### CO-25 Impact of Perioperative Anemia and red blood Cells Transfusion on post-operative complications after oncological surgery

#### Xavier Chapalain^1^, Yves Ozier^1^, Catherine Le Niger^2^, Olivier Huet^1^, Cécile Aubron^3^

##### ^1^Department of anesthesiology and intensive care unit, CHRU de Brest Hôpital de la Cavale Blanche, Brest, France; ^2^Haemovigilance Unit, CHRU de Brest, Brest, France; ^3^Medical Intensive Care unit, CHRU de Brest, Brest, France

###### **Correspondence:** Xavier Chapalain (xavier.chapalain@chu-brest.fr)

*Ann. Intensive Care* 2020, **10 (Suppl 1):**CO-25

**Rationale:** Between 36 and 75% of oncologic patients experience anemia and anemia is associated with poor prognosis. Up to 40% of surgical oncologic patients receive red blood cells (RBC). However, transfusion is associated with adverse events, and equipoise remains on the optimal transfusion strategy in oncologic patients in surgical setting.

**Patients and methods:** This is a retrospective, single center study. All adults admitted to the intensive care unit (ICU) after oncologic surgery from January 2017 to December 2018 were eligible. The following types of surgery for cancer or metastasis resection with a high risk of bleeding were eligible: thoracic, abdominal, neurosurgery, gynecologic, urologic, otorhinolaryngology or spinal surgery. The primary outcome was a composite outcome including post-operative complications (respiratory, cardiac, renal, thromboembolic, infectious and/or hemorrhagic) and/or hospital mortality.

**Results:** Of the 287 patients included, 142 patients (49.5%) had anemia (based on the WHO definition: hemoglobin level 10–11.9 g/dl for female; hemoglobin level 10–12.9 g/dl for male), 69 patients (24%) had moderate anemia (hemoglobin level: 8–9.9 g/dl) and 32 patients (12.5%) severe anemia (hemoglobin level < 8 g/dl). Fifty-six patients (19.6%) received at least one RBC transfusion during their hospital stay. Patients exposed to moderate and severe anemia required more often renal replacement therapy (RRT) for acute kidney injury (AKI) (1.1% vs. 10.9%; p = 0.003), had more surgery-related infections (7.6% vs. 22.8%; p = 0.004). Patients who received RBC had more often AKI with RRT (0.9% vs. 19.6%; p < 0.001), thromboembolic events (2.2% vs. 8.9%; p = 0.039), sepsis (4.3% vs. 16.1%; p = 0.004), pneumonia (4.8% vs. 14.3%; p = 0.024), surgical site infections (8.7% vs. 30.4; p < 0.001) and second surgery for infection (3% vs. 16.1%; p = 0.001). The multivariate analysis found an association between moderate and severe anemia (moderate anemia: OR 15.03 [2.73–282.3]; severe anemia: OR 16.65 [2.71–325.7]; p = 0.011) and severe post-operative complications (Fig. A). There was also an association between RBC transfusion and severe post-operative complications (OR 4.3 [2.2–8.8]; p < 0.001) (Fig. B).

**Conclusion:** Anemia was frequent in oncologic surgical patients. Anemia, including moderate anemia, was independently associated to patient outcomes; however, RBC transfusion also negatively impacts on patients’ prognosis. Our study highlights the need for further research to identify the optimal hemoglobin threshold for RBC transfusion in surgical oncologic patients.

**Compliance with ethics regulations:** Yes.

**Fig. 1:**
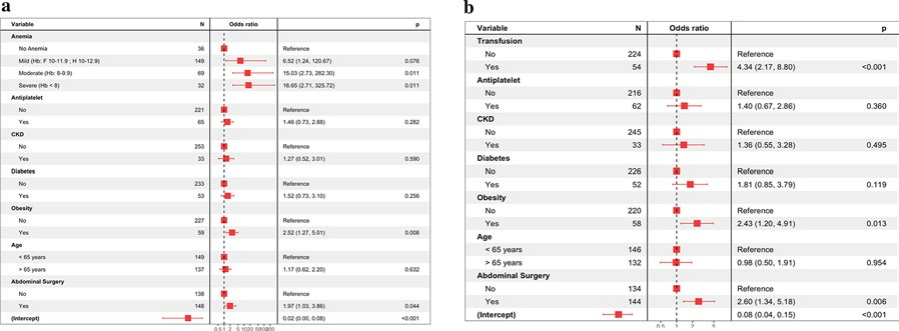
Multivariate analysis analyzing the risk factors for the primary outcome (severe post-operative complications and/or mortality) including anemia (**a**) or transfusion (**b**)

### CO-26 Impact of blood products transfusions on the susceptibility to ICU-acquired infections in septic shock

#### Edwige Péju^1^, Jean-FrançOis Llitjos^1^, Jean-Paul Mira^1^, Jean-Daniel Chiche^1^, Alain Cariou^1^, Julien Charpentier^1^, Matthieu Jamme^2^, Frédéric Pene^1^

##### ^1^Centre Hospitalier COCHIN (APHP), Paris, France; ^2^Centre Hospitalier TENON (APHP), Paris, France

###### **Correspondence:** Edwige Péju (edwigepeju@gmail.com)

*Ann. Intensive Care* 2020, **10 (Suppl 1):**CO-26

**Rationale:** Transfusions of blood products are common in critically ill patients, and have a potential for immunomodulation. The aim of this study is to address the impact of transfusion of blood products on the susceptibility to intensive care unit (ICU)-acquired infections in the high-risk patients with septic shock.

**Patients and methods:** This single-center retrospective study was conducted in the medical ICU of a tertiary care center over a 10-year period (January 2008 to December 2017). All consecutive patients diagnosed for septic shock within the first 48 h of ICU admission were included. Patients who were discharged or died within the first 48 h were excluded. The amounts of red blood cell, platelet and fresh frozen plasma transfusions were collected in all patients, and eventually censored at the onset of ICU-acquired infection. Statistical analysis was carried out using a multivariate time dependent Cox cause-specific proportional hazard model.

**Results:** 1152 patients were admitted for septic shock, with 893 patients remaining alive in the ICU after 48 h of management. The median age was 68 years (58–78), with a majority of male patients (63.7%). Thirty-six percent of them were deemed immunocompromised at the time of ICU admission. A first episode of ICU-acquired infection occurred in 28.3% of the 48-h survivors, with a majority of pulmonary infections (57%). Patients with ICU-acquired infections were more likely to have received red blood cell (52% vs. 38%, p < 0.001), platelet (28% vs. 17%, p = 0.009) and fresh frozen plasma (26% vs. 14%, p < 0.001) transfusions beforehand. In multivariate analysis, transfusion of platelets (OR = 1.45 [1.06–1.99], p = 0.02) and fresh frozen plasma (OR = 1.40 [1.02–1.93], p = 0.03) were independently associated with the occurrence of ICU-acquired infections.

**Conclusion:** Transfusions of platelet and fresh frozen plasma account for independent risk factors of ICU-acquired infections in patients recovering from septic shock. These results support a restrictive transfusion policy in critically ill patients, though indications of platelet and fresh frozen plasma transfusions have not been well investigated in this setting.

**Compliance with ethics regulations:** Yes.

### CO-27 Exploring the microvascular impact of Red Blood Cell Transfusion in intensive care unit patients

#### Geoffroy Hariri^1^, Simon Bourcier^1^, Zora Marjanovic^2^, Jérémie Joffre^1^, Jeremy Lemarie^3^, Jean-Rémi Lavillegrand^1^, Dominique Charue^3^, Thomas Duflot^4^, Naike Bige^1^, Jean-Luc Baudel^1^, Eric Maury^1^, Mohamad Mohty^2^, Bertrand Guidet^1^, Jeremy Bellien^5^, Olivier Blanc-Brude^3^, Hafid Ait-Oufella^1^

##### ^1^Medecine intensive Reanimation, Hôpital Saint-Antoine, Paris, France; ^2^Service d’hématologie, Hôpital Saint-Antoine, Paris, France; ^3^Inserm U970, Paris, France; ^4^Inserm U1096, Rouen, France; ^5^Medecine Inserm U1096, Rouen, France

###### **Correspondence:** Geoffroy Hariri (geoffroyhariri@hotmail.com)

*Ann. Intensive Care* 2020, **10 (Suppl 1):**CO-27

**Rationale:** Red blood cell (RBC) transfusion is a common treatment for hospitalized patients. However, the effects of RBC transfusion on microvascular function remain controversial.

**Patients and methods:** In a medical intensive care unit (ICU) in a tertiary teaching hospital, we prospectively included anemic patients requiring RBC transfusion. Skin microvascular reactivity was measured before and 30 min after RBC transfusion. Plasma was collected to analyze intravascular hemolysis and draw the lipidomic and cytokine profiles.

**Results:** In a cohort of 59 patients, median age was 66 [55–81] years and SAPS II was 38 [24–48]. After RBC transfusion, endothelium-dependent microvascular reactivity improved in 35 (59%) patients, but worsened in 24 others (41%). Comparing clinical and biological markers revealed that baseline blood leucokyte counts distinguished improving from worsening patients (10.3 [5.7; 19.7 vs. 4.6 [2.1; 7.3.109/L; p = 0.001) and correlated with variations of microvascular reactivity (r = 0.36, p = 0.005). Blood platelet count was also higher in improving patients (200 [97; 280] vs 160 [40; 199].103/mL, p = 0.03) but did not correlate with variations of microvascular reactivity. We observed no intravascular hemolysis (HbCO, heme, bilirubin, LDH), but recorded a significant increase in RBC microparticle levels in improving patients after transfusion (292 [108; 531] vs. 53 [34; 99] MP/µL; p = 0.03). The improve in microvascular dilation was positively correlated with RBC microparticle levels (R = 0.83, P < 0.001) and conversion of arachidonic acid into vasodilating eicosanoids.

**Conclusion:** Patients displaying an improved microvascular reactivity after RBC transfusion, had high blood leukocyte counts, increased RBC microparticles formation and enhanced metabolism of arachidonic acid into vasodilating lipids. Our data suggested a contribution of recipient leukocytes to the vascular impact of RBC transfusion.

**Compliance with ethics regulations:** Yes.

### CO-28 Effectiveness of a transfusion protocol to reduce the inappropriate use of red blood cell transfusions in critically ill patients: a before and after study

#### Eric Thiry, Eloïse Natalis, Deeba Ali, Julien Guntz, Philippe Devo, Pierre Demaret

##### chc liège, liège, belgium

###### **Correspondence:** Eric Thiry (eric.thiry@chc.be)

*Ann. Intensive Care* 2020, **10 (Suppl 1):**CO-28

**Rationale:** Red blood cell transfusions (RBCTs) are frequently prescribed in intensive care unit (ICU), but they are an expensive and scarce resource and they are not without risks. Appropriate transfusion strategies are thus required to optimize their use, and interventions aiming to reduce the rate of inappropriate transfusions should be implemented and evaluated.

**Patients and methods:** Prospective observational before-and-after study conducted in a 32-bed ICU affiliated to a non-university teaching hospital. All RBCTs were prospectively recorded during the 4-month-long phases I and III while a transfusion protocol was implemented in the ICU during the 2-month-long phase II. An RBCT was considered as inappropriate if not prescribed in accordance with the protocol.

**Results:** While the number of admissions to ICU was similar between phase I (745 admissions) and phase III (743 admissions), 236 RBCTs were prescribed during phase I versus 191 during phase III, corresponding to a 19%-reduction. We retained 358 RBCTs for analysis (203 during phase I, 155 during phase III). The mean (± standard deviation) pre-transfusion hemoglobin (Hb) was 72 ± 10.5 g/L during phase I, indicating restrictive transfusion practices. Despite this, the proportion of inappropriate RBCTs decreased from 100/203 (49.3%) during phase I to 59/155 (38%) during phase III (p = 0.035). Admission during phase III was independently associated with a reduced risk of inappropriate RBCT (adjusted OR 0.5, 95% confidence interval 0.3–0.85, p = 0.01).

**Discussion:** Our data indicate that an RBCT protocol may improve transfusion practices. However, in a recent international survey, only 29% of the respondents stated that they had an ICU-specific transfusion protocol in their unit. This simple tool is thus probably underused: our findings could help to increase its adoption. Several limitations of our study have to be acknowledged. It may be difficult to assess the appropriateness of some RBCT and our binary classification is probably not appropriate to reflect the complexity of the decisional process leading to some RBCTs. Furthermore, our study is susceptible to selection bias from secular changes in practice from phase I to phase III, and to the Hawthorne effect, i.e. an initial improvement in performance created by the act of observing the performance. Despite these limitations, our findings support the implementation of protocols to improve transfusion practices in ICU.

**Conclusion:** The implementation of an RBCT protocol in our ICU was associated with a 19%-reduction in the number of RBCT prescribed and was independently associated with a 50%-reduction in the risk of being inappropriately transfused.

**Compliance with ethics regulations:** Yes.

### CO-29 Effect of extracorporeal CO_2_ removal on right ventricular function in patients with acute respiratory distress syndrome: the ECCO2Rea pilot study

#### Suzanne Goursaud, Xavier Valette, Julien Dupeyrat, Cedric Daubin, Damien Du Cheyron

##### CHU de Caen Normandie, Service de Réanimation Médicale, 14000 Caen, France

###### **Correspondence:** Suzanne Goursaud (goursaud.suzanne@wanadoo.fr)

*Ann. Intensive Care* 2020, **10 (Suppl 1):**CO-29

**Rationale:** Right ventricular (RV) failure is a common complication in moderate to severe acute respiratory distress syndrome (ARDS). RV failure is exacerbated by hypercapnic acidosis and overdistension induced by mechanical ventilation. Veno-venous extracorporeal CO2 removal (ECCO2R) might allow ultraprotective mechanical ventilation strategy with a low tidal volume (VT) and plateau pressure (Pplat). This study investigated if ECCO2R therapy could have beneficial effects on RV function.

**Patients and methods:** This prospective monocentric pilot study was conducted in a French ICU from January 2017 to March 2019. Patients with moderate to severe ARDS with PaO2/FiO2 ratio between 80 to 150 mmHg were enrolled. Ventilation parameters, arterial blood gases, echocardiographic parameters performed by transthoracic echocardiography (TTE), low-flow ECCO2R system operational characteristics, outcomes and adverse events were collected during the protocol. Primary end point was evolution of RV echocardiographic parameters with ultraprotective ventilation strategy at 4 mL/Kg PBW during the 24-h following the start of ECCO2R.

**Results:** Eighteen patients were included. Efficacy of ECCO2R allowed an ultraprotective strategy in all patients. We observed a significant improvement of RV systolic function parameters assessed by TTE (Fig. 1). Tricuspid annular plane systolic excursion (TAPSE) increased significantly under ultraprotective ventilation compared to baseline (from 22.8 to 25.4 mm; p < 0.05). Systolic excursion velocity (S’) also increased after 1-day protocol (from 13.8 m/s to 15.1 m/s; p < 0.05). A significant improvement of aortic velocity time integral (VTIAo) under ultraprotective ventilation settings was observed. There were no significant differences in the values of systolic pulmonary arterial pressure (sPAP). When patients were separated in two groups according to baseline PaCO2 level above or under 50 mmHg, we showed the deleterious effect of hypercapnia on RV function, and observed in both groups a beneficial impact of an ultraprotective ventilation strategy on TAPSE. No severe adverse events directly related to ECCO2R were observed in our small cohort.

**Conclusion:** The low-flow ECCO2R allows ultraprotective ventilation strategy and improve RV function in moderate to severe ARDS patients. Similarly to prone positioning, ECCO2R could become a strategy that enables to reconcile lung protective approach with RV protective approach in ARDS patients. Large-scale clinical studies, including patients with severe RV dysfunction, will be required to confirm these results and to assess the overall benefits, in particular the best timing of beginning ECCO2R in ARDS patients.

**Compliance with ethics regulations:** Yes.

**Fig. 1:**
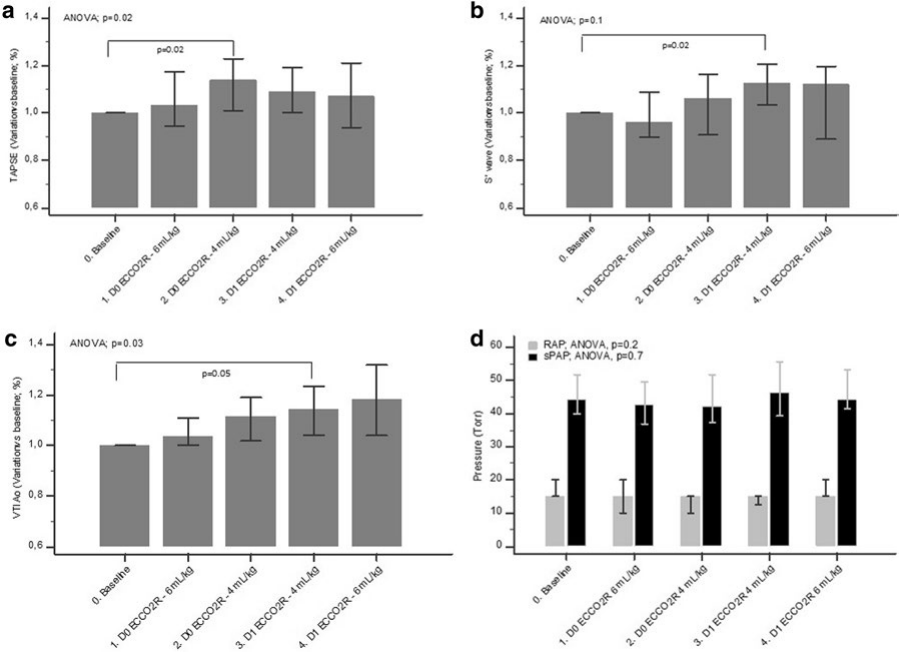
Time course of echocardiography variables during the study period

### CO-30 Typology of Published Randomized Controlled Trials in Critically Ill Patients with Acute Respiratory Failure

#### Guillaume Dumas^1^, Sylvie Chevret^2^, Marine le Corre^3^, Elie Azoulay^1^

##### ^1^Medical Intensive Care Unit, Saint-Louis University Hospital, Paris, France; ^2^ECSTRA team, Biostatistics and clinical epidemiology, UMR 1153 (center of epidemiology and biostatistic Sorbonne Paris Cité, CRESS), INSERM, Paris Diderot University, Paris, France; ^3^Department of Anesthesiology and Critical Care, Groupe Hospitalier Pitié-Salpêtrière Charles Foix, Paris, France

###### **Correspondence:** Guillaume Dumas (dumas.guillaume1@gmail.com)

*Ann. Intensive Care* 2020, **10 (Suppl 1):**CO-30

**Rationale:** We conducted a methodological review of published RCTs on ARF to report success rates according to primary and secondary endpoints.

**Patients and methods:** We searched MEDLINE, Cochrane Central Register of Controlled Trials, and Web of Science for RCTs in critically ill patients with ARF. Published trials in adult patients between January 1995 and December 2018 were evaluated. Clinical endpoints were classified into 3 different categories, namely, ventilation-based endpoints, clinically-based endpoints, or mortality.

**Results:** Seventy-one RCTs were included, which mainly compared oxygenation/ventilation strategies (94%), mostly in patients with various causes of ARF (63%), most often (58%) performed in a single center, and stopped prematurely in 20% of the cases. Twenty-two distinct primary endpoints were used. Classified into three different categories, the most frequently used primary endpoints were ventilation-based (43 RCTs, 60%), and consisted in the need for intubation (79%) assessed at 6 different timepoints followed by clinically-based endpoints in 19 trials (27%), and mortality in 9 (13%). Overall, 41 (58%) RCTs were positive (i.e. demonstrated a significant difference on the primary endpoint). A positive result was reported in 53% of the studies with ventilation-based endpoints, 79% in studies with clinically-based endpoints, and 33% in studies swith mortality endpoints. Adjusted on study quality, 3 factors were associated with a positive result: clinically-based primary endpoints (Odds Ratio (OR): 7.20, 95% Confidence Interval (95% IC): 1.08–48.17, p-value: 0.04), the use of a predefined standard of care in both the control and the intervention groups (OR: 4.60, 95% IC: 1.05–22.79, p-value:) and single center trials (OR: 3.17, 95% IC: 1.03–10.66, p-value:0.05).

**Conclusion:** In critically ill patients with ARF, the majority of published RCTs assessed the effectiveness of oxygenation/ventilation strategies, using the need for intubation as the primary endpoint, and were performed in a single center. The use of a predefined standard of care to manage patients in both groups is associated with more frequent positive trials. These results should be used to frame future trial designs in this field, and guide clinicians and researchers towards optimal research transfer to the bedside.

**Compliance with ethics regulations:** Yes.

### CO-31 Impact of radiation therapy exposure on outcome of cancer patients admitted in an intensive care unit for acute respiratory failure

#### Lucile Martin^1^, Michaël Darmon^2^, Virginie Lemiale^2^, Alexandre Demoule^3^, Frédéric Pene^4^, Anne-Pascale Meert^5^, Achille Kouatchet^6^, djamel MOKART^7^, Fabrice Bruneel^8^, Elie Azoulay^2^, Martine Nyunga^1^

##### ^1^Centre Hospitalier de Roubaix, Roubaix, France; ^2^Hôpital Saint-Louis, Paris, France; ^3^Hôpital Pitié-Salpêtrière, Paris, France; ^4^Hôpital Cochin, Paris, France; ^5^Institut Jules Bordet, Brussels, Belgium; ^6^Centre Hospitalier Régional Universitaire, Angers, France; ^7^Institut Paoli Calmettes, Marseille, France; ^8^Hôpital André Mignot, Versailles, France

###### **Correspondence:** Lucile Martin (lucile.martin.lm@gmail.com)

*Ann. Intensive Care* 2020, **10 (Suppl 1):**CO-31

**Rationale**: Cancer patients are increasingly admitted to the intensive care unit (ICU), and some have been exposed to radiation therapy (RT). Indeed, up to 40% of a cancer population is concerned by radiotherapy. The aim of our study was to assess impact of RT on outcome of these patients admitted in ICU for acute respiratory failure (ARF).

**Patients and methods**: We studied a cohort of cancer patients admitted in ICU for ARF. Data were extracted from 5 prospective and retrospective datasets performed by our study group. Results are reported as median (IQR) or n (%). Adjusted analyses were performed using cox model, then double adjustment (propensity score matching on risk of receiving RT then Cox model).

**Results**: Of the 490 patients, 151 (31%) have been exposed to RT. Preexisting history of surgery (22.1% vs 37.7% in patients without and with RT respectively, p = 0.001), female gender (43.4 vs. 53.0% p = 0.05) and allogeneic stem cell recipients (11.5% vs. 20.5% p = 0.012) had more frequently previous exposure to radiation therapy. Mortality rate on day 28 was 39.7%. RT was not associated with outcome (mortality of respectively 33.1% vs 41.9% in patients with and without RT, p = 0.0875). Lung cancer (p = 0.005) and need for vasopressors (p < 0.0001) were independently associated with mortality. After propensity score for matching, two groups of 122 patients were compared. Then RT was associated to survival (mortality 36.1% vs 52.5% in patients with and without RT respectively, p = 0.01), and did not impact clinical presentation of ARF. After adjustment on confounders, age (HR = 1.03 95% IC = [1–1.07] p = 0.033) and need for vasopressors (HR = 2.48 95% IC = [1.19–5.18] p = 0.015) were associated with mortality. RT was independently associated with survival (HR = 0.51 95% IC = [0.32–0.83] p = 0.0065).

**Conclusion**: This assessment is the first overview of the impact of radiotherapy in this population. Results suggest that exposure to radiation therapy does not worsen prognosis nor survival of cancer patients admitted in the ICU for ARF, regardless the underlying malignancy. Furthermore, radiotherapy is not associated with significant differences in the clinical presentation of respiratory failure. In this study, RT exposure in cancer patients admitted to the ICU for ARF is not associated with a poor prognosis after adjustment for confounders nor to changes in ARF features.

**Compliance with ethics regulations:** Yes.

**Fig. 1:**
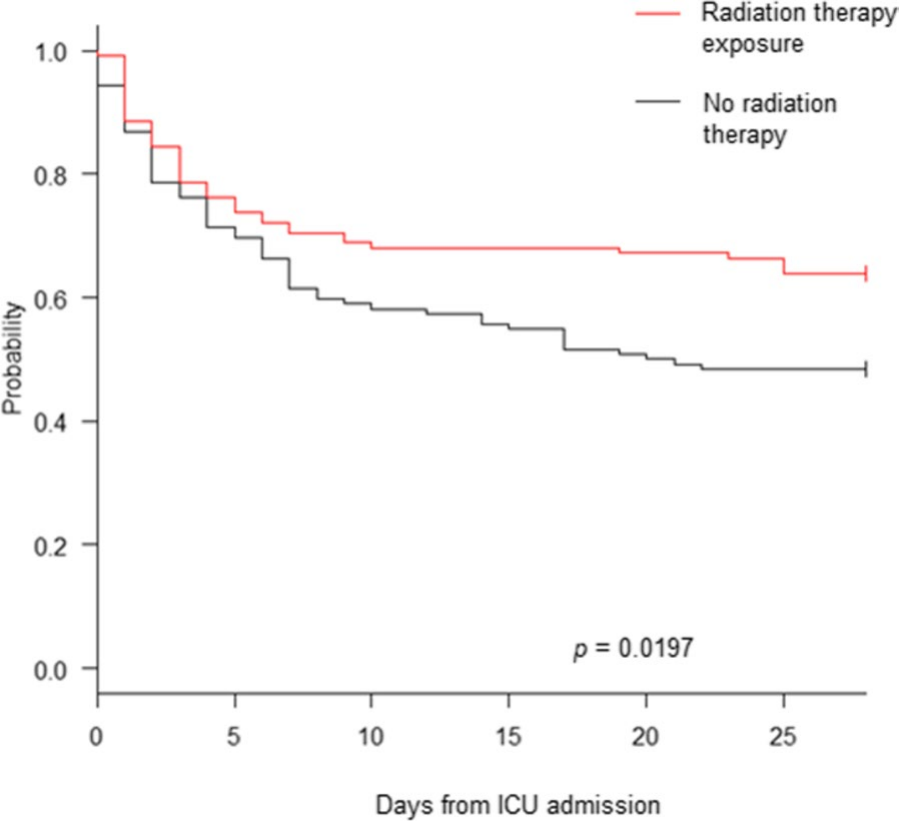
Probability of survival at day-28, after matching

### CO-32 Benefit-to-risk balance of bronchoalveolar lavage in the critically ill. A prospective, multicenter cohort study

#### Toufik Kamel^1^, Julie Helms^2^, Ralf Janssen-Langenstein^3^, Achille Kouatchet^4^, Antoine GUILLON^5^, Jeremy Bourenne^6^, Damien Contou^7^, Christophe Guervilly^8^, Rémi Coudroy^9^, Marie-anne Hoppe^10^, Jean-Baptiste Lascarrou^11^, Jean-Pierre Quenot^12^, Gwenhael Colin^13^, Paris Meng^14^, Jerome Roustan^15^, Christophe Cracco^16^, Mai-anh Nay^1^, Thierry Boulain^1^

##### ^1^Centre Hospitalier Régional d’Orléans, Service de Médecine Intensive Réanimation, Orléans, France; ^2^CHU de Strasbourg-Hôpital Civil, Service de Réanimation Médicale, Strasbourg, France; ^3^Médecine Intensive Réanimation, Hôpital de Haute pierre, Hôpitaux Universitaires de Strasbourg, Strasbourg, France; ^4^CHU d’Angers, Service de Réanimation Médicale et de Médecine Hyperbare, Angers, France; ^5^CHRU de Tours-Hôpital Bretonneau, Service de Réanimation Polyvalente, Tours, France; ^6^Médecine Intensive Réanimation, Réanimation des Urgences, Hôpital La Timone, Marseille, France; ^7^CH d’Argenteuil, Service de Réanimation Polyvalente, Argenteuil, France; ^8^Assistance Publique-Hôpitaux de Marseille, Hôpital Nord, Médecine Intensive Réanimation, Marseille, France; ^9^Médecine Intensive et Réanimation, CHU de Poitiers, Poitiers, France; ^10^CH de La Rochelle-Hôpital Saint-Louis Service de Réanimation Polyvalente, La Rochelle, France; ^11^Service de Médecine Intensive Réanimation, CHU de Nantes, Hôtel Dieu, Nantes, France; ^12^CHU de Dijon-Complexe du Bocage, Dijon, France; ^13^CHD Vendée–Hôpital de la Roche-sur-Yon, La Roche-Sur-Yon, France; ^14^Hôpital Raymond Poincaré, APHP, Service de Médecine intensive Réanimation, Garches, France; ^15^Centre hospitalier de Montauban, service de réanimation polyvalente, Montauban, France; ^16^CH d’Angoulême Service de Réanimation Polyvalente, Angoulême, France

###### **Correspondence:** Toufik Kamel (toufik.kamel@chr-orleans.fr)

*Ann. Intensive Care* 2020, **10 (Suppl 1):**CO-32

**Rationale:** Bronchoalveolar lavage (BAL) is usually deemed to allow the diagnosis of a large array of pulmonary diseases and is usually considered as well tolerated in intensive care unit (ICU) patients. However, recent data suggest that the diagnostic yield of BAL could be rather low (1), and may question its innocuity (2). The present study aimed at assessing the benefit-to-risk balance of BAL in ICU patients.

**Patients and methods:** The study was approved by the appropriate Ethics Committee and registered with clinicaltrials.gov (NCT03098888). In 16 ICUs, from April 2017 to October 2018, we prospectively collected adverse events (AE) during or within 24 h after BAL and assessed the BAL input for decision-making in consecutive adult patients. AEs were categorized in 5 grades of increasing severity. The occurrence of a clinical AE at least of grade 3, i.e. sufficiently severe to need therapeutic action (s), including modification (s) in respiratory support, defined poor BAL tolerance. The BAL input for decision-making was declared satisfactory if it allowed to interrupt or initiate one or several treatments.

**Results:** We included 483 BAL in 483 patients (age 63 yrs [IQR 53–72]; female gender: 162 [33.5%]; Simplified Acute Physiology Score II: 48 [IQR 37–61]; immunosuppression 244 [50.5%], chronic pulmonary disease [163/483 (33.7%)]). BAL was performed either in non-intubated patients receiving standard O2 therapy (n = 56 [11.6%]), or noninvasive ventilation (n = 4 [0.8%]), or high-flow nasal cannula O2 therapy (45 [9.3%]), or in patients under invasive mechanical ventilation (n = 378 [78.3%]). A total of 710 AEs were observed in 415 (85.9%) patients. Sixty-seven (13.9%) patients reached the grade 3 of AE or higher. The main predictor of poor BAL tolerance identified by logistic regression was the association of a BAL performed by a non-experienced physician (non-pulmonologist, or intensivist with less than 10 years in the specialty or less than 50 BAL performed) in non-intubated patients (OR: 31.8 [95% confidence interval 11.6–87.6]; P < 0.0001). Ordinal regression also showed that when BAL was performed by a non-experienced physician in a non-intubated patient, this was associated with an increased risk of AE of any grade (OR: 12.66 [6.27–25.57]). A satisfactory BAL input for decision-making was observed in 227 (47.0%) cases and was not predictable using logistic regression.

**Conclusion:** Adverse events related to BAL in ICU patients are frequent, and sometimes serious. Our findings call for an extreme caution when envisaging a BAL in ICU patients and for a mandatory accompaniment of the less experienced physicians.

**Compliance with ethics regulations:** Yes.

### CO-33 Meningitis is a rare complication of critically ill patients with severe pneumococcal community-acquired pneumonia

#### Paul Jaubert, Julien Charpentier, Jean-Daniel Chiche, Frédéric Pene, Alain Cariou, Guillaume Savary, Marine Paul, Jean-Paul Mira, Mathieu Jozwiak

##### Cochin, Paris, France; ^2^ Mignot, Versailles, France

###### **Correspondence:** Paul Jaubert (paul.jaubert@gmail.com)

*Ann. Intensive Care* 2020, **10 (Suppl 1):**CO-33

**Rationale:** Severe pneumococcal community-acquired pneumonia (PCAP) is a frequent infection requiring intensive care unit (ICU) admission. Pneumococcal meningitis associated with PCAP has been reported and could worsen the prognosis of patients. However, this complication is difficult to predict and lumbar puncture is not systematically performed, regardless the severity of PCAP. Thus, we investigated the characteristics of patients with PCAP associated with pneumococcal meningitis.

**Patients and methods:** We retrospectively included all patients admitted for PCAP in our ICU between 2006 (inception of our electronic medical sheet) and the end of 2018. Community-acquired pneumonia was defined according to the criteria of the American Thoracic Society. We excluded all patients admitted in ICU with initial suspicion of meningitis. Variables regarding epidemiology, clinical and microbiological characteristics, management and prognosis of these patients were collected and analyzed.

**Results:** Among the 264 patients admitted for PCAP (62 ± 17 years old, SAPS II 55 ± 22, 59% of men), 59% of the patients required mechanical ventilation and 29% vasopressors infusion. The ICU mortality was 16%. S. pneumoniae was documented by a positive antigen test in 81% of the patient and/or by a positive sputum smear, tracheal aspirate or distal protected airway specimen in 54% of the patients, and/or by pleural aspirate in 5% of the patients and/or by positive blood culture in 32% (n = 84) of the patients. A lumbar puncture was performed in 39% (n = 33) of the patients with bacteriemia and in 30% (n = 54) of the patients without bacteriemia, with a median delay of 12 h [interquartile range: 6–32] after the onset of antibiotherapy. All lumbar punctures (n = 87) were performed for neurological signs: 50% of coma, 46% of confusion and 1% of seizures. When a lumbar puncture was performed, meningitis was diagnosed in 24% (n = 8) of the patients with bacteriemia and in 2% (n = 1) of the patients without bacteriemia (p < 0.05). The ICU mortality (22% vs. 16%, respectively), age (58 ± 19 vs. 63 ± 17 years old, respectively), SAPS II (65 ± 27 vs. 54 ± 22, respectively) or ICU length of stay (17 ± 24 vs. 11 ± 16 days, respectively) were not different between patients with and without meningitis (each p = NS).

**Conclusion:** Meningitis is a rare complication of PCAP and is more frequent in patients with bacteriemia. Suprisingly, meningitis is not associated with higher ICU mortality. Further analyses are ongoing to identify independent risk factors of meningitis in patients with PCAP.

**Compliance with ethics regulations:** Yes.

### CO-34 Acute varicella zoster encephalitis admitted to the ICU: a French multicentric cohort

#### Adrien Mirouse^1^, Romain Sonneville^2^, Keyvan Razazi^3^, Sybille Merceron^4^, Laurent Argaud^5^, Naike Bige^6^, Stanislas Faguer^7^, Pierre Perez^8^, Guillaume Geri^9^, Claude Guérin^5^, Anne-Sophie Moreau^10^, Laurent Papazian^11^, René Robert^12^, François Barbier^13^, Frédérique Ganster^14^, Julien Mayaux^15^, Elie Azoulay^1^, Emmanuel Canet^16^

##### ^1^Hôpital Saint-Louis, Paris, France; ^2^Hôpital Bichat, Paris, France; ^3^Hôpital Henri Mondor, Créteil, France; ^4^Hôpital André Mignot, Le Chesnay, France; ^5^CHU de Lyon, Lyon, France; ^6^Hôpital Saint-Antoine, Paris, France; ^7^CHU de Toulouse, Toulouse, France; ^8^CHU de Nancy, Nancy, France; ^9^Hôpital Cochin, Paris, France; ^10^CHU de Lille, Lille, France; ^11^CHU de Marseille, Marseille, France; ^12^CHU de Poitiers, Poitiers, France; ^13^CHU de Orléans, Orléans, France; ^14^CH de Mulhouse, Mulhouse, France; ^15^Hôpital Pitié-Salpêtrière, Paris, France; ^16^CHU de Nantes, Nantes, Frances

###### **Correspondence:** Adrien Mirouse (adrien.mirouse@aphp.fr)

*Ann. Intensive Care* 2020, **10 (Suppl 1):**CO-34

**Rationale:** Varicella zoster virus (VZV) is the second most common cause of infectious encephalitis in France. The purpose of this study was to describe the clinical features and prognosis of VZV encephalitis in adult patients admitted to the intensive care unit (ICU).

**Patients and methods:** Retrospective multicenter cohort study including adult patients admitted to 18 French ICUs between 01/01/1999 and 01/09/2017 with a confirmed diagnosis of VZV encephalitis, defined by a positive VZV PCR in the cerebrospinal fluid (CSF). Neurological outcome was evaluated at one year with the modified Rankin Scale (mRS). Data are presented as median [interquartile range] or numbers (percentage).

**Results:** Fifty-five patients (29 men, 53%) with an age of 54 years [36–66] at diagnosis were included. Forty-three patients (78%) had an underlying cause of immunosuppression. Patients were admitted to the ICU 1 day [0–3] after the onset of the first neurological symptoms. The main reasons for ICU admission were confusion (n = 21, 38%), coma (n = 20, 36%) and status epilepticus (n = 10, 18%). At ICU admission, the Glasgow Coma Scale (GCS) score was 12 [7–14], and 17 (31%) patients had a CGS ≤ 8. The SOFA score at day 1 was 6 [4–9]. Twenty-five (55%) patients had fever and 37 (67%) patients had a skin rash. CSF analysis revealed lymphocytic pleocytosis (68 cells/mm^3^ [13–129], lymphocytes 70% [27–95]), mildly elevated CSF protein levels (1.37 g/L [0.77–3.67]) and CSF glucose level of 3.8 mmol/L [2.6–5.0]. A cerebral computed tomography was performed in 33 (60%) patients, normal in 23 (70%) cases, and a brain magnetic resonance imaging was performed in 30 (55%) patients, of whom 21 (70%) had pathologic findings. Patients were treated intravenously with acyclovir 15 mg/kg/day [15–15] during 14 days [9–20]. During ICU stay, 46 (84%) patients required invasive mechanical ventilation, 22 (40%) received vasopressors, and 28 (51%) required renal replacement therapy. The ICU length of stay was 15 days [6–38] and 41 (75%) patients were discharged alive from the ICU. One year after ICU admission, 31 (63%) patients were alive, of whom 20 (41%) had favorable neurological outcome (mRS 0–2), and 18 (37%) were deceased (lost to follow-up, n = 6, 11%).

**Conclusion:** Severe VZV encephalitis in adult patients requiring ICU admission occurs mainly in patients with a history of immune deficiency and is associated with a severe prognosis. At one year, 41% of the patients had a favorable neurological outcome, 22% had moderate to severe disability, and 37% were dead.

**Compliance with ethics regulations:** Yes.

### CO-35 Comparative analysis of host responses to septic and non-septic shock

#### Fabrice Uhel^1^, Brendon Scicluna^1^, Lonneke van Vught^1^, Olaf Cremer^2^, Marc Bonten^3^, Marcus Schultz^4^, Tom van der Poll^1^

##### ^1^Center for Experimental and Molecular Medicine, Amsterdam University Medical Centers, location Academic Medical Center, University of Amsterdam, Amsterdam, Netherlands; ^2^Department of Intensive Care Medicine, University Medical Center Utrecht, Utrecht, Netherlands; ^3^Department of Medical Microbiology, University Medical Center Utrecht, Utrecht, Netherlands; ^4^Department of Intensive Care Medicine, and Laboratory of Experimental Intensive Care and Anesthesiology (L·E·I·C·A), Amsterdam University Medical Centers, location Academic Medical Center, University of Amsterdam, Amsterdam, Netherlands

###### **Correspondence:** Fabrice Uhel (fabrice.uhel@gmail.com)

*Ann. Intensive Care* 2020, **10 (Suppl 1):**CO-35

**Rationale:** Shock is the clinical expression of a circulatory failure that results in inadequate cellular oxygen utilization. Whereas the host response to septic shock has been extensively described, knowledge of the pathogenesis of non-septic shocks remains limited. We aimed to characterize the systemic host response in shock related to non-septic conditions (NSsh) as compared with septic shock (Ssh).

**Patients and methods:** We performed a prospective study in two intensive care units (ICUs) in patients admitted for Ssh (n = 931) or NSsh (n = 1338). Immune responses were determined upon ICU admission by measuring 17 plasma biomarkers reflecting host response pathways implicated in the pathogenesis of critical illness (in 573 Ssh and 287 NSsh patients), and by applying genome-wide blood mRNA expression profiling (in 267 Ssh and 136 NSsh patients).

**Results:** Compared with NSsh, patients with Ssh had more chronic comorbidities, greater disease severity (APACHE IV score 89 [IQR 70–112] vs. 71 [IQR 56–98], p < 0.001) and worse outcomes resulting in higher mortality rates up to one year after ICU admission (53.8% vs. 38.8%, p < 0.001). Plasma biomarker analysis revealed severely disturbed host responses in both Ssh and NSsh patients. However, Ssh patients displayed more prominent inflammatory responses, endothelial cell activation, loss of vascular integrity and a more pro-coagulant state relative to NSsh patients. Blood leukocyte genomic responses were more than 80% common between Ssh and NSsh patients relative to health (Fig. 1A), comprising overexpression of innate pro- and anti-inflammatory pathways, and underexpression of lymphocyte and antigen-presentation gene sets. Direct comparison of Ssh to NSsh patients matched for severity (Fig. 1B) showed overexpression of genes involved in mitochondrial dysfunction and specific metabolic pathways, and underexpression of lymphocyte, NF-κB and cytokine pathways.

**Conclusion:** Patients with Ssh and NSsh present with largely similar host response aberrations at ICU admission; however, patients with septic shock show more dysregulated inflammatory and vascular host responses, as well as specific leukocyte transcriptome alterations consistent with greater metabolic reprogramming and more severe immune suppression.

**Compliance with ethics regulations:** Yes.

**Fig. 1:**
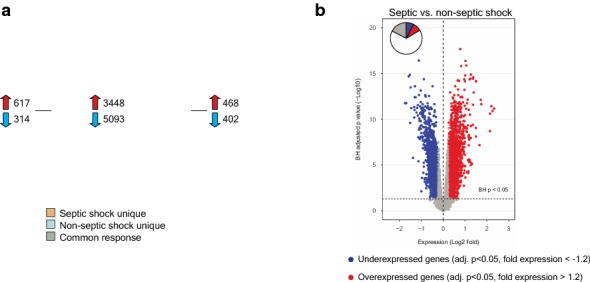
Leukocyte genomic responses in patients admitted to the intensive care unit with a septic or non-septic shock matched for severity upon admission

### CO-36 HepatoDengue: Severity and outcome of acute hepatitis in Dengue Fever in New Caledonia

#### Mathieu Série^1^, Darless Clausse^1^, Marine Noel^1^, Paul Abraham^1^, Carole Forfait^2^, Sophie Gaimard^1^, Delphine Betton^1^

##### ^1^CHT, Nouméa, France; ^2^DASS, Nouméa, France

###### **Correspondence:** Mathieu Série (mathieuserie@aol.com)

*Ann. Intensive Care* 2020, **10 (Suppl 1):**CO-36

**Rationale:** Dengue is a self-limited systemic viral infection transmitted by Aedes with one of four dengue viruses (DENV), and stands for the most common arbovirose worlwide. In 2009, the World Health Organization introduced a revised classification scheme consisting of the three following categories: dengue without warning signs, dengue with warning signs, and severe dengue, the last representing 1% of clinical cases requiring management in the ICU. In New Caledonia, hepatic injuries, seems to become a major cause of case severity during dengue fever epidemy. The aim of this study was to describe evolution and characteristics of hepatic injuries in patients with severe dengue fever admitted in New Caledonia territory-wide healthcare system.

**Patients and methods:** After approval by local ethic committee, all patients admitted in New Caledonia health care system with Dengue diagnosis during 2009 and 2017 epidemics were retrospectively included. Severity (WHO definition), hepatic failure and mortality were compared. Univariate and multivariate analysis between patients with and without severe hepatitis disease were also performed for patients from 2017 epidemy.

**Results:** Respectively 94 and 385 hospitalized patients in New Caledonia from 2009 and 2017 Dengue fever epidemics were included. A significant difference was found between 2009 and 2017 epidemics in terms of severity rates (respectively 15% vs. 34%, p = 0.006), acute hepatitis (AST or ALT ≥ 1000 units/L) (3.1% vs. 13.7%, p = 0.008) but not hepatic dysfunction (PTT < 50%) (Fig. 1). Mortality rates were not different between the two epidemics (3.1% in 2009, 3.3% in 2017), however hepatic dysfunction was associated with mortality. Indeed, in multivariate analysis, patients with acute severe hepatitis were younger (OR 4.74 [1.64–17.70], p = 0.003 for patients between 20 and 30 years old), had more sepsis (OR 3.2, p = 0.01). DENV Serotype 1 and 3 were more frequently associated with hepatitis (OR 6.15 and 10.39, p < 0.05). Absence of acetaminophen intake was also associated with acute severe hepatitis (OR 2.64 [1.21–5.77], p = 0.01).

**Conclusion:** Dengue fever severity seems to be increasing in New Caledonia within last epidemics with a bigger proportion of Hepatitis presentation. Dengue fever patients with acute hepatitis require close monitoring considering their higher mortality, and possibly bigger clinical burden. Acetaminophen in our study appears not to be responsible of this severe acute hepatitis.

**Compliance with ethics regulations:** Yes.

**Fig. 1:**
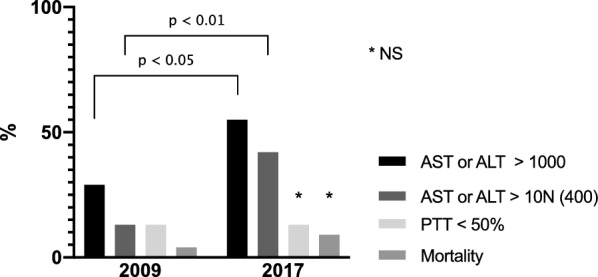
Hepatic injuries for 2009 and 2017 Dengue epidemics

### CO-37 Long-term prognosis after ICU discharge in kidney transplant recipients

#### Romain Arrestier^1^, Nathanael Lapidus^2^, Xavier Monnet^3^, Muriel Fartoukh^4^, Armand Mekontso Dessap^5^, Philippe Grimbert^6^, Antoine Durrbach^7^, Eric Rondeau^8^, Matthieu Jamme^9^

##### ^1^Medical Intensive Care Unit, Henri Mondor University Hospital, Assistance Publique-Hôpitaux de Paris, Créteil, France; ^2^Sorbonne University, INSERM, Institut Pierre Louis d’Epidémiologie et de Santé Publique IPLESP, Public Health Department, Saint-Antoine Hospital, Assistance Publique-Hôpitaux de Paris, Paris, France; ^3^Service de réanimation médicale, Hôpital de Bicêtre. Université Paris-Sud, Faculté de médecine Paris-Sud, Inserm UMR S 999, Le Kremlin-Bicêtre, France; ^4^AP-HP, Hôpital Tenon, Service de Réanimation médico-chirurgicale Faculté de médecine Sorbonne Université Groupe de Recherche Clinique CARMAS, Collégium Gallilée, UPEC, Paris, France; ^5^Service de Réanimation Médicale, Hôpitaux Universitaires Henri Mondor-Albert Chenevier, Assistance Publique-Hôpitaux de Paris. Groupe de Recherche Clinique CARMAS, Université Paris-Est Créteil, IMRB, Créteil, France; ^6^AP-HP, Nephrology and Renal Transplantation Department, Institut Francilien de Recherche en Néphrologie et Transplantation (IFRNT), Groupe Hospitalier Henri-Mondor/Albert-Chenevier Université Paris-Est-Créteil, DHU, VIC, IMRB, Equipe 21, INSERM U 955, Créteil, France; ^7^Nephrology Service, Centre Hospitalier de Bicetre UMRS1197, INSERM, Villejuif University Paris Sud, Orsay, Le Kremlin Bicêtre, France; ^8^Kidney transplant department, Tenon Hospital, Assistance Publique-Hôpitaux de Paris, Paris, France; ^9^Service de Réanimation Médico-Chirurgicale, CHI Poissy/Saint-Germain-en-Laye, Poissy, France

###### **Correspondence:** Romain Arrestier (romain.arrestier@gmail.com)

*Ann. Intensive Care* 2020, **10 (Suppl 1):**CO-37

**Rationale:** After kidney transplantation, 10% of kidney transplant recipients experience a hospitalization in intensive care unit (ICU). However, despite this high prevalence, knowledge about mortality and graft loss for patients discharged alive from ICU is scarce. The objective of our study is to determine incidence and risk factors of long term death and graft loss for these patients.

**Patients and methods:** We conducted a multicenter retrospective study in adult kidney transplant recipients discharged alive from ICU between 2013 and 2018. Data were collected from APHP shared database. Risk factors associated with composite criterion «death and/or graft-loss» were identified with a multivariate analysis by logistic regression, and each component of the composite criterion was studied in a secondary analysis.

**Results:** We identified 118 patients. Median age of patients was 62 years old [50–65] and median follow-up after ICU discharge was 9.6 months [4–21.2]. Nineteen (18.1%) patients died or lost their graft with a median time of 4.5 months [2–8] after ICU discharge. Acute rejection before ICU (OR = 4.3 [1.3; 15.4], p = 0.02) and need for dialysis in ICU (OR = 4.9 [1.6; 16.8], p = 0.01) were associated with the composite criterion. Subgroup analysis found that hospitalization in ICU for acute kidney injury was associated with graft loss (p = 0.038).

**Conclusion:** 18.1% of kidney transplant recipients experience graft loss or death in the first year after ICU discharge. History of acute rejection before ICU and the need of dialysis during hospitalization are associated with a poor outcome.

**Compliance with ethics regulations:** Yes.

### CO-38 Outcome of septic patients with augmented renal clearance after community-acquired infection

#### Romain Courcelle, Diego Castanares, Pierre-François Laterre, Philippe Hantson

##### Departement of intensive care unit, Cliniques Saint-Luc, Université Catholique de Louvain, Brussels, Belgium

###### **Correspondence:** Romain Courcelle (romcourcelle@hotmail.com)

*Ann. Intensive Care* 2020, **10 (Suppl 1):**CO-38

**Rationale:** The true incidence of augmented renal clearance (ARC) has not been well established yet in septic patients with community-acquired infection. In this study, we specifically investigated the effects of ARC on clinical outcome and relapse of infection.

**Patients and methods:** This single-center, retrospective, observational, cohort study included critically ill patients aged more than 18 years-old, hospitalized for a sepsis or a septic shock secondary to a community bacterial infection. Kidney function was assessed by the 24-h measured creatinine clearance (Ccr). ARC patient was defined as Ccr > 130 ml/min/1.73 m^2^. Primary endpoint was mortality at 28 days and at one year. Secondary endpoints were the incidence of ARC, recurrence of infection during hospitalization, the length of intensive care unit (ICU) and hospital stay.

**Results:** From 2015 to 2017, 128 patients were enrolled. Sources of infection were abdomen and lung in respectively 28% and 21% of the patients. Septic shock was present in 75% of the patients and 46% were ventilated. ARC was present in 23% of the patients. ARC patients were younger (p < 0.001), had less of chronic kidney failure (p = 0.001), less cardiovascular risk factors, less chronic cardiac failure (p = 0.023) and had a lower APACHE II score (p = 0.003). The SOFA score tended to be lower in ARC group (p = 0.16). Ccr of ARC patients at day 3 was significantly higher (141 vs. 42 ml/min/1.73 m^2^; p < 0.001). Renal replacement therapy was required in 23% of no-ARC patients vs. 4% of ARC patients (p = 0.014). In Kaplan–Meier analysis, mortality was significantly lower in ARC group at day 28 (10% vs. 38%) and at one year (28% vs. 51%). In multivariate logistic regression, ARC was found to be protective (adjusted OR: 0.1; 95% IC [0.02–0.51]; p = 0.006). SOFA score, cirrhosis and mechanical ventilation were recognized to be associated with mortality. In multivariate Cox regression analysis, ARC was associated with lower mortality (adjusted HR: 0.36; 95% IC [0.16–0.84]; p = 0.018). SOFA score, cirrhosis, mechanical ventilation and renal replacement therapy were found to be risk factors for mortality. There was no difference of the recurrence of infection between both groups (p = 0.89), nor in the length of ICU (p = 0.41) and hospital stay (p = 0.52).

**Conclusion:** In this limited series, we found a positive association between ARC and outcome in community-acquired infection.

**Compliance with ethics regulations:** Yes.

### CO-39 Transition from Acute Kidney Injury to recovery: A cohort study

#### Moustafa Abdel-Nabey, Etienne Ghrenassia, Eric Mariotte, Sandrine Valade, Virginie Lemiale, Lara Zafrani, Elie Azoulay, Michaël Darmon

##### Hopital Saint-Louis, Paris, France

###### **Correspondence:** Moustafa Abdel-Nabey (moustafa.abdelnabey@gmail.com)

*Ann. Intensive Care* 2020, **10 (Suppl 1):**CO-39

**Rationale:** AKI is associated with short and long term mortality and morbidity. Although recovery has been demonstrated to be associated with outcome of critically ill patients, interpretation of available data is limited by time dependent nature of recovery and by competing risks. Our objective was to describe renal recovery, pattern of recovery according to ADQI definitions and risk factor of this later.

**Patients and methods:** Monocenter retrospective cohort study. Adult patients admitted in our ICU from July 2018 to December 2018 were included. AKI was defined according to KDIGO criteria and recovery according to ADQI definition. Incidence of recovery at each time point was depicted using competing risk survival analysis. Risk of transition between AKI and no-AKI was assessed by a semi-Markov model. Last, a trajectoire analysis was performed to depict most frequent recovery patterns. Results are reported as n (%) or median (IQR).

**Results:** 350 patients were included with a median age of 57 (45–72). Median SOFA score at admission was 4 [2–7]. At ICU admission, 85 patients (24.2%) had an AKI stage 1, 44 patients (12.6%) an AKI stage 2 and 37 patients (10.6%) an AKI stage 3. According to ADQI criteria, AKI was defined as rapidly reversed in 42 patients (25.3% of AKI patients), persistent AKI in 16 patients (9.7%) and as acute kidney disease (AKD) in 64 patients (38.6%), remaining patients couldn’t be classified (n = 44).

Risk of recovery was of 9% per day until day 7 then 15% per day (Fig. 1a). Fine and Gray model, taking into account death as competing risk, identified 3 risk factors negatively associated with renal recovery, namely SOFA score (sHR = 0.94 per point; 95% IC = [0.89–0.99]), preexisting hypertension (sHR = 0.56; 95% IC = [0.32–0.98]) and AKI severity (stage 3 vs. stage 1 sHR = 0.17; 95% IC = [0.06–0.56]). Risk of de novo AKI was maximal during the first 7 days and ranged from 25 to 36% per day.

Trajectoire model identified 3 clusters of patients (Fig. 1b), closely associated with patients’ outcome: a) low patients’ severity and no or mild AKI (n = 251; hospital mortality: 8%); b) moderate to severe AKI but little associated organ dysfunction (n = 70, hospital mortality: 12.9%); c) severe AKI and multiple organ failure (n = 29; hospital mortality: 89.7%).

**Conclusion:** This study, assessing AKI recovery patterns, is the first to our knowledge using ADQI definition. Despite the high rate of early recovery and of rapidly reversed AKI, up to 40% of AKI patients had not recovered at day 7 and could therefore be classified has having AKD.

**Compliance with ethics regulations:** Yes.

**Fig. 1:**
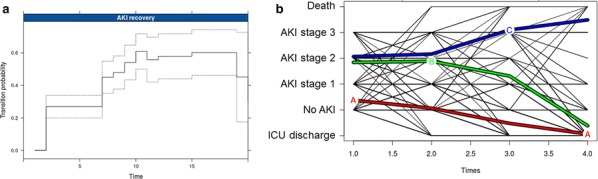
Evolution of the risk of renal recovery and overall patient trajectory

### CO-40 Attributable mortality and host response alterations associated with transient and persistent acute kidney injury in critically ill patients with sepsis

#### Fabrice Uhel^1^, Hessel Peters-Sengers^1^, Fahimeh Falah^1^, Brendon Scicluna^1^, Lonneke van Vught^1^, Marc Bonten^2^, Olaf Cremer ^3^, Marcus Schultz^4^, Tom van der Poll^1^

##### ^1^Center for Experimental and Molecular Medicine, Amsterdam University Medical Centers, location Academic Medical Center, University of Amsterdam, Amsterdam, Netherlands; ^2^Department of Medical Microbiology, University Medical Center Utrecht, Utrecht, Netherlands; ^3^Department of Intensive Care Medicine, University Medical Center Utrecht, Utrecht, Netherlands; ^4^Department of Intensive Care Medicine, and Laboratory of Experimental Intensive Care and Anesthesiology (L·E·I·C·A), Amsterdam University Medical Centers, location Academic Medical Center, University of Amsterdam, Amsterdam, Netherlands

###### **Correspondence:** Fabrice Uhel (fabrice.uhel@gmail.com)

*Ann. Intensive Care* 2020, **10 (Suppl 1):**CO-40

**Rationale:** Sepsis is the most frequent cause of acute kidney injury (AKI). The “Acute Disease Quality Initiative Workgroup” recently proposed new definitions for AKI, classifying it as transient or persistent. We aimed to determine the incidence, attributable mortality and host response characteristics of transient and persistent AKI in patients with sepsis.

**Patients and methods:** We performed a prospective observational study comprising consecutive admissions for sepsis in 2 intensive care units (ICUs) in the Netherlands, stratified according to the presence and evolution of AKI. Attributable mortality fraction (excess risk for dying with persistent AKI relative to transient AKI) was determined using a logistic regression model adjusting for confounding variables. In a subset of 866 sepsis patients, 16 plasma biomarkers indicative of major pathways involved in sepsis pathogenesis were measured. In a second subset of 392 patients, whole-genome blood-leukocyte transcriptomes were analyzed.

**Results:** 1545 sepsis patients were included. AKI occurred in 37.7% (n = 577), of which 18.4% (n = 106) was transient and 81.6% (n = 471) persistent. Patients with persistent AKI had higher disease severity scores on admission than patients with transient AKI or without AKI and more frequently had severe (Injury of Failure) RIFLE AKI-stages on admission (n = 322, 68.4%) than transient AKI patients (n = 33, 31.1%, P < .001). Persistent AKI, but not transient AKI, was associated with increased mortality by day-30 (adjusted OR 2.42, 95% CI 1.28–4.58; P = .006) (Figure) and up to 1-year (adjusted OR 2.10, 95% CI 1.12–3.92; P = .020). The attributable mortality of persistent relative to transient AKI by day-30 was 14.0% (95% CI 3.7–24.2%). Persistent AKI was associated with enhanced and sustained inflammatory and procoagulant responses during the first 4 days, and a more severe loss of vascular integrity compared with transient AKI. Baseline blood gene expression showed minimal differences with respect to the presence or evolution of AKI.

**Conclusion:** Persistent AKI is associated with higher sepsis severity, sustained inflammatory and procoagulant responses, and loss of vascular integrity as compared with transient AKI, and independently contributes to sepsis mortality.

**Compliance with ethics regulations:** Yes.

**Fig. 1:**
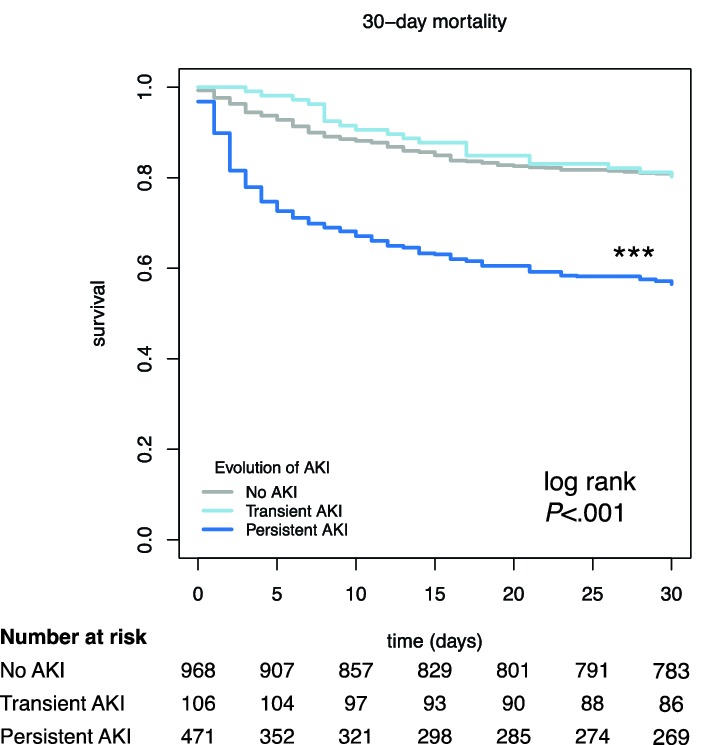
Kaplan-Meier 30-day survival plot of patients with sepsis stratified according to the evolution of acute kidney injury after admission to the intensive care unit

### CO-41 In-hospital cardiac arrest in intensive care units: evolution of patient profile, management and outcomes from 1997 to 2015

#### Clotilde Bailleul^1^, Etienne Puymirat^1^, Philippe Aegerter^2^, Bertrand Guidet^1^, Alain Cariou^1^, Nadia Aissaoui^1^

##### ^1^APHP, Paris, France; ^2^APHP, Boulogne-Billancourt, France

###### **Correspondence:** Clotilde Bailleul (clotilde.bailleul@aphp.fr)

*Ann. Intensive Care* 2020, **10 (Suppl 1):**CO-41

**Rationale:** To address the paucity of data on the epidemiology of patients admitted to intensive care units (ICUs) with in-hospital cardiac arrest (IHCA), we examined key features, mortality and trends in mortality in a large cohort of patients admitted in 33 French ICUs over the past 18 years.

**Patients and methods:** From 1997 to 2015 database of the Collège des Utilisateurs de Bases de données en Réanimation (CUB-Réa), we determined temporal trends in the characteristics of IHCA, patients’ outcomes and predictors of ICU mortality.

**Results:** Of the 376 325 ICU admissions, 33 126 (8.8%) were cardiac arrests and 15 324 were IHCA (4.1%). During the study period, the age of IHCA patients increased by 0.7 years (P = 0.04) and patients presented more comorbidities (chronic heart disease, chronic kidney disease and cancer). Patients were also more critically ill over the period as reflected by the increase of SAPS-II by 2.3% (P < 0.001). Paradoxically, in-hospital management became lighter through the time with reduced respiratory support (p < 0.001), renal support (p < 0.001) and use of vasoactive drugs (p < 0.001). Crude in-ICU mortality decreased from 78% to 62.5% over the past eighteen years (P < 0.001), Fig. 1. In multivariate analysis, a more recent time-period was an independent correlate of decreased mortality [OR 0.40, 95% CI 0.35–0.46]. Factors independently associated with increased ICU mortality were older age [ (age ≥ 75 years; OR 1.71, 95% CI 1.54–1.90], SAPS II [OR 1.03, 95% CI 1.02–1.03], and admission for intracranial hemorrhage [OR 2.95, 95% CI 2.22–3.92]. Interestingly, regarding the associated diagnosis with IHCA, myocardial infarction [OR: 0.62; 95% CI 0.56–0.69] and arrhythmia [OR 0.76, 95% CI 0.68–0.85] were associated with a reduced risk of ICU mortality.

**Conclusion:** Over the 1997–2015 period, IHCA patients became older and more critically ill while the ICU mortality rate decreased suggesting improved overall management. However, mortality rates remain high, warranting further research specifically dedicated to this population.

**Compliance with ethics regulations:** Yes.

**Fig. 1:**
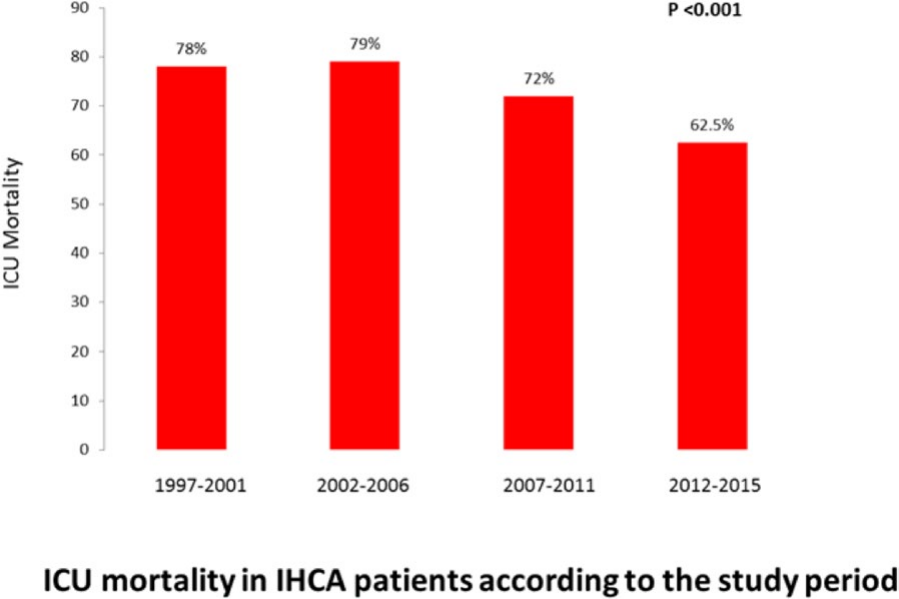
In-ICU mortality in IHCA patients according to time period

### CO-42 Can the cahp score help to predict the mode of death post cardiac arrest?

#### Marine Paul^1^, Sarah Benghanem^2^, Stéphane Legriel^1^, Florence Dumas^3^, Alain Cariou^2^

##### ^1^Intensive Care Unit, Centre Hospitalier de Versailles-Site André Mignot, Le Chesnay, France; ^2^Intensive Care Unit Cochin hospital, Paris, France; ^3^Emergency Department, Cochin hospital, Paris, France

###### **Correspondence:** Marine Paul (marine.1604@hotmail.fr)

*Ann. Intensive Care* 2020, **10 (Suppl 1):**CO-42

**Rationale:** Mortality after cardiac arrest (CA) remains very high, chiefly caused by post-resuscitation syndrome that may lead to refractory shock, severe brain damage and brain death. Withdrawal of care due to comorbid status and recurrence of cardiac arrest also contribute to this poor outcome. The Cardiac Arrest Hospital Prognosis (CAHP) score demonstrated a high discrimination value to predict global outcome after CA. Here we assessed its effectiveness in predicting the mode of death.

**Patients and methods:** We performed a retrospective monocentric study aiming to describe the mode of death after CA according to the CAHP score. All consecutive patients admitted in a tertiary CA center between 2007 and 2017 with a sustained return of spontaneous circulation (ROSC) were studied. Causes of death were recurrent sudden CA, refractory hemodynamic shock, neurological withdrawal of care, brain death and comorbid withdrawal of care. Risk of ICU death was calculated using the validated CAHP score including age, setting, initial cardiac rhythm, no flow as the time from CA to cardio-pulmonary resuscitation (CPR) and low flow as the time from CPR to ROSC, arterial pH, and epinephrine dose. The distribution of death modalities was reported for each of the 3 pre-defined subgroups of CAHP score: low risk (< 150 points), medium risk (150 to 200 points), and high risk (> 200 points).

**Results:** Among 1445 post-CA patients, CAHP score was available for 1293 patients. Median age was 64 years, median no flow and low flow were 3 min (IQR, 0–7) and 17 min (IQR, 10–25), respectively. Patients had an initial shockable rhythm in 50%, received epinephrine during CPR in 67% and 71% of patients developed a post-resuscitation shock. The median CAHP score was 173 (IQR, 135–206). ICU mortality was 62% (799/1293) with the following distribution: neurological withdrawal of care: 45%, refractory hemodynamic shock: 31.3%, brain death: 14.3%, recurrent sudden CA: 5.6% and comorbid withdrawal of care: 3.8%. Causes of death (Figure) differed significantly according to the CAHP subgroups (p < 0.001). Proportion of refractory shock as the cause of death increased in parallel with severity, especially in the most severe subgroup (> 200 points) (p < 0.001).

**Conclusion:** According to the CAHP score, the proportion of post–cardiac arrest patients dying from a refractory hemodynamic shock was more frequent in the most severe patients (CAHP score > 200). The CAHP may help to identify specific subgroups of post-cardiac arrest patients when testing new treatments.

**Compliance with ethics regulations:** Yes.

**Fig. 1:**
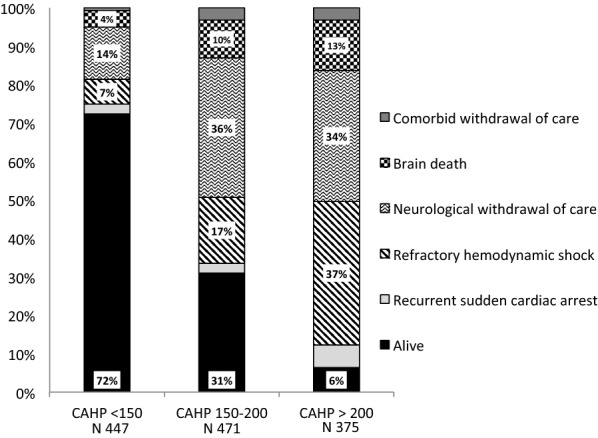
CAHP score and outcome with detailed mode of death

### CO-43 Neuron-Specific Enolase as a Predictor of Death or Poor Neurological Outcome After Out-of-Hospital Cardiac Arrest in patient with nonshockable rhythm

#### Arnaud-Félix Miailhe^1^, Alain Cariou^2^, Gwenhael Colin^3^, Jean-Pierre Quenot^4^, Didier Thevenin^5^, Nicolas Pichon^6^, Sylvie Vimeux^7^, Thierry Boulain^8^, Jean-Pierre Frat^9^, Elisabeth Coupez^10^, Stéphane Legriel^11^, Amélie le Gouge^12^, Bruno Giraudeau^12^, François Desroys du Roure^13^, Jean-Baptiste Lascarrou^14^

##### ^1^Medical Intensive Care Unit, University Hospital Center, Nantes, France; ^2^Medical Intensive Care Unit, Cochin University Hospital Center, Paris, France; ^3^Medical Intensive Care Unit, District Hospital Center, La Roche Sur Yon, France; ^4^Medical Intensive Care Unit, University Hospital, Dijon, France; ^5^Medical-Surgical Intensive Care Unit, General Hospital Center, Lens, France; ^6^University Hospital Center, Limoge, France; ^7^General Hospital Center, Montauban, France; ^8^Regional Hospital Center, Orleans, France; ^9^University Hospital Center, Poitiers, France; ^10^Medical Intensive Care Unit, University Hospital, Clermont Ferrand, France; ^11^Medical Intensive Care Unit, General Hospital, Versailles, France; ^12^INSERM CIC1415, CHRU de Tours, Tours, France; ^13^Biological unit, District Hospital Center, La Roche Sur Yon, France; ^14^Medecine Intensive Reanimation, University Hospital Center, Nantes, France

###### **Correspondence:** Arnaud-Félix Miailhe (flexmiailhe@hotmail.com)

*Ann. Intensive Care* 2020, **10 (Suppl 1):**CO-42

**Rationale:** The prognostication of hypoxic-ischaemic brain injury after resuscitation from cardiac arrest is based on a multimodal approach including biomarkers. The most used biological marker is the neuron-specific enolase (NSE). Currently, no multicenter randomized blinded biomarker study is interested in NSE after out-of-hospital cardiac arrest (OHCA) in patient with a nonshockable rhythm due to any cause. Our goal is to evaluate NSE as a predictor of death and cerebral injuries in this population.

**Patients and methods:** All patients included in this study were part of the HYPERION trial. The HYPERION trial was an blinded-outcome-assessor, multicenter, randomized clinical trial conducted in France between January 2014 and January 2018 with the objective of the randomized multicenter HYPERION trial was to assess whether, compared to targeted normothermia (37 °C). The NSE analysis was performed in 12 intensive care units (ICUs). Serum blood sample were taken from the patients at 24, 48, 72 h after return on spontaneous circulation. All samples were pretreated at the different sites, aliquoted and frozen to − 80 °C before shipment to the biology laboratory of La Roche sur Yon, where the NSE analyses were performed using a COBAS e601 line with an Electro-Chemi-Luminescent—Immuno-Assay.

**Results:** NSE analyses were performed for a total of 106 patients and 237 analyzable blood samples. Median NSE values were 22.6 ng/ml versus 33.6 ng/ml, 18.1 ng/ml versus 76.8 ng/ml, and 9 ng/ml versus 80.5 ng/ml for good versus poor outcome at 24, 48, and 72 h, respectively (p < 0.04; p < 0.0029;p < 0.001). At 48 h and 72 h, NSE predicted neurological outcome with areas under the receiver-operating curve of 0.79 and 0.9, respectively. The cut-off values at 72 h after ROSC provided the best capacity to predict outcome in OHCA in a nonshockable rhythm due to any cause. Management at 33 °C or 37 °C did not significantly affect NSE levels (p = 0.58).

**Conclusion:** We confirm in a large randomized multicentre trial on OHCA in a nonshockable rhythm due to any cause, that NSE values at 72 h are good predictors of poor outcome: for example, a value of 34.9 ng/ml was associated with a poor outcome with a Specificity of 100%.

**Compliance with ethics regulations:** Yes.

**Fig. 1:**
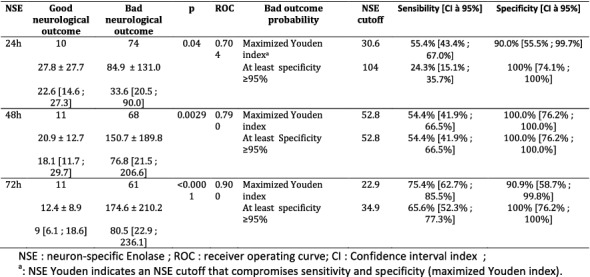
NSE values and NSE cutoff values at 24, 48, 72 h after ROSC for outcome prediction according to CPC at 6 months

### CO-44 Long-term disabilities in awakened survivors of out-of-hospital cardiac arrest: the Hanox study

#### Charles-Edouard Luyt^1^, Alain Cariou^2^, Nicolas Deye^3^, Emmanuel Guérot^4^, Julien Mayaux^1^, Romain Sonneville^5^, Pascale Pradat-Diehl^1^, Anne Peskine^1^

##### ^1^Pitié-Salpêtrière Hospital, Paris, France; ^2^Cochin Hospital, Paris, France; ^3^Lariboisière Hospital, Paris, France; ^4^Georges Pompidou European Hospital, Paris, France; ^5^Bichat Hospital, Paris, France

###### **Correspondence:** Charles-Edouard Luyt (charles-edouard.luyt@aphp.fr)

*Ann. Intensive Care* 2020, **10 (Suppl 1):**CO-44

**Rationale:** Little is known about long-term disabilities of awakened survivors of out-of-hospital cardiac arrest (OHCA). We performed this multicenter prospective observational study to describe 18-month outcome of survivors of OHCA who awoke during the first 2 weeks after cardiac arrest (CA), and risk factors associated with poor outcome.

**Patients and methods:** All survivors of OHCA who awoke during the first 2 weeks after CA (Glasgow coma score ≥ 12) were enrolled in 6 intensive care units from Paris and followed during 18 months (with evaluations performed at 3, 6, 12 and 18 months) in 4 rehabilitation departments. Primary outcome measure was Glasgow outcome scale-extended (GOS-E) at 18 months. Secondary outcome measures included evaluation of neurological, behavioral, and cognitive disabilities, as well as health-related quality of life (compared to sex- and age-matched population), anxiety and depression. Moreover, we explored risk factors of poor outcome (GOS-E ≤ 6) at 18 months.

**Results:** One-hundred and thirty-nine patients were included, of whom 98 were evaluable for the primary outcome measure. At 18-months follow-up, 64 (65%) patients had full recovery or minor disabilities (GOS-E > 6), 18 (18%) had moderate disabilities but were autonomous for activities of daily living (GOS-E = 6), 12 (12%) had poor autonomy (GOS-E < 6 but > 1) and 4 were dead. Proportion of patients with GOS-E > 6 increased significantly over time (Fig. 1). At 18 months, no patients had major neurological disabilities, 20% had cognitive disabilities, 1/3 had anxiety symptoms, 1/4 had depression symptoms, and their quality of life was impaired as compared to sex- and age-matched population. Except risk factors associated traditionally with poor outcome in OHCA patients (no-flow, low-flow, SOFA score at admission and mechanical ventilation at day 3 and 7), no specific risk factors of being classified with a GOS-E ≤ 6 at 18 months were observed.

**Conclusion:** Among patients who awoke (GCS ≥ 12) after OHCA, 35% had moderate–to–heavy disabilities or were dead at 18 months. Health-related quality of life, cognitive and behavioral functions were also altered in some survivors. Risk factors associated with poor outcome (GOS-E ≤ 6) were no- and low-flow times, severity on admission and persistence of mechanical ventilation on day 3 and 7.

**Compliance with ethics regulations:** Yes.

**Fig. 1:**
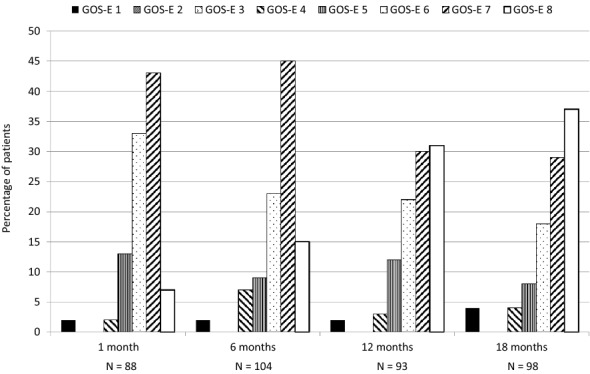
Outcomes of patients during follow-up. Proportion of patients in each Glasgow outcome scale extended (GOS-E) category at 3, 6, 12 and 18 months. Numbers of patients evaluated at each time point are given at the bottom

### CO-45 Non-inferiority study in pediatric cardiac surgery compared a short 24-h antibiotic prophylaxis with 48-h protocol

#### Jeanne Bordet, Lucie Petitdemange

##### CHU Strasbourg, Strasbourg, France

###### **Correspondence:** Jeanne Bordet (jeanne.bordet.pro@gmail.com)

*Ann. Intensive Care* 2020, **10 (Suppl 1):**CO-45

**Rationale:** In surgery, prophylaxis antibiotic aims at preventing the occurrence of post-operative infections. For adults, it is currently recommended to only use prophylactic antibiotic therapy during the time of the intervention. But in pediatric cardiac surgery, there is no consensus around the optimal duration of use of antibiotic prophylaxis. The protocol was modified in 2018 in the ICU and its time reduced to 24 h. We aimed to determine whether 24 h of post-sternotomy antibiotic prophylaxis was not less effective than 48 h treatment to help prevent care-associated infections.

**Patients and methods:** After agreement of the ethics committee of our institution, we performed a retrospective non inferiority study, with an inferiority margin to 10%. The primary objective is to compare the incidence of care-related infections between a second-generation cephalosporin (C2G) antibiotic prophylaxis during 48 h and a 24-h protocols. The secondary objectives are to determine the infection’s incidence, to identify the risk factors for nosocomial infections and to compare the incidence of multidrug-resistant infections.

**Results:** Between January 2013 and July 2019, 402 children underwent cardiac surgeries with sternal opening. 299 received 48 h of C2G antibiotic prophylaxis and 103 received 24 h of C2G treatment. Five previously infected children have been excluded. Both groups were demographically and surgically similar. The median age was 7 months (range a few hours of life to 14.5 years old) and the median weight was 6.7 kg. In the intent-to-treat analysis, incidence of care-related infections is at 10.03% in the C2G-48 h group and 10.68% in the C2G-24 h group. A multivariate analysis shows that the shorter 24-h time antibiotic prophylaxis is not inferior regarding infection prevention compared to 48 h of antibiotic prophylaxis, p = 0.046. As in the per protocole analysis, the C2G-48 h group rate was 9.1% and 6.6% for the G2G-24 h group.

**Conclusion:** It demonstrates that shortening the antibiotic prophylaxis treatment time to 24 h does not affect or increase the rate of infections after a pediatric sternotomy surgery compared to 48-h protocole. Prophylaxis in pediatric cardiac surgery should be short-lived. A multicenter prospective study would allow a consensus and confirm this decision.

**Compliance with ethics regulations:** Yes.

**Fig. 1:**
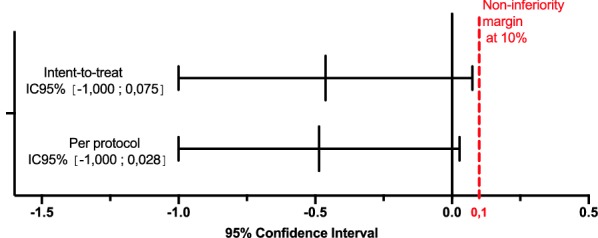
Non-inferiority testing, different in confidence interval of infections rate between intent-to-treat and Per-protocol analysis

### CO-46 Management of pediatric acute liver failure with extra corporeal therapies

#### Megan Nallet-Amate^1^, Benoit Colomb^1^, Etienne Javouhey^2^

##### ^1^Neonatal and Pediatric Intensive Care Unit, Department of Pediatrics, Dijon, Burgundy University, Dijon, France; ^2^Pediatric Intensive Care Unit, Department of Pediatrics, Lyon-Bron, Claude Bernard University, Lyon, France

###### **Correspondence:** Megan Nallet-Amate (megan.amate@gmail.com)

*Ann. Intensive Care* 2020, **10 (Suppl 1):**CO-46

**Rationale:** Pediatric acute liver failure (ALF) is a life-threatening emergency leading to multiorgan failure and requiring management in pediatric intensive care unit (PICU). Stabilization of the child during the waiting time to spontaneous recovery or to liver transplantation (LT) is crucial to prevent evolution to organ failures and death. In this study, we describe our experience on the use of Renal replacement therapy (RRT) in ALF or acute-on-chronic liver failure (AoCLF).

**Patients and methods:** We performed a retrospective, monocenter, observational study in Lyon-Bron PICU. Patients aged from 0 to 18 years, admitted to PICU between January 2010 and January 2018, who presented liver failure (definitions of the PALF Study group) and were treated either with continuous hemodiafiltration combined or not with molecular adsorbents recirculating system (MARS), were included. We compared biological (bilirubin, ammonia, transaminase, creatinine, lactate, aromatic amino acids) and clinical (hepatic encephalopathy clinical and electrophysiological grades) parameters of liver dysfunction before and after replacement therapy (continuous veno venous hemo-dia-filtration (CVVHDF) and MARS.

**Results:** Thirty-nine children were included (medium age: 38 months): 14 (35.9%) underwent MARS treatment, 18 (46.1%) had an AoCLF. Medium PELOD score at admission was 23 [21.0–32.0]. ALF were widely due to congenital (23.1%) or hematologic diseases (20.5%). All biological parameters, except bilirubin, trend to an improvement after 96 h of CVVHDF. Twenty-one children (53.8%) presented clinical signs of encephalopathy. A large part of those children had a grade III encephalopathy (N = 13). We observed a reduction of EEG grade III and IV after 48 h of RRT. Children who received MARS treatment had an improvement of all biological parameters but without statistically significance. The Fischer index increased during MARS treatment from 1.13 to 1.35 (p = 0.09). Overall survival was 62.5% in our cohort, 31% of children survived without liver transplant. For children less than 12 months, we observed a mortality rate of 56%. No side effect of MARS treatment was observed.

**Conclusion:** The results of this observational study suggest that CVVHDF could be a useful treatment to improve liver function (or to stabilize the patient), and that MARS treatment might be an additional safe support. Inscription on liver transplant list should not be delayed, regardless of the initiated technic, according to the literature.

**Compliance with ethics regulations:** Yes.

### CO-47 An automated modified Wood’s clinical asthma score to strengthen respiratory severity assessment in big data

#### Sally Al Omar^1,2^, Alex Lepage-Farrell^1,2^, Atsushi Kawaguchi^1,2,3^, Gabriel Masson^1,4^, Philippe Jouvet^1,2^, Guillaume Emeriaud^1,2^

##### ^1^Pediatric Intensive Care Unit, Sainte-Justine Hospital, Montreal, Canada; ^2^Department of Pediatrics, Université de Montréal, Montreal, Canada; ^3^Department of Pediatrics, University of Ottawa, Ottawa, Canada; ^4^Department of Anesthesiology and Intensive Care Medicine, University Hospital of Lille, Lille, France

###### **Correspondence:** Sally Al Omar (sally.alomar@gmail.com)

*Ann. Intensive Care* 2020, **10 (Suppl 1):**CO-47

**Rationale:** The use of “big data” is getting increasingly popular in the medical field, especially in intensive care where large amounts of data are continuously generated. However, big data can be misleading when essential clinical data are missing. The adequate adjustment for potential confounding factors (e.g., severity of respiratory distress) should be the key procedure in the big data analyses; however, it is challenging to capture the clinical severity within large electronic databases. Bronchiolitis is one main reason for admission to pediatric intensive care unit (PICU). The modified Wood’s clinical asthma score (mWCAS) is widely used to assess the severity of bronchiolitis. The objective of the study is to build an automated mWCAS (A-mWCAS) to continuously assess the severity of respiratory distress in critically ill children.

**Patients and methods:** This retrospective study included all infants < 2 years old with a clinical diagnosis of bronchiolitis, ventilated with non-invasive neurally adjusted ventilatory assist, in a Canadian PICU, between October 2016 and June 2018. We developed an algorithm, using Python 3.7, which was directly connected to the electronic medical record. The components of the score were collected using structured query language (SQL) queries and processed to derive the A-mWCAS. For validation, the A-mWCAS score was compared to the mWCAS manually computed by a clinical expert (M-mWCAS).

**Results:** Sixty-four infants were included in the study, for which 256 of A-mWCAS and M-mWCAS were generated respectively. The Cohen’s Kappa coefficient was applied to estimate the agreement between the two scores which was 0.71 (95% confidence Interval) (Table 1) which corresponds to 78.5% of complete agreement. 17.5% of the A-mWCAS scores were within ± 0.5 of the M-mWCAS. The Kappa coefficient for the each score component were: 0.91 for the oxygen saturation, 0.79 for the expiratory wheezing, 0.91 for the inspiratory breath sounds, 0.89 for the use of accessories muscles and 0.51 for the mental status, respectively.

**Discussion:** The largest discrepancy was observed in the mental status, which clinical evaluation is relatively subjective and varies among care team members (doctor, nurse, respiratory therapist…). The automated score likely decreases this variability by consistently using the same source (respiratory therapist), but its validity should be confirmed in a prospective study.

**Conclusion:** The A-mWCAS provides a valid estimation of the mWCAS that is fast and robust. After external prospective validation, it may help to add some clinical sense within large electronic databases, with improved assessment of the respiratory distress.

**Compliance with ethics regulations:** Yes.

**Fig. 1:**
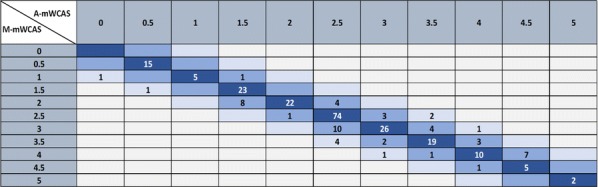
The agreement matrix showing the agreement between the 256 pairs of A-mWCAS and M-mWCAS

### CO-48 The experience of parents of critically ill children during their stay in a French paediatric intensive care unit

#### Clémentine Fort, Etienne Javouhey, Fabienne Bordet

##### Hôpital Femme Mère Enfant, Lyon, France

###### **Correspondence:** Clémentine Fort (clementine.fort@chu-lyon.fr)

*Ann. Intensive Care* 2020, **10 (Suppl 1):**CO-48

**Rationale:** In paediatric intensive care units (PICU), survival rates have dramatically improved. This has been accompanied by increased morbidity, including psychological morbidity. These new impairments, that can affect the survivors and their families have been conceptualized under the frame of post-intensive care syndrome (PICS) and PICS-family. The aim of this study was to explore the experience of critically ill children parent’s during the stay in PICU, and its impact on the family.

**Patients and methods:** We planned a prospective, single centre study for 3 months. We collected qualitative written data from parents whose child had been admitted to the PICU for the first time, for at least two nights.

**Results:** Fifty-seven questionnaires were analysed from thirty-seven admissions. PICU admissions were mostly unplanned. Among parents 40% experienced very painful memories during admission and 61% have feared for their child’s life. During the stay, noise has bothered 30% of parents, and many have described difficulties to rest at night. 81% had the sensation that their child was suffering, mostly from pain, tiredness, anxiety or fear. During PICU stay, 75% of parents had to stop working, and siblings schooling was impacted in 25% of cases, 77% of parents considered themselves to be useful for their child and 81% have participated to nursing care. More than 70% were satisfied about information given and communication, 77% appreciated empathy and support from care givers. Parents received support from family, friends, and also from other parents of hospitalized children. Parents expressed relief (46%) and serenity (38%) to leave PICU, 58% of them were in demand to meet PICU staff again after discharge.

**Conclusion:** PICU parent’s experience is tough, and the impact on family is clear. These are known risks factors for PICS. On a very positive note, parents seemed to be satisfied by family-centred care, and were able to preserve their parental role. However, there is still room for improvement of practices.

**Compliance with ethics regulations:** Yes.

### CO-49 Study of gastrointestinal dysfunction in ventilated adults with shock

#### Paul Decamps^1^, Amélie le Gouge^2^, Julie Boisramé-Helms^3^, Laurent Brisard^4^, Jean-Baptiste Lascarrou^5^, Nadia Anguel^6^, Laurent Argaud^7^, Karim Asehnoune^8^, Pierre Asfar^9^, Anne Bretagnol^10^, Hoang-nam Bui^11^, Emmanuel Canet^12^, Daniel da Silva^13^, Michaël Darmon^14^, Vincent Das^15^, Jérôme Devaquet^16^, Michel Djibre^17^, Maïté Garrouste^18^, Stéphane Gaudry^19^, Olivier Gontier^20^, Claude Guerin^21^, Bertrand Guidet^22^, Christophe Guitton^23^, Jean-Etienne Herbrecht^24^, Jean-Claude Lacherade^25^, Philippe Letocart^26^, Frédéric Martino^27^, Emmanuelle Mercier^28^, Jean-Paul Mira^29^, Saad Nseir^30^, Gaël Piton^31^, Jean-Pierre Quenot^32^, Jack Richecoeur^33^, Jean-Philippe Rigaud^34^, René Robert^35^, Carole Schwebel^36^, Michel Sirodot^37^, Didier Thevenin^38^, Jean Reignier^1^

##### ^1^Service de Médecine Intensive-Réanimation, CHU de Nantes, Nantes, France; ^2^CIC INSERM 1415, Hôpital Bretonneau, Tours, France; ^3^Medical Intensive Care Unit, Nouvel Hôpital Civil, Hôpitaux Universitaires de Strasbourg, Strasbourg, France; ^4^CHU de Nantes, Hôpital Laennec, Département d’Anesthésie et Réanimation, Nantes, France; ^5^Médecine Intensive Réanimation, Chu De Nantes, Nantes, France; ^6^Medical Intensive Care Unit, CHU de Bicêtre, Assistance Publique-Hôpitaux de Paris (AP-HP), Le Kremlin-Bicêtre, Paris, France; ^7^Medical Intensive Care Unit, Hospices Civils de Lyon, Hôpital Edouard Herriot, Lyon, France; ^8^Surgical Intensive Care Unit, Hotel Dieu, CHU de Nantes, Nantes, France; ^9^Medical Intensive Care and Hyperbaric Oxygen Therapy Unit, Centre Hospitalier Universitaire Angers, Angers, France; ^10^Medical Intensive Care Unit, CHR Orléans, Orléans, France; ^11^Medical Intensive Care Unit, Hôpital Pellegrin, CHU Bordeaux, Bordeaux, France; ^12^Médecine Intensive Réanimation, CHU de Nantes, Nantes, France; ^13^Medical-Surgical Intensive Care Unit, Centre Hospitalier de Saint-Denis, Saint-Denis, France; ^14^Medical-Surgical Intensive Care Unit, University Hospital, Saint Etienne, Saint Etienne, France; ^15^Medical-Surgical Intensive Care Unit, Centre Hospitalier Intercommunal André Grégoire, Montreuil, France; ^16^Medical-Surgical Intensive Care Unit, Hôpital Foch, Suresnes, Suresnes, France; ^17^Medical-Surgical Intensive Care Unit, Tenon University Hospital, Assistance Publique-Hôpitaux de Paris (AP-HP), Paris, France; ^18^Medical-Surgical Unit, Hôpital Saint-Joseph, Paris, France; ^19^Medical-Surgical Intensive Care Unit, Hôpital Louis Mourier, Assistance Publique-Hôpitaux de Paris (AP-HP), Colombes, France; ^20^Medical-Surgical Intensive Care Unit, Hôpital de Chartres, Chartres, France; ^21^Medical Intensive Care Unit, Groupement Hospitalier Nord, Hospices Civils de Lyon, Lyon, France; ^22^Medical Intensive Care Unit, Hôpital Saint-Antoine, Assistance Publique-Hôpitaux de Paris (AP-HP), Paris, France^; 23^Medical-Surgical Intensive Care Unit, Hôpital du Mans, Le Mans, France^; 24^Medical Intensive Care Unit, Hôpital de Hautepierre, Hôpitaux Universitaires de Strasbourg, France^; 25^Médecine Intensive Réanimation, Centre Hospitalier Départemental de la Vendée, La Roche sur Yon, France^; 26^Medical-Surgical Intensive Care Unit, Hôpital Jacques Puel, Rodez, France; ^27^Medical-Surgical Intensive Care Unit, CHU de Pointe-à-Pitre, Pointe-à-Pitre, France; ^28^Médecine Intensive Réanimation, Hôpital Bretonneau, CHU Tours, Tours, France^; 29^Intensive Care Unit, Cochin University Hospital, Assistance Publique-Hôpitaux de Paris (AP-HP), Paris, France^; 30^Medical Intensive Care Unit, CHU Lille, Lille, France; ^31^Intensive Care Unit, CHRU Besançon, Besançon, France; ^32^Medical-Surgical Intensive Care Unit, François Mitterrand University Hospital, Dijon, France^; 33^Medical-Surgical Intensive Care Unit, Hôpital de Beauvais, Beauvais, France^; 34^Medical-Surgical Intensive Care Unit, Hôpital de Dieppe, Dieppe, France^; 35^Medical Intensive Care Unit, CHU Poitiers, Poitiers, France; ^36^Medical Intensive Care Unit, CHU Albert Michallon Grenoble, Grenoble, France; ^37^Medical-Surgical Intensive Care Unit, Centre Hospitalier Annecy-Genevois, Metz-Tessy, France^; 38^Medical-Surgical Intensive Care Unit, Centre Hospitalier Docteur Schaffner, Lens, France

###### **Correspondence:** Paul Decamps (paul.decamps@hotmail.fr)

*Ann. Intensive Care* 2020, **10 (Suppl 1):**CO-49

**Rationale:** The Gut has been suspected to be involved in multiple organs dysfunction syndrome (MODS) in the Intensive Care Unit (ICU). Studies suggested a link between gastrointestinal dysfunction (GID) and outcomes. But these studies included very few patients and most of them were retrospective.

**Patients and methods:** This study is a secondary analysis of data from a previous study that included patients from 44 French ICUs. GID is defined as the association of vomiting and constipation or diarrhea during the first week after ICU admission. Patients included were treated with vasopressors and mechanical ventilation. The first goal was to determine if GID is a risk factor of 28-day mortality in this population. Secondary goals were to assess the impact of GID on nosocomial infections.

**Results:** Among 2410 included patients, 238 (9.9%) had GID. By day-28, 76 (32%) of the 238 patients with GID and 780 (36%) of the 2172 patients without GID had died (Odds Ratio 0.84 [0.96–1.01]; p = 0.25). Multivariable regression model did not show any association between gastrointestinal dysfunction and increased risk of 28-day mortality in patients (Odds Ratio 0.76 [0.56–1.04], p = 0.083). Gastrointestinal dysfunction was strongly associated with other secondary outcomes (Table 1). Patients with GID had longer ventilation duration, ICU length of stay and hospital length of stay. They also had more nosocomial infections, in particularly ventilator-associated pneumonia. This association still existed in a multivariable regression model for prediction of nosocomial infection including the same variables than the previous model (Odds Ratio 1.68 [1.19–2.37], p = 0.0028). No association with day-90 mortality was observed.

**Conclusion:** Gastrointestinal dysfunction was not a risk factor of day-28 mortality but was associated with an increased risk of nosocomial infection and an increased length of stay. This study is observational and no causality link can be done. However, our data suggest further studies on strategies aimed to limit GID.

**Compliance with ethics regulations:** Yes.

**Table 1:**
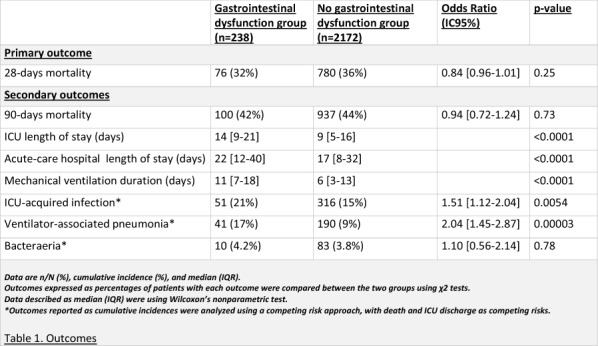
Outcomes

### CO-50 Incidence and risk factors of upper digestive tract ischemia after Out-of-Hospital Cardiac Arrest, the ENTRACT study

#### David Grimaldi^1^, Stéphane Legriel^2^, Nicolas Pichon^3^, Philippe Colardelle^2^, Sarah Leblanc^4^, Florence Canoui-Poitrine^5^, Omar Ben Hadj Salem^6^, Gregoire Muller^7^, Nicolas de Prost^8^, Sofia Herrmann^9^, Sophie Marque^10^, Aurore Baron^10^, Bertrand Sauneuf^11^, Jonathan Messika^12^, Jacques Creteur^13^, Jean pierre Bedos^14^, Fabio Taccone^13^, Emmanuelle Boutin^5^, Alain Cariou^6^

##### ^1^USI Hôpital Erasme, Bruxelles, Belgium; ^2^CH Versailles, Le Chesnay, France; ^3^CHU Dupuytren, Limoges, France; ^4^Gastro-enterologie CHU Cochin, Paris, France; ^5^Biostatistique CHU henri Mondor, Creteil, France; ^6^CHU Cochin, Paris, France; ^7^MIR CH Orléans, Orléans, France; ^8^CHU Henri Mondor, Creteil, France; ^9^Gastroentérologie CH Orléans, Orléans, France; ^10^Réanimation CH Sud Francilien, Corbeil, France; ^11^Réanimation CH Public du Cotentin, Cherbourg, France; ^12^Réanimation CHU Louis Mourier, Colombes, France; ^13^USI Hôpital Erasme, ULB, Bruxelles, Belgium; ^14^Réanimation CH Versailles, Le Chesnay, France

###### **Correspondence:** David Grimaldi (david.grimaldi@erasme.ulb.ac.be)

*Ann. Intensive Care* 2020, **10 (Suppl 1):**CO-50

**Rationale:** Post-cardiac arrest (CA) ischemia/reperfusion induces organ injury. Gastro-intestinal ischemia can lead to endotoxin translocation and worsening of organs failure. The ischemic injury of the gastro-intestinal (GI) tract after CA is suggested by digestive biomarkers and by frequent endotoxemia. However its incidence and its risk factors after out-of-hospital CA are unknown. The main objective was to measure the incidence of upper digestive tract ischemia after OHCA. The secondary objectives were to determine its severity and its risk factors.

**Patients and methods:** The ENTRACT study was a prospective, interventional, non-controlled, multicentric study. Included patients underwent a gastroscopy 2 to 4 days after OHCA if still intubated. Digestive ischemia was determined by the gastroenterologist. Severe lesion were defined as ulceration or necrosis, mild as mucosal oedema or erythema. We compared the patients’ caracteristics available before the gastroscopy according to the presence or not of digestive ischemia. Multivariable regression analysis were performed to identify variables associated with digestive ischemia.

**Results:** 221 patients were included in 9 ICUs, 214 were suitable for complete analysis. Mean age was 62, 74% were male. CA was witnessed in 90%, initial rhythm was VT/VF in 53%, median no-flow was 5 (0–9) min and low flow 21 (15–30) min. 121 (57% 95% IC 50–63%) patients had at least one ischemic lesion of upper digestivetract including 103 (85%) that didn’t present any digestive symptoms before endoscopy. Fundus was the most frequent localization of ischemia followed by antrum, duodenum and esophagus. Ischemic lesions were severe in 55/121 (45%) patients and mild in the others. Patients with digestive ischemia compared to patients without had similar demographic characteristics but were less frequently treated with PPI before CA (16% vs 31%), frequence of NSAID use (including aspirin) and H. pylori positive serology were similar in both groups. CA characteristics were similar except that patients with digestive ischemia received more epinephrine (2 (1–4) vs 1 (0–3) mg) despite similar low-flow. Multivariate regression identified peripheral artery disease (OR 0.3 (0.09–0.94)), previous PPI (OR 0.49 (0.23–1.03)) and hypochloremia (OR 0.9 (0.84–0.97)) as protective factors whereas hematocrit (OR 1.04 (1.00–1.09)) and epinephrine dose during CPR (OR 1.17 (1.03–1.32)) were associated with upper digestive ischemia.

**Conclusion:** In this multicentric study, upper digestive tract ischemia was frequent after CA (57% of the patients) and was associated with epinephrine dose during CPR. Digestive ischemia involved mainly the stomach, necrosis was rare. Main limitation is that patients extubated or dead before day-2 were not included.

**Compliance with ethics regulations:** Yes.

### CO-51 Severe acute cholangitis in French ICUs: a retrospective multicenter study. ICUte Cholangitis group

#### Jean-Rémi Lavillegrand^1^, Emmanuelle Mercier-des-Rochettes^1^, Claire Pichereau^2^, Gaël Piton^3^, Baron Elodie^4^, Frédéric Pene^4^, Idriss Razach^5^, Merad Monchi^6^, Damien Contou^7^, Gabriel Preda^8^, Chloe Molliere^9^, Arnaud Galbois^9^, Arnaud-felix Miailhe^10^, Jean Regnier^10^, Raphael Favory^11^, Guillaume Dumas^12^, Hafid Ait-Oufella^1^

##### ^1^Réanimation, Hopital Saint-Antoine, Paris, France; ^2^Réanimation, CH de Poissy, Poissy, France; ^3^Réanimation, CHU Besançon, Besançon, France; ^4^Réanimation, Hopital Cochin, Paris, France; ^5^Réanimation, CH de fontainebleau, Fontainebleau, France; ^6^Réanimation, CH de Melun, Melun, France; ^7^Réanimation, CH V Dupouy, Argenteuil, France; ^8^Réanimation, CH de Saint-Denis, Saint-Denis, France; ^9^Réanimation, CH Claude gallien, Qincy Sous Senart, France; ^10^Réanimation, CHU de Nantes, Nantes, France; ^11^Réanimation, Hopital Salengro, Lille, France; ^12^Réanimation, CHU Saint-Antoine, Paris, France

###### **Correspondence:** Jean-Rémi Lavillegrand (JRLavillegrand@gmail.com)

*Ann. Intensive Care* 2020, **10 (Suppl 1):**CO-51

**Rationale:** Acute cholangitis (AC), a bacterial infection related to an obstruction of the biliary tree, may be responsible for life-threatening organ failure. However, little is known about the outcome and the predictive factors of mortality of critically ill patients admitted in ICU for acute cholangitis. We aimed to describe characteristics of patients admitted in ICU for AC and to analyze predictive factors of in-hospital mortality including the time to biliary drainage procedure.

**Patients and methods:** Retrospective study of all cases of acute cholangitis admitted in 11 French ICUs (5 tertiary hospitals and 6 non-tertiary hospitals) for continuous period between 2005 to 2018. Data are expressed as percentage or median (1^st^ IQR; 3^rd^ IQR).

**Results:** Overall, 382 patients (age: 72 [62; 81] years; 64% men) were included, in-hospital mortality was 29%. SOFA score at admission was 8 [5; 11] and SAPS2 was 50 [38; 64]; 38% of patients underwent mechanical ventilation and 61% received vasopressor therapy. Biliary obstruction was mainly related to gallstone (53%) and cancer (22%).

Median total bilirubin and PCT were respectively, 83 [50; 147] µmol/L and 19.1 [5.3; 54.8] µg/L. 63% of patients (n = 252) have positive blood culture, mostly Gram negative bacilli (86%) and 14% producing extended spectrum beta lactamase Enterobacteriaecae. At ICU admission, persisting obstruction was frequent (79%) and therapeutic endoscopic retrograde cholangiopancreatography was performed in 76% of them.

In a multivariable analysis, at ICU admission, several factors were significantly associated with in-hospital mortality: SOFA score (OR = 1.14 [95% IC 1.05; 1.24] by point, p = 0.001), arterial lactate (OR = 1.21 [1.08; 1.36] by 1 mmol/L, p < 0.001), total serum bilirubin (OR = 1.26 [1.12; 1.41] by 50 umol/L, p < 0.001), obstruction non-related to gallstones (P < 0.05) and AC complications (liver abcess and/or pancreatitis) (OR = 2.74 [1.45; 5.17] p = 0.002). In addition, time > 48 h between ICU admission and biliary drainage was associated to in-hospital mortality (adjusted OR = 2.73 [1.30; 6.22] p = 0.02).

**Conclusion:** Acute cholangitis is responsible for high mortality in ICU. Organ failure severity, causes and local complications of cholangitis are predictive factors of mortality as well as delayed biliary drainage.

**Compliance with ethics regulations:** Yes.

### CO-52 Liver transplantation for critically ill cirrhotic patients

#### Thierry Artzner^1^, Baptiste Michard^1^, Emmanuel Weiss^2^, Louise Barbier^3^, Zaid Noorah^4^, Jean-Claude Merle^4^, Catherine Paugam^2^, Claire Francoz^2^, Francois Durand^2^, Olivier Soubrane^2^, Tasneem Pirani^5^, John O’grady^5^, Nigel Heaton^5^, Eleni Theocharidou^5^, William Bernal^5^, Ephrem Salame^3^, Helene Barraud^3^, Petru Bucur^3^, Francois Lefebvre^1^, Lawrence Serfaty^1^, Camille Besch^1^, Philippe Bachellier^1^, Francis Schneider^1^, Eric Lavesque^4^, François Faitot^1^

##### ^1^Hôpital de Hautepierre, Strasbourg, France; ^2^Hôpital Beaujon, Clichy, France; ^3^Hôpital Trousseau, Tours, France; ^4^Hôpital Henri Mondor, Créteil, France; ^5^King’s College Hospital, London, UK

###### **Correspondence:** Thierry Artzner (thierry.artzner@gmail.com)

*Ann. Intensive Care* 2020, **10 (Suppl 1):**CO-52

**Rationale:** Cirrhotic patients with acute-on-chronic liver failure associated with extra-hepatic organ failure have dismal transplant-free prognosis in the intensive care unit. Liver transplantation is the only effective treatment available for them and a number of studies have shown promising results for critically ill cirrhotic patients. However, there are no recommendations or data in the literature to guide clinicians who need to decide what the optimal transplantability window for these patients is and which patients are too sick to be transplanted. The aim of this study was to develop a prognostic model to predict post-transplantation survival in critically ill cirrhotic candidates for liver transplantation with multiple organ failure.

**Patients and methods:** Patients with grade-3 acute-on-chronic liver failure (ACLF-3) who underwent liver transplantation between January 1, 2007 and June 30, 2017 in 5 transplant centers (4 in France and 1 in the United Kingdom) were included (n = 152). Predictors of one-year mortality were retrospectively screened and tested on a single center training cohort. A predictive score was developed and tested on an independent multicenter cohort.

**Results:** Four independent pre-transplantation risk factors were associated with one-year mortality after transplantation in the training cohort: age ≥ 53 years (OR = 5.79, 95% CI = 1.05–32.04, p = 0.044), pre-transplantation arterial lactate level ≥ 4mml/l (OR = 9.99, 95% CI = 1.62–61.46, p = 0.013), mechanical ventilation with PaO2/FiO2 ≤ 200 mmHg (OR = 8.60, 95% CI = 1.30–56.88, p = 0.026) and pre-transplantation leukocyte count ≤ 10G/l (OR = 12.91, 95% CI = 2.26–73.87, p = 0.004). A simplified version of the model was derived by assigning 1 point to each risk factor: the Transplantation for ACLF-3 Model (TAM) score. A cut-off at 2 points distinguished a high-risk group (score > 2) from a low-risk group (score ≤ 2) with one-year survival of 8.3% vs. 83.9% respectively (p < 0.001). The model and its simplified version were validated on the independent multicenter cohort. There was a significant difference between the high-risk and low-risk group with one-year survival of 10% vs. 71.9% respectively (p < 0.001).

**Conclusion:** Liver transplantation can be an effective treatment for critically ill cirrhotic patients with hepatic and extra hepatic organ failure provided patients are carefully selected and that they are transplanted at the optimal time in the intensive care. The TAM score can help stratify post-transplantation survival and assist clinicians in the transplantation decision-making process at the bedside of ACLF-3 patients.

**Compliance with ethics regulations:** Yes.

**Fig. 1:**
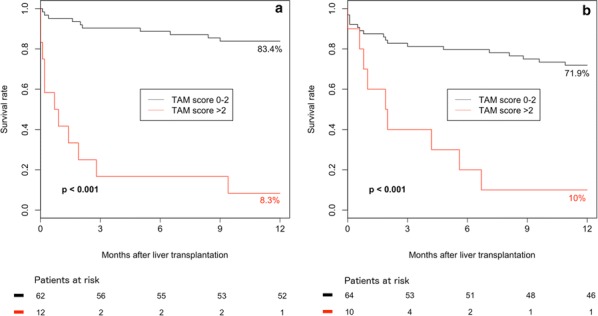
Survival rate after liver transplantation in the training cohort (A, n = 74) and in the validation cohort (B, n = 74) in high risk (red line) and low risk (black line) patients

### CO-53 Does Echocardiography Improve Clinical Outcomes in Shock States?

#### Habiba Hemamid, Mosbah Nabil, Abdelmalek Hakimi

##### CHU de Sétif, Setif, ALGERIA

###### **Correspondence:** Habiba Hemamid (habibahemamid@yahoo.com)

*Ann. Intensive Care* 2020, **10 (Suppl 1):**CO-53

**Rationale:** Trans-thoracic echocardiography (TTE) is commonly used in the initial management of patients with shock in ICU. There is little published evidence for any mortality benefit. We compared the effect of echocardiography protocol versus standard care for survival and clinical outcomes.

**Patients and methods:** This randomized controlled trial included selected shocked patients (systolic blood pressure < 90 mm Hg and signs of organ hypoperfusion) randomized to early TTE plus standard care versus standard care without TTE. The primary outcome measure was survival to 28 days. Secondary outcome measures included initial treatment and vasopressor weaning.

**Results:** 202 consecutive subjects with circulatory shock (low systolic arterial blood pressure (SAP) and signs of organ hypoperfusion) at the time of ICU admission are included in the study. In the TTE group: fluid prescription during the first 24 h was significantly lower (1519.62 ± 786.19 ml vs 1828.88 ± 1158.94 ml, P = 0.042), the vasopressor dose during the first 24 h was significantly lower (0.582 µg/kg/min [0.497–0.667] vs. 0.458 µg/kg/min [0.373–0.543] p = 0.044). TTE patients were weaned of vasopressors more quickly than those in the no TTE group (38.4 ± 20.88 vs. 57.8 ± 33.6 h p = 0.005). TTE patients received more dobutamine during the 72 h (165 versus 82 prescriptions). We found no important difference between groups for the primary outcome of survival (TTE group 61 of 100 patients versus standard care 67 of 102 patients).

**Conclusion:** TTE guided management was associated with, less fluid, less vasopressor dosing, faster vasopressor weaning and increased inotropic prescription. But TTE did not improve survival.

**Compliance with ethics regulations**: Yes.

### CO-54 Prognostic assessment of left ventricular diastolic and systolic function during septic shock

#### Philippe Vignon^1^, Cyril Charron^2^, Marine Goudelin^3^, Annick Legras^4^, Michel Slama^5^, Gwenael Prat^6^, Stein Silva^7^, David Vandroux^8^, Gregoire Muller^9^, Bruno Lévy^10^, Florence Boissier^11^, Bruno Evrard^3^, Xavier Repessé^2^, Julien Maizel^5^, Guillaume Geri^2^, Julie Leger^12^, Antoine Vieillard-Baron^2^

##### ^1^Réanimation polyvalente/Inserm CIC 1435/Inserm UMR 1092, CHU Dupuytren/Université de Limoges, Limoges, France; ^2^Médecine Intensive Réanimation, CHU Ambroise Paré, APHP, Boulogne Billancourt, France; ^3^Réanimation polyvalente/Inserm CIC1435, CHU Dupuytren, Limoges, France; ^4^Médecine Intensive Réanimation, CHU Bretonneau, Tours, France; ^5^Réanimation médicale, CHU Amiens, Amiens, France; ^6^Réanimation médicale, CHU Brest, Brest, France; ^7^Réanimation polyvalente, CHU Purpan, Toulouse, France; ^8^Réanimation polyvalente, CHU Félix Guyon, Saint Denis De La Réunion, France; ^9^Réanimation polyvalente, CHR d’Orléans, Orléans, France; ^10^Réanimation médicale, Hôpitaux de Brabois, CHU de Nancy, Vandoeuvre-Lès-Nancy, France; ^11^Réanimation médicale, CHU La Milétrie, Poitiers, France; ^12^Inserm CIC 1415, CHU Bretonneau, Tours, France

###### **Correspondence:** Philippe Vignon (philippe.vignon@unilim.fr)

*Ann. Intensive Care* 2020, **10 (Suppl 1):**CO-54

**Rationale:** Both the negative prognostic value and reversibility of left ventricular (LV) diastolic dysfunction in septic patients remain debated. The excess of mortality in septic shock patients with hyperdynamic profile has only been reported by small-size studies. Accordingly, the primary objective of the PRODIASYS study was to assess the impact of LV diastolic dysfunction (and its severity) and of LV hyperkinesia echocardiographically identified during the initial phase of septic shock on 28-day survival. The secondary objective was to assess the potential link between LV diastolic dysfunction, cumulative water balance (on day 4), and outcome.

**Patients and methods:** This was a multicenter, prospective, observational, cohort study. Patients older than 18 years hospitalized in ICU for septic shock (Sepsis-3 definition) were eligible. Exclusion criteria were administration of inotropes, severe left valvular disease, constrictive pericarditis and moribund patients. In each patient, echocardiography was first performed within 12 h after the diagnosis of septic shock and then daily until Day 3, after vasopressor discontinuation, at ICU discharge and on Day 28 or at hospital discharge, whichever occurred first. Vital and biological parameters usually monitored for septic shock management were collected at each echocardiographic assessment. Vital status was collected on Day 28. Associations between LV diastolic dysfunction or LV hyperkinesia and Day-28 mortality were analyzed using a Chi2 test. Adjusted analyses were performed using logistic regression models, including variables known to be linked with the prognosis of septic shock (e.g., severity scores, delay of antibiotherapy). The relationship between the grade (I to III) of LV diastolic dysfunction and 28-day survival were analyzed using a logistic regression model. The relationship between the presence of LV diastolic dysfunction and cumulated water balance on Day 4 were analyzed using a linear regression model adjusted on the body weight on admission. The relationship between the grade of LV diastolic dysfunction and cumulated water balance on Day 4 were analyzed using a linear regression model.

**Results:** From January 2017 to September 2019, the study enrolled the initially planned 440 patients for a total of more than 1400 echocardiographic examinations performed in the 10 participating sites.

**Conclusion:** This study promises to determine whether LV diastolic dysfunction using up-to-date consensual definition is independently linked to mortality of septic shock patients or not, whether it is associated with positive fluid balance and potentially reversible in survivors, and whether the hyperkinetic hemodynamic profile is prognostic in septic shock.

**Compliance with ethics regulations:** Yes.

### CO-55 Perfusion index: a non-invasive way to detect the effects of the end-expiratory occlusion test in critically ill patients

#### Alexandra Beurton, Francesco Gavelli, Jean-Louis Teboul, Nello De Vita, Xavier Monnet

##### Service de médecine intensive-réanimation, Hôpital de Bicêtre, Hôpitaux universitaires Paris-Saclay, Assistance publique—Hôpitaux de Paris, Inserm UMR S_999, Université Paris-Su, Le Kremlin-Bicêtre, France

###### **Correspondence:** Alexandra Beurton (alexandra.beurton@aphp.fr)

*Ann. Intensive Care* 2020, **10 (Suppl 1):**CO-55

**Rationale:** The end-expiratory occlusion (EEXPO) test consists in interrupting mechanical ventilation for 15-s and observing its effects on cardiac output. An increase in cardiac index (CI) ≥ 5% detects preload responsiveness. However, it has been described only with direct measurements of CI, as demonstrated with pulse contour analysis and echocardiography. The perfusion index (PI) is the ratio between the pulsatile and the non-pulsatile portions of the plethysmography signal. It is in part determined by stroke volume and can detect a positive passive leg raising (PLR) test. We hypothesised that the changes in PI could detect a positive EEXPO test and thus preload responsiveness in a totally non-invasive way.

**Patients and methods:** In critically ill patients, we measured PI (Radical 7, Masimo) and CI (PiCCO2, Pulsion Medical Systems) before and during a PLR test, a 15-s EEXPO test and, if decided, before and after volume expansion (VE) (500-mL saline).

**Results:** We included 31 patients. No patient was excluded due to unstable or absent plethysmographic signal. In 19 patients with a positive PLR test (increase in CI ≥ 10%), CI and PI increased during PLR by 17 ± 7% and 49 ± 23%, respectively, and during EEXPO test by 6 ± 2% and 11 ± 8%, respectively. In the 12 patients with a negative PLR test, PI did not significantly change during PLR and EEXPO test (4 ± 4% and 0 ± 1%). Only four patients received VE, causing an increase of CI and PI by 15 ± 3% and 32 ± 47% respectively. The correlation between the PI and CI PLR-induced changes was significant (r = 0.76, p < 0.001). During the PLR test, if PI increased by ≥ 9%, a positive response of CI (≥ 10%) was diagnosed with a sensitivity of 95 (74–100)% and a specificity of 75 (43–95)% (area under the receiver operating characteristics curve (AUROC): 0.94 (0.80–0.99), p < 0.0001 vs. 0.50). The correlation between the PI and CI EEXPO test-induced changes was significant (r = 0.69, p < 0.0001). During the EEXPO test, if PI increased by > 5%, a positive response of CI to the EEXPO test (≥ 5%) was diagnosed with a sensitivity of 87 (60–98)% and a specificity of 94 (70–100)% (AUROC curve: 0.92 (0.77–0.99), p < 0.0001). During the EEXPO test, if PI increased by > 3%, a positive response of CI to the PLR test (≥ 10%) was diagnosed with a sensitivity of 90 (67–99)%, p < 0.0001.

**Conclusion:** An increase in PI ≥ 5% during an EEXPO test accurately detects a positive response of CI to the EEXPO test. An increase in PI during PLR by 9% accurately detects a positive response of the PLR test as we have already shown.

**Compliance with ethics regulations:** Yes.

### CO-56 Mini and micro fluid challenge predicts preload responsiveness in critically ill patients

#### Nello de Vita, Francesco Gavelli, Jean-Louis Teboul, Arthur Pavot, Rui Shi, Xavier Monnet

##### Service de médecine intensive-réanimation, Hôpital de Bicêtre, Hôpitaux universitaires Paris-Saclay, Assistance publique—Hôpitaux de Paris, Inserm UMR S_999, Université Paris-Sud, Le Kremlin-Bicêtre, France

###### **Correspondence:** Nello de Vita (Nellodevita@hotmail.com)

*Ann. Intensive Care* 2020, **10 (Suppl 1):**CO-56

**Rationale:** The administration of a fluid bolus of 500 mL (fluid challenge) has been used to test preload responsiveness in critically ill patients. However, if the patient is not preload responder, the amount of fluid given may be detrimental, contributing to fluid overload. Thus, smaller volumes of fluid bolus have been proposed as an alternative method to test preload responsiveness.

**Patients and methods:** In patients with acute circulatory failure equipped with a PiCCO2 device (Pulsion Medical Systems, Feldkirchen, Germany), we evaluated the ability of the changes in CO induced by the rapid infusion of 50 mL of normal saline over 30 s (micro-fluid challenge) and of 100 mL over 90 s (mini-fluid challenge) to predict preload responsiveness. In preload responders, a fluid bolus of 500 mL of normal saline was administered, according to the judgement of the treating physician.

**Results:** Between May and September 2019, twenty-three consecutive patients were included. Septic shock was the main cause of acute circulatory failure in 21 patients (91%), while hypovolemia was the main cause in two patients (7%). Norepinephrine was administered to all the enrolled patients at the time of inclusion (0.35 [0.13–0.38] mcg/kg/min). Among the included patients, nine (49%) were preload responders, defined according to a PLR-induced increase in CO ≥ 10%, whereas 14 (61%) were non-responders. Micro-fluid challenge-induced changes in CO were significantly different among preload responders and non-responders (9.4[3.5–15.9]% vs. 0.0[0.0–0.7]%, respectively; p = 0.001). Analogously, mini-fluid challenge-induced changes in CO were different between responders and non-responders (11.2[7.2–15.3]% vs. 0.7[0.0–3.5]%, respectively; p = 0.002). The area under the receiver operating characteristic curve for micro and mini-fluid challenge to predict preload responsiveness was 0.968 ± 0.03 (p < 0.0001), with a sensitivity of 100% and specificity of 86% for both tests. The diagnostic threshold of micro-fluid challenge was 1.1%, whereas for mini-fluid challenge it was 4.1%.

**Conclusion:** Both micro and mini-fluid challenge reliably predict preload responsiveness in critically ill patients when their effects on cardiac output are detected by the pulse contour analysis method.

**Compliance with ethics regulations:** Yes.

### CO-57 Impact of diaphragm dysfunction on cardiac function and lung aeration during weaning

#### Emmanuel Rozenberg, Elise Morawiec, Julien Mayaux, Julie Delemazure, Thomas Similowski, Alexandre Demoule, Martin Dres

##### AP-HP. Sorbonne Université, Hôpital Pitié-Salpêtrière, Service de Pneumologie, Médecine intensive—Réanimation (Département “R3S”), F-75013, Paris, France

###### **Correspondence:** Emmanuel Rozenberg (emmanuelrozenberg@gmail.com)

*Ann. Intensive Care* 2020, **10 (Suppl 1):**CO-57

**Rationale:** Diaphragm dysfunction and weaning induced pulmonary edema are two frequent causes of weaning failure but their coexistence and interaction have been poorly investigated. We hypothesized that diaphragm dysfunction may not induce a sufficient decrease in intra-thoracic pressure to increase venous return and generate a weaning induced pulmonary edema. We therefore investigated whether weaning induced pulmonary edema and diaphragm dysfunction are or not associated and evaluated the effect of diaphragm dysfunction on cardiac function and lung aeration during a spontaneous breathing trial (SBT).

**Patients and methods:** Patients with readiness to wean criteria who had failed a first SBT were eligible. Before and after a second SBT, diaphragm function was assessed by measuring the change in tracheal pressure induced by a bilateral phrenic nerve stimulation (Ptr, stim), cardiac function (cardiac output, systolic pulmonary arterial pressure) was evaluated with echocardiography and lung aeration was estimated from the lung ultrasound score (LUS). Plasma protein concentration and hemoglobin were also sampled before and after the SBT. Diaphragm dysfunction was defined by Ptr, stim < − 7 cmH2O and weaning induced pulmonary edema was diagnosed in case of SBT failure associated with 1) increase in plasma protein concentration or hemoglobin > 5% during the spontaneous breathing trial and/or 2) early (E) over late peak diastolic velocity ratio > 0.95 or E over peak diastolic velocity ratio > 8.5.

**Results:** Fifty-three patients were included and 31/53 (58%) failed the SBT. Diaphragm dysfunction was present in 20/24 (83%) of patients with weaning induced pulmonary edema, in 11/22 (50%) patients with SBT success and in 2/7 (29%) patients with other causes of SBT failure (p < 0.01). During the SBT, diaphragm dysfunction induced a significant increase in systolic pulmonary arterial pressure but no change in cardiac output. Patients with diaphragm dysfunction had a higher LUS as compared to their counterparts (10 ± 9 vs. 3 ± 4, respectively, p < 0.01).

**Conclusion:** Diaphragm dysfunction induces a loss of lung recruitment and a significant increase in systolic pulmonary arterial pressure during the SBT. Coexistence of diaphragm dysfunction and weaning induced pulmonary edema is common in case of SBT failure but weaning induced pulmonary edema appears more likely to be involved than diaphragm dysfunction.

**Compliance with ethics regulations:** Yes.

### CO-58 Shear wave elastography, a new tool for diaphragmatic qualitative assessment in critically ill patients

#### Yassir Aarab^1^, Aurelien Flatres^2^, Fanny Garnier^1^, Romaric Larcher^1^, David Chapeau^1^, Kada Klouche^1^, Pascal Etienne^3^, Gilles Subra^4^, Samir Jaber^5^, Nicolas Molinari^6^, Lucie Gamon^6^, Stefan Matecki^2^, Boris Jung^1^

##### ^1^Medical Intensive Care Unit, Montpellier University and Montpellier Teaching Hospital, Montpellier, France; ^2^INSERM U1046, CNRS UMR9214, Université de Montpellier, Montpellier, France; ^3^Laboratoire Charles Coulomb (L2C), University of Montpellier, CNRS, Montpellier, France; ^4^Institut des Biomolécules Max Mousseron (IBMM), UMR5247 CNRS, ENSCM, Université de Montpellier, Montpellier, France; ^5^Saint Eloi Anesthesiology and Critical Care Medicine, Montpellier University and Montpellier Teaching Hospital, Montpellier, France; ^6^Biostatistics Department, Montpellier University and Montpellier Teaching Hospital, Montpellier, France

###### **Correspondence:** Yassir Aarab (y-aarab@f-montpellier.fr)

*Ann. Intensive Care* 2020, **10 (Suppl 1):**CO-58

**Rationale:** Diaphragmatic weakness in the Intensive Care Unit (ICU) is associated with poor outcome. Prolonged mechanical ventilation is associated either with a decrease (atrophy) or an increase (supposed injury) in diaphragmatic thickness, both associated with prolonged weaning. Shear wave elastography is a non-invasive technique that measures diaphragm shear modulus (SM), a surrogate of its mechanical properties. The aim of this study was to describe the diaphragm shear modulus during the ICU stay and to describe its relation with diaphragm thickness.

**Patients and methods:** This prospective and monocentric study included all consecutive critically ill patients. Ultrasound examination of the diaphragm (Aixplorer; SuperSonic-Imagine, Aix-en-Provence, France) was obtained by two investigators every other day until ICU discharge. Demographics, diaphragm thickness, SM and outcomes were collected. A mixed model regression was used to study the relation between SM and diaphragm thickness.

**Results:** We enrolled 102 patients from 2017 December 1st to 2018 June 1st, 88 being invasively mechanically ventilated during the stay. Diaphragm ultrasound evaluation was feasible in 94/102 (96%) patients. The duration of mechanical ventilation during the ICU stay was 7 [4–15] days with 3 [1–5] days spent on controlled mechanical ventilation. SM was 14.3 ± 4.3 kPa and diaphragm end-expiratory thickness was 0.20 ± 0.05 cm upon ICU admission. Increase and decrease ≥ 10% during ICU stay occured in 37 and 49 percent of the patients respectively for diaphragmatic thickness, and in 51 and 41 percent of the patients respectively for diaphragmatic SM. Diaphragm thickness over time was inversely correlated with diaphragm SM and with time spent under mechanical ventilation (Table). Diaphragm SM over time was correlated with time spent under pressure support ventilation or under spontaneous breathing (compared to controlled ventilation) and with time spent under deep sedation. Diaphragm SM was inversely correlated with age, sepsis, exposition to steroids (Table). No association was found between diaphragm SM and outcomes.

**Discussion:** Our results are in line with the myotrauma concept, suggesting alteration in diaphragm mechanical properties associated with increased diaphragm thickness in critically ill patients. We hypothesize that this observation most likely reflects muscle injury and tissue infiltration with edema and inflammatory cells.

**Conclusion:** Shear wave ultrasound elastography suggests that in critically ill patients, the increase in diaphragmatic mass is associated with an alteration in diaphragm mechanical properties as measured by SM.

**Compliance with ethics regulations**: Yes.

**Table 1:**
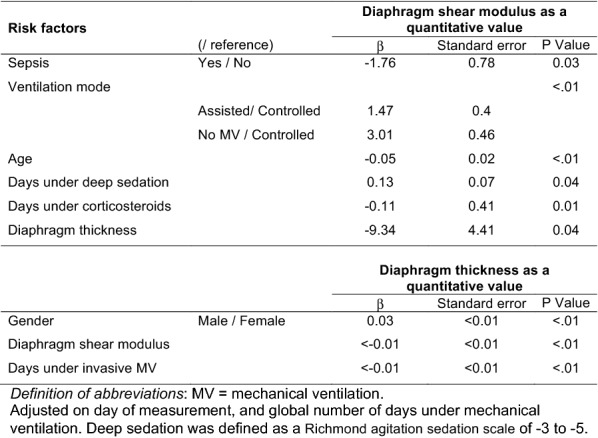
Factors associated with changes in diaphragm shear modulus and thickness during ICU stay. Adjusted multivariate analysis

### CO-59 ICU-acquired weakness but not diaphragm dysfunction strongly impacts long-term survival of critically ill patients

#### Clément Saccheri, Elise Morawiec, Julie Delemazure, Julien Mayaux, Bruno-Pierre Dubé, Thomas Similowski, Alexandre Demoule, Martin Dres

##### Service de Pneumologie, Médecine intensive Réanimation, Hôpital Pitié Salpêtrière, Groupe hospitalier universitaire APHP. Sorbonne Université, Paris, France

###### **Correspondence:** Clément Saccheri (clement.saccheri@gmail.com)

*Ann. Intensive Care* 2020, **10 (Suppl 1):**CO-59

**Rationale:** Diaphragm dysfunction and intensive care unit (ICU) acquired weakness (ICU-AW) are associated with poor outcomes in the ICU but their long term impact on prognosis and health-related quality of life (HRQOL) is poorly established. This study sought to determine whether diaphragm dysfunction is associated with negative long-term outcomes and whether the coexistence of diaphragm dysfunction and ICU-AW has a particular impact on two-year survival and HRQOL.

**Patients and methods:** We used a previous cohort study conducted in our institution to follow up mechanically ventilated patients in whom diaphragm and limb muscle functions were investigated at the time of liberation from mechanical ventilation. Diaphragm dysfunction was defined by tracheal pressure generated by phrenic nerve stimulation < 11 cmH2O and ICU-acquired weakness was defined by Medical Research Council (MRC) score < 48. HRQOL was evaluated with the SF-36 questionnaire.

**Results:** Sixty-nine of the 76 patients enrolled in the original study were included in the survival analysis and 40 were interviewed. Overall two-year survival was 67% (46/69): 62% (28/45) in patients with diaphragm dysfunction, 71% (17/24) in patients without diaphragm dysfunction, 46% (11/24) in patients with ICU-acquired weakness and 76% (34/45) in patients without ICU-acquired weakness. Patients with concomitant diaphragm dysfunction and ICU-acquired weakness had a poorer outcome with a 2-year survival rate of 36% (5/14) compared to patients without diaphragm function and ICU-acquired weakness (79% (11/14) (p < 0.01)). HRQOL was not influenced by the presence of ICU-acquired weakness, diaphragm dysfunction or their coexistence.

**Conclusion:** ICU-acquired weakness but not diaphragm dysfunction has a strong negative impact on two-year survival of critically ill patients. The presence of diaphragm dysfunction appears more likely to be a determinant of early prognosis and does not appear to have a significant impact on long-term survival.

**Compliance with ethics regulations:** Yes.

### CO-60 Role of limb weakness on extubation outcome among patients at high-risk of reintubation in the ICU

#### Arnaud Thille^1^, Florence Boissier^1^, Michel Muller^2^, Albrice Levrat^2^, Gaël Bourdin^3^, Sylvène Rosselli^3^, Jean-Pierre Frat^1^, Rémi Coudroy^1^, Emmanuel Vivier^3^

##### ^1^CHU de Poitiers, Médecine Intensive Réanimation ALIVE research group INSERM CIC 1402, Poitiers, France; ^2^Centre Hospitalier Annecy Genevoix, Réanimation Polyvalente, Metz-Tessy, France; ^3^Centre Hospitalier Saint Joseph Saint Luc, Réanimation Polyvalente, Lyon, France

###### **Correspondence:** Arnaud Thille (aw.thille@gmail.com)

*Ann. Intensive Care* 2020, **10 (Suppl 1):**CO-60

**Rationale:** Whereas ICU-acquired limb weakness may delay extubation in mechanically ventilated patients, its influence on extubation failure is poorly known. Research question: This study aimed at assessing the role of limb weakness on extubation failure in the ICU and the relation between limb weakness and cough strength.

**Patients and methods:** A secondary analysis of two previous prospective studies including patients at high-risk of reintubation after planned extubation, i.e. age greater than 65 years, with underlying cardiac or respiratory disease, or intubated for more than 7 days prior to extubation. Patients intubated less than 24 h and those with a do-not-reintubate order were not included. Limb and cough strength were assessed by physiotherapist just before extubation. Limb weakness was defined as Medical Research Council (MRC) score < 48 points, and severe weakness as MRC score < 36. Cough strength was assessed using a semi-quantitative 5-Likert scale. Extubation failure was defined as reintubation or death within the first 7 days following extubation.

**Results:** Among 344 patients at high-risk, 16% experienced extubation failure (56/344). These patients had greater severity, lower MRC score (41 ± 16 vs. 49 ± 13, p < 0.001) and were more likely to have ineffective cough than the others. Extubation failure rate were 12% (25/214) in patients with no limb weakness vs. 18% (12/65) and 29% (19/65) in those with moderate and severe limb weakness, respectively (p < 0.01). MRC score and cough strength were weakly but significantly correlated (Rho = 0.28, p < 0.001). After multivariate logistic regression analyses, the lower the MRC score the greater the risk of reintubation; and severe limb weakness was independently associated with extubation failure, even after adjustment on cough strength at time of extubation and severity score (SAPS II) at admission.

**Conclusion:** Limb weakness was independently associated with extubation failure in patients at high-risk of reintubation in the ICU.

**Compliance with ethics regulations:** Yes.

### CO-61 Lung bacteriobiota but not mycobiota predicts day 28 mortality among critically ill influenza patients

#### Renaud Prével^1^, Raphaël Enaud^2^, Alexandre Massri^3^, Erwan Begot^1^, Laurent Villeneuve^4^, Fabien Beaufils^2^, Marie-Edith Lafon^5^, Camille Ciccone^5^, Patrick Berger^2^, Alexandre Boyer^1^, Laurence Delhaes^6^, Didier Gruson^1^

##### ^1^CHU Bordeaux, Medical Intensive Care Unit, Bordeaux, France; ^2^Inserm U1045 Centre de Recherche Cardio-thoracique, Bordeaux, France; ^3^Intensive Care Unit, CH Francois Mitterrand, Pau, France; ^4^Bacteriology laboratory, CH Francois Mitterrand, Pau, France; ^5^CHU Bordeaux, Virology laboratory, Bordeaux, France; ^6^CHU Bordeaux, Mycology laboratory, Bordeaux, France

###### **Correspondence:** Renaud Prével (renaud.prevel@hotmail.fr)

*Ann. Intensive Care* 2020, **10 (Suppl 1):**CO-61

**Rationale:** Influenza can lead to severe condition with acute respiratory failure and acute respiratory distress syndrome due to a massive pulmonary inflammatory in response to the viral invasion. Lung bacteriobiota has been described to be associated with pulmonary inflammation in chronic respiratory diseases such as chronic obstructive pulmonary disease or cystic fibrosis. Lung mycobiota has been poorly investigated despite the well-known role for fungi in numerous respiratory diseases. The aim of our study was to assess the prognostic value of lung bacteriobiota and mycobiota among critically ill influenza patients.

**Patients and methods:** We prospectively included influenza patients admitted to ICU. Sputum were stored a -80 °C. Bacterial and fungal DNA were extracted thanks to QIAamp^®^ PowerFecal^®^ Pro DNA Kit. 16S rRNA gene V3-V4 regions and ITS2 regions were amplified by PCR and sequenced on Illumina MiSeq^®^. Taxonomic assignation was obtained by DADA2 pipeline and microbiota analysis were performed according to day-28 mortality by the mean of Phyloseq package on R 3.6.0 software.

**Results:** Thirty-nine patients were admitted to ICU for influenza with 23 sputa available and finally 18 DNA samples available after extraction. Bacteriobiota alpha diversity was significantly lower among non-survivors than survivors when expressed by the mean of Shannon index, Simpson index or Evenness (respectively p = 0.01, p = 0.02, p = 0.01). Area under the curve to predict day-28 mortality was 0.91, 95CI [0.77; 1.00] for Shannon index, 0.89 95CI [0.74; 1.00] for Simpson index and 0.91 95CI [0.77; 1.00] for evenness. β-diversity analysis also demonstrated significant differences between survivors and non-survivors (adjusted permutational multivariate ANOVA, p  =  0.01). Nonsurvivors had a higher abundance of Staphylococcus, Haemophilus, Streptococcus and Moraxella. None of the fungal alpha-diversity index nor beta-diversity were significantively different between survivors and non-survivors. Non-survivors had a higher proportion of Candida albicans and Malassezia but not of Aspergillus.

**Conclusion:** The lung bacteriobiota profile, but not the mycobiota one, of critically ill influenza patients is associated with day-28 mortality and may be used to identify subjects with a poor prognosis at the time of admission.

**Compliance with ethics regulations**: Yes.

### CO-62 Pathway mapping of leukocyte transcriptome in influenza patients reveals distinct pathogenic mechanisms associated with progression to severe infection

#### Yoann Zerbib^1^, Emily Jenkins^2^, Maryam Shojaei^2^, Anthony Mclean^2^, Klaus Schughart^3^, Benjamin Tang^2^

##### ^1^Department of medical Intensive Care, Amiens University Hospital, Amiens, France; ^2^Department of Intensive Care Medicine, Nepean Hospital, Sydney, Australia; ^3^Helmholtz Centre for Infection Research, Braunschweig, Germany

###### **Correspondence:** Yoann Zerbib (zerbib.yoann@chu-amiens.fr)

*Ann. Intensive Care* 2020, **10 (Suppl 1):**CO-62

**Rationale:** Influenza infections produce a spectrum of disease severity, ranging from a mild respiratory illness to respiratory failure and death. The host-response pathways associated with the progression to severe influenza disease are not well understood.

**Patients and methods:** To gain insight into the disease mechanisms associated with progression to severe infection, we analyzed the leukocyte transcriptome in severe and moderate influenza patients and healthy control subjects. Pathway analysis on differentially expressed genes was performed using a topology-based pathway analysis tool that takes into account the interaction between multiple cellular pathways. The pathway profiles between moderate and severe influenza were then compared to delineate the biological mechanisms underpinning the progression from moderate to severe influenza.

**Results:** 107 patients (44 severe and 63 moderate influenza patients) and 52 healthy control subjects were included in the study. Severe influenza was associated with upregulation in several neutrophil-related pathways, including pathways involved in neutrophil differentiation, migration, degranulation and neutrophil extracellular trap (NET) formation. The degree of upregulation in neutrophil-related pathways was significantly higher in severely infected patients compared to moderately infected patients. Severe influenza was also associated with downregulation in immune response pathways, including pathways involved in antigen presentation, CD4+ T-cell co-stimulation, CD8+ T cell and Natural Killer (NK) cells effector functions. Apoptosis pathways were also downregulated in severe influenza patients compared to moderate and healthy controls.

**Conclusion:** These findings showed that there are changes in gene expression profile that may highlight distinct pathogenic mechanisms associated with progression from moderate to severe influenza infection.

**Compliance with ethics regulations**: Yes.

### CO-63 Herpes Simplex Virus and Cytomegalovirus reactivation among severe ARDS patients under veno-venous ECMO

#### Sami Hraiech^1^, Eline Bonnardel^2^, Christophe Guervilly^1^, Jean-Marie Forel^1^, Melanie Adda^1^, Gabriel Parzy^1^, Guilhem Cavaille^1^, Benjamin Coiffard^1^, Laurent Papazian^1^

##### ^1^Service de Médecine Intensive-Réanimation, APHM, Hôpital Nord, Marseille, France; ^2^Magellan Medico-Surgical Center, South Department of Anaesthesia and Critical Care, Bordeaux, France

###### **Correspondence:** Sami Hraiech (sami.hraiech@ap-hm.fr)

*Ann. Intensive Care* 2020, **10 (Suppl 1):**CO-63

**Rationale:** Herpesviridae reactivation among non-immunocompromised critically ill patients is associated with impaired prognosis, especially during acute respiratory distress syndrome (ARDS). However, few is known about Herpes Simplex Virus (HSV) and Cytomegalovirus (CMV) reactivation occurring in patients with severe ARDS under veno-venous ExtraCorporeal Membrane Oxygenation (ECMO). We tried to determine the frequency of herpesviridae reactivation and its impact on patients’prognosis during ECMO for severe ARDS.

**Patients and methods:** We conducted an observational, retrospective study in a medical ICU (ARDS and ECMO referee center) between 2011 and 2017. Patients with a severe ARDS requiring a venovenous ECMO for 2 days or more were included. HSV and/or CMV reactivation occurring after ECMO insertion was screened for these patients. Patients with immunosuppression, antiviral therapy against HSV and/or CMV prior to inclusion, or HSV/CMV reactivation known at the time of ECMO insertion were excluded. HSV reactivation was defined by a positive qualitative throat sample (Virocult^®^) PCR or positive broncho-alveolar lavage (BAL) PCR. CMV reactivation was defined by a positive quantitative blood or BAL PCR.

**Results:** During a five-year period, 123 non-immunocompromised patients with a severe ARDS necessitating a veno-venous ECMO were included. Sixty-seven (54%) experienced HSV and/or CMV reactivation during ECMO course (20 viral co-infection, 40 HSV alone and 7 CMV alone). HSV reactivation occurred earlier than CMV after the beginning of MV (10 (6–15) vs. 19 (13–29) days; p < 0.01) and after ECMO implementation (4 (2–8) vs. 14 (10–20) days; p < 0.01). In univariate analysis, HSV/CMV reactivation was associated with a longer duration of mechanical ventilation (34 (22–52.5) vs. 17.5 (9–28) days; p < 0.01), a longer duration of ECMO (15 (10–22.5) vs. 9 (5–14) days; p < 0.01), and a prolonged ICU (29 (19.5–47.5) vs. 16 (9–30) days; p < 0.01) and hospital stay (44 (29–63.5) vs. 24 (11–43) days; p < 0.01). However, in multivariate analysis, viral reactivation remained associated with prolonged MV only. When comparing patients having CMV (alone or combined with HSV) vs. HSV reactivation alone, CMV positive patients had a longer mechanical ventilation duration and fewer ventilator-free days at day-28 and a longer ICU and hospital length of stay.

**Conclusion:** Herpesviridae reactivation is frequent among patients with sevre ARDS under veno-venous ECMO and is associated with a longer duration of mechanical ventilation. CMV seems to have a proper negative role on pulmonary fiunction as compared to HSV alone. HSV and CMV deserve to be researched in severe ARDS patients under ECMO.

**Compliance with ethics regulations**: Yes.

### CO-64 Comparison of RSV and influenza infection in ICU patients-the CAPTIF study

#### Charlotte Vandueren^1^, Benjamin Zuber^2^, Eve Garrigues^3^, Antoine Gros^4^, Nicolas Epaillard^5^, Guillaume Voiriot^6^, Yacine Tandjaoui-Lambiotte^7^, Jean-Baptiste Lascarrou^8^, Florence Boissier^9^, Virginie Lemiale^10^, Damien Contou^11^, Sami Hraiech^12^, Anne-Pascale Meert^13^, Bertrand Sauneuf^14^, Aline Munting^15^, Sylvie Ricome^16^, Guillaume Geri^3^, Jonathan Messika^17^, Gregoire Muller^18^, Julien Coussement^19^, David Grimaldi^20^

##### ^1^USI, Hôpital Erasme, ULB, Bruxelles, Belgium; ^2^Réanimation Hôpital Foch, Suresnes, France; ^3^Réanimation Hôpital Ambroise Paré, Boulogne, France; ^4^CH Versailles, Le Chesnay, France; ^5^Réanimation Hôpital Saint Antoine, Paris, France; ^6^Réanimation Hôpital Tenon, Paris, France; ^7^Réanimation Hôpital Avicennes, Bobigny, France; ^8^MIR CHU Nantes, Nantes, France; ^9^MIR CHU Poitiers, Poitiers, France; ^10^Réanimation Hôpital Saint Louis, Paris, France; ^11^Réanimation CH Argenteuil, Argenteuil, France; ^12^Réanimation Hôpital Nord-APHM, Marseille, France; ^13^USI, Hôpital BORDET, ULB, Bruxelles, Belgium; ^14^Réanimation CH Public du Cotentin, Cherbourg, France; ^15^CHU UCL Namur, Godinne, Belgium; ^16^Réanimation CH Aulnay, Aulnay S/bois, France; ^17^Réanimation Hôpital Louis Mourier, Colombes, France; ^18^CH Orleans, Orleans, France; ^19^Maladies Infectieuses, Hôpital Erasme, ULB, Bruxelles, Belgium; ^20^USI Hôpital Erasme ULB, Bruxelles, Belgium

###### **Correspondence:** Charlotte Vandueren (chavandueren@yahoo.fr)

*Ann. Intensive Care* 2020, **10 (Suppl 1):**CO-64

**Rationale:** Respiratory syncytial virus (RSV) is a common cause of pediatric bronchiolitis and influenza-like illness in adults. Its involvement in severe infections in adults remains unclear. The CAPTIF study aimed at comparing characteristics and prognosis of ICU patients infected with RSV and influenza, assuming that, based on the limited evidence, the mortality of RSV infection would be lower than the influenza related one.

**Patients and methods:** Multicenter Franco-Belgian retrospective study. Adults admitted to 18 ICUs between 1/Nov/2011 and 30/Apr/2017 with respiratory RSV infection were included and matched 1:1 to influenza patients on center and ICU admission date. Patients’ characteristics, clinical presentation, and outcome were compared between groups using univariate and multivariable analyses.

**Results:** We report here the results for the first 470 cases among 650 included patients. Mean age was 65.5 (16.7) years and SAPS-2 score was 42 (17), not different between groups. Compared to influenza patients, RSV patients more frequently had chronic respiratory failure (61% vs 39%, p < 0.001) or immune suppression (36 vs 26%, p = 0.03). Frequencies of cardiac, renal and hepatic chronic diseases were similar. Almost all patients had respiratory symptoms (> 95%), extrarespiratory symptoms were more frequent in influenza patients (9 vs 15%, = 0.04). RSV patients more frequently had bronchospasm (51 vs 36%, p = 0.001). Clinical presentation such as ARDS (20%), shock (30%) and pulmonary coinfection (32%) were similar, however SOFA score was higher in RSV patients (4.6 (3.4) vs 5.6 (4), p = 0.004). The P/F ratio was around 210 mmHg in both groups, PaCO_2_ was higher in RSV patients (55 vs 47 mmHg, < 0.001). Respiratory assistance at diagnosis tended to differ (p = 0.06), RSV patients receiving more non invasive ventilation (29 vs 19%) and less high flow oxygen therapy (10 vs 14%) but invasive ventilation was required similarly (36 vs 33%). During ICU stay, ARDS was more frequent in RSV patients (21 vs 30%, p = 0.03), accordingly prone position (1.3 vs 4.3%) and ECMO (2.5 vs 9.1%) were more frequently needed. Length of mechanical ventilation (2 days (0–8)) and ICU LOS (5 days (3–12)) were not different. ICU mortality was similar in RSV and influenza patients (18.4% and 21.3%), the multivariate analysis did not find an association between type of virus and mortality.

**Conclusion:** RSV infection is frequent in adult ICU patients. It presents more frequently than influenza as an acute on chronic respiratory failure with bronchospasm. Despite difference in case mix and clinical presentation, VRS severity and burden appear similar to influenza justifying effort to prevent and treat it.

**Compliance with ethics regulations:** Yes.

### CO-65 Epidemiology and 1-year functional outcome of ICU-admitted stroke patients

#### Benoit Brassart, Ahmed El Kalioubie, Saad Nseir

##### Université Lille, Centre Hospitalier Universitaire Lille, Lille, France

###### **Correspondence:** Benoit Brassart (brassart.benoit@gmail.com)

*Intensive Care* 2020, **10 (Suppl 1):**CO-65

**Rationale:** Stroke remains the major cause of severe disability and the second most common cause of death despite advances in prevention and treatments over the last two decades. New techniques of stroke management and ageing of population has led to an increase in ICU-admission of patients with stroke. The aim of our study was to describe the epidemiology and 1-year functional outcome of stroke patients admitted to our intensive care unit and to identify early predictors of poor outcome at admission in this population.

**Patients and methods:** Single Medical ICU Center at the Lille University Teaching Hospital. Retrospective analysis of patients with acute ischemic stroke (AIS) or non-traumatic intracranial hemorrhage (ICH) requiring intensive care admission between January 2008 and December 2015. Patients with subarachnoid hemorrhage were excluded. Admission (demographics, comorbidities, clinical data, cause of ICU admission), ICU (length of stay (LOS), adverse events, tracheotomy) and outcome data (in hospital and one year mortality, one year Modified Rankin Scale (mRS)) were collected from electronic records. Univariate then multivariate analysis were performed to identify risk factors for poor outcome (1 year mRS > 2).

**Results:** In the eight-year period, we admitted 556 patients, of whom 309 (56%) were male, with a mean age of 64 (52.76). 291 (52%) with AIS and 265 (48%) with ICH. Median admission NIHSS was 17. 483 patients (87%) were mechanically ventilated, for a median duration of 3 days (1.11). Patients were primarly admitted for neurologic (82.9%), then respiratory (12.9%) failures. Median ICU and hospital LOS were 4 (1.12) and 11 (2.36) days. Ventilation associated pneumonia occurred in 14.9%, and tracheotomy was performed in 13.5% of cases. A do-not-resuscitate decision was taken for 296 (53%) patients. 306 (55.1%) patients died during hospitalization. mRS > 2 (including death) at hospital discharge and at 1 year follow up involved respectively 91.2% and 84.4%. Only 87 (39.9%) of the 218 patients who were alive at one year were functionally independent. Admission risk factors for poor outcome (1-year mRS > 2), for combined AIS and ICH, were age [OR 1.25 95% IC (1.03, 1.53)], IGSII [OR 1.74 95% IC (1.44, 2.11)], endotracheal intubation [OR 2.31 95% IC (1.08, 4.97)]. In a sub-group analysis, vertebro-basilar AIC (101 (35%) patients), and lobar ICH (174 (66%) patients) were independently associated with a favorable 1-year mRS (respectively OR 0.23 95% IC (0.11, 0.50) and OR 0.24 95% IC (0.10.0.58)).

**Conclusion:** In our retrospective cohort, stroke patients requiring intensive care admission had high mortality rate and survivors were mostly dependent one year after. Further study is needed to better describe this population.

**Compliance with ethics regulations**: Yes.

### CO-66 Association of reason for endotracheal intubation with long-term survival in acute stroke patients requiring mechanical ventilation

#### Etienne de Montmollin^1^, Nicolas Terzi^2^, Claire Dupuis^3^, Maïté Garrouste^4^, Daniel da Silva^5^, Michaël Darmon^6^, Virginie Laurent^7^, Guillaume Thiery^8^, Johanna Oziel^9^, Guillaume Marcotte^10^, Marc Gainnier^11^, Shidasp Siami^12^, Benjamin Sztrymf^13^, Christophe Adrie^14^, Jean Reignier^15^, Romain Sonneville^1^, Jean-François Timsit^1^

##### ^1^Medical and Infectious Diseases Intensive Care Unit, Bichat-Claude Bernard Hospital, Paris, France; ^2^Medical Intensive Care Unit, Grenoble University Hospital, La Tronche, France; ^3^Medical Intensive Care Unit, Gabriel Montpied University Hospital, Clermont-Ferrand, France; ^4^Medical Unit, French British Hospital, Levallois-Perret, France; ^5^Intensive Care Unit, Delafontaine Hospital, Saint-Denis, France; ^6^Medical Intensive Care Unit, Saint-Louis Hospital, Paris, France; ^7^Intensive Care Unit, André Mignot Hospital, Le Chesnay, France; ^8^Intensive care Unit, Hôpital Nord, Saint-Etienne, France; ^9^Intensive Care Unit, Avicenne Hospital, Bobigny, France; ^10^Surgical Intensive Care Unit, Edouard Herriot Hospital, Lyon, France; ^11^Intensive Care Unit, La Timone Hospital, Marseille, France; ^12^Intensive Care Unit, Sud-Essonne Hospital, Etampes, France; ^13^Intensive Care Unit, Antoine Béclère Hospital, Clamart, France; ^14^Physiology department, Cochin Hospital, Paris, France; ^15^Medical Intensive Care Unit, Nantes University Hospital, Nantes, France

###### **Correspondence:** Etienne de Montmollin (edemontmollin@gmail.com)

*Ann. Intensive Care* 2020, **10 (Suppl 1):**CO-66

**Rationale:** Mortality in acute stroke patients requiring mechanical ventilation ranges from 60 to 90% at 1 year. Studies evaluating indicators of outcome in these patients have limitations, including single-center, retrospective designs and no adjustment for withholding/withdrawal of life-sustaining treatments (WLST). Our objective was to identify factors associated with 1-year survival in acute stroke patients requiring mechanical ventilation.

**Patients and methods:** Retrospective analysis of a prospective multicenter database between 1997 and 2016. ICU stroke patients entered in the database and requiring mechanical ventilation within 24 h were included. Were excluded patients with stroke of traumatic origin, subdural hematoma or venous cerebral thrombosis. Factors associated with 1-year survival were identified using a Cox model stratified on inclusion center, adjusted on WFLST occurring during the first 48 h. Data are presented as median [Q1-Q3] or percentages. Cox model results are presented as hazard ratios (HR) and 95% confidence intervals (CI).

**Results:** We identified 419 patients from 14 ICUs, aged 68 [58–76] years and 60% males. On admission, the Glasgow coma score (GCS) was 4 [3–8] and the SAPS 2 score was 58 [47–72]. Types of strokes were ischemic (46%), hemorrhagic (42%) and subarachnoid hemorrhage (SAH) (12%). Ischemic stroke patients received thrombolysis or thromboaspiration in 35/191 (18%) cases, and hemorrhagic stroke/SAH patients received neurosurgery or embolization in 35/228 (15%) cases. Reasons for endotracheal intubation were coma (72%), acute respiratory failure (12%), seizures (8%), cardiac arrest (5%) and elective procedure (3%). Sixty-five (16%) patients received a decision of WFLST in the first 48 h. One-year survival year was 23%. Variables independently associated with 1-year survival were stroke type (ischemic as reference, hemorrhagic HR 0.72 [0.56–0.93], SAH HR 0.61 [0.40–0.91], p = 0.013), specific stroke treatment (HR 1.79 [1.23–2.56], p = 0.002), reason for intubation (elective procedure as reference, seizures HR 0.57 [0.12–2.63], respiratory failure HR 0.24 [0.05–1.02], altered mental status HR 0.21 [0.05–0.88] and cardiac arrest HR 0.08 [0.02–0.37], p < 0.001), GCS score < 8 at admission (HR 0.54 [0.39–0.75], p < 0.001), cardiologic SOFA (per point, HR 0.91 [0.84–0.98], p = 0.017) and decision of WFLST within 48 h (HR 0.41 [0.30–0.56], p < 0.001) (Fig. 1). Inclusion period (1996–2002/2003–2009/2010–2016) or having a stroke unit on site was not associated with 1-year survival.

**Conclusion:** In acute stroke patients requiring mechanical ventilation, the reason for intubation and the opportunity to receive a specific stroke therapy are independently associated with long-term survival. These variables should be integrated in the decision process regarding initiation of MV in acute stroke patients.

**Compliance with ethics regulations:** Yes.

**Fig. 1:**
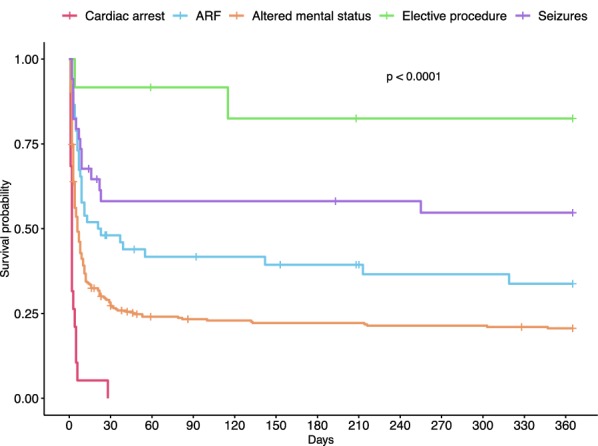
Kaplan–Meier’s survival estimates of patients according to the reason for endotracheal intubation

### CO-67 Association between targeted temperature management and long-term neurological prognosis: insights from two large datasets from France and north-America

#### Jean-Baptiste Lascarrou^1^, Florence Dumas^2^, Wulfran Bougouin^3^, Frankie Beganton^4^, Nadia Aissaoui^5^, Richard Chocron^2^, Nicolas Deye^5^, Daniel Jost^6^, Stéphane Legriel^7^, Lionel Lamhaut^8^, Antoine Vieillard-Baron^9^, Eloi Marijon^10^, Xavier Jouven^4^, Alain Cariou^5^

##### ^1^Médecine Intensive Réanimation, Nantes, France; ^2^Emergency Department, Paris, France; ^3^Medical Surgical Intensive Care Unit, Massy, France; ^4^INSERM, Paris, France; ^5^Médecine Intensive Réanimation, Paris, France; ^6^Brigade Sapeurs Pompiers Paris, Paris, France; ^7^Medical Surgical Intensive Care Unit, Versailles, France; ^8^Medical Intensive Care Unit, Paris, France; ^9^Médecine Intensive Réanimation, Boulogne Billancourt, France; ^10^Cardiology Department, Paris, France

###### **Correspondence:** Jean-Baptiste Lascarrou (jeanbaptiste.lascarrou@chu-nantes.fr)

*Ann. Intensive Care* 2020, **10 (Suppl 1):**CO-67

**Rationale:** International guidelines recommend targeted temperature management (TTM) between 32° and 36 °C for out-of-hospital cardiac arrest (CA) patients. However, it is unknown if this treatment is effective whatever the severity of the insult. We aimed to examine the association between TTM and long-term neurological outcome according to the risk evaluated at time of admission in intensive care unit (ICU) using a dedicated and validated score.

**Patients and methods:** We used data prospectively collected in the Sudden Death Expert Center (SDEC) registry (Great Paris area, France) between May 2011 and December 2017 and in the Resuscitation Outcome Consortium—Continuous Chest Compression (ROC-CCC) between June 2011 and May 2015. We used a modified version of the Cardiac Arrest Hospital Prognosis (mCAHP) score to assess the risk of poor outcome at ICU admission in each of 2 datasets. We finally studied the association between TTM use and long-term neurological prognosis according to mCAHP score at ICU admission divided into tertiles of severity in each of the 2 datasets.

**Results:** There were 2723 patients analyzed in the French dataset and 4202 in the North-American dataset. The mCAHP identified 3 categories: low risk (score < 80 points, 40% of unfavourable outcome), medium risk (80 ≤ score < 105, 80% of unfavourable outcome) and high-risk group (score > 105, 95% of unfavourable outcome). According to the mCAHP score at ICU admission, TTM was associated with a better long-term neurological prognosis in patients with low risk (aOR = 1.62 [1.15–2.30] (p = 0.006) in SDEC registry and aOR = 1.43 [1.02–2.02] (p = 0.038) in ROC-CCC) and high risk (aOR = 1.94 [1.06–3.54] (p = 0.026) in SDEC registry and OR = 2.42 [1.38–4.24] (p = 0.021) in ROC-CCC) of poor outcome, but the association was not significant in patients with a medium risk (aOR = 1.19 [0.76–1.87] (p = 0.424) in SDEC registry and OR = 1.12 [0.86–1.47] (p = 0.360) in ROC-CCC). The results were consistent in both French and north-America datasets.

**Conclusion:** TTM is significantly associated with better long-term neurological prognosis for patients with low risk (mCAHP < 80) and high risk (mCAHP ≥ 105) of poor outcome evaluated at ICU admission according to the CAHP score. Furthers studies are needed to individualize ICU care after cardiac arrest especially for TTM.‬‬‬‬‬‬‬.

**Compliance with ethics regulations**: Yes.

### CO-68 Cost-utility analysis of different treatments for acute ischaemic stroke: a Belgian, micro-costing health technology assessment

#### Evi Mellebeek^1^, Anouk Lesenne^2^, Jef Grieten^1^, Alain Wibail^3^, Ludovic Ernon^3^, Luc Stockx^3^, Elly Vandermeulen^3^, Pascal Vanelderen^4^, Joris Vundelinckx^5^, Sofie van Cauter^3^, Jill Grondelaers^3^, Elke Panis^3^, Dieter Mesotten^4^

##### ^1^KU Leuven/ZOL Genk, Genk, Belgium; ^2^Universiteit Gent/ZOL Genk, Gent, Belgium; ^3^ZOL Genk, Genk, Belgium; ^4^Universiteit Hasselt/ZOL Genk, Genk, Belgium; ^5^ZOL Genk, Genk, Belgium

###### **Correspondence:** Evi Mellebeek (evimellebeek503@hotmail.com)

*Ann. Intensive Care* 2020, **10 (Suppl 1):**CO-68

**Rationale:** Acute ischaemic stroke is associated with a high risk of mortality, morbidity and healthcare-related costs. Over the last decades new treatments, such as thrombolysis and thrombectomy, have been introduced. Because of their further improvement, complications have been decreasing. This also led to extending indications for treatment to patients who were previously not eligible. The impact of this evolution on long-term outcome and cost-effectiveness has mainly been assessed in clinical trials and simulation studies.

**Patients and methods:** This single-centre retrospective study included 564 patients treated for stroke between January 2017 and February 2019. Functional outcome at 90 days was assessed by the modified Rankin Scale (mRS). Cost data were retrieved from individual invoices of 538 patients. Undiscounted total healthcare costs were calculated for the index hospital stay, capped at 90 days. Contribution of 6 cost categories to total costs was analysed. mRS at 90 days was used as a proxy for utilities to define Quality-Adjusted Life Years (QALYs). Multivariate analysis was done for gender, age, Charlson comorbidity index, pre-stroke mRS, stroke severity (NIHSS) and treatment modality (thrombectomy, thrombolysis, thrombectomy + thrombolysis, no intervention). Incremental Cost-Effectiveness Ratios (ICERs), associated to each treatment modality, were calculated.

**Results:** No intervention was done in 328 patients (61.0%). 93 patients (17.3%) required thrombolysis, 64 (11.9%) thrombectomy and 53 (9.9%) the combination. Total costs were mean 15,787 EUR (IQR 3534–18,177). Hospitalisation costs (mean 11,275 EUR, IQR 2066–12,428) represented 71% of total costs, compared with drug costs (341 EUR, IQR 118–330), procedural costs (3283 EUR, IQR 652–2981), honoraria (290 EUR, IQR 111–395), lab (123 EUR, IQR 22–116) and imaging (475 EUR, IQR 334–539). Mean total costs differed between treatment modalities: 12,553 (IQR 3080–12,947) EUR for no intervention, 11,820 (IQR 3265–11,197) EUR for thrombolysis, 26,103 (IQR 12,308–35,893) EUR for thrombectomy and 30,301 (IQR 12,563–35,488) EUR for the combination (p < 0.0001). Drivers for total costs were treatment modality (p < 0.017) and NIHSS-stroke severity (p < 0.0001). Utility scores were 0.65 (IQR 0.42–0.93) for no intervention, 0.70 (IQR 0.42–0.95) for thrombolysis, 0.37 (IQR 0.38–0.62) for thrombectomy and 0.66 (IQR 0.42–0.95) for the combination (p < 0.0001). Thrombolysis was dominant over no intervention, while no intervention dominated thrombectomy. The ICER of the combination was 22.187.650 EUR/QALY compared with no intervention.

**Conclusion:** In a 90-day time horizon, thrombectomy or its combination with thrombolysis was systematically dominated or associated with extremely high ICERs. However, their cost-effectiveness needs to be evaluated in long-term health-economic models with a time horizon of at least 5 years.

**Compliance with ethics regulations**: Yes.

## Flash communications

### F-001 A Systematic Echography During Intubation Procedure to Predict Cardiovascular Collapse (EPIC)

#### Vanessa Jean-Michel^1^, Gwenael Prat^2^, Pierre Bailly^2^, James Benis^3^, Montaine Lefevre^4^, Pierre-Yves Egreteau^4^, Christophe Giacardi^5^, Cécile Aubron^2^, Erwan L’her^2^

##### ^1^CH de Tourcoing, Tourcoing, France; ^2^CHRU de La Cavale Blanche, Brest, France; ^3^CH de Quimper, Quimper, France; ^4^CH de Morlaix, Morlaix, France; ^5^HIA Clermont Tonnerre, Brest, France

###### **Correspondence:** Vanessa Jean-Michel (vanessa.jeanmichel@gmail.com)

*Ann. Intensive Care* 2020, **10 (Suppl 1):**F-001

**Rationale:** Emergency endotracheal intubation (ETI) in the Intensive Care Unit (ICU) often concerns hypoxemic patients with hemodynamic instability. A cardiovascular collapse (CVC) after ETI is a life-threatening complication. 2018 French guidelines suggested systematic fluid loading prior to ETI. Our study aimed to predict CVC after ETI, while using echocardiography, and to evaluate the impact of fluid loading.

**Patients and methods:** A prospective study of 70 consecutive intubations was performed from June 2017 to November 2018 in three ICUs. Patients were selected if mean blood pressure measurements ≥ 60 mmHg before ETI. CVC was defined as mean blood pressure < 60 mmHg within 15 min following ETI. Four echocardiographic examinations were performed: 1–30 min before and 2–30 min after ETI (or when a CVC occurred); 3—after passive leg raising; 4–3 h following ETI. Patients were classified as fluid responders when the left ventricular outflow tract velocity–time integral increased by at least 10% compared with baseline.

**Results:** 269 echocardiographic examinations were performed. CVC occurred in 32/70 procedures (46%). In CVC group, mean dose of Diprivan, used for fast sequence induction, was higher (2.8 ± 1 mg/kg vs 1.3 ± 0.7 mg/kg, p = 0.02). In the CVC group, fluid responsiveness was considered in 44% patients and left ventricular (LV) systolic dysfunction 13%. LV diastolic dysfunction did not concern any patient in the CVC group. Systolic blood pressure (SBP) < 120 mmHg was the sole independent risk factor for CVC occurrence in multivariate analysis: OR 5.9 CI 95% 1.7–21.2, p = 0.02. Fluid responsiveness independent risk factors for CVC patients was SBP < 120 mmHg (OR 8.7, CI 95% 1.1–65.1, p = 0.01) and tidal volume > 7 ml/kg IBW (OR 7.8 ICI 5% 1.02–60.3, p = 0.048).

**Conclusion:** CVC frequently occurs following ETI. Fluid loading does not seem to be mandatory for all patients to prevent CVC during the ETI procedure.

**Compliance with ethics regulations**: Yes.

### F-002 Heart rate variability as an indicator of outcome in the ICU? A prospective cohort

#### Laetitia Bodenes, Victoire Pateau, Julien Dolou, Quang-Thang N’guyen, Erwan L’her

##### Service de Medecine Intensive et Réanimation, CHRU de Brest, Brest, France

###### **Correspondence:** Laetitia Bodenes (Laetitia.bodenes@chu-brest.fr)

*Ann. Intensive Care* 2020, **10 (Suppl 1):**F-002

**Rationale:** The autonomic nervous system is highly adaptable and allows the organism to maintain its balance when experiencing stress. Heart rate variability (HRV) is a mean to evaluate cardiac effects of autonomic nervous system activity and a relation between HRV and outcome has been proposed in various types of patients. We attempted to evaluate the best determinants of such variation in survival prediction using a physiological data-warehousing program (ReaSTOC ClinicalTrials identifier NCT 02893462).

**Patients and methods:** Physiological tracings were recorded at 125 Hz from the standard monitoring system (Intelliview Philips MP70) using the Synapse Software (LTSI INSERM UMR 1099), for a 2 h period, during the 24 h following ICU admission. All measurements were recorded while patients were laying in bed, with the head at 30° and without any medical intervention. Physiological data were associated with metadata collection by a dedicated research assistant. HRV was derived using Kubios HRV, in either temporal ( (SDNN), (RMSSD) and triangular index (TI)), frequency ( (LF), (HF)), non-linear domains (Poincaré plotting) and entropy.

**Results:** 540 consecutive patients were recorded between May 2014 and April 2019. A lower LF/HF (< 0.9) and SD2/SD1 (< 1.3) ratios on admission were associated with a higher ICU mortality. Multivariate analysis enabled to develop a mortality predictive model (BICUS) associating SpO2/FiO2 and HRV parameters (LF/HF and Shannon entropy) with an AUC = 0.73 (p < 0.0001) for a BICUS value > 1 (Fig. 1).

**Conclusion:** HRV measured on admission enables to predict prognosis in the ICU, independently of the admission diagnosis, treatment and MV requirements. BICUS may help predict prognosis on a real time basis, using parameters derived from standard routine monitoring.

**Compliance with ethics regulations:** Yes.

**Fig. 1:**
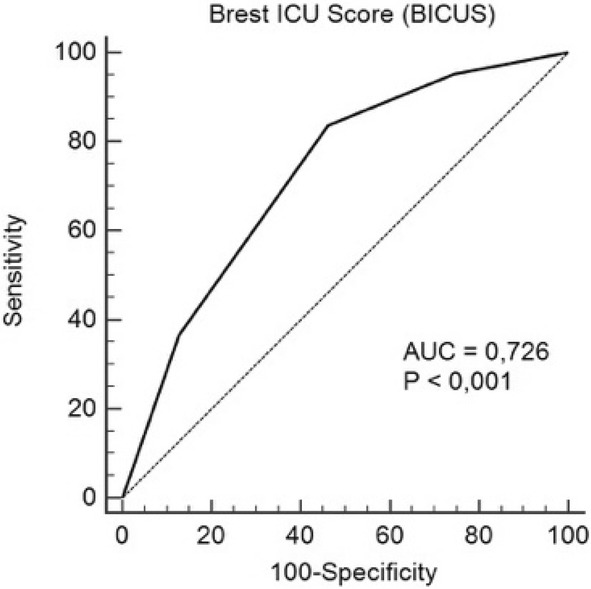
ROC curve for Brest ICU Score (BICUS)

### F-003 A mouse model of ischemic stroke in impaired glucose tolerance condition induced by chronical exposure of High Fat Diet

#### Coline Grisotto, David Couret

##### Service neuroréanimation, CHU Sud Reunion, Saint-Pierre, France

###### **Correspondence:** Coline Grisotto (grisottocoline@yahoo.fr)

*Ann. Intensive Care* 2020, **10 (Suppl 1):**F-003

**Rationale:** Stroke, in the context of type 2 diabetes (T2D) is associated with a worse outcome than in non-diabetic conditions, reflected by an increased ischemic volume and more intracerebral hemorrhage. An unbalanced diet is one of major risk for developing T2D. We aimed at creating a reproducible mouse model of stroke in impaired glucose tolerance condition induced by high fat diet.

**Patients and methods:** Adult C57BL6mice (28 male and 28 female) were fed for 2 months with either High Fat Diet (HFD, 43% lipids, 21% proteins, 35% carbohydrates) or a normal diet (ND, 8.4% lipids, 19.3% proteins, 72.4% carbohydrates). We used a model of Middle Cerebral Artery Occlusion (MCAO) by a monofilament for 90 min. Oral Glucose Tolerance Test and Insulin Tolerance Test were used for evaluating the pre-diabetic state. Mice were euthanized 20 h after reperfusion. Systemic inflammation, cerebral infarct volume and hemorrhagic transformation were determined.

**Results:** HFD was associated with an increased glycaemia following the Oral Glucose Tolerance Test. Plasma leptinlevels in stroke conditions were significantly higher in HFD vs ND group. The HFD group presented a significant increase of infarct volume (HFD: 51.86 ± 4.46 mm^3^ vs ND: 33.23 ± 4.29 mm^3^ p = 0.016) and hemorrhagic transformation (HFD: 2.67 ± 0.66 vs ND: 0.73 ± 0.28 p = 0.012) (Fig. 1) compared to ND group.

**Discussion:** In humans, one of the mechanisms leading to insulin resistance is low-grade inflammation. HFD increases gut permeability, which leads microbiota dysbiosis, thereby promoting metabolic endotoxaemia and a low-grade inflammation state. Experimental mouse models available for diabetes studies use leptin receptor deficient mice which develop T2D or destruction of pancreatic beta cells by streptozotocine injection (T1D). Studies using diet-induced insulin resistance models generally feed the mice for 12 weeks or more. However, metabolic disorders could appear earlier such as increase inflammatory markers. In our model, a short exposition to HFD (8 weeks) leads to an increase of the pro-inflammatory markers as plasma leptin and a more severe stroke status (infarct and hemorrhagic transformation).

**Conclusion:** Two months of HFD in adult mice altered hyperglycemia control. This metabolic disorder was associated with significantly higher leptin production, increased infarct volume and hemorrhagic complications than in normal-fed mice. This new model is particularly relevant to study stroke under pre-diabetic conditions induced by HFD.

**Compliance with ethics regulations:** Yes.

**Fig. 1:**
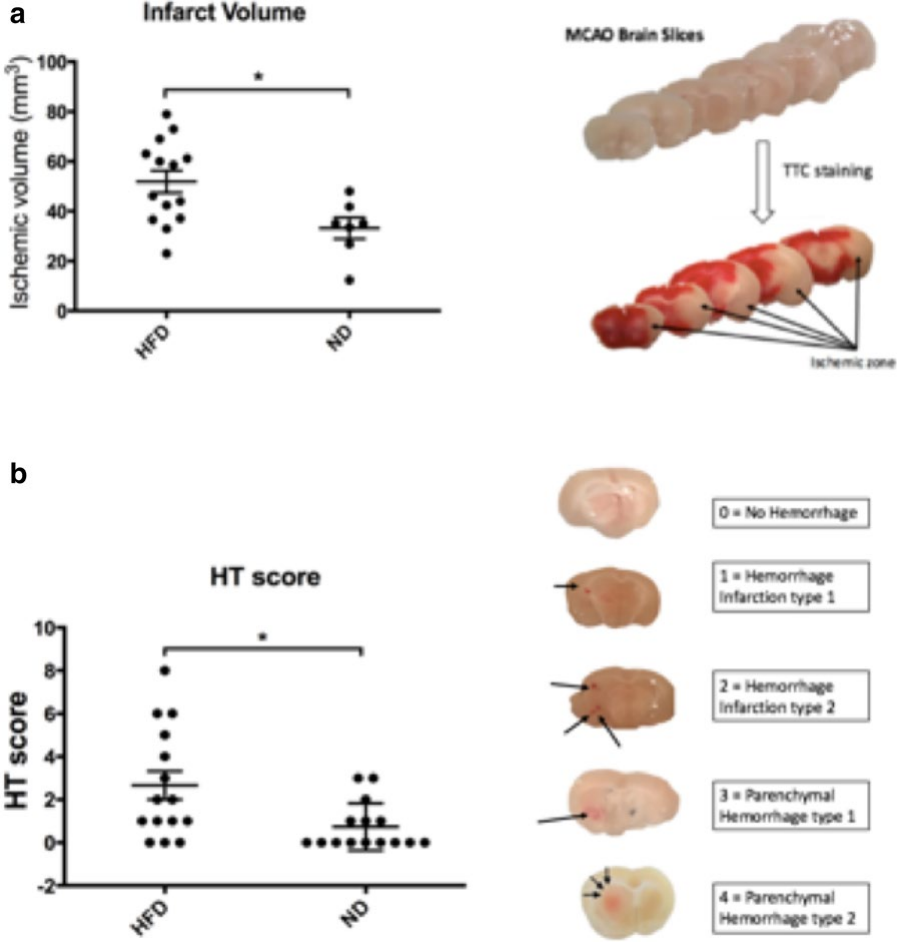
Eight weeks of HFD increase ischemic volume and hemorrhagic transformation. (A)-Infarct volume (V) 24 h after reperfusion, all value are mean ± SEM, HFD: V = 51.66 ± 4.46 mm^3^, n = 14, ND: V = 33.23 ± 4.26 mm^3^, n = 7, *p = 0.016 (B)—Hemorrhage Transformation (HT) score 24 h after MCAO. All value are mean ± SEM HFD: HT score = 2.67 ± 0.66, n = 15, ND: HT score = 0.73 +/+ 0.28, n = 15 *p = 0.012

### F-004 Does metformin act as a protective drug after a massive ischemia reperfusion induced by cardiac arrest? A survival study

#### Barthélémy Vallee^1^, Amélie Rolle^1^, Romain Rozier^2^, Frédéric Martino^1^, Jean-Ehrland Ricci^2^, Bertrand Pons^1^, Michel Carles^1^

##### ^1^CHU Guadeloupe, Les Abymes, France; ^2^Equipe 3-INSERM U1065-Université Nice Sophia Antipolis (UNS), Nice, France

###### **Correspondence:** Barthélémy Vallee (barthelemy.vallee@chu-guadeloupe.fr)

*Ann. Intensive Care* 2020, **10 (Suppl 1):**F-004

**Rationale:** Cardiac arrest (CA), as massive ischemia reperfusion (IR), is an universal health issue. Medication taken at the time of the CA could have prognosis consequences. No medication has proven its benefit on CA prognosis. Pharmacological pre- or postconditioning aims to reduce IR injury but with disappointing results. Metformin (MET) is a worldwide-prescribed antidiabetic drug, and several clinical reports plead for a potential protective effect in various settings of sterile and non sterile inflammation, including IR. Our hypothesis is that MET act as a preconditioning drug against CA-induced IR.

**Patients and methods:** Retrospective single academic medical center survival study (French West Indies) on resuscitated CA in ICU (institutional ethical committee approval). Data were extracted from medical charts, PMSI, and laboratory DBSYNERGY™ software. Anonymized data were entered on a Excel™ and transferred to IBM^®^—SPSS^®^ software (v 24.0.0.0) for analysis. Univariate study (Chi-2, Fisher exact tests, Student-t test, Mann–Whitney U-test if required) was followed by a multivariate model (Odd ratio OR and 95% IC: Kaplan–Meier estimator and non parametric Logrank test-Mantel Cox model). Assuming an overall in-hospital mortality for CA in ICU of 80% with an expected mortality decrease of 15% by MET, the number of patients to be included is 510.

**Results:** The inclusion period was 2012 to 2018, with 555 included patients (202 diabetic patients among whom 62 took MET). The D28 mortality was 67% in MET+ patients (n = 62) versus 82% in NoMET patients (n = 493), p < 0.01. Comparing alive (n = 115) versus deceased (n = 440) at D28 in univariate then multivariate analysis, asystole on the first EKG, number of iterative cardiac arrest, SOFA, No-Flow, lactate, Low-flow and SAPSII appear as independent criteria associated with D28 mortality. Conversely, MET intake showed up as a protective criterion (OR 0.477, CI95 0.237–0.957). The survival curve, including strata of Low-Flow duration at the cut-off 20 min, is reported on the Fig. 1. Among diabetic patients (n = 202), the mortality of patients in the MET+ (n = 62) was 67% versus 80% in the NoMET (n = 140), p = 0.04.

**Conclusion:** In diabetic patients suffering of massive IR related to resuscitated CA, a current treatment by MET is associated with a better survival. These results support a protective effect of MET and are important to initiate prospective evaluations, because of millions diabetic people around the world and the potential benefit of MET. The potential benefit in non diabetic patients and in sterile as well as non sterile inflammation should be addressed.

**Compliance with ethics regulations**: Yes.

**Fig. 1:**
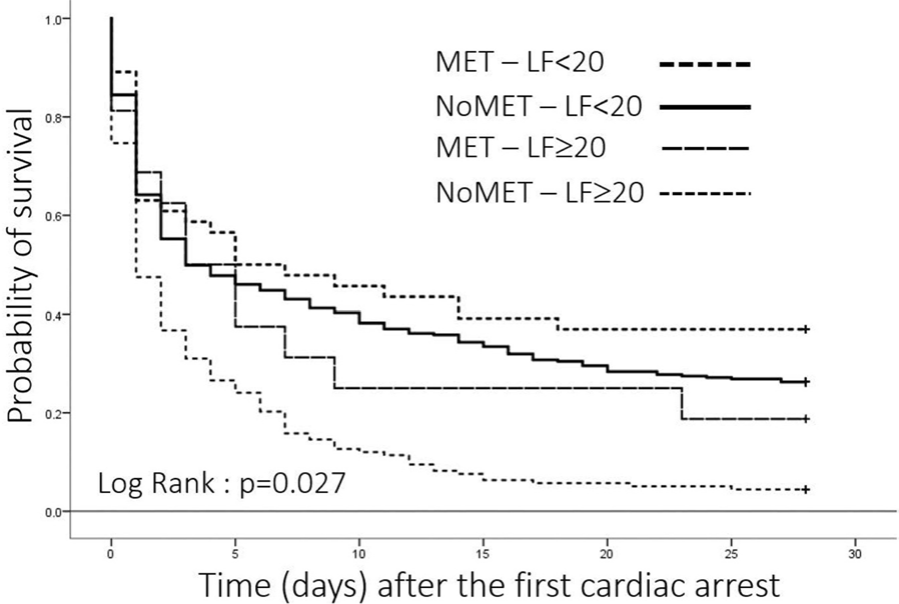
Survival analysis of the 555 having or not an ongoing treatment by MET at the time of the first CA, groups MET-LF < 20, MET-LF > 20, NoMET-LF < 20, NoMET-LF > 20)

### F-005 Ischemic and hemorrhagic complications of patients on venoarterial-extracorporeal membrane oxygenation for cardiogenic shock post myocardial infarction

#### Paul Masi, Maxence Le Guyader, Charles-Edouard Luyt, Guillaume Franchineau, Loïc Le Guennec, Simon Bourcier, Nicolas Brechot, Matthieu Schmidt, Alain Combes, Guillaume Hekimian

##### Réanimation Médicale Pitié Salpétrière, Paris, France

###### **Correspondence:** Paul Masi (paul.masi@aphp.fr)

*Ann. Intensive Care* 2020, **10 (Suppl 1):**F-005

**Rationale:** Short-term ischemic and hemorrhagic complications of patients on venoarterial-extracorporeal membrane (VA-ECMO) for cardiogenic shock post myocardial infarction (MI) have not yet been reported. Our objective was to describe frequencies, outcomes and risk factors for ischemic and hemorrhagic complications.

**Patients and methods:** From 2015 to 2019, we conducted a retrospective observational study in a single tertiary care center on patients who developed a cardiogenic shock post MI and required VA-ECMO.

**Results:** During this period, 177 patients were implanted with VA-ECMO for cardiogenic shock post MI. Mean age was 56 years and 78% were male. MI was complicated by cardiac arrest in 61%. Culprit lesion was respectively the left main coronary artery for 23 patients (14.6%), left anterior descending artery for 82 patients (46.3%), circumflex artery for 14 patients (8.9%) and right coronary artery for 36 patients (22.9%). Intraaortic balloon pump or Impella were implanted respectively for 89 patients (50.9%) and eight patients (4.6%). 108 (61.0%) patients died in intensive care, 76 (43.4%) patients had a hemorrhagic complication, 46 patients (26%) had a severe hemorrhagic complication defined by death or/and massive transfusion or/and hemostatic procedure (surgery or endoscopy), 87 (49.1%) patients had thrombopenia < 100 G/L, 50 (28.2%) patients had thrombopenia < 50 G/L, 53 patients (30.1%) and 43 patients (24.6%) required antiaggregation and anticoagulation withdrawal. Nine (5.6%) patients died secondary to bleeding. VA-ECMO membranes thrombosis occurred in six (3.5%) patients. A coronary angiography check was performed for 27 (15.6%) patients, 8 (4.6%) patients had stent thrombosis.

**Conclusion:** Ischemic and hemorrhagic complications are frequent in patients on VA-ECMO for cardiogenic shock post MI. The main findings of our study were an high rate of severe bleeding complications and thrombopenia requiring antiaggregation withdrawal associated with a very high prevalence of stent thrombosis.

**Compliance with ethics regulations:** Yes.

### F-006 The endoplasmic reticulum stress expression under cardiopulmonary bypass: the relationship with organ failure

#### Zoé Demailly^1^, Thomas Clavier^1^, Vincent Richard^2^, Fabienne Tamion^1^

##### ^1^Department of medical critical care-Rouen university hospital, Rouen, France; ^2^UMR inserm 1096 Endothelium, Valvulopathy & Heart Failure, Rouen, France

###### **Correspondence:** Zoé Demailly (lulu.demailly@gmail.com)

*Ann. Intensive Care* 2020, **10 (Suppl 1):**F-006

**Rationale:** During systemic inflammation, the accumulation of misfolded proteins in the endoplasmic reticulum (ER) induces ER stress (ERS). In animal models, the inhibition of ERS reduces inflammatory response and organ failure. Cardiopulmonary bypass (CPB) induces a significant systemic inflammatory response but ERS expression has never been described in cardiac surgery patients. Our objective was to describe the variations of the Glucose Related Protein of 78 kDa (GRP78), the final effector of the ERS, during CPB.

**Patients and methods:** We conducted a prospective monocenter study including patients undergoing cardiac surgery with CPB. Two samples (PAXGene^®^ tube + EDTA tube) were taken at three times: before CPB, 2 h after the end of CPB (H2-CPB) and 24 h after (H24-CPB). After RNA isolation and reverse transcription, we performed a quantitative polymerase chain reaction to evaluate the expression of gene encoding for GRP78 and determined the plasma level of GRP78 using Enzyme-Linked Immunosorbent Assay. Our main objective was to study the variation of GRP78 between pre-CPB and H2-CPB samples. Our secondary objectives were to evaluate the association of ERS with morbi-mortality: organ failure at 24 h (catecholamines and/or invasive ventilation and/or acute renal failure), troponinemia and PaO2/FiO2 ratio (lung damage control).

**Results:** We included 46 patients with a median age of 70[63; 75] years and a median CPB duration of 117[92; 139] min. We showed an increase in GRP78 gene expression (p < 0.0001) and a decrease in its plasma level at H2-CPB (4540[2519; 7575] ng/mL vs. 1902[939; 3133] ng/mL; p < 0.0001). H24-CPB GRP78 plasma levels were lower than baseline in patients with persistent organ failure at 24 h but returned to baseline in patients without persistent organ failure (− 37[− 61;− 18] % vs. 0.33[− 29; 43] %; p < 0.01; Fig. 1). We found an inverse correlation between GRP78 plasma level and troponinemia at H2 (r = − 0.31; 95% CI[− 0.56; − 0.10]; p = 0.037) and a correlation between the PaO2/FiO2 ratio and GRP78 plasma level at H2 (r = 0.37; 95% CI[0.10; 0.59]; p = 0.0064).

**Conclusion:** We showed a significant relationship between the variation in plasma concentration of GRP78 and post-operative organ failure after CPB. Further studies are needed to better understand the molecular mechanisms of ERS in acute inflammatory organ failure in humans.

**Compliance with ethics regulations**: Yes.

**Fig. 1:**
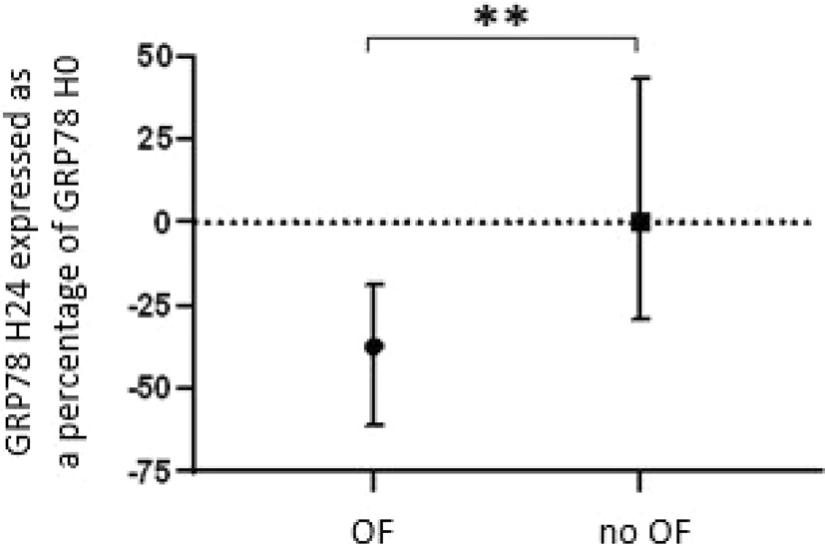
GRP78 concentration variations from baseline in patients with or without persistent organ failure at 24 h. GRP78: Glucose Related Protein 78 kDa; OF: organ failure; **p < 0.01

### F-007 Acute mesenteric ischemia in patients under mechanical support by veno-arterial ECMO

#### Marie Renaudier^1^, Quentin De Roux^1^, Wulfran Bougouin^2^, Johanna Boccara^1^, Baptiste Dubost^1^, Antonio Fiore^1^, Aurélien Amiot^1^, Olivier Langeron^1^, Nicolas Mongardon^1^

##### ^1^CHU Henri Mondor, Créteil, France; ^2^Hôpital Privé Jacques Cartier, Massy, France

###### **Correspondence:** Marie Renaudier (Mrenaudier@aol.com)

*Ann. Intensive Care* 2020, **10 (Suppl 1):**F-007

**Rationale:** Acute mesenteric ischemia is a dramatic complication in critically ill patients. However, acute mesenteric ischemia has never been evaluated in patients under veno-arterial ExtraCorporeal Membrane Oxygenation (VA-ECMO). This study was designed to determine the incidence of acute mesenteric ischemia in this population and to evaluate its risk factors, therapeutic modalities and outcome.

**Patients and methods:** In a retrospective monocentric study (01/2013–01/2017) conducted in cardio-vascular surgical intensive care unit (ICU) in Henri Mondor teaching hospital, all consecutive adult patients who underwent peripheral VA-ECMO were included, with exclusion of those dying in the first 24 h. Diagnosis of acute mesenteric ischemia was performed using digestive endoscopy, abdominal CT-scan or fist-line laparotomy. Significative results in the univariate analysis were analyzed in a multivariate analysis using logistic regression.

**Results:** 150 VA-ECMO were implanted. Median age was 58 (48–69) years and median SAPS II 54 (38–70). VA-ECMO was implanted after a cardiotomy in 43% of the cases and for a medical reason in 57% of the cases including 26% of refractory cardiac arrest. Patients characteristics are reported in the table. Acute mesenteric ischemia was suspected in 38 patients, with a delay of 4 (2–7) days after ECMO implantation. Digestive endoscopy was performed in 33 patients, CT-scan in five patients and first-line laparotomy in three patients. Acute mesenteric ischemia was confirmed in 14 patients, i.e. an incidence of 9%. Laparotomy was performed in six of the 14 patients, two having a stage I colitis ischemitis with stable conditions and 6 being considered too severe to undergo futile surgery. Overall mortality was 56%. All the patients with acute mesenteric ischemia died in the ICU. Independent risk factors of developing acute mesenteric ischemia were renal replacement therapy (OR 4.5 (95% IC 1.3–15.7, p = 0.02)) and onset of a second shock state within the first 5 days of ICU stay (OR 7.8 (95% IC 1.5–41.3, p = 0.02)). Conversely, early enteral nutrition was negatively associated with acute mesenteric ischemia (OR 0.15 (95% IC 0.03–0.69), p 0.02).

**Conclusion:** Acute mesenteric ischemia is a relatively frequent condition among patients under VA-ECMO for cardiogenic shock. Its extremely poor prognosis requires low threshold of suspicion.

**Compliance with ethics regulations**: Yes.

**Table 1:** Patients characteristics and initial management

Characteristics	All ECMO (n = 150)	AMI (n = 14)	No AMI (n = 136)	p
Age (years)	58 (48–69)	57 (47–70)	58 (48–68)	0.82
VA-ECMO for medical reason	85 (57)	6 (43)	79 (58)	0.21
Refractory cardiac arrest	39 (26)	5 (36)	34 (25)	0.28
Intra-aortic balloon pump	28 (19)	3 (21)	25 (18)	0.73
Delay between admission and VA-ECMO (days)	0 (0–1)	0 (0–2)	0 (0–1)	0.27
SAPS II	54 (38–70)	56 (45–73)	53 (38–70)	0.47
Lactate level at day 0 (mmol/L)	5.2 (3–9.1)	7.8 (2.6–10.2)	5.1 (3–9.1)	0.49
Vasoactive-Inotropic Score	70 (34–139)	136 (51–238)	70 (34–129)	0.05
Renal replacement therapy in the first 5 days	47 (32)	9 (64)	38 (28)	0.01
Highest norepinephrine dose in the first 5 days (µg/kg/min)	1.0 (0.5–2.0)	2.3 (1.4–3.0)	1.0 (0.5–1.9)	0.01
Second shock in the first 5 days	76 (51)	12 (86)	64 (47)	0.009
Enteral nutrition in the first 5 days	128 (88)	10 (71)	118 (90)	0.06
ICU mortality	84 (56)	14 (100)	70 (51)	< 0.001

### F-008 Comparison of pleural and esophageal pressure in supine and prone position in a porcine model of acute respiratory distress syndrome

#### Nicolas Terzi^1^, Sam Bayat^1^, Norbert Noury^2^, Jean-Pierre Giliberto^3^, Sylvie Roulet^3^, Emanuele Turbil^4^, Walide Habre^3^, Claude Guérin^2^

##### ^1^CHUGA, Grenoble, France; ^2^Université de Lyon, Lyon, France; ^3^Université de Genève, Genève, Switzerland; ^4^Université de Sassarie, Sassarie, Italy

###### **Correspondence:** Nicolas Terzi (nterzi@chu-grenoble.fr)

*Ann. Intensive Care* 2020, **10 (Suppl 1):**F-008

**Rationale:** Measurement of esophageal pressure (Pes) is the single method currently available to estimate pleural pressure (Ppl) in ICU patients (1). It allows the computation of trans-pulmonary pressure (2) and can be used to set positive end-expiratory pressure (PEEP) (3.4). Prone position (PP) can reduce mortality in patients with acute respiratory distress syndrome (ARDS), but PEEP selection in PP is controversial. In human ARDS end-expiratory Pes at zero flow (PEEPt,es) was not different between supine (SP) and PP at same PEEP (5). As no study measured Ppl in SP and PP in ARDS we aimed at comparing PEEPt,es and end-expiratory Ppl at zero flow (PEEPt,Ppl) in this condition. Our hypothesis was that PEEPt,es was close to dorsal PEEPt,Ppl (PEEPt,Ppldorsal) in SP and to ventral PEEPt,Ppl (PEEPt,Pplventral) in PP.

**Patients and methods:** In eight female pigs of 40 kgs intubated, sedated, paralyzed and mechanically ventilated, ARDS was induced by repeated saline lavage until PaO2/FIO2 < 100 mmHg under FIO2 1 and PEEP 5cmH2O. Pes was measured by Nutrivent catheter. Ppl was measured by custom-made pouch sensors inserted surgically into the right anterior and posterior sixth intercostal space. Ppl sensors were filled with air. After ARDS induction animals were randomly assigned to SP or PP. In each position, a recruitment manoeuver was performed and PEEP decreased from 20 to 5 cmH2O by steps of 5cmH2O lasting 10 min each, then the animals were crossed over into the alternate position where the same procedure was done. At the end of each step nonstressed volume and correct position (Baydur maneuver) were determined for Pes and Ppl sensors, then a 3-s end-expiratory occlusion was performed and Pes and Ppl recorded. Linear mixed model was used to compare the value of Pes and Ppl at each PEEP and position.

**Results:** Box-and-whisker plots of Pes and Ppl in SP and PP are shown in Fig. 1. There is marked dorsal-to-ventral gradient in Ppl at each PEEP in SP, which is reverted in PP at PEEP 10 and 15 only. There was no interaction between pressures and PEEP or position. With increasing PEEP Pes increased significantly from PEEP 5 in SP and PP. PEEPt,PplVentral was significantly lower than PEEPt,es in SP but not in PP.

**Conclusion:** PEEPtes follows PEEPt,Ppldorsal in SP and PEEPt,Pplventral in PP in this ARDS porcine model. References: (1) Akioumaniaki et al. AJRCCM 2014; (2) Yoshida et al. AJRCCM 2018; (3) Talmor et al. NEJM 2008; (4) Beitler et al. JAMA 2019; (5) Mezidi et al. AOIC 2018.

**Compliance with ethics regulations**: Yes.

**Fig. 1:**
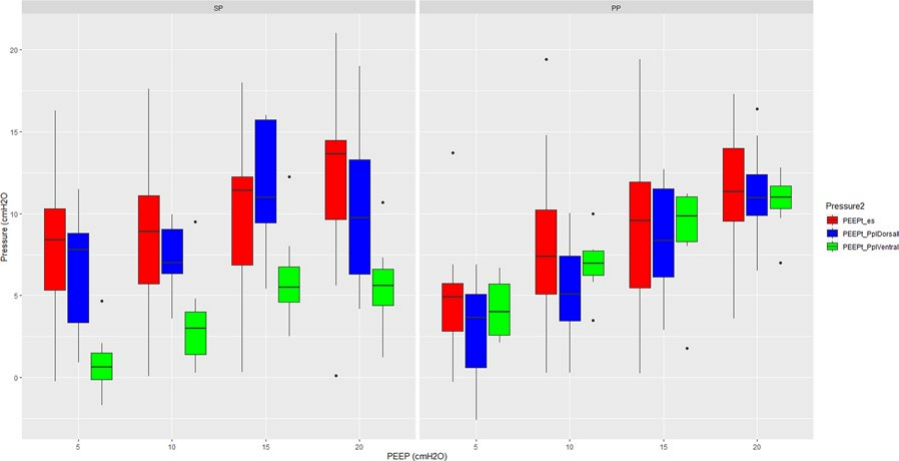
Box-and-whisker plots of esophageal pressure and pleural pressure in supine position and in prone position

### F-009 A systematic bench assessment of automatic tube compensation provided by intensive care unit ventilators

#### Nicolas Terzi^1^, Louis-Marie Galerneau^1^, Zakaria Riad^2^, Emanuele Turbil^3^, Carole Schwebel^1^, Claude Guérin^2^, Bruno Louis^4^

##### ^1^Médecine Intensive Réanimation, Grenoble, France; ^2^Médecine Intensive Réanimation, Lyon, France; ^3^Médecine Intensive Réanimation, Sassari, Italy; ^4^INSERM UMR 955 Eq13, Créteil, France

###### **Correspondence:** Nicolas Terzi (nterzi@chu-grenoble.fr)

*Ann. Intensive Care* 2020, **10 (Suppl 1):**F-009

**Rationale:** Automatic tube compensation (ATC) is an option available in intensive care unit (ICU) ventilators to compensate for endotracheal tube (ETT) resistance. To achieve this ICU ventilator delivers a certain amount of pressure/flow that compensates for the resistive pressure drop across ETT. It requires notifying size of ETT and percent of compensation. We reasoned that if ATC works properly tidal volume (VT) should be the same without ATC and no ETT as with ATC and ETT. We tested the performance of ICU ventilators on a bench, expecting furthermore differences between them.

**Patients and methods:** Seven ICU ventilators (Evita XL and V500 infinity (Draeger), C6 and S1 (Nihon-Khoden), Elisa 800 (Lowenstein), 980 (Medtronic), Carescape 860 (GE)) were set in pressure support 0 cmH2O, PEEP 4 cmH2O, FIO2 21% and equipped with the same double limb ventilator circuit (Intersurgical) without any humidification device. ASL 5000 bench model was set with 3 inspiratory/expiratory resistance (R) and compliance (C) combinations: R13/12-C54, R12/14-C39 and R22/18-C59 mimicking normal, ARDS and COPD conditions, respectively (1). Inspiratory effort generated by ASL 5000 consisted of 30 consecutive breaths obtained from the esophageal pressure in a real patient at the time of a spontaneous breathing trial. For each ICU ventilator and RC combination, two steps were performed: in the first, ATC was not activated and ventilator attached to ASL 5000 without ETT (ATC-ETT-); in the second, ATC was set on at 100% compensation for an ETT 8 mm ID and such an ETT (Shiley Hi contour, Covidien) joined ICU ventilator to ASL 5000 (ATC+ ETT+). The null hypothesis is that VTATC+ ETT+ minus VTATC-ETT—is 0. Primary end point was the breath by breath paired difference betwen ATC+ ETT+ and ATC-ETT-. It was tested to zero for each ventilator in each RC condition.

**Results:** Median VT was 213 ml. Table 1 displays mean (± SD) difference in VT (ml) between ATC+ ETT+ and ATC-ETT-: a negative value means that ATC under delivers and a positive value that ATC over delivers VT for a given patient’s inspiratory effort and RC. In four ventilators (C6, S1, Elisa 800 and 980) ATC almost systematically under delivered VT. In several instances under compensation was greater than 10% median VT. By contrast ATC performed better with the other three ventilators (Evita XL, V500 and Carescape 860).

**Conclusion:** ATC tended to under deliver VT. Furthermore, there were marked differences between ICU ventilators the clinician should be aware of when using the ATC option.

**Compliance with ethics regulations:** NA.

**Table 1:**
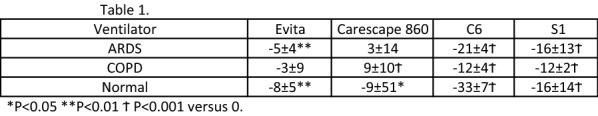
Change in tidal volume between no (Automatic tube compensation) ATC and ATC plus tube

### F-010 Impact of the mechanical power and its components on mortality in patients with ARDS: a post hoc analysis of a controlled randomized study

#### Radj Cally^1^, Claude Guérin^2^, Arnaud Gacouin^3^, Gilles Perrin^1^, Anderson Loundou^1^, Samir Jaber^4^, Jean-Michel Arnal^5^, Dider Perez^6^, Jean-Marie Seghboyan^7^, Jean-Michel Constantin^8^, Jean-Yves Lefrant^9^, Gwenael Prat^10^, Antoine Roch^1^, Laurent Papazian^1^, Jean-Marie Forel^1^

##### ^1^AP-HM, Marseille, France; ^2^CHU de Lyon, Lyon, France; ^3^CHU de Rennes, Rennes, France; ^4^CHU de Montpellier, Montpellier, France; ^5^Centre Hospitalier de Toulon, Toulon, France; ^6^Centre Hospitalier de Lons-le-Saunier, Lons-Le-Saunier, France; ^7^Hôpital Européen, Marseille, France; ^8^CHU de Clermont-Ferrand, Clermont-Ferrand, France; ^9^CHU de Nîmes, Nimes, France; ^10^CHU de Brest, Brest, France

###### **Correspondence:** Radj Cally (radj.cally@yahoo.fr)

*Ann. Intensive Care* 2020, **10 (Suppl 1):**F-010

**Rationale:** During the last decades, identification of factors associated with ventilation-induced lung injury has led to improved survival in patients with ARDS. The mechanical power of ventilation is the total energy transmitted from the ventilator to the respiratory system per unit of time and comprises three different components: elastic related to PEEP, elastic related to tidal volume and resistive. This integrative variable has been recently proposed as an useful predictor of ventilation-induced lung injury and death among ventilated patients. Our goal was to determine the respective impact of the total mechanical power and its three components on the outcome of patients with ARDS.

**Patients and methods:** We performed a post hoc analysis of a randomized, controlled study of patients with ARDS with a PaO2/FiO2 ratio < 150. The mechanical power at inclusion and averaged on the first 2 days after inclusion (total and its three different components) was computed according to the following equation: PowerRS (J/min) = 0.098 Respiratory Rate Tidal Volume [PEEP (1) + ½ Driving Pressure (2) +  (Peak Pressure—Plateau Pressure) (3)], where the (1), (2) and (3) parts correspond respectively to the elastic related to PEEP, elastic related to tidal volume and resistive components. The association between each of these four types of mechanical power evaluated during the first 2 days after inclusion and mortality at D90 was assessed one after the other through multiple logistic regression, allowing control for potential confounding variables at inclusion (age, IGS score without age, group of randomization, PaO2/FiO2, arterial pH).

**Results:** Data from 339 patients were analyzed, among which 115 (33.9%) died before D90. There was no difference concerning the mechanical power at inclusion between survivors and non survivors (either total or its three components). Among the four different types of mechanical power tested during the first 2 days after inclusion, the elastic component related to tidal volume was the only one that was independently associated with mortality at D90 (OR 1.030; 95% CI 1.003–1.058; p = 0.03) (Figure).

**Conclusion:** Our study shows that only the elastic component of the mechanical power related to tidal volume independently predicted mortality at D90 among patients with ARDS, whereas the total mechanical power, its elastic component related to PEEP and its resistive component did not. Further studies are needed to better define how the mechanical power of ventilation could be useful to synthetize the risk of ventilation-induced lung injury.

**Compliance with ethics regulations:** Yes.

**Fig. 1:**
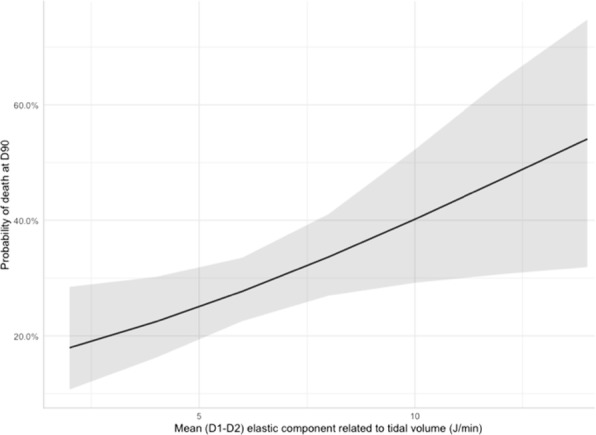
Probability of death at D90 as a factor of mean value (on D1-D2) of the elastic component related to tidal volume of the mechanical power. Others potential confounding variables included in the logistic regression model were age, IGS score without age, group of randomization, PaO_2_/FiO_2_, arterial pH (all at inclusion)

### F-011 A physiological systematic review and meta-analysis on positive end expiratory pressure-induced lung recruitment in patients with acute respiratory distress syndrome

#### Emanuele Turbil^1^, Louis-Marie Galerneau^2^, Antonia Koutsoukou^3^, Jean Jacques Rouby^4^, Jean Dellamonica^5^, Carole Schwebel^2^, Nicolas Terzi^2^, Claude Guérin^6^

##### ^1^University of Sassari, Sassari, Italy; ^2^C.H.U de Grenoble, La Tronche, France; ^3^University of Athens Medical School, Athens, GREECE; ^4^La Pitié Salpétrière Hospital, Paris, France; ^5^CHU de Nice, Nice, France; ^6^Hôpital de la croix rousse, Lyon, France

###### **Correspondence:** Emanuele Turbil (emanuele.turbil@gmail.com)

*Ann. Intensive Care 2020,* **10 (Suppl 1):**F-0011.

**Rationale:** Recruited lung volume (Vrec) elicited by positive end-expiratory pressure (PEEP) can be measured using pressure–volume (P–V) curve. Our purpose was to perform a meta-analysis of Vrec measured by P–V curve in patients with Acute Respiratory Distress Syndrome (ARDS). Primary aim was determination of the prevalence of recruiters (R). Secondary aim was comparison of physiologic data and mortality in R and non-recruiters (NR).

**Patients and methods:** We conducted a PubMed search using the key words: lung recruitment, alveolar recruitment, P–V curve, ARDS, PEEP, humans and adult. All articles were screened by two reviewers. Articles concerning animals, children, case reports and reviews were excluded. Disagreements were resolved by discussion. The same reviewers extracted data in a case record form, which included mortality at ICU discharge, baseline value of anthropometric variables, ARDS cause, physiologic characteristics of patients, ventilator settings, and respiratory mechanics. Vrec ≥ 150 ml was used as the threshold to define R. We first performed a meta-analysis on grouped data, which were pooled applying a random effects model. Then, authors were contacted to obtain individual data. We performed an individual-databased meta-analysis and compared R and NR and survivors and nonsurvivors. Logistic regression (LR) analysis was done to assess the role of Vrec on mortality after adjustment for confounders. These latter were identified by univariate LR analysis between survivors and nonsurvivors. Results were reported as mean, percentage or relative risk (RR), with 95% confidence intervals (CI).

**Results:** From a total of 817 studies, we retrieved 18 articles that included 361 patients on whom the grouped-data meta-analysis. From data on 283 patients, in part coming from original papers’ authors, the individual-data meta-analysis was performed. The prevalence of R was 74% (95% IC 64–84%) in grouped-data and 66% (60–71%) in individual-data analyses. In both grouped- and individual-data analyses there were no significant differences between R and NR for baseline age, male proportion, PEEP, PaO_2_/FIO_2_, ARDS cause, and days in ARDS before the start of the investigation. In grouped-data analysis compliance was significantly higher (mean difference 13.80 (0.14–27.52) ml/cmH_2_O) in R, but this result was not confirmed in individual-data analysis. Finally, RR of mortality in grouped-data was 1.20 (0.88–1.63), which was in accordance with individual-data analysis (p-value = 1). After adjusting for confounding variables (PEEP, PaO_2_/FIO_2_ and Plateau pressure), Vrec was not an independent risk factor of ICU mortality.

**Conclusion:** Most ARDS patients exhibited lung recruitment after increase in PEEP with no correlation with ICU mortality.

**Compliance with ethics regulations:** Yes.

### F-012 Early effects of mechanical ventilation on diaphragm function and its influence on weaning

#### Ahlem Trifi, Farah Ben Lamine, Cyrine Abdennebi, Foued Daly, Yosr Touil, Sami Abdellatif, Salah Ben Lakhal

##### Medical ICU, la Rabta hospital, Faculty of Medicine of Tunis, Tunis, Tunisia

###### **Correspondence:** Ahlem Trifi (trifiahlem2@gmail.com)

*Ann. Intensive Care 2020,* **10 (Suppl 1):**F-012

**Rationale:** To examine the effect of early-stage mechanical ventilation (MV) on diaphragmatic contractility. In the 2nd step, if a diaphragmatic dysfunction was detected, we assessed its influence on the weaning from ventilator.

**Patients and methods:** We measured prospectively the ultrasound-diaphragmatic thickening fraction (DTF) between 2 groups: a study group versus a control group (n = 30 for each). The study group included all adult patients receiving MV, in whom, the DTF was measured within a minimum of 48 h and a maximum of 5 days of MV. For the control group, were enrolled after their approval for participation, adult volunteers in spontaneous ventilation (SV). Patients with factors affecting the diaphragmatic contractility (neuromuscular disease, severe obesity, and neuromuscular blockers…) were excluded. The ultrasound measurements were obtained at the zone of apposition of the right hemithorax. Teleinspiratory and telexpiratory diameters (tid/ted) were taken on the 3 medio-axillary lines: posterior, median and anterior. The DTF was calculated as following: DTF =  (tid-ted/ted) x 100. At the 1st step, the DTFs were compared and at the 2nd step: the relationship between DTF and weaning was analysed.

**Results:** Our 2 groups were comparable in corpulence and co morbidities. The SV group was younger (35 vs. 47 years, p < 0.05) with a predominant female composition. The diaphragmatic exploration concluded that in the MV group, the mean tid tended to be higher but without significant difference (29.1 + 7 versus 26.1 + 5 mm, p = 0.09), the mean ted was significantly higher (20.9 + 6 versus 17.6 + 3.2 mm, p = 0.01) and DTF was significantly lower (39.9 + 12.5% versus 49 + 20.5%, p = 0.043). The ventilation mode had no effect on DTF (40.2 + 13% for control volume vs. 38.6 + 9% for PSV mode, p = 0.8). Fourteen among 30 ventilated patients had a successful weaning with a mean duration of 6 days. A negative correlation was found close to significance between DTF and weaning duration (Rho = − 0.464 and p = 0.08). A DTF value > 33% was associated with weaning success (OR = 2, 95% CI = [1.07–3.7] and p = 0.058) with sensitivity = 85.7%, Specificity = 50%, PPV = 60% and NPV = 80%.

**Conclusion:** The diaphragmatic contractile function was altered from the first days of MV. Weaning duration seemed to be negatively correlated with DTF, and a DTF at the first 5 days of MV greater than 33% was predictive of weaning success.

**Compliance with ethics regulations:** Yes.

### F-013 Shear wave elastography for assessing diaphragm activity in mechanically ventilated patients

#### Quentin Fossé^1^, Thomas Poulard^2^, Jean-Yves Hogrel^2^, Jean-Luc Gennisson^3^, Thomas Similowski^1^, Alexandre Demoule ^1^, Marie Cécile Niérat^4^, Damien Bachasson^2^, Martin Dres^1^

##### ^1^Service de Pneumologie, Médecine Intensive Réanimation, Pitié Salpétrière, GH Universitaire APHP-Sorbonne Université, Paris, France; ^2^Institut de Myologie, Paris, France; ^3^IR4M, Orsay, France; ^4^INSERM UMRS1158 Neurophysiologie respiratoire expérimentale et clinique, Paris, France

###### **Correspondence:** Quentin Fossé (quentin.fosse@gmail.com)

*Ann. Intensive Care 2020,* **10 (Suppl 1):**F-013

**Rationale:** Mechanical ventilation is a life-saving treatment that is however associated with lung injury and/or diaphragm dysfunction. The optimal ventilator settings to provide lung protective ventilation while maintaining safe diaphragm activity are difficult to determine. A noninvasive and bedside evaluation of the diaphragm activity could be helpful in this context. The present study investigated whether changes in diaphragm shear modulus (i.e. stiffness, ΔSMdi) assessed by ultrasound shear wave elastography (SWE) may be used as a surrogate of changes in transdiaphragmatic pressure (ΔPdi) in mechanically ventilated patients.

**Patients and methods:** Patients had to be ventilated for at least 24 h without contraindications for the placement of an œso-gastric catheter. Pdi was monitored continuously and SMdi was measured at the zone of apposition of the right hemi-diaphragm, at 2 Hz sampling rate. Measurements were performed twice under initial ventilator settings and at the end of a weaning trial. Pearson correlation coefficients (r) were computed to determine within-individual correlations between Pdi and SMdi and changes in Pdi and in SMdi occurring between initial ventilator settings and the end of the SBT were compared by a paired test.

**Results:** Twenty-five patients were enrolled and 8 displayed a significant correlation between ΔSMdi and ΔPdi (mean r = 0.73, range = 0.62–0.88, all p < 0.05) (Fig. 1A). Compared to their counterparts, patients with significant within correlations had a lower respiratory rate (16.8 ± 4.7 vs 23.9 ± 6.2breath/min. respectively; p < 0.01) and a significant increase in ΔSMdi (7.3 ± 5.5 kPa vs 13.4 ± 9.0 kPa. p < 0.01) between initial ventilator settings and the SBT. Patients without ΔSMdi-ΔPdi correlation only displayed an increase in ΔPdi (7.9 ± 5.9 vs 14.9 ± 7.9 cmH_2_O, p < 0.01) at the end of the SBT with no concomitant significant increase in ΔSMdi (7.6 ± 3.8 kPa vs 7.9 ± 5.3 kPa, p > 0.05). (Fig. 1B).

**Conclusion:** SMdi obtained by SWE appears as a promising technique to assess diaphragm activity in mechanically ventilated patients but technological improvements are necessary to increase SWE sampling rate before enabling its generalization in the ICU.

**Compliance with ethics regulations:** Yes.

**Fig. 1:**
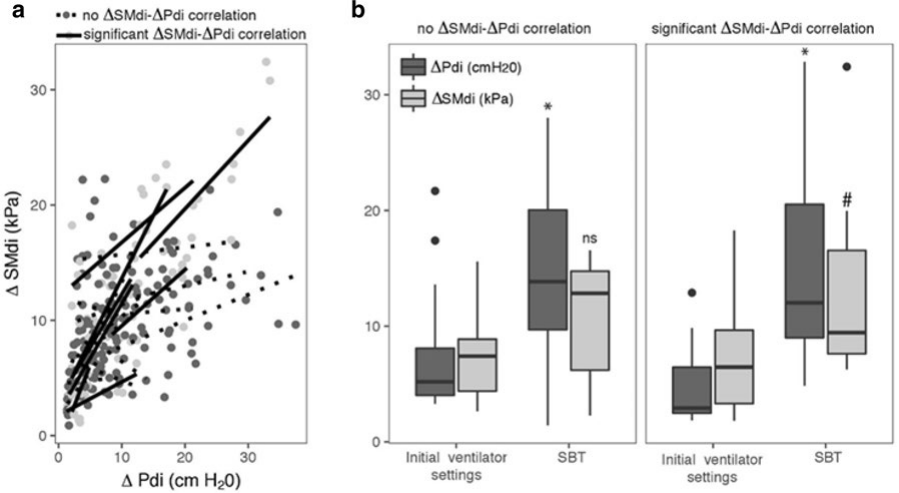
**a** Relationship between changes and diaphragmatic shear modulus.** b** ΔPdi and ΔSMdi under initial ventilator settings and at the end of the SBT

### F-014 Inspiratory effort can be measured during spontaneous breathing at the bedside: illustration of feasibility

#### Davy Cabrio, Filippo Bongiovanni, Philippe Eckert, Lise Piquilloud

##### CHUV, Centre Hospitalier Universitaire Vaudois, Lausanne, Switzerland

###### **Correspondence:** Davy Cabrio (cabriodavy@gmail.com)

*Ann. Intensive Care 2020,* **10 (Suppl 1):**F-014

**Rationale:** End-inspiratory (EIP) and end-expiratory (EEP) pauses are commonly used during volume assist control ventilation to assess plateau pressure and total positive end-expiratory pressure (PEEPtot). They can also be used during assisted ventilation (AV) for muscle pressure assessment. It requires ventilators able to perform EIP during AV. Plateau pressure (Pplat) usually increases in AV during EIP due to “hidden” inspiratory effort. Pressure muscular index (PMI) is equal to Pplat minus the sum of PEEPtot (measured during an EEP) and set pressure support (PS); it theoretically reflects patient’s effort without esophageal pressure (Pes) monitoring. Pes is the gold standard method to assess inspiratory muscle pressure (Pmus, difference of Pes drop at neural end-inspiration and correction factor for chest wall elastance and tidal volume). We aimed to illustrate the feasibility of measuring PMI using a standard ICU ventilator at the bedside and study the correlation between Pmus and PMI.

**Patients and methods:** Measurements were recorded in 4 ICU patients. Pes was measured using an nasogastric probe (equipped with an esophageal balloon) inserted for advanced monitoring (severe acute respiratory distress syndrome—ARDS) or for a study protocol (difficult weaning after COPD exacerbation). Recorded EIP, EEP and Pes were used for post hoc analyses. Results reported as ranges and median [IQR]. Correlation between Pmus and PMI tested with Spearman correlation test.

**Results:** 25 out of 28 EIP and EEP duos could be analyzed (2-esophageal spasm/1-calibration error). Ventilator mode was pressure support ventilation (PS 0–12 cmH2O). Pplat = 17.9 [15.1–21.4] cmH2O, PEEPtot = 5.5 [5–8.3] cmH2O, Pmus = 3.5 [1.7–9.5] cmH2O, PMI = 4.5 [2.4–7.8]. For all recordings, Spearman r coefficient between Pmus and PMI was 0.438 (p = 0.028).

**Conclusion:** Muscular effort can be assessed in AV using EIP and EEP using ICU ventilators. However, recordings can be influenced by expiratory muscles contraction. Patient’s ability to follow directions during the maneuvers is an important factor to obtain reliable values. There seem to be a correlation in our small sample between muscular pressure assessed without and with Pes.

**Compliance with ethics regulations:** Yes.

### F-015 Innate T cells during severe pneumonia and Acute Respiratory Distress Syndrome

#### Youenn Jouan^1^, Chloé Boisseau^2^, Antoine Guillon^1^, Yonatan Perez^1^, Pierre-François Dequin^1^, Mustapha Si-Tahar^2^, Christophe Paget^2^

##### ^1^Service de Médecine Intensive Réanimation, Tours, France; ^2^INSERM U1100, Tours, France

###### **Correspondence:** Youenn Jouan (youenn.jouan@gmail.com)

*Ann. Intensive Care 2020,* **10 (Suppl 1):**F-015

**Rationale:** Severe pneumonia can culminate in acute respiratory distress syndrome (ARDS). An uncontrolled inflammatory response is a key feature favoring transition towards ARDS. However, the underlying mechanisms remain poorly understood. In this context, the contribution of “innate T cells” (ITC) -a family of non-peptide reactive T cells comprising NKT cells, Mucosal Associated Invariant T (MAIT) cells and γδT cells—has never been explored. ITC have emerged as key players in orchestration of the host response during infections and inflammation processes. For these reasons, these cells are already seen as potential therapeutic targets in other medical fields (especially oncology). Here, we hypothesized that a tight regulation of their functions could be paramount to control the inflammatory response and to prevent ARDS development.

**Patients and methods:** To explore this, we combined a murine model of influenza A virus (IAV) infection mimicking ARDS symptoms and a clinical study recruiting patients admitted in ICU for severe pneumonia. Using flow-cytometry approaches, we investigated (1) the abundance and dynamics of ITC in various compartments, (2) their pattern of activation/regulation markers (respectively CD69 and PD-1) and (3) their cytokine production.

**Results:** During experimental IAV pneumonia, ITC were transiently recruited into the airways. Unlike γδT and NKT, MAIT cells phenotype was largely changed, displaying a progressive CD69 overexpression and increased IL-17A production. During the resolution phase, up to 90% of pulmonary MAITs expressed PD-1 (versus < 10% in controls), which can suggest emergence of regulatory functions. Last, using gene-targeted mice, we suggested that MAIT cells confer a protective effect during pneumonia. In the ongoing clinical study, the proportion of circulating MAIT cells in patients was markedly decreased compared to controls (1.0 ± 1.0% versus 5.7 ± 2.8% of T cells), but not for NKT or γδT cells. Notably, some patients with severe ARDS presented detectable levels of MAITs in their respiratory fluids. In addition, circulating MAIT cells in patients overexpressed CD69 and PD-1 (56.5% and 55% respectively), but with a reduced proportion able to produce IL-17 and IFNγ, compared to healthy controls. Lastly, proportion of activated (CD69 +) MAIT cells significantly decreased with clinical improvement.

**Conclusion:** This translational approach combining in vivo animal experiments and clinical samples with ex vivo experiments indicates a preferential modulation in MAIT cells functions during severe pneumonia. These data justify an in-depth analysis of MAIT cells activation mechanisms and functions in this context, in order to further explore a potential use as a disease-progression marker and -in a long term perspective—as a potential therapeutic target.

**Compliance with ethics regulations**: Yes.

**Fig. 1:**
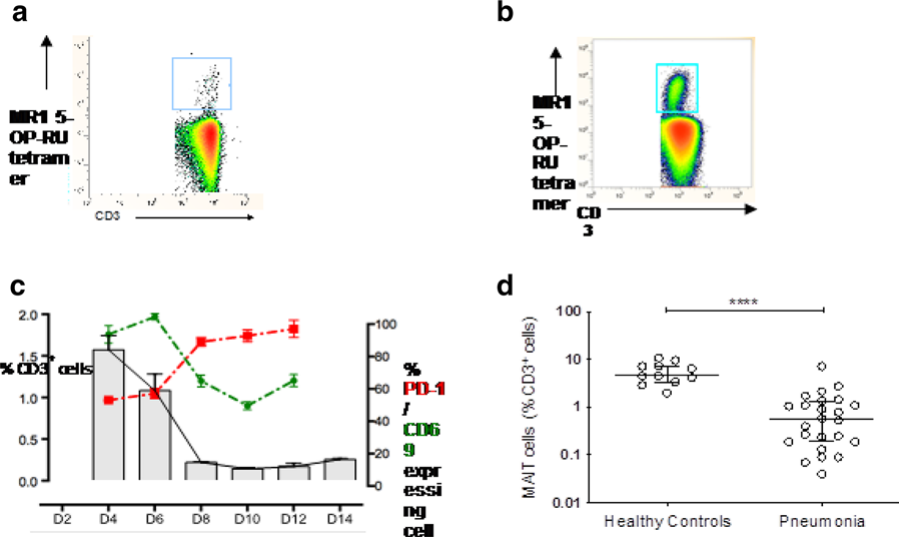
Representative flow-cytometry dot-plots of MAIT cells labelling using fluorophore-conjugated MR1 tetramers loaded with 5-OP-RU from lungs of an infected mouse (A) and blood sample of a patient with pneumonia (B). C: frequency of MAIT cells, proportion of CD69 and PD-1 + MAIT cells in bronchoalveolar lavage during experimental murine pneumonia. D: blood frequency of MAIT cells in patients with pneumonia compared with healthy controls (as % of total T cells)

### F-016 A dominant circulating PD-1/2B4 CD8+ T-cells pattern at day 1 correlates with mortality but not with secondary infections in septic patients admitted to the ICU

#### Damien Guinault^1^, Marie-Laure Nicolau-Travers^2^, Stein Silva^3^, Arnaud Del Bello^1^, Olivier Cointault^1^, Edith Hourcastagnou^3^, David Rousset^3^, Laurence Lavayssiere^1^, Marie-Béatrice Nogier^1^, Arnaud Mari^3^, Nassim Kamar^1^, François Vergez^2^, Stanislas Faguer^1^

##### ^1^Département de Néphrologie et Transplantation d’Organes, CHU de Toulouse, Toulouse, France; ^2^Laboratoire d’Hématologie-Immunologie, CHU de Toulouse, Toulouse, France; ^3^Département d’Anesthésie et Réanimation-Réanimation Médicale, CHU de Toulouse, Toulouse, France

###### **Correspondence:** Damien Guinault (guinault.d@chu-toulouse.fr)

*Ann. Intensive Care 2020,* **10 (Suppl 1):**F-016

**Rationale:** Immune paralysis following hyperinflammatory states increases the risk of secondary infections and death. Reversing T-cells exhaustion using recombinant IL7 or immune checkpoints inhibitors may improve the prognosis of patients with sepsis admitted to the ICU. However, there is an unmet need to better characterize the state of T-cells exhaustion in these patients, its reproducibility and its correlation with the outcomes before implementing immunotherapy in the therapeutic armamentarium against sepsis.

**Patients and methods:** Prospective observational cohort study performed in two tertiary-care ICUs in a university hospital. Peripheral blood mononuclear cells were collected at day 1 in 80 adult patients with sepsis admitted to the ICU. The level of CD4+ and CD8+ T-cells exhaustion was quantified using multi-color flux cytometry targeting the following exhaustion markers: PD-1, 2B4 and CD160. CD4+ regulatory T-cells (CD3+ CD4+ CD25hi CD127Lo cells) were also assessed.

**Results:** The 80 patients included in the study could be split in five clusters according to their dominant pattern of exhaustion markers on CD8+ T-cell (i.e. no markers, PD-1+, 2B4+, 2B4+ CD160+ and 2B4+ PD-1+) and independently of their underlying morbidities. No patients harbored a fully exhausted triple-positive pattern. By multivariate analysis, SAPS2 gravity score at day 1 (p = 0.007), a dominant 2B4 and/or PD-1 CD8+ pattern (p = 0.04) and lung sepsis (p = 0.02) where associated with the risk of death at day 28, whereas hemoglobin level was associated with survival (p = 0.04). No CD4+ or CD8+ exhaustion pattern independently predicted the risk of secondary infections. Neither the level of CD4+ regulatory T-cells nor the dominant CD4+ exhaustion pattern was associated with the outcomes.

**Conclusion:** CD8+ T-cells of individuals with sepsis may express one or two exhaustion markers at the admission to the ICU but fully exhausted phenotype is rare, suggesting an intermediate state of T-cell activation or differentiation. A dominant 2B4 and/or PD-1 CD8+ pattern at day 1 is independently associated with mortality.

**Compliance with ethics regulations:** Yes.

### F-017 Comparison of one-year outcome of severe community-acquired bacterial or viral pneumonia and pneumonia of unidentified etiology

#### Frédéric Sangla^1^, Pierre-Emmanuel Marti^2^, David Legouis^2^, Amélie Brebion^3^, Pierre Saint-Sardos^4^, Alexandre Lautrette^5^, Mireille Adda^5^, Bruno Pereira^6^, Bertrand Souweine^5^

##### ^1^CHU de Clermont-Ferrand, Clermont-Ferrand, France; ^2^Service de Soins Intensifs adultes, Hopitaux Universitaires de Genève, Genève, Switzerland; ^3^Laboratoire de Virologie, CHU Clermont-Ferrand, CNRS, Université Clermont Auvergne, Clermont-Ferrand, France; ^4^Laboratoire de Bactériologie, CHU Clermont-Ferrand, CNRS, INSERM, Université Clermont Auvergne, Clermont-Ferrand, France; ^5^Service de Médecine Intensive et réanimation, CHU Clermont-Ferrand, CNRS, Université Clermont Auvergne, Clermont-Ferrand, France; ^6^Département de Biostatistique, CHU Clermont-Ferrand, Clermont-Ferrand, France

###### **Correspondence:** Frédéric Sangla (fred.sangla@gmail.com)

*Ann. Intensive Care 2020,* **10 (Suppl 1):**F-017

**Rationale:** There is growing use of multiplex polymerase chain reaction (mPCR) for respiratory virus testing in patients with community-acquired pneumonia (CAP). Data on one-year outcomes in patients with severe CAP of bacterial, viral and unidentified etiology are scarce.

**Patients and methods:** A single-center retrospective study was performed in 123 intensive care unit (ICU) patients with known one-year survival status who had undergone respiratory virus testing for CAP by mPCR. One year after ICU admission, mortality rates and functional status were compared in patients with CAP of bacterial, viral or unidentified etiology.

**Results:** There were 19 (15.4%) patients in the bacterial group, 37 (30.1%) in the viral group and 67 (54.5%) with unidentified etiology. One-year mortality was 57.9% (n = 11/19), 27% (n = 10/37) and 28.4% (n = 19/67), respectively (p = 0.046). In multivariate analysis, one-year mortality was higher in the bacterial group than in the viral group (HR 2.92, 95% IC 1.71–7.28, p = 0.02), had a trend to be higher in the bacterial group compared to the unidentified etiology group (HR 2.07, 95% IC 0.96–4.45, p = 0.06) and was not different between the viral and unidentified etiology groups (HR 0.71, 95% IC 0.30–1.65, p = 0.43). Severe dyspnea (mMRC score = 4 or death), major adverse respiratory events (new homecare ventilatory support or death) and severe autonomy deficiencies (ADL Katz score ≤ 2 or death) were observed in 52/104 (50.0%), 65/104 (62.5%) and 47/104 (45.2%) patients, respectively, with no difference between groups.

**Conclusion:** CAP of bacterial origin was associated with a poorer prognosis than viral or unidentified etiology. Impaired functional status was observed in a substantial proportion at one-year, irrespective of the causative microorganisms involved.

**Compliance with ethics regulations:** Yes.

### F-018 Interest of UNYVERO multiplex PCR (CURETIS) for BAL rapid microbiologic and antibiotic susceptibility documentations in immunocompromised patients under antibiotic therapy

#### Jean-Luc Baudel^1^, Jacques Tankovic^2^, Redouane Dahoumane^2^, Salah Gallah^2^, Laurent Benzerara^2^, Jean-Remy Lavillegrand^1^, Razach Abdallah^1^, Geoffroy Hariri^1^, Naike Bige^1^, Hafid Ait-Oufella^1^, Nicolas Veziris^2^, Eric Maury^1^, Bertrand Guidet^1^

##### ^1^Service de Réanimation Médicale, Hôpital Saint-Antoine, Paris, France; ^2^Service de Bactériologie, Hôpital Saint-Antoine, Paris, France

###### **Correspondence:** Jean-Luc Baudel (jean-luc.baudel@aphp.fr)

*Ann. Intensive Care 2020,* **10 (Suppl 1):**F-018

**Rationale:** Our aim was to evaluate the interest of the Unyvero rapid (4.5 h) multiplex PCR assay (performed on bronchoalveolar lavage [BAL] samples) for the management of immunocompromised patients already treated with antibiotics and diagnosed with pneumonia (according to clinical and radiological findings). We thus performed an observational study that compared the results (and the length of time to obtain them) of routine microbiological evaluation and Unyvero assay.

**Patients and methods:** From July 2018 to January 2019 and from April to August 2019, we examined BAL samples from immunocompromised patients (coming from hematology, oncology, hepatology, gastroenterology, internal medicine, and neurology units) diagnosed with pneumonia (based on clinical and radiological findings), and already receiving antibiotic treatment. The following data were collected: age, gender, SAPS2 score, lung CT scan (92%) or X-ray (8%) results, duration and content of prior antibiotic therapy, direct examination, culture, antibiogram and Unyvero results, secondary confirmation of pneumonia or not, possible changes in antibiotic therapy that could have been made after obtention of Unyvero results. Informed consent was obtained from all patients.

**Results:** 40 BAL samples were analyzed in 38 immunocompromised patients (m/f ratio 2.17, SAPS2 51.5 ± 6.8) mostly with hematologic (76%) or oncologic (13%) diseases. The patients received either corticosteroids (47%), or chemotherapy (37%), or immunotherapy (8%). 40% of the patients were under mechanical ventilation, 15% under Optiflow. 32% presented a shock, 22% had aplasia or neutropenia, 24% were allografted, 16% were autografted. The duration of prior antibiotic therapy at the time of BAL were 9.3 ± 5.6 days. Direct examination was positive in 22.5% of the cases, culture (both above and under the classical threshold of 104 CFU/ml) in 60%, Unyvero in 47.5%. A retrospective analysis of all the cases confirmed the initial diagnosis of pneumonia in only 50% of the cases. Compared to culture, the sensitivity of Unyvero was 81%, its specificity 94%. Unyvero could permit to rapidly deescalate antibiotic therapy in 42% of the cases and to rapidly stop it in 32%.

**Conclusion:** The Unyvero assay on BAL samples is useful in this specific population for rapid obtention of microbiological results and also for confirmation of the negativity of cultures and thus permits a better management of antibiotic therapy, leading to a reduction of antibiotic resistance selection pressure in the ICU.

**Compliance with ethics regulations:** Yes.

### F-019 Do not underestimate RSV pneumonia among critically ill patients

#### Erwan Begot^1^, Suzanne Champion^1^, Charline Sazio^1^, Benjamin Clouzeau^1^, Alexandre Boyer^1^, Hoang-Nam Bui^1^, Marie-Edith Lafon^2^, Camille Ciccone^2^, Julia Dina^3^, Didier Gruson^1^, Renaud Prével^1^

##### ^1^CHU Bordeaux, Medical intensive care unit, Bordeaux, France; ^2^CHU Bordeaux, Virology laboratory, Bordeaux, France; ^3^National Reference Center for Measles Mumps and Rubella, CHU de Caen, Caen, France

###### **Correspondence:** Erwan Begot (erwan.begot@chu-bordeaux.fr)

*Ann. Intensive Care 2020,* **10 (Suppl 1):**F-019

**Rationale:** Respiratory syncitial virus (RSV) is a well-known cause of respiratory failure among neonates but its pathogenicity in adults is now emerging as a potential cause of viral pneumonia. Data are limited with conflicting results regarding RSV pneumonia severity in adults. Data are lacking about critically ill RSV patients’ characteristics and outcomes. The aim of this study is to compare RSV patients’ characteristics, care and outcomes to influenza patients’ ones.

**Patients and methods:** Patients diagnosed with RSV and influenza pneumonia admitted to our medical ICU were included. Data were retrospectively recorded. Quantitative data are expressed by median and interquartile range and compared by use of Mann–Whitney test. Qualitative data are expressed by number and percentages and compared by use of Fischer exact t-test. RSV strains were prospectively collected.

**Results:** Eighteen critically ill patients with RSV pneumonia and 95 with influenza pneumonia were included. RSV and influenza patients had the same characteristics at admission except for age (respectively 71yo [62; 81] vs 63yo [51; 70], p: 0.03). In particular, they had similar rates of underlying chronic pulmonary disease (respectively 10/18 (56%) vs 43/95 (45%), p = 0.45) or haematological malignancy (2/18 (12%) vs 9/95 (10%), p = 1.00). They exhibited similar presentation with similar SAPSII scores (respectively 46 [38; 63] vs 53 [34; 74], p = 0.55), PaO_2_/FiO_2_ ratio (respectively 195 [145; 256] vs 157 [100; 226], p = 0.15) and acute respiratory distress syndrome rates (respectively 7/18 (39%) vs 47/95 (49%), p = 0.44). They received similar treatment as suggested by oro-tracheal intubation rates (respectively 6/18 (33%) vs 52/95 (54%), p: 0.12) and antibiotics prescription (respectively 16/18 (89%) vs 88/95 (93%), p: 0.63). RSV and influenza patients also had the same rates of bacterial co-infections (4/18 (22%) vs 28 (29%), p: 0.78). Invasive aspergillosis remained a rare event but also occurred among RSV patients (1/18 (6%) vs 3/95 (3%), p: 0.51). Acute coronary syndromes were as frequent in both groups (respectively 2/18 (12%) vs 9/95 (10%), p = 1.00). Day-28 mortality was similar between RSV and influenza patients (respectively 3/18 (19%) vs 23 (24%), p = 0.76). RSV strains typing is under progress.

**Conclusion:** Patients suffering from RSV pneumonia admitted to ICU have similar presentation, care and day-28 mortality than critically ill influenza patients. The severity of RSV pneumonia diagnosis should not be underestimated by intensivists.

**Compliance with ethics regulations**: Yes.

### F-020 Determinants of respiratory distress from seawater drowning: a prospective observational study from seven intensive care units on the French Riviera

#### Alexandre Robert^1^, Denis Doyen^2^, Pierre-Marie Bertrand^3^, Michel Kaidomar^1^, Nicolas Clement^3^, Nicolas Bele^1^, Nihal Martis^2^, Hervé Quintard^4^, Hervé Hyvernat^2^, Gilles Bernardin^2^, Jean Dellamonica^2^

##### ^1^Fédération des réanimations du Var Est, Fréjus-Draguignan, France; ^2^Médecine Intensive Réanimation, Hôpital l’Archet 1, CHU de Nice, Nice, France; ^3^Médecine Intensive Réanimation, CH de Cannes, Cannes, France; ^4^Réanimation polyvalente, Hôpital Pasteur 2, CHU de Nice, Nice, France

###### **Correspondence:** Alexandre Robert (alex_robert@hotmail.fr)

*Ann. Intensive Care 2020,* **10 (Suppl 1):**F-020

**Rationale:** Respiratory distress from seawater drowning is commonly considered multifactorial. Etiologies are debatable and include heart failure, infection and acute respiratory distress syndrome (ARDS). Documented bacterial infections seems mostly related to the site of drowning. Data in this regard are scarce with prospective studies lacking. The objective of our study was to describe prospectively the characteristics and determinants of respiratory distress from Seawater Drowning.

**Patients and methods:** All patients admitted for seawater drowning to seven intensive care units (ICU) on the French Riviera in the summers of 2017 and 2018 were prospectively included. Recorded data included clinical features on examination, personal history, chest X-rays, echocardiography and biological results obtained within the first 48 h. A paired Student’s t-test was used to study statistical differences between quantitative variables on admission and during early evaluation (i.e. first 48 h).

**Results:** Forty-eight patients were admitted to seven centers of which 45 (94%) were diagnosed as having ARDS, 31 (65%) early pneumonia and 4 (8%) acute cardiogenic pulmonary edema. Twenty-one (44%) respiratory samples were collected but bacterial culture was positive in only 5 cases. Multidrug-resistant bacteria were not observed, and amoxicillin-clavulanate as first-line treatment was effective in all cases. Echocardiography performed in 38 (75%) patients was normal and unable to identify specific patient profiles. The median Clinical Pulmonary Infection Score (CPIS) on admission was 6 (IQR, 5–7) and decreased rapidly and significantly (p < 0.0001) within 24 h to 3 (IQR, 2–3) (Fig. 1).

**Conclusion:** Data from this multicenter cohort suggest that respiratory distress following seawater drowning can mimic bacterial pneumonia during the first 24 h with subsequent rapid clinical improvement in patients admitted to the ICU. Probabilistic antibacterial therapy should therefore be limited to the most severe patients. Isolate ARDS is often the only etiology found and is resolutive within 24 h. This prospective cohort is the largest of its kind and gives a better insight into the limited impact of cardiogenic and infectious processes on sea drowning-related respiratory distress.

**Compliance with ethics regulations:** Yes.

**Fig. 1:**
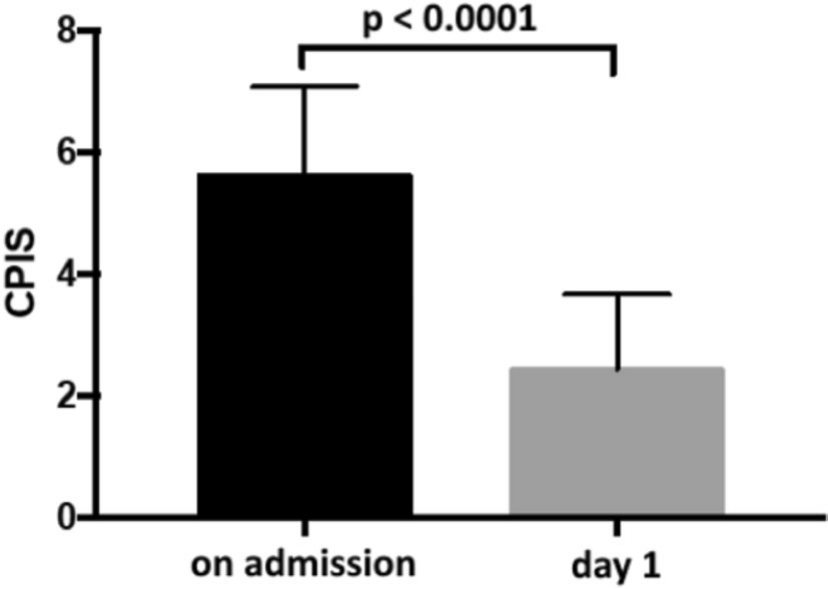
Modifications in Clinical Pulmonary Infection Scores (CPIS) from admission, to day 1 of follow-up in seawater drowned patients admitted to the ICU. Scores were compared using a Student’s paired T-test

### F-021 Impact of Extra-Corporeal Membrane Oxygenation (ECMO) support on piperacillin exposure in septic patients: a case–control study

#### Pierre Fillatre^1^, Florian Lemaitre^2^, Nicolas Nesseler^3^, Matthieu Schmidt^4^, Sebastien Besset^4^, Yoann Launey^5^, Adel Maamar^6^, Pierre Daufresne^7^, Erwan Flecher^8^, Yves Le Tulzo^9^, Jean-Marc Tadié^9^, Pierre Tattevin^9^

##### ^1^Service de Réanimation Polyvalente, Saint Brieuc, France; ^2^Service de Pharmacologie-CHU Pontchaillou, Rennes, France; ^3^Service de Réanimation de Chirurgie Thoracique et de Chirurgie Vasculaire-CHU Pontchaillou, Rennes, France; ^4^Service de Réanimation Médicale-CHU La Pitié Salpétrière, Paris, France; ^5^Service de Réanimation Chirurgicale-CHU Pontchaillou, Rennes, France; ^6^Service de Maladies Infectieuses et de Réanimation Médicale-CHU Pontchaillou, Rennes, France; ^7^Service d’hématologie-CHU Pontchaillou, Rennes, France; ^8^Service de Chirurgie Thoracique et de Chirurgie Vasculaire-CHU Pontchaillou, Rennes, France; ^9^Service de Maladies Infectieuses et de Réanimation Médicale-CHU Pontchaillou, Rennes, France

###### **Correspondence:** Pierre Fillatre (pierrefillatre@hotmail.com)

*Ann. Intensive Care 2020,* **10 (Suppl 1):**F-021

**Rationale:** Patients treated with “ExtraCorporeal Membrane Oxygenation” (ECMO) are at a higher risk of developing nosocomial infections and they are consequently often treated with beta-lactams. French guidelines recommend obtaining beta-lactam trough concentrations above four times the minimal inhibitory concentration (MIC) of the causative bacteria. The ECMO device may alter the pharmacokinetics of these medications, which may result in underexposure to beta-lactam antibiotics.

**Patients and methods:** This observational, prospective, multicenter, case–control study was performed in the intensive care units of two tertiary care hospitals in France. ECMO Patients with sepsis treated with piperacillin-tazobactam were enrolled. Control patients were matched according to SOFA score and creatinine clearance. The pharmacokinetics of piperacillin was described based on a population pharmacokinetic model, allowing to calculate the time spent above 4 × the MIC breakpoint for Pseudomonas aeruginosa susceptibility after the first dose and at steady state between two piperacillin infusions.

**Results:** Forty-two patients were included. The median age was 60 years [49–66], the SOFA score was 11 [9–14], and median creatinine clearance was 47 mL/min [5–95]. There was no significant difference in the time above 4 x MIC in patients treated with ECMO and controls during the first administration (p = 0.184) and at steady state (p = 0.309). There was no significant difference between the trough at steady state (p = 0.535), with 18/42 patients (43%) exhibiting concentrations of piperacillin lower than 4 x MIC. ECMO support was not associated with a steady state trough concentration below 4 x MIC (OR = 0.5 [0.1–2.1], p = 0.378). The only variable independently associated with this risk was a creatinine clearance ≥ 40 mL/min, (OR = 4.3 [1.1–17.7], p = 0.043).

**Conclusion:** ECMO support has no significant impact on piperacillin exposure. Intensive care unit patients with sepsis are, however, frequently underexposed with piperacillin, which suggest that therapeutic drug monitoring should be strongly recommended for severe infections.

**Compliance with ethics regulations:** Yes.

**Fig. 1:**
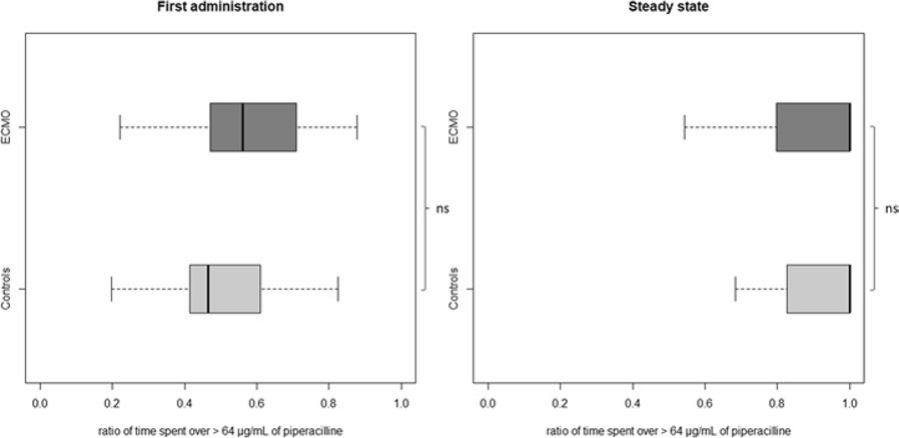
Ratio of the time between two piperacilline infusion spent above 64 µg/mL during the first administration or at steady state in ECMO patients and in control patients

### F-022 End-of-life management strategies the opinion of Tunisian intensivists in the Vincent lambert’s case

#### Meriem Fatnassi^1^, Hela Maamouri^1^, Nassereddine Foudhaili^1^, Nozha Brahmi^1^

##### ^1^Service de réanimation toxicologique et polyvalente centre Mahmoud Yaacoub, Tunis, Tunisia

###### **Correspondence:** Meriem Fatnassi (merifatnassi@gmail.com)

*Ann. Intensive Care 2020,* **10 (Suppl 1):**F-022

**Rationale:** Decision-making at the end of life is a very difficult situation frequently encountered by Intensivists. The media debate over the case of Vincent Lambert focused on issue related to the end-of-life management strategies. This study aims to discover the opinion of Tunisian physicians in different hospitals.

**Patients and methods:** We have realized a questionnaire composed of 17 items. Sixty intensivists participated to our study. They have different status in intensive care and anesthesia: 10 professors, 26 senior doctors and 24 interns.

**Results:** We collected 60 opinions. 96.6% of intensivists had taken care of patients in vegetative coma and 88.1% (n = 52) agreed with the fact that patients in vegetative coma are eligible to withold or even withdraw treatment. 50.8% were against active treatment limitating decisions. It was related to humanitarian believes in 62.1% of cases and to religious convictions in 20.7%. The other causes were moral, legal and ethic. 83.9% thought that the treatment limitation decision was not prohibited by Islamic religion. Furthermore, 79.3% approved passive limiting care et 83.1% were against euthanasia. In pratice, 72.9% have used deep sedation as palliative care. The common methods of care limiting were: not using catecholamines in 94.9%, stopping enteral and parenteral nutrition in 88.1% and administration of deep sedation while maintaining the intubation in 62.7%. Most of intensivists (96.6%) recognized that Vincent lambert’s affair raised the problem of end-of-life management. 59.3% agreed with the medical and the partner decision. 56.1% found that the lawsuit initiated by parents to human rights was disproportionate and that media influenced negatively the matter. Finally, the majority of doctors suggested that advance directives may help in those situations.

**Conclusion:** The end of life management remains a subject of controversy. Advance directives and formulated laws such as Leonetti law can be helpful.

**Compliance with ethics regulations**: Yes.

### F-023 Impact of a visual support dedicated to prognosis of patients on symptoms of stress of family members

#### Pascal Beuret^1^, Gabrielle Burelli^1^, Arthur Vincent^1^, Jeremy Mallard^1^, Sarah Meffre^1^, Alizee Maarek^1^, Sixtine Bonnet^1^, Jerome Morel^2^

##### ^1^Réanimation polyvalente/Centre hospitalier, Roanne, France; ^2^Réanimation polyvalente B/CHUI, Saint Etienne, France

###### **Correspondence:** Pascal Beuret (pascal.beuret@ch-roanne.fr)

*Ann. Intensive Care 2020,* **10 (Suppl 1):**F-023

**Rationale:** Family members commonly have inaccurate expectations of patient’s prognosis. Adding to classic oral information a visual support, depicting day by day the evolution of the condition of the patient, improves the concordance in prognosis estimate between physicians and family members. The objective of this study was to evaluate the impact of this support on symptoms of anxiety/depression of family members.

**Patients and methods:** We conducted a bi-center prospective before-after study. All consecutive patients admitted in the two ICUs were eligible. In the before period (3 months), family members received classic oral information. In the after period (3 months), in addition to classic oral information, the visual support (Fig. 1) was available for family members in the patient’s room from the day of admission until discharge from the ICU. At day 5 and 90 from admission, symptoms of anxiety/depression of referent family member were evaluated by Hospital Anxiety and Depression Scale (HADS).

**Results:** 140 patients and their referent family members were included (77 in period before and 63 after). Characteristics of patients of the two groups were similar regarding age, reason for admission, SAPS II at admission and SOFA score at day 5. Also characteristics of referent family members were comparable in terms of age, sex ratio, type of relationship with the patient and number of visits since admission. At day 5, total HAD score was 17 [9–25] in the group before without the support and 15 [10–22] in the group after with the support (p = 0.43). The prevalence of symptoms of anxiety (HAD-A score > 7) and depression (HAD-D score > 7) was similar in the two groups (respectively 66.2% and 49.4% in the group before, and 68.3% and 36.5% in the group after (NS)). At day 90, total HAD score was 11 in the group before [7–16] and 9 [5–16] in the group after (p = 0.38). By multivariate analysis the following factors were significantly associated with total HAD score > 12 at day 5: age of patient (OR 0.98 [0.96–0.999]), number of visits of referent (OR 2.72 [1.09–6.76]) and previous or current treatment of referent for anxiety or depression (OR 2.76 [1.08–7.06]).

**Conclusion:** In this study, the use of a visual support dedicated to prognosis of patients did not modify the level of stress of family members.

**Compliance with ethics regulations:** Yes.

**Fig. 1:**
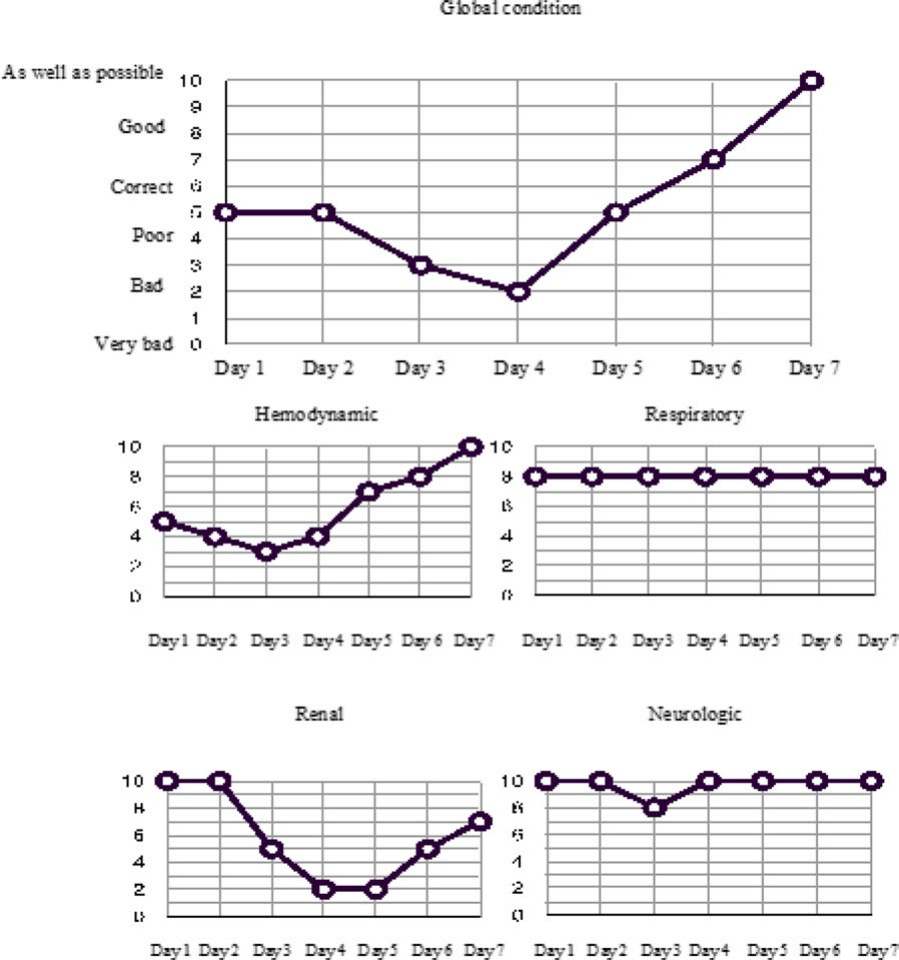
An example of visual support

### F-024 Sedation practice and discomfort during withdrawal of mechanical ventilation in critically ill patients at End-of-Life: a post hoc analysis of a multicenter study

#### René Robert^1^, Amélie Le Gouge^2^, Jean Reignier^3^

##### ^1^Université de Poitiers, Poitiers, France/Inserm CIC 1402, ALIVE, service de Médecine Intensive Réanimation, Poitiers, France; ^2^Inserm CIC 1415, Tours, Tours, France; ^3^Université de Nantes, Nantes, France

###### **Correspondence:** René Robert (r.robert@chu-poitiers.fr)

*Ann. Intensive Care* 2020, **10 (Suppl 1):**F-024

**Rationale:** The use of sedation and opioids at the end of life is a topic of considerable ethical debate. Incidence of discomfort during the end-of-life of ICU patients and impact of sedation on discomfort are poorly known.

**Patients and methods:** Post-hoc analysis of an observational prospective multicenter study comparing terminal weaning vs. immediate extubation for end-of-life in ICU patients, aimed at assessing the incidence of discomfort events according to levels of sedation. Discomforts including gasps, significant bronchial obstruction or high behavioral pain scale score, were prospectively assessed by nurses from mechanical ventilation withdrawal until death. Level of sedation was assessed using the Richmond Agitation Sedation Scale (RASS).

**Results:** Among the 450 patients included in the original study, 226 (50%) experienced discomfort after mechanical ventilation withdrawal. Patients with discomfort received lower doses of midazolam and equivalent morphine, and less frequently had deep sedation (RASS -5) than patients without discomfort (59% vs 79%, p < 0.001). After multivariate logistic regression, immediate extubation was the only factor associated with discomfort whereas deep sedation and administrations of vasoactive drugs were two factors independently associated with no discomfort. Death occurred less rapidly in patient with discomfort than in those without discomfort (7.3 h [1.9–25.0] vs 1.6 [0.3–6.0], P < 0.0001) (Figure). Long-term evaluation of psychological disorders in family members of dead patients did not differ between those with discomfort and the others.

**Discussion:** Despite the theoretically expected anticipatory titrated doses of opioids and benzodiazepines to alleviate any discomfort after withdrawal of mechanical ventilation, half of the patients did not receive sedation or opiate when the decision to withdraw mechanical ventilation was taken. A major point that could interfere with the continuous deep sedation practice until death is the fear of potentially hastening death, and there is much controversy regarding its proper use in end-of-life care.

**Conclusion:** Discomfort was frequent during end-of-life of ICU patients and was mainly associated with terminal extubation and less profound sedation.

**Compliance with ethics regulations:** Yes.

**Fig. 1:**
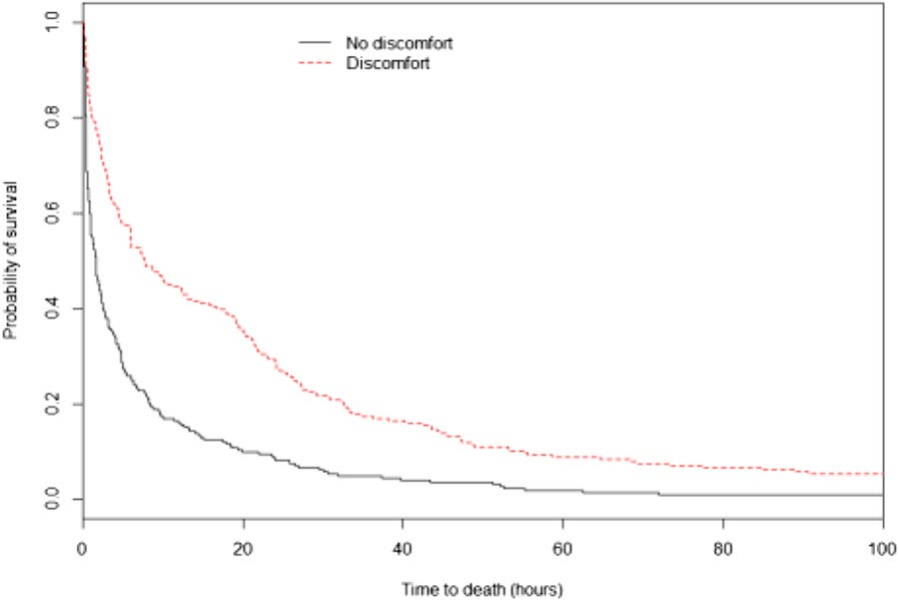
Survival probability in patients with and without discomfort

### F-025 Post-death meeting for relatives of ICU deceased patients: a way to alleviate grief symptoms?

#### Thibault Dekeyser, Dominique Malacrino, Caroline Sejourne, Marie-Christine Dufossez, Mehdi Marzouk, Imen Rahmani, Laura Lstiburek, Christophe Vinsonneau

##### Service de Médecine Intensive Réanimation, Centre hospitalier de Béthune Germon et Gauthier, 62408, Béthune, France

###### **Correspondence:** Thibault Dekeyser (thibault.dekeyser@live.fr)

*Ann. Intensive Care* 2020, **10 (Suppl 1):**F-025

**Rationale:** Bereavement in Intensive Care Unit (ICU) is associated with psychiatric disorders on relatives called Post-Intensive Care Syndrome Family (PICS-F). No isolated intervention (such as condolence letter) has shown a positive effect on these disorders, despite a well acceptance by relatives. We thought that a more integrated bereavement program should be considered. The goal of this study is to evaluate a combined psychologist-physician post-death meeting (PDM) in a bereavement program to evaluate needs and adhesion of relatives, and the effect on symptoms of anxiety and depression.

**Patients and methods:** Monocentric, prospective study focused on relatives of patient admitted > 48 h and deceased in ICU. During patient’s stay, relatives’ presence was allowed on a 24 h-basis and they could meet a clinician psychologist. Formal meeting between relatives and the staff was realized at patient’s admission and after important decision-making treatment. Two weeks after patient’s death, the psychologist called relatives to offer emotional support and to invite to a PDM. PDM occurs 3 weeks after patient’s death with the psychologist and the physician in charge of the patient. The objectives of the meeting were to provide emotional support, to answer medical question, and to detect symptoms of anxiety and/or depression with the Hospital Anxiety and Depression Scale (HADS). We hypothesized that PMD would be able to alleviate PICS-F at 3 months. We aimed to enroll 70 families to detect a 20% lowering of HADS.

**Results:** The rate of PDM acceptance was lower than expected. After 53 inclusions, only 12 relatives accepted the PDM, whereas the phone call was well perceived (86%). Main association with acceptance of PMD was a short duration of ICU stay (4.3 days [2–5.3] vs 7.3 days [3.7–10.9] P = 0.027) and ICU admission for acute respiratory failure (66.7% vs 19.5%, P = 0.003) (Table 1). We found no relation between the number of in ICU meeting (psychologist of medical staff) and PMD acceptance. For relatives who accept PMD we found a high proportion of symptoms of anxiety and depression (92% and 83%) with a HADS at 28.5 [22–34.5] (Median, IQR). No evaluation was performed at 3 months.

**Conclusion:** Post death contact appears well perceived by relatives but PMD quite useless. This result may be explained by the inclusion of only late death (> 48 h) where psychologist and medical staff had the opportunity to support relatives. Further study should focus on early death (< 48 h).

**Compliance with ethics regulations**: Yes.

**Table 1:**
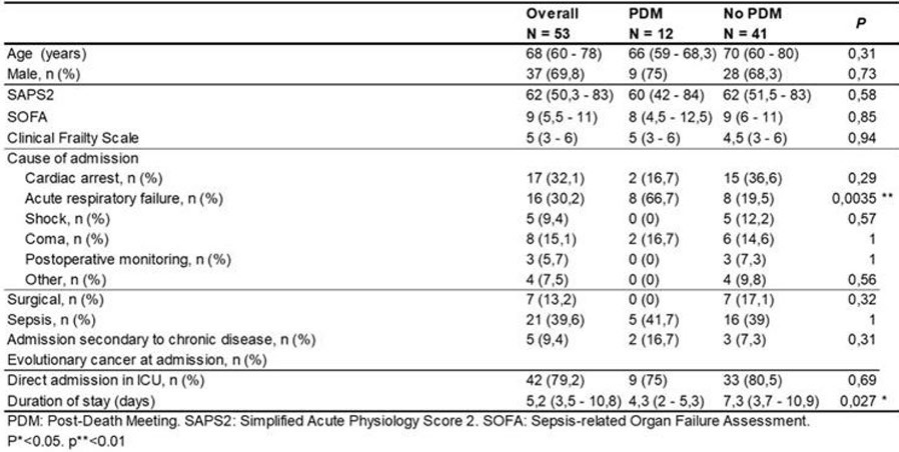
Demographic admission data of the overall study population and subset with post-death meeting acceptance or not

### F-026 The pediatric intensivist’s position when confronted to parents opposed to termination of life-sustaining treatments on their child

#### Delphine Brown, Marcel-Louis Viallard, Laure de Saint-Blanquat, Agathe Béranger, Lucie Derauglaudre, Marion Gregnon, Sylvain Renolleau, Marie-France Mamzer, Laurent Dupic

##### Necker, Paris, France

###### **Correspondence:** Delphine Brown (delphine.brown@aphp.fr)

*Ann. Intensive Care* 2020, **10 (Suppl 1):**F-026

**Rationale:** Pediatric intensivists frequently question themselves on the issue of limitation or termination of life-sustaining treatments (LLST) carried out on children. Such a decision comes under the Claeys-Leonetti Law which forbids doctors from applying unreasonable treatment However, every so often, parents oppose themselves to a collegial LLST decision that the medical and paramedical team had taken. Such cases can even end up in Court. In order to sort out this problem, this study focused on the factors that underlie the disagreement and the solution brought forward by pediatricians whenever parents demand to persue treatments although considered as unreasonable obstinacy.

**Patients and methods:** We carried out a qualitative study involving three multipurpose pediatric critical care unit. All pediatricians operating within these units were contacted. Those who volonteered were met individually for a semi-directed interview. Every interview was recorded and entitled to a complete hand-written retranscription. The interviews were analysed following the phenomenological interpretive analysis method and were subject to dual listing.

**Results:** 16 pediatricians out of 30 took part in the study. 8/16 claimed they would increase treatments or carry out cardiopulmonary resuscitation acts if asked to do so by parents, even if this went against the initial collegial decision. 8/16 claimed they would persue treatments although not beyond the current level. 2/16 said they would oppose themselves to parents concerning blood transfusion for comfort reasons. Several key factors were identified as leading a doctor to the non-application of a LLST decision: the certainty regarding the child’s death on a short or mid-term basis (16/16), the litigiousness risk (14/16), the apprehension of mediatic pressure (14/16), the fear of a violent reaction from parents (13/16), other self-interest positions within the medical team (12/16), empathy towards parents (11/16), the uncertainty concerning the neurological prognosis (5/16), the lapse of time needed to fully accept the application in force of a decision (5/16). 12 pediatricians out of 16 admitted their own-suffering when confronted to the situation.

**Conclusion:** This study points out that pediatricians tend to follow parents’ position when confronted to parental opposition. In such situations, pediatricians go against their own decision in order to safeguard the parental alliance even if it leads to unreasonable obstinacy, thus conflicting with medical deontological Code obligations.

**Compliance with ethics regulations:** Yes.

### F-027 Advance directives: a general overview

#### Ameni Abidi, Hela Maamouri, Meriem Fatnassi, Takoua Khzouri, Nozha Brahmi

##### Service de réanimation toxicologique et polyvalente, Centre Mahmoud Yaacoub d’assistance médicale urgente, Tunis, Tunisia

###### **Correspondence:** Ameni Abidi (drabidiameni@gmail.com)

*Ann. Intensive Care* 2020, **10 (Suppl 1):**F-027

**Rationale:** End-of-life management strategies are clearly a worldwide issue of major importance that intensivists have to deal with on a daily basis. Advance directives may be the solution sought to guide physicians to take such difficult decisions. Yet, health care directives are not legislated in Tunisia. The objective of this project was to draw a general descriptive overview to assess patients’ wishes in Tunisia.

**Patients and methods:** Data were collected from a 39-item-questionnaire based on the French Intensive Care Society’s form for advance directives which was filled by 101 people of general population in Tunisia, including doctors and paramedics, from May to mid-September 2019. All people included were 18 or older and well informed of the form’s utility.

**Results:** A total of 101 participants were included. The mean age was 34.6 ± 13.5 years with extremes of 18 and 76 and a sex ratio of 1.7. Fourty-one (36.6%) were either doctors or nurses and 3 (3%) did suffer from a severe medical condition. Among all the participants, 30 (29.7%) thought that end-of-life decisions were up to the doctor. For the rest, they willingly chose to be hospitalized in an ICU, to undergo cardiopulmonary rescuscitation and to have ventilation support with orotracheal intubation or tracheostomy respectively in 63 (87.5%), 60 (83.3%) and 67 (93.1%) of the cases. Only 4 (5.6%) refused temporary dialysis. When asked about sequelae they can live with, participants accepted hemiplegia in 63.9% and paraplegia in58.3% of the cases. On the contrary, 68 (94.4%) refused to live in permanent coma and 55 (79.9%) disagreed to undergo tracheostomy and ventilation for life. Moreover, 31 (43.1%) found that serious un aesthetic sequelae was a fatal consequence they could not survive. As well, only 7 (9.7%) consented to live with deep intellectual deficiency. Regarding palliative care, 53 (73.6%) participants wished to be profoundly sedated until death, 65 (90.3%) prefered to die home over 2 (2.8%) in hospital. Sixty-two (84.9%) desired to see a representative of their religion. Furthermore, 65 (89%) were for organ donnation. Gender, being a health care professional and age under 30 versus equal or over30 were not significant in dependent factors (p > 0.05).

**Conclusion:** It is our duty as health care professionals to spread advance directives awareness and education. Nevertheless, the law should keep the pace with ethics evolution.

**Compliance with ethics regulations:** Yes.

### F-028 Development of a hemodialysis model in rats with septic acute kidney injury

#### Nicolas Benichou, Stéphane Gaudry, Sandrine Placier, Juliette Hadchouel, Alexandre Hertig, Christos Chatziantoniou, Didier Dreyfuss

##### French National Institute of Health and Medical Research (INSERM), UMR_S1155, Remodeling and Repair of Renal Tissue, Hôpital Tenon, F-75020, Sorbonne Université, Paris, France

###### **Correspondence:** Nicolas Benichou (nico.benichou@gmail.com)

*Ann. Intensive Care* 2020, **10 (Suppl 1):**F-028

**Rationale:** Adapted organ support techniques are needed to enhance reliability of preclinical animal experiments in the intensive care setting (Guillon, Annals of Intensive Care-2019). A few renal replacement therapy (RRT) models have already been developed in rats, mostly hemodialysis in chronic kidney disease models or hemofiltration techniques in sepsis experiments. Mounting evidence from clinical (Gaudry, NEJM-2016) and histopathological studies suggest that RRT for acute kidney injury (AKI) could impair renal recovery by acting as a ‘second hit’ leading to a maladaptive repair of tubular epithelium. We aimed to study this hypothesis in a hemodialysis model in rats with septic AKI.

**Patients and methods:** On day 1, Sprague–Dawley rats were injected with lipopolysaccharide or placebo (NaCl 0.9%) intraperitoneally. On day 2, anesthetized rats underwent femoral artery catheterization for hemodynamic parameters monitoring. At the same time, one femoral vein and one carotid artery were catheterized for arterio-venous sterile extracorporeal circulation with or without passing through a miniature sterile Polyester Sulfone hemodialyzer (20 cm2 surface, 50 kDa pores, MicroKros^®^) filled with dialyzate liquid in the outer compartment (Table 1). Vessels were ligated after the procedure and rats allowed to awaken. On day 3, rats were sacrificed.

**Results:** All rats injected with lipopolysaccharides O127:B8 10 mg/kg survived at day 2. Anesthesia was much challenging: Ketamine + Xylazine and Tiletamine-Zolazepam + Xylazine required induction and maintenance intraperitoneal injections. These medications induced important hemodynamic parameters fluctuations and high mortality. Isoflurane gas inhalation enabled better stability, less hypothermia and quick awakening. Adequate temperature was controlled with a heating pad during the procedure and an incubator after. Supine position was maintained. The whole circuit was anticoagulated with 1 ml of heparinized saline 100 UI/ml, since clots occurred in the absence of anticoagulation and bleeding when higher dosing was used. Circuit (< 1.5 ml including dialyzer) was filled with saline solution before initiation, and total restitution of blood at the end of the experiment prevented any blood transfusion requirement. Hematocrit was determined at beginning (50%) and end of experiment (40%). A peristaltic pump provided a blood flow rate of 1.0 mL/min, (higher rate was not tolerated) for 2 h. Of note, rats who underwent sham procedure (vessels ligature only) survived and did not display AKI. Circulation of a counterflow dialysate in the dialyzer is planned but has not been performed yet.

**Conclusion:** This hemodialysis system for rats is feasible at a reasonable price and might help research involving RRT in either CKD or AKI.

**Compliance with ethics regulations:** Yes.

**Table 1:** Hemodialysis model in rats: characteristics

RRT type	Arterio-venous hemodialysis
Anesthesia	Isoflurane gas
Vascular access	Carotid artery, femoral vein
Anticoagulation	Heparinized saline 100 UI/ml: 1 ml at the beginning
Extracorporeal circuit volume	Approx. 0.7 ml (without dialyzer), 1.3 ml (with dialyzer)
Priming	NaCl 0.9% (no transfusion)
Hemodialyzer (membrane)	MicroKros^®^ (Spectrum Lab), surface 20 cm^2^, 50 kDa pores, 20 cm length, fiber inner diameter 0.5 mm
Blood flow	1 ml/min, controlled with a peristaltic pump
Duration	120 min

### F-029 Incidence and renal prognosis of positive antibiotic associated crystalluria in infective endocarditis patients

#### Matthieu Jamme^1^, Leopold Oliver^2^, Julien Ternacle^2^, Raphael Lepeule^3^, Sovannarith San^2^, Amina Moussafeur^2^, Antonio Fiore^4^, Nicolas Mongardon^5^, Michel Daudon^6^, Pascal Lim^2^, Emmanuel Letavernier^6^

##### ^1^Renal ICU and Kidney Transplantation, Tenon university hospital, APHP, Paris, France; ^2^Cardiology, Mondor university hospital, APHP, Creteil, France; ^3^Infectious mobile unit, Mondor university hospital, APHP, Creteil, France; ^4^Heart surgery, Mondor university hospital, APHP, Creteil, France; ^5^Surgical ICU Mondor university hospital, APHP, Creteil, France; ^6^Physiology unit, Tenon university hospital, APHP, Paris, France

###### **Correspondence:** Matthieu Jamme (mat.jamme@gmail.com)

*Ann. Intensive Care* 2020, **10 (Suppl 1):**F-029

**Rationale:** Infective endocarditis (IE) is a rare disease characterized by an high mortality. High-dose antibiotic has long been recognized to induce acute kidney injury (AKI) through various routes of injury. Next to acute interstitial nephritis, antibiotic-associated crystal (AAC) nephropathy has been increasingly acknowledged as a cause of severe AKI. We aimed at describing the prevalence of AAC of patient admitted and treated for suspected IE, identifying factors associated with positive AAC and assessing renal prognosis of positive AAC patients.

**Patients and methods:** All patients admitted consecutively for suspicion of IE to the cardiology unit of Mondor hospital (APHP, France) between 2017, June, 1st and 2018, June, 1st were included in the present study. IE was defined according to the modified Duke criteria. Urinary samples was collected during the first week of hospitalization and transferred to Tenon hospital (APHP, France) laboratory within 4 h. Outcome principal was the occurrence of AAC defined by the observation of antibiotic crystal formation with combining polarized microscopy and infrared spectroscopy. Secondary outcomes were the occurrence of AKI stage ≥ 2 according the KDIGO classification and the estimated glomerular filtration rate (GFR) estimating by MDRD equation at day-14.

**Results:** 34 patients were included in the analysis. Most of them were men (71%) and the median age was 70 [62–77] years. Pathogen was identified in 82% of patients. The most frequently identified pathogen was *Streptococcus*. Modified DUKE criterias defined IE as definite in 21 (64%), possible in 4 (12%) and suspected in 9 (24%) cases. First line antibiotherapy was composed by Amoxicilline (94%), Gentamycine (87%) and Cefazoline (37%). 65 crystallurias were performed within a median time 5[3–6] days. AAC was observed in 18 urinary analysis from 14/34 patients. Multivariate logistic mixed effect analysis identified blood amoxicillin concentration (OR = 1.02[1.01–1.05]) and urinary pH (OR = 0.79[0.62–0.94]) as risk factors to AAC. Severe AKI occurred in 18 (53%) patients. Statistical trend was observed with the occurrence of AAC (p = 0.07). Estimated GFR was below to 60 mL/min/1.73 m^2^ at day 14 in 32% of patients. AAC was not associated with a eGFR < 60 mL/min (p = 0.10). After exclusion of patients with CKD, we observed a statistical trend with the occurrence of AAC (p = 0.08).

**Conclusion:** AAC is a common complication of patients treated by high dose of antibiotics for suspected IE. Our results suggest of potential renal short term impact of AAC.

**Compliance with ethics regulations:** Yes.

**Fig. 1:**
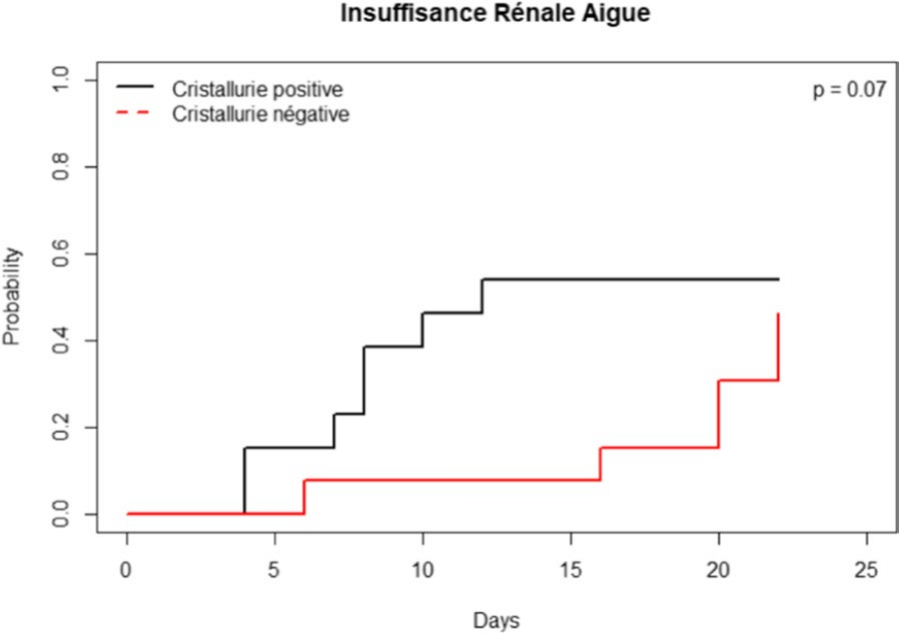
Probability of acute kidney injury in patients with (black line) and without crystalluria (red line)

### F-030 Cytogenetic Abnormalities and Risk for Acute Kidney Injury in Critically Ill Patients with Acute Myeloid Leukemia (AML)

#### Inna Mohamadou^1^, Michaël Darmon^1^, Swann Bredin^1^, Emmanuel Canet^1^, Lara Zafrani^1^, Etienne Lengline^1^, Achille Kouatchet^2^, Alexandre Demoule^3^, Pierre Perez^4^, Frédéric Pene^5^, Djamel Mokart^6^, Christine Lebert^1^, Fabrice Bruneel^7^, Emmanuel Raffoux^1^, Virginie Lemiale^1^, Elie Azoulay^1^

##### ^1^Hôpital Saint-Louis, Paris, France; ^2^CHU de Poitiers, Poitiers, France; ^3^Hôpital de la Pitié Salpêtrière, Paris, France; ^4^CHRU Nancy Brabois, Nancy, France; ^5^Hôpital Cochin, Paris, France; ^6^Institut Paoli-Calmettes, Marseille, France; ^7^Hôpital André Mignot, Versailles, France

###### **Correspondence:** Inna Mohamadou (inna.m@hotmail.fr)

*Ann. Intensive Care* 2020, **10 (Suppl 1):**F-030

**Rationale:** Acute kidney injury (AKI) is a challenging organ dysfunction in hematology patients. Indeed, optimal management relies on patient’s ability to receive the best standard of care and optimal chemotherapy regimen, which may not be possible in case of severe AKI. Moreover, in ICU patients with high tumoral burden, patients with baseline AKI are at high risk of tumor lysis syndrome (TLS), need for renal replacement therapy (RRT), delayed renal recovery and increased mortality. In patients with aggressive hematologic malignancies, risk factors for AKI have been previously identified. However, in critically ill patients with AML, the risk for AKI across different AML French-American-British (FAB) classes and on cytogenetic abnormalities has never been assessed.

**Patients and methods:** Patients were classified based on cytogenetic data according to the WHO classification into three groups (favorable, intermediate, or unfavorable). AKI was defined based on KDIGO definitions using creatinine level at ICU admission and need for RRT. A Cox proportional-hazards model was used to identify factors associated with long term survival.

**Results:** Among the 144 AML patients (age 58y [45–68], 60% men, performance status 1 [0–2]), 54 (37.5%)) were AML4 or 5, 24 (16.7%) were AML3, and 66 (45.8%) had other FAB classes. According to the WHO-cytogenetic classification, 13 (9%) patients were in the favorable group, 77 (54%) in the intermediate group, and 30 (21%) in the unfavorable group. AML3 was found in 24 (16.7%) patients. Patients were untreated in 78.5% of the cases and half of them were hyperleukocytic. SOFA score was 5 (4–9) at admission. Baseline creatinine was 88 (80–97). Based on KDIGO, 68 (47.2%) patients had no AKI, 30 (20.8%) had a stage 1, 17 (12%) a stage 2 and 29 (20.1%) a stage 3 AKI. Throughout the ICU stay, septic shock occurred in 31 (21%) patients, 23 (16%) presented TLS, 67 (46%) received vasopressors, 42 (29.2%) RRT, and 4 (31%) died in the ICU. By multivariable analysis, factors associated with AKI were male gender, poor performance status, hyperleukocytic AML, SOFA score, DIC and the WHO-cytogenetic classification (HR 2.60 [2.17–96.7] for the intermediate class, 1.98 [1.18–69.7] for the unfavorable class). FAB and WHO cytogenetic-based classifications were not significantly associated with mortality.

**Conclusion:** Half the critically ill AML patients present with AKI, a condition that is independently associated with increased mortality. Patients in the intermediate and unfavorable WHO-cytogenetic groups are at high risk of AKI, independently of leukocytosis and severity. Studies to elucidate the mechanisms pertaining AKI in these specific subgroups are warranted.

**Compliance with ethics regulations:** NA.

**Fig. 1:**
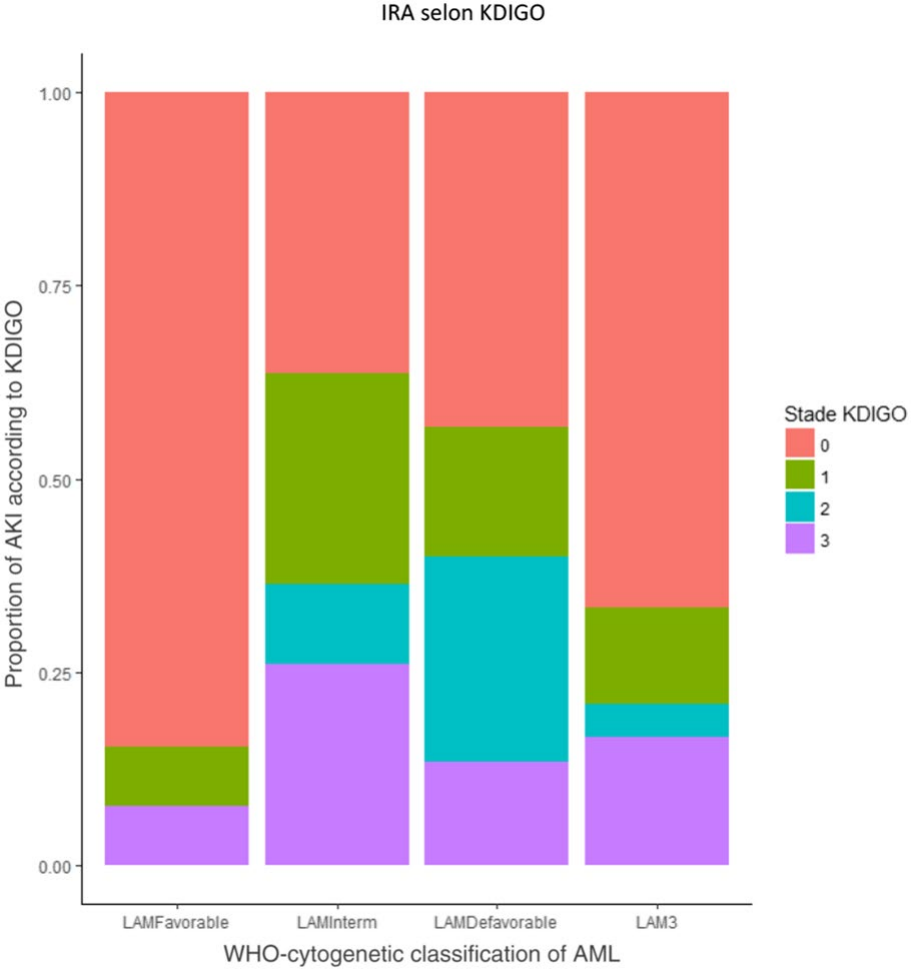
AKI according to WHO-cytogenetic classification of AML

### F-031 Do Renal Resistive Index (RRI) measurement predict renal function evolution in critically ill patients with stage 2 Acute Kidney Injury (AKI)?

#### Patricia Wiesen, Didier Ledoux, Paul Massion, Benoit Misset

##### CHU Liège-Soins Intensifs, Liege, Belgium

###### **Correspondence:** Patricia Wiesen (p.wiesen@chuliege.be)

*Ann. Intensive Care* 2020, **10 (Suppl 1):**F-031

**Rationale:** The place of Doppler-derived Renal Resistive Index (RRI) as a potential renal function predictor remains controversial because of its low specificity. Patient’s vascular compliance is known to affect this measurement. Moreover, the optimal timing for the most accurate prediction is unknown.

**Patients and methods:** This prospective observational study included 57 patients where RRI was measured when they met at least stage 2 Acute Kidney Injury (AKI) according to KDIGO criteria. Risk factors, severity score, presumed AKI aetiology (hypoperfusion, sepsis, congestion, vascular glomerular dysregulation), renal evolution, Renal Replacement Therapy (RRT) requirement, clinical and biological data were recorded. Patients were classified according to their RRI value (cut off: 0.70) for comparison.

**Results:** Initial RRI measurement was higher than 0.7 for 39 patients (68%).

There were no significant relationship between RRI and past medical history or severity score.

We observed a significant negative correlation between RRI and diastolic arterial pressure (p = 0.004) and heart rate (p = 0.004) as it could be expected by RRI formula. An increased RRI was associated with higher potassium (p = 0.019) and higher creatinine levels (p = 0.042).

Although not significant, we found a higher rate of subsequent RRT in the high RRI group (23% vs 6%, p = 0.146).

Over the first 3 days, fluid balance was significantly different between groups (2217 ml vs –1314 ml respectively for low and high RRI group, p = 0.017). Since standard of care were similar, this suggests different fluid volume status between the two groups. In the low RRI group, the cause of AKI could predominantly be prerenal since positive fluid balance was not explained by more severe AKI with refractory oliguria as shown by the low RRT rate. Nevertheless, we did not observed any relationship between RRI and the evolution of serum urea or creatinine levels, nor with the presumed aetiology of AKI.

**Conclusion:** When focussing on the first RRI measurement once stage 2 AKI was reached, RRI ≤ 0.7 seems to be in favour of prerenal and transient renal dysfunction even if this is not supported by creatinine serum evolution.

**Compliance with ethics regulations**: Yes.

**Table 1:** main characteristics of the population of the study

Clinical data		n/p (%) Median (p25; p75)	n/p (%) Median (p25; p75)	
(n = 57)	Systolic arterial pressure (mmHg)	108 (102; 130)	114 (104; 130)	P = 0.536
(n = 57)	Diastolic arterial pressure (mmHg)	63 (55; 70)	53 (47; 61)	P = 0.004
(n = 57)	Mean arterial pressure (mmHg)	79 (72; 88)	74 (65; 83)	P = 0.101
(n = 56)	Central venous pressure (mmHg)	8 (4; 10)	9 (7; 13)	P = 0.256
(n = 25)	Cardiac output	4.9 (4.0; 7.4)	4.6 (3.3; 5.5)	P = 0.379
(n = 57)	Cardiac frequency (b/min)	96 (87; 108)	80 (70; 90)	P = 0.004
(n = 57)	Vasopressor requirement	8/18 (44.4%)	18/39 (46.2%)	P = 0.904
(n = 57)	Inotropic requirement	0/18 (0%)	6/39 (15.4%)	P = 0.162
Laboratory	Haemoglobin (g/dl)	9.2 (8.3; 10.1)	9.3 (8.1; 10.6)	P = 0.718
(n = 57)	White Blood count (/mm^3^)	9905 (7850; 16,730)	12,490 (9000; 16,000)	P = 0.643
(n = 57)	CRP (mg/l)	139 (40; 263)	69 (34; 171)	P = 0.345
(n = 57)	Na (mmol/l)	144 (139; 146)	140 (138; 145)	P = 0.235
(n = 57)	Cl (mmol/l)	102 (98; 107)	102 (96; 105)	P = 0.104
(n = 57)	K (mmol/l)	3,8 (3.6; 4.2)	4.39 (3.79; 4.8)	P = 0.019
(n = 56)	PO4 (mmol/l)	1.05 (0.84; 1.37)	1.27 (1.0; 1.68)	P = 0.078
(n = 57)	Creatinine Kinase (UI/l)	64 (27; 205)	132 (49; 336)	P = 0.042
(n = 57)	Albumin (g/l)	32 (29; 35)	32 (28; 34)	P = 0.580
(n = 47)	Δ urea (mg/dl)	− 13.5 (− 21; − 2)	5 (− 37; 34))	P = 0.459
(n = 47)	Δ creatinine (mg/dl)	− 0.18 (− 0.43; − 0.09)	− 0.15 (− 0.65; 0.19)	P = 0.902
(n = 37)	Cumulated fluid balance D3 (ml)	2217 (1083; 4086)	− 1314 (− 2821; 46)	P = 0.017
(n = 57)	Diuretics	5/18 (27.8%)	12/39 (30.7%)	P = 1
Prognosis (n = 56)	RRT	1/18 (6%)	9/39 (22.5%)	P = 0.146

### F-032 Doppler based resistive index measurement in ICU patients and influence of inter-operator variability: Results of a multicenter cohort study

#### Jean Jacques Tudesq^1^, David Schnell^2^, Marie Reynaud^3^, Stéphane Rouleau^2^, Ferhat Meziani^4^, Alexandra Boivin^4^, Mourad Benyamina^5^, Francois Vincent^6^, Alexandre Lautrette^7^, Christophe Leroy^7^, Yves Cohen^8^, Matthieu Legrand^5^, Jerome Morel^3^, Jeremy Terreaux^3^, Aurelie Bourmaud^9^, Michaël Darmon^5^

##### ^1^Saint-Louis University Hospital, Paris, France; ^2^Angouleme Hospital, Angouleme, France; ^3^Saint-Etienne Hospital, Saint-Etienne, France; ^4^Strasbourg Hospital, Strasbourg, France; ^5^Saint-Louis Hospital, Paris, France; ^6^Montfermeil Hospital, Montfermeil, France; ^7^Clermont-Ferrand Hospital, Clermont-Ferrand, France; ^8^Avicenne Hospital, Bobigny, France; ^9^Robert Debre Hospital, Paris, France

###### **Correspondence:** Jean Jacques Tudesq (jean-jacques.tudesq@aphp.fr)

*Ann. Intensive Care* 2020, **10 (Suppl 1):**F-032

**Rationale:** Clinical data regarding factors that may influence Doppler-based resistive index (RI) at bedside are limited. Moreover, influence of operator has poorly been assessed. This study aimed at evaluating clinical characteristics associated with RI at bedside and to delineate influence of inter-operator variability as this regard.

**Patients and methods:** Post-hoc analysis of a multicentre prospectively collected dataset. Adult patients requiring mechanical ventilation were included. Patients with severe chronic renal dysfunction or known renal artery stenosis were excluded. AKI was defined according to KDIGO definition. Renal Doppler was performed at study inclusion. Operators involved in this study were anonymised. Results are reported in n (%) or median (IQR). Adjusted factors associated with AKI development were assessed using mixed linear regression model with the operator as random effect on the intercept.

**Results:** Overall, 351 patients were included in this study, including 149 patients with AKI stage 1 (42.5%), 33 patients with AKI stage 2 (9.0%) and 51 patients with AKI stage 3 (14.5%). Median age was 62 years (IQR 51–70), and 129 were of female gender (36.8%). Vasopressors was required in 184 patients (52.5%). RI was associated with AKI severity, with a RI of 0.65 (0.59–0.70) in patients without AKI and increasing steadily to 0.72 (0.62–0.78) in patients with AKI stage 3. After adjustment for confounders, factors independently associated with RI were age (estimate per year: 0.001, sd: 0.0003; p < 0.001), case mix (emergency surgery estimate (vs. medical): − 0.06, sd:0.03; p = 0.03), hypovolemia at ICU admission (estimate: 0.03, sd:0.02; p = 0.02), underlying cardiac comorbidity (estimate: 0.05, sd:0.02; p = 0.002), use of norepinephrine (estimate: 0.03, sd:0.0; p = 0.007), and AKI stage 3 (vs. no AKI) (estimate: 0.04, sd:0.02; p = 0.02). When forced in the final model, mean arterial pressure, pulsed pressure, plateau pressure, heart rate, sepsis were not selected. Operator was found to influence significantly RI in the mixed model (Fig. 1a; mean OR 0.03 [0.01–0.05]; p < 0.001). To further depict the influence of inter-operator variability, adjusted changes in RI according to age (1a) and AKI KDIGO stage (1b) are reported for each operator (each line depicting an operator).

**Conclusion:** Our study suggests that Doppler-based RI in critically ill patients is influenced by patients’ case-mix, age, underlying dehydration or shock, cardiac comorbidities, and AKI severity. Our study confirms and illustrates a strong inter-operator variability of RI measurement in ICU that persists after adjustment. Whether this inter-operator variability impairs RI reliability and usefulness at bedside deserve to be more carefully assessed.

**Compliance with ethics regulations:** Yes.

**Fig. 1:**
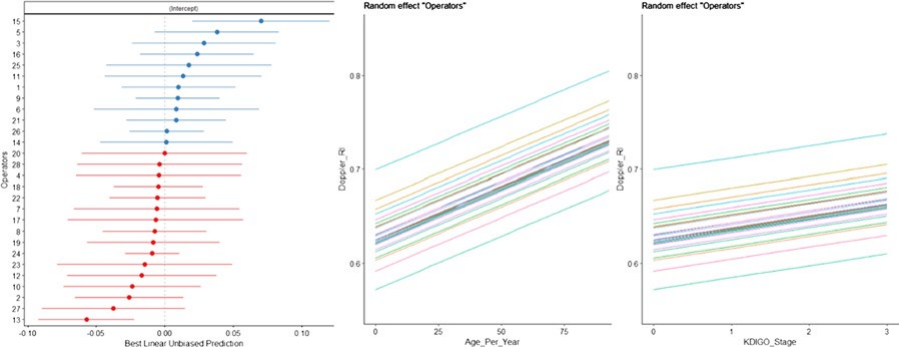
Reporting change in RI according to operators after adjustment for confounders (one line per operator)

### F-033 Echocardiographic follow-up of right ventricular dysfunction is the best way to evaluate cardiorenal syndrome (CRS) in ICU

#### Mario Geneix^1^, Sebastien Moschietto^2^, Antoine Frouin^2^, Fanny Depeyre^2^, Thibault Dupont^3^, Florent Montini^2^

##### ^1^CH Timone, Marseille, France; ^2^CH Henri Duffaut, Avignon, France; ^3^CHU Kremlin Bicêtre, Paris, France

###### **Correspondence:** Mario Geneix (mario.geneix@gmail.com)

*Ann. Intensive Care* 2020, **10 (Suppl 1):**F-033

**Rationale:** Type 1 Cardiorenal syndrome (CRS) is defined by acute decompensated heart failure (AHF) leading to secondary acute kidney injury (AKI) due to persistent congestion. Diagnosis is often difficult and relies on compatible clinical history, symptoms and biology. There are currently no studies evaluating the reliability of transthoracic echocardiography (TTE) as a diagnostic tool in CRS. Therefore, the aim of this study was to assess quantitative and qualitative echocardiographic parameters in patients with CRS in the ICU at baseline and after appropriate care (pharmacologic therapy, with or without additional ultrafiltration).

**Patients and methods:** We conducted an observational, prospective, single-center study in the ICU department of a general hospital. Patients admitted in the ICU and presenting with type 1 CRS were included. Diagnosis of CRS was made based on clinical context, biology and confirmed by two attending physicians (nephrologist and cardiologist). Patients presenting with other causes of AKI were excluded. Transthoracic echocardiography was performed at baseline and at day 7 after treatment by the same trained operator for the same patients. We report various echocardiographic indices of right and left ventricular function, filling pattern and venous pressure at these two timepoints.

**Results:** A total of 27 patients were included in this study. At baseline (D0), 96.3% of patients had signs of congestion (IVC dilation > 2 cm), 76% had an altered S-wave (< 11.5 cm/s), 72.73% had an altered TAPSE (< 15 mm), 85.19% had an elevated RV/LV surface ratio (> 0.6). Between baseline and D7, under appropriate management, IVC size significantly decreased ([− 1.8; − 0.3] p < 0.001), the number of patients with an elevated RV/LV diameter ratio (> 0.6) also decreased (OR [IC 95%] = 0.087 [0.02; 0.37] p < 0.001), weight decreased (4.1 kg (5.24) p < 0.001), whereas natriuresis significantly increased (47.11 mM/ml (45.55) p < 0.001), and the amount of vasopressors support decreased (− 0.04 ug/kg/min [− 5.9; 0.46] p < 0.001). Other parameters including creatinine level (− 14.33 uM/L [− 42.02; 13.35]), cardiac index (+ 0.35 L/min/m^2^ [− 0.05; 0.73]), and S-wave velocity (0.32 cm (2.67)) showed non-significant changes.

**Conclusion:** Main echocardiographic findings at baseline in patients with type 1 CRS consist of a right ventricular dysfunction associated with a state of increased venous pressure and congestion in the form of an IVC dilation. We report that weight, RV/LV diameter ratio, and IVC diameter might constitute good follow-up parameters to monitor treatment response.

**Compliance with ethics regulations:** Yes.

**Fig. 1:**
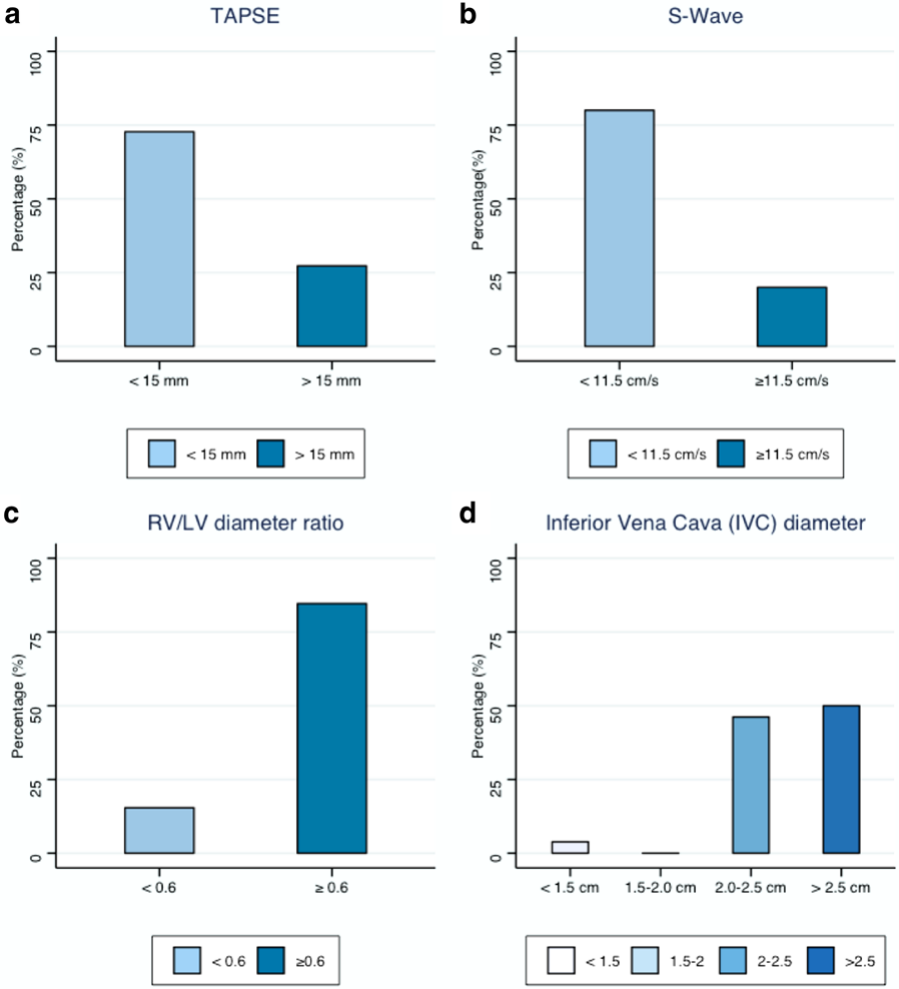
Echocardiographic right ventricular parameters at baseline

### F-034 Is citrate required for heparin-free intermittent hemodialysis in critically ill patients?

#### Chloé Medrano, Olivier Cointault, Laurence Lavayssiere, Marie-Béatrice Nogier, Nassim Kamar, Stanislas Faguer

##### Département de Néphrologie et transplantation d’organes, Toulouse, France

###### **Correspondence:** Chloé Medrano (chloemedrano@hotmail.fr)

*Ann. Intensive Care* 2020, **10 (Suppl 1):**F-034

**Rationale:** Critically ill patients are at higher risk of bleeding but also dialysis filter clotting (inflammatory state). Intermittent hemodialysis with calcium-free citrate-containing (0.8 mmol/L) dialysate (CafCit-IHD) recently emerged as a new safe and simple alternative to continuous renal replacement therapy allowing heparin-free extended dialysis sessions (> 5 h). In this study, we aimed to answer to two issues still unresolved: (i) can citrate contained in the dialysate accumulate and lead to citrate intoxication in patients with liver disorders, and (ii) can citrate be avoided using citrate- and calcium-free dialysate (CCF-IHD)?

**Patients and methods:** Monocentric retrospective study. Among the 450 sessions performed with CafCit-IHD, the 20 IHD sessions (18 critically ill patients) with citrate measurement available before and after the dialysis filter were reviewed. Estimation of the liver clearance was performed using the Picco Lemon^®^ system (Pulsion). In addition, 8 sessions performed using CCF-IHD were reviewed.

**Results:** All the patients had liver disorders (post-liver transplantation period n = 2; cirrhosis with Child > A6). Among the eighteen CafCit-IHD patients, fifteen (75%) and six (30%) received mechanical ventilation or vasopressive drugs, respectively. The median time of the dialysis session was 5 h [2–5], with hourly ultrafiltration rate of 400 mL (one premature termination not related to dysfunctional catheter). In all patients, ionized calcium (iCa) decreased below 0.40 mmol/L after the filter, whereas post-filter calcium reinjection according to ionic dialysance led to a stable pre-filter (i.e. patient) iCa. Median citrate concentrations were all below 0.8 mmol/L after the filter (minimal concentration to obtain anticoagulation 3 mmol/L) and all except one below the normal value (< 125 µmol/L) before the filter. During all the sessions, ionized to total calcium ratio was below 2.1 and the strong ionized gap decreased. When available (n = 7), no correlation could be identified between serum citrate concentration and liver clearance. Last, in 8 CCF-IHD sessions performed in critically ill patients, no premature termination occurred (median time of the sessions 5 h) and post-filter iCa also decreased below 0.45 mmol/L.

**Conclusion:** No citrate accumulation could be identified in critically ill patients (even with liver disorders) and receiving extended dialysis sessions (5 h or more) using calcium-free citrate containing-IHD. Interestingly, we demonstrated that citrate is not required to obtain optimal regional anticoagulation (i.e. post-filter iCa < 0.45 mmol/L), and a citrate- and calcium-free dialysate could be a safe alternative.

**Compliance with ethics regulations:** Yes.

### F-035 Assessment of diaphragmatic function in mechanically ventilated children using the neuromuscular efficiency index

#### Benjamin Crulli, Guillaume Emeriaud

##### Pediatric intensive care unit, Department of pediatrics, CHU Sainte Justine, Université de Montréal, Montreal, Canada

###### **Correspondence:** Benjamin Crulli (benjamin.crulli@umontreal.ca)

*Ann. Intensive Care* 2020, **10 (Suppl 1):**F-035

**Rationale:** Ventilator induced diaphragmatic dysfunction is highly prevalent in adult critical care and associated with worse outcomes. Specificities in pediatric respiratory physiology suggest that critically ill children may be at high risk of developing this complication, but no study has described the evolution of diaphragmatic function in critically ill children undergoing mechanical ventilation. This study aims to validate a method to quantify diaphragmatic function in mechanically ventilated children.

**Patients and methods:** In this prospective single-center observational study, 10 children between 1 week and 18 years old intubated for elective ENT surgery and without pre-existing neuromuscular disease or recent muscle paralysis were recruited. Immediately after intubation, diaphragmatic function was evaluated using brief airway occlusion maneuvers during which airway pressure at the endotracheal tube (Paw) and electrical activity of the diaphragm (EAdi) were simultaneously measured for 5 consecutive spontaneous breaths, while the endotracheal tube was occluded with a specific valve. Occlusion maneuvers were repeated 3 times. In order to account for central respiratory drive and sedation use, we recorded the neuromechanical efficiency ratio (NME, Paw/EAdi), in addition to the maximal inspiratory force (MIF). In order to determine the optimal measure of NME during an occlusion, the variability over the three occlusion maneuvers of different variables (first breath, last breath, breath with maximal Paw deflection, breath with maximal NME value, and median NME value) was assessed using coefficients of variation and repeatability coefficients.

**Results:** Patients had a median age of 4.9 years (interquartile range 3.9–5.5), a median weight of 18 kg (14–23), and 5 were male (50%). The median evolution of Paw, EAdi, and NME ratio over the 5 occluded breaths are represented on Fig. 1. NME values corresponding to the last breath and the breath with maximal Paw deflection were the least variable, with median coefficient of variation of 23% and 23% and repeatability coefficients of 3.44 and 3.44, respectively.

**Conclusion:** Brief airway occlusions can be used to assess diaphragmatic function in intubated children through both MIF and NME ratio, and the latter should ideally be computed on the last breath or the breath with the largest pressure deflection to improve repeatability and decrease variation.

**Compliance with ethics regulations:** Yes.

**Fig. 1:**
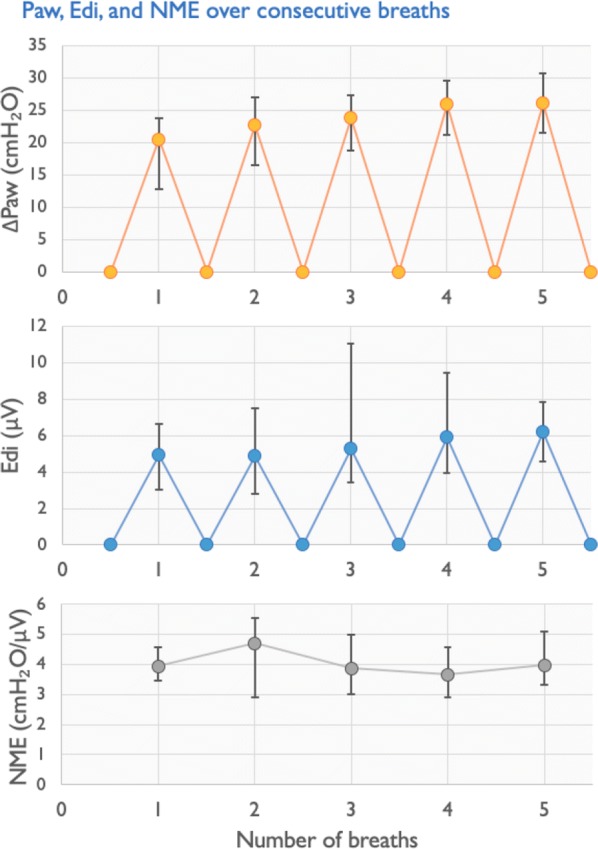
Median (interquartile) evolution of Paw, eadi, and NME ratio over the 5 breaths of occlusion maneuvers

### F-036 Efficacy and safety of dexmedetomidine as sole sedation for children undergoing MRI in comparison to general anesthesia: a single-center retrospective study (DEX-IRM)

#### Hélène Lepeltier^1^, Arnaud Lepetit^2^, Maxime Gauberti^3^, Clément Escalard^3^, Anne Lesage^2^, David Brossier^4^, Isabelle Goyer^5^

##### ^1^CHU CAEN, Département de pédiatrie, Caen, France; ^2^CHU CAEN, Département d’anesthésie, Caen, France; ^3^CHU CAEN, Département de radiologie, Caen, France; ^4^CHU CAEN, Département de réanimation pédiatrique, Caen, France; ^5^CHU CAEN, Département de pharmacie, Caen, France

###### **Correspondence:** Hélène Lepeltier (helene.lepeltier@orange.fr)

*Ann. Intensive Care* 2020, **10 (Suppl 1):**F-036

**Rationale:** MRI requires prolonged motion-free periods. This may be difficult to obtain in the pediatric patient population. This need for prolonged immobility justifies the use of general anesthesia or procedural sedation in children. Recently, national drug safety agencies have warned physicians about the risk of neurotoxicity of conventional anesthetic agents targeting GABAergic and glutamatergic neurotransmission pathways in children. Dexmedetomidine, a sedative agent with works as an α2 pre-synaptic receptor agonist, does not act on these usual pathways. This molecule has raised great interest in the procedural sedation of children, especially since it preserves respiratory drive. The objective of this study was to evaluate the efficacy and safety of dexmedetomidine sedation in pediatric MRI compared to conventional general halogenated anesthesia.

**Patients and methods:** This was a retrospective monocentric cohort study performed in the University Hospital of Caen. This study included all patients under 18 years of age who received sedation for MRI between August 1st, 2018 and March 31st, 2019. Patients were retrospectively divided into 2 groups according to the performed sedation modality (DEX and GA).

**Results:** 78 patients were included (26 in the DEX group and 52 in the AG group). Dexmedetomidine sedation was significantly associated with a decrease in the use of invasive ventilation (p < 0.001) with no difference in image quality. The failure rate of sedation was 42% in the DEX group vs. 0% in the AG group (p < 0.001). None of the patients had any significant adverse reactions to dexmedetomidine.

**Conclusion:** Dexmedetomidine seems suitable for procedural sedation during MRI in children. It provides an alternative to halogenated general anaesthesia with the aim of limiting children’s exposure to conventional anaesthetic agents and the use of invasive ventilation.

**Compliance with ethics regulations:** Yes.

### F-037 Incidence of withdrawal syndrome after sedation/analgesia in a surgical pediatric intensive care units using the Withdrawal Assessment Tool-1 (WAT-1) score

#### Pauline Ponsin^1^, Guillaume Geslain^2^, Chloé Tridon^1^, Charline Riaud^1^, Nicolas Robin^1^, Alina Lazarescu^1^, Gilles Orliaguet^1^

##### ^1^Department of anaesthesia and intensive care, Assistance Publique-Hôpitaux de Paris, Necker-Enfants Malades Hospital, Paris, France; ^2^Pediatric Intensive Care Unit, Assistance Publique-Hôpitaux de Paris, Robert Debre University Hospital Université de Paris, Paris, France

###### **Correspondence:** Pauline Ponsin (pauline_2014@hotmail.com)

*Ann. Intensive Care* 2020, **10 (Suppl 1):**F-037

**Rationale:** Withdrawal syndrome (WS) is a known side effect of prolonged sedation/analgesia in pediatric intensive care units (PICU). Epidemiology is poorly understood due to the rare use of validated diagnostic tools. The main objective of the study was to determine, by systematically calculating the WAT-1 score, the incidence of WS in our surgical PICU. The secondary objective was to analyze the risk factors, consequences and management modalities of WS.

**Patients and methods:** Following Institutional Review Board approval, we conducted a prospective monocentric study between July 2018 and January 2019. All consecutive mechanically ventilated children admitted in our surgical PICU with sedation/analgesia by continuous intra-venous (IV) benzodiazepines (BZD) and/or opioids for at least 48 h were included. As soon as sedation was decreased and during 72 h following their total discontinuation, WAT-1 score was assessed twice a day. WS was defined by a WAT-1 score > 3. The search for risk factors and consequences associated with WS was performed by univariate analysis (Mann–Whitney and Chi2 test). Ethical standards were satisfied and the lack of opposition from patients and their parents was systematically checked.

**Results:** The incidence of WS was 50% among the 46 patients of our cohort including 54% of children admitted postoperatively and 35% after severe traumatic brain injury (TBI). Significant results are reported in Table 1. Our results show that even for sedation time less than 5 days, children could develop WS (11/23 patients). On the other hand, age, severity (PELOD 2 score), number of previous surgeries and severe TBI were not associated with WS. Our study also demonstrated that cessation of sedation and prevention of WS was not uniform in our unit.

**Conclusion:** The high incidence of withdrawal syndrome in our study, even in children sedated for less than 5 days, and its consequences require thinking about prevention. We suggest a systematic monitoring of the occurrence of this adverse event using a validated score, from 3 days of continuous IV sedation/analgesia.

**Compliance with ethics regulations:** Yes.

**Table 1:** Risk factors and consequences of withdrawal syndrome

	WAT-1 ≥ 3 (n = 23)	WAT-1 < 3 (n = 23)	p
Duration of opioids (d)	6.0 (4.9; 5.0)	4.0 (3.0; 5.5)	0.006
Total dose of opioids (µg/kg equivalent sufentanil)	41.7 (30.9; 127.6)	17.25 (132.0; 35.9)	< 0.001
Duration of BZD infusion (d)	5.0 (5.0; 9.0)	4.0 (3.0; 5.5)	0.006
Total dose of BZD (mg/kg equivalent midazolam)	33.2 (20.8; 58.9)	14.0 (5.6; 20.4)	< 0.001
Length of mechanical ventilation (d)	9.0 (6.5; 13.0)	5.0 (4.0; 7.0)	< 0.001
Length of stay in the PICU (d)	13.0 (10.0; 23.0)	9.0 (5.0; 10.5)	< 0.001
Data presented as median (range). BZD: benzodiazepine, d: days, PICU: pediatric intensive care unit. A p value < 0.05 was considered statistically significant.			

### F-038 Automatic Real-time Classification of the Validity of Intracranial Pressure Signals Recorded by Ventricular Drain Using a Machine Learning Method

#### Sally Al Omar^1^, Floriane Cannet^2^, Gabriel Masson^3^, Philippe Jouvet^1^, Guillaume Emeriaud^1^

##### ^1^CHU Sainte-Justine, Montreal, Canada; ^2^Polytech Marseille, Marseille, France; ^3^University Hospital of Lille, Lille, France

###### **Correspondence:** Sally Al Omar (sally.alomar@gmail.com)

*Ann. Intensive Care* 2020, **10 (Suppl 1):**F-038

**Rationale:** Severe traumatic brain injury (TBI) is a major healthcare problem. Amplitude and duration of intracranial hypertension is highly associated with patient outcome. The intracranial pressure (ICP) is therefore one key parameter to monitor in the acute phase. When ICP is monitored with an external ventricular drain, the pressure recorded by the monitor does not always correspond to the real ICP, depending on the status (open/closed) of the 3-way tap. Misleading values could therefore be sent to the patient medical record. Our hypothesis is that a machine-learning algorithm will be able to identify automatically and in real time the reliable and non-reliable values of the ICP signal.

**Patients and methods:** We retrospectively studied pediatric patients having an external ventricular drain between July 2018 and July 2019, in a single pediatric intensive care unit. The ICP signals were extracted from a high-frequency database (128 Hz) and pre-processed adequately. To train the algorithms, an annotated database was manually created with two classes: reliable ICP vs. non-reliable ICP (drain system opened to allow cerebrospinal fluid removal). Eleven signal characteristics were compared between the two classes (Mann–Whitney test), and significantly differing variables were tested in the algorithms. We compared the performance of two machine-learning algorithms: the K-Nearest Neighbors (KNN) and the Support Vector Machine (SVM). Using 10-fold cross-validation method, 75% of the data was used to train the algorithms and 25% was used for testing. The best classifier was further validated by simulating a real-time ICP analysis, using a 15 s sliding-window approach with 50% overlap. The study was approved by the local research ethics committee.

**Results:** Sixteen patients were included in the study. The training database created from 14 patients, contained 320 segments (of 15 s duration) per class and per patient. Eight signal variables were identified and kept to define the segments. The KNN algorithm, with k = 3, led to the best performance, with a mean of 98% (mean ± SD: 98% ± 0.29%). The KNN was then visually validated on ICP signals from the remaining two patients (Figure). By simulating a real-time ICP extraction, our algorithm was able to efficiently identify the reliable ICP segments, and to display a mean value only for valid segments.

**Conclusion:** The proposed machine learning algorithm can help identifying the validity of ICP values recorded using a ventricular drain in real time. After external validation, this algorithm could be implemented in future clinical decision support system to optimize the care of TBI patients.

**Compliance with ethics regulations**: Yes.

**Fig. 1:**
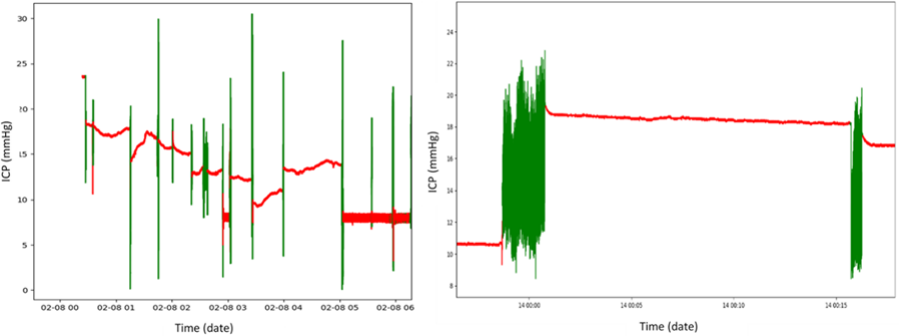
Results of real-time ICP signal classification with the KNN algorithm using the sliding-window approach. Green: reliable ICP. Red: non-reliable ICP (ventricular drain system opened for fluid removal)

### F-039 Epidemiology and prognosis of acute encephalitis in pediatric intensive care unit

#### Hélène Lienard^1^, Jérôme Naudin^1^, Florence Renaldo^2^, Fleur Le Bourgeois^1^, Anna Deho^1^, Maryline Chomton^1^, Géraldine Poncelet^2^, Guillaume Geslain^2^, Stéphane Dauger^1^, Michael Levy^1^

##### ^1^Pediatric Intensive Care Unit, Hôpital Robert-Debré, Assistance Publique Hôpitaux de Paris, Paris, France; ^2^Pediatric Neurology, Hôpital Robert-Debré, Assistance Publique Hôpitaux de Paris, Paris, France

###### **Correspondence:** Hélène Lienard (lienardhelene@gmail.com)

*Ann. Intensive Care* 2020, **10 (Suppl 1):**F-039

**Rationale:** Acute encephalitis is a rare but potentially severe disease leading to an admission in Pediatric Intensive Care Units (PICU) in 18 to 40% of pediatric patients. However, there are few epidemiological data on these severe forms and little is now about their outcome. The main objective of this study was to describe the etiologies of acute encephalitis in PICU as well as their presentations. Secondary objectives included an evaluation of the outcome (mortality and sequalae) as well as related risk factors of poor outcome.

**Patients and methods:** A monocentric retrospective study was performed between January 2005 and December 2018 in Robert-Debré University Hospital PICU (Paris). All consecutive children (1 month–18 years) admitted for acute encephalitis were included and diagnosis was confirmed using the 2013 consensus conference criteria’s. Data regarding clinical, biological and radiological presentations were collected as well as data on the therapeutics used and outcomes at discharge and at the last medical consultation.

**Results:** 106 patients were included with a mean age of 6.2 years (range 0.1 to 17 years old). Infectious causes were identified in 45% (n = 48), autoimmune causes in 8% (n = 8) and acute demyelinating encephalomyelitis in 4% (n = 4) of cases. Etiology remained undetermined in 43% of cases (n = 46). The most common pathogens were, in order of frequency, Influenzae virus, Mycoplasma pneumoniae and Epstein-bar virus. The main clinical features were fever (88% n = 93); epileptic seizures (80% n = 85) and coma (46% n = 49). Regarding therapeutics, 52% of patients required mechanical ventilation and 20% of patients required hemodynamic support. 47% received corticosteroids, 17% intravenous immunoglobulins and 12% plasmatic exchanges. The use of these specific treatments was heterogeneous, especially in infectious and undetermined encephalitis, where respectively 48% and 38% received boluses of corticoids. The mean length of stay in PICU was 10.7 days (range 1–155 days). The mortality rate was 10% and the overall rate of sequelae at discharge was 76% and 61% at distance, with 21% considered as severe (GOSE-PED score > 5). The use of mechanical ventilation and young age at diagnosis were risk factors associated with poor prognosis at discharge.

**Conclusion:** The etiology of acute encephalitis remains indeterminate in more than 40% cases with a clear predominance of infectious causes when an etiology is found. This is a severe pathology responsible for significant mortality and morbidity requiring long-term follow-up.

**Compliance with ethics regulations**: Yes.

### F-040 Prognostic value of cerebral oxymetry in critically ill children undergoing extracorporeal membrane oxygenation

#### Meryl Vedrenne-Cloquet, Raphaël Lévy, Judith Chareyre, Manoëlle Kossorotoff, Sylvain Renolleau, Marion Grimaud

##### Hôpital Necker-Enfants Malades, Paris, France

###### **Correspondence:** Meryl Vedrenne-Cloquet (meryl_vedrenne@yahoo.fr)

*Ann. Intensive Care* 2020, **10 (Suppl 1):**F-040

**Rationale:** Preserving neurological outcome of children under Extracorporeal Membrane Oxygenation (ECMO) remains challenging. Acute Brain Injury (ABI) is a frequent complication of ECMO that could be prevented by continuous neuromonitoring. Cerebral Near InfraRed Spectroscopy (NIRS) is routinely used for detecting cerebral complications of cardiac surgery. In adults and infants under prolonged ECMO, cerebral hypoxia is associated with poor neurological outcome. The aim of this study was to assess the value of an impaired cerebral oxygenation on mortality and occurrence of an ABI in children under ECMO.

**Patients and methods:** Children under 18 years old were included in this observational retrospective monocentric study if they needed veno-venous (V–V) or veno-arterial (V-A) ECMO for respiratory and/or circulatory failure and had concomittant NIRS monitoring. Cerebral desaturation was defined as a rScO_2_ value under 50% or under 20% from the baseline; cerebral hyperoxia was defined as a rScO_2_ value above 80%. Proportion of time in cerebral desaturation and hyperoxia were recorded. Neurological lesions were identified on imaging (MRI or scan) by blinded radiologist and classified as major or minor. ABI was defined as any hemorragic or ischemic lesion on cerebral imaging, including brain death.

**Results:** 63 patients were included. ECMO duration was 9 [5; 13] days. The mortality rate was 32 (50.8%), and the proportion of ABI was 34 (54%) including 5 brain deaths, 10 (15.9%) major lesions, and 19 (30.2%) minor lesions. Mean rScO_2_ was 73 ± 9% in the right hemisphere, and 75 ± 9% in the left hemisphere. There was no significant difference in cerebral hypoxia between survivors and non survivors, and between patients with and without an ABI. Cerebral hyperoxia was associated with a better survival (p = 0.03 in the right hemisphere, and p = 0.02 in the left hemisphere). In V–V ECMO and at the right hemisphere, proportion of patients in hyperoxia was higher in survivors (78 [72; 81.8]% versus 58 [56; 70]%, p = 0.04); proportion of time in hyperoxia was also more important (42 [12; 57] % in survivors versus 4.5 [0; 23.3]% in non survivors, p = 0.049).

**Conclusion:** In our study, cerebral hypoxia was not associated with poor neurological outcome, but cerebral hyperoxia seems to be protective especially in V–V ECMO. This is the first study assessing the value of cerebral oxymetry in all age ranges pediatric ECMO. In this population, multimodal monitoring might be better than NIRS alone to predict neurological impairment. Further prospective studies are needed to assess first the feasibility, then the impact of such a monitoring.

**Compliance with ethics regulations**: Yes.

### F-041 Cerebral autoregulation impairment is associated with acute neurological events during pediatric extracorporeal membrane oxygenation

#### Nicolas Joram^1^, Erta Beqiri^2^, Stefano Pezzato^3^, Pierre Bourgoin^1^, Alexis Chenouard^1^, Jean-Michel Liet^1^, Marek Czosnyka^2^, Pierre-Louis Leger^4^, Peter Smielewski^2^

##### ^1^Pediatric Intensive Care Unit, University hospital of Nantes, Nantes, France; ^2^Division of academic Neurosurgery, Department of clinical neurosciences, Addensbrooke ‘s Hospital, University of Cambridge, Cambridge, UK; ^3^Pediatric Intensive Care Unit, IRCCS Giannina Gaslini Institute, Genoa, Italy; ^4^Pediatric Intensive Care Unit, Trousseau University Hospital, Paris, France

###### **Correspondence:** Nicolas Joram (nicolas.joram@chu-nantes.fr)

*Ann. Intensive Care* 2020, **10 (Suppl 1):**F-041

**Rationale:** Children supported by extracorporeal membrane oxygenation (ECMO) present a high risk of adverse neurological complications. As some animal studies have shown, cerebral autoregulation (CA) impairment after exposure to ECMO, may be a key factor. Our main objective was to investigate the feasibility of CA continuous monitoring during ECMO treatment. The second objective was to analyze the relationship between CA impairment and neurological outcome.

**Patients and methods:** An observational prospective study including children treated by ECMO in 2 centers was conducted. A correlation coefficient between the variations of regional cerebral oxygen saturation (rScO_2_) and the variations of mean arterial blood pressure (MAP) was calculated as an index of CA (cerebral oxygenation reactivity index, COx) during ECMO. A COx > 0.3 was considered as indicative for dysautoregulation. COx values were averaged inside 2 mmHg-MAP bins, allowing determining optimal MAP (MAPopt) and lower (LLA) and upper (ULA) limits of autoregulation in 8-h periods. Neurological outcome was assessed by the onset of an acute neurologic event (ANE) defined by occurrence of hemorrhagic or ischemic stroke and/or clinical or electrical seizure and/or brain death during the ECMO treatment.

**Results:** Twenty-nine patients (31 ECMO runs) treated by veno-arterial (VA, n = 23) or veno-venous ECMO (VV, n = 6) were included (median age 71 days, weight 4.6 kg). COx was always available in all patients and MAPopt, LLA and ULA in 89.8% of time. CA variables were similar during VA and VV ECMO runs. Among children who presented ANE (15/29, 51.8%) versus others, the median time spent with a COx > 0.3 was significantly higher (33% (23.5–64.2) vs 20% (16.6–23.7), p < 0.001). These patients spent also significantly more time with MAP below LLA (10.4% (6.1–26.8) vs 5.2% (1.8–9.2), p = 0.03). After adjustment on the onset of cardiac arrest before or during cannulation and the type of ECMO (VA vs VV), percentage of time spent with a Cox > 0.3 higher than 25% was independently associated with ANE (aOR 8.03, IC 95% 1.02–63.12, p = 0.04).

**Conclusion:** CA assessment seems to be feasible in pediatric ECMO. The impact of the time below autoregulation threshold on neurological outcome is significant. However, the underlying mechanisms of CA impairment during ECMO need to be explored further.

**Compliance with ethics regulations:** Yes.

**Fig. 3:**
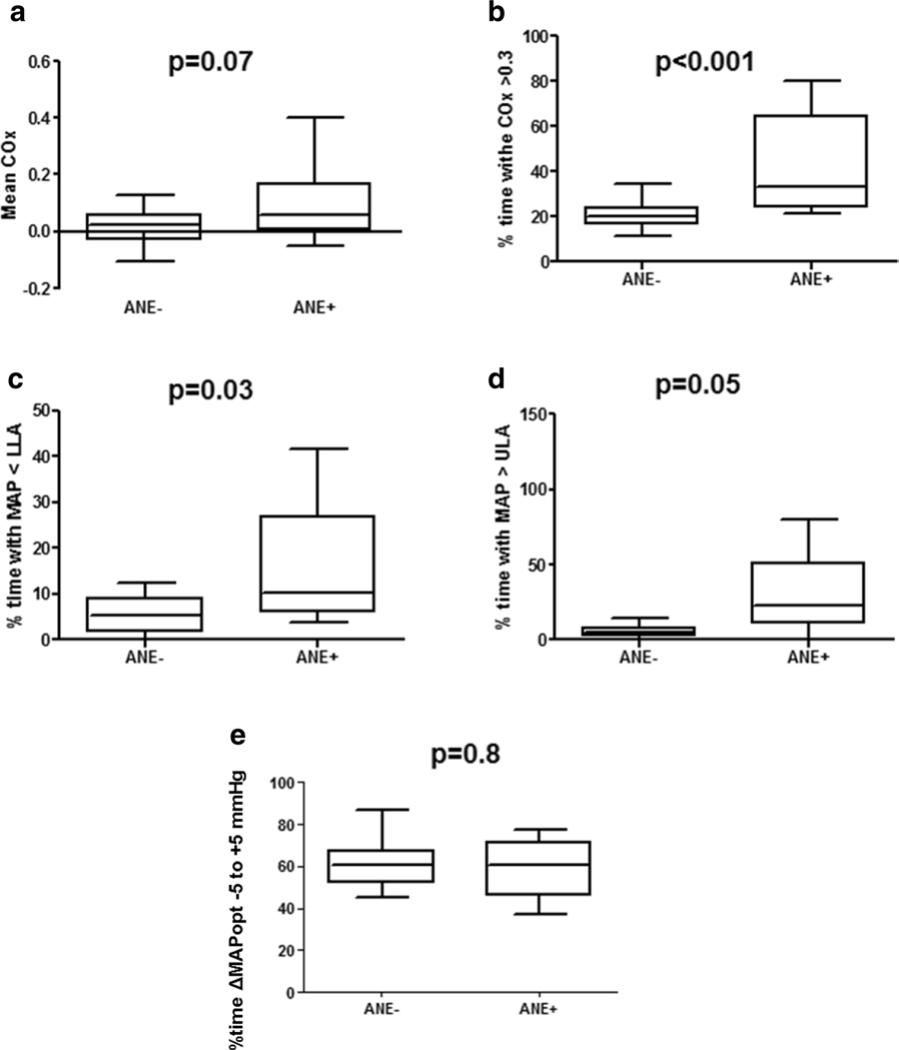
Autoregulation parameters according to neurological outcome. A. Mean COx. B. Percentage of time with COx > 0.3. C. Percentage of time with MAP < LLA. D. Percentage of time with MAP > ULA. E. Percentage of time with MAP—MAPopt between − 5 and + 5 mmHg. AU, Area Under; MAP, Mean Arterial Pressure; LLA, Lower Limit of Autoregulation; ULA, Upper Limit of Autoregulation; ΔMAPopt = median MAP-MAPopt. Data are shown in box-and-whisker plots, indicating the median, interquartile range, and range. The Mann–Whitney test was used to compare the 2 groups of infants

### F-042 Protective mechanisms of pharmacologic preconditioning against myocardial ischemia reperfusion injury: role of Bcl-2 family proteins

#### Romain Rozier^1^, Rachel Paul-Bellon^1^, Elodie Villa^2^, Amélie Rolle^3^, Marc Aimé Raucoules^4^, Jean-Ehrland Ricci^1^, Michel Carles^3^

##### ^1^Université Côte d’Azur-CHU Nice-C3M INSERM U1065 Equipe 3, Nice, France; ^2^Feinberg School of Medicine, Northwestern University, IL, Chicago, USA; ^3^CHU Point-à-Pitre, Point-À-Pitre, France; ^4^CHU Nice-Université Côte d’Azur, Nice, France

###### **Correspondence:** Romain Rozier (romainrozier13@gmail.com)

*Ann. Intensive Care* 2020, **10 (Suppl 1):**F-042

**Rationale:** Myocardial ischemia reperfusion (IR) injury is the leading cause of perioperative morbi-mortality. Protective effect of pharmacologic preconditioning such as anesthetic preconditioning (APC) with sevoflurane (SEV) has been widely demonstrated in animal and human models. APC seems to protect myocardial cells from apoptosis, a programmed process of cell death tightly controlled by Bcl-2 family proteins. However, the involved mechanisms in APC have yet to be characterized. We hypothesized that APC protects against myocardial apoptotic cell death by regulating Bcl-2 anti-apoptotic members.

**Patients and methods:** To study the SEV-induced APC mechanisms against myocardial IR, we used a validated in vitro model reproducing IR injury. Rat cardiomyoblast cells H9c2 were cultivated in 0.1% O2 hypoxia in the presence of ischemia-mimicking medium. After 90 min of ischemia, the reperfusion injuries are induced by replacing the culture medium with a Krebs–Henseleit normoxic medium for 60 min. APC was performed by adding SEV directly into the culture medium at an initial concentration of 20 mM, prior to ischemia, for 60 min. We then used another preconditioning agent, metformin (MET), to explore the same signaling pathways. Apoptotic cell death was measured by caspase activity assay and western blotting (expression of cleaved caspase 3) under IR and APC conditions.

**Results:** Our model faithfully reproduced the protective effect of APC which results in a significant decreased apoptosis under IR (50% reduction of the Caspase 3 enzymatic activity, correlated with a decrease of Caspase 3 cleavage). We showed that SEV induces overexpression of the anti-apoptotic protein Bcl-xL, which is responsible for the protective effect of APC. Furthermore, these observations were confirmed in vivo in mouse heart lysates. We demonstrated that Bcl-xL overexpression was due to the activation of the protein kinase Akt. Interestingly, we were able to show that preconditioning with MET reproduces the protective effect of SEV by inducing an Akt-dependent Bcl-xL overexpression. Indeed, SEV and MET, which are both complex 1 inhibitors of mitochondrial respiratory chain, seem to share a common Reactive Oxygenated Species-dependent protective mechanism responsible for Bcl-xL protein regulation.

**Conclusion:** Our results elucidates the molecular mechanisms by which SEV induces APC against IR injuries, i.e. the role of Bcl-xL (Bcl2 family protein). Moreover, this study shows that pharmacologic preconditioning with MET promotes similar protective effect, sharing with SEV the same signaling pathways. Altogether, our results could be of interest to improve the perioperative management of patients at risk of ischemia reperfusion events, such as patients with a high cardiovascular risk.

**Compliance with ethics regulations:** Yes.

### F-043 Early sepsis worsens outcome in experimental ischemic stroke despite recanalization

#### Thomas Rambaud^1^, Lucas Di Meglio^1^, Adrien Cogo^1^, Veronique Ollivier^1^, Mialitiana Solo Nomenjanahary^1^, Sébastien Dupont^1^, Yacine Boulaftali^1^, Nathalie Kubis^2^, Benoit Ho Tin Noe^1^, Mikael Mazighi^3^, Romain Sonneville^4^

##### ^1^INSERM U 1148 laboratory for vascular translationnal science, Paris, France; ^2^INSERM U 1148 laboratory for vascular translationnal science/Service de Physiologie Clinique-Explorations Fonctionnelles, AP-HP, Hôpital Lariboisière, Paris, France; ^3^INSERM U 1148 laboratory for vascular translationnal science/Department of Interventional Neuroradiology, Rothschild Foundation Hospital, Paris, France; ^4^INSERM U 1148 laboratory for vascular translationnal science/medical and infectious intensive care unit, Bichat hospital, aphp, Paris, France

###### **Correspondence:** Thomas Rambaud (rambaud.t@gmail.com)

*Ann. Intensive Care* 2020, **10 (Suppl 1):**F-043

**Rationale:** Despite early endovascular treatment with successful recanalization, 50% of acute ischemic stroke (AIS) patients experience a poor functional outcome after a large vessel occlusion. Sepsis is frequent at the acute phase of stroke and is associated with poorer short and long term outcomes. We aimed to investigate the cerebral consequences of sepsis after recanalized AIS and explore possible mechanisms involved.

**Patients and methods:** Male C57Bl6 mice were randomly assigned to a 2x2 factorial plan to one of the 4 following groups: 1) a 60-minute middle cerebral artery (t-MCAO) transient occlusion under inhaled general anesthesia, followed 5 min after recanalization by intraperitoneal (I.P.) sepsis (LPS, 10 µg/g diluted in 100µL of NaCl0.9%), (tMCAO/LPS group); 2) t-MCAO followed by I.P. placebo (100µL of NaCl0.9%) (tMCAO/placebo group); 3) Sham operation (cervicotomy without carotid catheterization) followed by I.P. LPS. (Sham/LPS group); 4) Sham operation followed by I.P. placebo, (Sham/placebo group). In all groups, animals received subcutaneous fluid resuscitation (200µL NaCl 0.9%) immediately after the procedure and 1 h later. Twenty-four hours after recanalization, animals were scored for sepsis features and neurological deficit (on the modified neurological severity scale), (mNSS) before sacrifice. The primary outcome measurement was a composite of death and hemorrhagic transformation at 24 h. Secondary outcome measurements included neurological deficit, sepsis features, neutrophil activation reflected by plasmatic myeloperoxydase (MPO) levels, stroke volume, and microglial activation in brain parenchyma (infarct core, perilesional area, controlateral hemisphere).

**Results:** t-MCAO/LPS animals had higher mNSS (1.7 fold, p = 0.02) and sepsis (6 fold, p = 0.0018) scores at 24 h with increased plasma MPO levels at 1 h (2.8 fold, p < 0.01) and 24 h (5.9 fold, p < 0.0001), as well as, lower temperature (3.0 °C reduction, p = 0.01) and glycemia (0.9 g/l reduction, p = 0.01) as compared to tMCAO/placebo animals. t-MCAO/LPS animals had a higher risk of unfavorable outcome at 24 h (4-group comparison: p = 0.03; 2x2 analysis: t-MCAO/LPS, 6/12 − 50%- vs. t-MCAO/placebo 1/17–6%-, p < 0.01), whereas stroke volumes were not significantly different between groups. Detailed results are presented in Table 1. Compared to t-MCAO/placebo group, t-MCAO/LPS animals had 1.3 fold increase (p = 0.05) in the mean number of microglial cells in the hemisphere controlateral to t-MCAO, whereas no significant difference was observed in infarct core or peri-infarct parenchyma.

**Conclusion:** Early sepsis after experimental AIS worsens outcome and neurological deficit, without impacting stroke volume. Early sepsis-induced systemic activation of neutrophils and increased microglial activation in the hemisphere contralateral to ischemia may have an important role on neurological outcomes observed in this setting.

**Compliance with ethics regulations**: Yes.

**Table 1:** detailed results of primary outcome measurement

Outcome	Sham + placebo	Sham + LPS	t-MCAO + placebo	t-MCAO + LPS	4 group comparison
Death or Hemorrhagic transformation at 24 h	0% [0–42%] (0/5)	0% [0–42%] (0/5)	6% [3–27%] (1/17)	50% [25.3–74.6%] (6/12)	p = 0.03
Death at 24 h	0% [0–42%] (0/5)	0% [0–42%] (0/5)	6% [3–27%] (1/17)	33% [14–61%] (4/12)	p = 0.03
Hemorrhagic transformation at 24 h	0% [0–42%] (0/5)	0% [0–42%] (0/5)	0% [0–18%] (0/17)	25% [9–53%] (3/12)	p = 0.01

### F-044 Extracellular vesicles as a marker of lung injury after cardiac surgery with cardiopulmonary bypass

#### William Allali^1^, Erwan Dumontet, Murielle Gregoire, Mathieu Lesouhaitier, Arnaud Gacouin, Sebastien Biedermann, Erwan Flecher, Karin Tarte, Jean-Marc Tadié

##### ^1^CHU Rennes, Rennes, France

###### **Correspondence:** William Allali (William.ALLALI@chu-rennes.fr)

*Ann. Intensive Care* 2020, **10 (Suppl 1):**F-044

**Rationale:** Extracellular vesicles (EVs) regulate diverse cellular and biological processes via facilitating intercellular cross-talk. Several studies have suggested an association between lung injury and the generation of EVs derived from platelets, neutrophils, monocytes, lymphocytes, red blood cells, endothelial cells, and epithelial cells. Every year more than 25,000 patients require cardiac surgery with cardiopulmonary bypass (CPB). This CPB allows a substitution of the heart pump function and an oxygenation of the blood permitting a stop of the mechanical ventilation (MV). Stopping MV during CPB is responsible for lung damage, leading to postoperative systemic inflammation while maintaining MV with positive expiratory pressure (PEEP) diminished the occurrence of atelectasis and the postoperative inflammatory response. In addition, this surgery is marked by immune dysfunction, leading to real immunosuppression of patients in postoperative care. A link between pulmonary injury and postoperative immunosuppression has been established, however, the mechanisms underlying this association are not fully known and EVs may have a role in this post-operative immunosuppression. The purpose of this study is to investigate whether lung injury induced during cardiac surgery with CPB lead to the emergence of EVs. The effect of MV during CPB on the production of these EVs has also been studied.

**Patients and methods:** Patients were prospectively divided into two groups: without MV during CPB and dead space MV with positive end-expiratory pressure during CPB. PaO_2_ (arterial oxygen tension)/FIO_2_ (inspired oxygen fraction) ratio, biological markers of lung injury (CXCL10, CCL2, TNF-α, IL-1β, IL-10, RAGE, IL-8) and blood cell count were collected before, 24 h and 7 days after surgery. The quantification of plasma EVs was performed using Turnable Resistive Pulse Sensing and characterization of EVS was performed using flow cytometry before, 24 h and 7 days after surgery.

**Results:** After surgery, patients presented a significant decreased in plasmatic EVs levels. Patients with MV during CPB had a concentration of EVs significantly closer to normal 7 days after surgery than patients without MV. This suggests the existence of a sub-population of EVs with a protective role. In a second set of experiments using flow cytometry, we found that MV during CPB increased EVs derived from platelets compared to patients without MV during CPB.

**Conclusion:** Since high levels of circulating EVs have been associated with better outcome in ARDS, our data suggest that a significant decrease in plasmatic EVs after cardiac surgery with CPB could be a reliable marker of lung injury that could be prevented maintaining MV during CPB.

**Compliance with ethics regulations**: Yes.

### F-045 Prone positioning decreases macrophage recruitment in ventral lung regions of animals with experimental acute respiratory distress syndrome

#### Laurent Bitker^1^, Nicolas Costes^2^, Didier Le Bars^2^, Maciej Orkisz^3^, Mehdi Mezidi^1^, Nazim Benzerdjeb^4^, Jean Christophe Richard^1^

##### ^1^Médecine Intensive-Réanimation, hôpital de la Croix Rousse, Hospices Civils de Lyon, Lyon, France; ^2^CERMEP-Imagerie du vivant, Lyon, France; ^3^CREATIS, Lyon, France; ^4^Service d’anatomo-pathologie, Centre Hospitalier Lyon-Sud, Hospices Civils de Lyon, Lyon, France

###### **Correspondence:** Laurent Bitker (laurent.bitker@chu-lyon.fr)

*Ann. Intensive Care* 2020, **10 (Suppl 1):**F-045

**Rationale:** The benefit of prone positioning (PP) during moderate to severe acute respiratory distress syndrome (ARDS) may be related to its impact on the inflammatory response to ventilator-induced lung injuries. [11C]-PK11195 is a positron emission tomography (PET) radiotracer that allows the non-invasive quantification of macrophages. We aimed to evaluate the effects of PP on [11C]-PK11195 lung uptake in animals with experimental ARDS.

**Patients and methods:** Experimental ARDS (by hydrochloric acid) was induced in 10 pigs in supine position (SP), to obtain a PaO_2_/FiO_2_ < 300 mmHg. Animals were under general anesthesia, neuromuscular blockade, and ventilated with a 6 ml kg^−1^ tidal volume, and 5 cmH_2_O of positive end-expiratory pressure (PEEP). Immediately after experimental ARDS, animals were randomized to be prone positioned, or to remain in SP. PET and computerized tomography (CT) were acquired 4 h after randomization (H4). [11C]-PK11195 uptake was measured on the whole lungs, and by dividing the lungs into 8 regions or slices-of-interest (SOI) along the ventro-dorsal axis, and was quantified by the standardized uptake value (SUV), corrected for lung tissue density.

**Results:** PP was performed in 6 animals, and SP in 4. After ARDS induction, PaO_2_/FiO_2_ was 107 [IQR, 89–163] mmHg, and the elastance-derived transpulmonary pressure (∆PL) increased from 8 [7–10] to 17 [14–18] cmH_2_O (P < 0.01). At H4, PP animals had lower ∆PL compared to SP animals (13 [12–13] vs. 20 [17–23] cmH_2_O, p = 0.02). CT analysis showed an increase of poorly- and non-aerated lung volumes (− 500 to + 100 Hounsfield units) in ventral SOI of PP animals, compared to SP (most ventral SOI: 16 [8–17] vs. 3 [3–4]% of total SOI volume), but a decrease in dorsal SOI (most dorsal SOI: 76 [65–85] vs. 99 [99–99]%, p < 0.01 between groups on all 8 SOI). At H4, [11C]-PK11195 uptake of whole lungs of PP animals was 1.4 [1.2–1.4], and 1.4 [1.2–1.7] in SP animals (p = 0.61). In PP animals, [11C]-PK11195 SUV was significantly lower in ventral SOI, compared to SP, and significantly increased in dorsal SOI (Fig. 1, *: p < 0.01 between groups in a given SOI). In univariate analysis, [11C]-PK11195 regional SUV was positively associated with regional CT-measured PEEP-related increase in gas volume, and negatively with PEEP-related lung recruitment, but not with regional tidal volume.

**Conclusion:** During experimental ARDS, PP redistributed lung macrophage recruitment estimated by [11C]-PK11195 uptake from ventral lung regions to dorsal regions, without affecting global macrophage influx. The intensity of macrophage recruitment was associated with PEEP-related lung inflation.

**Compliance with ethics regulations:** Yes.

**Fig. 1:**
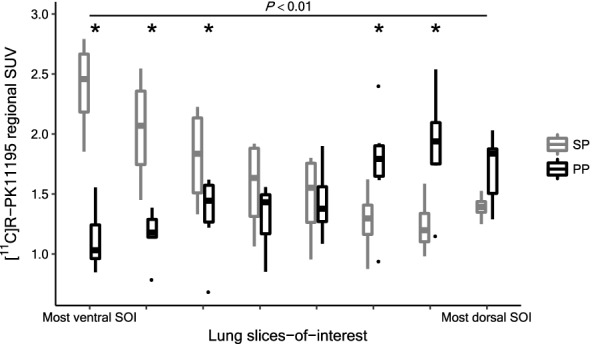
Effect of PP on regional macrophage recruitment during experimental ARDS

### F-046 Plasma exosomes as promising biomarkers for Acute Respiratory Distress Syndrome

#### Gilles Parzibut, Julien Guiot

##### CHU-Sart Tilman, Liège, Belgium

###### **Correspondence:** Gilles Parzibut (g.parzibut@gmail.com)

*Ann. Intensive Care* 2020, **10 (Suppl 1):**F-046

**Rationale:** Acute respiratory distress syndrome (ARDS) is a pleiomorphic disease characterized by a severe respiratory failure associated with an increased mortality. Nowadays, predicting clinical outcome of patients suffering from ARDS remains difficult. Therefore, identifying new biomarkers to predict patient outcome, to evaluate response to therapy and to identify new potential pathways of interest are highly needed. Exosomes are extracellular vesicles involved in cell–cell communication by transferring microRNAs (miRNAs) from donor to recipient cells. Thus, exosomal miRNAs can significantly affect biological pathways within recipient cells resulting in alterations of cellular function and the development of a pathological state. As biomarkers are highly needed in the particular field of ARDS, we realized a monocentric and prospective study to identify a new potential biomarker of interest. Therefore, a prospective plasma sampling at the diagnosis of moderate to severe ARDS according to the definition of “Berlin” has been performed. We analysed miRNA content of exosomes from plasma ARDS patients compared to healthy subjects (HS) in order to identify new potential predictive biomarkers in ARDS.

**Patients and methods:** During one-year period, patients hospitalized in the ICU of CHU Sart Tilman suffering from infectious moderate-to-severe ARDS have been included. The ethical committee review boards of the hospital approved the research protocol (B707201422832, ref: 2014/302), and informed consents were obtained. Exosomes were isolated from plasma samples of 10 ARDS patients and 10 HS with standard ultracentrifugation protocol. Exosomal miRNA content was analyzed using small RNA sequencing method, and diseases/biological processes associated to altered miRs were determined by bioinformatic analysis.

**Results:** For the first time, exosomal miRNA expression modifications were studied in patients with moderate-to-severe infectious ARDS. We identified a new signature statistically significant composed of three up-regulated miRNAs (miR-122, miR-23a and miR-126) and one down-regulated (miR-Let-7b).

**Conclusion:** We identified potential biomarkers for ARDS from plasma exosomes. Our findings may thus lead to predict ARDS outcome but also a better understanding about the roles of these miRs in the pathogenesis of ARDS and thus open new avenues for therapeutic approaches. In particular, exploit and develop the pro-fibrotic pathway induced by down-expression of miR-Let-7b. But also confirm in the future the current interest about miR-126 in its ability to restore pulmonary integrity after trauma.

**Compliance with ethics regulations**: Yes.

**Fig. 1:**
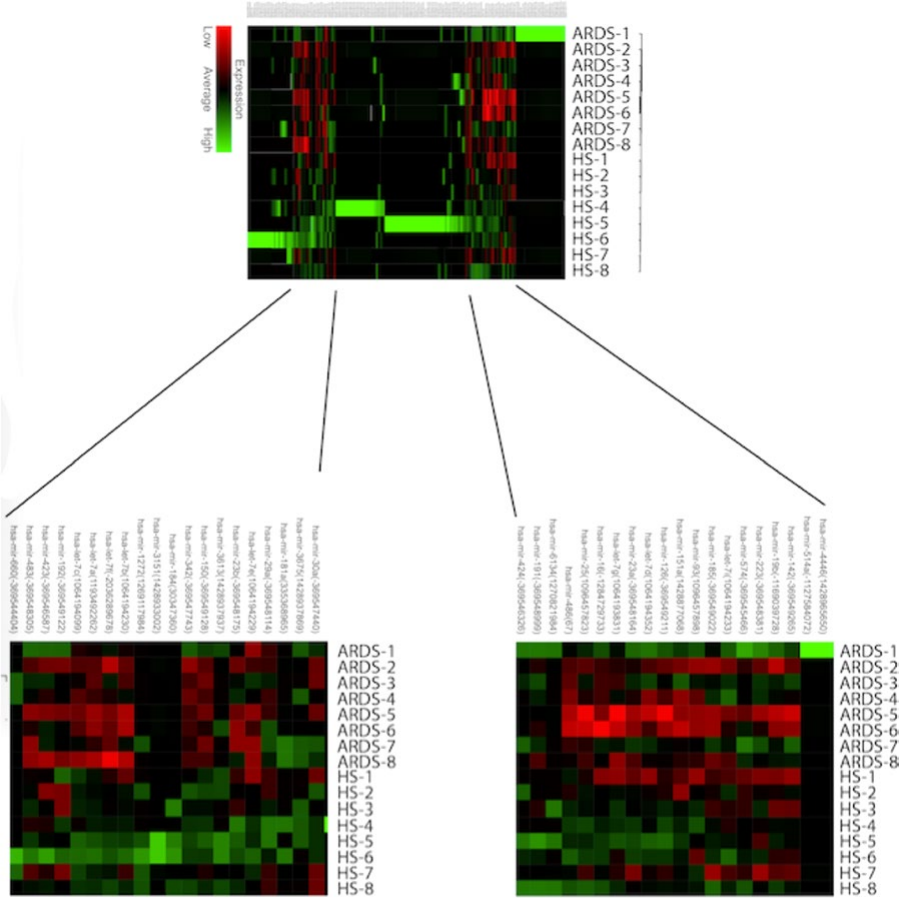
Example of heat map about expression of miRNAs in selection of ARDS population (8) vs healthy subjects (8)

### F-047 Assessment of red blood cell deformability in patients with diabetic ketoacidosis

#### Dorian Leroy^1^, Bruno Sirault^2^, Patrick Biston^1^, Karim Zouaoui Boudjletia^3^, Michael Piagnerelli^1^

##### ^1^Intensive Care. CHU-Charleroi. Université Libre de Bruxelles., 6042-Charleroi, Belgium; ^2^Internal Medicine. CHU-Charleroi. Université Libre de Bruxelles., 6042-Charleroi, Belgium; ^3^Experimental Medicine Laboratory. CHU-Charleroi. Université Libre de Bruxelles., 6110-Montigny-Le-Tilleul, Belgium

###### **Correspondence:** Dorian Leroy (Dorian.Leroy@ulb.ac.be)

*Ann. Intensive Care* 2020, **10 (Suppl 1):**F-047

**Rationale:** Diabetic ketoacidosis (DKA) is a life-threatening emergency. Microvascular hyporeactivity was reported in these patients and was completely reversibly when pH was corrected with treatment: aggressive rehydration, electrolyte replacement and insulin therapy (1). Red blood cell (RBC), a component of the microcirculation, showed alterations of their shape in diabetic patients (2) but no data were available concerning the time course of the RBC deformability during treatment for DKA. We aimed to assess the RBC deformability during DKA treatment in ICU patients.

**Patients and methods:** After approval by the ethics committee, RBCs deformability was assessed, in all ICU patients admitted for DKA and without infection, by ektacytometry technique (Laser-assisted Optical Rotational Red Cell Analyzer—LORRCA): at ICU admission, +8 h, +24 h and at the end of the ICU stay (36–48 h). Elongation index (EI) was defined as (L − W)/(L + W), where L is the length and W is the width. At 37 °C, EI values were determined in the function of shear stress (SS) in a range of 0.5–50 Pa, based upon the laser diffraction pattern changes. A higher EI indicates greater RBC deformation. RBC deformability from patients with DKA was compared at ICU admission to healthy volunteers (V) and to diabetic patients followed in consultation (D). We also studied the evolution of deformability during treatment.

**Results:** 15 ICU DKA patients compared to 31 D and 20 V were studied. As expected, glycemia and glycated hemoglobin were significantly higher in DKA compared to D (respectively: glycemia: 549 (444–872) vs 139 (92–181) mg/dL and 11.7% (9.4–13.4) vs 7.8 (7.3–8.2); all p < 0.001). DKA patients received 6279 (5482–8308) ml of fluids and 2.24 UI/Kg BW (1.71–2.42) of insulin during their first 24 h of ICU stay. RBCs deformability from DKA patients was significantly more altered at ICU admission compared to others groups (Fig. 1) and these alterations persists despite treatment. No correlations were observed between these alterations and quantity of fluids or insulin received, glycemia, glycated hemoglobin, pH, natremia, age or length of diabetes history.

**Conclusion:** In contrast of reversible microvascular hyporeactivity, RBC deformability from DKA patients was already altered at ICU admission and remains altered despite treatment. These alterations could contribute to the blood flow abnormalities observed in these patients.

**Compliance with ethics regulations:** Yes.

**Fig. 1:**
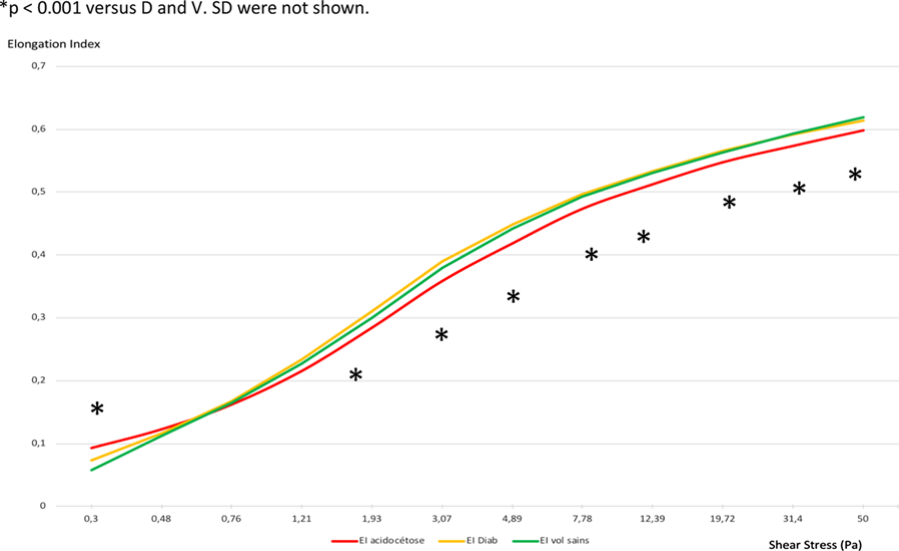
RBC deformability in DKA, diabetic patients and volunteers. *p < 0.001 versus D and V. SD were not shown

### F-048 Description of the early hemodynamic profile of septic patients assessed by focused echocardiography in the Emergency Department

#### Thomas Lafon^1^, Pauline Feydeau^2^, Alexandra Appert-Decle^3^, Mathilde Hadj^3^, Vincent Bigrat^2^, Vincent Legarcon^3^, Marine Goudelin^4^, Bruno Evrard^4^, Ana Catalina Hernandez Padilla^5^, Arthur Baisse^3^, Philippe Vignon^6^

##### ^1^Service d’Accueil des Urgences/Inserm CIC 1435, CHU Dupuytren, Limoges, France; ^2^SAMU/SMUR, CHU Dupuytren, Limoges, France; ^3^Service d’Accueil des Urgences, CHU Dupuytren, Limoges, France; ^4^Réanimation polyvalente, CHU Dupuytren, Limoges, France; ^5^Inserm CIC 1435, CHU Dupuytren, Limoges, France; ^6^Réanimation polyvalente/Inserm CIC 1435/Inserm UMR 1092, CHU Dupuytren/Université de Limoges, Limoges, France

###### **Correspondence:** Thomas Lafon (thomas.lafon@chu-limoges.fr)

*Ann. Intensive Care* 2020, **10 (Suppl 1):**F-048

**Rationale:** Sepsis remains the first cause of acute circulatory failure in the Emergency Department (ED). Standardized fluid resuscitation may not be adapted in certain patients, especially those with early sepsis-induced cardiac dysfunction in whom excessive fluid administration could be deleterious. Information on early hemodynamic profile of septic patients in the ED are scarce. Accordingly, we aimed at describing hemodynamic profiles encountered in septic patients assessed shortly after their ED admission using focused echocardiography.

**Patients and methods:** We prospectively enrolled adult patients with sepsis (qSOFA score ≥ 2) from January 2017 to July 2019 in the ED (NCT02974790). Focused echocardiography were performed by emergency physicians previously trained to ECMU 1 level. Each patient was evaluated according to a standardized protocol based on a limited number of simple binary clinical questions. Investigators interpreted on-line the echocardiographic examination, determined the hemodynamic profile based on simple yet robust criteria (hypovolemia, left ventricular [LV] or right ventricular [RV] failure, vasoplegia with hyperdynamic state, tamponade, severe mitral or aortic regurgitation, or apparently normal profile), and recorded any substantial change in planned therapeutic management (Surviving Sepsis Campaign 2016). Data were digitally stored and validated off-line by an expert in critical care echocardiography.

**Results:** Focused echocardiography were performed in 81 patients (mean age: 70 ± 15 years; men: 58%; source of infection: pulmonary 37%, urinary 25%, abdominal 25%) after a median fluid loading of 500 mL (IQR: 500–1500 mL). According to Sepsis-3 definition, 44 patients had sepsis and 37 sustained septic shock. Mean SOFA score was 5.3 ± 2.9 (hemodynamic failure 85%, respiratory failure 72%, renal failure 67%), mean lactate reached 4.7 ± 4.3 mmol/L, ICU admission involved 38% of patients and overall 28-day mortality reached 35%. Hemodynamic profile was hypovolemia in 54 patients (67%), vasoplegia in 26 patients (32%), cardiac failure in 21 patients (26%) (LV failure: n = 15; RV failure: n = 6) and without relevant hemodynamic abnormality in 9 patients (11%). Ongoing therapy was altered based on early echocardiographic assessment in 26% of cases. Mortality rate was not significantly different between groups (p = 0.46).

**Conclusion:** Although hypovolemia was predominantly identified in patients presenting to the ED with sepsis during hemodynamic assessment, early ventricular dysfunction involved one-quarter of patients. These results suggest that early focused echocardiographic assessment promises to help the front-line physician tailoring the therapeutic management of septic patients in ED, especially regarding fluid resuscitation.

**Compliance with ethics regulations:** Yes.

### F-049 Right ventricular failure in septic shock Characterization, incidence and impact on fluid-responsiveness

#### Guillaume Geri^1^, Amélie Prigent^2^, Xavier Repessé^2^, Marine Goudelin^3^, Gwenael Prat^4^, Bruno Evrard^3^, Cyril Charron^1^, Philippe Vignon^3^, Antoine Vieillard-Baron^1^

##### ^1^Ambroise Paré Hospital, Boulogne-Billancourt, France; ^2^Ambroise Paré Hospital, medical ICU, APHP, Boulogne-Billancourt, France; ^3^CHU Limoges, Limoges, France; ^4^CHU Brest, Brest, France

###### **Correspondence:** Guillaume Geri (guillaume.geri@aphp.fr)

*Ann. Intensive Care* 2020, **10 (Suppl 1):**F-049

**Rationale:** Right ventricular (RV) failure was defined by RV dilatation with systemic congestion. Tricuspid annular plane systolic excursion (TAPSE) could be of limited value. We report the incidence of RV failure in patients with septic shock, its potential impact on the response to fluids, as well as TAPSE values.

**Patients and methods:** Ancillary study of the HEMOPRED prospective multicenter study including patients under mechanical ventilation with circulatory failure. 282 with septic shock were analyzed. Patients were classified in 3 groups based on central venous pressure (CVP) and RV size (RV/LV end-diastolic area, EDA). In group 1, patients had no RV dilatation (RV/LVEDA < 0.6). In group 2, patients had RV dilatation (RV/LVEDA ≥ 0.6) with a CVP < 8 mmHg (no venous congestion). RV failure was defined in group 3 by RV dilatation and a CVP ≥ 8 mmHg. Passive leg raising (PLR) was performed.

**Results:** 41% of patients were in group 1, 17% in group 2 and 42% in group 3. In group 2 and 3, RV/LV EDA was higher than in group 1, 0.7 [0.7; 0.9] versus 0.5 [0.4; 0.5]. CVP was 12 [10; 14.5] mmHg in group 3. A correlation between RV size and CVP was only observed in group 3. Higher RV size was associated with a lower response to PLR (Figure). A large overlap of TAPSE values was observed between the 3 groups. 36.5% of patients with RV failure had an abnormal TAPSE.

**Conclusion:** RV failure is frequent in septic shock and alters fluid responsiveness. TAPSE was not accurate enough to diagnose RV failure.

**Compliance with ethics regulations:** Yes.

**Fig. 1:**
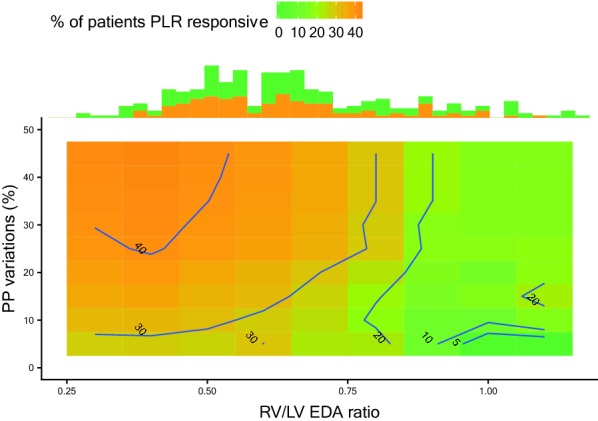
.

### F-050 Weaning failure of cardiac origin in high-risk patients: role of the fluid balance

#### Marine Goudelin^1^, Pauline Champy^2^, Jean-Bernard Amiel^1^, Bruno Evrard^1^, Anne-Laure Fedou^1^, Thomas Daix^3^, Bruno François^3^, Philippe Vignon^3^

##### ^1^Réanimation polyvalente, CHU Dupuytren, Limoges, France; ^2^Anesthésie secteur digestif et urologie, CHU Dupuytren, Limoges, France; ^3^Réanimation polyvalente/Inserm CIC 1435/Inserm UMR 1092, CHU Dupuytren, Limoges, France

###### **Correspondence:** Marine Goudelin (marine.goudelin@chu-limoges.fr)

*Ann. Intensive Care* 2020, **10 (Suppl 1):**F-050

**Rationale:** Weaning-induced pulmonary oedema (WIPO) is a leading cause of weaning failure in high-risk patients (heart failure, COPD, obesity). We hypothesized that hypervolemia associated with positive fluid balance facilitates WIPO in high-risk patients.

**Patients and methods:** In this prospective, observational, single-center study, patients with COPD and/or heart failure with reduced ejection fraction (< 40%) were studied. Exclusion criteria were non-sinus rhythm, severe mitral valve disease and inability to obtain adequate echocardiographic views. Echocardiography was performed immediately before and during spontaneous breathing trial (SBT, 30-min T-tube). Patients who failed SBT were treated according to echocardiographic results before undergoing a second SBT. Fluid balance and body weight were collected at each SBT.

**Results:** Of 304 eligible patients, 114 died before weaning, 114 had non-sinus rhythm, 8 had severe mitral valve disease, 5 had no usable echocardiographic image and 4 underwent self-extubation. Finally, 59 patients were studied (39 men; median age: 62 years [52–73]; SAPS2: 38.5 [30–53]; SOFA: 6 [4–9]; 75% heart failure and 36% COPD), and 12 of them (20%) developed WIPO. When compared to patients who succeeded, body weight (+ 0.75 kg [− 2.95; + 5.57] vs − 2.5 kg [− 4.8; − 1.0]: p = 0.02) and cumulative fluid balance (+ 143 ml [− 2654; + 4434] vs − 2326 ml [− 3715; + 863]: p = 0.007) were increased relative to admission. Doppler mitral profile depicted a higher E wave maximal velocity (101 cm/s [84–144] vs 83 cm/s [62–91]: p = 0.046) and E/A ratio (1.65 [1.28–2.38] vs 0.87 [0.69–1.06]: p = 0.001), and shorter E wave deceleration time (107 ms [94–129] vs 173 ms [128–213]: p = 0.002). After echocardiography-guided treatment, all patients who failed the first SBT could be successfully extubated after a second conclusive SBT. Fluid balance was then negative (− 2224 ml [− 7056; + 100] vs + 146 ml [− 2654; + 4434]: p = 0.005) and left ventricular filling pressures were lower than that measured during the first SBT (E wave velocity: 84 cm/s [67; 127] vs 102 cm/s [85; 149]: p = 0.017; E/E’ ratio: 7.3 [5.0; 10.4] vs 8.9 [5.9; 13.1]: p = 0.028) (Table 1).

**Conclusion:** In high-risk patients, WIPO is related to hypervolemia and excessive fluid balance. Echocardiography-guided treatment allows further successful weaning.

**Compliance with ethics regulations:** NA.

**Table 1:** Sub-group of patients who failed the first SBT

	1st SBT failed	2nd SBT succeeded	p
Before SBT			
E, cm/s	102 (85–149)	84 (67–127)	0.017
E/A	1.65 (1.17–2.66)	1.33 (0.83–2.09)	0.139
DTE, ms	103 (92–134)	133 (103–184)	0.139
E/E’	8.9 (5.9–13.1)	7.3 (5.0–10.4)	0.028
End of SBT			
E, cm/s	122 (93–168)	100 (85–122)	0.009
E/A	2.45 (1.05–3.64)	1.54 (0.88–2.31)	0.009
DTE, ms	81 (71–128)	130 (97–133)	0.093
E/E’	9.3 (7.5–12.6)	7.7 (6.2–9.9)	0.047
Weight and fluid balance			
Weight difference (admission), kg	+ 1.6 (− 2.1/+ 6.5)	− 5.0 (− 9.5/− 1.7)	0.005
Cumulated fluid balance, ml	+ 146 (− 2654/+ 4434)	− 2224 (− 7056/+ 100)	0.005

### F-051 Measurement site accuracy of inferior vena cava diameters to predict fluid responsiveness in spontaneously breathing patients

#### Morgan Caplan, Julien Goutay, Perrine Bortolotti, Thierry Onimus, Raphael Favory, Sébastien Préau

##### CHRU Lille, Lille, France

###### **Correspondence:** Morgan Caplan (Morgan.Caplan@gmail.Com)

*Ann. Intensive Care* 2020, **10 (Suppl 1):**F-051

**Rationale:** The collapsibility index of the inferior vena cava (cIVC) shows interesting performance to predict fluid responsiveness in spontaneously breathing patients. Nevertheless, measurement sites of inferior vena cava (IVC) diameters remain controversial for that purpose. The aim of the study was to test the accuracy of different measurement sites of cIVC to predict fluid responsiveness in spontaneously breathing patients.

**Patients and methods:** This study is a post hoc analysis of two prospective cohorts. We included spontaneously breathing patients without mechanical ventilation presenting with sepsis-related acute circulatory failure and considered for volume expansion (VE). We assessed hemodynamic status at baseline and after a fluid challenge (FC) induced by a 30 min-infusion of 500 mL-gelatin 4%. The IVC diameters were measured off-line with ultrasonography using the bi-dimensional mode on a subcostal long-axis view. The cIVC was calculated as [ (expiratory-inspiratory)/expiratory] diameters during standardized (cIVC-st) and unstandardized breathing (cIVC-ns) conditions. Breathing standardization consisted of a deep inspiration with concomitant control of buccal pressures and passive exhalation. Patients were referred to be responders to FC (i.e. fluid responsive) when the stroke volume increased by ≥ 10%.

**Results:** Among the 81 patients included in the study, 41 (51%) were responders to FC. The accuracy of cIVC-st and cIVC-ns before FC to predict fluid responsiveness differed significantly by measurement sites (interaction p value < 0.0001 and < 0.001, respectively). Measuring IVC diameters 4 cm from the junction of the IVC and the right atrium provided the best accuracy to predict fluid responsiveness (Fig. 1). At 4 cm caudal to the right atrium, cIVC-st was significantly better than cIVC-ns to predict fluid responsiveness: area under ROC curve 0.98 (95% CI 0.97–1.0) versus 0.84 (95% CI 0.78–0.94), p < 0.001. At 4 cm, a cIVC-st ≥ 44% and a cIVC-ns ≥ 33% predicted fluid responsiveness with sensitivity of 93% and 66%, and specificity of 98% and 92%, respectively.

**Conclusion:** Accuracy of cIVC to predict fluid responsiveness in spontaneously breathing patients depends on both measurement sites of IVC diameters and breathing conditions. Measuring IVC diameters during a standardized inspiration maneuver at 4 cm caudal to the right atrium is the most relevant mean to optimize cIVC performance to guide VE.

**Compliance with ethics regulations:** Yes.

**Fig. 1:**
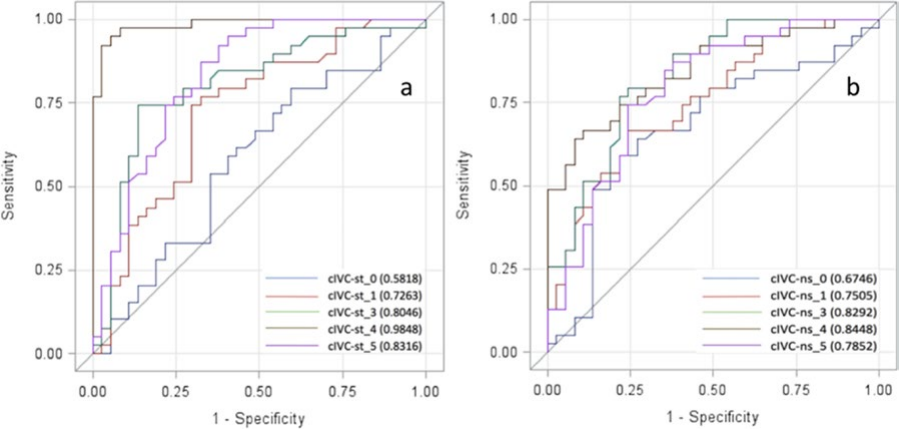
Area under ROC curves of the collapsibility index of the inferior vena cava (cIVC) assessed during standardized (cIVC-st, panel a) and unstandardized (cIVC-ns, panel b) breathing conditions to predict fluid responsiveness. 0, 1, 3, 4, 5: measurement site of cIVC 0, 1, 3, 4 and 5 cm caudal to the right atrium

### F-052 Search for trans-thoracic echocardiography predictive criteria of hemodynamic instability during intermittent hemodialysis in critically ill patients

#### Maxime Leclerc, Benoit Courteille, Cedric Daubin, Julien Dupeyrat, Suzanne Goursaud, Aurelie Joret, Toufiq Rhanem, Xavier Valette, Damien Cheyron

##### CHU Caen Côte de Nacre, Caen, France

###### **Correspondence:** Maxime Leclerc (leclerc.maxime@hotmail.fr)

*Ann. Intensive Care* 2020, **10 (Suppl 1):**F-052

**Rationale:** Intermittent hemodialysis (IHD) is increasingly used in patients admitted to intensive care unit (ICU) with acute kidney injury (AKI) requiring renal replacement therapy (RRT). However, this technique is associated with nearly 20% of episodes of perdialytic hemodynamic instability (HI), a common cause of increased morbidity and mortality. At the same time, trans-thoracic echocardiography (TTE) has become widely used in intensive care units and is now one of the hemodynamic monitoring methods used daily in the ICU setting.

**Patients and methods:** Search for one or more pre-dialysis TTE criteria predictive of perdialytic HI, defined by a systolic blood pressure (SBP) lesser than 90 mmHg or a suddain decrease in SBP of more than 40 mmHg. Prospective, observational study of standard care in a medical ICU. Collection of demographic, clinical and pre-dialysis echocardiographic data from included patients.

**Results:** Twenty-five patients with a total of 98 sessions of IHD between November 2017 and November 2018 were included in the study. TTE was performed for each patient before each IHD session. HI occurred in 31 hemodialysis sessions. In univariate analysis, the existence of prior heart disease (38% vs 0%, p = 0.04), a greater diameter of the left atrium (4.2 vs 3.6 cm, p = 0.0001), a lower cardiac output (5.8 vs 6.6 l/min, p = 0.04), a right dysfunction assessed by lowered TAPSE and S-wave (16 vs 24 mm, p < 0.0001 and 12.8 vs 15.7 cm/s, p = 0.002, respectively) and an increase in PAPS (45 vs 34 mmHg, p = 0.003) were significantly associated with the occurrence of perdialytic HI (Fig. 1). In multivariate logistic regression analysis, right cardiac dysfunction assessed by lower TAPSE (OR: 0.6, 95% Confidence intervals (CI)[0.4–0.9]; p = 0.03) and higher PAPS (OR: 1.3, 95% CI[1.1–1.5]; p = 0.03) were predictive of perdialytic HI. This result is confirmed by an analysis using a generalized estimation equation (GEE) model that finds lower TAPSE significantly associated with perdialytic hypotension episodes (adjusted OR: 0.90; 95% CI [0.82–0.99]; p = 0.03).

**Conclusion:** Right cardiac dysfunction appears to be an easily assessable echocardiographic predictor of the occurrence of hemodynamic instability during IHD in critically ill patients.

**Compliance with ethics regulations:** Yes.

**Fig. 1:**
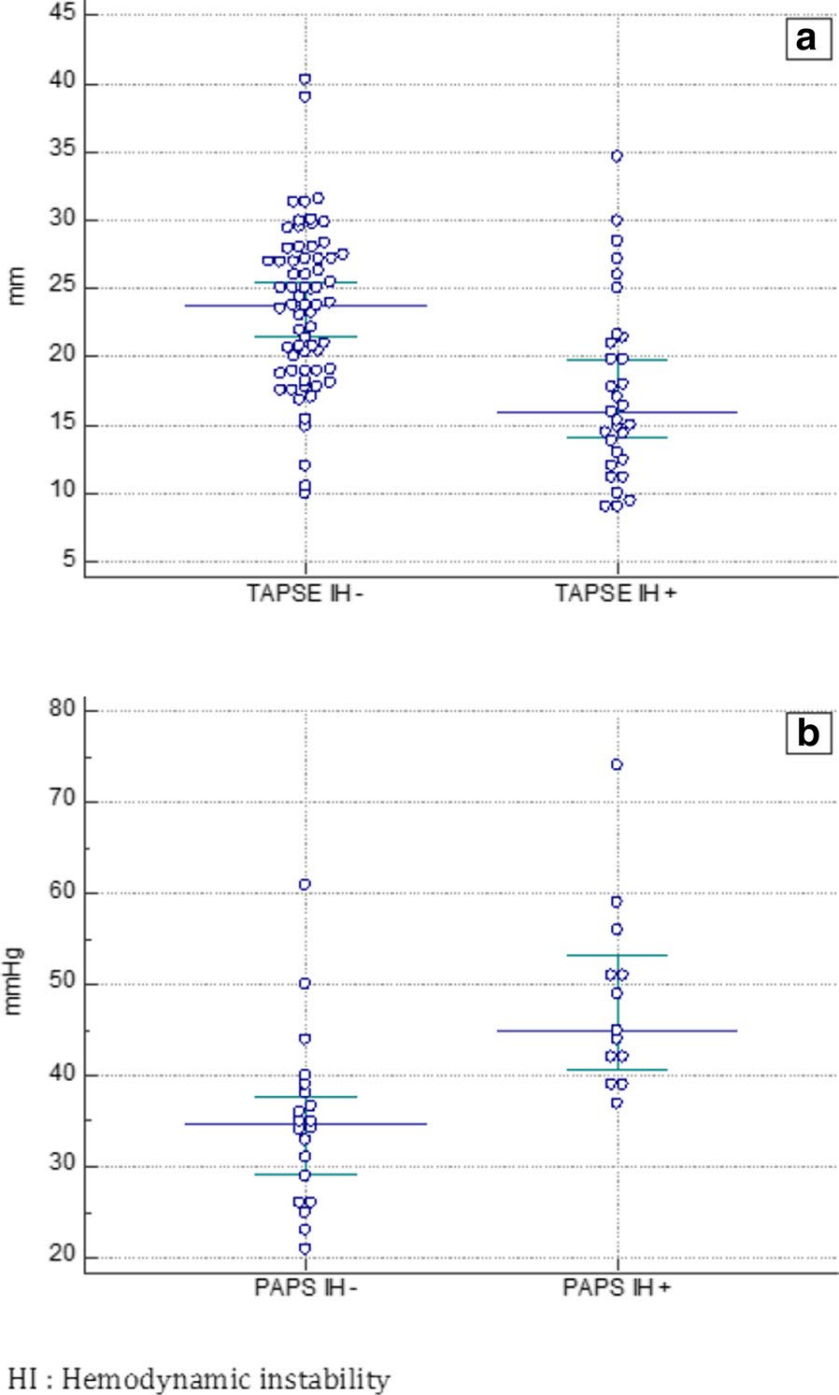
Comparison of predialysis TAPSE (**a**) and PAPS (**b**) values according to the occurrence or not of hemodynamic instability during the dialysis session

### F-053 Global longitudinal strain is the best transthoracic echocardiography parameter of left and right ventricular systolic function in critically ill patients

#### Pierre Dupland, Ariane Gavaud, Frédéric Pene, Jean-Daniel Chiche, Jean-Paul Mira, Alain Cariou, Mathieu Jozwiak

##### Service de Médecine Intensive Réanimation, Hôpitaux universitaires Paris-Centre, Hôpital Cochin, Paris, France

###### **Correspondence:** Pierre Dupland (pierre.dupland@gmail.com)

*Ann. Intensive Care* 2020, **10 (Suppl 1):**F-053

**Rationale:** Several transthoracic echocardiography (TTE) parameters of left (LV) and right ventricular (RV) systolic function are available. We compared the ability of these different parameters to track changes in LV or RV systolic function and to detect LV or RV systolic dysfunction in critically-ill patients.

**Patients and methods:** In 20 patients (15 mechanically ventilated and 2 with atrial fibrillation), TTE examinations were performed before and after i) infusion of 500-mL of saline (n = 10), ii) changes in norepinephrine (n = 8), iii) or in dobutamine (n = 2) dosage. For the LV systolic function, we compared the mitral annular plane systolic excursion (MAPSE), the systolic (s’) peak velocity of the lateral mitral annulus and the global longitudinal strain (GLSLV) to the LV ejection fraction (LVEF), considered as the gold standard. For the RV systolic function, we compared the tricuspid annular plane systolic excursion (TAPSE), the systolic peak (S) velocity of the tricuspid annulus and the global longitudinal strain (GLSRV) to the RV fractional area change (FAC), considered as the gold standard.

**Results:** After pooling all values, LVEF (55 ± 12% at baseline) was better correlated to GLSLV (r^2^ = 0.71) than to MAPSE (r^2^ = 0.25) and s’ wave (r^2^ = 0.13) (each p < 0.05). The concordance rate between changes (in %) in LVEF and in the other parameters of LV systolic function was 87% for GLSLV, 72% for MAPSE and 67% for s’ wave. Both MAPSE and s’ wave could not reliably detect moderate (30% ≤ LVEF ≤ 40%) or severe (LVEF < 30%) LV dysfunction. Conversely, a GLSLV > − 12% predicted moderate LV dysfunction with a sensitivity of 100% (95% IC: 29–100%) and a specificity of 89% (95% IC: 71–98%) and a GLSLV > − 9.5% predicted severe LV dysfunction with a sensitivity of 100% (95% IC: 16–100%) and a specificity of 96% (95% IC: 82–100%). After pooling all values, FAC (32 ± 11% at baseline) was better correlated to GLSRV (r^2^ = 0.57) than to TAPSE (r^2^ = 0.31) and S wave (r^2^ = 0.24) (each p < 0.05). The concordance rate between changes (in %) in FAC and in the other parameters of RV systolic function was 81% for GLSRV, 72% for TAPSE and 50% for S wave. Both TAPSE and S wave could detect RV dysfunction (FAC ≤ 35%) with moderate reliability only. Conversely, a GLSRV > − 12% detected RV dysfunction with a sensitivity of 56% (95% IC: 30–80%) and a specificity of 100% (95% IC: 77–100%).

**Conclusion:** In critically-ill patients, GLSLV and GLSRV seem to be the best TTE parameters of LV and RV systolic function. Enrolments are still ongoing, which may allow further analysis.

**Compliance with ethics regulations:** Yes.

### F-054 The Applicability of Fluid Responsiveness Indices in Circulatory failure (AFRIC study)

#### Rui Shi, Nello De Vita, Francesco Gavelli, Jean-Louis Teboul, Arthur Pavot, Xavier Monnet

##### Service de médecine intensive-réanimation, Hôpital de Bicêtre, Hôpitaux universitaires Paris-Saclay, Assistance publique—Hôpitaux de Paris, Inserm UMR S_999, Université Paris-Sud, Le Kremlin-Bicêtre, France

###### **Correspondence:** Rui Shi (shiruidingding@hotmail.com)

*Ann. Intensive Care* 2020, **10 (Suppl 1):**F-054

**Rationale:** Passive leg raising (PLR), pulse pressure variation (PPV), and the 15-second end-expiratory occlusion test (EEXPO) are frequently used to assess preload responsiveness. However, there are conditions in which they are not valid or feasible, which may preclude their applicability in the daily clinical practice. The aim of this study was to estimate the prevalence of such conditions in critically ill patients with acute circulatory failure.

**Patients and methods:** Between January and April 2019, all patients of a 25-bed medical ICU were daily screened and those with acute circulatory failure, defined by norepinephrine infusion or fluid therapy > 1L during the previous 24 h, were included. In each of them, we screened the criteria of validity/feasibility of PPV, PLR and EEXPO.

**Results:** Eighty-four patients (78% with septic shock, 8% with cardiogenic shock, 8% with hypovolemic shock, 6% with non-septic vasoplegic shock) were enrolled in the study. Among them, norepinephrine infusion was ongoing at the time of enrolment in 95% of the patients whilst 69% were under mechanical ventilation, and 30% with acute respiratory distress syndrome. PLR was not applicable in 37% of cases. This was mainly due to venous compression stocking (17% of cases), intra-abdominal hypertension (11% of cases), and either an absence of cardiac output monitoring or impossibility to perform echocardiography (9% of cases). Among the 58 intubated patients, PPV was applicable in 16% of cases, including cases with high PPV under conditions generating false negatives (low tidal volume or lung compliance) or low PPV values under conditions generating false positives (spontaneous breathing, cardiac arrythmias). However, PPV was not interpretable in 84% of cases. This was mainly due to low tidal volume ventilation (27% of cases), spontaneous breathing activity (12% of cases), while the remaining non-interpretable cases (51%) had more than one reason. In the 58 intubated patients, EEXPO was not applicable in 21% of cases. This was due to impossibility for patients to sustain a 15-s hold of mechanical ventilation in 58% of cases, and either an absence of cardiac output monitoring or the impossibility to perform echocardiography in 42% of cases. PLR and EEXPO were both valid and feasible in 35% of the patients, and the three tests were all feasible in only 4% of patients.

**Conclusion:** In patients with acute circulatory failure, among the different indices of preload responsiveness, PLR cannot be performed in 37% of all cases, mainly due to venous compression stocking and intra-abdominal hypertension. PPV cannot be used in 84% of intubated cases. EEXPO is not feasible in 21% of cases.

**Compliance with ethics regulations:** Yes.

### F-055 Sleep Apnea Syndrome in hypercapnic respiratory failure: prevalence and validation of screening scores

#### Fadoua Houri, Wiem Nouira, Hedia Ben Ahmed, Zeineb Hammouda, Manel Lahmar, Fekri Abroug, Lamia Besbes

##### CHU F.Bourguiba, Monastir, Tunisia

###### **Correspondence:** Fadoua Houri (hourifadwa@gmail.com)

*Ann. Intensive Care* 2020, **10 (Suppl 1):**F-055

**Rationale:** Comorbid association between chronic respiratory diseases and sleep apnea syndrome (SAS) revealed frequent with systematic search in ICU following ICU stay. This association carries prognosis impact depending whether specific treatment is implemented or not. NoSAS and Stop Bang scores are proposed for screening of SAS in general population. The aim of the present study is to report the prevalence of SAS in ICU patients admitted for hypercapnic respiratory failure and compare association of NoSAS and Stop Bang score with SAS severity.

**Patients and methods:** The study was conducted between January 2016 and September 2018. Patients consecutively admitted in the ICU for hypercapnic respiratory failure had calculation of a no SAS and STOP Bang scores at admission. In survivors nocturnal polygraphic records was performed 3 to 4 weeks following ICU discharge. The association between the number of apnea–hypopnea episodes, BMI, and clinical variables suggestive of SAS, was tested by POISSON regression model.

**Results:** During the study-period, 65 patients (mean age: 69 ± 9 years, pH 7.29 ± 0.03, PaCO_2_ 75 ± 16) were admitted for hypercapnic respiratory failure. Non invasive ventilation was used in 85% and death occurred in six patients. Polygraphic records were performed in 45 (9 lost to follow-up) Mean apnea–hypopnea index was 40 ± 15 with a minimum of 7 and a maximum of 62. Poisson logistic regression showed that No SAS (p = 0.006) but not Stop Bang (p = 0.1) was associated with the level of apnea–hypopnea index.

**Conclusion:** ICU patients admitted for hypercapnic respiratory failure have a very high rate of associated Sleep Apnea Syndrome. Among existing screening scores, No SAS and not Stop Bang are associated with severity.

**Compliance with ethics regulations:** NA.

### F-056 Extracorporeal CO_2_ Removal in acute exacerbation of COPD not responding to non-invasive ventilation: a single center experience

#### Daniel Silva^1^, Rita Serbouti^2^, Laurent Laine^1^, Nathalie Memain^1^, Vincent Ioos^1^, Mathilde Lermuzeaux^1^, Camille Legouy^1^, Morgan Benais^1^, Luis Ferreira^1^, Jérôme Aboab^1^

##### ^1^Médecine Intensive Réanimation-Centre Hospitalier de Saint-Denis, Saint-Denis, France; ^2^Fresenius Medical Care France, Fresnes, France

###### **Correspondence:** Daniel Silva (daniel.silva@mac.com)

*Ann. Intensive Care* 2020, **10 (Suppl 1):**F-056

**Rationale:** Extracorporeal carbon dioxide removal (ECCO_2_R) has shown a raising interest in the context of acute exacerbation of chronic obstructive pulmonary disease (eCOPD) to reduce length or avoid mechanical invasive ventilation (MIV) when non-invasive ventilation (NIV) has failed.

**Patients and methods:** We conducted a single center retrospective analysis on successive eCOPD patients for whom NIV failed. ECCO_2_R was performed with the iLA Activve (Xenios Novalung, Heilbronn, Germany) device from February 2015 to February 2019. We compared blood gas measurements and complication rates on eCOPD patients treated with MIV after NIV failure from January 2010 to February 2015. 28 patients were treated with ECCO_2_R and 26 patients with MIV. Variables are reported as mean ± SD. Quantitative values are compared with t test (95% confidence interval) and qualitative values with Pearson’s Chi squared test withYates’ continuity correction. Population distribution was tested with the Shapiro–Wilk normality test.

**Results:** In the ECCO_2_R group (SAPS 48 ± 15, age 66.1 ± 11.4 years), the ECCO_2_R duration was 5.7 ± 4.2 days. The arterial blood pH 6 h before ECCO_2_R was 7.24 ± 0.05 and 7.41 ± 0.06 at decannulation (p < 0.001). The PaCO_2_ value 6 h before ECCO_2_R was 85 ± 21 and 53 ± 9.9 before decannulation (p < 0.001). In the MIV group (SAPS 50 ± 14, age 71.4 ± 11 years), the MIV duration was 28.2 ± 42.5 days. The mean arterial blood pH 6 h before intubation was 7.30 ± 0.15 and 7.40 ± 0.06 before extubation (p = 0.0741). The mean PaCO_2_ value 6 h before MIV was 77 ± 32 and 51 ± 13.3 before extubation (p < 0.001). Three patients (10.7%) needed intubation despite the ECCO_2_R. Three patients had major bleeding complications in the ECCO_2_R group. Three patients had device related thrombosis and one had a venous thrombosis. In the MIV group, 8 patients (29%) had transfusions, five (18%) had extubation failure, 6 patients (21%) had autoextubation and 4 (14%) got a ventilator associated pneumonia. Length of stay in the ICU for the ECCO_2_R and the MIV group was 19 ± 15.5 days and 31 ± 42.2 days respectively. Mortality at 28 days and 90 days was 8% and 14% respectively in the ECCO_2_R group and 16% and 30% in the MIV group.

**Conclusion:** Our results show that ECCO_2_R is feasible and brings significant improvement on pH and PaCO_2_ with relatively low complication rate. Evidence for a mortality benefice of ECCO_2_R in eCOPD patients failing NIV therapy has yet to be proven in a larger and randomized study.

**Compliance with ethics regulations:** Yes.

### F-057 High-Flow Nasal nebulization of Salbutamol during severe acute exacerbation of Chronic Obstructive Pulmonary Disease

#### Clément Beuvon^1^, Nicolas Marjanovic^1^, Rémi Coudroy^1^, Christophe Rault^1^, Vanessa Bironneau^1^, Xavier Drouot^1^, René Robert^1^, Arnaud Thille^1^, Jean-Pierre Frat^1^

##### ^1^CHU de Poitiers, Poitiers, France

###### **Correspondence:** Clément Beuvon (cbeuvon@hotmail.fr)

*Ann. Intensive Care* 2020, **10 (Suppl 1):**F-057

**Rationale:** Patients with severe acute exacerbations of Chronic Obstructive Pulmonary Disease (COPD) may benefit from high-flow nasal oxygen regarding its physiological effects and good tolerance. Bronchodilator vibrating mesh nebulization through high-flow nasal oxygen circuit has been described to induce similar effect to standard facial mask jet nebulization in stable COPD patients. We aim to evaluate whether vibrating mesh nebulization of salbutamol through high-flow nasal oxygen circuit is efficient in unstable patients with COPD.

**Patients and methods:** We conducted a monocenter non-randomized physiological prospective cross-over study, between January and September 2019, including ICU patients with severe acute exacerbation of COPD and respiratory acidosis treated by salbutamol nebulization. Spirometry and airway resistances records were performed after a 3-h wash-out period without bronchodilator, before and after vibrating mesh nebulization of 5 mg salbutamol through high-flow nasal oxygen circuit. The primary endpoint was Forced Expiratory Volume in 1 s after salbutamol nebulization. Secondary endpoints included other spirometry parameters, clinical parameters, dyspnea assessed by a Borg scale.

**Results:** Fourteen consecutive patients were included, Forced Expiratory Volume in 1 s increased significantly after salbutamol nebulization through high-flow nasal oxygen (91 ± 111 mL, p = 0.005), as well as Forced Vital Capacity (190 mL ± 193, p = 0.005). Airway resistances were not significantly changed after nebulization (− 0.73 ± 1.67, p = 0.08) as well as Peak Expiratory Flow (+ 231 mL ± 439, p = 0.09). No difference was observed on Borg scale (p = 0.06) and respiratory rate (p = 0.18) after salbutamol nebulization, while heart rate increased significantly (p = 0.01).

**Discussion:** Salbutamol nebulization using vibrating mesh nebuliser placed on high-flow nasal oxygen circuit induces a significant but moderate bronchodilation in patients with severe acute exacerbation of COPD. Moreover, improvement of Forced Vital Capacity after salbutamol nebulization suggests a reduction of dynamic hyperinflation.

**Conclusion:** Salbutamol vibrating mesh nebulization through high-flow nasal oxygen circuit increases significantly Forced Expiratory Volume in 1 s.

**Compliance with ethics regulations**: Yes.

### F-058 T-piece versus sub-therapeutic pressure support for weaning from invasive mechanical ventilation in patients with chronic obstructive pulmonary disease: a comparative prospective study

#### Amira Jamoussi, Fatma Jarraya, Samia Ayed, Takoua Merhabene, Jalila Ben Khelil, Mohamed Besbes

##### Abderrahmen Mami Hospital, Tunis, Tunisia

###### **Correspondence:** Amira Jamoussi (dr.amira.jamoussi@gmail.com)

*Ann. Intensive Care* 2020, **10 (Suppl 1):**F-058

**Rationale:** The best weaning strategy for patients with chronic obstructive pulmonary disease (COPD) remains unknown. The spontaneous breathing trial (SBT) represents a crucial step of weaning, but the choice between the T-piece (SV-Tube) or the sub-therapeutic setting of the level of pressure support without positive expiratory pressure (PSV) is still a matter of debate. We aimed to compare the success of extubation between two groups of COPD patients according to the SBT type (VS-Tube vs PSV).

**Patients and methods:** It was a prospective and comparative study, from April 2017 to March 2019, at the Abderrahmen Mami hospital’s intensive care unit (ICU). COPD patients who underwent invasive mechanical ventilation (MV) for at least 24 h and met the criteria for weaning were included and randomized to SV-Tube or PSV. A multivariate analysis was performed to determine the association between the SBT modality and the success of extubation (no re-intubation during the 48 h following extubation).

**Results:** During the two years’ study, 32 patients were included. The mean age was 66 ± 10 years, the sex-ratio was 4.33. Weaning process was simple in 16 patients (50%), difficult in 10 patients (31%) and prolonged in 6 patients (19%). Fifteen and 17 patients were respectively randomized to the SV-Tube and PSV groups. The mean duration of MV before randomization was comparable between the 2 groups (SV-tube 6.87 ± 4.3 days vs PSV 6.06 ± 4.8 days, p = 0.622). Mean weaning time (days) was 3.73 [1–16] for the SV-Tube group and 4.35 [1–20] for the PSV group. The mean total MV duration (days) was higher in the SV-Tube group than in the PSV group (15.13 vs 7.65, p = 0.105). The number of re-intubated patients within 48 h following extubation was higher in the PSV group (7/17 vs 1/15, p = 0.024) as well as the overall reintubation rate (64.7% vs 20%, p = 0.011). In multivariate analysis, the SBT’s trial was independently associated to the success of extubation (OR = 0.081, IC [0.008–0.865], p = 0.037) in favor of SV-tube’ modality. The median length of stay in intensive care was 16 days [10; 26]. The mortality was higher in the PSV group (7/17 vs 2/15, p = 0.08). Extubation failure was a factor associated with mortality (OR = 8.333, CI [1.392, 49.872], p = 0.02).

**Conclusion:** Ventilation weaning was easy in 50% of intubated COPD patients. SV-Tube as SBT modality was associated to success of extubation in patients with COPD. Mortality in intensive care was significantly higher in re-intubated patients.

**Compliance with ethics regulations**: Yes.

### F-059 The incidence of laryngo-tracheal lesions in tracheotomized patients in Post Intensive Care Rehabilitation Unit

#### Laurence Donetti, Dominique Kaminski, Mohamed Laissi, Jean-Claude Marchal, Pascal Meyer, Karim Nssair, Gérald Choukroun

##### CH Forcilles, Ferolles Attilly, France

###### **Correspondence:** Laurence Donetti (laurdonetti@gmail.com)

*Ann. Intensive Care* 2020, **10 (Suppl 1):**F-059

**Rationale:** Post intensive care rehabilitation units (PICRU or SRPR) are dedicated to chronic critically ill patients management: particularly prolonged ventilator weaning, removal of tracheotomy tube (decannulation) and functional recovery. In our unit, more than 80% of patients are tracheotomized. A broncoscopy is systematically performed prior to decannulation either immediately before or to assess laryngeal abnormalities in case of swallowing disorders. The anatomical and functional abnormalities are investigated and the good positioning of the tracheal cannula is checked.

**Patients and methods:** We conducted a retrospective single-centre observational study from January 1st 2017 to June 30th 2019 in order to evoluate the rates of the different bronchoscopic abnormalitie patterns among the trachotomized patients admitted during the study period.

**Results:** During the period, 169 patients were admitted in our unit. 109 patients were eligible for analysis. 36 patients (33%) didn’t have lesion vs 73 patients (67%) who had one or more lesions. 30 patients had swallowing disorders. Characteristics of patients without lesion were: mean age of 63 years, lengh of stay of 30.3 days and a 100% success rate of tracheotomy removal after the bronchoscopy. Characteristics of patients with lesions were: mean age of 63 years, length of stay of 49 days. 86% of them underwent successfull decannulation: 47% immediately after the first bronchoscopy and 39% later. Laryngal lesions are listed in the table.

10 patients had both epiglottis and arytenoides edema, 7 patients had both epiglottis, arytenoides and vocal cords edema. Tracheal disorders patterns were: tracheal ring fractures (15 patients), granulomas (11 patients), tracheomalacia (7 patients), subglottic edema (4 patients), tracheal stenosis (2 patients).

Tracheal cannula malposition was found in 9 patients.

**Conclusion:** Laryngeal or tracheal abnormalities are frequent among chronic critically ill patients admitted in PICRU and probably associated with delayed ventilator weaning and decannulation. Further studies are needed to understand better causes and consequences.

**Compliance with ethics regulations:** Yes.

**Table 1:** Patterns of laryngeal lesions

Lesions	**Number of patients**
Epiglottis edema	25
Arytenoides edema	43
Vocal cords edema	10
Vocal cords nodule	1
Vocal cords posterior notch	2
Larynx abnormal sensibility	19
Larynx abnormal mobility	18

### F-060 Which score to predict NIV failure in Hypercapnic Respiratory Failure: HACOR score vs ROX index

#### Wiem Nouira, Zeineb Hammouda, Syrine Maatouk, Hedia Ben Ahmed, Amir Bedhaifi, Yed Maatouk, Fadoua Houri, Fekri Abroug, Lamia Besbes

##### CHU F.Bourguiba, Monastir, Tunisia

###### **Correspondence:** Wiem Nouira (wiemnouira1@gmail.com)

*Ann. Intensive Care* 2020, **10 (Suppl 1):**F-060

**Rationale:** Non-invasive ventilation has become the mainstay in hypercapnic respiratory failure. Delaying intubation and invasive ventilation is associated with a worse outcome in these patients. Although a predictive score of NIV failure has been validated for hypoxemic respiratory failure no such score exists in hypercapnic respiratory failure. The aim of our study is to compare the performance of two scores in the predictive NIV failure hypercapnic respiratory failure.

**Patients and methods:** Consecutive patients admitted between January 2017 and July 2019 for hypercapnic respiratory failure, were included. HACOR score and ROX score were calculated in each patient at admission. In patients ventilated non-invasively, the outcome (NIV success or failure) was noted. The area under curve (AUC) and operative characteristics were computed for both scores.

**Results:** During the study-period, 107 out of 133 patients admitted for hypercapnic respiratory failure received NIV as the primary ventilatory mode. These patients were mainly men (77/30), had a mean age of 66.7 ± 10 years and had the following pulmonary disease: COPD exacerbation 50.5%, obesity-hypoventilation syndrome 18.7%, bronchiectasis 15.9%, and other diseases: 8.4%. NIV failure occurred in 39 patients (36.4%) and ICU mortality in 18.7%. Mean HACOR score and ROX score were 5.8 ± 3.6 and 18.3 ± 2, respectively. The AUC under ROC was higher for HACOR than ROX (0.91 and 0.76 respectively) (Fig. 1). The HACOR score (cut-off 6) had a sensitivity of 0.93 and specificity of 0.85.

**Conclusion:** HACOR score seems more accurate in predicting NIV failure in hypercapnic respiratory failure. Further prospective validation is needed.

**Compliance with ethics regulations**: NA.

### F-061 Characteristics and outcome of patients managed for difficult weaning in a weaning and rehabilitation care unit

#### Sarah Makoudi, Julie Delemazure, Martin Dres, Alexandre Demoule, Elise Morawiec

##### Unité de soins de rééducation post réanimation Service de Pneumologie, Médecine Intensive, Paris, France

###### **Correspondence:** Sarah Makoudi (smmakoudi@gmail.com)

*Ann. Intensive Care* 2020, **10 (Suppl 1):**F-061

**Rationale:** Published data on outcomes in respiratory weaning centers are limited and seem to depend on the organisation of healthcare systems and patient case-mix. The weaning center of our university hospital (Post intensive care rehabilitation unit) admits for weaning and rehabilitation patients from medical and surgical intensive care units without severe neurological pathologies. The aim of this study was to describe patient’s characteristics and outcome (weaning outcomes and survival) and to compare in subgroups according to the initial medical, surgical or cardiac surgical context.

**Patients and methods:** We conducted a monocentric retrospective observational study between 01/02/2016 and 30/06/2018. «Successful outcome» was defined by the association of survival and weaning from invasive ventilation. Factors associated with evolution were investigated by uni- and multivariate analysis. Survival after discharge was analysed according to the initial context and according to the type of ventilation at discharge.

**Results:** Among 215 patients included, 167 (77.6%) had a successful outcome with high use of non-invasive ventilation (NIV) (37%). Respiratory history (p = 0.02), female gender (p < 0.001), IGS2 score at admission to the SRPR (p = 0.001) and non-cardiac surgical setting (p < 0.012) were associated with an adverse course. The 6-month survival rate was 84% in discharged patients. The outcome was not different in the tree subgroups. NIV rate at discharge was high in the subgroup of cardiac surgery patients.

**Conclusion:** A multidisciplinary and personalised approach by a specialized weaning unit can provide a successful service model for patients who require liberation from prolonged invasive mechanical ventilation.

**Compliance with ethics regulations:** Yes.

### F-062 Hemodynamic impact of High-dose Insulin Euglycemic Therapy in calcium channel blockers poisoning

#### Julien Goutay^1^, Erika Parmentier-Decrucq^1^, Olivier Nigeon^2^, Julien Poissy^1^, Daniel Mathieu^1^, Thibault Duburcq^1^

##### ^1^CHU de Lille, Pôle de Réanimation, Hôpital Salengro, Lille, France; ^2^CH de Lens, Service de Réanimation, Lens, France

###### **Correspondence:** Julien Goutay (julien.goutay@gmail.com)

*Ann. Intensive Care* 2020, **10 (Suppl 1):**F-062

**Rationale:** High-dose Insulin Euglycemic Therapy (HIET) is recommended as first line therapy for calcium channel blockers (CCBs) poisoning because of its inotropic effect. Our first objective was to study its hemodynamic impact.

**Patients and methods:** We performed a retrospective cohort study of all consecutive patients admitted for CCBs poisoning treated with HIET, in one ICU at the University Hospital of Lille between January 2013 and July 2018. The hemodynamic impact was studied through mean arterial pressure (MAP), Vasoactive-Inotropic Score (VIS) and MAP/VIS ratio during the 24 h following HIET initiation. Metabolic parameters were also collected.

**Results:** 104 patients admitted for CCBs poisoning. 43 patients treated with HIET in ICU (7 patients without circulatory shock, 5 patients with shock after HIET and 31 patients with shock at baseline before HIET). Among shocked patients at baseline (n = 31), no hemodynamic improvement was found except an increased MAP/VIS ratio at H24 (p < 0.05). On the contrary, an initial worsening of VIS (137 [26–267] at H0 vs. 143.4 [40.1–330] at H2, p = 0.03) was observed. 22 patients (51.2%) presented a severe hypokalemia and 10 (23.2%) a severe hypoglycemia. The existence of severe conduction abnormality and the value of Simplified Acute Physiology Score II were associated with mortality in the multivariate analysis.

**Conclusion:** No hemodynamic improvement was observed in the first hours following HIET. Moreover, severe metabolic disorders occurred. A randomized prospective study aiming to assess the HIET risk–benefit ratio is needed.

**Compliance with ethics regulations:** Yes.

**Fig. 1:**
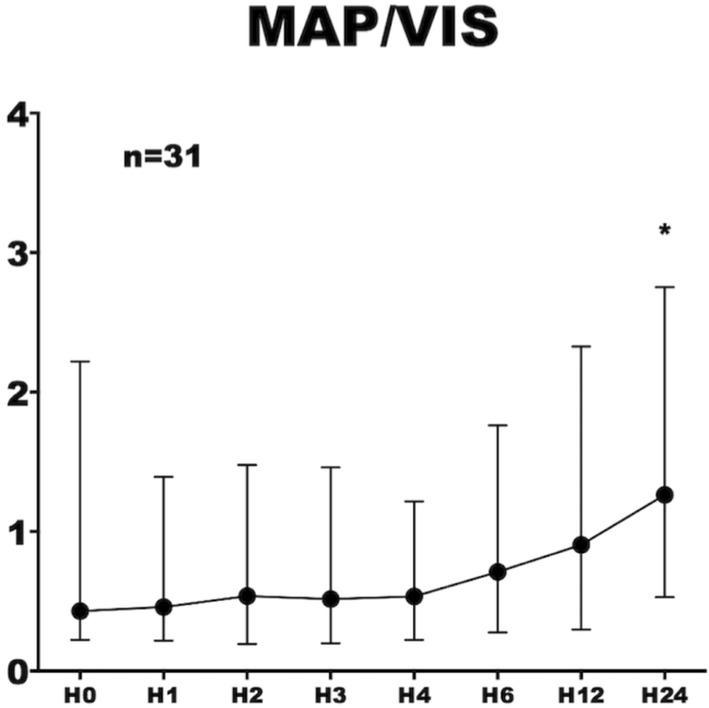
Evolution of MAP/VIS ratio among shocked patients at baseline

### F-063 Liver Dysfunction and Ketamine use in critically ill patients with ARDS: a Post Hoc analysis of the ACURASYS study

#### Florence Daviet^1^, Benjamin Coiffard^1^, Arnaud Gacouin^2^, Gilles Perrin^3^, Anderson Loundou^4^, Samir Jaber^5^, Jean-Michel Arnal^6^, Dider Perez^7^, Jean-Marie Seghboyan^8^, Jean-Michel Constantin^9^, Jean-Yves Lefrant^10^, Claude Guérin^11^, Gwenael Prat^12^, Jean-François Timsit^13^, Antoine Roch^1^, Laurent Papazian^1^, Jean-Marie Forel^1^

##### ^1^Médecine Intensive Réanimation, Service du Pr PAPAZIAN, Hôpital Nord, Marseille, France; ^2^Service des maladies infectieuses et réanimation médicale, hôpital Pontchaillou, centre hospitalier universitaire de Rennes, Rennes, France; ^3^Réanimation des Urgences et Medicale, CHU la Timone 2, Marseille, France; ^4^Unité d’Aide Méthodologique à la Recherche Clinique-Assistance Publique-Hôpitaux de Marseille, Marseille, France; ^5^Département d’Anesthésie-Réanimation, hôpital Saint-Éloi, Montpellier, France; ^6^Service de Réanimation Polyvalente, Hôpital Sainte Musse, Toulon, France; ^7^Service de Réanimation-USC, Hôpital Lons-le-Saunier, Lons-Le-Saunier, France; ^8^Réanimation Hopital Européen, Marseille, France; ^9^Department of Perioperative Medicine, University Hospital of Clermont-Ferrand, Clermont-Ferrand, France; ^10^Department of Anaesthesiology, Critical Care and Emergency Medicine, CHU de Nîmes, Nimes, France; ^11^Réanimation Médicale Hôpital de la Croix Rousse, Lyon, France; ^12^Medical Intensive Care Unit, Cavale Blanche University Hospital, Brest, France; ^13^Medical and Infectious Diseases ICU (MI2), Bichat Hospital, Paris, France

###### **Correspondence:** Florence Daviet (florence.daviet@ap-hm.fr)

*Ann. Intensive Care* 2020, **10 (Suppl 1):**F-063

**Rationale:** Ketamine is used in the induction and maintenance of general anesthesia. Recently, there were concerns regarding its liver toxicity. We conducted a study to investigate the link between Ketamine use and Liver Dysfunction (LD) in Intensive Care Unit (ICU) patients.

**Patients and methods:** Data were extracted from the [anonymized] study, a randomized controlled trial designed to evaluate the effect of Cisatracurium on 90-day mortality rate in moderate and severe Acute Respiratory Distress Syndrome (ARDS) patients. The main endpoint was the occurrence of a LD defined as a total serum bilirubin superior or equal to 33 micromol/l. A matched case–control cohort was created: cases, receiving at least 1 day of continuous Ketamine infusion, were paired 1 for 1 with controls according to treatment with Cisatracurium, hepatic and cardiovascular SOFA sub-score, total serum bilirubin level at the time of inclusion, age, sex, ARDS from septic origin, shock any-time after inclusion. An analysis was also made on the whole cohort comparing the patients receiving at least 1 day of continuous Ketamine infusion to all patients who did not fulfill this criterion.

**Results:** 66 cases were identified and matched to 66 controls. In the Ketamine group, the median Ketamine duration was 5 (3–8) days, and median total cumulative dose 18.6 (9.6–42.5) g. The occurrence of LD was higher in the Ketamine group than in the matched control group (53.8% versus 28.6%, p = 0.002, Fig. 1). The Hazard ratio (HR) for LD in the Ketamine group was 2.03 (95% CI 1.15–3.59, p = 0.015). There was an increased risk of LD of 5.9% per day of exposure to Ketamine (HR 1.06, 95% CI 1.02–1.1 p = 0.003) and of 0.8% per gram of Ketamine infused (HR 1.01, 95% CI 1.00–1.01, p = 0.006), with a risk starting to be statistically significant after 4 days and 18gr. In multivariate analysis on the whole cohort, Ketamine exposure (HR 2.49, 95% CI 1.40–4.44, p = 0.02), cumulative dose in gram (HR: 1.02, 95% IC: 1.01–1.04, p = 0.002) and ketamine exposure in days (HR: 1.16, 95% IC: 1.07–1.25, p < 0.001) remained independent risk factors for LD occurrence.

**Conclusion:** Ketamine use in critically ill patients treated for ARDS is associated to a higher risk of liver dysfunction, assessed by total serum bilirubin. This risk is dose-dependent and increases with duration of treatment. The prescription of high doses or prolonged treatment with Ketamine should probably be avoided in critically ill patients.

**Compliance with ethics regulations:** Yes.

**Fig. 1:**
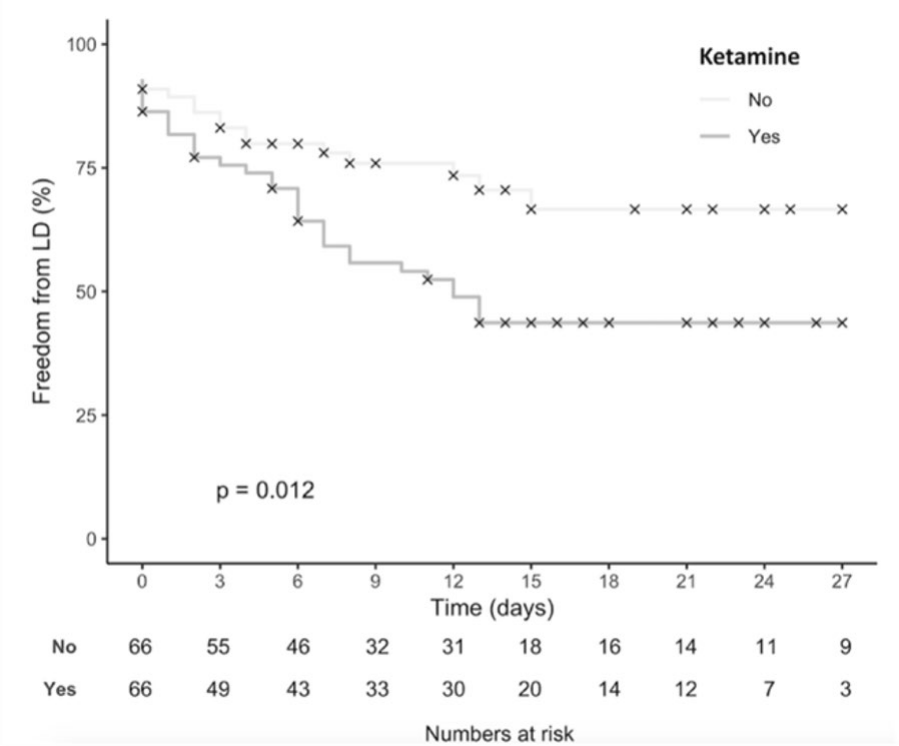
Kaplan–Meier Curves of Probability of Liver Dysfunction (LD) free days according to Ketamine exposure in the matched cohort

### F-064 Cardiovascular complications following Ciguatera fish poisoning in the French West Indies—A case series

#### Dabor Resiere^1^, Florentin Jonathan^2^, Mahi Zacharia^2^, Papa Gueye^2^, Hatem Kallel^3^, Hossein Mehdaoui^1^

##### ^1^Department of Critical Care, University Hospital of Martinique, Fort-De-France, France; ^2^University Hospital of Martinique, Fort-De-France, France; ^3^Intensive Care Unit, Cayenne General hospital, Cayenne, 97300, French Guiana, France

###### **Correspondence:** Dabor Resiere (dabor.resiere@chu-martinique.fr)

*Ann. Intensive Care* 2020, **10 (Suppl 1):**F-064

**Rationale:** Ciguatera is one of the most common cases of marine poisoning associated with fish consumption in the world. The incidence of this intoxication is largely unreported. In Martinique, the incidence of this intoxication seems constantly increasing. During the last 3 years, numerous cases of large collective poisonings have been reported in Martinique, especially during summer. The spectrum of clinical manifestations is large including gastrointestinal, neurological and cardiovascular symptoms. Ciguatoxin, the toxin responsible for ciguatera fish poisoning is considered as a sodium channel agonist with cholinergic and adrenergic activity. It is rarely fatal and management of poisoned patients is essentially based on supportive care. The objective of this study was to describe the clinical characteristics and complications of ciguatera poisoning in Martinique, focusing on the cardiovascular ones.

**Patients and methods:** Observational, retrospective, single—center study covering six-year period from October 2012 to September 2018, including all patients admitted to the Emergency Department of the University Hospital of Martinique (CHU), and all patients who were declared to the Regional Health agency (ARS) for ciguatera intoxication.

**Results:** One hundred and forty-nine patients (149) who were ciguatera-affected were included. The incidence rate found was to be 0.67 cases per 10.000 patient-years in Martinique over the period. About 90% of patients had gastrointestinal symptoms such as nausea, vomiting, diarrhea, or abdominal pain; 83% neurological disorders and 42% cardiovascular symptoms including, bradycardia, hypotension and interventricular block. Ingestion of Carangue fish was related to a major risk of chronic signs.

**Conclusion:** The incidence of ciguatera in Martinique is increasing, with 0.67 cases/10.000 patient-years. The clinical presentation is defined mainly by digestive signs, followed by peripheral neurological disorders and cardiovascular symptoms. Ciguatera fish poisoning in Martinique presents similar clinical presentation to that of the other Caribbean Islands. There is no specific treatment. Acute ciguatera poisoning is responsible for significant cardiovascular complications. Physicians should be aware of the potential cardiovascular risk of ciguatera poisoning.

**Compliance with ethics regulations:** Yes.

**Reference**

1. Friedman MA, Fleming LE, et al. Ciguatera fish poisoning treatment, prevention and management. Mar Drugs 2008; 6:456

### F-065 Acute organophosphate poisoning

#### Fahd Moussaid, Reda Elhadrami, Hamza Elhamzaoui, Taoufik Abouelhassan

##### Emergency department, University Hospital Mohamed VI, Marrakec, MOROCCO

###### **Correspondence:** Fahd Moussaid (fahdmoussaid5@gmail.com)

*Ann. Intensive Care* 2020, **10 (Suppl 1):**F-065

**Rationale:** Pesticides have represented the most incriminated products in severe acute poisonings, in the developing countries, due to the availability of these products. Organophosphate poisoning accounts for 3 million poisonings/year worldwide. Organophosphate (OP) pesticides are used mainly as insecticides in agriculture. the Moroccan anti-poison and pharmacovigilance centrer shows that OP poisoning are responsible for 13% of all poisonings combined. The aim of our study: epidemiological, clinical, management and prognostic factors.

**Patients and methods:** A retrospective study was conducted on patients with OP poisoning admitted to our nine-bed medical intensive care unit between January 2018 and December 2018. inclusion criteria were: all patients over 16 years of age and the exlusion criteria were: pesticide poisoning other than OP, alcohol poisoning, drug poisoning, scorpionic poisoning and snake bites. statistical analysis was performed with SPSS software.

**Results:** Forty patients were admitted for acute OP poisoning. In Morocco, organophosphores are available over-the-counter in several forms: Rodentocides, Malathion, Cockroach trap, Baygon Insecticide (Fig. 1). The average age was 26 years with a female prévalence of 74.1%. The intoxications were mostly intentional (85%). The symptomatology was determined by the three syndromes: central syndrome in 50%, muscarinic syndrome in 69%, nicotinic syndrome in 10%. Rhythm disorders in 8%, and cardiovascular collapse in 9%. The symptomatic treatment was applied to all patients, antidotic treatment was administered in 80% of patients. The average length of hospitalization was 06 days.

**Conclusion:** Acute OP poisoning is a real public health problem. Its associated symptomatic treatment (respiratory and neurological resuscitation) and antidotic treatment. The mortality remains high in our context, therefore, we must attach great importance to the prevention.

**Compliance with ethics regulations:** Yes.

**Table 1:** The distribution of the acute organophosphate poisonings according to the responsible agent

OP	Patient percentage
Rodenticides	80%
Malathion	5%
Cockroach trap	10%
Baygon pesticide	5%

### F-066 Long-term exposure to Sargassum-seaweed pollution in the French Caribbean Islands: clinical consequences and outcome

#### Dabor Resiere^1^, Ruddy Valentino^1^, Rishika Banydeen^2^, Jonathan Florentin^1^, Papa Gueye^1^, Alain Blateau^3^, Hatem Kallel^4^, Bruno Megarbane^5^, Remi Neviere^1^, Hossein Mehdaoui^1^

##### ^1^Department of Critical Care, University Hospital of Martinique, F-97200 Fort-de-France, France; ^2^Department of Epidemiology & Methodology, University Hospital of Martinique, F-97200 Fort-de-France, France; ^3^ARS Martinique, Fort-De-France, France; ^4^Intensive Care Unit, Cayenne General Hospital, Cayenne, 97300 French Guiana, France; ^5^Department of Medical and Toxicological Critical Care, Lariboisière Hospital, Paris-Diderot University, INSERM UMRS1144, Paris, France

###### **Correspondence:** Dabor Resiere (dabor.resiere@chu-martinique.fr)

*Ann. Intensive Care* 2020, **10 (Suppl 1):**F-066

**Rationale:** Since the beginning of 2018, there has been an unexplained increasing invasion of Sargassum on the coast of Caribbean countries, including Martinique, Guadeloupe (1). Over an 8-month period, health officials in Guadeloupe and Martinique reported more than 12.000 such cases. Assault of these brown algae represents not only an environmental and economic disaster, but also a threat for human health. After 48 h on seashore, large amounts of toxic gas are produced by matter decomposition, including hydrogen sulfide (H2S) and ammoniac (NH3). The acute effects on humans after exposure to high concentrations of H2S are well described and of increasing severity with concentration, leading to potentially fatal hypoxic pulmonary, neurological and cardiovascular injuries (Table 1); however, the association of long-term exposure to Sargassum and health events is unknown. Although less documented, long term exposures may result in conjunctiva and upper airways irritation, headaches, vestibular syndrome, memory loss, and modification of learning abilities. In the absence of any available antidote, management of H2S intoxication relies on supportive care and prevention using individual protection. The objective of this study was to evaluate the clinical characteristics and consequences of long-term exposure to Sargassum among the local population.

**Patients and methods:** We conducted a prospective observational cohort study including all patients admitted to the emergency department at the University hospital of Martinique from March 2018 to December 2018 due to exposure to sargassum. Patients were managed according to the protocol established by the Research Group on Sargassum in Martinique. We assessed the patients exposure to Sargassum and air pollutants using 14 monitor located near of the patient’s residence. Demographics and clinical data (including cardiovascular, neurological and respiratory events) were collected. Data are presented as mean ± SD or %. Comparisons were performed using univariate analysis.

**Results:** In 8 months, 160 patients were included (age: 48 ± 20 years, 54 M/146 W, past history: hypertension (N = 25), diabetes (N = 29), asthma (14). Patients arrived with referral letter from their general practitioner (80%) and presented headaches (76%), developed gastrointestinal disturbances (79%), dizziness (54%), skin lesions (30%), cough (44%) and conjunctivitis (33%). Not all patients were clinically symptomatic. In the patients presented in June (14%), symptoms more frequently occurred in the workplace or at home (p < 0.05). Initial lung function tests were normal (50%). Three patients were admitted in intensive care unit.

**Conclusion:** Our study indicates that the magnitude of health effects following long-term exposure to Sargassum may be larger than previously recognized. Efforts to limit long-term exposure are mandatory.

**Compliance with ethics regulations:** Yes.

**Table 1:**
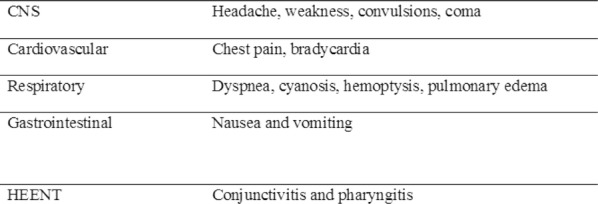
Clinical Characteristics of acute hydrogen sulfide poisoning

### F-067 Prognosis value of acute liver dysfunction after out-of-hospital cardiac arrest

#### Marie-Charlotte Delignette^1^, Thomas Baudry^1^, Marie Simon^1^, Flavie Lavigne^1^, Eric Bonnefoy-Cudraz^2^, Laurent Argaud^1^, Martin Cour^1^

##### ^1^Médecine Intensive Réanimation-Hôpital Edouard Herriot, Lyon, France; ^2^USIC-Hôpital Louis Pradel, Lyon, France

###### **Correspondence:** Marie-Charlotte Delignette (marie-charlotte.delignette@chu-lyon.fr)

*Ann. Intensive Care* 2020, **10 (Suppl 1):**F-067

**Rationale:** Liver consequences of out-of-hospital cardiac arrest (OHCA) have been poorly studied. The aim of this study was to describe the characteristics of OHCA-induced acute liver dysfunction and its association with outcomes.

**Patients and methods:** We analyzed all consecutive OHCA patients admitted to two academic centers between 2007 and 2017. Patients treated with vitamin K antagonist were not included. Acute hepatocellular insufficiency (AHI), liver failure (LF) and hypoxic hepatitis (HH) were defined as a prothrombin (PT) ratio < 50%, a hepatic SOFA sub-score > 2 and an increase in transaminases > 20 times the normal values, respectively. Indocyanine green (ICG) clearance was used as the reference measure of liver function in a subset of patients. Multivariate logistic regression was used to identify potential risk factors for day 28 mortality.

**Results:** Of the 418 patients included (median age: 64 years; sex ratio: 1.4; nonshockable OHCA: 73%), 67 (16%), 3 (0.7%) and 61 (14.6%) presented AHI, LF and HH at admission, respectively. Death occurred before day 28 in 337 (80.6%) patients. Among the conventional static function liver tests, only bilirubin did not significantly differ between patients who survived or died (p = 0.91). ICG clearance, available in 23 patients, was abnormal in 17 (73.9%) cases. PT ratio was the only static test of liver function correlating significantly with ICG clearance (r = − 0.66, p < 0.01). After multivariate analysis, PT < 50% at admission (OR 10.63; 95% IC [1.92–200.9]), alkaline phosphatase at admission ≥ 100 UI/L (OR 5.26; 95% CI [2.29–13.23]), OHCA occurring at home (OR 3.83; 95% IC [1.85 8.11]), absence of bystander cardiopulmonary resuscitation (OR 2.94; 95% IC [1.40–6.39]), non-cardiac cause of OHCA (OR 5.22; 95% IC [2.48–11.43]) and low flow duration ≥ 20 min (OR 7.26; 95% IC [3.28–17.45]) were independently associated with day 28 mortality. As shown in Fig. 1, survival significantly differed according to the degree of decrease in PT ratio (p < 0.0001, log rank test).

**Conclusion:** Early AHI, as defined by PT < 50% at ICU admission, occurred frequently after OHCA and was independently associated with day 28 mortality.

**Compliance with ethics regulations:** Yes.

**Fig. 1:**
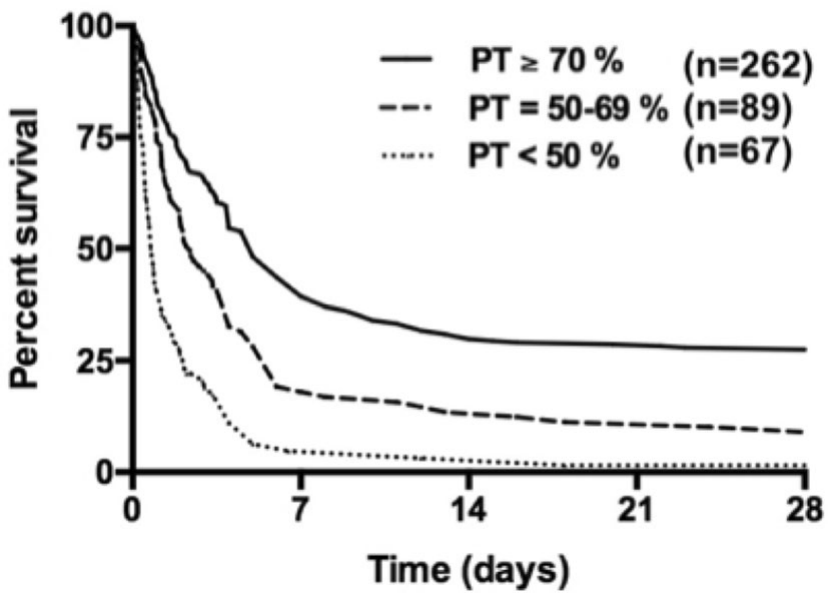
Survival according to PT ratio at ICU admission

### F-068 Using neuron-specific-enolase (NSE) for prediction of severe brain damage after cardiac arrest: insights from the Parisian Registry

#### Guillaume Savary^1^, Antonin Ginguay^2^, Phuong-Nhi Bories^2^, Paul Jaubert^1^, Ariane Gavaud^1^, Elodie Baron^1^, Pierre Dupland^1^, Jean-Paul Mira^1^, Florence Dumas^3^, Alain Cariou^1^

##### ^1^Medical Intensive Care Unit, Cochin Hospital, APHP, Centre—Université de Paris, Paris, France; ^2^Biochimie, Cochin Hospital, APHP, Centre—Université de Paris, Paris, France; ^3^Emergency Department, Cochin University Hospital, APHP, Paris, France

###### **Correspondence:** Guillaume Savary (guillaume.savary@aphp.fr)

*Ann. Intensive Care* 2020, **10 (Suppl 1):**F-068

**Rationale:** Neuron-specific-enolase (NSE) is commonly used as a biomarker reflecting the extent of brain injury in different settings. In post-cardiac arrest patients, previous clinical studies reported that an increase in NSE was predictive of a poor outcome but did not specifically focused on neurological outcome. In this prospective study, we aimed to determine the NSE performance for prediction of severe brain damage in post-cardiac arrest patients.

**Patients and methods:** All consecutive patients admitted in our ICU after cardiac arrest between January 2017 and February 2019 that were still comatose at H48 and had at least one measurement of serum NSE were included. Blood samples for NSE measurement were serially collected at 48 (H48) and 72 h (H72) after cardiac arrest and serum NSE levels were measured within 4 h. We used the following criteria for the definition of severe brain damage (primary endpoint): Cerebral Performance Categories (CPC) 3 or 4 level at discharge, brain death or withdrawal of life-sustaining treatments (WLST) based on neurological status. We also assessed the predictive value of serum NSE using all-cause mortality as a secondary endpoint.

**Results:** During the study period, 155 patients were available for the analysis. They were mostly male (66.9%), with an age of 60.6 years. Among these patients, 83 (50.3%) had a good neurologic outcome (CPC 1–2) and 68 patients were classified as having a severe brain damage (52 WLST based on neurological status, 11 brain deaths and 5 survivors with CPC 3–4). In univariate analysis, patients with severe brain damage less frequently received bystander CPR, had longer duration of no-flow, less initial shockable rhythm, more post-resuscitation shock and higher NSE values: mean at H48 were 217.3 versus 26.2; and 270.1 versus 21.4 at H72 (p < 0.001). NSE levels at H48 and H72 were strong predictors of severe brain damage (AUC of 0.948 and 0.972 respectively, Figure 1) and also predicted all-cause mortality (AUC of 0.896 and 0.931 respectively). To predict severe brain damage with 100% specificity, best NSE cutoff values at H48 and H72 were 52.3 and 53.2 µg/l, with a sensitivity of 77.6 and 82.9% respectively.

**Conclusion:** A high serum NSE measured at H48 and H72 after cardiac arrest accurately predicted severe brain damage with a high specificity. Our results support the use of NSE for neuroprognostication after cardiac arrest, in combination with other predictors.

**Compliance with ethics regulations:** Yes.

**Fig. 1:**
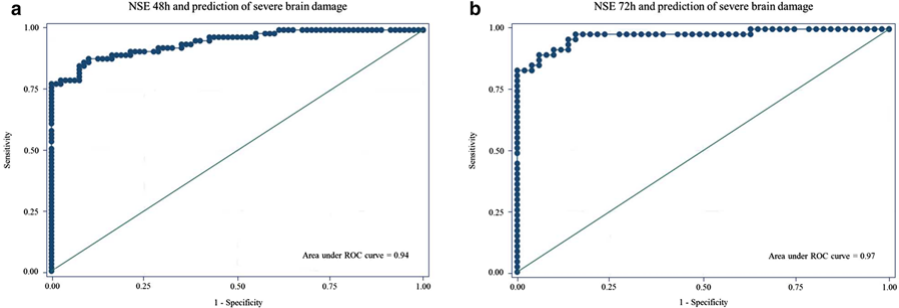
NSE Receiver-Operating curves

### F-069 Availability of psychologists in French-speaking intensive care units

#### Jean-Baptiste Lascarrou^1^, Saber Davide Barbar^2^, Florence Boissier^3^, Guillaume Decormeille^4^, Nicholas Heming^5^, Bertrand Hermann^6^, Sami Hraiech^7^, Gwenaëlle Jacq^8^, Jean-FrançOis Llitjos^6^, Lamia Besbes^9^, Laurent Poiroux^10^, Xavier Monnet^11^, Gaël Piton^12^

##### ^1^Medical Intensive Care Unit, Nantes, France; ^2^Medical Intensive Care Unit, Nîmes, France; ^3^Medical Intensive Care Unit, Poitiers, France; ^4^Medical Surgical Intensive Care Unit, Toulouse, France; ^5^Medical Intensive Care Unit, Garches, France; ^6^Medical Intensive Care Unit, Paris, France; ^7^Medical Intensive Care Unit, Marseilles, France; ^8^Medical Surgical Intensive Care Unit, Versailles, France; ^9^Medical Intensive Care Unit, Monastir, Tunisia; ^10^Medical Intensive Care Unit, Angers, France; ^11^Medical Intensive Care Unit, Le Kremlin-Bicêtre, Francel; ^12^Medical Intensive Care Unit, Besançon, France

###### **Correspondence:** Jean-Baptiste Lascarrou (jeanbaptiste.lascarrou@chu-nantes.fr)

*Ann. Intensive Care* 2020, **10 (Suppl 1):**F-069

**Rationale:** The psychological care of patients, their relatives and of healthcare workers is a major issue in the intensive care unit (ICU). Psychologists may provide emotional support during trying times. The intervention of a psychologist may alleviate long term mental health issues such as post-traumatic stress disorder. The main objective of our study was to describe the availability of psychologists in French-speaking ICUs.

**Patients and methods:** Internet survey conducted between March and May 2019 using SurveyMonkey (San Mateo, USA). Survey consisting of 20 questions sent to subscribers of the SRLF mailing list via MailChimp software (Atlanta, USA). Frequencies and percentages were determined for categorical variables and median and interquartile range for continuous variables. The ICUs with or without psychologist were compared using nonparametric Fisher exact test. STATA 14 used (Lakeway Drive, TE, USA).

**Results:** 263 responses were obtained from 197 unique ICUs in France (n = 182), Belgium (n = 6), Switzerland (n = 5), Algeria (n = 2), Morocco (n = 1) and Tunisia (n = 1). 164 (82%) ICUs were part of public hospitals, 33 (18%) of private facilities. 187 (94%) ICUs cared for adult patients, 10 (6%) for children. The median number of beds was 20 [15–26]. 79 (40%) ICUs were open to visitors 24/7, 46 (23%), to visitors > 8 h/day and 72 (37%) to visitors < 8 h/day. Psychological consults were established in 89 (45%) wards (2 ICUs did not answer). Pediatric ICUs employed more psychologists than adult ICUs (P = 0.006). Comparison of ICUs based on the presence or not of a psychologist appears in Table 1. In ICUs where a consulting psychologist is available, their effective availability is 0.5 [0.2–1] full time equivalent. Consults are delivered to: patients (100%), families (100%) or healthcare workers (60%). Out of the 106 ICUs without a psychological consult, responders from 5 (2.5%) ICUs believe that a psychological consult is undesirable. Out of the 106 ICUs without psychological consult, 24 (22%) responders cannot obtain a psychological consult, whatever the circumstances, 41 (39%) can require an outside psychological consult when needed, while 41 (39%) can require assistance from a psychologist working in another unit (several answers possible for each respondent).

**Conclusion:** Psychologists consult in only half of adult ICUs but in almost all pediatric ICUs. 22% of ICUs are unable to provide a psychological consult. Psychological consults are delivered in similar proportions to patients, their family and to a lesser extent to healthcare workers. Responders from 2.5% ICUs without an established psychological consult believe that the availability of a psychologist is undesirable.

**Compliance with ethics regulations:** NA.

**Table 1:** .

	n = 197 ICUs	P
Presence of a psychologist	89 (54%)	
Presence of a psychologist		
Public hospital	71/162 (44%)	0.34
Private hospital	18/33 (54%)	
Unknown	2	
Presence of psychologist		
Adult ICU	80/185 (43%)	0.006
Pediatric ICU	9/10 (90%)	
Presence of a psychologist according to the opening of the ICU		
< 8 h	39/79 (49%)	0.58
> 8 h	21/45 (46%)	
24 h	29/71 (41%)	

### F-070 Should Intermediate care units and intensive care units be 2 separate units?

#### Juliette Audibert, Adel Ben Salah, Alexandre Conia, Olivier Gontier, Mouldi Hamrouni, Cecile Jourdain, Benedicte Mauchien, Pierre Kalfon

##### Hôpitaux de Chartres, Chartres, France

###### **Correspondence:** Juliette Audibert (juliettesouhaid@gmail.com)

*Ann. Intensive Care* 2020, **10 (Suppl 1):**F-070

**Rationale:** Comfort of patients in Intensive Care Unit (ICU) is now a real concern for the healthcare teams. Perceived patient discomfort assessment is a daily practice for our staff. The primary objective of our study was to assess whether the overall discomfort score reported by patients hospitalized in a separate intermediate care unit differs from that reported by patients hospitalized in ICU.

**Patients and methods:** A tailored multicomponent program consisting of assessment of ICU-related self-perceived discomforts with a 18-item questionnaire, immediate and monthly feedback to healthcare teams and site-specific tailored interventions, was applied in our department, located in a general hospital, and comprising a 12-bed ICU and a separate 6-bed intermediate care unit. From May 1, 2018 to June 30, 2019, all patients, who were hospitalized in the ICU or in the intermediate care unit, aged 18 years or older, who survived a stay of 3 calendar days or more, were eligible for inclusion and were asked to answer the 18-item questionnaire.

**Results:** During this period of 13-months period, 173 ICU patients whereas 163 intermediate care unit patients were included in the study with complete questionnaire. Age and sex ratio were similar between the 2 groups whereas the length of stay and IGSII were higher in ICU. The overall discomfort score was higher in the ICU group than in the intermediate care unit group (23 ± 15 vs. 18 ± 11, p = 0.005) as well as scores for thirst (3.43 ± 3.61 vs. 2.53 ± 3.53, p = 0.016), feeling of cold (2.31 ± 3.18 vs 0.70 ± 1.81, p < 10–3), dyspnea (3.43 ± 3.52 vs. 2.07 ± 2.76, p = 0.001), lack of phone (1.50 ± 2.86 vs. 0.43 ± 1.57, p < 10–3), isolation (1.70 ± 2.80 vs. 0.99 ± 2.18, p = 0.020) and depression (2.39 ± 3.23 vs. 1.34 ± 2.49, p = 0.002). In the separate intermediate care unit, the only 2 following items reported with higher scores than in the ICU were feeling of heat (2.01 ± 3.13 vs. 0.96 ± 2.18, p = 0.001), probably because of the absence of air conditioner, and bed-related discomfort (2.63 ± 3.18 vs. 1.90 ± 2.97, p = 0.009).

**Conclusion:** Overall discomfort score was lower in our separate intermediate care unit than in our ICU. In the future, intermediate care units and intensive care units should be 2 separate units to ensure maximum comfort for our patients.

**Compliance with ethics regulations:** Yes.

### F-071 Evaluation of an early post-ICU discharge consult program on handoff quality

#### Camille Vissac^1^, Cécilia Tabra^1^, Rusel Leon^1^, Marina Axus^1^, Morgane Commereuc^1^, Frédérique Schortgen^1^, Jerome Cecchini^1^

##### ^1^Réanimation et surveillance continue adulte, Hôpital intercommunal de Créteil, Créteil, France

###### **Correspondence:** Camille Vissac (camille.vissac@chicreteil.fr)

*Ann. Intensive Care* 2020, **10 (Suppl 1):**F-071

**Rationale:** The transition period surrounding the discharge from ICU to hospital ward is a critical period in the course of the patient. Handoff of complex patients is at high risk for communication failures between providers, inaccurate cares and ICU readmission. A transition program including a post ICU follow-up has been proposed to improve handoff quality. Post ICU consults by ICU team represent, also, an opportunity for improving feedback on the quality of ICU cares. The goal of the present study is to assess the feasibility and the impact of a systematic early post-ICU consult (EPICUC) program on handoff quality in a 14 bed mixed ICU.

**Patients and methods:** Before the development of the EPICUC program, standardized handoffs were already applied including identified day and hour of discharge and both verbally communicate and written medical and nurse information for receiving team. From 1st March to 30th October 2019, all patients who were discharged to the ward of our hospital were candidates for EPICUC. EPICUC were performed by ICU staff (at least one ICU physician) within the 3 days following discharge. The EPICUC consisted of a face-to-face discussion with the receiver team to assess the accuracy, completeness and understanding of passing information and of a patient visit. A standardized form was used for collecting data. The impact of EPICUC on handoff quality was assessed by the number of communication failures and the number of patients in whom EPICUC resulted in a management change. Personal feeling of EPICUC providers on its usefulness was assessed by a 0–10 rating scale.

**Results:** Among the 209 candidates for EPICUC, 4 were dead and 74 already discharged alive from hospital at EPICUC time. EPICUC were performed in 131 patients (63%) within 4 ± 3 days after ICU discharge. 85 EPICUC (65%) were performed by both, nurse and ICU physician. 111 (85%) patients and 84 receiver teams (64%) were available at EPICUC time. EPICUC duration was 11 ± 4 min. A communication failure was identified in 38 EPICUC (29%), either a rectification of passing information (n = 30; 23%) and/or a change in patient management (n = 19; 15%). The usefulness of the EPICUC was rated at 4 ± 3 and 5 ± 3 by ICU physicians and nurses, respectively.

**Conclusion:** The time spent for EPICUC appears reasonable. EPICUC identified a communication failure in one-third of handoffs and allowed care readjustment in one quarter of patients. Factors associated with handoff failures will be presented during the congress.

**Compliance with ethics regulations**: Yes.

### F-072 Patients’ perceptions of quality of life after ICU discharge

#### Danielle Prevedello, Claire Steckelmacher, Marianne Devroey, Jean-Charles Preiser

##### Hopital Erasme, Bruxelles, Belgium

###### **Correspondence:** Danielle Prevedello (danielle.prevedello@gmail.com)

*Ann. Intensive Care* 2020, **10 (Suppl 1):**F-072

**Rationale:** Surviving a critical illness is a challenging condition for patients and relatives. The psychological aspects are directly affected by physical status and performance. Patients can feel depressed or anxious facing difficulties during recovery time. The aim of this study was to correlate patients’ perceptions of his health status and his clinical performance measured after ICU discharge.

**Patients and methods:** This is a prospective pilot study of an ICU follow-up clinic conducted in a single center from January 2018 to July 2019. This clinic is multidisciplinary and includes two visits at 3 and 6 months after ICU discharge. Patients with more than 5 days of ICU LOS were eligible. All patients at 3 and 6-m visit were evaluated with SF-36, 6MWT, MRC and time-up-and-go test. We conducted an analysis comparing clinical performance data and qualitative data between 3 and 6 months after ICU discharge.

**Results:** The investigation included 90 patients who had at least 5 days of ICU length of stay. 50 patients attended the consult at 3-m and 28 patients attended the consult both times. The median age (IQR) was 63 (54–71) and 56% were men. 48%, 30% and 22% of patients had medical, scheduled surgical and emergency surgical admission causes respectively, with median (IQR) SAPS III score 53 (44–73). 38%, 14% and 80% of patients had sepsis, delirium and mechanical ventilation as a support. The physical status was progressively increased overtime likewise the physical capacity assessed by SF-36 score with p-value 0.007 between 3 and 6-m. However, no significant difference between the subjective dimension of SF-36, which analyses the perception of the patient about his physical capacity, assessed at 3-m and at 6-m was demonstrated (p 0.35).

**Conclusion:** In this pilot-phase of following a cohort of critically ill patients, the natural physical improvement does not seem to change the patient’s perception of their performances. This paradigm rouses a different perspective that should take into account when setting up rehabilitation programs.

**Compliance with ethics regulations**: Yes.

### F-073 Post-traumatic stress disorder after discharge from an acute medical unit

#### Basma lahmer^1^, naoufel madani^1,2^, jihane belayachi^1,2^, redouane abouqal^1,2^

##### ^1^Acute Medical Unit, Ibn Sina University Hospital, Rabat, Morocco; ^2^Laboratory of Biostatistics, Clinical, and Epidemiological Research, Faculty of medicine and pharmacy—university Mohammed V, 10000, Rabat, Morocco

###### **Correspondence:** Basma lahmer (basma.lahmer.93@gmail.com)

*Ann. Intensive Care* 2020, **10 (Suppl 1):**F-073

**Rationale:** Post-Traumatic Stress Disorder (PTSD) occurs after exposure to a traumatic event and comprises of symptoms of repeated re-experiencing of the said event, avoidance of reminders, emotional numbing and persistent hyperarousal. In individuals exposed to “medical stress”, various studies found evidence of PTSD occurring after the onset, diagnosis, or treatment of physical illness. Our study aims to determine PTSD’s risk factors in patients of an acute medical unit (AMU) after their discharge.

**Patients and methods:** It was a prospective, analytical study conducted over a period of 2 months at an acute medical unit. We collected sociodemographic and clinical data, patients’ medical history, and evaluated the symptoms of anxiety and depression during their stay using the Hospital Anxiety and Depression Scale (HADS). The prevalence of severe PTSD symptoms was assessed with the Impact of Events Scale-Revised (IES-R) at 6 weeks and 3 months using a cutoff of 33. Associations between PTSD as evaluated by IES-R at 3 months and patients’ characteristics, including HADS scores at admission were investigated using unadjusted linear regression, for univariate and multivariate regression analysis. Statistical analyses were carried out using SPSS for Windows (SPSS, Inc., Chicago, IL, USA).

**Results:** We included 141 patients in our study with a mean age of 49.74 ± 17.8. In our population, 20.8% of patients scored higher than a 33 IES-R cutoff at 6 weeks compared to 17.6% at 3 months. The mean HADS-Anxiety score is 7.8 ± 6 and that of the HADS-Depression score is 7.1 ± 6.3. On one hand, higher HADS-anxiety Score during the stay in the AMU was linked to higher IES-R scores at 3 months β: 1.47 IC95% (0.61; 2.26) p < 0.001. On the other hand, living alone but with family in close proximity β: − 14.1 IC95% (-25.22; − 2.97) p = 0.01 and a longer stay in the AMU β: − 0.37 IC95% (− 0.71; − 0.03) p = 0.03 were found to be predictive of less severe PTSD symptoms.

**Conclusion:** We identified that patients who had shown less significant anxiety symptoms during their stay, lived alone with family nearby and stayed longer in the AMU were less at risk of developing PTSD after their discharge.

**Compliance with ethics regulations**: Yes.

### F-074 Quadriceps strength of critically ill patients: how weak are they?

#### Anne Meunier, Jean-Louis Croisier, Isabelle Kellens, Benoit Misset, Jean Joris, Anne-Françoise Rousseau

##### University Hospital of Liège, Liège, Belgium

###### **Correspondence:** Anne Meunier (anne.meunier@student.uliege.be)

*Ann. Intensive Care* 2020, **10 (Suppl 1):**F-074

**Rationale:** Objective of critical care includes restoration of functional capacities. Prompt identification of muscle acquired weakness (ICU-AW) is crucial to target efficient rehabilitation. In published literature, data of quadriceps strength (QS) cannot be compared because of insufficient standardization of measurement protocols. We recently validated a highly standardized protocol of QS measurement. In order to build basic and comparable knowledge and to identify the weakest patients, this study aimed to describe QS of critically ill (CI) patients during their short-term evolution, and to compare them to surgical (S) and healthy (H) subjects.

**Patients and methods:** This observational study included CI patients who spent at least 2 days in ICU, patients scheduled for elective colorectal surgery (S) and young healthy volunteers (H). Maximal isometric QS was assessed using a handheld dynamometer (MicroFet2^®^) and expressed in Newton/kg (N/kg). Dominant leg was tested in supine position using a highly standardized procedure. CI and S patients were tested at T0 (as soon as collaborative in ICU) and 1 month after discharge (M1). Medical Research Council (MRC) test and Barthel questionnaire were used at same time points. Data are expressed as median (min–max).

**Results:** 38, 32 and 34 subjects were included in CI, S and H groups, respectively. Demographic data (age, sex, body mass index) were similar in CI and S groups. At T0, QS was lower in CI group than in S and H groups: 3.01 (0.77–7.61), 3.6 (2.26–5.98) and 5.5 (3.19–7.96) N/kg, respectively. At T0, only 13 CI patients (34%) had a MRC < 48 (definition of ICU-AW) but their QS was not lower than those with MRC ≥ 48. QS did not improve at M1 in CI patients, especially if previously sedentary (Fig): QS was 2.96 (1.32–5.33) N/kg. CI patients with QS < 2.1 N/kg (i.e. the weakest QS in S patients) remained more dependent at M1 than those with QS ≥ 2.1 N/kg: Barthel was 87.5 (30–100) vs 100 (40–100), respectively (p = 0.0103). QS at M1 in CI group was significantly associated with QS at T0 (r = 0.67, p < 0.0001) and inversely correlated with age (r = − 0.39, p = 0.0173).

**Conclusion:** Muscle function is a key component of post-ICU recovery. A huge QS heterogeneity was observed among CI patients. QS in ICU was correlated with QS at M1, but was not consistent with ICU-AW diagnosis based on MRC. Special attention should probably be paid to CI patients previously sedentary and to patients with QS < 2.1 N/kg: they were weaker or more dependent at M1.

**Compliance with ethics regulations**: Yes.

**Fig. 1:**
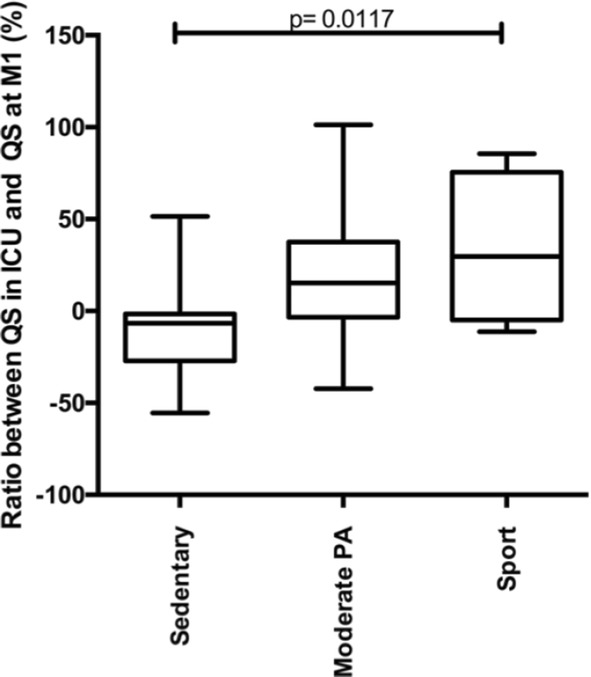
QS change at M1 in CI patients according to previous physical activity

### F-075 Healthcare trajectories before and after critical illness: population-based insight on diverse patients clusters

#### Youenn Jouan^1^, Leslie Grammatico-Guillon^2^, Antoine Guillon^1^, Stephan Ehrmann^1^

##### ^1^Service de Médecine Intensive Réanimation, CHU de Tours, Tours, France; ^2^Service d’Information Médicale, d’Epidémiologie et d’Economie de la Santé, Tours, France

###### **Correspondence:** Youenn Jouan (youenn.jouan@gmail.com)

*Ann. Intensive Care* 2020, **10 (Suppl 1):**F-075

**Rationale:** The post intensive care syndrome (PICS) gathers various disabilities, associated with a substantial healthcare use. However, patients’ comorbidities and active medical conditions prior to intensive care unit (ICU) admission may partly drive healthcare use after ICU discharge. To delineate the relative contribution of critical illness and PICS per se to post-critical illness increased healthcare use, as opposed to pre-existing comorbidities, we conducted a population-based evaluation of patients’ healthcare use trajectories.

**Patients and methods:** Using discharge databases in a 2.5-million-people region in France, we retrieved, over three years, all adult patients admitted in ICU for septic shock or acute respiratory distress syndrome (ARDS), intubated at least 5 days and discharged alive from hospital. Healthcare use (days spent in healthcare facilities) was analyzed two years before and two years after ICU admission. Healthcare trajectories were next explored at individual level: patients were assembled according to their individual pre-ICU healthcare use trajectory by clusterization with the K-Means method.

**Results:** Eight-hundred and eighty-two (882) patients were included. Median duration of mechanical ventilation was 11 days (interquartile ranges [IQR] 8; 20), mean SAPS2 was 49, and median hospital length of stay was 42 days (IQR 29; 64). Prior to ICU admission, we observed, at the scale of the whole study population, a progressive increase in healthcare use. However, clusterization of individual according to pre-ICU healthcare trajectories identified patients with elevated and increasing healthcare use (n = 126), and two main groups with low (n = 476) or no (n = 251) pre-ICU healthcare use. Patients with high healthcare use had significantly more comorbidities than those with low healthcare use. In ICU, however, SAPS2, duration of mechanical ventilation and length of stay were not different across the groups. Interestingly, analysis of post-ICU healthcare trajectories for each group revealed that patients with low or no pre-ICU healthcare (which represented 83% of the population) switched to a persistent and elevated healthcare use during the two years post-ICU.

**Conclusion:** For 83% of ARDS/septic shock survivors, critical illness appears to have a pivotal role in healthcare trajectories, with a switch from a low and stable healthcare use prior to ICU, to a sustained higher healthcare recourse two-years after ICU discharge. This underpins the hypothesis of long-term critical illness and PICS-related quantifiable consequences in healthcare use, measurable at a population level.

**Compliance with ethics regulations**: Yes.

**Fig. 1:**
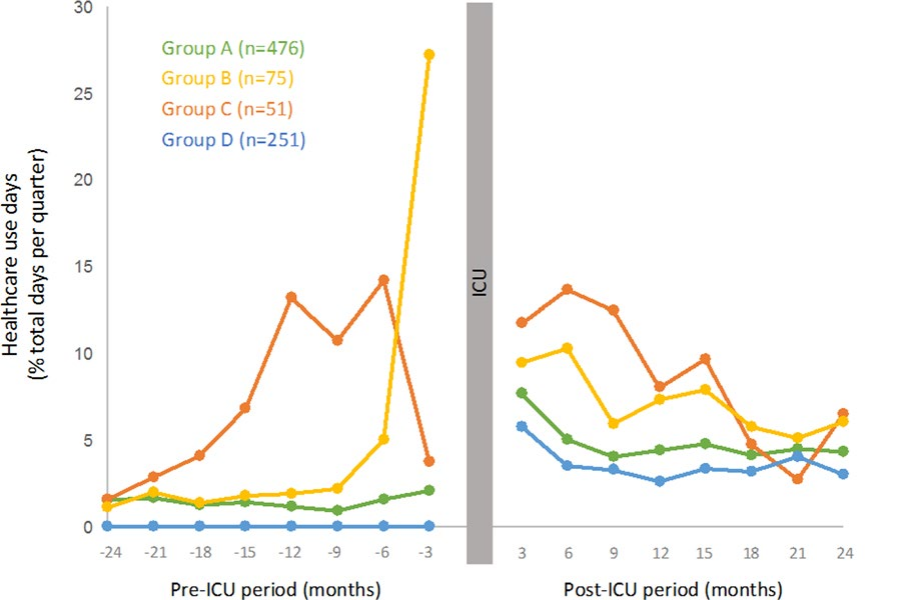
Healthcare use days (% of total days per quarter spent in acute care settings) during the pre-ICU period and the post-ICU period (B) after clustering based on total pre-ICU healthcare use

### F-076 Prehospital triage in severely traumatized children: experience of a French regional trauma system

#### Jordan Porteaud^1^, Guillaume Mortamet^2^, Francois-Xavier Ageron^3^, Pierre Bouzat^1^

##### ^1^Département d’anesthésie, CHU de Grenoble, Grenoble, France; ^2^Service de réanimation pédiatrique, CHU de Grenoble, Grenoble, France; ^3^CH Annecy, Annecy, France

###### **Correspondence:** Jordan Porteaud (jporteaud@chu-grenoble.fr)

*Ann. Intensive Care* 2020, **10 (Suppl 1):**F-076

**Rationale:** Traumatic injuries are the leading cause of death and a major cause of disability in children. Prehospital triage is a key element in the management of trauma patients. This study aims (1) to describe the pre-hospital grading protocol developed by the Northern French Alps Emergency Network (TRENAU) for children, (2) to evaluate its quality to detect the most severe trauma patients and (3) to assess the accuracy of this procedure to perform an adequate triage.

**Patients and methods:** Our regional trauma system included 13 hospitals categorized as Level I, II or III pediatric trauma centers. Each patient was graded A, B or C by an emergency physician, according to the seriousness of their injuries at presentation on scene. The triage was performed according to this grading and the categorization of centers. This study is a registry analysis of an 8-year period (2009 to 2017).

**Results:** A total of 1142 children (mean age 10 years, 65% were boys) with severe trauma were included in the cohort. Fifty-seven, 22% and 21% of patients were admitted to a level I, II and III, respectively. Road accident was the main mechanism of injury (35% of patients). Thirty-six percent of patients had a severe trauma, defined as an Injury Severity Score (ISS) higher than 15. One quarter of patients had at least 2 severe lesions and one-third of patients had a trauma brain injury. The pre-hospital gradation was closely related with injury severity score (ISS) and intra-hospital mortality rate. The triage protocol had a sensitivity of 78% and a specificity of 33% to predict adequate admission of patients with ISS more than 15. Using a specific trauma score (including occurrence of death, an admission in Intensive Care Unit and the need for urgent surgery), sensitivity and specificity reached 91 and 41%, respectively. Fourty-six percent of patients were not graded at the scene (Non-graded group). Undertriage rate was significantly reduced in the Graded group compared with the Non-graded group, (22% versus 45%), without significant modification of the overtriage rate (43% versus 45%). Overall, mortality at discharge from hospital was 2%, but 33% in Grade A patients.

**Conclusion:** Implementation of a regional pediatric trauma system with a specific pre-hospital triage procedure was effective in detecting severe pediatric trauma patients and in lowering the rate of pre-hospital undertriage.

**Compliance with ethics regulations**: Yes.

### F-077 Glomerular filtration rate estimation formulas for assessing piperacillin clearance in critically ill children

#### Agathe Béranger^1^, Sihem Benaboud^2^, Saïk Urien^3^, Thao Nguyen Khoa^4^, Inès Gana^2^, Julie Toubiana^5^, Yi Zheng^2^, Fabrice Lesage^6^, Sylvain Renolleau^8^, Déborah Hirt^2^, Jean-Marc Treluyer^2^, Mehdi Oualha^1^

##### ^1^Réanimation pédiatrique, Necker Enfants Malades, Paris, France; ^2^Pharmacologie clinique, Cochin, Paris, France; ^3^URC, Cochin-Tarnier, Paris, France; ^4^Biochimie générale, Necker Enfants Malades, Paris, France; ^5^Pédiatrie générale, Necker Enfants Malades, Paris, France; ^6^Réanimation pédiatrique, Necker, Paris, France

###### **Correspondence:** Agathe Béranger (agathe.beranger@gmail.com)

*Ann. Intensive Care* 2020, **10 (Suppl 1):**F-077

**Rationale:** Critically ill children suffer from pathophysiological changes, leading to large between-subject variability in drug clearance. Since piperacillin is eliminated mainly via the kidney, changes in renal function go along with a modified elimination, and possible sub-therapeutic or toxic drug concentrations. We aimed to determine the most accurate glomerular filtration rate (GFR) estimation formula for assessing piperacillin clearance in critically-ill children.

**Patients and methods:** All children hospitalized in pediatric intensive care unit and receiving piperacillin were included. Piperacillin was quantified by high performance liquid chromatography. Pharmacokinetics were described using the non-linear mixed effect modeling software MONOLIX. In the initial pharmacokinetics model, GFR was estimated according to the Schwartz 1976 formula. In the study, GFR was estimated with 11 additional formulas, developed with plasma creatinine and/or cystatin C. Biases, precisions, Spearman’s rank correlation coefficient and normalized prediction distribution error (NPDE) were used to assess the models.

**Results:** We included 20 children with a median (range) postnatal age of 1.9 (0.1–19) years, body weight of 12.5 (3.5–69) kg and estimated GFR according to the Schwartz 1976 formula of 160.5 (38–315) mL min–1.1.73 m^2^. Piperacillin concentrations were best predicted with the model using the creatinine clearance. The correlations were most accurate: r2 = 0.7 between the population-predicted and the observed concentrations, r2 = 0.0004 and r2 = 0.04 for the NPDE versus population-predicted concentrations and time, respectively. Concerning the individual predicted concentrations, bias and precision were respectively − 4.1 mg L^−1^ and 14.7 mg L^−1^. GFR estimations based on serum creatinine were higher than those based on cystatin C (p = 0.02).

**Conclusion:** In summary, the 12-h creatinine clearance is the best predictor of piperacillin clearance and this could be investigated for drugs with renal elimination. As a whole, literature and our findings strongly suggest using creatinine clearance to also estimate GFR in critically ill children. The gap between the GFR estimations is large depending on the formulas, with higher estimations with equations based on serum creatinine.

**Compliance with ethics regulations**: Yes.

**Fig. 1:**
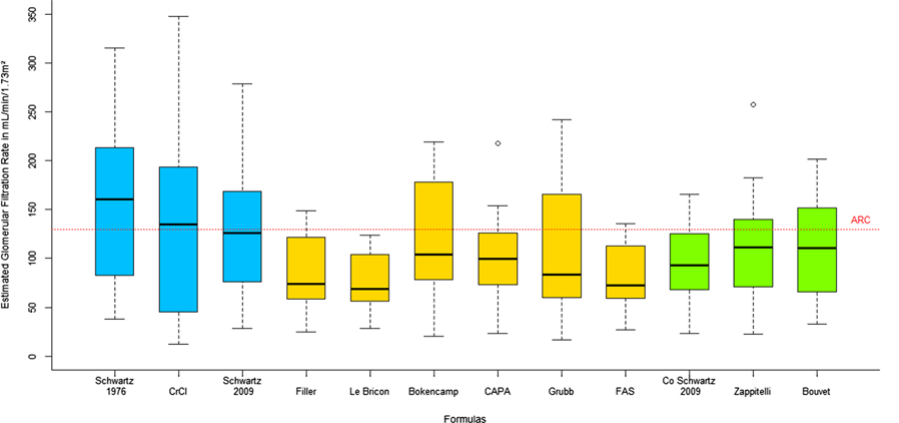
Boxplots of the glomerular filtration rate estimates in mL min^−1^ 1.73 m^−2^ according to 12 formulas. Boxplots of the glomerular filtration rate estimates in mL min^−1^ 1.73 m^−2^ according to 12 formulas. In blue, yellow and green, formulas depending respectively on creatinine, cystatin C and both. In dotted red line, the limit defining augmented renal clearance (ARC), at 130 mL min^−1^ 1.73 m^−2^

### F-078 Acute pancreatitis in the pediatric intensive care unit: French retrospective study

#### Elsa Amouyal^1^, Julie Sommet^1^, Jerome Rambaud^2^, Anna Deho^1^, Jérôme Naudin^1^, Michael Levy^1^, Géraldine Poncelet^1^, Fleur Le Bourgeois^1^, Guillaume Geslain^1^, Arielle Maroni^1^, Stéphane Dauger^1^, Maryline Chomton^1^

##### ^1^Robert Debré Hospital, Paris, France; ^2^Trousseau Hospital, Paris, France

###### **Correspondence:** Elsa Amouyal (elsa_amouyal@hotmail.com)

*Ann. Intensive Care* 2020, **10 (Suppl 1):**F-078

**Rationale:** Acute pancreatitis (AP) incidence have increased dramatically over the past years. New guidelines in 2018 were recently published in order to standardize the definition and management of AP. The aim of this study is to describe the management of children that were diagnosed with AP from the pediatric intensive care unit (PICU) in two French hospitals.

**Patients and methods:** This retrospective cohort study included children aged under 18 years old, who were admitted to the PICU of Robert-Debré hospital and Trousseau from 2006 to 2018 with a discharge diagnosis of AP. Data collected included management, severity and outcomes. We have also obtained data on clinical, biological and radiological presentation.

**Results:** Sixty patients were included, the median age was 8 years (5–14) and 75% had a co-morbidity mainly hematologic (26/60). Most of the AP were moderate (52%) or severe (45%). Hemodynamic failure was the main reason for PICU admission requiring a median fluid resuscitation 56 ml/kg complemented by a median intravenous fluid therapy of 4 ml/kg/h (2–5) during the first 24 h. Twenty patients (37%) required mechanical ventilation. Fasting has been instituted in 59 patients (98%) for a median of 4 days (1–6), whereas 54 patients (90%) received parenteral nutrition, only 18 patients (30%) received enteral nutrition. Antibiotic therapy was given to 53 patients (88%) including 13% for curative therapy. The median length of stay in PICU was 4 days (2–6). The mortality rate was 13%.

**Conclusion:** This is the first French study which precisely described the management of patients with AP in PICU. It highlighted the differences with the new international guidelines. This study could improve the management of PA in PICU and open research perspectives.

**Compliance with ethics regulations**: Yes.

### F-079 Therapeutic plasma exchange for immunologic disorders: a well-tolerated and safe process for pediatric life-threatening conditions

#### Jean-Sebastien Diana, Sandra Manceau, Tioka Rabeony, Caroline Elie, Anne-Marine Lenzotti, Valerie Jolaine, Melodie Aubart, Pierre Tremolieres, Nadege Salvi, Christine Bodemer, Brigitte Bader-Meunier, Christine Barnerias, Franck Iserin, Christophe Chardot, Florence Lacaille, Sylvain Renolleau, Remi Salomon, Francois Lefrere, Mehdi Oualha, Marianne Delville, Laurent Dupic

##### APHP, Paris, France

###### **Correspondence:** Jean-Sebastien Diana (jean-sebastien.diana@aphp.fr)

*Ann. Intensive Care* 2020, **10 (Suppl 1):**F-079

**Rationale:** Apheresis and Therapeutic plasma exchange (TPE) for children diseases has been poorly investigated in mostly small-uncontrolled studies. The purpose of this study is to describe indications and safety of TPE in children.

**Patients and methods:** In this single center and retrospective study, we included 94 patients who underwent TPE with an age < 18 years old in the pediatric center of Necker-Enfants-Malades hospital from January 2005 to December 2014. Data were retrospectively collected in an electronic case report form via a web-based data collection system.

**Results:** 78 patients with a median age of 9.8 years [range 0.53; 17.93] were selected. They achieved a total number of 731 procedures. Indications were antibody-mediated rejection (n = 33; 42%) or desensitization therapy (n = 5; 6%) for solid organ or hematopoietic transplantations; microangiopathy (n = 17; 22%); renal diseases (n = 6; 8%) and pediatric inflammatory diseases (n = 16; 21%); or hyperviscosity syndrome (n = 1; 1%). Each patient had an average of 6 procedures for the first session [range 1; 19] with a median volume of 1834 ml [range 500; 5000 ml] corresponding to a median (rang) total plasma volume (TPV) equivalent of 1.39 l/m2 [0.58–2.1]. Within 15 days since the beginning of sessions, 72 patients (92%) present a total of 311 Adverse Events (AEs) potentially related to TPE. There was a median (range) of 5 AEs/patients [0–24]. There was no association between AEs and diseases, severity of patients, venous access, plasma substitute and body weight. Few of AEs (n = 23 for 72 patients) were potentially life-threatening and concerned mostly critically ill children. Allergic reactions represented only 20 AEs for 14 patients (grade I n = 18; grade II n = 1; grade III n = 1). At the 12 months endpoint, 15 (19%) patients died and 10 (13%) patients had severe persistent disease. No death had been related to the TPE process.

**Conclusion:** We describe one of the largest retrospective pediatric cohort updated to the last international recommendations. TPE in children is performed for specific and potentially refractory disease. It is feasible without a major risk of life threatening adverse events.

**Compliance with ethics regulations:** Yes.

### F-080 Epidemiology and risk factors of infections on catheters with implantable chamber in the pediatric population

#### Yacine Benhocine

##### University hospital nedir mohamed, Tizi-Ouzou, ALGERIA

###### **Correspondence:** Yacine Benhocine (yacine001@yahoo.fr)

*Ann. Intensive Care* 2020, **10 (Suppl 1):**F-080

**Rationale:** Although analysis of literature data shows that implantable chamber catheters (ICCs) are less at risk of infectious complications than other central venous catheters, these complications can be serious, which may differ from ongoing treatments such as chemotherapy, and may lead to the removal of the implanted device. The literature on preventing these infections is quite disparate, as practices. Purpose: to evaluate the incidence of infections, to identify responsible germs and to measure the impact of preventive measures.

**Patients and methods:** Prospective, descriptive, mono-centric study, from January 2012 to January 2019. All patients under the age of 15 who have benefited from an implantable chamber catheter, whose insertion procedure is as follows: local anesthesia, surgical asepsis (polyvidone iodine) in an operating room, double disinfection, no antibiotic prophylaxis, routes used: subclavian (89%), internal jugular (11%) by anatomic registration. The main criteria of judgment are: the incidence of local and general infections, their time of onset, responsible microorganisms. Statistical analysis used the Statistical Package for the Social Sciences software.

**Results:** 4480 patients were included, the average incidence density of early infection is 0.61/1000 day-catheters. The time of onset of infection is essentially between the 2nd and 3rd week post-exposure, of which 20% is general infection. Ablation involved 52% of infected catheters. The causative organisms are mainly Gram-positive cocci (67.99%), Gram-negative bacilli are less involved (23.33%), with a significant number of candida infections (8%).

**Discussion:** Higher incidence of data from the literature. To remedy this requires the implementation of additional hygiene measures: antiseptic showers preoperatively, Chlorhexidine??, and practice changes: echo guidance, antibiotic prophylaxis or locks? Second generation catheters? Our practices are disparate especially since the recommendations specifically concerning the prevention of infectious risk associated with internationally published ICCs are rare.

**Conclusion:** At the end of this work, our perspectives are to: update the procedure, highlight risk factors on which it is possible to act, the adhesion of the different staff to the protocols.

**Compliance with ethics regulations**: Yes.

### F-081 Effect of a compliant bundled care on severity and mortality in children with severe sepsis hospitalized in PICU. Retrospective analysis of the DIABACT III study

#### Camille Beaucourt^1^, Fleur Lorton^2^, Elise Launay^2^, Florent Baudin^3^, Christèle Gras-Leguen^2^, Etienne Javouhey^4^

##### ^1^CHRU Jean Minjoz, Besancon, France; ^2^Hôpital Mère Enfant, Nantes, France; ^3^Hôpital Femme Mère Enfant, Bron, France; ^4^Hôpital Femme Mère Enfant, Lyon, France

###### **Correspondence:** Camille Beaucourt (camille.beaucourt91@gmail.com)

*Ann. Intensive Care* 2020, **10 (Suppl 1):**F-081

**Rationale:** The 2014 sepsis and septic shock pediatric guidelines advise to treat patients using care bundles. In the first hour, the «Resuscitation bundle» contains an appropriate fluid resuscitation, a broad-spectrum antibiotics administration after blood cultures, and initiation of inotrope if needed. The objectives were to evaluate the Resuscitation bundle compliance in a cohort of septic children with cardiovascular dysfunction, and to analyze the effect on severity and outcome in pediatric intensive care unit (PICU).

**Patients and methods:** Retrospective analysis of the DIABACT III study. This study analyzed the care course of children with severe community-acquired bacterial infection, hospitalized in PICUs in France’s west departments, between August 2009 and January 2014. Children with severe sepsis and cardiovascular dysfunction were retrospectively included.

**Results:** We included 92 children of whom 6 (6.5%) had compliant bundled care. The severity scores at PICU’s admission were similar between groups (p = 0.55 for the PRISM score and 0.58 for the PELOD 2). There was the same proportion of fluid-refractory shock (p = 0.65), mechanical ventilation (p = 1.0), neurological dysfunction (p = 1.0) and cardiac arrest (p = 0.39). In the «Resuscitation bundle compliant» group, 33.3% died versus 15.1% in the other group (p = 0.46). We highlighted a severity bias: the sickest patients were more likely to receive compliant bundled care.

**Conclusion:** In our cohort, the Resuscitation bundle’s compliance was low. We did not show some effect on morbidity nor mortality. However, this study helps understand the factors associated with Resuscitation bundle’s compliance.

**Compliance with ethics regulations**: Yes.

### F-082 Epidemiology of nosocomial infections with extended-spectrum β-lactamase producing gram-negative bacilli in a neonatal and paediatric Tunisian intensive care unit

#### Ahmed Ayari^1^, Asma Bouziri^1^, Ahmed Hajji^1^, Shatila Hadj Hassine^1^, Assaad Louati^1^, Khaled Menif^1^, Aïda Borgi^1^, Nejla Ben Jaballah^1^

##### ^1^Service de Réanimation Pédiatrique Polyvalente/Bechir Hamza Children’s Hospital of Tunis, Tunis, Tunisia

###### **Correspondence:** Ahmed Ayari (Ahmed.alayari@gmail.com)

*Ann. Intensive Care* 2020, **10 (Suppl 1):**F-082

**Rationale:** Nosocomial infections with extended-spectrum β-lactamase (ESBL) producing gram-negative bacilli (GNB) are an important cause of hospital morbidity and mortality. The objective of this study was to determine the incidence and risk factors of nosocomial ESBL-producing GNB infections in a paediatric intensive care unit (PICU).

**Patients and methods:** A prospective surveillance study was performed from January 2015 through March 2019 in a PICU. All patients hospitalized for more than 48 h were included. Centers for Disease Control and Prevention criteria were applied for the diagnosis of nosocomial infection.

**Results:** During the study period, 1783 patients (median age: 12 ± 434 days) were included. The average length of stay was 9 ± 11 days with a total of 15,980 days of hospitalization. Newborns accounted for 61.8% of patients. Sixty-two per cent of patients were colonized with multi drug resistant Gram-negative rods, on admission or during their stay in the PICU. One hundred and nineteen bacterial infectious episodes were registered (7.4/1000 patient days). One hundred infectious episodes were caused by a GNB and 44 (36.9%) by ESBLs producing GNB with an incidence of 2.7/1000 patient days (bloodstream infections: 21 episodes, ventilator acquired pneumonia: 16 episodes). ESBLs producing GNB infection had a specific incidence of 10.9 per 1000 catheter-days, and 2.3 per 1000 mechanical ventilation-days. Fifty-nine percent of patients infected with ESBLs producing GNB had a prior digestive colonization with a multidrug-resistant GNB. Forty-one episodes (93%) occurred in patients with central venous catheters. Klebsiella Pneumoniae was the most frequently isolated bacteria (45.4%). Mortality in the ESBLs producing GNB group was high (29.5%). Associated factors of nosocomial ESBLs producing GNB infection were mechanical vrntilation (p < 0.001), central venous catheterization (p < 0.001) and colonization with multiple drug-resistant Gram-negative bacteria (p < 0.001).

**Conclusion:** Nosocomial ESBL-producing GNB infection had an incidence of 2.7 per 1000 patient days in our unit and seems to increase the mortality rate. Factors associated with this infection were identified.

**Compliance with ethics regulations**: Yes.

### F-083 Serum phosphate variation precedes acute kidney injury in Tumor Lysis Syndrome

#### Marie Lemerle^1^, Aline Schmidt^2^, Valérie Thepot-Seegers^3^, Achille Kouatchet^1^, Valérie Moal^4^, Mélina Raimbault^5^, Corentin Orvain^2^, Jean-Francois Augusto^6^, Julien Demiselle^1^

##### ^1^CHU Angers, Médecine intensive réanimation, Angers, France; ^2^CHU Angers, Maladie du sang, Angers, France; ^3^CHU Angers-ICO, Angers, France; ^4^CHU Angers, Pharmacie, Angers, France; ^5^CHU Angers, LABORATOIRE de Biochimie, Angers, France; ^6^CHU Angers, néphrologie dialyse transplantation, Angers, France

###### **Correspondence:** Marie Lemerle (marielemerle@yahoo.fr)

*Ann. Intensive Care* 2020, **10 (Suppl 1):**F-083

**Rationale:** Acute kidney injury (AKI) is associated with high morbidity and mortality in the setting of tumor lysis syndrome (TLS). Thus, strategies aimed at preventing AKI occurrence represent a major goal to improve prognosis of patients with TLS. The role of hyperphosphatemia as a risk factor of TLS has been poorly analyzed. The aim of this study was to study the association between hyperphosphatemia and AKI, and to determine whether a cut-off value of phosphatemia or phosphatemia’s variation was associated with AKI development during TLS.

**Patients and methods:** In this retrospective and monocentric study, we included all patients with TLS and whithout AKI at admission, admitted to hematology, nephrology and intensive care units of the University Hospital of Angers between 01/01/2007 and 31/12/2017.

**Results:** One hundred and thirty TLS episodes were identified in 120 patients. AKI developed during 56 episodes of TLS (43%). Hospital mortality was much higher in AKI patients (26.8% versus 10.8%, p = 0.018). Phosphate maximal values (2.2 ± 0.7 versus 1.9 ± 0.3) and LDH maximal values (4337.5 ± 4511.9 versus 2437.7 ± 2937.0) were higher in TLS with AKI, before AKI occurrence (p = 0.006 and p = 0.009, respectively). We found no association between the other biological parameters of TLS and AKI (serum calcium, uric acid and potassium). After adjustment for cofounders, there was a strong association between a rise in phosphate level of 0.1 mmol/L (HR 1.31 IC 95% [1.19–1.42], p < 0.0001), exposure to platinum salts (HR 3.66 IC 95% [1.74–7.71], p = 0.0006) and increasing maximal LDH value (HR per 1000UI/L increase 1.10 IC 95% [1.03–1.17], p = 0.030) with AKI.

**Conclusion:** This study highlights the utmost importance of serum phosphate in the setting of TLS: phosphate is an early relevant biomarker for the risk of AKI development. Further studies are needed to assess whether aggressive prophylactic treatment to control serum phosphate concentration, such as renal replacement therapy before AKI onset, constitutes a valuable approach.

**Compliance with ethics regulations**: Yes.

### F-084 Chimeric Antigen Receptor T Cell-related toxicities requiring ICU admission: a single-centre experience

#### Clara Sortais^1^, Amélie Seguin^2^, Thomas Gastinne^1^, Amandine Le Bourgeois^1^, Steven Le Gouill^1^, Jean-Baptiste Lascarrou^3^, Charlotte Garret^3^, Maelle Martin^3^, Arnaud-Felix Miailhe^3^, Helene Migueres^3^, Laura Crosby^3^, Olivier Zambon^3^, Jean Reignier^3^, Emmanuel Canet^3^

##### ^1^Service d’Hématologie Clinique/CHU de Nantes, Nantes, France; ^2^Service de Médecine Intensive Et Réanimation/CHU de Nantes, Nantes, France; ^3^Service de Médecine Intensive Et Réanimation/CHU de Nantes, Nantes, France

###### **Correspondence:** Clara Sortais (clara.sortais@chu-nantes.fr)

*Ann. Intensive Care* 2020, **10 (Suppl 1):**F-084

**Rationale:** Chimeric Antigen Receptor T cells (CARTs) are genetically modified T lymphocytes with promising results in refractory or relapsed (R/R) lymphoid hematologic malignancies. Severe forms of CARTs-related toxicities may require ICU admission and management. Our aim was to describe the clinical features and outcome of patients admitted to the ICU after CARTs infusion.

**Patients and methods:** Retrospective cohort of patients admitted to the medical ICU of university affiliated hospital after CARTs treatment between August 2018 and August 2019.

**Results:** Of the 20 patients treated by CARTs in the haematology department, 7 (35%) were subsequently admitted to ICU. Median age was 66 [50.5–68.5] years, and 5 (71.4%) were female. CARTs were indicated for R/R lymphoma. The median time between CARTs injection and ICU admission was 5 [3.5–5.75] days. All patients had Cytokine Release Syndrome (CRS), and 6 (85.7%) developed CAR-related encephalopathy syndrome (CRES). Median SOFA score and SAPS 2 were 4 [2–4.5] and 45 [40.5–49.5], respectively. Four (57.1%) patients had hypotension treated by fluid bolus (n = 3) or vasopressors (n = 2), and 2 (28.6%) had acute respiratory failure requiring oxygen therapy (n = 1) or mechanical ventilation (n = 1). Six (85.7%) patients had neurological symptoms (impaired consciousness n = 4, confusion n = 2, transient aphasia n = 1), of whom one developed refractory convulsive status epilepticus afterwards. All patients received broad spectrum antibiotics, of whom 2 (28.6%) had documented infections. Six (85.7%) patients received interleukin-6 inhibitor (single dose n = 2, multiple doses n = 4), and 5 (71.4%) received intravenous dexamethasone. One patient died in the ICU from septic shock. Median ICU and hospital length of stays were 4 [4–12] and 24 [21.5–28.75] days, respectively. Two (28.6%) patients died from relapsing malignancy before hospital discharge. Three months after ICU admission, four (57.1%) patients were alive in complete remission.

**Conclusion:** More than 30% of patients treated with CARTs required ICU admission for the management of a CRS or a CRES. Early ICU admission, close collaboration between haematologists and intensivists, and prompt administration of appropriate therapy (IL-6 inhibitor and/or dexamethasone) and supportive care resulted in a good prognosis.

**Compliance with ethics regulations**: Yes.

### F-085 Pediatric Intensive Care Unit (PICU) management after Tisagenlecleucel (CTL019 Chimeric Antigen Receptor T Cell Therapy) infusion for B-Cell Acute Lymphoblastic Leukemia (ALL): experience of the first pediatric French patients

#### Jérôme Naudin^1^, Arthur Felix^2^, Maryline Chomton^1^, Michael Levy^3^, Marie-Emilie Dourthe^4^, Delphine Chaillou^4^, Karima Yakouben^4^, André Baruchel^5^, Stéphane Dauger^3^

##### ^1^Service de réanimation pédiatrique-CHU Robert Debré-APHP, Paris, France; ^2^Institut Gustave Roussy, Villejuif, France; ^3^Service de réanimation pédiatrique-CHU Robert Debré-APHP-Université de Paris, Paris, France; ^4^Service d’hématologie pédiatrique-CHU Robert Debré-APHP, Paris, France; ^5^Service d’hématologie pédiatrique-CHU Robert Debré-APHP-Université de Paris, Paris, France

###### **Correspondence:** Jérôme Naudin (jerome.naudin@aphp.fr)

*Ann. Intensive Care* 2020, **10 (Suppl 1):**F-085

**Rationale:** Tisagenlecleucel (CTL019) is a chimeric antigen receptor T cell therapy that reprograms autologous T cells to target CD19 + leukemia cells, approved in the US since August 2017 and in the EU since August 2018 for children and young adult (< 25 years old) with relapsed/refractory B-cell acute lymphoblastic leukemia (B-ALL). This study reports the experience of PICU management of CTL019 toxicity in patients treated in Robert-Debré University Hospitals.

**Patients and methods:** All patients (age < 18 years old) treated by Tisagenlecleucel infusions between March 1, 2016 and September 15, 2019, included in sponsored-clinical trials or treated within the French compassionate program or with the commercial product, were retrospectively analyzed.

**Results:** Twenty-four patients were infused and 8 patients (33%) were managed in PICU for 11 stays. (2 stays: n = 1 and 3 stays: n = 1). Median age at PICU admission was 11.5 years old [10.9; 13.2] with a median delay after CAR-T cells infusions of 5 days [4.5; 6]. The median length of stay in PICU was 2 days [1.5; 3] with a max at 25 days. Cytokine release syndrome (CRS) was the main indication of PICU hospitalization (37.5%, n = 9) with grade 2 (n = 5) and grade 3 (n = 4) according to American Society for Transplantation and Cellular Therapy (ASTCT) consensus grading system and treated by corticosteroid (n = .9) and Tocilizumab (n = 5, only one infusion). Norepinephrine was the only vasopressor used. The median Vaso-Inotrope Score (VIS) for grade 3 was 20 [17.5; 22.5] with a maximum at 30. Neurologic toxicity was observed in 2 patients with a grade 4 (status epilepticus) and grade 3 (focal edema on neuroimaging with depressed level of consciousness) according to Immune effector Cell-associated Neurotoxicity Syndrome (ICANS) grading system from ASTCT consensus. The status epilepticus was managed with anti-epileptic drugs without mechanical ventilation. The focal edema was related to HHV6 and toxoplasmosis encephalitis. Evolution was positive with foscavir and ganciclovir and 14 days of mechanical ventilation. One patient was hospitalized for septic shock secondary to gram-negative central line bloodstream infection in aplasia, with a VIS score at 20. Evolution was favorable with antibiotics and central line removal. No death in PICU from severe Tisagenlecleucel toxicity was observed since the beginning of the CAR-T cells program.

**Conclusion:** Toxicity profile of Tisagenlecleucel required frequent and early PICU hospitalization after infusions for severe CRS and ICANS management.

**Compliance with ethics regulations**: Yes.

### F-086 CAR-T cell therapy in ICU patients: a single-center experience

#### Sandrine Valade^1^, Eric Mariotte^1^, Virginie Lemiale^1^, Lara Zafrani^1^, Adrien Mirouse^1^, Yannick Hourmant^1^, Jean Jacques Tudesq^1^, Asma Mabrouki^1^, Igor Theodose^1^, Catherine Thieblemont^2^, Nicolas Boissel^2^, Michaël Darmon^1^, Elie Azoulay^1^

##### ^1^APHP, Saint Louis Hospital, Medical ICU, Paris, France; ^2^APHP, Saint Louis Hospital, Hematology, Paris, France

###### **Correspondence:** Sandrine Valade (sandrine.valade@aphp.fr)

*Ann. Intensive Care* 2020, **10 (Suppl 1):**F-086

**Rationale:** CAR-T cell (chimeric antigen receptor T) therapy is a promising treatment in refractory acute lymphoid leukemia (ALL) and diffuse large B cell lymphoma (DLBCL). The main complication consists in a cytokine release syndrome (CRS) leading to an inflammatory state that can be very severe with life-threatening organ failure. Neurological toxicity is also reported. We aim to describe CAR-T cells-related complications in ICU patients.

**Patients and methods:** this is a single-center prospective study conducted between July 2017 and August 2019. All the patients who have received CAR-T cells and who required ICU admission were included. CRS grading was defined according to the most recent classification of the ASBMT and neurological toxicity was assessed with the CARTOX scale. Each admission is considered independent and therefore corresponds to one patient.

**Results:** 48 admissions, representing 41 patients (27 men and 14 women), were considered. The median age was 56 years [27–65]. Two-thirds of the patients have been diagnosed with DLBCL (n = 34, 71%) and one-third with ALL (n = 14, 29%), 31 months [23–59] ago. They had received 4 lines [3–4] of chemotherapy and had a high tumor burden (65% of lymphomas classified stage IV). The majority of the patients was admitted because of hemodynamic failure (n = 23, 48%) or respiratory failure (n = 9, 19%), 5 days [3–7] after CAR-T cells infusion. SOFA at admission was 4 [2–6]. All the patients presented at least one complication (Figure), the most common being CRS (n = 39, 81%) with a median grade of 2 [1–2]. Neurological toxicity was reported in 17 (35%) patients (worst grade at 3 [2–4]). Documented bacterial infection involved 29% of the patients and consisted in catheter-related infections for half of the cases. In the ICU patients were managed with fluid resuscitation (n = 27, 56%) during the first day, vasopressors (n = 14, 29%) and broad spectrum antibiotics (98%). A single patient required mechanical ventilation and two patients underwent dialysis. Tocilizumab (anti-IL6 receptor) was given to 34 patients (87% of CRS) in a median time of 5.5 h [2.1–21.5] after ICU admission. 30 patients (61%) received corticosteroids. The median ICU length of stay was 4.5 days [3–6]. 3 patients (6%) died in the ICU and hospital mortality was 14%.

**Conclusion:** CAR-T cells-induced complications occur in more than 50% of the patients. They are mostly represented by CRS. Infections are common and should always be considered and empirically treated.

**Compliance with ethics regulations**: Yes.

### F-087 5-Fluorouracil-induced encephalopathy: a French national survey

#### Alice Boilève^1^, Laure Thomas^2^, Coralie Coccini^3^, Agnès Le-Lillo Louët^4^, Louise Gaboriau^5^, Laurent Chouchana^6^, Michel Ducreux^1^, David Malka^1^, Valérie Boige^1^, Antoine Hollebecque^1^, Dominique Hillaire-Buys^3^, Mathieu Jozwiak^7^

##### ^1^Département de Médecine Oncologique, Institut Gustave Roussy, Villejuif, France; ^2^Centre Régional de PharmacoVigilance, Assistance Publique des Hôpitaux de Paris, Hôpitaux Universitaires Paris-Est, Hôpital Henri Mondor, Créteil, France; ^3^Centre Régional de PharmacoVigilance, Hôpital Universitaire de Montpellier, Montpellier, France; ^4^Centre Régional de PharmacoVigilance, Hôpital Européen George Pompidou, Paris, France; ^5^Centre Régional de PharmacoVigilance, Assistance Publique des Hôpitaux de Paris, Hôpitaux Universitaires Paris-Centre, Hôpital Européen Georges Pompidou, Lille, France; ^6^Centre Régional de PharmacoVigilance, Assistance Publique des Hôpitaux de Paris, Hôpitaux Universitaires Paris-Centre, Hôpital Cochin, Paris, France; ^7^Service de Médecine Intensive Réanimation, Assistance Publique des Hôpitaux de Paris, Hôpitaux Universitaires Paris-Centre, Hôpital Cochin, Paris, France

###### **Correspondence:** Alice Boilève (alice.boileve@gmail.com)

*Ann. Intensive Care* 2020, **10 (Suppl 1):**F-087

**Rationale:** The 5-Fluorouracil (5-FU)-induced hyperammonemic encephalopathy is a rare but serious 5-FU adverse drug reaction, which could require the admission of patients in intensive care unit (ICU). Given the paucity of data regarding this 5-FU adverse drug reaction, we performed a retrospective national survey from the French pharmacovigilance database to better characterize 5-FU-induced hyperammonemic encephalopathy and its management.

**Patients and methods:** Since the inception of the French pharmacovigilance database, we identified all patients that experienced 5-FU-induced encephalopathy. Variables regarding epidemiology, characteristics, management and prognosis of these patients were collected and analyzed.

**Results:** From 1985 to 2018, 30 patients (60[55–66] years-old, 43% of women) were included. Overall mortality was 17% (n = 5) and 57% (n = 17) of patients were admitted in ICU. The 5-FU-induced hyperammonemic encephalopathy started 2[1–4] days after the onset of 5-FU infusion. The most common neurological disorders were consciousness impairment, confusion and seizures. Abnormalities in CT scan, MRI, electroencephalogram and lumbar puncture were found in 9%, 65%, 77% and 16% of the whole population respectively, similar in ICU and non-ICU patients. Ammonemia was dosed in 50% of the whole population and in 65% of ICU patients. Hyperammonemia tended to be higher in ICU than in non-ICU patients (250[133–522] vs. 139[68–220] µmol/L, respectively, p = NS) and in patients with the lowest Glasgow outcome scale, but was not different between survivors and non-survivors. Among ICU patients, 70% required mechanical ventilation and 47% anti-epileptic drugs administration. Besides 5-FU discontinuation, lactulose intake, renal replacement therapy or ammonium chelators were used to decrease hyperammonemia in 17%, 27% and 7% of patients respectively. A complete neurological recovery was observed in up to 70% of ICU and non-ICU patients within a delay of 5[2–10] days. A dihydropyrimidine deshydrogenase (DPD) deficiency was found in 21% of tested patients. A 5-FU rechallenge was considered in 47% (n = 14) of patients with complete neurological recovery, including a patient with a partial DPD deficiency, within a delay of 19[18–28] days after recovery. A 5-FU-induced hyperammonemic encephalopathy relapse was observed in 57% of patients with 5-FU rechallenge. No relapse was observed when 5-FU rechallenge was performed with a decreased 5-FU dosage.

**Conclusion:** We report the first national survey and the largest cohort of patients with 5-FU-induced hyperammonemic encephalopathy so far. This serious 5-FU adverse drug reaction must be known by intensivists, since more than half of patients are admitted in ICU and specific treatments are available.

**Compliance with ethics regulations**: Yes.

### F-088 Immune related adverse events: a retrospective look into the future of oncology in the intensive care unit

#### Adrien Joseph^1^, Annabelle Stoclin^2^, Antoine Vieillard-Baron^3^, Guillaume Geri^3^, Jean-Marie Michot^4^, Olivier Lambotte^5^, Elie Azoulay^6^, Virginie Lemiale^6^

##### ^1^U1138, INSERM, Equipe 11 labellisée Ligue Nationale Contre le Cancer, Centre de Recherche des Cordeliers, Paris, France; ^2^Département d’anesthésie-réanimation, Institut Gustave Roussy, Villejuif, France; ^3^Service de réanimation médico-chirurgicale, Hôpital Ambroise Paré, Assistance Publique Hôpitaux de Paris, Boulogne Billancourt, France; ^4^Département d’innovations thérapeutiques et d’essais précoces (DITEP), Institut Gustave Roussy, Villejuif, France; ^5^Service de médecine interne et immunologie clinique, Hôpital Bicêtre, Assistance Publique Hôpitaux de Paris, Le Kremlin-Bicêtre, France; ^6^Service de réanimation médical, Hôpital Saint-Louis, Assistance Publique Hôpitaux de Paris, Paris, France

###### **Correspondence:** Adrien Joseph (adrien.joseph@hotmail.fr)

*Ann. Intensive Care* 2020, **10 (Suppl 1):**F-088

**Rationale:** Immune checkpoint inhibitors (ICI) represent a paradigmatic shift in oncology. With their new position as a mainstay in cancer treatment, new toxicities called immune related adverse events (irAEs) have emerged.

**Patients and methods:** Retrospective study including patients admitted in the ICU within 60 days after treatment with an ICI in 3 French hospitals. Patients were classified into 3 groups according to the reason for admission: irAE, intercurrent adverse event (intAE) or event related to tumor progression (TumProg).

**Results:** 84 patients were admitted during the course of an ICI treatment, including 21 irAE, 25 intAE and 38 TumProg, with a significant increase between 2013 (n = 1) and 2018 (n = 24 patients, p for trend < 0.001). irAE included 5 pneumonitis, 4 colitis, 4 diabetes complications, 2 hypophysitis, nephritis, myocarditis and cardiac disorders, hepatitis or allergic reaction and 1 meningitis. The immune related nature of the complication was known before admission in only 4 (18%) cases. Mean age was 61 (± 14) years and 73% had a performance status of 0–1. Primary tumors were melanomas (14, 67%), non-small cell lung cancers (4, 19%), urothelial carcinomas (2, 10%) and Hodgkin lymphomas (1, 5%). ICI at the time of admission included anti-CTLA4 (5, 24%), anti-PD1/PDL1 (10, 48%) and anti-CTLA4/anti-PD1 combination in 6 (29%) patients. Mean duration of stay in the ICU was 6.5 (± 9) days. Three patients required vasopressor therapy alone, 2 with mechanical ventilation and one with extracorporeal membrane oxygenation. Three patients required non-invasive ventilation and 2 renal replacement therapy alone. Six required only endocrine or electrolytic equilibration and 4 others did not receive any form of organ support. ICU mortality was 14%. Compared with other admissions, anti-CTLA4 or anti-CTLA4/anti-PD1 combination treatments were associated with irAE diagnosis (OR = 6.1 [1.1–43.4], p = 0.021 for anti-CTLA4 and 5.7 [1.2–31.4] for anti-CTLA4/anti-PD1, p = 0.014) and so was the diagnosis of melanoma (5.7 [1.8–20.1], p = 0.001). There was no difference in terms of ICU and post-ICU survival between irAE (median post-ICU survival 14 months [7-NA]), intAE (20.9 [9.5-NA]) and TumProg (8.2 [7.3-NA]). Six patients admitted for an irAE were rechallenged with the same ICI after ICU discharge and 3 achieved complete response.

**Conclusion:** We conducted the first study describing patients admitted in the ICU for irAEs. Their specific and heterogeneous profile, along with the expected increase in the number of admissions, underlines the need for an in-depth knowledge for ICU physicians in order to take part in the multidisciplinary care required by these patients.

**Compliance with ethics regulations**: Yes.

**Fig. 1:**
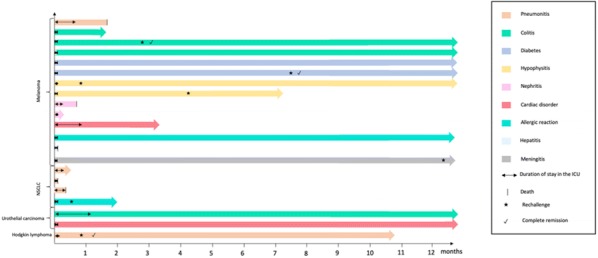
Swimmer plots of patients admitted in the intensive care unit for an immune related adverse event

### F-089 Tyrosin kinase inhibitor: an effective tool against lung cancer involvement responsible for acute respiratory failure in ICU

#### Yanis Akrour^1^, Yacine Tandjaoui-Lambiotte^1^, Boris Duchemann^2^, Frederic Gonzalez^3^, Annabelle Stoclin^4^, Fadwa El Kouari^5^, Aude Gibelin^6^, Paul Jaubert^7^, Anne-Sophie Moreau^8^, Florent Wallet^9^, Etienne De Montmollin^10^, Damien Contou^11^, Nicolas Dufour^12^, Romain Persichini^13^, Marc Pineton De Chambrun^5^, Alexandre Lautrette^14^, Anne Oppenheimer^15^, Yves Cohen^16^, Stéphane Gaudry^16^

##### ^1^Réanimation médico-chirurgicale, CHU Avicenne, APHP, Bobigny, France; ^2^Oncologie, CHU Avicenne, APHP, Bobigny, France; ^3^Réanimation, Institut Paoli Calmette, Marseille, France; ^4^Réanimation, Institut Gustave Roussy, Villejuif, France; ^5^Réanimation, CHU Pitié-Salpêtrière, APHP, Paris, France; ^6^Réanimation, CHU Tenon, APHP, Paris, France; ^7^Réanimation, CHU Cochin, APHP, Paris, France; ^8^Réanimation, CHRU, Lille, France; ^9^Réanimation, CHU Lyon, Lyon, France; ^10^Réanimation, CHU Bichat, APHP, Paris, France; ^11^Réanimation, Argenteuil, France; ^12^Réanimation, CH Réné Dubos, Pontoise, France; ^13^Réanimation, CHU de la Réunion, Saint Denis De La Réunion, France; ^14^Réanimation, CHU, Clermont-Ferrand, France; ^15^EA 7285 Research Unit ‘Risk and Safety in Clinical Medicine for Women and Perinatal Health’, Versailles-Saint-Quentin University, Clamart, France; ^16^Réanimation, CHU Avicenne, APHP, Bobigny, France

###### **Correspondence:** Yanis Akrour (akrour.yanis92@gmail.com)

*Ann. Intensive Care* 2020, **10 (Suppl 1):**F-089

**Rationale:** Patients with advanced-stage non-small-cell lung cancer have high mortality rates in the intensive care unit (ICU). In this context, acute respiratory failure due to cancer involvement is the worst situation. In the last two decades, targeted therapies have changed the prognostic of patients with lung cancer outside the ICU. Unlike cytotoxic chemotherapy, the fast efficacy of targeted therapies led some intensivists to use them as rescue therapy for ICU patients. We sought to investigate the outcomes of patients with lung cancer involvement responsible for acute respiratory failure and who received Tyrosine Kinase inhibitor during ICU stay.

**Patients and methods:** We performed a national multicentric retrospective study with the participation of the GRRROH (Groupe de Recherche en Réanimation Respiratoire en Onco-Hématologie). All patients with non-small-cell lung cancer admitted to the ICU for acute respiratory failure between 2009 and 2019 were included in the study if a Tyrosine Kinase Inhibitor was initiated during ICU stay. Cases were identified using hospital-pharmacies records. We collected demographic and clinical data in ICU charts. Vital status was assessed at the time of study completion (August 2019). The primary outcome was overall survival 90 days after ICU admission.

**Results:** Twenty-nine patients (age: 60 ± 14 years old) admitted to a total of 14 ICUs throughout France were included. Seventeen patients (59%) were nonsmoker. The most frequent histological type was adenocarcinoma (n = 20, 69%) and a majority had metastatic cancer (n = 21, 72%). Epithelial Growth Factor Receptor mutation was the most common oncologic driver identified (n = 15, 52%). During the ICU stay, 16 (55%) patients required invasive mechanical ventilation, 12 (41%) catecholamine infusion, 3 (10%) renal replacement therapy and one (3%) extracorporeal membrane oxygenation. In addition to Tyrosine Kinase Inhibitor, 20 (69%) patients received steroids (beyond 0.5 mg/Kg/day) and 3 (10%) cytotoxic chemotherapy during ICU stay. Seventeen patients (59%) were discharged alive from ICU and 9 (31%) were still alive after 90 days (see Kaplan–Meier curve Figure). Moreover, 5 patients (17%) were alive one year after ICU discharge.

**Conclusion:** Despite a small sample size this study showed that, in the context of lung cancer involvement responsible for acute respiratory failure, the use of Tyrosine Kinase Inhibitor should not be refrained in patients with severe condition in ICU.

**Compliance with ethics regulations**: Yes.

**Fig. 1:**
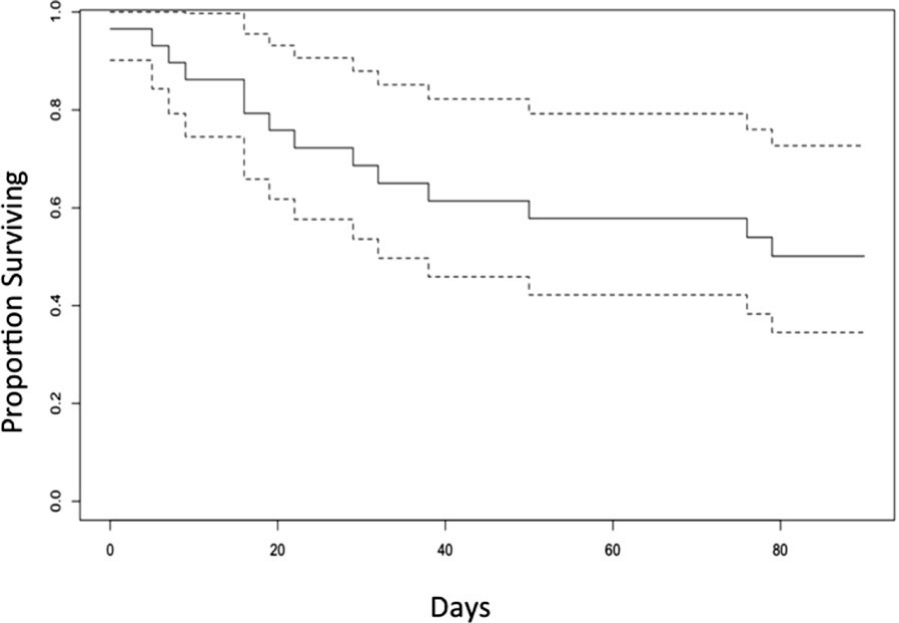
probability of survival after ICU admission

### F-090 Center effect in intubation risk in critically-ill immunocompromised patients with acute hypoxemic respiratory failure

#### Guillaume Dumas

##### ^1^Service de Médecine intensive-réanimation, Hôpital Saint-Louis, Paris APHP, Inserm UMR 1153, Paris, France

###### **Correspondence:** Guillaume Dumas (dumas.guillaume1@gmail.com)

*Ann. Intensive Care* 2020, **10 (Suppl 1):**F-090

**Rationale:** Acute respiratory failure is the leading reason for intensive care unit (ICU) admission in immunocompromised patients and the need for invasive mechanical ventilation has become a major clinical end-point in randomized controlled trials (RCT). However, data are lacking on whether intubation is an objective criteria that is used unbiasedly across centers. This study explores how this outcome varies across ICUs.

**Patients and methods:** Hierarchical models and permutation procedures for testing multiple random effects were applied on both data from observational cohort (the TRIAL-OH study: 703 patients, 17 ICUs) and randomized controlled trial (the HIGH trial: 776 patients, 31 ICUs) to characterize ICU variation in intubation risk across centers.

**Results:** The crude intubation rate varied across ICUs from 29% to 80% in the observational cohort and from 0 to 86% in the RCT. This center effect on the mean ICU intubation rate was statistically significant, even after adjustment on individual patient characteristics (observational cohort: p-value = 0.013, Median OR 1.48 [1.30–1.72]; RCT: p-value:0.004, Median OR 1.51 [1.36–1.68]). Two ICU-level characteristics were associated with intubation risk (the annual rate of intubation procedure per center and the time from respiratory symptoms to ICU admission) and could partly explain this center effect. In the RCT that controlled for the use of high-flow oxygen therapy, we did not find significant variation in the effect of oxygenation strategy on intubation risk across centers, despite a significant variation in the need for invasive mechanical ventilation.

**Conclusion:** Invasive mechanical ventilation has become an important endpoint in immunocompromised patients with acute respiratory failure. However, we found significant variation in intubation risk across ICU in both an observational cohort and a randomized controlled trial. Our results highlight the need to take into account center effect in analysis because it could be an important confounder. Reasons for heterogeneity are various (case-mix differences, center practices). This gives opportunities to future improvement in care management and study design.

**Compliance with ethics regulations**: Yes.

### F-091 The impact of Influenza on 28-day survival in ARDS non-trauma patients

#### Mathieu Lesouhaitier, Jean-Marc Tadié, Aurélien Frérou, Sonia Rafi, Claire Lhommet, Adel Maamar, Yves Le Tulzo, Arnaud Gacouin

##### CHU Rennes, Rennes, France

###### **Correspondence:** Mathieu Lesouhaitier (mathieu.lesouhaitier@chu-rennes.fr)

*Ann. Intensive Care* 2020, **10 (Suppl 1):**F-091

**Rationale:** Influenza virus (IV) infection is a major cause of ARDS that has been the focus of attention since the pandemic 2009 H1N1 (H1N1pdm2009) IV. Although IV-mediated damage of the airway has been extensively studied emphasizing specificity compared to other causes of ARDS, the impact of IV infection on the prognosis of ARDS patients, compared to the other causes of ARDS, has been few assessed.

**Patients and methods:** Systematic detection of IV in times of epidemic using RT-PCR in respiratory specimen is routine practice in our ICU along with prospective data collection of patients admitted to our ICU for ARDS with PaO_2_/FiO_2_ ratio ≤ 150 mmHg. All patients received lung-protective ventilation, the Sequential Organ Failure Assessment (SOFA) score was calculated on the first 3 days of mechanical ventilation. The primary endpoint compared the 28-day survival from the diagnosis of ARDS between patients with and without IV infection.

**Results:** From October, 2009 to May, 2019, 509 patients (pts) [median SAPS II score = 59 (33–67); age 58 years (47–68); PaO_2_/FiO_2_ ≤ 100 mmHg, n = 308 (61%)] were admitted to our ICU for ARDS with PaO_2_/FiO_2_ ratio ≤ 150 mm/Hg, including 100 pts (20%) with IV infection (H1N1pdm2009 IV A, n = 45; H3N2 A virus, n = 45; B virus, n = 10; associated bacteria, n = 46). Other main causes of ARDS were bacterial pneumonia without IV (39%), aspiration (15%), non-pulmonary sepsis (18%). 221 (42%) received prone positioning, and 47 (9%) extra-corporeal membrane oxygenation. The overall mortality rate at day-28 for the entire population was 33% (22 pts (22%) with IV infection versus 148 pts (36%) without IV infection, p = 0.007). Kaplan-Meier survival curves showed that survival was significantly higher in patients with IV infection than in those without IV infection. IV infection remained independently associated with a better prognosis at day-28 when entered as dichotomous variable (IV infection, yes/no) (adjusted hazard ratio (HR) = 0.60, 95% CI 0.37–0.98, p = 0.04) and when IV infection only was distinguished from other causes of ARDS including mixed infection IV plus bacteria (adjusted HR = 0.37, 95% CI 0.19–0.72, p = 0.003). Of note, within the first 3 days of mechanical ventilation, non-pulmonary SOFA scores were significantly lower in IV patients although similar pulmonary SOFA scores.

**Conclusion:** Our results suggest that patients with IV related ARDS have less severe non-pulmonary organ dysfunctions than those with ARDS from other and a lower mortality at day-28 despite similar ARDS severity.

**Compliance with ethics regulations**: Yes.

### F-092 Acute Respiratory Distress Syndrome: Acute Respiratory Distress Syndrome among French Army. Retrospective analysis about war casualties on fifteen years study

#### Johan Schmitt^1^, Mathieu Boutonnet^2^, Goutorbe Philippe^1^, Raynaud Laurent^3^, Cyril Carfantan^4^, Antoine Luft^5^, Pierre Pasquier^2^, Eric Meaudre^1^, Julien Bordes^1^

##### ^1^Military Teaching Hospital Saint Anne, Toulon, France; ^2^Military Teaching Hospital Percy, Paris, France; ^3^Tropical Medecine Institute’s French Health Army Service, Marseille, France; ^4^Military Medical Service, Ventiseri, France; ^5^French Army Health Service Direction, Paris, France

###### **Correspondence:** Johan Schmitt (schmitt.johan83@gmail.com)

*Ann. Intensive Care* 2020, **10 (Suppl 1):**F-092

**Rationale:** Acute Respiratory Distress Syndrome (ARDS) remains frequent in intensive care unit (ICU) with 20% to 40% mortality. According to Joint Theater Trauma System, ARDS occurs among 30% of war casualties: direct lung trauma, blast lesions, burn, massive transfusion and systemic inflammatory response syndrome lead to ARDS development. However, there is no data reporting ARDS among French evacuated casualties from forward environment. Our study’s aim is to describe ARDS incidence and its severity concerning medical evacuations from War Theater.

**Patients and methods:** This is an observational retrospective multicentric study analyzing all evacuated patient from War Theater and admitted in ICU. All patients developing ARDS according to Berlin definition have been included. Study has been approved by local ethic committee. Primary study endpoint was ARDS developing. Second study endpoints were ARDS severity, duration of invasive ventilation, ARDS treatments, ICU length of stay and mortality.

**Results:** 141 patients have been admitted in ICU between 2003 and 2018. 5 have been excluded. A total of 136 patients have been analyzed. 84% (n = 48) were military aged 30 (25–36) years. 42% (n = 57) developed ARDS. We found 57% (n = 32) war casualties, 30% (n = 17) trauma not related to war and 14% (n = 8) medical patients. Among severe trauma, median ISS was 34 (27–44), AIS thorax 3 (2–4), and 71% benefited from surgery on forward environment and 37% (n = 18) received massive transfusion. 22% (n = 13) suffered from mild ARDS, 42% (n = 24) moderate ARDS and 36% (n = 20) severe ARDS. Evacuation time was 26 (24–48) h. At admission in ICU, PaO_2_/FiO_2_ ratio was 241 (144–296) (Fig. 1). All patients were intubated. ARDS treatments used were curarization (76%, n = 43), prone position (16%, n = 9), inhaled nitric oxide (NOi) (10%, n = 6), almitrine (7%, n = 7) and extracorporeal life support (ECLS) (4%, n = 2). Invasive ventilation duration was 13 (7–27) days, length of stay 18 (9–33) days, and 3-month mortality 21% (n = 12).

**Conclusion:** According to our study, ARDS among French evacuated patients from war theaters remains frequent: it occurs on 42% among ICU admitted patients. 36% suffer from severe ARDS with 21% global mortality. Those datas are consistent with US studies. Also, we wonder if we must adapt our treatment capacities on forward environment for the most severe patients. In US army, a specialized team (Acute Lung Rescue Team) is trained to care the most hypoxemic war casualties with more treatment options as NOi, ECLS.

**Compliance with ethics regulations**: Yes.

**Fig. 1:**
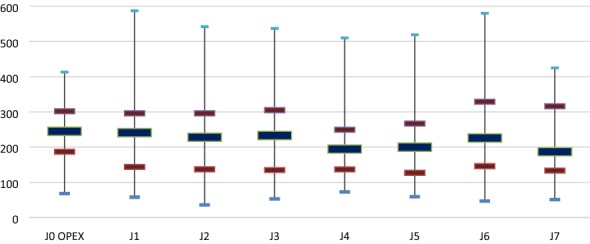
7-day evolution in PaO_2_/FiO_2_ ratio

### F-093 Title: Acute respiratory failure due to *Pneumocystis* with or without acute respiratory distress syndrome requiring ICU admission

#### Laure Folliet, Jean-Christophe Richard, Damien Dupont, Meja Rabodonirina, Laurent Argaud, Vincent Piriou, Thomas Rimmele, Claude Guérin

##### HCL, Lyon, France

###### **Correspondence:** Laure Folliet (laure.folliet@chu-lyon.fr)

*Ann. Intensive Care* 2020, **10 (Suppl 1):**F-093

**Rationale:** *Pneumocystis jirovecii* pneumonia (PCP) is an opportunistic severe respiratory infection occurring in immunocompromised individuals. The main objective of present study was to evaluate the mortality of PCP patients with acute respiratory failure hospitalized in ICU, according to the presence of acute respiratory distress syndrome (ARDS) within the first 24 h of ICU stay.

**Patients and methods:** We performed a multicenter retrospective study in 4 adult ICUs in Lyon, France. Patients were included if they were admitted to ICU for acute respiratory failure with a respiratory sample tested positive for *Pneumocystis jirovecii*. ARDS was defined according to the Berlin definition. The primary endpoint was the mortality at ICU discharge. Patients were compared according to survival status at ICU discharge and ARDS within the first 24 h of ICU stay for demographic and anthropometric variables, underlying chronic medical condition, severity of the acute illness, PCP characteristics, ventilatory management and adjunct therapies during the ICU stay. Univariate comparisons were made by using non parametric tests. A multivariate logistic regression analysis was performed by using as covariates the variables which differed between survivors and not survivors in the univariate analysis, the ICU and the ARDS. No propensity score was used.

**Results:** From January 1st 2014 to September 30th 2018, 245 patients were included. The mortality at ICU discharge was 45.7% (112/245). In univariate analysis, non-survivor subjects were older, had higher SOFA score and SAPS2 score (69 vs. 66; p = 0.012), had more solid tumor (50.9% vs. 36.1%; p = 0.020), interstitial lung disease (21 vs. 10; p = 0.008) and less HIV (1.8% vs 10.5%;p = 0.006). Ninety-one patients (37.1%) met ARDS criteria. ARDS was more frequent in the non-survivor group in comparison to the survivor group (49.1% vs 27.1% p < 0.001). The results of the multivariate analysis indicated that the factors associated with mortality in PCP patients with respiratory failure were: chronic underlying interstitial lung disease, solid tumor. The factors associated with mortality reduction were oxygen therapy alone, high flow oxygen therapy alone, and adjunction of steroids for PCP.

**Discussion:** PCP is a severe respiratory infection that can complicate the evolution of many diseases accompanied by immunosuppression. We found that some risk factors are modifiable, and hence would allow an adapted and personalized care in patients with PCP admitted to ICU for acute respiratory failure.

**Conclusion:** This study identifies risk factors of mortality in patients with PCP and admission to ICU for acute respiratory failure.

**Compliance with ethics regulations**: Yes.

### F-094 Impact of bronchoalveolar lavage in mechanically ventilated patients: an electrical impedance tomography—based study

#### Guillaume Franchineau, Paul Masi, Nicolas Brechot, Guillaume Hekimian, Loïc Le Guennec, Simon Bourcier, Charles-Edouard Luyt, Alain Combes, Matthieu Schmidt

##### Médecine Intensive-Réanimation, CHU Pitié-Salpêtrière, Paris, France

###### **Correspondence:** Guillaume Franchineau (gfranchineau@gmail.com)

*Ann. Intensive Care* 2020, **10 (Suppl 1):**F-094

**Rationale:** Impact of bronchoalveolar lavage (BAL) on regional ventilation during mechanical ventilation patients has not been thoroughly studied. Electrical impedance tomography (EIT) allows monitoring tidal and end-expiratory lung volumes, and their respective regional distribution on mechanical ventilation. Objective: To describe, using EIT, the effects of BAL on tidal volume distribution and end-expiratory lung volume in mechanically ventilated patients.

**Patients and methods:** Monocentric prospective observational study including mechanically ventilated patients requiring a BAL between December 2018 and May 2019. Tidal volume distribution (i.e. tidal impedance (Δz)), variation of end-expiratory lung volume (i.e. End-Expiratory Lung Impedance (EELI)), and global static compliance were recorded before, immediately after, and every hour until 6 h after BAL in two distinct sub-groups, namely PaO_2_/FiO_2_ < 200 or ≥ 200. Results are expressed as median (1st–3rd quartiles) and number (percentage).

**Results:** Twenty-one patients (62% male, 62% with a PaO_2_/FiO_2_ < 200) were included. Median BAL duration was 6:37 (5:28–8:48) min and was mostly performed in the right lung (18/21 patients). Median volume injected was 120 mL, resulting in 53 (40–67) mL collected. Global compliance decreased after BAL from 33.5 (22.8–42.2) to 29.3 (20.4–36.2) mL/cmH_2_O, before and 30 min after, respectively (P = 0.09). In patients with PaO_2_/FiO_2_ ≥ 200, Δz in the BAL-side during the following 6 h was significantly lower than before BAL. The lowest values were obtained at 30 min and 1 h (P = 0.01). Meanwhile, percentage of total EELI shifted from 55 (49–64) to 49 (46–56) % at 30 min in the BAL-side (P = 0.09) (Fig. 1). Same trends were observed in patients with PaO_2_/FiO_2_ < 200 though not reaching significance.

**Conclusion:** BAL in mechanically ventilated patients induces a significant decrease of 1) the global and regional static compliances, and 2) the end-expiratory lung volume in the BAL-side, which was still present 6 h after the procedure. These results reached significance only in patients with PaO_2_/FiO_2_ ≥ 200. The severe ventilation inhomogeneity before the BAL in patients with PaO_2_/FiO_2_ < 200 could have attenuated the BAL effect and may therefore explains these findings.

**Compliance with ethics regulations**: Yes.

**Fig. 1:**
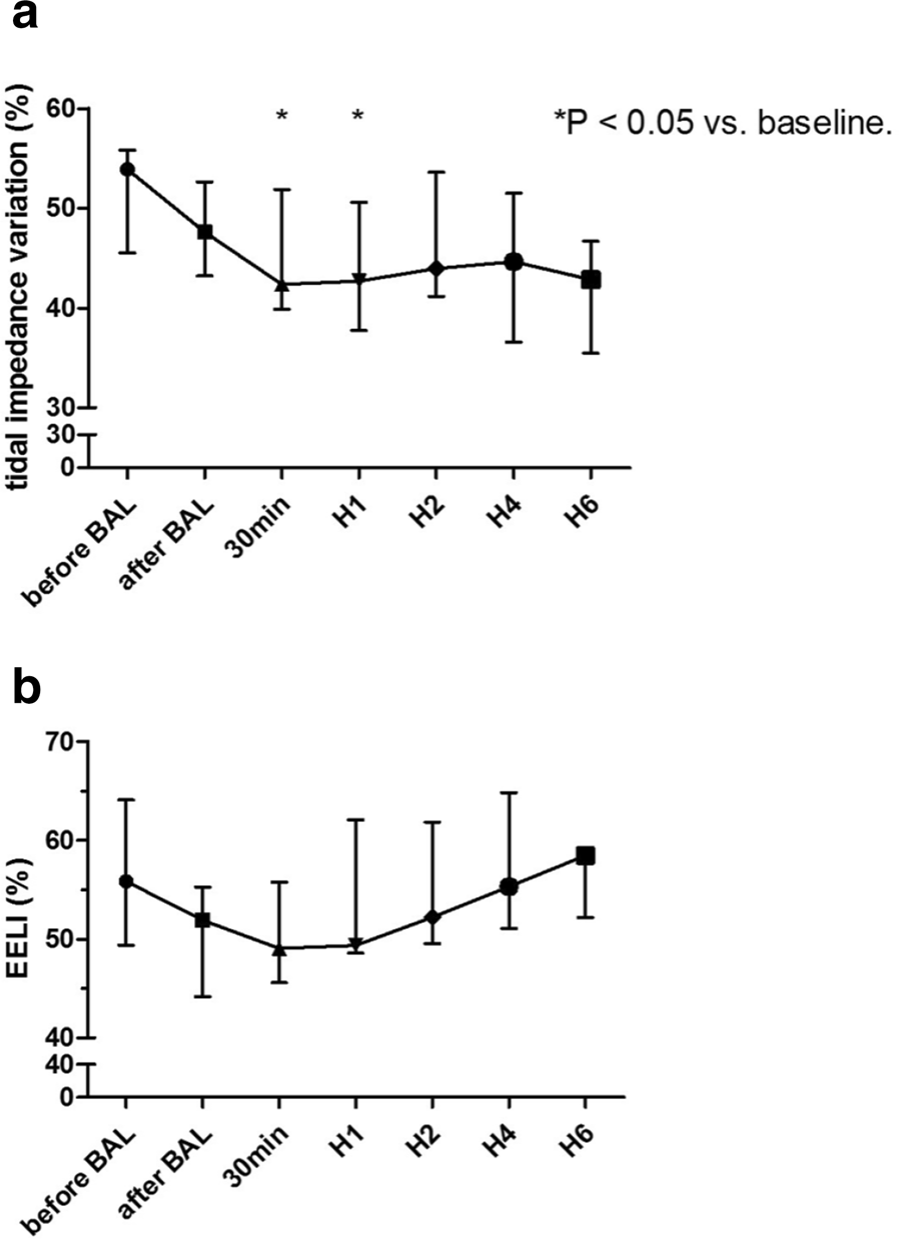
Distribution of A) median tidal impedance variation and B) median End-Expiratory Lung Impedance, in the side of broncho-alveolar lavage on patients without Acute Respiratory Distress Syndrome

### F-095 Effect of transesophageal echocardiography on tracheal microaspiration and ventilator-associated pneumonia in intubated critically-ill patients: a multicenter prospective observational study

#### François Bagate^1^, Anahita Rouze^2^, Farid Zerimech^3^, Florence Boissier^4^, Vincent Labbe^5^, Keyvan Razazi^1^, Guillaume Carteaux^1^, Saad Nseir^2^, Armand Mekontso Dessap^1^

##### ^1^CHU Henri Mondor, Service de Médecine Intensive Réanimation, Créteil, France; ^2^CHU Lille, Service de Médecine Intensive Réanimation, Lille, France; ^3^CHU Lille, Centre de Biologie Pathologie, Lille, France; ^4^CHU Poitiers, Service de Médecine Intensive Réanimation, Poitiers, France; ^5^CHU Tenon, Service de réanimation polyvalente, Paris, France

###### **Correspondence:** François Bagate (francois.bagate@aphp.fr)

*Ann. Intensive Care* 2020, **10 (Suppl 1):**F-095

**Rationale:** Microaspiration of gastric and oropharyngeal secretions is the main mechanism of ventilator-associated pneumonia (VAP) pathogenesis. Transesophageal echocardiography (TEE) is performed routinely in intensive care unit and could potentiate microaspiration. The study aimed at evaluating the impact of TEE on microaspiration and VAP in adult intubated critically ill patients.

**Patients and methods:** Four-center prospective observational study. Quantitative measurement of microaspiration biomarkers (pepsin and salivary amylase) concentrations were performed in tracheal aspirates before and after TEE. The primary endpoint was the proportion of patients with TEE-associated microaspiration, as defined by: i) ≥ 50% increase in biomarker concentration between pre TEE and post TEE samples, and ii) a significant post TEE biomarker concentration (> 200 µg/L for pepsin and > 1685 IU/L for salivary amylase). Secondary endpoints included the occurrence of VAP within 3 days after TEE and the evolution of tracheal cuff pressure during TEE.

**Results:** Among 100 patients enrolled, VAP occurred in 19 patients (19%) within the 3 days following TEE. Patients with VAP had a larger tracheal tube size, had more attempts of TEE probe introduction, and were more often under anticoagulation treatment, as compared to their counterparts. We identified 17 patients with TEE-associated microaspiration (23%) among the 74 patients analyzed for biomarkers, but overall, pepsin and salivary amylase levels were not significantly different before and after TEE, with a wide interindividual variability. TEE induced an increase in tracheal cuff pressure, especially during insertion and removal of the probe.

**Conclusion:** Our results suggest no significant impact of TEE on microaspiration markers of gastric contents and oropharyngeal secretions and on VAP in intubated critically ill patients.

**Compliance with ethics regulations**: Yes.

### F-096 Severe Chest Trauma as a Risk Factor of Post-Traumatic Pulmonary Embolism in the ICU

#### Mariem Dlela^1^, Yassmine Kammoun^2^, Olfa Turki^1^, Kamilia Chtara^1^, Mounir Bouaziz^1^

##### ^1^Habib Bourguiba hospital, Sfax, Tunisia; ^2^Hedi Chaker hospital, Sfax, Tunisia

###### **Correspondence:** Mariem Dlela (mariem241090@gmail.com)

*Ann. Intensive Care* 2020, **10 (Suppl 1):**F-096

**Rationale:** It is wellestablished nowadays that trauma patients bear a significantly increased risk of venous thrombo-embolic (VTE) events. Recent literature suggests that severe chest injury could constitute a risk factor for post-traumatic pulmonary embolism. The aim of this study was to determine predictive risk factors of pulmonary embolism (PE) in ICU trauma patients and evaluate whether severe chest trauma is independently associated to post-traumatic PE.

**Patients and methods:** We conducted a prospective cohort including trauma patients admitted to our ICU over a 20-month period between January 1^st^, 2017 and August 31^st^ 2018. Patients were screened for PE at least once during ICU stay. PE was diagnosed based on computed tomography pulmonary angiogram (CTPA) results. Severe chest trauma was defined based on an Abbreviated Injury Scale (AIS) over three. Both univariate and multivariate analysis were used to determine level of significance.

**Results:** A total of 365 trauma patients were admitted to our ICU during the study period and 66 patients (18.08%) were diagnosed with PE. Patients had a mean age of 40 ± 14 years and a mean Injury Severity Score (ISS) of 34 ± 10. Thirty-eight patients (10.4%) were diagnosed with severe blunt chest injury (AIS ≥ 3). Univariate analysis identified the following variables as predictive of post-traumatic PE, including older age, obesity (BMI > 30), severe head injury (AIS head ≥ 3), severe chest trauma (AIS chest ≥ 3), higher SOFA scoring, lower PaO_2_/FiO_2_ ratio on admission and post-traumatic disseminated intravascular coagulation. On multivariate analysis, we found that severe chest trauma was an independent factor predictive of post-traumatic PE (p = 0.008; OR = 8.7; CI = 7.76–43.4), in addition to severe head injury (p = 0.035; OR = 2.3; CI = 1.16–5.15) and obesity (p < 0.001; OR = 4.8; CI = 2.03–12.3).

**Conclusion:** This cohort study confirms that severe chest trauma should be recognized as a newly identified risk factor of post-traumatic PE. This knowledge should lead clinicians to maintain a high index of suspicion in injured patients.

**Compliance with ethics regulations**: Yes.

### F-097 Impact of pulmonary and non-pulmonary sepsis on susceptibility to secondary bacterial pneumonia: a pivotal role for the alveolar macrophage?

#### Jean-FrançOis Llitjos^1^, Zakaria Ait Hamou^1^, Christophe Rousseau^1^, Matthieu Benard^1^, Hugues Vicaire^1^, Clémence Martin^2^, Pierre-Régis Burgel^2^, Jean-Paul Mira^3^, Jean-Daniel Chiche^3^, Maha Zohra Ladjemi^1^, Frédéric Pene^3^

##### ^1^INSERM U1016, Institut Cochin, 22 rue Méchain, 75014, Paris, France; ^2^Service de pneumologie, hôpital Cochin, Paris and INSERM U1016, Institut Cochin, 22 rue Méchain, 75014, Paris, France; ^3^Médecine Intensive-Réanimation, hôpital Cochin, Paris and INSERM U1016, Institut Cochin, 22 rue Méchain, 75014, Paris, France

###### **Correspondence:** Jean-FrançOis Llitjos (jllitjos@gmail.com)

*Ann. Intensive Care* 2020, **10 (Suppl 1):**F-097

**Rationale:** We recently reported that septic shock patients with pneumonia exhibit a high risk of ICU-acquired pneumonia, suggesting that a primary pulmonary insult may drive profound alterations in lung defence towards secondary infections (1). Given their importance in lung immune surveillance, alveolar macrophages (AM) are likely to play a pivotal role in this setting. The objective of this experimental study is to address the impact of primary pulmonary or non-pulmonary infectious insults on lung immunity.

**Patients and methods:** We established relevant double-hit experimental models that mimic common clinical situations. C57BL/6 J mice were first subjected either to polymicrobial peritonitis induced by caecal ligation and puncture (CLP), or to bacterial pneumonia induced by intra-tracheal instillation of *Staphylococcus aureus* or *Escherichia coli*. Respective control mice were subjected to sham laparotomy or intra-tracheal instillation of phosphate-buffered saline. Seven days later, mice that survived the primary insult were subjected to intra-tracheal instillation of *Pseudomonas aeruginosa* (PAO1 strain). We assessed survival and pulmonary bacterial clearance of post-septic animals subjected to *P. aeruginosa* pneumonia, as well as the distribution and functional changes in alveolar macrophages.

**Results:** When compared to sham-operated mice, post-CLP animals exhibited increased susceptibility to secondary *P. aeruginosa* pneumonia as demonstrated by defective lung bacterial clearance and increased mortality rate (50% vs. 0%, p < 0.05). In contrast, all post-pneumonia mice survived and even exhibited improved bacterial clearance as compared to their control counterparts. When addressing whole-lung immune cell distribution at the time of second hit (day 7), amounts of AM were decreased in post-CLP mice while preserved or even increased in post-pneumonia mice. Antigen-presenting functions of AM appeared similar in all conditions. Percentages of apoptotic (AnnexinV^+^) and necrotic (7-AAD^+^) AM were comparable at day 1 and day 7 after the first hit. Interestingly, both Ly6C^high^ and Ly6C^low^ monocytes were sustainably increased in the lungs of post-CLP mice, while only transiently expanded following pneumonia, suggesting that differences in AM counts could be related to modulated turnover from precursor monocytes.

**Conclusion:** Using clinically relevant double-hit experimental models, a primary pulmonary infection conferred resistance to secondary bacterial pneumonia. Ongoing investigations are aimed at addressing the antibacterial AM functions, as well as the turnover-driving mechanisms.**Compliance with ethics regulations**: Yes.

**Fig. 1:**
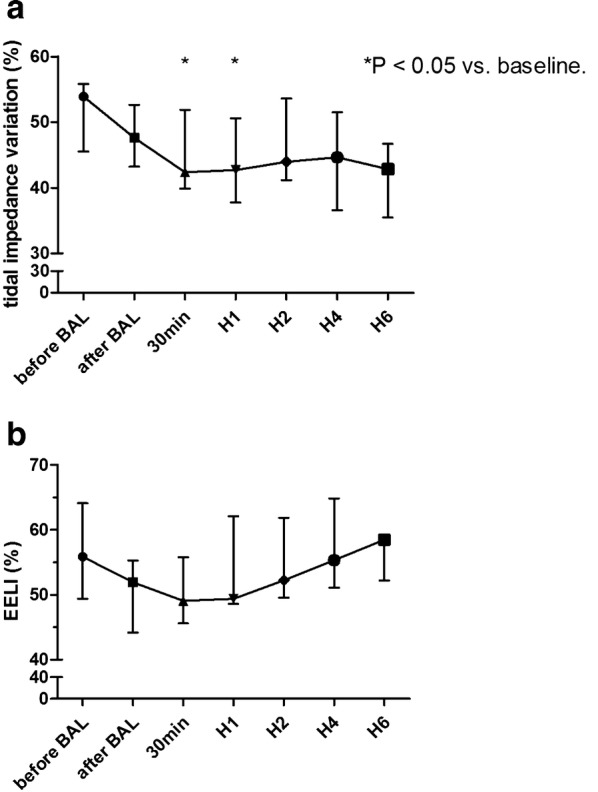
.

**Reference**
1. Llitjos JF et al., Ann Intensive Care 2019.

### F-098 Local signs at insertion site and prediction of catheter-related infections in short-term central venous and arterial catheters in the ICU. Individual findings from 4 multicenter randomized controlled trials

#### Niccolo Buetti^1^, Stephane Ruckly^1^, Jean-Christophe Lucet^1^, Olivier Mimoz^2^, Bertrand Souweine^3^, Jean-François Timsit^1^

##### ^1^INSERM IAME DESCID, Paris, France; ^2^Services des Urgences Adultes and SAMU 86, Centre Hospitalier Universitaire de Poitiers, Poitiers, France; ^3^Medical ICU, Gabriel-Montpied University Hospital, Clermont-Ferrand, France

###### **Correspondence:** Niccolo Buetti (niccolo.buetti@gmail.com)

*Ann. Intensive Care* 2020, **10 (Suppl 1):**F-098

**Rationale:** Little is known on the role of exit-site signs in predicting intravascular catheter infections. The current study aimed to describe the association between local signs at the exit-site and catheter-related bloodstream infection (CRBSI), which factors substantially influenced local signs and which clinical conditions may predict CRBSIs if inflammation at insertion site is present.

**Patients and methods:** We used individual data from 4 multicenter randomized-controlled trials in intensive care units (ICUs) that evaluated various prevention strategies regarding colonization and CRBSI in central venous and arterial catheters. We used univariate and multivariate logistic regression stratifying by center in order to identify variables associated with redness, pain, non-purulent discharge, purulent discharge and ≥ 1 local sign and subsequently evaluate the association between CRBSI and local signs. Moreover, we evaluated the role of the different local signs for developing CRBSI in subgroups of clinically relevant conditions.

**Results:** A total of 6976 patients, 14,590 catheters (101,182 catheter-days) and 114 CRBSI (0.8%) from 25 ICUs with described local signs were included. Redness, pain, non-purulent discharge, purulent discharge and ≥ 1 local signs at removal were observed in 1633 (11.2%), 59 (0.4%), 251 (1.7%), 102 (0.7%) and 1938 (13.3%) episodes, respectively. The sensitivity of ≥ 1 local sign for CRBSI was by 40.4%, whereas the highest specificities were observed for pain (99.6%) and purulent discharge (98.4%). Positive predictive value (PPV) was low for redness (2%), pain (3%), non-purulent discharge (3%) and ≥ 1 local sign (2%), but increased for purulent discharge (12.7%). Negative predictive values were high for all local signs. After adjusting on confounders, CRBSI was associated with redness, non-purulent discharge, purulent discharge and ≥ 1 local sign (Fig. 1). Conditions independently associated with ≥ 1 local sign were age ≤ 75 years old (OR 1.23, 95% CI 1.07–1.40, p < 0.01), SOFA score (SOFA < 8 OR 1.45, 95% CI 1.23–1.71, p < 0.01), non-immunosuppression (OR 1.38, 95% CI 1.12–1.68, p < 0.01), catheter maintenance > 7 days (OR 3.36, 95% CI 3.03–3.73, p < 0.01) and insertion site (OR for subclavian site 1.63, 95% CI 1.37–1.93, p < 0.01). However, the presence of ≥ 1 local sign was more predictive for CRBSI in the first 7 days of catheter maintenance (OR 6.30, 95% CI 3.53–11.24 vs. > 7 catheter-days OR 2.61, 95% CI 1.58–4.32, p heterogeneity = 0.02).

**Conclusion:** This post hoc analysis showed that local signs were related to CRBSIs in the ICU. Local signs were independently associated with specific patient’s and catheter’s conditions. In the first 7 days of catheter maintenance, local signs were predictive for CRBSI.

**Compliance with ethics regulations**: Yes.

**Fig. 1:**
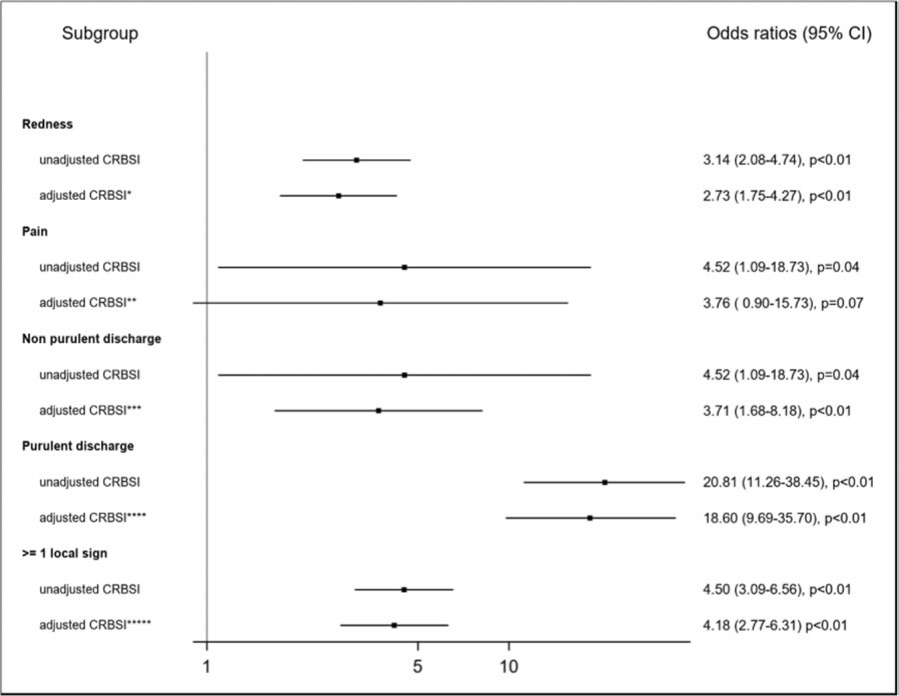
Unadjusted and adjusted CRBSI-risk for the different local signs

### F-099 Epidemiology and Outcomes of Pneumococcal meningitis with Sepsis in France

#### Claire Dupuis^1^, Michael Thy^1^, Bruno Mourvillier^1^, Lila Bouadma^1^, Stephane Ruckly^2^, Anne Perozziello^3^, Damien Van-Gysel^4^, Etienne de Montmollin^1^, Romain Sonneville^1^, Jean-François Timsit^1^

##### ^1^Réanimation médicale et infectieuse, CHU Bichat, AP-HP, Paris, France; ^2^UMR 1137 IAME Team Descide, Paris, France; ^3^UMR 1137-IAME Team 5–DeSCID, Paris, France; ^4^Département d’Informations Médicales, CHU Bichat, APHP Paris, Paris, France

###### **Correspondence:** Claire Dupuis (cdup83@gmail.com)

*Ann. Intensive Care* 2020, **10 (Suppl 1):**F-099

**Rationale:** Pneumococcal meningitis (PM) is the leading cause of bacterial meningitis in adult patients requiring ICU admission and is associated with a high case fatality rate (CFR), ranging from 15 to more than 33% (1–3). Patients with PM may develop sepsis or septic shock that may impact management and outcomes. We aim to describe the epidemiology and outcomes of PM associated with sepsis in adult patients in France.

**Patients and methods:** We analysed the occurrence of PM with sepsis from 2010 to 2015 in adult patients, using the national French hospital Database PMSI (Programme de Médicalisation des Systèmes d’Information). For all analyses, only the first hospital admission was considered. Cases were identified using a combination of a diagnosis code for PM plus a diagnosis code for sepsis (either a code for organ failure or a procedure code for organ support). Data recorded included comorbidities (4), characteristics of the hospital stay, severity of the patients including major intracranial complications and characteristics of the infection. Costs and endpoints were determined at the end of all the hospital stays related to the first admission for PM with sepsis. Standardized incidence, hospital mortality, and CFR were estimated. Temporal trends were assessed using Cochran Armitage tests of trends and linear trend analyses.

**Results:** A total of 1236 PM with sepsis aged ≥ 18 years were hospitalized in France during 2010–2015. The incidence of PM decreased from 3.7 to 2.6 per 1 M inhabitants (p < 0.02) (Fig. 1). Most of them came from home (94%), were admitted in an academic institution (93%) and benefited from ICU (93%). Their median age was 62 [51; 73] years. Two-third of them had at least one comorbidity. The initial neurological presentations included coma (58%), focal signs (15%), seizures (12%) and brain stem involvements (16%). The SAPS II score was 57 [44; 69] points. The main neurological complications were cerebrovascular complications (6%), cerebral abscess (4%) and hydrocephaly (2%). PM was associated with pneumococcal septicaemia or pneumococcal pneumonia in 30% and 23% of cases respectively. The length of ICU and hospital stays were 10 [4; 21] and 22 [10; 41] days respectively and only ICU length of stay decreased over time (p < 0.01). The prognosis was poor since only 27.6% of the patients were discharged to home. Indeed, 42.6% of them died and 18% were transferred to rehabilitation units. No temporal trends could be observed for these outcomes. The average hospital costs per case were 21,717€ [13.198; 34.232].

**Conclusion:** PM with sepsis in adult in France remained a real burden associated with a high mortality rate, and disability.

**References**
1. Auburtin, Crit Care Med. 2006.
2. Muralidharan, Eur J Neurol. 2014.
3. Tsai, Clin Infect Dis. 2008.
4. Quan H, Med Care. 2005.

**Compliance with ethics regulations**: NA.

**Fig. 1:**
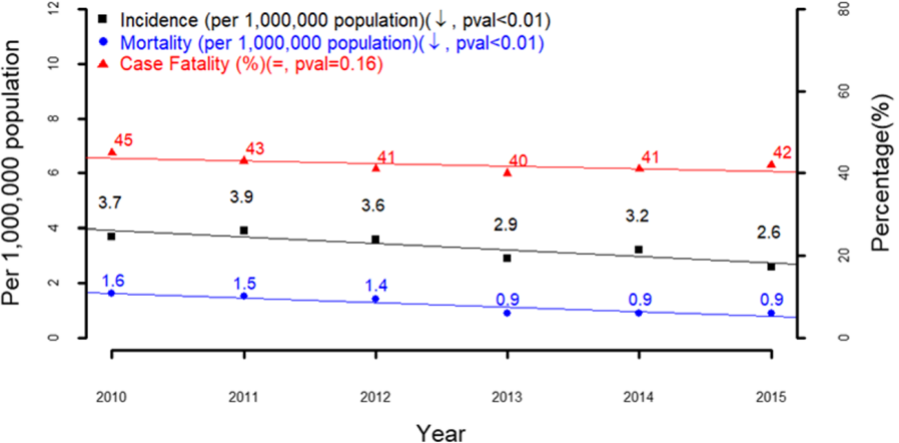
Incidence, mortality and case fatality of pneumoccocal meningitis in France from 2010 to 2015

### F-100 Poor outcome associated with Mucormycosis in critically-ill hematological patients: Results of a multicenter study

#### Matthieu Jestin^1^, Adrien Mirouse^1^, Jean-Jacques Tudesq^1^, Yannick Hourmant^1^, Fabrice Bruneel^2^, Julien Mayaux^3^, Martin Murgier^4^, Frédéric Pene^5^, Eric Mariotte^6^, Virginie Lemiale^6^, Lara Zafrani^6^, Elie Azoulay^6^, Michaël Darmon^6^, Sandrine Valade^6^

##### ^1^Service de Médecine Intensive et Réanimation, Hôpital Saint-Louis, Paris, France; ^2^Service de réanimation médico-chirurgicale, Centre hospitalier de Versailles, Le Chesnay, France; ^3^Service de Pneumologie, Médecine Intensive et Réanimation, Hôpital Universitaire Pitié-Salpêtrière, Paris, France; ^4^Service de Réanimation polyvalente, Centre hospitalo-universitaire de Saint-Etienne, Saint-Etienne, France; ^5^Service de Médecine Intensive et Réanimation, Hôpital Cochin, AP-HP, Paris, France; ^6^Service de Médecine Intensive et Réanimation, Hôpital Saint-Louis, Paris, France

###### **Correspondence:** Matthieu Jestin ^(^m_jestin56@hotmail.com)

*Ann. Intensive Care* 2020, **10 (Suppl 1):**F-100

**Rationale:** Mucormycosis is an emerging fungal infection, especially in patients with hematological malignancies. Although this infection may lead to multi organ failure, no study has been dedicated to critically ill patients with hematological malignancy. The primary objective was to assess outcome in this setting. The secondary objective was to assess prognostic factors.

**Patients and methods:** This retrospective cohort study was performed in 5 ICUs. Critically ill adult patients with hematological malignancies and mucormycosis were included between 2002 and 2018. Mucormycosis was classified as “probable” or “proven” regarding EORTC criteria. Variables are reported as median [IQR] or number (%). Adjusted analysis was performed using Cox Model.

**Results:** Twenty-six patients were included with a median age of 38 years [IQR, 26–57]. Acute leukemia was the most frequent underlying disease (n = 13, 50%). Nine patients (35%) were allogeneic stem cell transplantation (SCT) recipients. Nineteen patients (73%) had neutropenia and 16 patients (62%) had received steroids. The main reason for admission was acute respiratory failure (n = 14, 54%) followed by shock (n = 5, 19%). The median SOFA score at admission was 7 [IQR, 5–8] points. Only 3 patients (11%) had received prior anti-fungal prophylaxis effective against mucorales. Mucormycosis was “proven” in 14 patients and “probable” in 12 patients. Diagnosis was made by histopathologic examination in 14 patients, direct microscopy or culture in 5, and polymerase chain reaction in 7. *Rhizopus* and *Mucor* were the most frequent documented species. Seven patients (27%) had concurrent *Aspergillus* infection. Mucormycosis was diagnosed 1 day [− 4 to + 6] after ICU admission. Ten patients (38%) had pulmonary involvement whereas five patients (19%) had rhino-cerebral involvement. Infection was disseminated in eight patients (31%). Twenty-two patients (85%) were treated with liposomal amphotericin B. Twelve patients (46%) received antifungal combination including posaconazole in 7. Eight patients (31%) underwent curative surgery. Multiple organ failure was frequent, 21 patients (81%) requiring invasive mechanical ventilation (IMV), 19 (69%) vasopressors, and 9 (35%) renal replacement therapy. ICU and hospital mortality rates were 77% and 88%, respectively. Only two patients were alive at day 90. Three variables were associated with mortality in a Cox model including allogeneic SCT (HR 4.84 [95% IC 1.64–14.32]; figure), SOFA score (HR 1.19 [95% IC 1.02–1.39]) and dual therapy (HR 3.02 [95% IC 1.18–7.72]) (Fig. 1).

**Conclusion:** Mucormycosis is associated with a high mortality rate in patients with hematological malignancies, especially in allogeneic SCT recipients. Futility of ICU management in these patients is to be considered and strategies aiming to improve these patients’ outcome are urgently needed.

**Compliance with ethics regulations**: Yes.

**Fig. 1:**
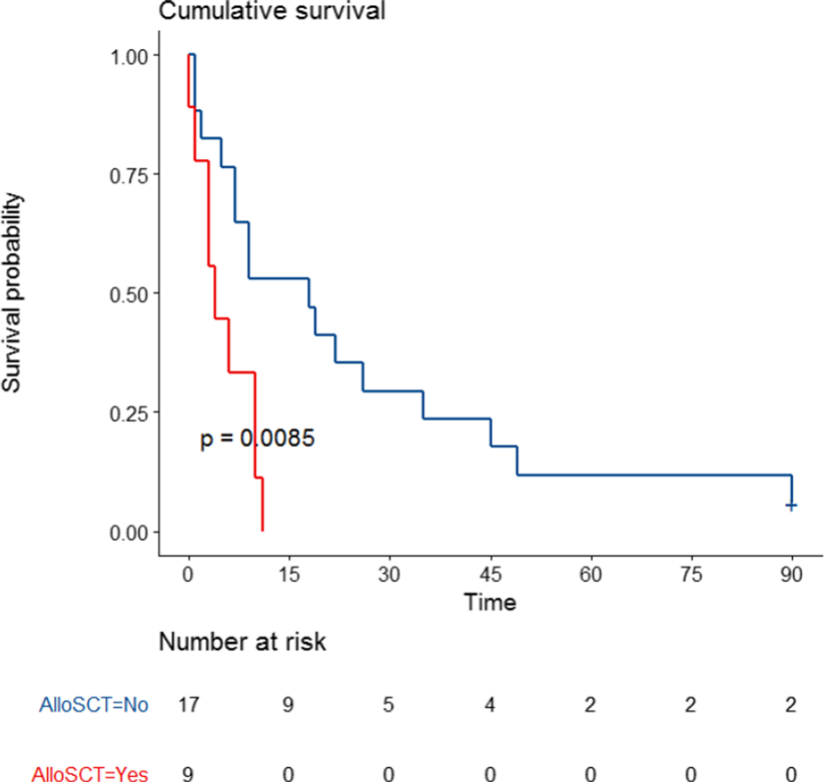
Kaplan-Meier curve reporting unadjusted influence of allogenic stem cell transplantation (alloSCT)

### F-101 Prediction of clinical phenotypes with biomarkers during presumed sepsis: a non-supervised analysis of the Captain cohort

#### Benoit Misset^1^, François Philippart^2^, Virginie Moucadel^3^, Mariana Parlato^4^, Catherine Fitting^5^, Jean-Pierre Bedos^6^, Jean-Luc Diehl^7^, Olfa Hamzaoui^8^, Djillali Annane^9^, Didier Journois^7^, Jean-Marc Cavaillon^5^, Joël Coste^7^

##### ^1^CHU Sart-Timan, Liège, Belgium; ^2^GHPSJ, Paris, France; ^3^Biomérieux, Lyon, France; ^4^Inserm, Paris, France; ^5^Pasteur Institute, Paris, France; ^6^CH, Versailles, France; ^7^APHP, Paris, France; ^8^APHP, Clamart, France; ^9^APHP, Garches, France

###### **Correspondence:** Benoit Misset (benoit.misset@chuliege.be)

*Ann. Intensive Care* 2020, **10 (Suppl 1):**F-101

**Rationale:** Sepsis is a life-threatening organ dysfunction caused by a dysregulated host response to infection. Several mediators, alone or in combination, were proposed to characterize individual response, but none was proven to have good external validity. The aim of this work was to establish whether some combinations are linked to clinical phenotypes in patients with presumed sepsis, using the data collected in the Captain multicenter cohort which methods and first results were previously published (Parlato, ICM 2018).

**Patients and methods:** 279 patients were prospectively included at the time of sepsis criteria, 188 (67%) of whom with a secondary confirmed infection. Community acquired pneumonia was causal in 70% of infections. SAPS2 score = 55 points [50–61], age = 65 years [57–78], male sex = 64%. 203 patients were followed for more than 3 days, in whom usual ICU clinical and biological parameters were collected, as well as 29 plasma biomarkers and 10 leucocyte associated RNAs. Patients were clinically classified according to their acute severity (SOFA score, serum lactate), confirmed initial infection, outcome (secondary infection occurrence, ICU survival). Non-supervised principal component analysis of the maximal values of biomarkers assessed on first 2 days of sepsis, and Varimax rotation technique of the selected components using SAS software.

**Results:** 203 patients, med SOFA day1 = 5 pts, med serum lactates day 1 = 1.5 mEq/L, bacterial infection = 141 (69%), *Enterobacteriaceae* infection = 45 (22%), VAP and/or bacteremia after day 5 = 30 (15%), alive at ICU d/c = 146 (72%). Five components explain 57% of the variance of the biomarkers. The first component (26% of the variance) was not linked to the clinical predetermined phenotypes. The second component (11% of the variance) was principally made of HLA-DR RNA, CD74 RNA and CX3CR1 RNA, and linked to a lower initial severity (r = − 0.32, p = 0.0001), a less frequent confirmation of initial infection (p = 0.0001), a lower occurrence of pneumonia or bacteremia (p = 0.02) or death (p = 0.008).

**Conclusion:** In our cohort, using non supervised analysis, we could separate a biomarker association linked to lower initial severity, lower rate of a bacterial cause to sepsis, and better outcome. The markers found are among those which are regularly considered as describers of the peripheral alteration of the immune system observed during sepsis (Pachot, CCM 2005; Friggeri, CC 2016; Peronnet ICM 2017).

**Compliance with ethics regulations**: Yes.

### F-102 Pro-adrenomedullin and Procalcitonin serial levels in predicting infection relapse or superinfection and outcome in ICU—a post hoc analysis of the PRORATA biobank

#### Lila Bouadma^1^, Tiphaine Robert^1^, Charles-Edouard Luyt^2^, Philippe Montravers^3^, Carole Schwebel^4^, Stephane Ruckly^1^, Jean-François Timsit^1^

##### ^1^Hôpital Bichat Claude Bernard, Paris, France; ^2^Hôpital de la Pitié Salpêtrière, Paris, France; ^3^Hôpital Michallon, Paris, France; ^4^Hôpital Michallon, Grenoble, France

###### **Correspondence:** Lila Bouadma (lila.bouadma@aphp.fr)

*Ann. Intensive Care* 2020, **10 (Suppl 1):**F-102

**Rationale:** Prorata trial (1) compared a standard of care to a procalcitonin (PCT) oriented use of antimicrobials for sepsis in 8 iCUs. Serial blood samples were biobanked in 4/87 ICUs (455/621 patients enrolled for pro-adrenomedullin (ProADM) and PCT concentrations).

**Patients and methods:** The aim of the study was to evaluate the respective impact of serial PCT and proADM measurements in predicting relapse or superinfection and death on day 28*. Relapse was defined as the growth of one or more of the initial causative bacterial strains (i.e., same genus, species) from a second sample taken from the same infection site at 48 h or more after stopping of antibiotics, combined with clinical signs or symptoms of infection. Superinfection was defined as the isolation from the same or another site of one or more pathogens different from that identified during the first infectious episode, together with clinical signs or symptoms of infection. Methods: Outcomes of interest were recurrence or reinfection, D28 mortality, or both. Data are presented as median [interquartile ranges] or n (%) as appropriate. ProADM and PCT were entered in a multivariate Cox model as time dependent variables. Best thresholds were estimated by maximizing the HR of death for both biomarkers and from available literature (2.3).

**Results:** Of the 455 patients included (age: 63 [54–74] years, SAPS II: 47 [35; 60] points and SOFA: 8 [4–11] points at amission, medical admission: 427 (95%), immunocompromised: 81 (18%), on mechanical ventilation 306 (67%), PCT and ProADM at inclusion were 2 [0.6–9.3] ng/mL and 1.5 [0.4–2.7] nM/L respectively. 173 (38%) patients developed a first episode of recurrence or supereinfection after a median delay of 11 days [8–16] and 98 (22%) died before d28. The HR maximization process proposed an optimal cut point of 1 ng/mL for PCT and 2 nM/L for Pro ADM to predict d28 death. In the multivariate Cox model, both PCT and ProADM were associated with death but not with relapse or superinfection (Table 1).

**Conclusion:** Conclusion: Both serial measurements of PCT and ProADM are independent predictors of death in patients treated for sepsis in ICU. Our study confirmed the use of 2 nM/L as a good prognosis cut point for ProADM. 1.

**Compliance with ethics regulations**: Yes.

**Table 1:**
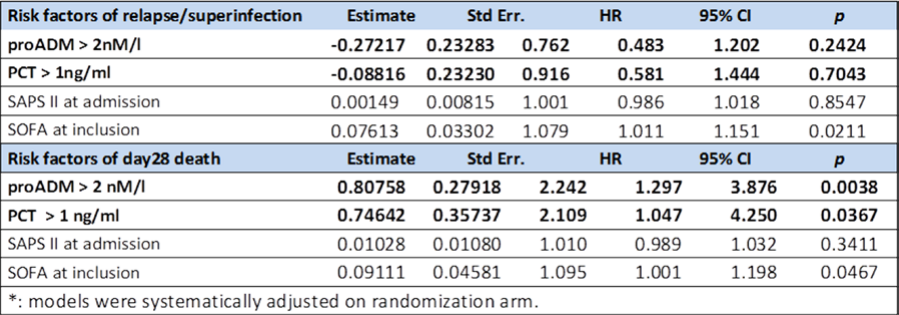
Multivariate Cox model

**References**
1. Bouadma L et al.—Lancet 2010; 375: 463–74 2.
2. Maisel A et al. J Am Coll Cardiol 2011;58:1057–67. 3.
3. Ara-Somohano C et al.—Minerva Anesthesiol 2017 Aug;83 (8):824–835.

### F-103 Performance of repeated (1-3)-β-d-glucan measures, a post hoc analysis of the EMPIRICUS Randomized Clinical Trial

#### Claire Dupuis^1^, Clément Le Bihan^1^, Muriel Cornet^2^, Stephane Ruckly^3^, Carole Schwebel^2^, Lila Bouadma^1^, Elie Azoulay^4^, Jean-François Timsit^1^

##### ^1^Réanimation médicale et infectieuse, CHU Bichat, APHP, Paris, France; ^2^Mycologie-Parasitologie, CHU Grenoble, Grenoble, France; ^3^UMR 1137, IAME, équipe Descide, Université de Paris, Paris, France; ^4^Réanimation médicale, CHU Saint Louis, APHP, Paris, France

###### **Correspondence:** Claire Dupuis (cdup83@gmail.com)

*Ann. Intensive Care* 2020, **10 (Suppl 1):**F-103

**Rationale:** The performance of serum (1-3)-β-d-glucan (BDG) and its evolution to predict the occurrence of invasive fungal infection (IFI) in a high risk non immunocompromized population remains to be determined (1).

In a post hoc analysis of the EMPIRICUS Randomized Clinical Trial (2), we aimed to assess the prognostic value of repeated measures of BDG on the occurrence of invasive fungal infections.

**Patients and methods:** Non-neutropenic, non-transplanted, critically ill patients with ICU-acquired sepsis, multiple *Candida* colonization, multiple organ failure, exposed to broad-spectrum antibacterial agents, and enrolled between July 2012 and February 2015 in 19 French ICUs were included. BDG were collected in ICU at day 0, 3, 7, 14 and 28 after inclusion. A value Time 0 of more than 80 pg/mL, 250 pg/mL and an increase by more than 24% from the previous measurement (threshold of measurement error) were assessed at baseline and overtime. For that purpose, we conducted cause specific hazard models with death as a competing risk. We also planned subgroup analyses on the placebo and the micafungin groups. Cumulative risk (CumRisk) of IFI at day 28 were derived from models.

**Results:** 234 patients were included: age: 64.2 years [53.4; 73.7], SAPS II: 48 points [38; 57]. 52 (22.2%) were surgical patients. The delay between ICU admission and trial inclusion and the corresponding first measurement of BDG was 10 days [7; 16] for a result of 95.6 pg/mL [41.9; 200.7]. 114 patients received micafungin. 28 patients presented an IFI and 68 (29.1%) patients died at day 28. Finally, only BDG at baseline over 80 pg/mL was associated with an increased risk of IFI. We found those results in the whole cohort (CSHR (IFI) = 4.98 [1.73; 14.35]; CSHR (Death) = 1.28 [0.75; 2.17], with a CumRisk of IFI (CumRisk IFI) of 19.8% [19.5; 20.0] (BDG > 80 pg/ml) versus 5.24% [5.1; 5.37] (BDG < 80 pg/ml)) (Fig. 1) and in the placebo subgroup (CSHR (IFI) = 6.41 [1.47; 28.06]; CSHR (Death) = 1.09 [0.52; 2.27], CumRisk IFI (BDG > 80) = 25.9% [25.1; 26.6] versus CumRisk IFI (BDG < 80) = 5.4% [5.1; 5.7]), but not in the micafungin sub group (CSHR (IFI) = 3.55 [0.77; 16.46], CSHR (Death) = 1.53 [0.71; 3.31], CumRisk IFI (BDG > 80 pg/ml) = 13.4% [13.2%; 13.8%] versus CumRisk IFI (BDG < 80 pg/ml) = 5% [4.7; 5.2]). Neither a BDG > 250 pg/mL, nor an increase by 24% of BDG over time were associated with the occurrence of IFI. Similar results were found in the placebo subgroup.

**Conclusion:** Among high risk patients, a first measurement of BDG over 80 pg/mL was highly associated with the occurrence of IFI. Neither a cut-off of 250 pg/mL, nor repeated measurements of BDG over time seemed to be useful to predict the occurrence of IFI. The cumulative risk of IFI in the placebo group if BDG > 80 pg/ml is 25.39% questioning about the potential interest of empirical therapy in this subgroup.

**Compliance with ethics regulations**: Yes.

**Fig. 1:**
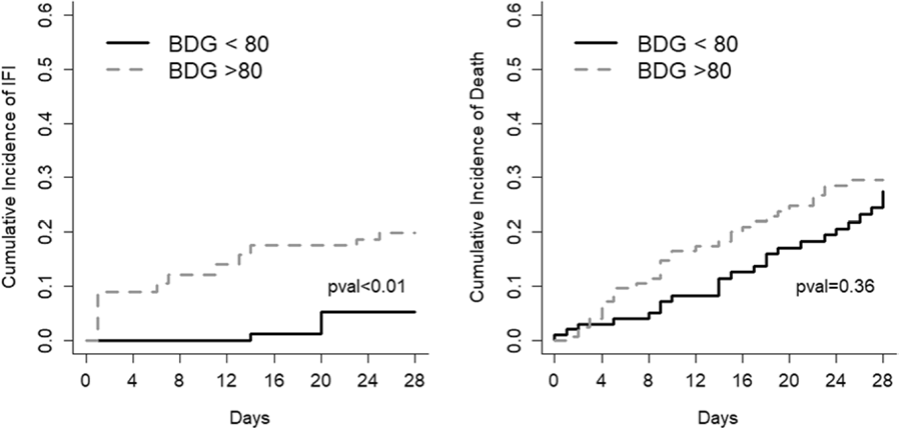
Cumulative incidence of invasive fungal infection and death depending on the serum (1-3)-β-d-glucan (BDG) level at baseline

**References**
1. Clancy, J Clin Microbiol. 2018.
2. Timsit, JAMA. 2016.

### F-104 Prehospital measurement of lactatemia and blood culture drawing for septic shock

#### Barbara Alves, Benoit Vivien, Romain Jouffroy

##### Anesthesia and Intensive Care Unit—SAMU 75—Assistance Publique des Hôpitaux de Paris –Necker Enfants Malades Hospital, Paris, France

###### **Correspondence:** Barbara Alves (brb.alv@gmail.com)

*Ann. Intensive Care* 2020, **10 (Suppl 1):**F-104

**Rationale:** Since the 2016 Sepsis-3 conference, the distinction between sepsis and septic shock is based on blood lactate value. Septic shock may be encountered in the pre-hospital setting. In order to reduce the mortality, the precocity of treatments implementation has been emphasized, particularly early antibiotic administration. Prior antibiotic administration, and blood culture drawing must be performed. The aim of this survey was to clarify the capabilities of French prehospital emergency service (PEMS) to measure blood lactate and to draw blood culture prior to hospital admission for septic shock.

**Patients and methods:** We performed an electronic survey of 26 auto-questions addressed to the deputy chair of the French PEMS in 2018.

**Results:** Sixty PEMS (60%) participated in the survey. Twenty-five percent are able to measure blood lactate and 45% are able to draw blood culture in the prehospital setting. Ninety-five percent declared lactate measurement is helpful in assessing severity. Ninety percent claimed that the lactate value influences the hospital facility, emergency department vs. intensive care unit. Twenty-eight percent believe that the impossibility to draw blood culture precludes prehospital antibiotic administration. Sixty-three percent estimate that a protocol for septic shock management would be beneficial.

**Conclusion:** Few French PEMS are able to measure lactate and draw blood culture in the prehospital setting. The impact of blood lactate measurement and blood culture drawing by PEMS on septic shock outcome requires further studies.

**Compliance with ethics regulations**: Yes.

### F-105 Head Injury Imaging-Timing is Critical

#### Mufaddal Jivanjee

##### ^1^London North West Healthcare NHS Trust, London, UK

###### **Correspondence:** Mufaddal Jivanjee (mufaddal.jivanjee@nhs.net)

*Ann. Intensive Care* 2020, **10 (Suppl 1):**F-105

**Rationale:** Head injury is a common cause of morbidity and mortality in the first four decades of life, accounting for approximately 200,000 annual hospital admissions in the United Kingdom. The majority of patients recover without intervention, however some may develop a long-term disability or even die. The early detection of pathology is therefore absolutely critical in determining patients’ prognosis, helping to provide appropriate timely management. The National Institute for Health and Care Excellence (NICE) adult head injury guidelines, recommend that head injuries with specific risk factors should have a CT scan within 1 h of risk factors being identified. Furthermore the provisional report should be made available within 1 h of the scan. This audit assessed the compliance of staff to the NICE adult head injury guidelines.

**Patients and methods:** The previous 40 adult CT head scans, requested due to head injury, from the Emergency Department (ED) at London North West Healthcare NHS Trust were analysed for compliance to the NICE guidelines. The standards measured were: (1) Time from request of scan to completion of scan should be within 1 h; (2) Time from completion of scan to publication of provisional report should be within 1 h. The locally agreed target for both standards was 100%.

**Results:** On review of the 40 CT scans, 32 (80%) were completed within 1 h of request. From the 8 scans (20%) not completed within the hour, 4 were due to porter unavailability, 1 due to an uncooperative patient and the remaining 3 reasons were not clear from documentation. Following completion of the scan, 38 scans (95%) were provisionally reported within 1 h.

**Conclusion:** This study highlighted a good compliance by hospital staff in ensuring patients with head injuries are managed appropriately, following detection of risk factors indicating a CT head scan. Having said that, the locally agreed targets were just short of being met. One factor resulting in delayed scans was porter availability. An intervention recently introduced is the use of the “e-portering” application, which will endeavour to save time for referrers requesting porters and allow patient tracking. It is also worth educating porters, via email bulletins, on the importance of priority scans, such as CT head following injury. Furthermore, the findings of the audit were relayed to the radiology department to help improve reporting times and to the ED to re-emphasize prompt requesting of CT head scans when clinically indicated.

**Compliance with ethics regulations**: Yes.

### F-106 Does continuous insufflation of oxygen without intubation improve mortality after cardiac arrest?

#### Arnaud Gaillard, Cécile Ricard, Guillaume Berthet, Olivier Baptiste, Vincent Peigne

##### Haute Savoie Fire Department, Meythet, France

###### **Correspondence:** Arnaud Gaillard (arnaud.gaillard@sdis74.fr)

*Ann. Intensive Care* 2020, **10 (Suppl 1):**F-106

**Rationale:** Continuous insufflation of oxygen (CIO) performed with specific endotracheal tube during cardiopulmonary resuscitation (CPR) is as effective as intermittent ventilation on endotracheal tube. Experimental data suggest that CIO improves the efficacy of external cardiac massage and reduces gastric dilatation. As endotracheal intubation is a cause of CPR interruption and requires skilled staff, a specific device has been developed to perform CIO without intubation. This device has been implemented progressively in our fire department since 2015. We evaluated this practice.

**Patients and methods:** Longitudinal study comparing the patients with out-of-hospital cardiac arrest managed by our fire department with CIO or bag-valve ventilation between January 2015 and April 2019. Patients who received mechanical chest compression were excluded. The main outcome was hospital survival. Secondary outcomes were the return of spontaneous circulation (ROSC) and CPR quality. Univariate and multivariate analysis was performed in the whole cohort and in the sub-groups of patient with shockable and non-shockable rhythms to take into account factors associated with survival (shockable rhythm, witness, age).

**Results:** Among the 793 patients included, 262 have been ventilated with CIO and 531 with valve-bag. The mortality was similar in the two groups (CIO: 85.1% valve-bag: 88.1% p 0.23). Mortality and ROSC were not associated with CIO in the multivariate analysis (odds ratio OR 1.19 95%-confidence interval CI95 [0.75–1.9] and 0.97 [0.59–1.6], respectively). CPR quality was better with CIO than with valve-bag regarding CPR fraction (ratio of duration of chest compressions on total duration of CPR, 84 versus 76% p < 0.0001) and adequacy to the guidelines of the rhythm and depth of chest compressions (70% vs 50% p < 0.0001 and 47% vs 38% p < 0.0001, respectively). In both subgroups of patients, CPR quality was still better with CIO than with valve-bag. In the subgroup of patients with shockable rhythm, univariate analysis showed a lower mortality among the 59 patients with CIO than among the 140 patients with valve-bag (57.6% vs 82.9% p < 0.001) but this difference was not confirmed by the multivariate analysis (OR 0.4 CI95 [0.10–1.03], p 0.06).

**Conclusion:** CIO without intubation is associated with an improvement of CPR quality but neither with mortality nor return of spontaneous circulation in case of out-of-hospital cardiac arrest.

**Compliance with ethics regulations**: Yes.

### F-107 Can New Wearable Technology significantly increase the Efficacy Cardiopulmonary Resuscitation? A controlled, randomized trial testing

#### Michele Musiari^1^, Samuele Ceruti^2^, Roberta Sonzini^3^, Luciano Anselmi^4^, Andrea Saporito^4^, Xavier Capdevila^5^, Tiziano Cassina^6^

##### ^1^Hôpital Fribourgeois Regional (HFR), Fribourg, Switzerland; ^2^Hôpitaux Universitaires de Genève, Genève, Switzerland; ^3^Clinica Luganese, Lugano, Switzerland; ^4^Ente Ospedaliero Cantonale, Bellinzona, Switzerland; ^5^Centre Hôspitalier Universitaires, Montpellier, France; ^6^CardioCentro Ticino, Lugano, Switzerland

###### **Correspondence:** Michele Musiari (michelemusiari@bluewin.ch)

*Ann. Intensive Care* 2020, **10 (Suppl 1):**F-107

**Rationale:** Cardiovascular accidents are a leading cause of death. A cardiopulmonary resuscitation (CPR) of quality has well shown that can reduce the mortality; despite this, survival rate has not changed significantly during last years. The aim of this study is to test a new wearable glove to provide lay people with instructions during out-of-hospital CPR.

**Patients and methods:** We performed a blinded, controlled trial on an electronic mannequin AmbuMan to test the performance of adult volunteers, non-healthcare professionals performing a simulated CPR both, without and with glove, following the glove instructions. The group without glove, also called “no-glove” is intended as control group. Each compression performed on the electronic mannequin AmbuMan was recorded by a connected laptop computer, drawing a depth frequency curve over the time. Primary outcome was to compare the accuracy of the two simulated CPR sessions in terms of depth and frequency of chest compressions performed by the same lay volunteers. Secondary outcome was to compare the decay of performance and percentage of time in which the candidate performed accurate CPR. Finally, the participants were asked if the glove was useful for CPR maneuvers. The difference between the two groups in regard to change in chest compression depth over time due to fatigue, defined as decay were also analyzed.

**Results:** 571 chest compressions were included: 293 in control group, 278 in glove group (Table 1). Mean depth of compression in the control group was 55.17 mm versus 52.11 mm in the glove-group (p = 0.000016). Compressions with an appropriate depth were not statistically different (81.9% vs 73.6%, p = 0.017). Mean frequency of compressions in the group with glove was 117.67 rpm vs 103.02 rpm in the control group (p < 0.00001). The percentage of compression cycles with an appropriate rate (> 100 rpm) was 92.4% in the group with the glove versus 71% in the control group, with an observed difference of 21.4% between the two groups, which was statistically significant (p < 0.0001, CI = 95%). A mean reduction over time of compressions depth of 5.3 mm (SD 10.28) was observed in the control group versus a mean reduction of 0.83 mm in the group wearing the glove (SD 9.91), but this mean difference in the decay of compressions delivery was not statistically significant (f-ratio = 5.680, SS = 579.61, df = 1, MS = 579.61, p = 0.018).

**Conclusion:** The visual and acoustic feedbacks provided by the device were useful in dictating the correct rhythm for non-healthcare professionals, translating in a significantly more accurate CPR.

**Compliance with ethics regulations**: Yes.

**Fig. 1:**
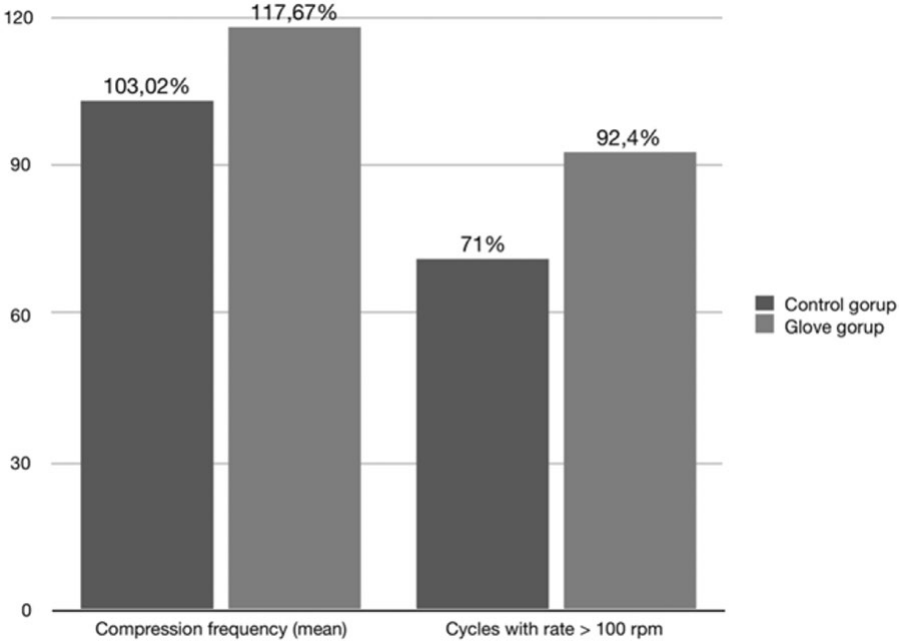
Differences about compression frequency and cycles with rate greater than 100 rpm during CPR between the two groups. All values are intended as mean values

### F-108 Early DiaGnosis of Anoxic brain injury for Resuscitated patients (EDGAR): value of continuous aEEG, quantitative automated pupillometry and Cardiac Arrest Hospital Prognosis (CAHP) score for early neurologic prognosis after adult cardiac arrest

#### Sébastien Gette, Rostane Gaci, Guillaume Louis, Cyril Cadoz, Damien Barraud, Boris Glavnik, Guillaume Jay, Julie Larde, Nicolas le Berre, Nouchan Mellati, Emmanuel Novy, Jessica Perny, Adeline Perrein, Yoann Picard, Kevin Podrez, Béatrice Schnitzler, Christophe Goetz, Serge Le Tacon

##### Hôpital Mercy CHR Metz-Thionville, Metz, France

###### **Correspondence:** Sébastien Gette (s.gette@chr-metz-thionville.fr)

*Ann. Intensive Care* 2020, **10 (Suppl 1):**F-108

**Rationale:** Neuroprognostication after cardiac arrest (CA) is a crucial issue and current guidelines recommend delayed multimodal approach. We aimed to describe reasons for death in a prospective cohort of CA patients and evaluate the diagnostic accuracy of early combined neurological prognostication tools such as automated pupillometry (AP), continuous amplitude electroencephalography (aEEG) and Cardiac Arrest Hospital Prognosis (CAHP) score performed 24 h after return of spontaneous circulation (ROSC).

**Patients and methods:** We set up a monocentric prospective cohort of adult CA patients admitted in ICU after sustained ROSC and collected data according to Utstein style recommendations. Reasons for death were described under recently proposed classification: withdrawal of life-sustaining therapies (WLST) for neurological reasons, WLST due to comorbidities, refractory shock or recurrence of sudden CA or respiratory failure. For patients who kept abnormal neurologic state after ROSC with Glasgow Coma Scale < 15, we analysed accuracy of early neuroprognostication tools (AP, aEEG and CAHP score) to predict poor neurological outcome, i.e. Cerebral Performance Category (CPC) > 2 at hospital discharge.

**Results:** 144 patients were admitted after sustained ROSC from CA during the period (31.08.2018 to 10.07.2019). In-hospital mortality was 51%. Neurological WLST was the first reason for death (62%). Exhaustive early neuroprognostication with AP, aEEG and CAHP score was available for 75 patients. Among them, poor neurological outcome at hospital discharge (CPC > 2) was observed for 39 patients (52%). AP < 13% performed at H24 always predicted poor neurological outcome (PPV 100%; CI 95% [0.84–1]). Type I aEEG was associated with good neurological outcome (PPV 82%; CI 95% [0.62–0.92]). Prognostic values of combined tests (CAHP score + AP + aEEG) were respectively PPV 0.82 (CI 95% [0.67–0.91]) and NPV 0.81 (CI 95% [0.62–0.92]) if at least one positive, PPV 0.97 (CI 95% [0.8–1]) and NPV 0.70 (CI 95% [0.54–0.82]) if at least 2 positives, PPV 1 (CI 95% [0.87–1]) and NPV 0.55 (CI 95% [0.42–0.68]) if 3 positives.

**Conclusion:** Most deaths after CA resuscitation occur after WLST because of poor neurologic prognosis. A stepwise model with automated pupillometry and then aEEG if AP > 13% could discriminate about the neurologic prognosis at hospital discharge 72% of patients as soon as 24 h after ROSC (Fig. 1). This strategy would falsely misclassificate 4% of patients in a good neurologic outcome category. Other survivors (24%) should then be investigated with further classical delayed neuroprognostication tools.

**Compliance with ethics regulations**: Yes.

**Fig. 1:**
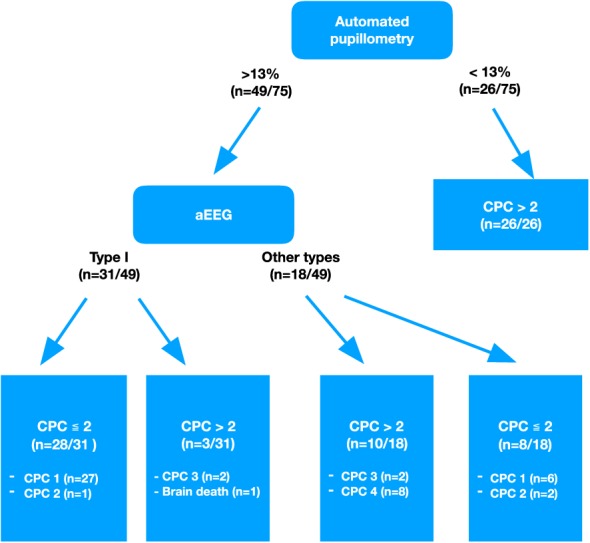
Stepwise model for early neuroprognostication

### F-109 Impact of delayed specialized advice on the quality of care at the emergency department

#### Hadil Mhadhbi, Meher Arafa, Mounir Hagui

##### ED HMPIT, Tunis, Tunisia

###### **Correspondence:** Hadil Mhadhbi (hadil.mhadhbi@gmail.com)

*Ann. Intensive Care* 2020, **10 (Suppl 1):**F-109

**Rationale:** Management delay is one of the determining factors in the assessment of emergency department quality of care. Asking for a specialized advice seems to increase the time of delay. Our study aimed at measuring the delays in obtaining specialized advice and identify their major causes.

**Patients and methods:** We conducted a prospective study over the period of 1 month. We included all adult patients presenting to the emergency department who required specialized advice. Data of all patients was collected. Waiting times and influencing factors were studied.

**Results:** A total of 75 patients were included. The main reason for calling for a specialized advice was to ask for a department transfer in 68% of cases. The time of the day when specialized advice was solicited (n (%)): in the morning 51 (68); in the afternoon 17 (23); in the evening 9 (12). The main solicited specialties were (n (%)): visceral surgery 18 (24), trauma medicine 15 (20), cardiology 10 (13), urology 5 (7), and pulmonology 5 (7). The average waiting time between calling for and getting the specialized advice was 176 ± 115 min. Seventy-five percent of the specialized advice was obtained within 1 h. The causes of the delay were (n (%)): Physician busy in the operating room 22 (39), unreachable physician 9 (12), physician in the outpatient clinics 7 (10). The impact of the waiting time was (n (%)): conflict 20 (27), worsening patient state 4 (5). The average time between calling for the specialized advice and reaching a management decision was 210 ± 127 min.

**Conclusion:** The increasing length of stay of patients in the ED is strongly correlated to the delay in obtaining specialized advice. The implementation of a strategy to reduce the waiting time is necessary to avoid overcrowding the emergency departments and provide optimal care.

**Compliance with ethics regulations**: Yes.

### F-110 Hypnosis to reduce anxiety and pain during prolonged ICU stay for Guillain-Barré syndrome (GBS)

#### Sylvie Calvat^1^, Sylvie Colombani^2^, Christophe Cracco^1^, Charles Lafon^1^, Stéphane Rouleau^1^, David Schnell^1^

##### ^1^Centre Hospitalier d’Angoulême, Angoulême, France; ^2^Institut Bergonié, Bordeaux, France

###### **Correspondence:** Sylvie Calvat (sylvie.calvat@ch-angouleme.fr)

*Ann. Intensive Care* 2020, **10 (Suppl 1):**F-110

**Rationale:** Hypnoanalgesia has been used since few years to reduce ICU-patients physical and psychological discomfort during invasive procedures. However, feasibility of overall well-being management of intubated patients with hypnosis has not been described.

**Patients and methods:** We report here the hypnotic accompaniment of a 16-year old patient without significant medical history hospitalized in our ICU for a severe GBS during 3 months. The GBS was diagnosed by electrophysiological study and immunologic markers. Patient had nearly complete paralysis of all extremities, but no facial or bulbar muscles. He received mechanical ventilation during 87 days, including weaning time. Tracheotomy was performed at day 15. Sedative drugs were stopped 2 days after intubation. Hypnosis sessions were started very early after intubation by one of our trained intensivist. Eight hypnotic sessions of hypnoanalgesia or hypnotherapy were performed after approval of the patient and his parents. Time distribution is reported in Fig. 1. First and second sessions were performed in order to induce relaxation and reduce anxiety. Following sessions were dedicated to: 1) decrease pain intensity (initially neuropathic, then induced by physiotherapy), 2) attenuate the negative perception of paralysis, 3) reduce the discomfort of tracheotomy 4) promote the belief in healing 5) facilitate swallowing exercises. Furthermore the patient was quickly trained to use self-hypnosis in order to dissociate him from pain, anxiety and ICU pollutions.

**Results:** Feasibility of hypnosis was judged satisfactory by the operating physician, despite mechanical ventilation. After extubation, final debriefing with the patient indicates that the most efficient sessions were those focused on anxiety disorders (using the suggestion of a safe place) and suggestions of mobility (using a mangas metaphor). The patient reported very positive perception of hypnosis use. He explained that self-hypnosis was effective to reduce many discomfort. He used it frequently (generally twice a day) for a puff of anxiety or before enoxaparin injection.

**Conclusion:** Our observation suggests that hypnosis seems feasible in ICU—awake patients and may be an interesting way to improve their ICU lived experience in combination with validated measures. Further investigations are needed to evaluate its effects on post-traumatic-stress disorder.

**Compliance with ethics regulations**: Yes.

**Fig. 1:**
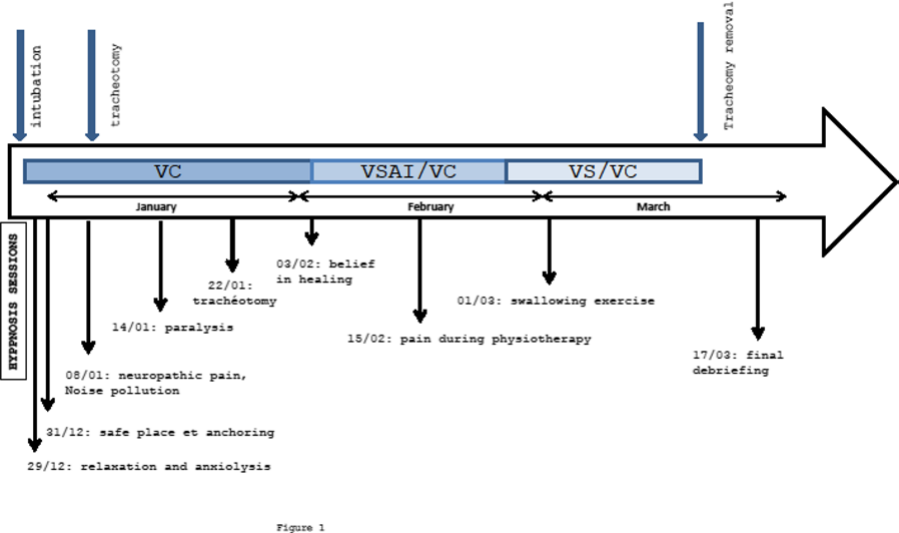
Time distribution of the eight hypnotic sessions

### F-111 Resuscitation Hypnosis: Benchmarks for Effective Practice

#### Gwenael Prat^1^, Anne Renault^1^, Christelle Teiten^1^, Montaine Lefevre^2^, Erwan L’her^1^

##### ^1^Medical Intensive care unit, CHRU Brest, Brest, France; ^2^Medical Intensive care unit, CH Morlaix, Morlaix, France

###### **Correspondence:** Gwenael Prat (gwenael.prat@chu-brest.fr)

*Ann. Intensive Care* 2020, **10 (Suppl 1):**F-111

**Rationale:** There is little medical reference for hypnosis in the intensive care field. Closed specialties such as anesthesia, emergency medicine can help and refer to hypnosis for certain technical procedures. Objective: To propose landmarks for a successful implementation of hypnosis by intensivists within the intensive care unit.

**Patients and methods:** This monocentric prospective observational study was performed from February 2018 to June 2019 in the 15-bed medical ICU of Brest University Hospital. Collected data were: characteristics of patients and hypnosis sessions performed, demographic data, physiological parameters (heart and respiratory rates) and objective and subjective evaluation of hypnosis sessions quality.

**Results:** 60 patients were included (mean age 55.4 ± 19 years, SAPS II 34.5 ± 14 points). 100 hypnosis sessions were performed, of which 1/3 under mechanical ventilation. Patterns of hypnosis sessions were: anxiety/comfort (53%), during a technical procedure (38%): TOE, CVC placement, thoracic drainage, upper digestive or bronchial endoscopy), initiation of noninvasive ventilation or before intubation. Most of time, the hypnotic trance was permitted by formal hypnosis techniques with travel and nature themes suggestion. Efficacy was qualitatively assessed and rated as “total effectiveness” for 60% of sessions. Qualitative evaluation by hypnotherapist, technical operator and observers was respectively 7.25 ± 1.5, 9.25 ± 0.5 and 9 ± 1/10. Heart rate decreased from 93 ± 17 to 88 ± 13 bpm and respiratory rate/min decreased from 22 ± 7 to 17.7 ± 5 rpm during sessions.

**Discussion:** After a meeting, the healthcare team carried out a brainstorming to propose hypnosis in our unit. Several difficulties were observed to explain implementation failures such as: finding competent patient, respiratory assistance, difficult communication, noisy environment, many nursing care, unexpected emergencies, etc.…). This experience allowed writing a vademecum to perform hypnosis in intensive care. Our aims are to get more trained caregivers and to integrate hypnosis during our post-resuscitation consultation, especially for post-traumatic stress.

**Conclusion:** Hypnotic tools can facilitate technical procedures and improve patients’ and caregivers’ quality of life within the ICU.

**Compliance with ethics regulations**: Yes.

### F-112 Effect of a musical intervention during central venous catheterization in an intensive care unit: the MUSIC CAT prospective randomized pilot study

#### Sophie Jacquier, Brice Sauvage, Gregoire Muller, Thierry Boulain, Mai-Anh Nay

##### CHR, Orléans, France

###### **Correspondence:** Sophie Jacquier (sophie.jacquier@chr-orleans.fr)

*Ann. Intensive Care* 2020, **10 (Suppl 1):**F-112

**Rationale:** Evaluate the effect of a musical intervention on patient anxiety during a central venous access or a dialysis catheter implantation in an intensive care unit.

**Patients and methods:** The MUSIC CAT study was a prospective, single-centre, controlled, open-label, two-arm randomized trial, conducted from February 2018 to February 2019. Central venous catheterization with musical intervention was compared to standard care, i.e., the usual procedure of central venous catheterization without listening to music. Eligible patients had to be able to hear, understand explanations and consent. Randomisation was stratified according to ventilation type (mechanical ventilation or not) and catheter site (superior vena cava or femoral vein). The MUSIC CARE^®^ (Paris, France) application was used to make the patients listen to music through headphones. Each patient chose his/her musical topic on a digital tablet, just before the catheterization. The primary outcome was the change in anxiety Visual Analogic Scale (VAS) between the beginning and the end of the catheterization procedure (T0-TF anxiety VAS). Secondary outcomes included the patient’s pain VAS at the end of the procedure (TF pain VAS).

**Results:** 31 patients were included in the standard care group versus 36 in the musical intervention group. Main reasons for admission were the need of central catheter for chemotherapy (27, 40%), and sepsis and/or shock in both groups (26, 39%). Catheters were inserted in the internal jugular vein in most cases (56, 82%) and about one-third were tunnelled in both groups. There was no between-group difference regarding median T0-TF anxiety VAS: 0 [IQR:− 3 to 0] in the standard care group versus − 1 [− 3 to 0] in the music intervention group (P = 0.20) (Fig. 1), with no significant interaction between the variables of stratification or the operator experience and the intervention. The median TF pain VAS was not statistically different between groups: 0 [0 to 4.1] in standard care group and 0 [0 to 2] in music intervention group (P = 0.60), with no significant interaction between the variables of stratification or the operator experience and the intervention.

**Conclusion:** In this first randomized pilot study of musical intervention for central venous catheterization in awake patients in the intensive care unit, the musical intervention did not reduce patients’ anxiety as compared to usual care. As the study may have been underpowered, larger size trials are needed.

**Compliance with ethics regulations**: Yes.

**Fig. 1:**
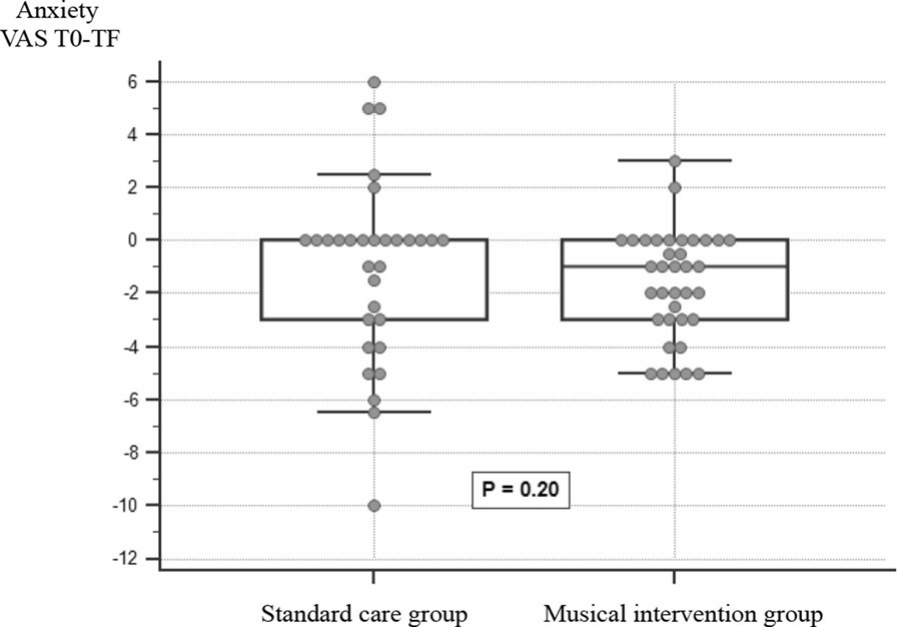
Changes in anxiety VAS T0-TF in both groups

### F-113 Sleep in non-intubated patients admitted to ICU for acute respiratory failure: A prospective observational monocenter study

#### Damien Marie^1,2^, Stephanie Barrau^1,2^, Clément Beuvon^1,2^, Faustine Reynaud^1,2^, Adrien Pépin-Lehalleur^1,2^, Christophe Rault^1,3^, Jean-Claude Meurice^1,4^, Rémi Coudroy^1,2^, Jean-Pierre Frat^1,2^, René Robert^1,2^, Xavier Drouot^1,3^, Arnaud Thille^1,2^

##### ^1^INSERM CIC 1402, Groupe ALIVE, Université de Poitiers, Poitiers, France; ^2^Réanimation Médicale, CHU de Poitiers, Poitiers; ^3^Neurophysiologie Clinique et Explorations Fonctionnelles, CHU de Poitiers, Poitiers; ^4^Pneumologie, CHU de Poitiers, Poitiers

###### **Correspondence:** Damien Marie (damien.marie01@gmail.com)

*Ann. Intensive Care* 2020, **10 (Suppl 1):**F-113

**Rationale:** Sleep is markedly altered in ICU-patients under mechanical ventilation and may be due to noise, light, patient-care activities, patient-ventilator asynchronies, or the result of acute brain dysfunction induced by sedative drugs. To our knowledge, sleep has never been studied at ICU admission before any sedation. Our study aimed at assessing sleep quality of non-intubated sedation-free patients admitted to ICU for acute respiratory failure.

**Patients and Methods:** Observational study performed in a single centre of a teaching hospital. Patients admitted to ICU for acute respiratory failure (respiratory rate ≥ 25 breaths/min and PaO_2_/FiO_2_ < 300 mm Hg under high-flow nasal oxygen) could be enrolled. Patients with hypercapnia, central nervous disease, intubated early after admission and those with a do-not-intubate order were excluded. Sleep was evaluated by complete polysomnography (PSG) that started in the afternoon following admission and was continuously performed until the next morning.

**Results:** Over a 3-year period 128 patients were screened and 44 patients were included. Among them, 10 patients were excluded for the following reasons: 1 patient was intubated shortly after PSG initiation, 1 PSG was lost, and 8 EEG recordings (18%) were stopped before midnight (electrodes turned off or loss of signal). Therefore, 34 patients in whom PSG was complete during the nocturnal period were retained in the analysis (27 males [74%], age 62 years [60–69], SAPS II 32 [24–37], PaO_2_/FiO_2_ 133 mmHg [100–186], PaCO_2_ 34 mmHg [30–39]). The recording time of polysomnography was 16 h in median [IQR 15–18] and total sleep time was 4.2 h [2.6–6.9] (28% of the total recording time). Sleep architecture was close to normal sleep with a median light sleep time (stage N1 and N2) of 60% [42–71] and a median deep sleep time of 29% [14–44]. By contrast, rapid eye movement (REM) sleep was completely absent in 13 patients (38%) and represented only 2.4% of total sleep time [0–7.8], that is 9 min [0–29]. Sleep was fragmented by numerous arousals and awakenings (11 events [9–16] per hour of sleep in median). Five of the 34 patients needed intubation after PSG (15%).

**Conclusion:** Our results suggest for the first time that patients admitted to the ICU for acute respiratory failure have a sleep time and architecture close to normal during the first night after admission, except for an almost compete disappearance of REM sleep, an essential stage of sleep.

**Compliance with ethics regulations:** Yes.

### F-114 Evaluation of Association of Secondary Brain Insults and Outcome in Convulsive Status Epilepticus: a Post Hoc Analysis of the Hybernatus Trial

#### Candice Fontaine^1^, Virginie Lemiale^2^, Matthieu Resche-Rigon^2^, Maleka Schenck^3^, Jonathan Chelly^4^, Thomas Geeraerts^5^, Aicha Hamdi^6^, Christophe Guitton^7^, Ferhat Meziani^8^, Jean-Yves Lefrant^9^, Bruno Mégarbane^10^, Hervé Mentec^11^, Cendrine Chaffaut^2^, Alain Cariou^12^, Stéphane Legriel^1^

##### ^1^Centre Hospitalier de Versailles, Le Chesnay, France; ^2^Centre Hospitalier Universitaire Saint Louis, Paris, France; ^3^Hôpitaux Universitaires de Strasbourg-Hôpital de Hautepierre, Strasbourg, France; ^4^Centre Hospitalier de Melun, Melun, France; ^5^Centre Hospitalier Universitaire de Toulouse, Toulouse, France; ^6^Centre Hospitalier De Montreuil, Montreuil, France; ^7^Centre Hospitalier du Mans, Le Mans, France; ^8^Hopitaux Universitaires de Strasbourg-Nouvel Hopital Civil, Strasbourg, France; ^9^Hopital Universitaire de Nimes, Nimes, France; ^10^Centre Hospitalier Universitaire Lariboisiere, Paris, France; ^11^Centre Hospitalier d’Argenteuil, Argenteuil, France; ^12^Centre Hospitalier Universitaire Cochin, Paris, France

###### **Correspondence:** Candice Fontaine (candicefontaine@yahoo.com)

*Ann. Intensive Care* 2020, **10 (Suppl 1):**F-114

**Rationale:** Convulsive status epilepticus (CSE) is a common neurological emergency associated with high mortality and morbidity rates. There are strong experimental data suggesting a potential impact of secondary brain insults (SBI) on outcome after CSE. However, there is no clinical proof to support this hypothesis. Our objective was to evaluate the association between SBI (mean arterial blood pressure, arterial partial pressure of carbon dioxide, arterial partial pressure of oxygen, temperature, natremia, and glycemia) at day 1 and neurological outcomes 90 days after CSE.

**Patients and methods:** This was a post hoc analysis of the HYBERNATUS multicenter open-label clinical trial randomized 270 critically ill patients with CSE requiring mechanical ventilation to either therapeutic hypothermia (32–34 °C for 24 h) plus standard care or standard care alone. 265 patients still alive at day 2 after inclusion were enrolled from March 2011 to January 2015 in 11 French medico-surgical ICUs. The primary outcome was favourable outcome 90 days after CSE defined as a Glasgow Outcome Scale score of 5.

**Results:** Median age was of 57 years [45–68]. A previous history of epilepsy was noted in 130 (49%) patients. Most episodes (173/265, 65%) occurred out-of-hospital, and 230 (87%) were witnessed from their onset. CSE was refractory in 86 (32%) patients and total seizure duration was 67 min (35–120). A favorable 90-day outcome occurred in 126 (48%) patients. Maximal glycemia value and hyperglycemia > 9.9 mmol/L at day 1 were the only SBI variables associated with outcome in univariate analysis. By multivariate analysis, age > 65 years (OR, 0.46; 95% IC, 0.26–0.83; P = 0.01), refractory CSE (OR, 0.50; 95% IC, 0.26–0.96; P = 0.04), and primary brain insult (OR, 0.50; 95% IC, 0.25–0.99; P = 0.047) were associated with an increased risk of poor outcome, and a bystander-witnessed onset of CSE (OR, 2.49; 95% IC, 1.05–5.59; P = 0.04) was associated with a decreased risk of poor outcome.

**Conclusion:** In our population, secondary brain insults were not associated with outcome in critically ill patients with convulsive status epilepticus; whereas age, bystander-witnessed onset of status epilepticus, refractory status epilepticus and primary brain insult were identified as strong predictors of 90-day functional impairment. Further studies are warranted to confirm our findings.

**Compliance with ethics regulations:** Yes.

### F-115 Acute stroke admitted to the ICU: prognosis and functional outcome

#### Thibaut Carval^1^, Charlotte Garret^1^, Benoit Guillon^2^, Amélie Seguin^1^, Maelle Martin^1^, Arnaud-Felix Miailhe^1^, Hélène Migueres^1^, Olivier Zambon^1^, Laura Crosby^1^, Jean-Baptiste Lascarrou^1^, Jean Reignier^1^, Emmanuel Canet^1^

##### ^1^Medical ICU, Nantes, France; ^2^Neurology department, Nantes, France

###### **Correspondence:** Thibaut Carval (thibautcarval@hotmail.com)

*Ann. Intensive Care* 2020, **10 (Suppl 1):**F-115

**Rationale:** Acute stroke (AS) is a leading cause of morbidity and mortality worldwide. However, data on the prognosis and functional outcome of patients with AS requiring ICU management is limited. Our purpose was to identify factors associated with good outcome (defined by a modified Rankin score (mRS) of 0–2) 6 months after ICU admission.

**Patients and methods:** Retrospective cohort of patients admitted to the medical ICU of a university-affiliated hospital between January 2014 and December 2018 and coded for acute stroke using the ICD-10 criteria. Patients with traumatic stroke and isolated subarachnoid hemorrhage were excluded.

**Results:** We identified 323 patients. Median age was 67 [54.5–77] years and 173 (53.6%) were males. Main reasons for ICU admission were coma (87%), hemodynamic instability (28.2%), acute respiratory failure (26%), and cardiac arrest (5.3%). Glasgow coma score at ICU admission was 6 [4–10] and SAPS 2 was 54 [35–64] points. Types of stroke were hemorrhagic in 248 (76.8%) patients and ischemic in 75 (23.2%). Mechanical ventilation was required in 257 patients (79.6%). Seizures occurred in 11.8% of the patients and convulsive status epilepticus in 3.1%. Pneumonia was diagnosed in 60 (18.6%) patients (aspiration pneumonia n = 20, ventilator associated pneumonia n = 40). Thrombolysis or thromboaspiration were performed in 18 (24%) patients with ischemic stroke. Surgical evacuation of expanding hematoma was performed in 21 (6.5%) patients, 19 (5.9%) had craniectomy, and 61 (18.9%) had external shunt for hydrocephalus. ICU and hospital mortality were 58.8% and 61%, respectively. Six months after ICU admission, 44 (13.6%) patients had a good outcome (mRS 0–2), 42 (13.1%) had significant disability (mRS 3–5), and 200 (61.9%) were deceased (lost follow-up n = 37, 11.5%). On multivariable analysis, age (OR 0.95 per year (0.91–0.99), p = 0.01), SAPS 2 (OR 0.95 per point (0.90–0.99), p = 0.03), and hemorrhagic stroke (OR 0.27 (0.08–0.9), p = 0.03) reduced the likelihood of good outcome (mRS 0–2) 6 months after ICU admission.

**Conclusion:** In our study, prognosis of acute stroke requiring ICU admission was poor and a good functional outcome occurred in less than 15% of the patients at 6 months. Age, severity at ICU admission, and type of stroke predicted outcome.

**Compliance with ethics regulations**: Yes.

### F-116 Hemorrhagic brain injuries in critical care units: a systematic review of prognostic models

#### Jeanne Simon-Pimmel^1^, Yohann Foucher^1,^ Maxime Leger^2^, Fanny Feuillet^3^, Laetitia Bodet-Contentin^4^, Raphaël Cinotti^5^, Denis Frasca^6^, Etienne Dantan^1^

##### ^1^INSERM UMR 1246 SPHERE, Nantes University, Tours University, Nantes, France; ^2^Medical Intensive Care, Angers University Hospital, Angers, France; ^3^INSERM UMR 1246 SPHERE, Nantes University, Tours University-Biostatistics and Methodology Unit, Nantes University Hospital, Nantes, France; ^4^Intensive Care Unit, Tours University Hospital, Tours, France; ^5^Anaesthesia and critical care department, Nantes University Hospital, Nantes, France; ^6^Anaesthesia and critical care department, Poitiers University Hospital, Poitiers, France

###### **Correspondence:** Jeanne Simon-Pimmel (jeanne.pimmel@gmail.com)

*Ann. Intensive Care* 2020, **10 (Suppl 1):**F-116

**Rationale:** In intensive care units, severe spontaneous hemorrhagic brain injuries have a poor prognosis for mortality and functional outcomes. Affected patients face particular ethical issues regarding the difficulty of anticipating their eventual recovery. In this context, prognostic scores can help clinicians in patients/relatives counseling and therapeutic decisions. The previous reviews pointed out many prognostic tools for intracranial hemorrhage and subarachnoid hemorrhage but did not focus on injuries explicitly severe nor assessed the methodological limitations of the models. Our systematic review aimed to assess methodologically prognostic tools for functional outcomes in severe spontaneous haemorrhagic brain, with particular attention to their clinical utilities.

**Patients and methods:** Following PRISMA recommendations, we queried Medline, Embase, Web of Science, and the Cochrane by February 19, 2019. We included multivariate prognostic models explicitly developed or validated on adults with severe intracranial or subarachnoid haemorrhage. We evaluated the articles following the CHARMS recommendations (CHecklist for critical Appraisal and data extraction for systematic Reviews of prediction Modelling Studies) and the TRIPOD statements (Transparent Reporting of a multivariable prediction model for Individual Prognosis.

**Results:** Our review confirmed the multiple publications of prognostic scores, as we found 71 articles aiming to develop or validate prognostic tools. Relying on guidelines, we discarded 52 articles due to the lack of prognostic capacities, validation, or predictor selection. 8 articles developed and validated a prognostic tool and 19 externally validated existing models (Fig. 1). No score was of good methodological quality in intracranial hemorrhage. We highlighted two prognostic scores in subarachnoid hemorrhages: the SAHIT predicting unfavorable outcome or mortality at 6 months and the FRESH predicting unfavorable outcome at 12 months.

**Conclusion:** Although prognostic studies on haemorrhagic brain injuries abound in the literature, they generally lack of methodological robustness or show incomplete reporting. With the numerous published scores, we believe that it is time to stop developing new scores. Ongoing validation, recalibration, and impact studies would keep improving existing good tools. The use of “patient-centered” approaches could also enhance them, and be more appropriate to inform patients and families about their long-term potential recovery. These considerations should drive future research in the modern era of neurocritical care prognosis.

**Compliance with ethics regulations**: NA.

**Fig. 1:**
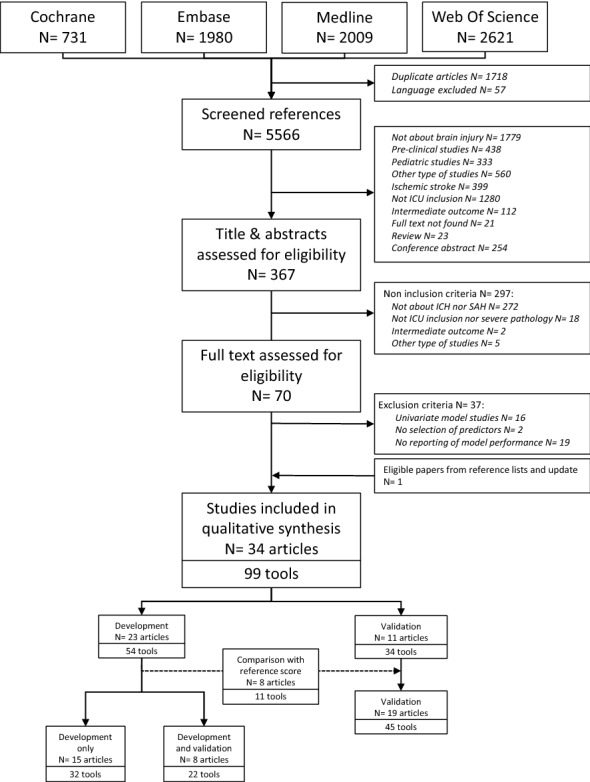
Flow chart of the systematic review

### F-117 RETRACT System: a computerized 3D video analyzing system for the monitoring of respiratory retraction signs in children

#### Haythem Rehouma^1^, Gabriel Masson^2^, Sandrine Essouri^2^, Rita Noumeir^3^, Philippe Jouvet^2^

##### ^1^École de Technologies Supérieure (ETS) of Montreal (QC) Canada, Montreal, Canada; ^2^CHU Sainte-Justine (CHUSJ), Mother and Child University Hospital Center, Montréal, Canada, Montreal, Canada; ^3^École de Technologies Supérieure (ETS) of Montreal (QC) Canada, Montreal, Canada

###### **Correspondence:** Haythem Rehouma (rhoumahaythem@gmail.com)

*Ann. Intensive Care* 2020, **10 (Suppl 1):**F-117

**Rationale:** Respiratory pattern analysis by a visual examination is an important part of clinical assessment but is dependent on caregiver expertise and is subjective. Furthermore, there is no easy medical device used in PICU to measure Tidal Volume (Vt) and Minute Ventilation (MV) in spontaneous breathing patients. The clinical research unit in critical care of CHUSJ and ETS have developed a non-invasive computerized 3D video analyzing system (RETRACT System) to detect and perform a video analysis of respiratory status in children. The aim of this study is to test the reliability of the RETRACT System to monitor respiratory distress in critically ill children.

**Patients and methods:** The RETRACT System is detailed in reference 1. In summary, cameras reproduce in 3D the thorax and abdomen of a subject. The respiratory status (respiratory rate (RR), tidal volume (Vt), minute ventilation (MV)) assessed by the RETRACT System was compared on a bench test (high-fidelity mannequin) and in critically ill children, to the ventilator measurements and clinician expert evaluation (gold standard). Bland–Altman plots were used for comparison.

**Results:** We observed a significant agreement, on mannequin, between RETRACT System and gold standard method in estimating Vt, RR and MV, i.e. 95% of the paired differences were within the limits of agreement in Bland–Altman plots, as illustrated in Fig. 1. In critically ill children (n = 2), the correlation between the pairs of measures was also high (r > 0.95, p < 0.001) and the coefficient of determination with a high fit (0.90 < R2 < 0.99, p < 0.0001). For good correlation, the RETRACT System needed to have a visual access to thorax and abdomen in a quiet subject.

**Conclusion:** The RETRACT System measurements of Vt, RR and MV for respiratory distress monitoring in patients seems reliable. More testing are required to validate this method in usual practice and to develop the retractions signs video analysis.

**Compliance with ethics regulations:** Yes.

**Fig. 1:**
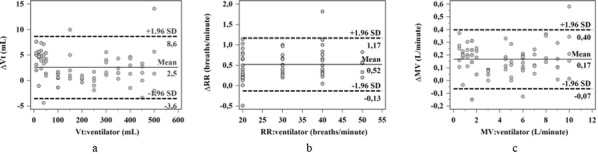
Bland-Altman plots of phantom’s quantitative measures. The plots are showing the agreement between the gold-standard method (ventilator) and our method (3D system) in estimating Vt (a), RR (b), MV (c) quantitative measures

**Reference**
1. Rehouma H, Noumeir R, Bouachir W, Jouvet P, Essouri S. Comput Med Imaging Graph 2018;70:17–28.

### F-118 Design and evaluation of a training support for pediatric tracheostomy care

#### Thibault Lassalle, Thomas Sagardoy, Astrid Botte

##### CHU Bordeaux, Bordeaux, France

###### **Correspondence:** Thibault Lassalle (thibaultlassalle40@gmail.com)

*Ann. Intensive Care* 2020, **10 (Suppl 1):**F-118

**Rationale:** Pediatric tracheostomy care of the child is unknown and feared by health professionals. The e-learning courses include the advantage of training a maximum of people over a minimum of time, and harmonize everyone’s knowledge. The objective of this study is to create and validate a training video for pediatric tracheostomy care, accessible by e-learning for health professionals.

**Patients and methods:** To design this training video, we followed the ADDIE 5-step educational engineering model: context and needs analysis, video design, video development, video implementation, evaluation of the effectiveness of the video. The evaluation of the video was carried out according to the first 3 phases of the Kirkpatrick model: first, learner satisfaction, second, learning evaluation (knowledge assessment and self-assessment of comfort before video) before video, immediately after video and at 3 months, third behavior modification. We have included health professionals performing tracheostomy care (pediatric nurses, childcare auxiliaries, physiotherapists, medical residents, senior doctors) in the main tracheostomy treatment centers in the New Aquitaine region who have agreed to participate to our study.

**Results:** 262 health professionals were included. 163 responded 3 months after the video. Learners were satisfied with the video. Their knowledge and skills were significantly improved (p < 0.001) (Fig. 1). Their improvement persisted at 3 months (p < 0.001). 93% of health professionals had modified their practice at 3 months.

**Conclusion:** This study made it possible to create and validate a training video for pediatric tracheostomy care which is accessible by e-learning for health professionals.

**Compliance with ethics regulations**: Yes.

**Fig. 1:**
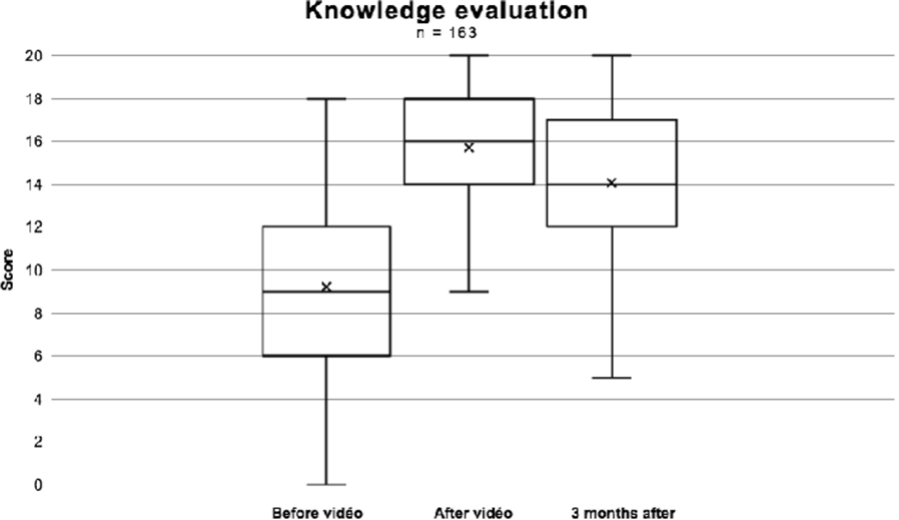
Knowledge evaluation

### F-119 Nurse-driven non-invasive ventilation weaning protocol in severe bronchiolitis in PICU is feasible and safe

#### Camille Guillot, Mathilde Periot-Jarry

##### Hopital Jeanne de Flandre-CHU Lille, Lille, France

###### **Correspondence:** Camille Guillot (cami.guillot@gmail.com)

*Ann. Intensive Care* 2020, **10 (Suppl 1):**F-119

**Rationale:** Severe bronchiolitis requires hospitalization in paediatric intensive care unit (PICU). Non-invasive ventilation (NIV) has been demonstrated to treat them since twenty years, its use is well defined but there is no consensus for the weaning. This study evaluated the application of a nurse-driven NIV weaning protocol in hospitalized infants with severe bronchiolitis and verified its safety.

**Patients and methods:** This was a retrospective monocentric study in a PICU of Robert Debré hospital-Paris, France. In the epidemic period of bronchiolitis between 2015 and 2017, all patients under one year old with severe bronchiolitis and requiring NIV were included. Two groups were compared: one group using the nurse-driven NIV weaning protocol and one group without using this protocol. Occurrences of complications, duration of ventilatory support and length of stay (LOS) in PICU and total LOS were compared.

**Results:** 191 patients were included in the study, 115 in the no-protocol group, and 72 in the protocol group. The nurse-driven protocol was using at the rate of 90% (n = 72/80). The use of protocol did not increase the occurrence of complications (n = 45/115, 37.8% in no-protocol group versus n = 22/72, 30.6% in protocol group p = 0.308). The ventilatory support duration and LOS did not increased by the protocol utilization. The ventilation support total duration was 41 h [19–74] in the no-protocol group versus 49 h [29–89] in the protocol group (p = 0.082), and the CPAP (Continuous Positive Airways Pressure) duration was 27 h [17–49] in the no-protocol group and 30 h [13–51] in the protocol group (p = 0.522). PICU LOS were 3.5 days [3–5] in the no-protocol group versus 4 days [3–5.5] in the protocol group (p = 0.383), hospital LOS was 10 days [7–14] in the no-protocol group versus 10 days [7–12] in the protocol group (p = 0.447) (Fig. 1).

**Conclusion:** The use of this first nurse-driven NIV weaning protocol was feasible and simple with a very good application rate. Its utilization was safe. The occurrence of complications did not increase by the use of this protocol. It would allow an optimal NIV weaning without prolonging the ventilatory support duration nor PICU LOS or hospital LOS. The professional practices appeared to be coordinated and the nurses appeared to be more autonomous.

**Compliance with ethics regulations**: Yes.

**Fig. 1:**
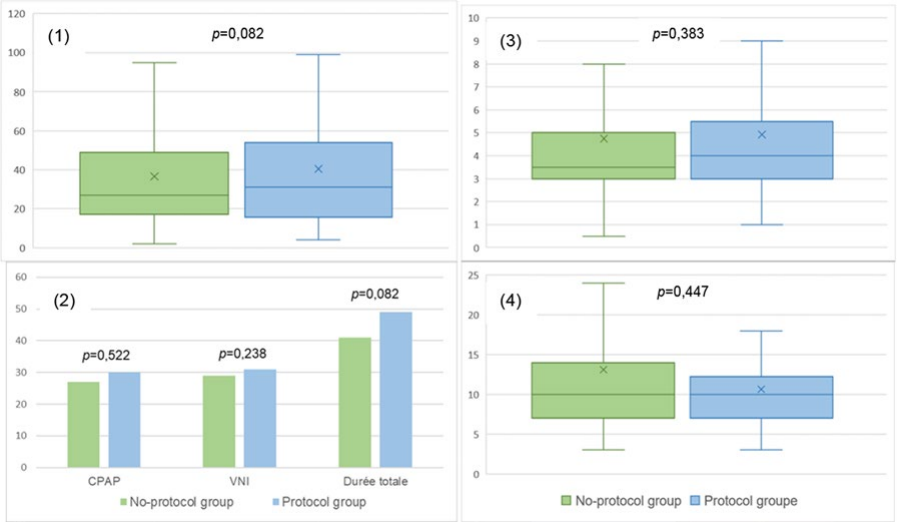
No-protocol and protocol groups comparison: CPAP duration (1), ventilatory support duration (2), PICU LOS (3), hospital LOS (4)

### F-120 Weaning from Non-Invasive Ventilation and High-Flow Nasal Cannula therapy in acute bronchiolitis in infants—A survey of practice in French-speaking countries

#### Marie Suzanne^1^, Isabelle Pin1, Guillaume Mortamet^2^

##### ^1^Service de pédiatrie, CHU de Grenoble, Grenoble, France; ^2^Service de réanimation pédiatrique, CHU de Grenoble, Grenoble, France

###### **Correspondence:** Marie Suzanne (msuzanne@chu-grenoble.Fr)

*Ann. Intensive Care* 2020, **10 (Suppl 1):**F-120

**Rationale:** First-line management of severe acute bronchiolitis in infants is mainly based on Non-Invasive Ventilation (NIV) and High-Flow Nasal Cannula (HFNC) therapy. However, pediatric data regarding weaning from NIV/HFNC are lacking. This study aims to identify the weaning practices from NIV/HFNC in children with severe bronchiolitis.

**Patients and methods:** The WeaNIV-survey is a cross-sectional survey. A questionnaire was sent to French-speaking physicians with key roles in pediatric intensive care units.

**Results:** A total of 73% (24/33) of French University Hospital were represented in the study. Only 9% of pediatric centers used a protocol for weaning from NIV/HFNC and nurses were considered as key-actors of the weaning process for half of participants. Continuous Positive Airway Pressure (CPAP) was the mode of ventilation mainly used as the first-line therapy in clinical practice. The main criteria considered to initiate weaning process were: no or slight respiratory distress, a FiO_2_ < 40%, a respiratory rate < 50/min and no significant apnea. Three strategies to discontinue NIV/HFNC were identified: 1/gradual decrease of ventilatory parameters (pressure or flow), 2/abrupt discontinuation and 3/gradual increase in off-ventilation time. Abrupt weaning strategy was the most commonly used, no matter the mode of ventilation. A significant level of respiratory distress, the presence of apneas, an increase in oxygen requirement, and a respiratory rate > 60/min were identified as weaning failure criteria by most pediatric intensive care physicians.

**Conclusion:** In most centers, the weaning process does not follow any protocol. Abrupt weaning seems to be commonly used as weaning strategy in children with severe bronchiolitis supported by NIV/HFNC. Based on the study findings, we suggest that criteria for weaning initiation and for weaning failure must be defined and weaning protocols generated.

**Compliance with ethics regulations**: Yes.

### F-121 Prone positioning for severe pediatric acute respiratory distress syndrome: an observational study in four French intensive care units

#### Laura Labro, Jerome Rambaud

##### Pediatric and neonatal intensive care unit, Armand-Trousseau hospital, Sorbonne university, Paris, France

###### **Correspondence:** Laura Labro (laura.glt@wanadoo.fr)

*Ann. Intensive Care* 2020, **10 (Suppl 1):**F-121

**Rationale:** Prone positioning is frequently used for management of adult Acute Respiratory Distress Syndrome (ARDS). However, no positive effects were proved in pediatric population. The first objective of our study was to evaluate the use and the efficacy of prone positioning in severe Pediatric-ARDS.

**Patients and methods:** We performed a retrospective descriptive multicenter study including all children aged from 28 days to 18 years old and suffering from severe ARDS in four referral paediatric intensive care units from January 2007 to December 2018. We evaluated the use of prone positioning, oxygenation parameters and ventilator settings at the beginning, 6 h and the end of positioning, survival and complications secondary to prone positioning.

**Results:** 93 patients were included indeed 59 (63%) benefited from prone positioning for a mean of 2.5 runs for 18.2 h. We found a decreasing of OSI (17.8 vs 10.8, p = 0.001), OI (26 vs 13, p = 0.0006) and an improvement of the PaO_2_/FiO_2_ ratio (75 vs 101, p = 0.002) throughout the first 6 h of prone positioning and between the beginning and the end of prone positioning (17.8 vs 6.7, p < 0.001), (26 vs 8, p = 0.007) and (75 vs 144, p = 0.007). Unfortunately, the survival rate was not significantly higher in the prone positioning group (80% vs 62%, p 0.07). Complications secondary to prone positioning occured for 6 patients (12.2%).

**Conclusion:** This first study, which evaluate prone positioning efficacy in severe P-ARDS shows evidence that prone positioning improves oxygenation parameters and survival rate. These results highlight the necessity to develop a multicentric prospective randomized study to confirm these conclusions.

**Compliance with ethics regulations:** Yes.

### F-122 Extracorporeal membrane oxygenation (ECMO) for immunocompromised children with acute respiratory distress syndrome (ARDS): a French ECMO center cohort

#### Jerome Rambaud, Blandine Robert

##### Pediatric and neonatal intensive care unit, Armand-Trousseau hospital, Sorbonne university, Paris, France

###### **Correspondence:** Jerome Rambaud (jerome.rambaud@aphp.fr)

*Ann. Intensive Care* 2020, **10 (Suppl 1):**F-122

**Rationale:** Immunocompromised children are likely to develop refractory acute respiratory distress syndrome (ARDS) and the usefulness of providing extracorporeal life support (ECLS) to these patients is a subject of continuing debate. The aim was to report outcomes and to compare factors associated with mortality between immunocompromised and non-immunocompromised children respiratory supported with ECMO.

**Patients and methods:** We performed a retrospective monocentric study in the pediatric French ECMO center of Armand Trousseau, including all pediatric patients aged from 1 month to 18 years requiring ECLS for ARDS.

**Results:** From 2007 to 2018, one hundred and eleven (111) patients were respiratory supported with ECMO; among them twenty-five (25) were immunocompromised. Survival rate 6 months after hospital intensive care discharge was significantly lower for immunocompromised patients compared to immunocompetent ones (41.7% vs. 62.8%; p = 0.04). ARDS severity was similar between groups. Fungal pneumonias were reported only for immunocompromised patients (12.5% versus 0% in the control group; p = 0.001). None of the clinical or biological data gathered prior to ECMO were different between the two groups. Bleeding complications were significantly more frequent in the immunocompromised group and blood product transfusions were also more frequently required in this group. There was no significant difference for the occurrence of nosocomial infections between groups.

**Conclusion:** Survival rate of immunocompromised children supported with ECMO for pediatric ARDS is lower than that of non-immunocompromised ones. But, the expectation for a favorable outcome is real and it is worth if the prognosis of their underlying disease is likely to be compatible with a good long-term quality of life.

**Compliance with ethics regulations**: Yes.

### F-123 Impact of establishment of a pediatric mobile ECMO team

#### Géraldine Poncelet^1^, Mathieu Genuini^1^, Jérôme Naudin^1^, Pierre-Louis Leger^2^, Guillaume Geslain^1^, Arielle Maroni^1^, Maryline Chomton^1^

##### ^1^Réanimation et surveillance continue pédiatriques, CHU Robert Debré, Paris, France; ^2^Réanimation et surveillance continue pédiatriques, CHU Armand Trousseau, Paris, France

###### **Correspondence:** Géraldine Poncelet (geraldine.poncelet@aphp.fr)

*Ann. Intensive Care* 2020, **10 (Suppl 1):**F-123

**Rationale:** Extracorporeal Membrane Oxygenation (ECMO) is used as a rescue therapy for critically ill children when usual respiratory and hemodynamic therapies fail to maintain vital functions. Since our hospital is not a pediatric ECMO center, children requiring this therapy need to be transferred to a referral ECMO center. Since November 2014, a pediatric mobile ECMO team composed of a medical transport team and a pediatric ECMO team is able to initiate ECMO in our Pediatric Intensive Care Unit (PICU). The aim of this study was to describe our population who required to be transported to a referral ECMO center.

**Patients and methods:** A retrospective observational study in a PICU, including all patients aged < 18 years old from 2014 to 2019 who required transfer to an ECMO center. Patient conditions, respiratory and hemodynamic parameters, time taken in charge by mobile ECMO team, immediately before cannulation were collected.

**Results:** Forty-three patients were included, median age was 21 months and 9 were newborns. Main indications for ECMO were Acute Respiratory Distress Syndrome (40%), refractory septic shock (23%), cardiac diseases (16%), Pertussis (9%), diaphragmatic hernia (5%) and cardiac arrest (2%). Survival at discharge was 53%. Number of organ failures (4 vs 2) and Vasoactive-Inotropic Score (VIS) (205 vs 50) were significantly higher in the non-survivor group. Cannulation was veno-venous (14%) or veno-arterial (67%) and 8 patients (19%) were finally not initiated on ECMO. We observed an increase of patients cannulated in our PICU over time (Fig. 1). There was no significant difference in mortality between patients transported on ECMO after cannulation in our PICU and those who were transported to be cannulated in a referral ECMO center. The median time between the decision and the cannulation was 3.25 h and the median time taken in charge by PICU transport team was approximately 1 h. These periods were not significantly different between cannulation on site or in an ECMO center and between survivors and not-survivors.

**Conclusion:** In our study, multiple organ dysfunction, particularly hematologic and acute renal failures, seems to be a risk factor of mortality. The delay between decision and management is similar whatever the cannulation site. Specific ECMO mobile team and PICU transport team seem to be essential, fast and trained to transfer these patients. It would be interesting to compare our cohort with children requiring ECMO already hospitalized in a referral ECMO center.

**Compliance with ethics regulations**: Yes.

**Fig. 1:**
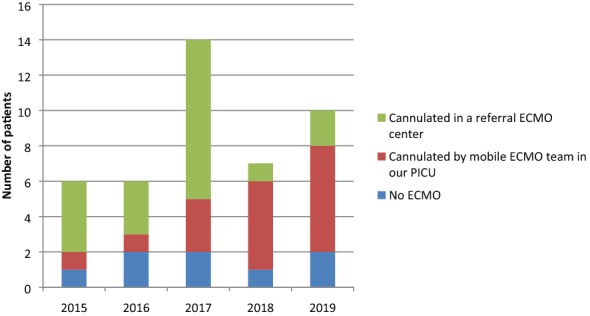
Number of patients transferred to an ECMO center over time

## E-posters

### P-001 The Use of ICU Resources in Metastatic Breast Cancer

#### Guillaume Beinse^1^, Jean-Jacques Tudesq^1^, Luis Teixeira^2^, Edith Borcoman^1^, Adrien Mirouse^1^, Yannick Hourmant^1^, Asma Mabrouki^1^, Michaël Darmon^1^, Virginie Lemiale^1^, Elie Azoulay^1^

##### ^1^Medical ICU, Saint-Louis Hospital, AP-HP, Paris, France; ^2^Breast diseases unit, Saint-Louis Hospital, AP-HP, Paris, France

###### **Correspondence:** Guillaume Beinse (guillaume.beinse@gmail.com)

*Ann. Intensive Care* 2020, **10 (Suppl 1):**P-001

**Rationale:** Life expectancy in patients with metastatic breast cancer (MBC) has substantially improved over the last decade. Life threatening complications result from advanced diseases, infection and treatment-related toxicity. Only few studies have assessed outcomes in this setting. We performed a hospital-wide study to investigate how ICU resources are needed in patients with MBC.

**Patients and methods:** All patients with MBC managed at our hospital between 2010 and 2019 were retrospectively included. The primary outcome was overall survival (OS). Factors associated with ICU mortality were identified using a multivariable Cox proportional hazard model with sensitivity analysis. Results are expressed as median [interquartile ranges] unless stated otherwise.

**Results:** Among the 1128 patients managed at our hospital, 68 (6%, including 1 male) were admitted to the ICU (8 [2–15] patients per year). Age was 55 [49–67] years. Patients were receiving their 2nd [1st–3rd] line of treatment and had 3 [2–3] metastatic sites. SOFA score at admission was 3 [1–8]. Main reason for ICU admission was sepsis (n = 23, 34%), acute respiratory failure (n = 22, 32%), coma (n = 9, 13%) and metabolic disorder (n = 7, 10%). Invasive mechanical ventilation was required for 18 patients (26%) and renal replacement therapy for 10 (15%). Sixteen (24%) patients died in ICU. Following ICU discharge, median OS was 6.4 months (95% CI [1.7–17.9]) and 22/52 (42.3%) patients died within 3 months. An antineoplastic treatment was resumed for 33/52 (62%) patients alive after ICU discharge. Factors independently associated with mortality were performance status ≥ 2 (HR 1.85, 95% IC [1.01–3.40]) and SOFA score at day 1 (HR 1.19 per point, 95% IC [1.11–1.27]). After sensitivity analysis, the number of treatment lines at ICU admission was not associated with mortality.

**Conclusion:** ICU admission is required in the course of the MBC disease for 6% of the patients. Determinants of short term outcomes rely on performance status and disease severity but not on the characteristics of the underlying disease. Ongoing analyses will assess whether ICU survivors reach life expectancy of patients never admitted to the ICU.

**Compliance with ethics regulations**: Yes.

### P-002 Factors associated with survival of patients with solid cancer alive after intensive care unit (ICU) discharge between 2005 and 2013

#### Hubert Gheerbrant^1^, Jean-François Timsit^2^, Nicolas Terzi^1^, Stephane Ruckly^3^, Mathieu Laramas^1^, Matteo Giaj Levra^1^, Emmanuelle Jacquet^1^, Loic Falque^1^, Denis Moro-Sibilot^1^, Anne-Claire Toffart^1^

##### ^1^CHU Grenoble Alpes, Grenoble, France; ^2^APHP, Paris, France; ^3^OutcomeRea, Bobigny, France

###### **Correspondence:** Hubert Gheerbrant (hgheerbrant@chu-grenoble.fr)

*Ann. Intensive Care* 2020, **10 (Suppl 1):**P-002

**Rationale:** The prognosis of critically ill cancer patients admitted in intensive care unit (ICU), remains an issue. Our objective was to assess the factors associated with 3- and 6-month survival of ICU cancer survivors.

**Patients and methods:** Based on the French OutcomeRea™ database, we included solid cancer patients discharged alive, between December 2005 and November 2013, from the medical ICU of the university hospital in Grenoble, France. Patient characteristics and outcome at 3 and 6 months following ICU discharge were extracted from available database.

**Results:** Of the 361 cancer patients with unscheduled admissions, 253 (70%) were discharged alive from ICU. The main primary cancer sites were digestive (31%) and thoracic (26%). The 3- and 6-month mortality rates were 33% and 41%, respectively. Factors independently associated with 6-month mortality included ECOG performance status (ECOG-PS) of 3–4 (OR, 3.71; 95% IC: 1.67–8.23), metastatic disease (OR, 2.24; 95% IC: 1.22–4.09), admission for cancer progression (OR, 2.64; 95% IC: 1.32–5.30), SAPS II of 45 to 58 (OR, 4.41; 95% IC: 1.85–10.53), and treatment limitation decision at ICU admission (OR, 3.89; 95% IC: 1.60–9.43). Interestingly, cancer chemotherapy prior to ICU admission was independently associated with lower 3-month mortality (OR, 0.38; 95% IC: 0.2–0.75). Among patients with an ECOG-PS 0–1 at admission, 70% (n = 66) and 61% (n = 57) displayed an ECOG-PS 0–2 at 3 and 6 months, respectively. At 3 months, 74 (55%) patients received anticancer treatment, 13 (8%) were given exclusive palliative care.

**Discussion:** Factors associated with 6-month mortality are almost the same as those known to be associated with ICU mortality. We highlighted that most patients recovered an ECOG-PS of 0–2 at 3 and 6 months, in particular those with a good ECOG-PS at ICU admission, and could benefit from an anticancer treatment following ICU discharge.

**Conclusion:** These results should be taken into account when deciding upon ICU admission. It is of paramount importance to have an evaluation of both patient’s general condition and anticancer treatment opportunities following ICU discharge.

**Compliance with ethics regulations**: Yes.

**Fig. 1:**
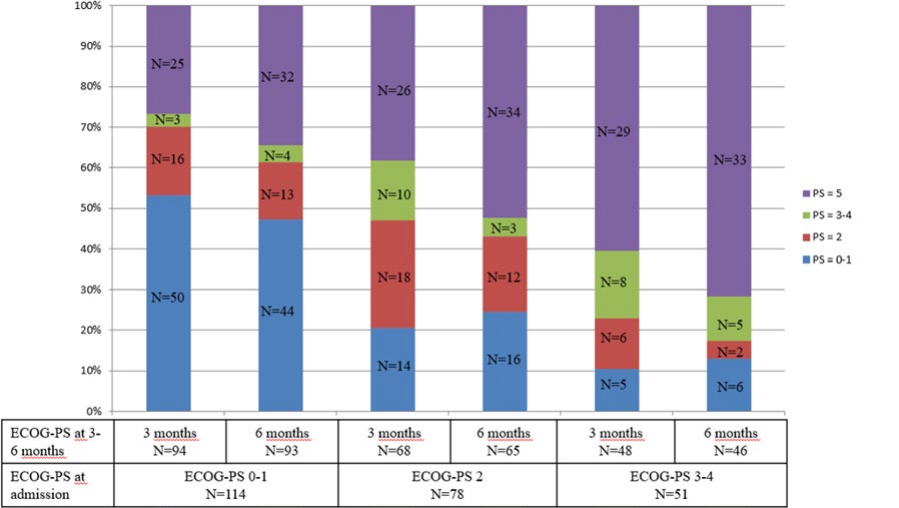
ECOG-PS at 3 and 6 months according to ECOG-PS at ICU admission

### P-003 Initiation of chemotherapy within ICU: a retrospective study

#### Maxence Chicoisneau, Lieveke Ameye, Marianne Paesmans, Jean-Paul Sculier, Anne-Pascale Meert

##### Institut Jules Bordet, Brussels, Belgium

###### **Correspondence:** Maxence Chicoisneau (Maxence.Chicoisneau@ulb.ac.be)

*Ann. Intensive Care* 2020, **10 (Suppl 1):**P-003

**Rationale:** The decision to urgently initiate medical anti-cancer treatment in cancer patients admitted to intensive care unit for cancer-related organ failure is an issue. We currently lack criteria to select patients who may benefit from the treatment initiation. The purpose of our exploratory study was therefore to evaluate the characteristics of patients whose medical anti-cancer treatment is initiated in ICU and to identify prognostic factors for in-hospital mortality. in these patients.

**Patients and methods:** We analyzed retrospectively, over a period of 11 years (1/1/2007 to 31/12/2017), cancer patients over 18-year old admitted to our ICU Bordet and in whose anti-cancer medical treatment was initiated during in-ICU stay. To identify prognostic factors for in-hospital mortality, we carried out a multivariate analysis of the factors influencing this mortality, considered as a binary. We also analyzed the long term survival of patients alive after their hospital stay (from the day of going out of hospital).

**Results:** Overall, 147 patients were included, 78 men (53%) and 69 women (47%), with a median age of 58 years (18–86). Of these, 79 patients (54%) had a solid tumor and 68 (46%) had a hematological tumor. In-ICU mortality is 23% (95% CI 17–31%) and in-hospital mortality 32% (95% CI 25–40%). The prognostic factors for in-hospital mortality were age (mean 61 vs 54 in those who survived), the SOFA score (median 6 vs 2), the SAPS II score (mean 41 vs 31), the Charlson score (mean 8 vs. 6.5), the number of organ failure (mean 2.5 vs 1.4) and the presence of a therapeutic limitation (NTBR stated within 24 h: 68% vs 16%). Survival at 1 year of patients who survived the hospital stay was 37% and median survival time was estimated to be 0.5 year (95% CI 0.4–0.9). In patients with a solid tumor, 1-year survival was 17% and 61% in those with a hematological tumor (p < 0.001).

**Conclusion:** We observed, in selected cancer patients admitted to the ICU for a cancer-related complication, that the initiation of an anti-cancer medical treatment is feasible and can lead to interesting results, particularly in patients with a hematological tumor.

**Compliance with ethics regulations**: Yes.

### P-004 Adequacy and prognosis of ICU admission or denial in critically ill onco-hematology patients

#### Wafa Zarrougui^1^, Emna Ennouri^1^, Tarek Ben Ahmed^2^, Khaoula Meddeb^1^, Radhouane Toumi^1^, Amal Baccari^1^, Imen Ben Saida^1^, Mohamed Boussarsar^1^

##### ^1^Medical Intensive Care Unit, Farhat Hached University Hospital. Université de Sousse, Faculté de médecine de Sousse, LR No LR12SP09. Heart Failure, Sousse, Tunisia; ^2^Department of medical oncology, Farhat Hached University Hospital, Sousse, Tunisia

###### **Correspondence:** Wafa Zarrougui (wafa.zarrougui91@gmail.com)

*Ann. Intensive Care* 2020, **10 (Suppl 1):**P-004

**Rationale:** Considerable progress in the management of onco-hematology (OH) malignancies led to an increase in the number of patients proposed for Intensive Care Unit (ICU) admission. Several guidelines offer decision models for ICU transfer of these patients. We aimed to describe prognosis, adequacy of ICU admission and denial in onco-hematology patients.

**Patients and methods:** We included all OH patients proposed for ICU admission in a Tunisian medical ICU, between January 2016 and July 2019. From an admission proposal registry, were collected patient underlying condition, functional status, malignancy and predicted prognosis, acute critical illness and its reversibility, adequacy of ICU admission approval or denial according to the 2016 AFSOS guidelines, and patients outcomes.

**Results:** During the study period, 173 patients were proposed for ICU admission, only 8 (4.6%) were admitted. They were 96 (55%) male; 44.6 ± 15.6 mean aged; 3.6 ± 2.3, mean Charlson Index; 49 (28.3%) had World Health Organisation (WHO) score ≥ 3. Underlying Malignancy were; 23 (13.3%) non metastatic cancer; 43 (24.9%), metastatic cancer; 103 (59.5%), haematological malignancy. Predicted prognosis was estimated at under 3 months for 68 (39.3%) of patients. Acute critical illness were mainly acute circulatory failure, 66 (38.2%) and acute respiratory failure, 52 (30.1%) with 99 (57.2%) considered reversible. Mortality was 7 (87.5%) for admitted and 83 (50.3%) for denied patients. Decision of ICU admission was adequate with guidelines in 5 (62.5%) and 139 (84.2%) in denials.

**Conclusion:** Physicians’ clinical judgement regarding ICU admission of OH patients seems satisfying. However, a subset of patients could benefit from ICU but are denied admission due to ICU beds unavailability and misuse.

**Compliance with ethics regulations**: Yes.

### P-005 Characteristics and Outcomes of Critically Ill Cancer Patients admitted to a Tunisian Intensive Care Unit

#### Imen Ben Saida^1^, Hela Kallal^1^, Yosri Ben Ali^1^, Nesrine Fraj^1^, Wafa Zarrougui^1^, Imtinene Belaid^2^, Mohamed Boussarsar^1^

##### ^1^Medical intensive care unit, Farhat Hached hospital, Université de Sousse, Faculté de Médecine de Sousse, UR No LR12SP09. Heart Failure, Sousse, Tunisia; ^2^Oncology department, Farhat Hached hospital, Université de Sousse, Faculté de Médecine de Sousse, Sousse, Tunisia

###### **Correspondence:** Imen Ben Saida (imen.bensaida@yahoo.com)

*Ann. Intensive Care* 2020, **10 (Suppl 1):**P-005

**Rationale:** Cancer patients frequently need intensive care support for a life-threatening condition due to the underlying neoplasm or an adverse therapy-related event. However, there are poor data on their characteristics and outcomes in the intensive care setting. The aim of the present study was to describe clinical characteristics and to identify factors associated with in-ICU mortality in critically ill cancer patients.

**Patients and methods:** It is a retrospective study conducted in the medical ICU of Farhat Hached teaching hospital between January 2007 and December 2018. All cancer patients with complete records were included. Baseline characteristics, clinical parameters, severity of illness, primary tumor location and outcomes were collected. Univariate and multivariate regression analyses were carried out to identify factors independently associated to poor prognosis.

**Results:** During the study period, 3569 patients were admitted, 59 (1.65%) had malignancy. Among these, 27 (45.8%) had hematological malignancies and 32 (54.2%) had solid tumors, of whom 15 (25.4%) had evidence of metastases. Clinical characteristics were: mean age, 55.3 ± 14.7 years; male, 38 (64.4%); WHO Performance status 0 to 1, 55 (93.2%); median SAPSII, 53 [41–67]; invasive mechanical ventilation (IMV), 45 (76.6%); median duration of IMV, 2 [1–5]days; vasopressors use, 43 (72.9%). The main reasons for admission were: septic shock, 21 (35.6%); coma, 11 (16.9%); pneumonia 9 (15.3%); pulmonary edema, 8 (13.6%) and miscellaneous 11 (18.6%). Median length of ICU stay was 3 [2–8] days and mortality rate was 67.8%. On univariate analysis, the factors associated with mortality were, IMV on admission (26.3% vs 80%; p < 0.001); vasopressors use (26.3% vs 95%;p < 0.001) and septic shock (45% vs 15.8%; p = 0.029). Multivariate regression model identified two factors as independently associated to mortality: IMV on admission (OR, 9.4; 95% IC, [1.6–55.1]; p = 0.013) and vasopressors use (OR, 46.5; 95% IC, [6.5–330.3]; p < 0.001).

**Conclusion:** In the present study, invasive mechanical ventilation and vasopressors use on ICU admission were the independent predictive factors of mortality in critically ill cancer patients.

**Compliance with ethics regulations**: Yes.

### P-006 Prognosis and mortality risk factors of denied onco-hematology patients for ICU admission

#### Emna Ennouri^1^, Wafa Zarrougui^1^, Tarek Ben Ahmed^2^, Khaoula Meddeb^1^, Radhouane Toumi^1^, Amal Triki^1^, Imen Ben Saida^1^, Mohamed Boussarsar^1^

##### ^1^Medical Intensive Care Unit, Farhat Hached University Hospital. Université de Sousse, Faculté de médecine de Sousse, LR No LR12SP09. Heart Failure, Sousse, Tunisia; ^2^Department of medical oncology, Farhat Hached University Hospital., Sousse, Tunisia

###### **Correspondence:** Emna Ennouri (m.na.ennouri@gmail.com)

*Ann. Intensive Care* 2020, **10 (Suppl 1):**P-006

**Rationale:** Thanks to therapeutic advances, prognosis of patients with malignancies significantly improved during the last decades. However, occurrence of life-threatening condition could be a turning point in those patients evolution, especially when ICU facilities are not available.

We aimed to determine prognosis and risk factors of mortality of denied onco-hematology patients requiring ICU admission.

**Patients and methods:** A retrospective study was conducted in a Tunisian medical ICU between January 2016 and July 2019. Were included all consecutive denied onco-hematology patients proposed for ICU admission. Patients baseline characteristics, underlying malignancy and 3 months prognosis, severity of illness and its reversibility and outcome were collected. Univariate and multivariate regression analysis were used to identify factors independently associated with mortality.

**Results:** During the study period, 165 onco-hematology patients were denied to ICU admission out of 173 proposed patients. Mean age, 45.1 ± 15.6 years; sex ratio 1; mean Charlson Index, 3.6 ± 2.4; median [IQR] World Health Organisation (WHO) score, 2 [0–2]. Underlying malignancies were: non metastatic cancer, 21 (12.5%); metastatic cancer, 42 (25.5%) and haematological malignancies, 98 (59.4%). Mortality rate was 83 (50.3%). Univariate analysis yielded the following as factors associated with mortality: 3-month malignancy prognosis (p = 0.000), predicted reversibility of critical illness (p = 0.000), WHO score ≥ 2 (p = 0.005), acute neurological impairment (p = 0.006) and pulmonary embolism (p = 0.008). Multivariate regression model identified 3 factors to be independently associated with mortality: reversibility of critical illness (OR, 7.4; 95% IC, [2.5–22.9]; p = 0.000); WHO score ≥ 2 (OR, 0.44; 95% IC, [0.19–0.99]; p = 0.048) and acute neurological impairment (OR, 0.3; 95% IC, [0.1–0.9]; p = 0.035).

**Conclusion:** The present study among onco-hematological patients denied for ICU admission, identified, underlying condition, neurological impairment and predicted reversibility of critical illness, as factors associated with short term in-hospital mortality.

**Compliance with ethics regulations**: Yes.

### P-007 Influence of underlying hematological malignancy grade on outcome of critically ill hematological patients

#### Pablo Pagliarani, Vincent Rebiere, Lara Zafrani, Virginie Lemiale, Djamel Mokart, Alexandre Demoule, Martine Nyunga, Achille Kouatchet, Frédéric Pene, Michaël Darmon, Elie Azoulay

##### APHP, Paris, France

###### **Correspondence:** Pablo Pagliarani (pablopagliarani@gmail.com)

*Ann. Intensive Care* 2020, **10 (Suppl 1):**P-007

**Rationale:** Prognostic impact of underlying malignancy seems limited in most studies assessing outcome of critically ill cancer patients [1]. However, only limited number of characteristics, namely disease progression status and preexisting stem cell transplantation, were usually assessed [1]. Primary objective of this study was to assess influence of hematological malignancy aggressiveness on hospital outcome. Secondary objective was to assess influence hematological malignancy aggressiveness on type of infection.

**Patients and methods:** Post-hoc analysis of prospective multicenter cohort performed in 17 hospitals in France and Belgium and including critically ill adults with underlying hematological malignancy admitted in ICU from Jan 2010 to May 2011. A Cox model was used to adjust for confounding variables then a propensity score matching on characteristics associated with underlying malignancy aggressiveness was performed.

**Results:** Of the 1011 included patients, 300 (29.7%) had low grade malignancy (LG), the most frequent being myeloma (n = 126), chronic lymphocytic leukemia (n = 76), and myelodysplasia (n = 46). Patients with LG malignancy were older, underwent more frequently autologous stem cell transplantation (SCT) and had less frequently altered performans status. They had more severe organ failure at ICU admission (SOFA score 6 [4–9] vs. 5 [3–8], P = 0.03). Before adjustment, mortality was 36% (n = 108) and 39.9% (n = 284) respectively in patients with and without LG malignancy (P = 0.27). After adjustment for confounder using a Cox model, a higher mortality was associated with non-low grade malignancy (OR 1.49; 95% IC 1.17–1.9). A propensity score then allowed a 1:1 matching upon variable associated with malignancy aggressiveness. After matching unadjusted mortality was 36% (n = 108) in patients with LG malignancy and 48.8% (n = 146) in patients with high grade malignancy (P = 0.002) (Figure). In the matched cohort and after adjustment for confounder, high grade malignancies were associated with lower mortality (OR 1.42; 95% IC 1.10–1.84). Risk of fungal infection was unchanged by underlying malignancy before adjustment (6% vs. 8.6% of patients with and without LG malignancy; P = 0.26) or after adjustment (HR 1.29; 95% IC 0.71–2.41).

**Conclusion:** Despite anti-cancer advances, aggressiveness of hematological malignancies is associated with overall ICU outcome. Low-grade malignancies displaying a better prognosis than non-low grade. Aggressiveness of the underlying malignancy is not associated with risk of fungal infection.

**Compliance with ethics regulations**: Yes.

**Fig. 1:**
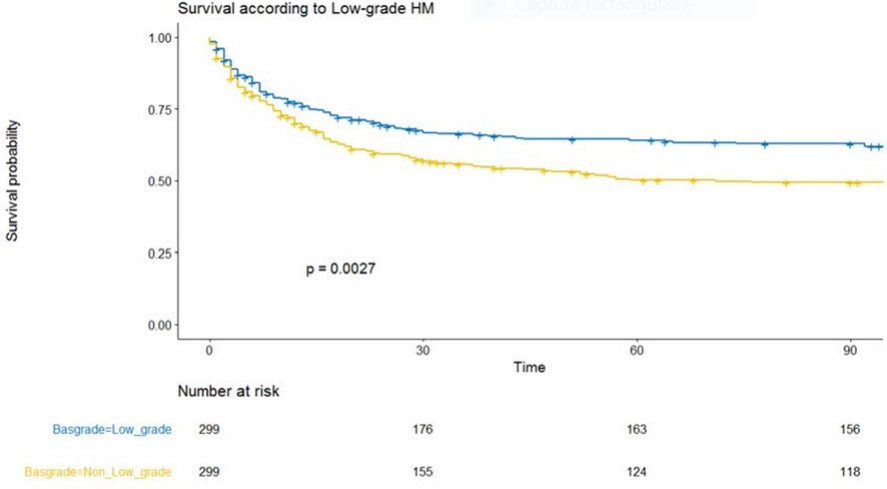
Survival probability according to underlying malignancy after propensity score matching

### P-008 Pulmonary embolism in ICU of the CHU of ORAN: retrospective study about 22 cases

#### Soulef Bousbia, Soumia Benbernou, Nabil Ghomari, Houria Djebli

##### Faculté de medecine d’Oran, Oran, ALGERIA

###### **Correspondence:** Soulef Bousbia (bsoulef90@gmail.com)

*Ann. Intensive Care* 2020, **10 (Suppl 1):**P-008

**Rationale:** The pulmonary embolism (PE) remains a serious condition that requires intensive care unit (ICU) admission.

**Patients and methods:** We aimed to determine the clinical, therapeutic and features scalable of patients with PE within the Department of resuscitation of the CHU of ORAN. It is a retrospective study including patients admitted to the CHU of ORAN from January 2014 to December 2017 with PE. Several parameters have been collected including age, gender, reason for admission, symptomatology, PE risk factors, the Wells score, therapy and evolution.

**Results:** We collected 22 patients suffering of EP among 1038 patients admitted to the ICU. The median age was 64 years, 75% of the patients were female, the main symptoms were dyspnea in more than 50% of the patients, onset of symptomatology was brutal in 87.5%. Physical signs were dominated by tachycardia in all patients, blood gases highlighted hypoxia in 60% of the patients. D. dimeres plasma levels were increased in all patients. Angioscanner found proximal thrombus in 75% of patients. All the patients received curative dose HBPM and finally mortality was around 50%.

**Conclusion:** Tachycardia and polypnea are the main clinical characteristics of patients with PE in the emergency ward. Beside tools for PE diagnosis and risk stratification may be helpful in patient management.

**Compliance with ethics regulations**: Yes.

### P-009 Pulmonary embolism feature in patients with Guilliain Barre syndrom

#### Olfa Turki, Rezk Gorbel, Mariem Dlela, Najeh Baccouche, Abir Bouattour, Mabrouk Bahloul, Mounir Bouaziz

##### HABIB BOURGUIBA University Hospital, Sfax, Tunisia

###### **Correspondence:** Olfa Turki (olfa.turki.rea@gmail.com)

*Ann. Intensive Care* 2020, **10 (Suppl 1):**P-009

**Rationale:** Guillain-Barré syndrome is the most common cause of acute flaccid paralysis and is associated with pulmonary embolism due to the mobility limitation. The aim of this study is to describe the incidence, the severity of pulmonory embolism in patients admitted to an intensive care unit (ICU) for Guillain–Barre syndrome (GBS).

**Patients and methods:** Twenty-eight adults patients with confirmed diagnosis of GBS were admitted to the ICU in our university hospital center over a 10-year period and they were all included. Prevalence, risk factors and course of VTE were analyzed in ICU patients with various forms and severity of GBS.

**Results:** During the study period, 23 adult GBS patients were included. Five (17.9%) developped pulmonary embolism. The mean age was 51.2 ± 16.7 years and the sex ratio was 0.86. The comparaison betewen the 2 groups with and without PE showed that factors associated with the development of this complication were: respiratory failure requiring mecanical ventilation (p = 0.03), infectious complications (p < 0.001), blood pressure lability (p = 0.029), the delay of ICU admission (p = 0.02), the delay to treatment initiation (p = 0.036), the SOFA score (p = 0.03) and the presence of quadriplegia (p = 0.031).

**Conclusion:** Pulmonary embolism is a frequent complication in patients with GBS. Factors associated with this complication were: respiratory failure requiring mecanical ventilation, infectious complications, the delay of ICU admission, the delay to treatment initiation, a high SOFA score and the presence of quadriplegia. Preventive measures in this category of patients have to be improved.

**Compliance with ethics regulations:** Yes.

### P-010 Acute respiratory distress syndrome among burns in Tunisia: state of play and prognosis

#### Wael Chemli, Lilya Debbiche, Hana Fredj, Sarra Ben Zarrouk, Hana Benali, Manel Ben Saad, Amel Mokline, Amenallah Messaadi

##### Burns intensive care unit, CTGB, Ben Arous, Tunisia

###### **Correspondence:** Wael Chemli (wchemli90@gmail.com)

*Ann. Intensive Care* 2020, **10 (Suppl 1):**P-010

**Rationale:** Acute respiratory distress syndrome (ARDS) is a life-threatening pathology associated with very high morbidity and mortality (35–45%) in intensive care units (ICU) and with even higher mortality among the severly burned patients worldwide (36 à 80%). The aim of our study was to describe in Tunisia burn patients with ARDS and to identify prognosis factors.

**Patients and methods:** We conducted a descriptive retrospective study between 01-01-2017 to 31-12-2018, in burns ICU, in Ben Arous, in Tunisia. All burns who presented an ARDS, according to the Berlin 2012 definition, during their stay in the ICU, were included. When clinical or gasometric data was uncomplete, these patients were excluded.

**Results:** During the study period, 691 patients were admitted to our burn unit including 246 ventilated patients. Fifty patients presented an ARDS: fifteen patients were excluded for lack of information, and 35 patients were retained. The sex ratio was 2.5. Patients had a mean age of 36 ± 12 years, an average burned area of 44% ± 22%, an average unit of burn skin score (UBS score) of 94 ± 77 and an average sequential organ failure assessment score (SOFA score) of 4. None of the patients had a history of cardiovascular or pulmonary diseases. The average time of onset of ARDS was 5 ± 4 days. ARDS was mild in 1 case, moderate in 11 and severe in 23. The etiology of ARDS was pulmonary in 25 cases (71%) and extra-pulmonary in 10 (29%). The pulmonary ARDS had as cause pneumonia isolated in 15 patients, an isolated pulmonary burn in 6 patients and a combination of pneumonia and lung burns in 4 patients. Extra-pulmonary ARDS were all due to sepsis and mainly to bacteremia. Septic shock was associated with ARDS in 20 patients (57%). The treatment was a conventional treatment based on protective ventilation, curarization and prone positioning in addition to the etiological treatment. The average length of stay in ICU was 9 days and mortality was 85% in these patients.

**Conclusion:** Mortality from ARDS in burns in Tunisia, is important especially in those with pulmonary burns as well as those with sepsis. The introduction of new treatments, such as extracorporeal membrane oxygenation, remains essential to improve the prognosis of burn patients.

**Compliance with ethics regulations**: Yes.

### P-011 Risk factors and outcomes of aspiration pneumonia

#### Oussama Jaoued, Makni Saba, Abid Emna, Sik Ali Habiba, Fekih Hassen Mohamed, Elatrous Souheil

##### Hôpital Taher sfar, Mahdia, Tunisia

###### **Correspondence:** Oussama Jaoued (oussamajaoued@gmail.com)

*Ann. Intensive Care* 2020, **10 (Suppl 1):**P-011

**Rationale:** Aspiration pneumonia (AP) is common in intensive care unit (ICU). The incidence of AP among adults hospitalized with pneumonia ranges between 5 and 53.2%. Usually one or more risk factors are identified to be involved in AP. The aim of this study was to determine the risk factors and predictors of mortality on patients with AP.

**Patients and methods:** We retrospectively included patients aged more than 18 years and who were hospitalized in our ICU for AP. Patients were excluded if they had history of tuberculosis, if they have bronchiectasis or metastatic brain tumor.

**Results:** A total of 102 patients were included. History of diabetes, hypertension, epilepsy and ischemic stroke were found respectively in 22.2%, 21.5%, 16.7%, and 7.8% of cases. The reason of ICU admission were coma (35%), acute respiratory failure (33%), poisoning (27%) and cardiac arrest (5%). The incidence of acute respiratory distress syndrome (ARDS) was 17%. The most common organism isolated was staphylococcus aureus (4 cases). Risk factors for AP were epilepsy (20%), swallowing disorders (18%), ischemic stroke (12%), COPD (9%) and degenerative neurological disease (5%). The mortality rate was 17.6%. The median duration of mechanical ventilation was 12 days [IQR 10–23]. In multivariate logistic regression analysis; SAPS II score (OR = 1.05, 95% IC [1.001–1.1], p = 0.046) and ARDS (OR = 44.04, 95% IC [3.91–495.57], p = 0.002) were independently associated with mortality.

**Conclusion:** Risk factors for aspiration pneumonia were epilepsy, swallowing disorders and ischemic stroke. ARDS and SAPS II score were independent predictive factors of mortality.

**Compliance with ethics regulations:** Yes.

### P-012 Hyperoxia in intensive care: impact on morbidity and mortality

#### Rim Jemmeli, Samia Ayed, Amira Jamoussi, Dhouha Lakhdhar, Jalila Ben Khelil, Mohamed Besbes

##### Abderrahmen Mami Hospital, Ariana, Tunisia

###### **Correspondence:** Rim Jemmeli (rimjemmeli@gmail.com)

*Ann. Intensive Care* 2020, **10 (Suppl 1):**P-012

**Rationale:** Oxygen therapy is a common treatment in intensive care unit (ICU). While its efficiency in hypoxia is well-known, several studies have shown a harmful effect of oxygen when prescribed at high doses. The threshold value defining pathological hyperoxia remains undetermined. The aim of this study was to evaluate the impact of hyperoxia on morbidity and mortality.

**Patients and methods:** This was a prospective study performed in the ICU of Abderrahmen Mami Hospital during a 4-month period. All patients admitted in ICU during the study-period were included. Those who didn’t need oxygen therapy or in end of life stage were excluded. Arterial blood gases were analyzed daily and each day with at least one value of oxygen arterial saturation (SaO2) > 92% was considered as a day with hyperoxia. For each patient included, the number of times and days spent in hyperoxia was recorded as well as complications during the ICU stay and the outcome.

**Results:** During the study-period, 140 patients were included but only 112 were eligible. Mean age was 58 ± 18 years. Acute on chronic respiratory failure was the most frequent reason of admission (67%). Non-invasive ventilation was required for 18% of patients and invasive mechanical ventilation was necessary in 65% of cases. Overall mortality was 32%. Hyperoxia was observed in 96% of cases, with an average of 10 ± 10 times during the ICU stay and 6 ± 6 days. A statistically significant association was observed between a long duration of hyperoxia and the occurrence of ventilator acquired pneumonia (p < 10–3), ventilator acquired bronchitis (p = 0.001), acute respiratory distress syndrome (p < 10–3), atelectasis (p < 10–3), septic shock (p < 10–3), rythm disorders (p = 0.003), reintubation (p < 10–3) and tracheostomy (p = 0.038). On multivariate analysis, independent factors of mortality were: simplified acute physiology score II, cardiac failure, need for invasive mechanical ventilation and septic shock. Hyperoxia was not independently associated with mortality.

**Conclusion:** Hyperoxia is frequent in ICU. It is significantly associated with ICU complications but not independently associated with mortality.

**Compliance with ethics regulations:** Yes.

### P-013 Experience of the practice of prone position in patients with acute respiratory distress syndrome in intensive care (CHU Oran)

#### Nabil Ghomari, Soumia Benbernou, Djebli Houria

##### Faculté de medecine d’Oran, Oran, Algeria

###### **Correspondence:** Nabil Ghomari (nabilghomari@hotmail.fr)

*Ann. Intensive Care* 2020, **10 (Suppl 1):**P-013

**Rationale:** Mechanical ventilation (MV) in the prone position (PP) and low tidal volume have become recommendations with a high level of scientific evidence in recent years. The PP has been practiced for 7 years in the CHU Oran emergency resuscitation service. We wanted to report the service experience in the practice of PP in patients with ARDS.

**Patients and methods:** Retrospective study performed in patients with severe hypoxia ARDS with SPO2 < 88% under FIO2 > 80% or PAO2/FIO2 < 150 during the period March 2011 to December 2018.

**Results:** 38 patients received ventilation in PP. ARDS was secondary to thoracic trauma in 42% of patients, septic shock in 32% and aspiration pneumonitis in 26%. Analysis of the success factors and improvement of oxygenation found that lobar ARDS, the delay < 72 h and a duration of PP ≥ 18 h were statistically significant.

**Conclusion:** The PP must be integrated into the arsenal of care of the patients in ARDS especially in our country where we do not have all the therapeutic options.

**Compliance with ethics regulations:** Yes.

### P-014 Impact of Cannula Size on Clinical Outcomes in Venovenous Extracorporeal Membrane Oxygenation

#### Julien Goutay, Nicolas Cousin, Thibault Duburcq, Erika Parmentier-Decrucq

##### CHU de Lille, Pôle de Réanimation, Hôpital Salengro, Lille, France

###### **Correspondence:** Julien Goutay (julien.goutay@gmail.com)

*Ann. Intensive Care* 2020, **10 (Suppl 1):**P-014

**Rationale:** In Veno-Venous Extracorporeal Membrane Oxygenation (VV-ECMO) therapy, blood flow is the main determinant of arterial oxygenation and should be 60–80 mL/kg/min in adults. This flow rate is determined by several factors including the size of the inflow cannula. The impact on clinical outcomes of arterial cannula’s size in Veno-Arterial ECMO (VA-ECMO) has already been studied, and showed no difference for survival to discharge, weaning success rate and initial flow rate between a small cannula group and a larger one. Our first objective was to describe the impact of inlet cannula size on the assistance flow rate in patients treated with VV-ECMO. Secondary objectives were to analyze its impact on ECMO weaning, mechanical ventilation characteristics and mortality.

**Patients and methods:** We retrospectively reviewed all cases of respiratory failure treated with VV-ECMO admitted in the medical intensive care unit (ICU) of Lille’s teaching hospital from January 1st, 2013 through March 31st, 2019. Inlet cannula size was collected and divided into two groups: the “small cannula” group had inlet cannula less than or equal to 23Fr, while “large cannula” were larger than 23Fr. Primary endpoint was the initial flow rate according to the inlet cannula size, and its changes during the first 48 h of assistance. Secondary endpoints were the analysis of predictive factors associated with the choice of a larger inlet cannula, and the impact of its size on clinical outcomes such as successful ECMO weaning.

**Results:** 74 patients treated with VV-ECMO were admitted in our hospital. Eleven (15%) were cannulated with a large inlet device. Mean initial ECMO flow rate was statistically higher in the “large cannula” group than in the “small cannula” one: 5.8 L/min (± 0.7) versus 4.7 (± 0.8) respectively, p < 0.0001. The difference was also significant during the first 48 h of assistance. We found no difference between the two groups on clinical outcomes such as ECMO weaning time. In univariate analysis, weight was heavier in the “large cannula” group [94 (± 26) kg] than “small cannula” [81 (± 20)], p < 0.05.

**Conclusion:** ECMO initial flow rate was higher in a “large inlet cannula” group (internal diameter more than 23 Fr) compared with a “small cannula” group. We found no correlation with cannula-related haemorrhagic or thrombotic complications. Inlet cannula size did not influence ECMO weaning, and duration time, but this may be a lack of statistical power. Further prospective studies should confirm this results.

**Compliance with ethics regulations**: Yes.

**Fig. 1:**
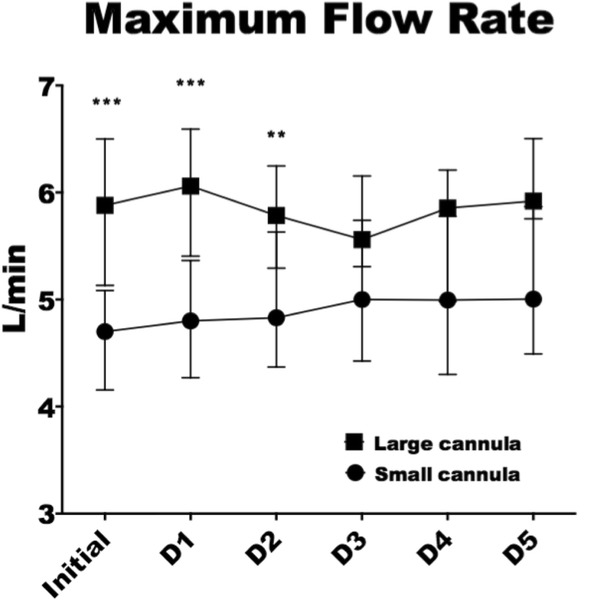
Difference and evolution of maximum ECMO flow rate in patients with small (n = 60) and large inlet cannula (n = 10)

### P-015 Colonization with carbapenemase-producing Gram-negative bacilli in burn patients in Tunisia

#### Sonia Ben Behi^1^, Sarra Dhraief^1^, Hana Fredj^2^, Lilya Debbiche^2^, Amenallah Messaadi^2^, Lamia Thabet^1^

##### ^1^Centre de Traumatologie et des Grands Brûlés de Ben Arous-Laboratoire de biologie médicale et banque du sang, Ben Arous, Tunisia; ^2^Centre de Traumatologie et des Grands Brûlés de Ben Arous-Service de réanimation des brûlés, Ben Arous, Tunisia

###### **Correspondence:** Sonia Ben Behi (benbehi.sonya@gmail.com)

*Ann. Intensive Care* 2020, **10 (Suppl 1):**P-015

**Rationale:** Burn patients are at risk of multidrug-resistant (MDR) bacterial infections with high mortality rate. Therefore, monitoring the emergence of MDR pathogens in these vulnerable patients is important. This study aimed to assess digestive colonization with carbapenemase-producing Gram-negative bacilli (CP-GNB) in patients admitted to the burn intensive care unit.

**Patients and methods:** Our study was prospective and conducted over a one-year period (January 2018 to December 2018). Every admitted patient was subjected to the screening. A double swab set was used to collect rectal swab specimens. One swab was used for MDR screening by disk diffusion method on selective media; the other for multiplex real-time PCR (Cepheid’s GeneXpert^®^) allowing detection of the most common carbapenemase-encoding genes (CEG) (blaOXA-48, blaKPC, blaNDM, blaVIM and blaIMP).

**Results:** Among the 125 studied patients, 71 (56.8%) were detected positive at admission for CP-GNB by the GeneXpert^®^ Carba-R assay. Eleven patients, initially not colonized, acquired positive faecal carriage subsequently during their hospital stay. Forty-two colonized patients (59.1%) developed CP-GNB infection during their hospitalization. The CEG blaNDM quantitatively dominated by far with 54 detections; either alone (15 cases) or associated with other CEG (39 cases). The second most frequent gene was blaOXA-48. It was detected alone eight times and in association with other CEG 38 times. Forty-three patients carried blaVIM gene, usually in association with other CEG (93%). However, only one patient carried blaKPC gene. The parallel screening by classical microbiology methods (disk diffusion on selective media) detected the presence of CP-GNB in all molecular positive samples.

**Conclusion:** Our study describes the characterization of carbapenemase in burn patients and highlights their alarming spread. This emphasizes the importance of an active surveillance program by early detection of CP-GNB carriers and an isolation policy to limit the MDR infections expansion.

**Compliance with ethics regulations**: Yes.

### P-016 Evaluation of Antifungal Therapy in Burn ICU

#### Selma Abid, Hana Fredj, Amel Mokline, Bahija Gasri, Manel Ben Saad, Amenallah Messaadi

##### Intensive Burn Care Departement, Burn and Trauma Center-Tunis, Tunisia, Ben Arous, Tunisia

###### **Correspondence:** Selma Abid (darly2910@gmail.com)

*Ann. Intensive Care* 2020, **10 (Suppl 1):**P-016

**Rationale:** Invasive fungal infections are increasingly observed in the ICUs especially in burn units. In the absence of simple and accessible techniques for early microbiological diagnosis, the use of antifungal treatment is increasing. Little is known about the extent of the problem of antifungal prescription in burn ICUs. We aimed to evaluate the antifungal prescription in major burn patients.

**Patients and methods:** During the study period (2018–2019), all prescriptions of antifungals were analysed. Analysis concerned demographics, clinical circumstances, as well as the basis of antifungal prescribing (targeted vs. empiric).

**Results:** Among the 691 patients admitted in this period, 58 patients were treated with antifungals (sex ratio: 1.4; mean age: 38 ± 19 years, with low associated comorbidity). The TBSA was 34.25% [19.5–53.25], UBS was 57 [32.75–91]. Most of the patients (87.9%) were transferred from another hospital structure within 62 ± 96 h. Antifungal treatment was started at the average of the seventh day post wound injury, more often on an empiric basis. SOFA score at the beginning of the treatment was 11 ± 3.9. Lymphopenia was present in 32% and thrombopenia was present in 41%. Index of colonisation was positif only in 4 cases. The average candida score was 2.72 ± 1.15. Microbiological results were tardily collected, within 3 weeks, in 27%. Fungal urine infections were found in 9 cases. Candidemia and catheter-related infections were considered only in 2 cases. The risk factors of fungal infection as described in literature were found in most of the patients including mechanical ventilation (84.4%), length period of stay (16 days [8.75–29.5]), central venous line (100%), severe sepsis or septic shock (100%), large-spectrum antibiotherapy for more than 3 days (100%).

**Conclusion:** The management of antifungal infections in major burn patients is still challenging. Antifungal prescription is based on clinical presumption. The empirical prescription reflects the lack of efficient laboratory support and late microbiological results prompting physicians to rely on clinical informations. The management of fungal infections is based on the improvement of mycological investigations.

**Compliance with ethics regulations**: NA.

### P-017 Epidemiology of candidemia in a medical ICU: a 12-years retrospective study

#### Salma Klaii^1^, Ahlem Trifi^2^, Aicha Kallel^1^, Foued Daly^2^, Yosr Touil^2^, Sami Abdellatif^2^, Kalthoum Kallel^1^, Salah Ben Lakhal^2^

##### ^1^Parasitology/Mycology laboratory, la Rabta hospital, Faculty of Medicine of Tunis, Tunis, Tunisia; ^2^Medical ICU, la Rabta hospital, Faculty of Medicine of Tunis, Tunis, Tunisia

###### **Correspondence:** Salma Klaii (kalai.salma89@gmail.com)

*Ann. Intensive Care* 2020, **10 (Suppl 1):**P-017

**Rationale:** Invasive candidiasis is a widespread and alarming infection in intensive care units (ICU) patients. Its diagnosis is often difficult because of the lack of specificity of clinical signs and the low sensitivity of blood cultures. While the Candida albicans species remain the most common cause of bloodstream infections, non-albicans are emerging. These infections are serious, associated with high mortality rate and requiring early diagnosis and appropriate treatment. In Tunisia, few data are available. We aimed to determine the epidemiological profile of a series of candidemia in ICU, the risk factors associated with the occurrence of candidemia and to describe the modalities of the mycological diagnosis of candidemia and their etiological profile.

**Patients and methods:** A retrospective longitudinal descriptive study conducted in the Parasitology—Mycology laboratory with the collaboration of the medical ICU of la Rabta hospital—Tunis over a 12-year period from January 1, 2006 to December 31, 2017. All hospitalized ICU patients with at least one Candida-positive blood culture were included.

**Results:** Forty-three patients among 2585 hospitalized patients during the study period had at least one candidemia infection. The main risk factors for development of candidemia infection include invasive procedures, a prior use of antibiotics and parenteral nutrition. C. Albicans was the most common species, detected in 41.9% of patients. Non-albicans Candida species were prominent (58.1%), represented by C Parapsilosis, followed by C. Tropicalis and C. Krusei then C. Glabrata and finally C. Lusitaniae. All the isolates tested were sensitive to the common antifungal agents. The mortality rate of our patients was high (76.7%), and the detection of the Albicans species in blood cultures was the only prognostic factor identified (OR = 9.38 [0.83–105.23], p = 0.06).

**Conclusion:** Candidemia in the medical ICU patients is common and is associated with high mortality rate. Despite the progress of biological tools, the diagnosis is difficult and needs to take into account the risk factors of the patients as well as scores based on clinical and microbiological parameters. A better identification of risk patients may help to early initiate empirical antifungal treatment.

**Compliance with ethics regulations**: Yes.

### P-018 Necrotizing soft-tissue infections in the intensive care unit: a retrospective hospital-based study

#### Kais Regaieg, Sabrine Nakaa, Arnaud Mailloux, Madjid Boukari, Johana Cohen, Dany Goldgran-Toledano

##### Groupe hospitalier intercommunal Le Raincy-Montfermeil, Montfermeil, France

###### **Correspondence:** Kais Regaieg (kais.regaieg@gmail.com)

*Ann. Intensive Care* 2020, **10 (Suppl 1):**P-018

**Rationale:** The objective of our study is to describe the epidemiological and clinical characteristics of Necrotizing soft-tissue infections (NSTI) and to improve therapeutic management.

**Patients and methods:** We conducted a retrospective observational study that included patients admitted in the intensive care unit (ICU) of general hospital between September 2014 and Aout 2019 with a primary or secondary diagnosis of NSTI. We collected demographic and clinical data, cultured pathogens, lengths of stay, and in-ICU mortality.

**Results:** During the study period, a total of 20 patients admitted to the ICU were diagnosed with NSTI (0.3% of the total number of patients). The mean of age was 69 years. The sex ratio (M/W) was 0.6. Ten patients (50%) were directly admitted to the ICU, others were transferred from medical or surgical wards. The mean of SAPS II was 61.6 (26.6). The main indication to admission in ICU was shock (50%). The most common comorbidity was diabetes (50%). The other co-morbidities associated with NSTI were cardiovascular diseases (33%), obesity (25%) and carelessness (25%). The sites most commonly affected were extremities in 15 patients (75%) and abdomen/ano-genital in 4 patients (20%). In ICU, a total of 17 patients (85%) were mechanically ventilated [ (median duration: 4.70 days (8.63)], 15 patients (75%) were given vasopressors, and 4 patients (20%) underwent renal-remplacement. All patients underwent one or more chirurgical intervention. 17 patients (85%) underwent radical necrosectomy. In 3 cases, an amputation was necessary. Polymicrobian infection was seen in 10 patients (50%). In 8 patients (40%), we used vacuum assited closure therapy, which in 3 patients was followed by definitive reconstruction by split skin grafts. The mortality in ICU was 50%. The mean stay in ICU was 12 days (2–73). The mean duration of hospitalization of the patients who survived was 75 days (15–180). On the basis of a univariate analysis, higher SAPS II score and lactate levels were associated with increased mortality (p < 0.01).

**Conclusion:** NTSI is rare in ICU but it’s a life-threatening and disabling disease with a high mortality requiring a multidisciplinary management. Early diagnosis and adequate treatment are necessary to improve clinical outcome and must be known by everyone. More studies are needed to estimate the interest and delay of new strategies such as negative pressure therapy.

**Compliance with ethics regulations:** Yes.

### P-019 Bacteriological profile and antimicrobial susceptibility of isolated bacteria in a burn intesive care unit in Tunisia

#### Karim Mechri^1^, Sarra Dhraief^1^, Emna Hammami^1^, Amel Mokline^2^, Kawther Faleh^2^, Amenallah Messaadi^2^, Lamia Thabet^1^

##### ^1^Centre de Traumatologie et des Grands Brûlés de Ben Arous-Laboratoire de biologie médicale et banque du sang, Ben Arous, Tunisia; ^2^Centre de Traumatologie et des Grands Brûlés de Ben Arous-Service de réanimation des brûlés, Ben Arous, Tunisia

###### **Correspondence:** Karim Mechri (karimmechri91@gmail.com)

*Ann. Intensive Care* 2020, **10 (Suppl 1):**P-019

**Rationale:** Nosocomial infections remain a major cause of mortality and morbidity in burn patients. Providing information about the main causative bacterial agents and determination of their susceptibility to antibiotics may improve empiric therapy and early detection of emerging antimicrobial resistance. The aim of our study was to investigate the species distribution and antibiotic susceptibility of isolated strains from a burn intensive care unit (ICU).

**Patients and methods:** This study was performed retrospectively on all bacteriological samples taken from the burn ICU at the trauma and burn center in Tunisia during a seven year period (from January 2012 to December 2018). All isolated microorganisms were identified on the basis of standard microbiological techniques. Antibiotic susceptibility testing was carried out by the agar disk diffusion method, and susceptibility results were interpreted using clinical breakpoints according to CA-SFM guidelines. Minimum inhibitory concentration of Colistin was determined using the E-Test^®^ method (BioMérieux), then using the EUCAST broth micro-dilution method (UMIC, Biocentric^®^) since May 2017.

**Results:** During the study period, the most frequent identified species were Pseudomonas Aeruginosa (15.7%), Staphylococcus Aureus (15%), Klebsiella Pneumoniae (12.6%) and Acinetobacter Baumannii (12%). These strains have been mainly isolated from blood cultures (37%) and skin samples (26.6%). Pseudomonas Aeruginosa resistance to Ceftazidime increased from 9.2% in 2012 to 53.5% in 2018 and resistance to Imipenem and Ciprofloxacin was 63.3% and 42.9%, respectively. Four strains were resistant to Colistin. The rate of Methicillin resistant Staphylococcus Aureus (MRSA) decreased from 65.3% in 2012 to 41.6% in 2018. Resistance rate to Gentamicin and Ciprofloxacin was 52% and 50.8%, respectively. All strains were susceptible to glycopeptides, Tigecyline and Linezolid. Most of A. Baumannii strains were multidrug resistant with 81.8% of resistance to Ceftazidime, 88.9% to Amikacin, 90.5% to Ciprofloxacin and 94.5% to Imipenem. Sixteen strains were resistant to Colistin. Concerning K. Pneumoniae, 77.5% of the strains were resistant to Cefotaxime and 5.2% to Imipenem. Two strains were resistant to Colistin. Vancomycin resistance in Enterococcus Faecium increased from 33.4% in 2012 to 72.2% in 2018.

**Conclusion:** Multidrug resistance in burn patients imposes an epidemiological surveillance of microbial ecology and better application of hygienic measures.

**Compliance with ethics regulations:** Yes.

### P-020 Prognostic factors of community-acquired peritonitis

#### Khalid Khaleq, Amine Zerhouni, Driss Elhammoudi, Rachid El Harrar

##### CHU Ibn Rochd, Casablanca, Morocco

###### **Correspondence:** Khalid Khaleq (khaleq20@gmx.fr)

*Ann. Intensive Care* 2020, **10 (Suppl 1):**P-020

**Rationale:** Community-acquired peritonitis is a heterogeneous condition characterized by peritoneum inflammation in response to a bacteria injury. The aim of our study is to describe the epidemiological, clinical, bacteriological, etiological, therapeutic characteristics of community peritonitis, and to evaluate the prognostic factors.

**Patients and methods:** This is a retrospective descriptive and analytical study spanning three years (Between January 2015 and December 2017) involving 114 cases of community peritonitis, hospitalized in the surgical emergency resuscitation department P33 Ibn Rochd Casablanca University Hospital. Our study included adult patients with community-acquired peritonitis who underwent medical and surgical management. The studied parameters are the demographic data, the clinical and paraclinical signs, the care taken and the evolution of the patients.

**Results:** The study showed that the mean age was 54.2 ± 17.2 years, with a sex ratio of 2.17. Patients medical history included tobacco (31.60%), extra-abdominal signs [hemodynamic failure (45%), renal failure (n = 39, 50%), hematological disorders (n = 80, 70%) and respiratory disorders (n = 38, 60%)]. Therapeutic management was based on perioperative resuscitation, treatment of organ failure, probabilistic antibiotic therapy and median laparotomy surgery. The main etiologies of community peritonitis were: digestive perforation (76.3%), purulent effusion (50%), intestinal necrosis (16.7%), cholecystitis (3.5%). Intraoperative bacteriological specimens yielded the following bacteriological profile: predominance of NGB (85.9%) dominated by E. Coli (46.8%) followed by Klebsiella Pneumoniae and Enterobacter Cloacae (28.2%) The mean hospital stay was 5.4 ± 5.5 days. The mortality rate was 55.3%.

**Conclusion:** Improvement in the prognosis of community-acquired peritonitis can only be achieved by constant assessment of very early diagnosis and initiation of appropriate resuscitation and antibiotic therapy associated with a complete surgery carefully codified according to guidelines.

**Compliance with ethics regulations**: Yes.

### P-021 Bloodstream infections caused by KPC-producing bacteria

#### Sabrine Bradai, Kamilia Chtara, Basma Mnif, Karama Bouchaala, Mabrouk Bahloul, Mounir Bouaziz

##### University of Sfax, Sfax, Tunisia

###### **Correspondence:** Sabrine Bradai (sabrine.bradai2@gmail.com)

*Ann. Intensive Care* 2020, **10 (Suppl 1):**P-021

**Rationale:** Klebsiella Pneumoniae carbapenemase (KPC)-producing bacteria are a group of emerging highly drug-resistant Gram-negative bacilli causing infections associated with significant morbidity and mortality. The aim of our study is to point out the incidence of bloodstream infections (BSI) caused by KPC in ICU patients, its clinical presentation and course.

**Patients and methods:** We conducted a retrospective descriptive study. All patients hospitalized in the ICU of our hospital who developed BSI caused by KPC from January 01, 2016 to December 31, 2017 were included.

**Results:** During the study period, 20 patients were included. The mean age was 45.7 ± 22.5 years ranging from 8 to 91 years. Sex ratio (M/F) was 3. Trauma was the major cause of hospitalization in 12 cases (60%). The most common past medical diseases were arterial hypertension in 7 patients (35%). Length of hospital stay prior to ICU admission was 4 ± 7.2 days. At infection onset, mean SAPS II was 49 ± 13.2, mean SOFA was 10.4 ± 4.8 and mean APACHE II was 19.8 ± 6.7. During ICU hospitalization, all patients required invasive mechanical ventilation during 27.2 ± 13.2 days, had a central venous catheter (CVC) and an indwelling urinary catheter in place, 15 patients (63.9%) had tracheotomy, 12 (60%) underwent surgery, 9 (45%) presented acute kidney failure and 4 (20%) needed hemodialysis. Before the isolation of KPC, all patients presented infections. Antibiotics prescript were: Colistin in 14 patients (70%), Carbapenems in 12 patients (60%), Amoxicillin/clavulanic acid in 11 patients (55%), Cephalosporins in 9 patients (45%), Fluoroquinolones in 6 patients (30%), Tigecycline in 5 patients (25%), Aminosids in 4 patients (20%), Rifampicin in 4 patients (20%), Fosfomycin in 3 patients (15%), Glycopeptides in 2 patients (10%). The delay for KPC-BSI onset was 17.7 ± 7.5 days. The most common infection sources responsible of KPC-BSI were: CVC in 6 patients (30%) and pneumonia in 5 patients (25%). KPC infection was responsible of septic shock in 18 patients (50%). Resistance rates were: Gentamycin (65%), Amikacin (20%), Colistin (15%), Fosfomycin (10%) and Tigecycline (10%). Antibiotics used to treat KPC bloodstream infection were resumed in Table 1. The mean length of ICU stay was 36.1 ± 16.8 days. Out of the 20 included patients, 7 patients died (The mortality rate was 35%). Death was related to KPC infection in 5 patients.

**Conclusion:** The high prevalence of KPC-BSI in ICU patients dictates the importance of implementation of infection control measures and strict antibiotic policies.

**Compliance with ethics regulations**: Not applicable.

**Table 1:** antibiotics used to treat KPC bloodstream infections

Empiric antibiotic	**Number**	**Adapted antibiotic**	**Number**
Cefotaxime Tigecycline	1 1	Amikacin Fosfomycin Tigecycline	1 1 1
Imipenem + colistin Imipenem + amikacin Amoxicillin/clavulanic acid +amikacin Ciprofloxacin + colistin Tigecycline + colistin Tigecycline + ciprofloxacin Fosfomycin + amikacin Piperacillin/tazobactam + ciprofloxacin Piperacillin/tazobactam + colistin	4 2 1 1 1 1 1 1 1	Imipenem + colistin Tigecyline + colistin Bactrim + amikacin Ciprofloxacin + tygecycline Tygecycline + colistin Fosfomycin + amikacin Piperacillin/tazobactam + ciprofloxacin Piperacillin/tazobacta + coli Piperacillin/tazobactam + fosfomycin Imipenem + tygecycline	4 2 1 1 1 1 1 1 1 1
Imipenem + colistin + ciprofloxacin Imipenem + colistin +fosfo Imipenem + colistin + tigecycline Imipenem + colistin + vancomycin Tigecycline + rifampicin + ciprofloxacin	1 1 1 1 1	Imipenem + Bactrim + colistin Piperacillin/tazobactam + amikacin + colistin	1 1
No use of empiric antibitic	0	KPC not treated	0

### P-022 Incidence of healthcare-associated infections in a Tunisian intensive care unit

#### Rania Ammar Zayani, Farah Zouari, Yousfi Mounir, Mariem Dlela, Chokri Ben Hamida, Mounir Bouaziz

##### Faculty of medecine, University of Sfax, Sfax, Tunisia

###### **Correspondence:** Rania Ammar Zayani (rania.ammarzayani@gmail.com)

*Ann. Intensive Care* 2020, **10 (Suppl 1):**P-022

**Rationale:** Healthcare-associated infections are the most frequent adverse event in healthcare delivery worldwide. It causes significant increase in morbidity, mortality and financial burden on the healthcare system. Our objective was to determine the incidence and clinical aspect of healthcare-associated infections.

**Patients and methods:** A longitudinal study of incidence including patients who had exceeded 48 h in the intensive care unit at Habib Bourguiba teaching hospital (Sfax Tunisia) over a period of 3 month (01/02/2019 to 31/05/2019).

**Results:** We included 136 patients whose mean age was 48.3 ± 20 years (4–90). Sex ratio was 2.31. The average SAPS II at admission was 33.5 points ± 16.22. The average SOFA score at admission was 6.89 ± 3.98 points. The median of ICU lengths of stay was 9 days (2–80). We identified 103 episodes of nosocomial infections in 56 patients, representing a cumulative incidence rate of 41.17 per 100 exposed patients. The incidence density was 30.43 infections per 1000 days of hospitalization. The prevalence of pneumonia was 38.83%, followed by urinary tract infections 21.35%, central venous catheterization infections 15.53%, bacteriemia16.3%, meningitis 6.7% and surgical site infections 1.9%. The incidence rate of intubation-related pneumonia was 24.47/1000 day of exposure. The incidence rate of bladder-related urinary tract infection was 9.7/1000 day of exposure. The incidence rate of positive culture of the central venous catheter was 7.05/1000 day of exposure. The incidence rate of bacteremia related to stay was 9.2/1000 day of exposure. The mortality rate was 26.5% with a significant difference between infected and uninfected patients (p = 0.041). Microorganisms were gram negative bacteria in 80% of cases.

**Conclusion:** Epidemiological surveillance of healthcare-associated infections is needed to establish prevention plans.

**Compliance with ethics regulations**: Not applicable.

### P-023 Prognostic factors of digestive oncological emergencies in the elderly

#### Khalid Khaleq, Karima Mourabit, Fatimazahra Bensardi, Rachid Al Harrar, Aziz Fadil

##### Faculté de médecine et de pharmacie Université Hassan II/CHU Ibnou Rochd, Casablanca, Morocco

###### **Correspondence:** Khalid Khaleq (khaleq20@gmx.fr)

*Ann. Intensive Care* 2020, **10 (Suppl 1):**P-023

**Rationale:** The purpose of this study was to assess the prognosis factors of care improvement and management of elderly before, during and after surgery, the predictors of mortality and morbidity following emergency oncological digestive surgery in patients aged 65 years and older.

**Patients and methods:** Overall, 86 patients admitted to visceral emergencies for an urgent syndrome revealing or complicating a primary or secondary digestive cancer, and who required immediate surgical intervention and who had stayed at the surgical resuscitation department in our hospital on a duration of 4 years. Several data were recorded and analyzed using the SPSS version 20 software:

- Epidemiological, concerning age and gender.
- Clinical parameters including risk factors, history, general condition of the patient and physical examination.
- Paraclinical parameters, interesting biological data and imaging.
- Medical and surgical therapeutics.
- Postoperative follow-up.
- Treatment results.

**Results:** The most frequent sites were rated in order of increasing frequency: Colo-rectal (40.7%), small intestine (22.1%), pancreas (10%), and biliary tract (8.1%). 72% of patients were between 65 and 75 years and, while only 27.9% were 76 years old and over. This study included 44 women and 42 men with a sex ratio of 0.96. The evolution method was mostly acute in 95% of cases. Patients had consulted for urgent clinical presentations mainly occlusive syndrome in 59% of cases. Abdominal CT scan was performed in 71%, followed by abdominal Xray in 31% and ultrasonography. The therapeutic management was medical and surgical. The surgery done in 62% for palliative indication: 55% were operated for an ostomy discharge, 32% for a palliative resection, 17% for an Ostomy Supply and 13% for a digestive bypass. Post-operative outcomes were 35% morbidity and 48% mortality. The main cause of death was hemodynamic instability in 34% of cases. In multivariate statistical analysis, four factors were significantly associated with mortality: Morbidity: p < 0.001; OR = 17.20; IC: [4.16; 71.13], The CONUT score: p < 0.001; OR = 2.85; IC:[1.34; 6.03], hypoalbuminemia: p = 0.030; OR = 2.35; IC: [1.63; 8.75] and admission for a Bowel obstruction: p < 0.001; OR = 2.65; IC: [1.68; 10.36].

**Conclusion:** Small changes in morbidity and mortality could have a significant impact, both on the results, on the high cost of prolonged hospitalization, and on the incidence of perioperative complications. This will require a good knowledge of the predictive mortality factors both by the anesthetist and by the visceralist surgeon.

**Compliance with ethics regulations**: NA.

### P-024 Usefulness of shock index for prehospital triage of septic shock by the SAMU regulation

#### Romain Jouffroy^1^, Anastasia Saade^1^, Pascal Philippe^1^, Papa Gueye^2^, Emmanuel Bloch-Laine^3^, Patrick Ecollan^4^, Benoit Vivien^1^

##### ^1^Department of Anesthesia & Intensive Care Unit, SAMU, Hôpital Necker-Enfants Malades, Paris, France; ^2^Prehospital Medical System, SAMU de Martinique, University Hospital Pierre Zobda Quitman, Martinique, Fort-De-France, France; ^3^Emergency department, hospital Cochin 24 rue du Faubourg Saint-Jacques 75014 Paris France and emergency department—SMUR, Hospital Hôtel-Dieu, Paris, France; ^4^Intensive Care Unit, SMUR, Pitie Salpetrière Hospital, Paris, France

###### **Correspondence:** Romain Jouffroy (romain.jouffroy@gmail.com)

*Ann. Intensive Care* 2020, **10 (Suppl 1):**P-024

**Rationale:** Scoring systems were developed for risk-stratification of septic shock (SS) patients but their performance is poor in the prehospital setting. The aim of this study was to evaluate the ability of the shock index (SI) in prehospital triage of SS patients to predict their admission in intensive care unit (ICU).

**Patients and methods:** We performed a 2-month retrospective study of call records received by the Paris SAMU 75 regulation center concerning patients with presumed SS. The outcome was the in-ICU admission. Results are expressed by Odd Ratio (OR) with 95 percent confidence interval [95 CI].

**Results:** Among the 30 642 calls received, 140 concerned patients with presumed SS were included. Twenty-two patients (16%) were admitted to ICU and 118 (84%) to the emergency department. The AUC of the SI was 0.76 [0.65–0.86]. Using a threshold for SI > 0.9, the sensitivity was 82%, the specificity was 67%, the positive predictive value was 32% and the negative predictive value was 95%. After logistic regression analysis, the OR for SI > 0.9 reached 7.65 [2.33–35.00]. Using propensity score analysis, the OR for SI > 0.9 was 1.34 [1.15–1.52].

**Conclusion:** SI is a reliable tool for risk stratification of SS patients managed in the prehospital setting. Using a threshold of 1 for the SI may be helpful to screen patients requiring ICU admission by the SAMU 15 regulation call center. Prospective studies including SI in the decision-making process in the prehospital triage of SS patients are needed to validate these results.

**Compliance with ethics regulations**: Yes.

### P-025 Clinical impact of Atrial Fibrillation in patients hospitalized for diabetic ketoacidosis at Emergency Department

#### Nadia Zaouak, Yosra Yahya, Khedija Zaouche, Abdelwahab Mghirbi, Radhia Boubaker, Hamida Maghraoui, Kamel Majed

##### Emergency Department of La Rabta teaching hospital, Tunis, Tunisia

###### **Correspondence:** Nadia Zaouak (zaouak.nadia@yahoo.fr)

*Ann. Intensive Care* 2020, **10 (Suppl 1):**P-025

**Rationale:** Atrial fibrillation (AF) is a common heart arrhythmia. Many studies have shown that AF led to increased in-hospital complications. The main objective of this study was to determinate the clinical impact of AF in patients with diabetic ketoacidosis.

**Patients and methods:** This is a retrospective observational study performed in all patients aged more than 18 years hospitalized to Emergency Department (ED) for diabetic ketoacidosis on a period of 6 months. Data of all patients were collected. The statistical analysis compared 2 groups of patients based on the AF presence or not at initial electrocardiogram. The main endpoints of the study were in-hospital mortality and need of critical care defined by use of vasoactive drugs and mechanical ventilation. Secondary endpoints was the length of stay.

**Results:** The study included 56 patients admitted for diabetic ketoacidosis. The mean age was 55 ± 20 years with a sex ratio of 1.2. 86% had not AF and 14% had AF at initial electrocardiogram. The rate of mortality in the AF group was 28.6%. 20% of them required mechanical ventilation and 40% of vasoactive drugs. In the no AF group, the in-hospital mortality was 8.9%. 10.3% of them needed vasoactive drugs and 11% mechanical ventilation. Duration of stay was higher in AF group: 47 ± 32 h versus 36 ± 42 h (p < 0.05). There was no significant correlation betwen mortality, need of drugs and mechanical ventilation (p = 0.56; p = 0.34; p = 0.13, respectively).

**Conclusion:** Our results showed that AF was not significantly correlated with in-hospital mortality and critical care in patients admitted at ED for diabetic ketoacidosis. However, AF led to a significant increased length of stay.

**Compliance with ethics regulations:** Yes.

### P-026 Prehospital shock index to assess mortality of septic shock

#### Romain Jouffroy^1^, Jean-Pierre Tourtier^2^, Papa Gueye^3^, Emmanuel Bloch-Laine^4^, Vincent Bounes^5^, Guillaume Debaty^6^, Josiane Boularan^7^, Benoit Vivien^1^

##### ^1^Department of Anesthesia & Intensive Care Unit, SAMU, Hôpital Necker-Enfants Malades, Paris, France; ^2^Paris Fire Brigade, Teaching military hospital Bégin, Paris, France; ^3^Prehospital Medical System, SAMU de Martinique, University Hospital Pierre Zobda Quitman, Martinique, Fort-De-France, France; ^4^Emergency department, hospital Cochin 24 rue du Faubourg Saint-Jacques 75014 Paris France and emergency department—SMUR, Hospital Hôtel-Dieu, Paris, France; ^5^SAMU 31, Toulouse University Hospital, Toulouse, France; ^6^SAMU 38, Grenoble University Hospital, Grenoble, France; ^7^SAMU 31, Castres Hospital, Castres, France

###### **Correspondence:** Romain Jouffroy (romain.jouffroy@gmail.com)

*Ann. Intensive Care* 2020, **10 (Suppl 1):**P-026

**Rationale:** In the prehospital setting, early identification of septic shock (SS) with high risk of mortality is essential to guide hospital orientation (emergency department (ED) or intensive care unit (ICU)) prior to early treatment initiation. In this context, the severity assessment is most of the time restricted to clinical tools. In this study, we describe the association between prehospital shock index (SI) and mortality at day 28 of patients with SS initially cared for in the prehospital setting by a mobile intensive care unit (MICU).

**Patients and methods:** Patients with SS cared for by a MICU between January 2016 and May 2019 were retrospectively analyzed. Association between SI and mortality was assessed by Odd Ratio (OR) with 95 percent confidence interval [95 CI] using propensity score analysis.

**Results:** One-hundred and fourteen patients among which 78 males (68%) were analysed. The mean age was 71 ± 14 years old. SS was mainly associated with pulmonary (55%), digestive (20%) or urinary (11%) infection. Overall mortality reached 33% (n = 38) at day 28. Median SI differed between alive and deceased patients: 0.73 [0.61–1.00] vs 0.80 [0.66, − 1.10], p < 0.001*). After adjusting for confounding factors, the OR of SI > 0.9 was 1.17 [1.03–1.32].

**Conclusion:** In this study, we reported an association between prehospital SI and mortality of patients with prehospital SS. A SI > 0.9 is a simple tool to assess severity and to optimize prehospital triage between ED and ICU of patients with SS initially cared for in the prehospital setting by a MICU. The association of SI with biomarkers may be helpful to improve the screening for SS and decision making of SS in the prehospital setting.

**Compliance with ethics regulations:** Yes.

### P-027 Ultrasound guidance and the set up of central venous catheters in emergency situations

#### Walid Sellami

##### Département d’anesthésie réanimation, LR12DNO1, Hôpital militaire de Tunis, Tunis, Tunisia

###### **Correspondence:** Walid Sellami (drsellamiwalid@yahoo.fr)

*Ann. Intensive Care* 2020, **10 (Suppl 1):**P-027

**Rationale:** In intensive care unit, we are confronted to place venous catheters in urgent situations. Although the femoral site is preferred, it remains difficult to handle in case of abdominal pelvic surgery or ventral position. In addition it does not allow to develop a diagnostic approach. The purpose of our study was to see if ultrasound guided cannulation of the internal jugular vein could be an alternative to the femoral one.

**Patients and methods:** It was a prospective, monocentric, observational, and comparative study conducted in the anesthesia resuscitation department of the Military Hospital Main Instruction of Tunis over a period of 12 months. There were 118 patients, 58 in the group “guided femoral vein catheterization (FVC)” and 60 in the group “guided internal jugular vein catheterization (IJVC)”. The rate of failure and complications (mechanical, infectious and thrombotic) was compared; the number of punctures and access times too. The threshold of statistical significance was chosen at 0.05.

**Results:** The failure rate was 10.3% for FVC, compared to 8.3% for IJVC (p = 0.47, (p > 0.05)). The risk of hematoma was 3.4% for FVC, 1.7% for IJVC (p = 0.5). No case of pneumothorax was noted, 2 malpositions of the IJVC were reported. Catheter-related infection was 6.9% for FVC, 3.3% for IJVC (p = 0.3). Venous thrombosis was 20.7% for FVC, 10% for IJVC, (p = 0.08). The number of attempts and the access time were lower for IJVC (respectively 1.3 ± 0.4 and 204 s ± 46.3 vs 1.9 ± 0.7 and 256.5 s ± 90.4 for the FVC, p < 0.0001).

**Conclusion:** The failure rate and complications were comparable between the 2 groups, but the ultrasound-guided internal jugular catheter appears to be faster to insert and requires fewer punctures, so it could be an alternative to the femoral one in emergency situations.

**Compliance with ethics regulations:** Yes.

### P-028 Neuromyelitis optica attacks treatment assessment and analysis

#### Hossein Mehdaoui, Maeva le Goic, Ruddy Valentino, Cyrille Chabartier, Jean-Louis Ferge, Dabor Resiere, Shazima Vally, Emmanuelle Guerin, Agathe Chaplain, Marie Sabia, Ronan Hinaut, Philippe Cabre

##### University hospital of Martinique, Fort-De-France, France

###### **Correspondence:** Hossein Mehdaoui (hossein.mehdaoui@chu-martinique.fr)

*Ann. Intensive Care* 2020, **10 (Suppl 1):**P-028

**Rationale:** Neuromyelitis optica (NMO) is a rare but severe disease. The prognosis of treated NMO attacks remains unclear. We evaluated our practice, the early evolution and the prognosis of NMO patients.

**Patients and methods:** An observational study was performed on patients with NMO attacks presenting with visual or medullar symptoms admitted for plasma exchange (PE) therapy from January 2017 to August 2019. Treatment efficiency was defined as a negative shift of the visual or motor disability score (EDSS). Nonparametric Mann–Whitney and Fisher exact tests were used for statistical analysis as required.

**Results:** Twenty-four patients had 110 PE sessions. Characteristics of the cohort are described in Table 1. 5 (20.8%) died from complications of NMO attacks. Treatment had an effect in 15 (85.2%) patients. The shift in the ambulatory and visual EDSS was respectively − 0.4 + 2.3 and − 1.7 + 1.7. The 5 non-survivor patients had all AQP4 antibodies (p < 0.05). Residual EDSS was higher in the non-survivor group (8.7 + 1.0 vs 6.1 + 1.7, p < 0.01). Pulse steroids were administered in 1 (20%) patient in the non-survivor group vs 15 (78%) patients in the survivor group (P < 0.05). Twelve (80%) patients previously given pulse steroid therapy responded to PE.

**Discussion:** We assessed the handling of NMO attacks and identified our flaws. We concluded that pulse steroid therapy should not be withheld or replaced by lower dosage. We also need to find a way to make attacks identified by physicians earlier to shorten the delay between its onset and patient’s admission in a specialized care unit. We observed that the mean improvement is modest during the early phase of our treatment. But a modest improvement in the EDSS can have a great impact in the patient’s quality of life and even survival.

**Conclusion:** NMO attacks remain a threatening disease despite aggressive treatment. Shortening the delay of treatment and ensure adequate pulse steroid therapy coupled to PE could be a way to improve the prognosis.

**Compliance with ethics regulations**: Yes.

**Table 1:** Characteristics of the cohort

Age	45 ± 17
Sex ratio (F/M)	3.0 (18/6)
Acute myelitis	15 (62.5%)
Optic neuritis	16 (66%)
AQP4 Antibodies	12 (52%)
MOG Antibodies	4 (17%)
Seronegative patients	7 (30%)
Pulse steroid administration	16 (66.7%)
Pulse steroid administration delay	7.5 ± 7.7 d
Plasma exchange	22 (91%)
Plasma exchange delay	16.1 ± 10.8 d
Deaths	5 (20.8%)

### P-029 Guillain-Barré syndrome in Intensive Care Unit: a 10 years experience

#### Olfa Turki, Rezk Gorbel, Mariem Dlela, Hela Kallal, Kamilia Chtara, Mabrouk Bahloul, Mounir Bouaziz

##### Habib Bourguiba University Hospital, Sfax, Tunisia

###### **Correspondence:** Olfa Turki (olfa.turki.rea@gmail.com)

*Ann. Intensive Care* 2020, **10 (Suppl 1):**P-029

**Rationale:** Guillain-Barré syndrome (GBS), an acute inflammatory demyelinating polyneuropathy, is the most common generalized paralytic disorder. One-third of patients with GBS require admission to the intensive care unit (ICU). Respiratory failure, which is the major problem, may require mechanical ventilation (MV) and is associated with additional complications, significant risk of morbidity, mortality, and incomplete recovery. This study sought to describe the demographic, clinical, laboratory and neurophysiological characteristics of patients with GBS who were hospitalised in ICU between 2010 and 2019.

**Patients and methods:** We conducted a single-center, retrospective, observational, epidemiological study. 37 patients with confirmed diagnosis of GBS were admitted to the ICU in our university hospital center over a 10-year period and they were all included.

**Results:** Thirty-seven patients were included. There were 17 (45.9%) female and 20 (54%) male patients. The mean age was 40.75 ± 23.84 years. The mean delay of ICU admission was 10.67 days. Ventilatory failure was the common cause for ICU admission. The majority of the patients (43.2%) had a history of respiratory infection. The clinical feature was areflexic flaccid weakness for 32 patients (86.5%). Albuminocytological dissociation was observed for 56.8% of the patients. The major clinical subtypes of GBS were acute motor-sensory axonal neuropathy and acute motor axonal neuropathy (55%). The mean SOFA score was at 1.82 ± 2.27, 19 patients (51.4%) required MV. The mean ICU LOS was at 11.72 ± 11.56 days. The most observed complications during ICU stay were pneumonia (29.7%), sepsis (16.2%), pulmonary embolism (13.5%), bed-sores (5.4%) and dysautonomia (5.4%). Overall, ICU mortality was 18.9%, and increased to 35% in the MV group. The poor prognosis was significantly associated with MV and nosocomial infection (p = 0.017; p = 0.028 respectively).

**Conclusion:** GBS represents a small but increasing proportion of ICU admissions with more then half of patients receiving MV and with a poor prognosis. Larger, prospective, multi-centre studies will be required.

**Compliance with ethics regulations:** Yes.

### P-030 Medical treatment of immediate post partum hemorrhage

#### Janati Adnane, Lina Berrada, Amine Mohamed Amine

##### Obstetric intensive care unit, Casablanca, MOROCCO

###### **Correspondence:** Janati Adnane (adnanejanati@gmail.com)

*Ann. Intensive Care* 2020, **10 (Suppl 1):**P-030

**Rationale:** Immediate postpartum hemorrhage (HPPI) remains a real public health problem. It is the leading cause of death in obstetrics. It is an extreme emergency that brings into play the maternal–fetal vital prognosis. The aim of this work was to highlight the frequency of HPPI, its main etiologies as well as the therapeutic principles in order to improve the prognosis and reduce the morbidity and the maternal–fetal mortality linked to it.

**Patients and methods:** We conducted a 5-year retrospective study from January 1, 2014 to December 31, 2018 in the department of anesthesia and obstetric resuscitation at the IBN ROCHD CHU, including immediate postpartum hemorrhages managed by medical treatment.

**Results:** During the study period, there were 123 cases of immediate postpartum haemorrhage, 101 cases of haemorrhage medically treated (82.11%). The average age of the parturients was 30 years with extremes of 16 and 42 years old. Multiparas accounted for 48.5% of cases (49), and primiparas accounted for (28.7%) (29). There were 81 (80.2%) of the cases referred by peripheral hospitals, 89 (88.11%) parturients led a pregnancy not followed. Vaginal delivery occurred in 86 (85%) cases; of which 9 cases (8.9%) of instrumental extraction. The delivery was spontaneous in 35 cases (34.6%), artificial in 15 cases (14.85%) and directed in 51 (50.5%) cases, the initial clinical examination found an uterine inertia in 43% of cases, placental retention in 37%, then cervico-vaginal wounds in 19% of cases. Initial management was mainly represented by uterine massage in 30% of cases and wound sutures in 14% of cases. Blood transfusion was required in 12 (11.8%) of the cases, oxytocin in all cases and misoprostol in 61 (60%) of the cases. The outcome was favorable with stopping bleeding in 100 (99%), and 44 (43%) of the cases were anemia-dominated.

**Conclusion:** Early, effective and multidisciplinary medical treatment allows a reduction in morbidity and mortality related to immediate postpartum haemorrhage.

**Compliance with ethics regulations**: NA.

### P-031 Predicting outcome in ICU trauma patients with AKI

#### Mariem Dlela, Kamilia Chtara, Farah Zouari, Mabrouk Bahloul, Mounir Bouaziz

##### Habib bourguiba hospital, Sfax, Tunisia

###### **Correspondence:** Mariem Dlela (mariem241090@gmail.com)

*Ann. Intensive Care* 2020, **10 (Suppl 1):**P-031

**Rationale:** Acute kidney injury in trauma patients is a problem that has been little studied in the intensive care unit (ICU). Its occurrence has been shown to be associated with high morbidity and mortality. We aim to determine the outcome of ICU trauma patients with acute kidney injury (AKI), including the incidence of death in the ICU, of non-reversible renal impairment and ICU complications.

**Patients and methods:** This is a prospective study, conducted in the department of emergencies and ICU, including trauma patients with a minimum ICU stay of 7 days. Renal failure was defined based on the new KDIGO classification. Predictors of mortality and poor outcome were identified using univariate and then multivariate analysis.

**Results:** One hundred and fifty patients were admitted during the study period for the management of post-traumatic injuries, among which 98 patients were included. The incidence of AKI in the studied population was 47% (46 cases) with 26 (56%) diagnosed with stage one, ten (22%) with stage two and ten (22%) with stage three. The overall mortality of patients with post-traumatic AKI was 34.8% (16 patients) with a mean ICU lengh of stay (LOS) at 22 ± 18 days and of days on ventilator at 17 ± 15. Eight patients (17.4%) needed renal replacement therapy and thirty-four had non-reversible renal impairement (74%). During ICU stay, eight patients (17%) were diagnosed with pulmonary embolism. On univariate analysis, the following variables were associated to mortality in patients with post-tramatic AKI including; age, hemodynamic instability on the day of diagnosis and bilirubin levels on the day of AKI diagnosis. Besides, according to our analysis, the use of renal replacement therapy and the non-reversibility of renal impairment during ICU stay were also associated to ICU mortality. Among these factors, the non-reversibility of renal impairment in the ICU was a predictor of mortality on multivariate analysis (p = 0.009, OR = 29, CI 4–142). In this cohort, the following variables were predictive of non-reversible renal impairment during ICU stay; including age (with a best cut-off of 55 years old), medical history of hypertension, higher ISS and diuretics’ administration. On multivariate analysis, the age (p = 0.004, OR = 0.9, CI 0.80–0.97) and use of diuretics (p = 0.003, OR = 33, CI 3.1–359) were associated to non-reversible AKI in the ICU.

**Conclusion:** Our study confirms that post-traumatic AKI in the ICU is associated to high morbidity and mortality. The identification of outcome predictors could be valuable to guide the management of AKI.

**Compliance with ethics regulations:** Yes.

### P-032 Predictors of post-traumatic AKI in the ICU

#### Mariem Dlela, Farah Zouari, Olfa Turki, Mabrouk Bahloul, Mounir Bouaziz

##### Habib bourguiba hospital, Sfax, Tunisia

###### **Correspondence:** Mariem Dlela (mariem241090@gmail.com)

*Ann. Intensive Care* 2020, **10 (Suppl 1):**P-032

**Rationale:** The occurrence of acute kidney injury (AKI) in trauma patients is a problem that has been little studied to date. Its presence has been shown to be associated with an increased risk of morbidity and mortality in affected individuals. To determine the incidence of post-traumatic AKI and identify its predictive risk factors that could be eventually prevented.

**Patients and methods:** This is a 10-month long prospective cohort-study, conducted in the department of emergencies and intensive care unit (ICU) of a university hospital, including trauma patients with a minimum ICU stay of 7 days. Renal failure was defined based on the new KDIGO classification. Predictors of AKI were identified using univariate and then multivariate analysis.

**Results:** One hundred thirty patients were admitted during the study period for the management of post-traumatic injuries, among which 86 patients were included. The incidence of AKI in the studied population was 53% (46 cases) with 26 (56%) diagnosed with stage one, ten (22%) with stage two and ten (22%) with stage three. On univariate analysis, older age and medical history of diabetes or hypertension were predictors of AKI. Injury assessment found traumatic brain injury (AIS > 3), Glasgow (GCS) on admission, and the diagnosis of fat embolism to be associated to post-traumatic AKI. Moreover, hemodynamic instability on admission and during ICU stay, shock-index on admission, the amount of fluid administered the use of vasoactive drugs, sepsis, hyperbilirubinemia, P/F ratio and acute respiratory distress syndrome (ARDS) were also associated to post-traumatic AKI. Among these factors, ARDS (p = 0.001, OR = 9, CI 6–100), fat embolism (p = 0.028, OR = 2, CI 1.6–2.5), Shock index (p = 0.02, OR = 15.2, CI 2.2–105), and bilirubin levels (p = 0.006, OR = 1.035, CI 1.01–1.06) were identified as independent predictors of post-traumatic AKI on multi-variate analysis. Besides, according to our analysis, the following variables were predictive of stage 3 AKI, including bilirubin levels on the day of AKI diagnosis (p = 0.026, OR = 1.032, CI 1.004–1.061), transfusions (p = 0.02, OR = 1.5, CI 1.1–1.9), fat embolism (p = 0.015, OR = 11.3, CI 1.6–75), and diuretics’ administration (p = 0.004, OR = 10.4, CI 1.8–57).

**Conclusion:** Post-traumatic AKI could be associated with significant morbi-mortality in the ICU. The identification of predictors from the initial onset of trauma could be valuable to guide its management.

**Compliance with ethics regulations**: Yes.

### P-033 Prevalence and risk factors of hypotension associated with preload-dependence during continuous renal replacement therapy in critically ill patients

#### Guillaume Chazot, Laurent Bitker, Mehdi Mezidi, Hodane Yonis, Louis Chauvelot, Paul Chabert, Laure Folliet, Jean-Christophe Richard

##### Service de Médecine Intensive Réanimation. Hôpital de la Croix-Rousse. Hospices Civils de Lyon, Lyon, France

###### **Correspondence:** Guillaume Chazot (guillaume.chazot@chu-lyon.fr)

*Ann. Intensive Care* 2020, **10 (Suppl 1):**P-033

**Rationale:** Hypotension is a frequent complication of continuous renal replacement therapy (CRRT) in intensive care patients. Postural tests (i.e. passive leg raising in the supine position or Trendelenburg in the prone position) combined with continuous measurement of cardiac output are highly reliable to identify preload dependence, and may provide new insights into the mechanisms involved in CRRT-related hypotension. We aimed to assess the prevalence and risk factors of preload-dependence associated hypotension during CRRT.

**Patients and methods:** We conducted a single-center prospective observational study in ICU patients with acute kidney injury KDIGO 3, started on CRRT in the last 24 h, and monitored with a PiCCO^®^ device. The primary endpoint was the rate of hypotension associated with preload dependence during the first 7 days of CRRT. Hypotension was defined as the occurrence of a mean arterial pressure below 65 mm Hg requiring any of the following therapeutic intervention (initiation or increase in vasopressor dose, discontinuation of fluid removal by CRRT, or fluid bolus). Preload dependence was assessed every 4 h, and during each hypotensive episodes, and was deemed present if the continuous cardiac index increased by at least 10% during a passive leg raising test. Data are expressed in median [1rst quartile-3rd quartile], unless stated otherwise.

**Results:** Twenty-three patients (61% male, age 70 [61–75] year, SAPS-2 71 [64–79]) were included 8 [2–16] h after CRRT initiation, and studied continuously for 95 [61–146] h. At inclusion, 19 patients (83%) underwent mechanical ventilation, 19 (83%) met the Sepsis-3 criteria, 22 (96%) were under norepinephrine with a median dose of 0.7 [0.3–2.7] µg kg min^−1^, and preload dependence was identified in 12 patients (52%, [95% confidence interval: 33–71%]). A median of 4 [3–7] hypotensive episodes occurred per patient over the observation period, for a pooled total of 130 hypotension episodes. 76 episodes (58% [CI95%: 50–67%]) were associated with preload-dependence, 52 (40%, [CI95%: 32–49%]) without preload-dependence, and 2 were unclassified. Multivariate analysis (using variables collected prior to hypotension) identified the following variables as risk factors for the occurrence of hypotension associated with preload-dependence: preload-dependence before hypotension (odds ratio = 4.01, p < 0.001), fluid removal rate by CRRT (OR = 0.71 per 1 increase in SD, p < 0.001), and lactate levels (OR = 1.32 per 1 increase in SD, p < 0.001).

**Conclusion:** In this single center study, preload dependence-associated hypotension was slightly more frequent than hypotension without preload dependence in ICU patients undergoing CRRT. Testing for preload dependence to adjust fluid removal could help prevent hypotension incidence during CRRT.

**Compliance with ethics regulations**: Yes.

### P-034 Comparison of the RIFLE, AKIN, CK and KDIGO criteria to predict acute renal failure and mortality in critically ill patients

#### Rania Ammar Zayani, Emna Ennouri, Karama Bouchaala, Sabrine Bradai, Chokri Ben Hamida, Mounir Bouaziz

##### Faculty of medecine, university of sfax, Sfax, Tunisia

###### **Correspondence:** Rania Ammar Zayani (rania.ammarzayani@gmail.com)

*Ann. Intensive Care* 2020, **10 (Suppl 1):**P-034

**Rationale:** Acute kidney injury is a common complication in critically ill patients. Since 2004, various criteria have been proposed to define and grade acute kidney injury (AKI). Objective: To assess the power of the different methods of AKI classification to predict incidence of ARF and mortality.

**Patients and methods:** A prospective study conducted in a Tunisian intensive care unit (ICU) over a period of 6 months (January to June 2018). We included patients ≥ 18 years old. Acute renal failure (ARF) was defined according to the RIFLE, AKIN, CK and KDIGO criteria. We excluded patients < 18 years, patients with end-stage chronic renal disease (CKD) already undergoing iterative hemodialysis, patients with CKD without information on baseline creatinine levels and patients with hospital stay less than 48 h.

**Results:** During the study period, 428 patients were admitted in our ICU. We included 171 patients. The mean age (SD) was 49.26 ± 19.86 years. Sex ratio at 2. The mean (SD) APACHE II was 15.1 ± 7.5. The mean (SD) SOFA was 6.7 ± 2.9. The mean (SD) length of stay was 14.2 ± 12.1 days. The mortality rate was 30%. Among the patients included, 81 patients (47%) had creatinine levels that corresponded to at least one of the 4 definitions RIFLE, AKIN, KDIGO or CK. In univariate analysis, ARF predictive factors were age (p < 0.00001), hypertension (p = 0.002), diabetes mellitus (p = 0.002), heart failure (p = 0.002), shock (p = 0.001), polytrauma (p = 0.001), hypoxemia (p = 0.001), APACHE II score (p = 0.000) and SOFA score (p = 0.001). In multivariate study, the risk factors independently associated to the occurrence of ARF were: age (OR1.030, CI [1.006–1.054]; (p = 0.014)), APACHEII score (OR1.079, CI[1.005–1.158]; (p = 0.036)) and shock at admission (OR 15.561, CI[1.751–138.266]; p = 0.014)). The comparison between the different classifications showed that the incidence of ARF varied according to the classification adopted. The incidence according to AKIN and KDIGO were identical (46.8%) and very closer to the general incidence which was described above (47%). The incidence according to RIFLE criteria was 32.2% and 45% according to CK criteria. The ROC curve showed that the KDIGO and AKIN curves were confused and had the best predictive power of ARF occurrence with AUC = 0.994 than CK (AUC = 0.975) and RIFLE (AUC = 0.840). The mortality predictive powers of each of the classifications studied by analyzing the ROC curve showed that the AKIN and KDIGO curves were confused and had the best predictive power with AUC = 0.763 than CK (ASC = 0.753) and RIFLE (ASC = 0.696).

**Conclusion:** The AKIN and KDIGO criteria were good tools to estimate the incidence of ARF and to predict mortality in critically ill patients.

**Compliance with ethics regulations:** Not applicable.

### P-035 Renal replacement therapy in a Medical Intensive Care Unit: Prognosis and challenges

#### Ghalia Boubaker^1^, Asma El Hadhri^1,2^, Khaoula Meddeb^1^, Wafa Zarrougui^1^, Emna Ennouri^1^, Amal Triki^1^, Imen Ben Saida^1^, Imed Chouchene^1^, Mohamed Boussarsar^1^

##### ^1^Medical intensive care unit farhat hached university hospital, Université de Sousse, Faculté de Médecine de Sousse, LR No LR12SP09. Heart Failure, Sousse, Tunisia; ^2^Hospital hygiene unit, Farhat Hached University Hospital, Sousse, Tunisia

###### **Correspondence:** Ghalia Boubaker (boubaker15ghalia@gmail.com)

*Ann. Intensive Care* 2020, **10 (Suppl 1):**P-035

**Rationale:** Severe acute kidney injury (AKI) is a well-recognized complication of ICU patients with an important impact on mortality. Renal replacement therapy (RRT) represents a considerabe escalation in the complexity and cost of care for those patients. The aim of the present study is to describe characteristics and outcomes of hemodialyzed patients in a medical ICU.

**Patients and methods:** This is a retrospective study including patients who received RRT from January 2013 to September 2019 in the ICU of Farhat Hached Sousse –Tunisia. Were collected all patients characteristics including underlying condition, diagnostic and severity at admission, ARF characteristics, RRT characteristics and outcomes.

**Results:** 40 patients received intermittent haemodialysis. They were 61.3 ± 20 years mean aged; 20 (52%) male. Mean SAPS II 47.9 ± 15.5; mechanical ventilation 27 (71%); vasopressors 28 (74%). Mean ICU length-of-stay (LOS) 7.53 ± 6.9 days. The most frequent etiology of ARF requiring RRT was tubular necrosis 26 (68%) with oligoanuria 33 (86.8%). The average timing to initiation of RRT after the onset of ARF was 29 ± 30 h. Only one haemodialysis session was necessary in 27 (71%). The most frequent indications of haemodialysis were severe acidosis 14 (36%) and pulmonary edema 10 (26%). Median duration of sessions was 3.9 [4–4] h. Mean LOS after ARF onset was at 4.8 ± 5.1 days. Median LOS after first haemodialysis session and last session were respectively 2.7 [1.27–4.95] days and 21.5 [12.5–60.75] h. Mortality rate was at 26 (68.5%).

**Conclusion:** The present study demonstrated the high severity and poor outcome of patients who received RRT leading to question the indication, delays, dose and efficiency of RRT.

**Compliance with ethics regulations:** Yes.

### P-036 Sepsis induced acute kidney injury: incidence, risk factors and prognostic impact in critically ill patients

#### Karama Bouchaala, Rania Ammar Zayani, Chokri Ben Hamida, Mabrouk Bahloul, Mounir Bouaziz

##### Habib bourguiba university hospital, Sfax, Tunisia

###### **Correspondence:** Karama Bouchaala (karamamnif@gmail.com)

*Ann. Intensive Care* 2020, **10 (Suppl 1):**P-036

**Rationale:** Acute kidney injury (AKI) is a frequent complication in critically ill patients and is associated with increased morbidity and mortality. Sepsis is one of the most common cause of AKI.

**Patients and methods:** A prospective study was conducted over 6 months (January 01–June 30, 2018). We included patients with septic shock at admission or at any time during hospitalization. Septic shock was defined according to the Third International Consensus definitions for Sepsis and Septic Shock. The AKI staging was based on KDIGO criteria. Patients were divided into two groups, a group with AKI (AKI +) and a group without AKI (AKI-). Then we compared the baseline characteristics, laboratory and physiologic data. Patients with AKI (AKI +) were subdivided according to their prognosis.

**Results:** Were enrolled 75 patients. The mean (SD) age was 56.43 (± 18) years. Sex ratio was 1.91. Fifty-two (70%) patients developed AKI. SAPS II and SOFA score in admission were higher in patients with kidney injury [59 vs. 44 points (p = 0.002), 6.5 vs. 4 points; (p = 0.003)] respectively. The serum lactate level was significantly higher in (AKI +) group patients during the first day of septic shock [6.12 ± 1.38 mmol/l (AKI +) vs. 4.11 ± 0.79 mmol/l (AKI-); (p = 0.002)] and its clearance was lower [ (32 ± 10.99% (AKI +) vs. 61 ± 13% (AKI-); (p = 0.001)]. A significant difference was observed in C reactive protein level [224 ± 114 mg/l (AKI +) vs. 124 ± 77 mg/l (AKI-); (p = 0.004)]. Among (AKI +) patients, KDIGO III was observed in 59.6% of cases. Nineteen (36.5%) patients received hemodialysis. A normal kidney function was recovered in 40.4% of cases. AKI+ patients had a higher occurrence in disseminated intravascular coagulation (32 vs. 3 patients, p = 0.002), acute respiratory distress syndrome (18 vs. 2 patients; p = 0.023) and cardiac dysfunction (20 vs. 1 patient, p = 0.001). Mortality was higher in AKI group (67% vs. 9%; p = 0.001).

**Conclusion:** The development of septic AKI was associated with poor outcomes and prognosis. A better understanding of sepsis induced AKI pathway will enable us to develop targeted therapeutic protocols. Newer tools, permitting AKI early detection, may make these therapies more fruitful.

**Compliance with ethics regulations**: Yes.

### P-037 Is augmented renal clearance a predictor of morbidity and mortality in severely burned patients?

#### Somai Mehdi, Amel Mokline, Laajili Achref, Kawther Faleh, Hager Zouari, Hana Fredj, Manel Ben Saad, Amenallah Messaadi

##### Centre de traumatologie et des grands brulés, Tunis, Tunisia

###### **Correspondence:** Somai Mehdi (mehdi.somai.87@gmail.com)

*Ann. Intensive Care* 2020, **10 (Suppl 1):**P-037

**Rationale:** Augmented renal clearance (ARC) is common in critically ill patients. This may affect PK/PD characteristics of antibiotics. Our study aims to evaluate the prognostic impact of ARC in severely burned patients with septic shock.

**Patients and methods:** Retrospective, descriptive and comparative study, conducted in intensive burn care department in Tunis over 1 year (October 2017-September 2018). Were included adult patients with an acute burn surface area (ABSA) > 20% and an ICU stay > 3 days. ARC is defined as a creatinine clearance > 130 ml/min during a period ≥ 3 days.

**Results:** 70 patients were included. Two groups were individualized: G1 [ARC + , n = 43] and G2 [ARC-, n = 27]. The average age was 39.5 ± 14.4 years for G1 vs. 46.3 ± 21.1 years for G2 (p = 0.15). The ideal average weight (8 kg for both groups) and admission weight (78 kg vs 77 kg) were comparable for both groups, as well as for the delta weight (9.6 vs 8.3; p = 0.77). Burns were less extensive for G1 (ARC +) compared to G2 (ARC-), respectively 40.1 ± 15.7% vs. 46.2 ± 19% (p = 0.15). The use of mechanical ventilation and catecholamines was comparable in both groups (81% for G1 vs. 85% for G2; p = 0.75). Although the frequency of septic shock occurrence was comparable in both groups (75% for G1 vs. 71% for G2; p = 0.71), septic shock mortality was statistically higher in patients with ARC +  (100% for G1 vs. 78% for G2; p = 0.014).

**Conclusion:** ARC in severely burned patients is accompanied by a high mortality in septic burn patients (100%), related probably to sub-therapeutic exopsure of antibiotics in these patients. So, therapeutic drug monitoring of antibiotics must be necessary to guide dose optimization, reduction of therapeutic failures and emergence of resistant bacteria.

**Compliance with ethics regulations**: Yes.

### P-038 Post-traumatic acute renal failure in critically ill patients: risk factors

#### Rania Ammar Zayani, Karama Bouchaala, Yousfi Mounir, Kamilia Chtara, Mabrouk Bahloul, Chokri Ben Hamida, Mounir Bouaziz

##### Faculty of Medicine, University of Sfax, Tunisia, Sfax, Tunisia

###### **Correspondence:** Rania Ammar Zayani (rania.ammarzayani@gmail.com)

*Ann. Intensive Care* 2020, **10(Suppl 1):**P-038

**Rationale**

Acute renal failure (ARF) in polytrauma was a life-threatening complication.

**Objective:** To determine the factors that predicts the occurrence of ARF.

**Patients and methods:** A cohort study including patients with trauma in an intensive care unit for 3 months studying the occurrence of ARF. The ARF was defined according to KDIGO criteria.

**Results:** We include 37 patients. The incidence of ARF was 24.32% (9 patients). Patient in the group ARF (ARF+), the mean age was 38.66 ± 19.43 years, the mean SAPS2 was 41.88 ± 15.25, the mean SOFA score was 12.33 ± 10.68 and the average mean arterial pressure (MAP) was 77.3 ± 28.02 mmHg with catecholamine’s used in 77.77%. Patients in the group non ARF (ARF−), the mean age was 36.07 ± 15.5 years, the mean IGS2 was 25.57 ± 10.52 and the mean SOFA was 6.6 ± 7.81 and the mean MAP was 99.57 ± 24.21 mmHg with catecholamine’s used in 0.39%. The mean time of occurrence of ARF was the 2.11 ± 2.26 days. According to KDIGO criteria: stage 1 (5 patients), stage 2 (2 patients) and stage 3 (2 patients). Oligoanuria was observed in 2 patients. One patient required hemodialysis. The return to normal renal function was observed in 7 patients. A mortality rate was 33.33% in (ARF+), while it was 7.14% in (ARF−). The SAPS2 was statistically significant between the two groups (p = 0.01). The analysis of the ROC curve showed that SAPS2 > 31.1 points was statistically associated with the occurrence of ARF (p = 0.002), sensitivity 77.8%, specificity 75% and AUC 0.839.

**Conclusion:** Predictive factors of the occurrence of post-traumatic acute renal failure in critically ill patients are related to the nature and clinical consequences of the trauma.

**Compliance with ethics regulations:** Yes.

### P-039 Velocity time integral in transcranial doppler (TCD) in healthy controls (C) versus ICU patients (P) for detection of cerebral blood flow (CBF) abnormalities

#### Jack Richecoeur^1^, Danièle Combaux^1^, Anne Sagnier^1^, Romain Mercier^1^, Nathalie Verrier^1^, Romain Debock^1^, Marie-Anais Bastide^1^, Cecile Caplin^1^, Chloe Soulignac^1^, Jean-Louis Dubost^2^, Bruno Gelee^2^, David Luis^1^

##### ^1^CH Simone Veil, Beauvais, France; ^2^CH Rene Dubos, Pontoise, France

###### **Correspondence:** Jack Richecoeur (jack.richecoeur@gmail.com)

*Ann. Intensive Care* 2020, **10(Suppl 1):**P-039

**Rationale:** Few studies report the relation between functionnal brain alterations during and after ICU stay and abnormalities of CBF displayed on TCD. Using VTI as hemodynamic parameter is unusual for evaluation of CBF. The purpose of this preliminary study was to compare the values of VTI of healthy Controls (C) versus ICU (P) with usual parameters (i.e. diastolic (Vd) and mean velocities (Vm), resistance (IR) and pulsatility index (IP)).

**Patients and methods:** 38 C and 42 Pts were consecutively included during a 2 months period (August to September 2019). Brain damaged Pts and Pts with no temporal window were excluded segment M1 middle cerebral artery (MCA) velocities and VTIMCA were sampled using TCD. Left ventricular out flow (VTIAo) was assessed by transthoracic echocardiography. Demographics data, reason for admission in ICU, SAPS, sedative, vasopressor use and ventilator support were recorded as well as MAP, Vs, Vd, Vm, IP, IR, arterial blood gaz. Data are shown as median (interquartile : 25–75). T-test or non parametric test (if appropriate) were used. P < 0.05 was considered as significant.

**Results:** Age sex ratio were respectively 40 y (30–51.75), sex ratio:1 for C and 58 y (50.0–64.5), sex ratio: 2.5 Pts group with SAPS II 52 (41–69). Reasons for admission were sepsis: 14, cardiac arrests: 7, intoxications: 4, cardiogenic shock: 4, others: 9. All patients but 7 had MV, vasopressor support: 16 Pts and sedative drugs: 33 Pts. 25 Pts had low VTIAMCA (range 13.67 to 34 cm) under the lowest value of 35 cm among C. 7 Pts among the 25 Pts had low Vd, below the cut-off of 25 cm/s linked to CBF abnormalities. In addition, low VTIAo and Vd was significantly correlated to low VTIMCA but not Vm.

**Conclusion:** Pts versus C had dramatically collapsed VTIMCA. Using VTIMCA cut-off of 35 cm, more Pts (2/3) might have abnormal CBF compared Vd threshold of 25 cm/s. Low VTIAo should prompt VTIMCA assessment.

**Compliance with ethics regulations:** Yes.

**Table 1:** Summarize relevant data

	C (n=38)		P (n=38)		p
MAP mmHg	95.00	(89.00–98.00)	79	(65.50–64.50)	< 0.001
ITVao cm	21.50	(19.50–30.00)	16	(13.90–18.75)	< 0.001
ITVMCA cm	50.80	(44.00–60.00)	30.30	(22.93–39.55)	< 0.001
Vs cm/s	90.70	(57.00–104.0)	84.30	(52.58–109.50)	0.11
Vd cm/s	40.30	(35.00–50.00)	31.32	(20.50–43.68)	0.03
Vm cm/s	58.50	(54.70–66.15)	43.96	(28.38–60.95)	0.01
IR	0.55	(0.500–0.587)	0.591	(0.5160–0.690)	NS
IP	0.88	(0.726–1.01)	0.900	(0.729–1.240)	NS

### P-040 Habituation to the auditory startle reflex is a new sign of minimally conscious state

#### Bertrand Hermann^1^, Amina Ben Salah^1^, Mélanie Valente^2^, Lionel Naccache^3^

##### ^1^Université Paris Descartes, Paris, France; ^2^Institut du Cerveau et de la Moelle Epinière, Paris, France; ^3^Sorbonne Université, Paris, France

###### **Correspondence:** Bertrand Hermann (bertrand.hermann@gmail.com)

*Ann. Intensive Care* 2020, **10(Suppl 1):**P-040

**Rationale:** Accurate diagnosis of the level of consciousness is a challenge and different states such as coma, vegetative state (VS) or minimally conscious state (MCS) are often confused while they convey meaningful prognostic information. This distinction rely on the Coma Recovery Scale-revised (CRS-R) gold-standard. However, this clinical scale is imperfect since unresponsive patients can exhibit genuine signs of consciousness using advance neuroimaging techniques. Expanding the range of behaviors indexing consciousness at bedside is thus of decisive importance.

**Patients and methods:** We designed and proposed a new clinical sign of MCS, the habituation to auditory startle reflex (ASR), based on the blink response to repeated sounds: either inhibition of the automatic ASR response (extinguishable) or no habituation (inextinguishable response). We prospectively tested this new sing in patients suffering from disorders of consciousness after severe brain injury and first compared its diagnostic performances with the current gold-standard (CRS-R) using standard discrimination metrics (AUC, sensitivity, specificity, likelihood ratios) and their 95% confidence interval. We then investigated the correlates of this new sign on two validated neuroimaging diagnostic procedures (multivariate EEG-based classification of the state of consciousness and FDG-PET metabolic index of the best preserved hemisphere) using an ANOVA with the state of consciousness and the ASR response as independent variable.

**Results:** 96 patients were included between January 2014 and July 2019, mean age 44.2 ± 16.4 years, sex ratio 1.8, median delay since injury 58 [31–236] days, main etiologies anoxo-ischemic encephalopathy (41%) and traumatic brain injury (28%), with 51% patients under mechanical ventilation. Among the 48 VS patients 32 had an inextinguishable response and 16 an extinguishable one, while the reverse pattern was found in the 48 MCS patients with 16 and 36 patients, respectively. The AUC of an extinguishable response to diagnose MCS was 0.71 [0.61–0.8] with 75% [60–86] sensitivity, 67% [50–80] specificity, 2.25 [1.46–3.47] positive and 0.38 [0.22–0.64] negative likelihood ratios, ranking second among all the CRS-R items defining MCS. Furthermore, patients with an extinguishable response had a significantly higher probability of being classified MCS on the EEG-based algorithm and higher metabolic index on the PET, independently of their clinical state of consciousness (main effect of ASR habituation, p = 0.0002 and p = 0.0391 respectively, Fig. 1).

**Conclusion:** A successful inhibition of the automatic ASR response is a valid new sign of MCS compared with behavioral and neuroimaging validated procedures. This simple sign could be easily implemented at bedside as a screening tool or complement to the time-consuming CRS-R.

**Compliance with ethics regulations:** Yes.

**Fig. 1:**
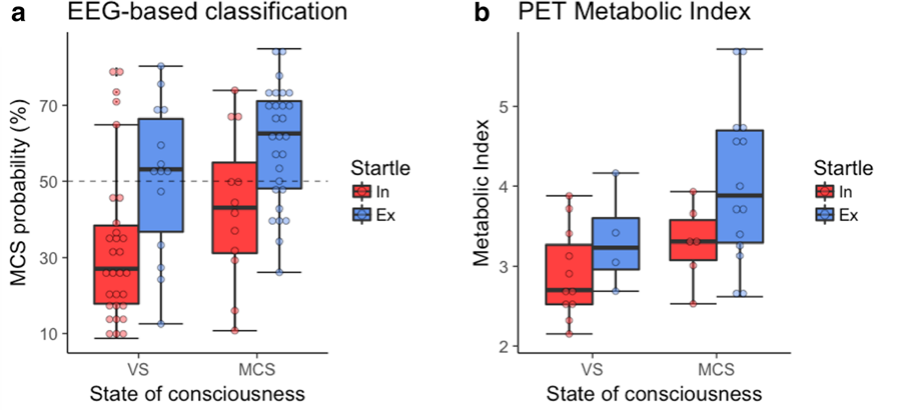
Main effect of the ASR response on EEG-based classification and PET metabolic index

### P-041 Teaching the basics of bedside electroencephalography to critical care staffs: a prospective multicentre study

#### Stéphane Legriel^1^, Gwenaëlle Jacq^1^, Amandine Lalloz^2^, Guillaume Geri^3^, Pedro Mahaux^3^, Cedric Bruel^4^, Sandie Brochon^4^, Benjamin Zuber^5^, Cécile Andre^5^, Krystel Dervin^6^, Mathilde Holleville^6^, Alain Cariou^2^

##### ^1^Centre Hospitalier de Versailles, Le Chesnay, France; ^2^Hôpitaux Universitaires Paris Centre-Cochin, Paris, France; ^3^Hôpital Ambroise Paré, Boulogne, France; ^4^Groupe hospitalier Paris Saint Joseph, Paris, France; ^5^Hôpital Foch, Suresnes, France; ^6^Hôpitaux Universitaires Paris Nord Val de Seine-Beaujon, Clichy, France

###### **Correspondence:** Stéphane Legriel (slegriel@ch-versailles.fr)

*Ann. Intensive Care* 2020, **10(Suppl 1):**P-041

**Rationale:** Although continuous electroencephalography (cEEG) is commonly recommended in neurocritical care patients, implementation of this monitoring in routine is facing the need for a specific training of professionals. We evaluated the effectiveness of a training program for the basic interpretation of cEEG to critical care staffs in a prospective multicentre study.

**Patients and methods:** After completion of a pre-test, participants (physicians and nurses) recruited in 6 French intensive care units (ICU) received a face-to-face EEG learning course, followed by additional e-learning sessions at day-1 (post-course), day-15, day-30 and day-90, based on training tests followed by illustrated and commented answers. Each test was designed in order to evaluate knowledge and skills through correct recognition of 10 predefined EEG sequences covering the most common normal and abnormal patterns. The primary objective was to achieve a success rate of more than 80% of correct answers at day-90 in at least 75% of participants.

**Results:** Among 250 participants, 108 (43.2%) completed the full training program and 77 of these 108 (71.3%) full-training participants achieved at least 80% of correct answers at day-90. Paired comparisons between scores obtained at each evaluation demonstrated a statistically significant increase over time. At day 90, rates of correct answers were greater than 80% for all predefined usual EEG sequences, excepted for the recognition of periodic and burst-suppression patterns and reactivity, which were identified in only 42.6% (95% CI 36.4–48.8) and 60.2% (54.1–66.3) and 70.4 (64.7–76.1) tests, respectively.

**Discussion:** This multicentric prospective study, which evaluated a training program for the basics of electroencephalography offered to critical care teams, provides interesting information about the training process and its impact on learners according to their different characteristics. We believe that participants reflect the heterogeneity of the various use of cEEG in the critical care setting. Participants came from university and non-university ICUs, and whereas some of them used to monitor patients with cEEG, others were in an implementation process when the last monitored neurocritical care patients with intermittent EEG. In accordance with previous studies, we focused to the entire medical and nursing ICU staffs.

**Conclusion:** A 3-months training program aiming to teach the basic interpretation of continuous EEG in the intensive care units was associated with a significant attrition in participation over time. However, participants who received the full training program were capable to accurately recognize the vast majority of EEG patterns that are encountered in critically ill patients.

**Compliance with ethics regulations:** Yes.

### P-042 Prognostic factors for stroke in CHU Oran emergency department

#### Houria Djebli, Soumia Benbernou, Nabil Ghomari

##### Faculte de médecine d’Oran, Oran, Algeria

###### **Correspondence:** Houria Djebli (djeblihouria@yahoo.fr)

*Ann. Intensive Care* 2020, **10(Suppl 1):**P-042

**Rationale:** Stroke is a real public health problem in the world, it is the second leading cause of death in Western countries. In Maghreb countries such as Algeria, few studies have addressed the issue. A study in this field had become necessary, thus making it possible to determine the frequency as well as the prognostic factors of this pathology. The aim was to study the prognostic factors related to mortality at 1 month and 1 year.

**Patients and methods:** Prospective descriptive study for analytical and prognostic purpose carried out during the period from January 1, 2016 to December 31, 2017. Any patient over the age of 15 with signs of a sudden loss of facility whose diagnosis of stroke is confirmed by the CT scan. Patients with recurrent stroke. The Cox regression model for uni-varied and multi-variate analyzes was designed to study the prognostic factors associated with 28-day mortality and 1-year mortality following stroke.

**Results:** This is a study that has collected 1212 patients admitted to the Medical Emergencies of CHU Oran for Stroke. Their prevalence was 61.7. Stroke accounts for 19.31% of all emergency room visits 55.3% are female, average age: 64.22+ or − 17.45. 78% were ischemic stroke, 15% hemorrhagic, 4% cerebral venous thrombosis and 3% meningeal hemorrhage. Only 8.2% of patients arrived within a time frame for thrombolysis. The final model of the 1-year Cox model survival analysis retained three prognostic factors at a significance level α = 5%. The age at which the risk of death is multiplied by 2.86 (HZa = 2.86, [1.89–4.34]). The number of Comorbidities factors multiplies the risk of death by 2.25 times (HZa = 2.25; [1.33–3.80]). In hypertensive patients, the probability of survival at 6 months was 65.6% CI [60.4–70.8]; however, it was 73.1% CI [67.1–79.1] in non-hypertensive patients. The survival rate of patients with no risk factor was 83.9% [76.9–90.9] whereas those with 4 or more risk factors were 65.4%. IC [52.2–78.6]; p = 0.039. The Glasgow Coma initial score where the risk of death is multiplied by 6 and a half times (HZa = 6.55; [4.68–9.17]). Survival was 78.4% at 30 days compared with 63.2% at 1 year.

**Conclusion:** Stroke is a common pathology in emergencies, knowledge of their prevalence and prognostic factors can establish appropriate strategies for management.

**Compliance with ethics regulations:** Yes.

### P-043 The clinical and progressive aspects of cerebral venous thromboses at the Oran hospital and university center

#### Mourad Goulmane

##### Oran Hospital and University Center, Oran, Algeria

###### **Correspondence:** Mourad Goulmane (goulmane.mourad@univ-oran1.dz)

*Ann. Intensive Care* 2020, **10(Suppl 1):**P-043

**Rationale:** Cerebral venous thrombosis (CVT) is a rare but very serious disease with various clinical and etiological aspects. Unlike ischemic arterial accidents, epidemiological studies are limited. The aim of our work was to study the clinical, etiological and evolutionary features of CVT in the Algerian population from a sample of 28 patients.

**Patients and methods:** This is a retrospective observational study conducted in the neurology department of the CHU d’Oran between January 2016 and December 2017. In a clinical context suggestive of CVT, the diagnosis of certainty was provided by brain MRI coupled with MRA. All subjects benefited from a complete etiological assessment. The anticoagulant treatment was based on the low molecular weight heparin relayed by the anti-vitamin K. The duration of the follow-up was 12 months.

**Results:** The mean age was 38.26 ± 13.59 years, the sex ratio was 6 (24F/4H), the onset was subacute in 55% of cases. The main early signs were headache (88.8%), visual disturbances (50%), epileptic seizures (44.4%) and motor deficit (44.4%). Thrombosis predominated in the upper sagittal sinus and lateral sinuses; Parenchymal lesions were associated in 2/3 of the cases. Gynecologic obstetric causes were by far the most frequent. The evolution was favorable in 83.3% of the cases.

**Discussion:** CVT is characterized by its clinical polymorphism, its predominance in young women, and its most often favorable evolution. The causes are multiple and often intricate requiring the realization of a systematic etiological assessment even if the cause seems obvious. The treatment of choice remains early anticoagulation, based on heparinotherapy even in case of hemorrhagic softening.

**Conclusion:** The characteristics of CVT in the Algerian population are distinguished by a high frequency of gynecological obstetric causes. Awareness campaigns for women of childbearing age are useful.

**Compliance with ethics regulations:** Not applicable.

### P-044 Analysis on prognostic factors of patients with ruptured intracranial aneurysm in intensive care unit

#### Karim Badaoui, Rachid Cherkab, Chafik El Kettani, Barrou Lahoucine

##### CHU ibn Rochd, Casablanca, Morocco

###### **Correspondence:** Karim Badaoui (karimbadaoui61@gmail.com)

*Ann. Intensive Care* 2020, **10(Suppl 1):**P-044

**Rationale:** Subarachnoid hemorrhage due to ruptured intracranial aneurysm (SAH) is a serious affection with a global mortality of 26% in France. The purpose of this work was to screen the prognostic factors of the aneurysms operated at the acute phase.

**Patients and methods:** Retrospective study spread over 7 years (from 1st January 2012 to 1st January 2019), performed in the surgical emergency department (P17) Ibn Rochd, including all patients operated for SAH. All demographic, clinical, biological and therapeutic data were collected. Univariate analysis used Chi-square tests and Student’s “t” test, while multivariate analysis was performed using the logistic regression model. The measurement of the scores discriminating power was based on the Receiver Operating Characteristic (ROC) curves and the comparison of the areas under the curves with the Hanley and McNeil method survival was estimated by the Kaplan–Meier method followed by the Log-rank test for comparison of survival curves.

**Results:** 132 patients were included with an average age of 50 ± 10 years. Mortality was 31.3%. Univariate analysis identified 12 prognostic factors that had an impact on mortality: poor Glasgow Coma scale (p < 0.01), WFNS III and IV (p < 0.01), Hunt and Hess IV (p: 0.005), hyperthermia (p = 0.02), Fisher III and IV (p = 0.003 and 0.02), ventricular inondation (p = 0.02), anterior aneurysmal location (p = 0.000), tracheotomy (p = 0.001), postoperative deficit (p = 0.03), prolonged ventilation (p = 0.003), dysnatremias (p = 0.037 and p = 0.019) and onset time of surgery (p = 0.000). In multivariate analysis, the independent variables that appeared to be of poor prognosis were: a prolonged duration of ventilation (OR: 2.56), a prolonged duration of surgery (OR: 0.36), a WFNS IV score (OR: 0.65)

**Conclusion:** Mortality related to intracranial aneurysm remains high hence the interest of early and adapted management of any arachnoid hemorrhage by rupture of cerebral aneurysm.

**Compliance with ethics regulations:** Yes.

**Table 1:** Comparison of our results with medical literature

	This study (2012/2019)	Rivero Rodriguez et al. (2005–2014)	Jaja et al. (2015)	Meneghelli et al. (2016)	Charpentier et al. (1999)	Qureshi et al. (1999)	Spatenkova et al. (2017)
Age		p = 0.31		p = 0.023	p = 0.008		
Sexe		p = 0.0067					
Atcdshta		p = 0.0001	P < 0.01				
GCS	p < 0.01					p = 0.014	
Fever	p = 0.02						
Wfns score	p < 0.01	p = 0.0021	p = 0.05 p < 0.001		p = 0.025		
Hunt et Hess	p = 0.05			p = 0.000			
Fisher ¾	p = 0.003 p = 0.02	p = 0.0008					
Anterior aneurismal location	p = 0.000						
Dysnatremia	p = 0.037 p = 0.019	p = 0.0073				p = 0.0083	P = 0.001 p < 0.01
Vasospasm		p = 0.0000			p = 0.001		
Hydrocephalus		p = 0.0039				p=0.011	
Prolongated ventilation	p = 0.003 p = 0.02						
Rebleeding		p = 0.0019					
Post-operative deficit	p = 0.03						
Onset time of surgery	p < 0.01						

### P-045 Decompressive hemicraniectomy in malignant middle cerebral artery infarction about 20 cases

#### Rania Ammar Zayani, Karama Bouchaala, Kamilia Chtara, Yousfi Mounir, Mabrouk Bahloul, Chokri Ben Hamida, Mounir Bouaziz

##### Faculty of Medicine, University of Sfax, Sfax, Tunisia

###### **Correspondence:** Rania Ammar Zayani (rania.ammarzayani@gmail.com)

*Ann. Intensive Care* 2020, **10(Suppl 1):**P-045

**Rationale:** Malignant middle cerebral artery (MCA) infarction is associated with an up to 80% mortality rate due to ischemic edema and brain herniation. The aim was to study the impact of decompressive hemicraniectomy (DC) in patients with MCA.

**Patients and methods:** Twenty patients with MCA treated with DC were studied during 5 years (2015–2019) in a Tunisian intensive care unit.

**Results:** Mean age was 59.3 ± 10.99 years. Mean length of stay was 24 ± 18.79 days. Mean duration of mechanical ventilation was 19. 05 ± 17.19 days. Mean SAPS II was 41. 6 ± 11.35. Mean SOFA was 9.1 ± 4.5. Mean GCS was 8.5 ± 3.06. The etiology was arrytmia (23. 8%), hypertensive urgency (52. 4%) and atherosclerosis (42.9%). The affected artery was the left sylvian artery in 55%. The operative indications were deterioration of the state of consciousness in all cases (100%), pupillary modification (25%), mass effect and deviation of the median line > 5 mm (100%), brain herniation (50%) and hemorrhagic transformation (25%). Mean time of DC was 3.1 ± 2. 55 days. Tracheotomy was performed in 14 patients (66. 7%) with a mean at 11.5 ± 6.44 days. Osmotic treatment was initiated in 76, 2% of cases and antiepileptic treatment in 52.4%. The mortality rate was 57.1%. The functional prognosis was assessed by the modified Rankin Score (mRS): a good prognosis (mRS between 0–3) was found in one patient, a poor prognosis (mRS between 4–6) was found in 19 patients. The complications associated with DC were brain abscess (14, 3%), meningitis (19%), local infection (19%), hemorrhagic transformation (61.9%) and convulsion (42.9%). SAPSII score was significantly associated with mortality (p = 0.23). SAPSII ≥ 41.5 points had Se 70%, Sp 70% and an area under the curve (AUC) 0.807. SOFA score was significantly associated with mortality (p = 0.007). SOFA ≥ 6.5 had Se 91.7%, Sp 75% and AUC 0.865.

**Conclusion:** Hemicranectomy decompression in sylvian malignant infarction don’t improve good outcomes in severe critically ill patients.

**Compliance with ethics regulations:** Not applicable.

### P-046 Prediction of outcome after acute stroke: comparison of CT-DRAGON score and a simplified score

#### Anouk Lesenne^1^, Jef Grieten^1^, Alain Wibail^1^, Ludovic Ernon^1^, Luc Stockx^1^, Patrick Wouters^2^, Elly Vandermeulen^1^, Sam Van Boxstael^1^, Pascal Vanelderen^1^, Sven Van Poucke^1^, Joris Vundelinckx^1^, Sofie Van Cauter^1^, Dieter Mesotten^1^

##### ^1^Ziekenhuis Oost-Limburg Genk, Genk, Belgium; ^2^Universiteit Gent/Universitair Ziekenhuis Gent, Gent, Belgium

###### **Correspondence:** Anouk Lesenne (anouk.lesenne@ugent.be)

*Ann. Intensive Care* 2020, **10(Suppl 1):**P-046

**Rationale:** The CT-DRAGON score was developed to predict long-term functional outcome after acute stroke in the anterior circulation treated by thrombolysis. Its implementation in clinical practice is hampered by the plethora of variables included. In addition, the score has not been validated in important subgroups such as stroke patients undergoing thrombectomy. Given these limitations, the current study was designed to evaluate the use of a simplified score based on machine learning, as a possible alternative.

**Patients and methods:** This single-centre retrospective study included 564 patients treated for stroke, in the anterior and posterior cerebral circulation, between 01-2017 and 02-2019. At 90 days, favourable (modified Rankin Scale (mRS): 0–2) and miserable outcome (mRS: 5–6) were predicted by CT-DRAGON. Machine learning selected the features of the simplified score. Discrimination, calibration and misclassification of both models were tested.

**Results:** 32% had proximal anterior stroke, 2% proximal posterior stroke and 50% lacunar infarcts in either circulation. In 16% no thrombus was objectivated. 13% of patients were treated with thrombectomy, 17% received thrombolysis and 9% underwent both thrombolysis and thrombectomy. 61% only received anti-platelet therapy. The area under the receiver-operating-characteristic curve (AUC-ROC) for CT-DRAGON was 0.78 (95% CI 0.74–0.81) for favourable and 0.78 (95% CI 0.72–0.83) for miserable outcome. R2 of CT-DRAGON was 0.30 and 0.22 for favourable (lack of fit, p = 0.75) and miserable (lack of fit, p = 0.8) outcome respectively. Misclassification rate was 29% for favourable and 13% for miserable outcome with CT-DRAGON. Selection of predictors from the CT-DRAGON was done by logistic regression, bootstrap forest and decision tree analysis. NIH Stroke Scale, pre-stroke mRS and age were identified as the strongest contributors to favourable and miserable outcome, and included in the simplified score. AUC-ROC was 0.83 (95CI% 0.79–0.86) and 0.83 (95CI% 0.77–0.87) for the prediction of favourable and miserable outcome respectively. R2 was 0.42 and 0.29 for the prediction of favourable (lack of fit p = 0.34) and miserable (lack of fit p = 1.0) outcome respectively. Misclassification rate was 26% for favourable and 14% for miserable outcome with the simplified score. The simplified score had better discriminative power than CT-DRAGON for both outcomes (both p < 0.005).

**Conclusion:** The CT-DRAGON score revealed acceptable discrimination in our cohort of both anterior and posterior circulation strokes, receiving a variety of treatment modalities. The simplified score had a better discrimination, while maintaining comparable and good specificity and misclassification rate for miserable outcome. The simplified score needs further validation in a prospective, multi-centre study.

**Compliance with ethics regulations:** Yes.

**Table 1:** Predictive performance of CT-DRAGON and simplified score

	Favourable outcome (%)	Miserable outcome (%)
CT-Dragon score		
Misclassification rate	29	13
Sensitivity	67	10
Specificity	74	100
Simplified score		
Misclassification rate	26	14
Sensitivity	77	23
Specificity	73	97

### P-047 Adherence to GOLD 2017 guidelines treatment recommendations in critically ill COPD patients

#### Imen Ben Saida, Hela Kallal, Sana Rouis, Wessem Ammar, Nesrine Fraj, Wafa Zarrougui, Rym Chelbi, Mohamed Boussarsar

##### Medical intensive care unit, farhat hached hospital, Université de Sousse, Faculté de Médecine de Sousse, UR N° LR12SP09.Heart Failure, Sousse, Tunisia

###### **Correspondence:** Imen Ben Saida (imen.bensaida@yahoo.com)

*Ann. Intensive Care* 2020, **10(Suppl 1):**P-047

**Rationale:** The 2017 GOLD report represents a major revision to GOLD strategy guidelines. It brings new recommendations regarding diagnosis, severity assessment, and both pharmacologic and non-pharmacologic treatment of COPD. However, adherence to evidence-based therapeutic guidelines is often poor in low-income developing countries and represents a significant barrier to optimal management. The aim was to describe the adherence rates to GOLD 2017 guidelines in critically ill COPD patients and to identify predictors of low adherence.

**Patients and methods:** A prospective cohort study conducted from December 2017 to April 2019 in a 9-bed medical intensive care unit of Farhat Hached hospital. All adult patients admitted for AECOPD during the period of the study were included. Demographic and clinical data were recorded. Adherence to GOLD 2017 was evaluated. Univariate and multivariate regression analyses were carried out to identify factors independently associated to non-adherence to GOLD 2017 guidelines.

**Results:** Seventy-seven patients were recruited. Patients’ characteristics were : mean age, 65.5 ± 9 years; male 71 (92.2%); median duration of the disease, 6 [13–14] years; mMRC scale ≥ 2, 67 (88.2%); health insurance coverage rate, 57 (75%); pulmonologist follow up, 34 (59,6%); frequent exacerbator (≥3 exacerbations in the last year), 26 (34.2%); median exacerbations episodes, 2 [1–3]. Long-term oxygen use and home mechanical ventilation were respectively used in 10 (13.2%) and 5 (6.6%). Eight (10.5%), 14 (18.4%) and 54 (71.1%) belonged to COPD groups B, C and D, respectively. Pharmacological treatment included: SABA-ICS combination, 25 (32.9%), LABA-ICS, 10 (13.2%), LABA-LAMA, 10 (13.2%) and LAMA-LABA-ICS, 9 (11.8%). Overall adherence to 2017 GOLD guidelines treatment recommendations for the different stages of COPD was 22 (28.9%). Two patients (2.6%) were over treated and 52 (68.4%) were undertreated. Inappropriate treatment rate was 6 (75%) in Gold B, 12 (85.7%) in Gold C and 36 (66.7%) in Gold D. Univariate analysis identified two factors associated with non-adherence to GOLD 2017: the absence of pulmonologist follow-up (50% vs. 9.1%; p = 0.01) and the low income (35.2% vs. 9.1%; p = 0.021). In multivariate analysis only the lack of pulmonologist follow-up was identified as an independent risk factor associated with GOLD guidelines discrepancies (OR, 10; 95% CI [2.1–47.0]; p = 0.04).

**Conclusion:** There is a lack of adherence to GOLD 2017 guideline treatment recommendations in Tunisian COPD patients. This may lead to severe exacerbations. Discrepancies were due to the poor access of severe COPD patients to an appropriate pulmonologist follow-up.

**Compliance with ethics regulations:** Yes.

### P-048 Use of video-Airtraq in difficult intubation situation: experience of Tizi-Ouzou University Hospital

#### Yacine Benhocine

##### Tizi-Ouzou University Hospital, Tizi-Ouzou, Algeria

###### **Correspondence:** Yacine Benhocine (yacine001@yahoo.fr)

*Ann. Intensive Care* 2020, **10(Suppl 1):**P-048

**Rationale:** Since 2015, several scientific societies have proposed an algorithm to guide physicians to the most appropriate intubation method, especially in case of difficult intubation including recent devices type video laryngoscopes. Following the recommendations of the SFAR 2017, our CHU acquired the video-Airtraq in the composition of the trolley of difficult intubation. Purpose: To evaluate the efficiency of the video-Airtraq in a situation of difficult intubation in the operating theaters of Tizi-Ouzou University Hospital.

**Patients and methods:** Prospective, mono-centric study, from March 2018 to March 2019. The operating theaters concerned were: the otolaryngology block, ophthalmology, vascular and thoracic surgery, and gynecological surgery. All patients over 18 years of age were enrolled using the clinical parameters of difficult intubation (Arne score > 11), which will benefit from orotracheal intubation. The main judgment criteria were: first-pass success rate, intubation time, which is defined as the time between inserting the slide into the patient’s mouth and obtaining the capnography curve, the Cormack-Lehane score and the POGO score (percentage of opening of the glottis). Statistical analysis used SPSS software.

**Results:** A total of 62 patients were included. No cases of failure with this device were observed, the duration of intubation was on average 21.5 s (only 3 cases required more than 1 min). The Cormack-Lehane score 1 and 2 involved 55 patients (88.7%), and the POGO score greater than 50% involved 51 patients (82.25%). One case required the setting up of an LMA-Fastrach (desaturation). A case of glottic edema has been noted.

**Discussion:** This study shows a very high success rate with this technique (95.16% in the first trial and 4.83% in the second trial), in the context of a predictable difficult intubation. The video-Airtraq allows a very good visualization of laryngeal structures, a shortening of the duration of intubation, and is rarely responsible for immediate or secondary complications. All the data in the literature go in the same direction.

**Conclusion:** At the end of this work, our perspectives are to update the difficult intubation procedure, integrating the video-Airtraq into our algorithm, as well as into our difficult intubation trolley. To take into consideration the cost of this device to eventually generalize it to all our structures.

**Compliance with ethics regulations:** Yes.

### P-049 Added-value of clinical pharmacist (CP) in intensive care unit (ICU)?

#### Justine Lemtiri, Elodie Matusik, Fabien Lambiotte, Hatem Boughanmi, Emilie Herbez, Etienne Cousein, Nabil El Beki

##### Centre hospitalier de Valenciennes, Valenciennes, France

###### **Correspondence:** Justine Lemtiri (lemtiri-j@ch-valenciennes.fr)

*Ann. Intensive Care* 2020, **10(Suppl 1):**P-049

**Rationale:** In an effort to harmonize practices and to improve medication management for patients, on a polar impulse, the ICU has recruited a full-time CP since May 2017 and hospital pharmacy resident since May 2018. The objective was to define the activities and the impact of the CP.

**Patients and methods:** Our hospital has 23 ICU beds (neurosurgical [7] polyvalent [16]) and 15 beds of continuous monitoring. The activity of the CP is organized in a medical visit in the morning and in conducting projects in the afternoon. The activity is presented using a 2-years balance sheet

**Results:** The activity of pharmaceutical interventions (PI) or answers to requests from teams is shown in Table 1. The solicitations doubled the second year. The CP is involved in the conduct of internal or polar projects (set up of cooperative sedation, nutrition…), the good use of health products (relay IV/PO, infusion, crushed tablets and compatibility with gastric probe, drug incompatibilities, proton pump inhibitors…), the efficiency of the drug circuit (link with the pharmacy, reflection on the improvement of the circuit, regular meetings with nurses), medico-economic analysis of health products spending and the formalization of actions by protocolisation. He is also very involved in clinical research: patient screening, clinical study setup: BLIPIC study (Beta-lactam’s dosing In Pneumonia in ICU in patients treated by Continuous renal replacement therapy; ClinicalTrials NCT03897582) or in CANDIAREA project (Invasive infections to Candida and preemptive treatment guided by biomarkers; in progress). A satisfaction survey submitted at 6 months to nurses (12 answers/60) or to doctors/residents (13/25) reported CP competence in the accompaniment of teams (> 80%) [in medico-economical, contribution of knowledge, vigilance reflex…], relevance of information transmitted (> 85%) [administration of drugs, dosage adjustments, …] and his relationship adapted to the units (> 90%) [communication, availability].

**Conclusion:** The development of clinical pharmacy in ICU involves mastery of the specificities of ICU by the CP, requiring a learning period and relationships adapted to clinical situations and teams. Many health products projects specific to critical care are coordinated by the CP and made possible by medical and paramedical involvement. The CP appears as a vector of good use both in medical (reasoned prescription) and paramedical (good practices) with increasing solicitation of teams since his arrival. This reception has been facilitated by an innovative approach of clinical pharmacy deployment in our ICU on an impulse of the clinical pole

**Compliance with ethics regulations:** Yes.

**Table 1:** Main elements of pharmaceutical interventions (PI) analysis and solicitations

	PI	Solicitations
Number	2458	1272
Main distribution	Inappropriate way and administration: 20% Underdosing/overdosing: 16.8% Drug not indicated: 15% Non-compliance with the recommendations: 14.6% Indication not treated: 12.4%	Therapeutic strategy: 20% Antibiotherapy: 16% Dosage: 14.5% Computerized prescription: 10% Administration: 7%
Acceptance	89% (2197/2458)	–
CLEO scale impact (by the French Society of Clinical Pharmacy)	Vital/major clinical impact: 373/1764 (21%) Average clinical impact: 765/1764 (43%) Economic impact: 270/1764 (15%)	–

### P-050 Experience of young internal doctors in learning of placement of central venous lines echo guided in emergency department CHU ORAN (Algeria)

#### Soulef Bousbia, Soumia Benbernou, Nabil Ghomari, Houria Djebli

##### Faculté de medecine d’oran, Oran, Algeria

###### **Correspondence:** Soulef Bousbia (bsoulef90@gmail.com)

*Ann. Intensive Care* 2020, **10(Suppl 1):**P-050

**Rationale:** Ultrasound in resuscitation square continues to increase and be used in the realization of central line catheter (CLC). We wanted to evaluate the techniques used for the implementation of the central venous lines at the level of the medical emergencies of the CHU of ORAN ICU and see her interest in reduction of complications that can occur when this technique is performed blindly.

**Patients and methods:** This is a prospective study in ICU medical emergencies of the CHU from ORAN in August 2017 to September 2017 grouping. The approach is “in plane ‘ where the needle walks in the ultrasonic plan. 45 CLC were asked, two groups have been determined, 1 brought together 22 CLC posed by ultrasound, then 2nd 23 CLC placed over anatomical landmarks, 6 interns participated in this experience.

**Results:** In the first group: 17 CLC on the intern jugular venous and 5 subclavian, we had no failure rate pose, no complications, the time necessary to pose the line was 5 min on mono puncture. In the second group: 19 CLC on the intern jugular venous and 3 subclavian, we have one failure rate pose, 2 pneumothorax, 3 arteriel puncture and one or 2 puncture tentative, the time of pose was 7 or 8 min.

**Conclusion:** The use and training of residents must become a routine procedure for the placement of central venous catheter by ultrasound in resuscitation. However, operators must also master the pose of CVC blind to certain circumstances (emergencies material unavailable…).

**Compliance with ethics regulations:** Yes.

### P-051 Learning emergency procedures at the faculty of medicine and pharmacy in Marrakech: medical simulation model

#### Fahd Moussaid, Youssef Bouidir, Hamza Elhamzaoui, Taoufik Abouelhassan

##### Emergency Department, University Hospital Marrakech, Marrakech, MOROCCO

###### **Correspondence:** Fahd Moussaid (fahdmoussaid5@gmail.com)

*Ann. Intensive Care* 2020, **10(Suppl 1):**P-051

**Rationale:** Training in primary and secondary emergency procedures is provided in potential emergency modules, life-threatening emergencies and collective risks. The pedagogical techniques used are of the “Active” type with simulations, trial and error, questioning, analysis with practitioners… It aims to acquire knowledge by new doctors from their medical studies to the management of a person in an emergency situation. At the level of the Faculty of Medicine in Marrakech, it is intended for students in the 3rd and 6th grades as part of an integrated educational programme. Our objective was to assess the degree of satisfaction of 3rd and 6th grade students with this training and its impact on their medical practice.

**Patients and methods:** It is a study carried out through an analysis of a questionnaire distributed to students in the 3rd and 6th year of the Faculty of Medicine of Marrakech, during the period from October to December 2018. Based on its data, a cross-sectional descriptive analytical study was carried out using Excel software.

**Results:** 150 questionnaires were used for 3rd year students, and 120 questionnaires for 6th year students. We objectified a degree of satisfaction between average to good with well-defined pedagogical objectives, ordered content and objective-oriented scenarios. The causes of dissatisfaction of some students were mainly related to the limited duration of the training, and the general facilitation skills, which varied according to the trainers.

**Conclusion:** The results of our survey suggest that, despite explicitly prescriptive regulatory provisions for training in emergency procedures, the training of future general practitioners needs to be improved at the Marrakech Faculty of Medicine despite the high level of satisfaction among training students.

**Compliance with ethics regulations:** Yes.

### P-052 NEWS, Heart Rate Variability and iROX: predictor of poor outcomes in ICU patient under spontaneous ventilation?

#### Nicolas Ferrière, Laetitia Bodenes, Victoire Pateau, Julien Dolou, Erwan L’her

##### CHRU Brest, Brest, France

###### **Correspondence:** Nicolas Ferrière (nicolas.ferriere@chu-brest.fr)

*Ann. Intensive Care* 2020, **10(Suppl 1):**P-052

**Rationale:** Predicting outcomes of ICU patients remains a challenge. Predicting models such as the NEWS has been developed in the emergency department, but it has only been fewly evaluated in the ICU. Heart rate variability (HRV) reflects the autonomic nervous system response in various pathological situations and may vary according to patients’ physiological status. The ROX index, which reflects the acute respiratory failure severity, seems to be a good predictor of high-flow nasal canula failure. The aim of this study was to evaluate the potential value of NEWS, HRV and iROX (inversed ROX) as poor outcome predictors, using artificial intelligence and machine learning.

**Patients and methods:** A retrospective analysis of a prospective datawarehousing project (ReaStoc clinicalTrials identifier NCT 02893462) on ICU patients who did not require invasive ventilation. Physiological parameters were collected on admission, within a 24-h delay. NEWS, HRV (in time, frequency, and non-linear domains), and iROX were computed and integrated into the prediction model. Analysis was performed using MedCalc and Matlab machine-learning work-package.

**Results:** One hundred and twelve patients were included. Patients who died in the ICU (n = 8) had highest NEWS as compared with ICU survivors (9.0 [5.0–11.6] vs. 6.0 [5.0–7.0] respectively; p = 0.03). The iROX was higher (18.4 [15.7–19.8] vs. 9.0 [5.8–20.9], p = 0.008) and most HRV parameters also depicted higher values for ICU survivors. Considering a composite ICU prognostic outcome parameter (mortality and/or need for any form of respiratory assistance and/or an ICU LOS > median LOS), there was also a difference for NEWS, HRV and iROX (p < 0.0001). The best value to predict ICU mortality for NEWS was 8 (AUC = 0.73, p = 0.005), iROX > 9.5 (AUC = 0.76, p = 0.03) and HRV (Shannon entropy) > 2.9 (AUC = 0.74, p = 0.04). The best model to predict the need fo respiratory assistance combines iROX and HRV (SD1/SD2; AUC = 0.63, p = 0.0001). Adding Shannon entropy on this model predicts either the need for respiratory assistance and ICU survival (respectively AUC 0.62, p = 0.006 and AUC 0.72, p = 0.005).

**Conclusion:** In ICU spontaneously breathing patients, NEWS, iROX and HRV are different in between survivors and patients who died. The best model to predict the need for respiratory assistance combines iROX and HRV (SD1/SD2).

**Compliance with ethics regulations:** Yes.

**Fig. 1:**
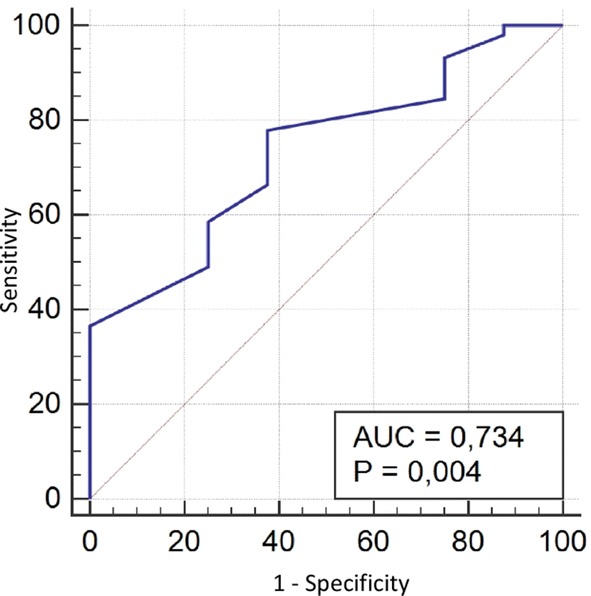
Receiver operating characteristics (ROC) curve analysis the new early warning score (NEWS) in predicting ICU mortality

### P-053 Which optimal dose of enoxaparine in burn patients

#### Sarra Dhraief, Kawther Faleh, Meriem Oueslati, Hana Fredj, Amel Mokline, Lamia Thabet, Hana Benali, Selma Abid, Lilya Debbiche, Amenallah Messaadi

##### Department of Clinical Biology, Burn and Trauma Center–Tunis, Tunisia, Ben Arous, Tunisia

###### **Correspondence:** Sarra Dhraief (dhraiefsarra@gmail.com)

*Ann. Intensive Care* 2020, **10(Suppl 1):**P-053

**Rationale:** The incidence of thromboembolic complications in burn patients may be partially due to ineffective preventive treatment. We conducted this study to determinate antiXa activity in burns using total body skin area (TBSA) and weight to achieve adequate anti factor Xa levels.

**Patients and methods:** A prospective study was conducted in burn center in Tunis during 19 months. Acute burn patients admitted to the burn center and anticipated to be non ambulatory for greater than 48 h were included. Were excluded patients with any contraindiaction of anticoagulation, and those with creatinine clearance < 30 ml/min or creatinine > 1.6 mg/dl. Patients received Enoxaparin as following: Enoxaparin dose in mg Q12Hrs = 22.8 + (3.3 × % TBSA/10) + (1.89 × (weight in kg)/10 [1]. Peak antiXa was obtained between 3 and 5 h after the third enoxaparin dose. The recommended antiXa varied between 0.2 and 0.4 U/ml.

**Results:** Ninety three burned patients were included. The mean age was 36 ± 17 years with a sex ratio of 2.33. The average TBSA was 32.7 ± 17% and the average of body weight was 72.7 ± 19.9 kg. Forty-three patients were well dosed in the first assay (46, 25%). Thirty-nine patients were under dosed in the first assay (41, 93%) and eleven patients were over dosed (11, 82%) without clinical impact. The average enoxaparin dose was 0.44 mg Q12Hrs for all patients. For the well dosed patients, anti Xa was 0.274 ± 0.05; 0.126 ± 0.06 for the under dosed patients and 0.52 for the over dosed patients.

**Conclusion:** The dosage of enoxaparin in burns taking into account the TBSA and the weight makes it possible to have an effective anti-Xa activity.

**Compliance with ethics regulations:** Not applicable.

### P-054 Outcomes in critically ill patients non admitted in the intensive care unit from the emergency department

#### Hadil Mhadhbi, Khedija Zaouche, Hamida Maghraoui, Yosra Yahya, Kamel Majed

##### Emergency Department la Rabta, Tunis, Tunisia

###### **Correspondence:** Hadil Mhadhbi (hadil.mhadhbi@gmail.com)

*Ann. Intensive Care* 2020, **10(Suppl 1):**P-054

**Rationale:** Critical care constitutes a significant and growing proportion of the practice of emergency medicine. Numerous factors can cause a delay or absence of transfer to an intensive care unit (ICU) for critically ill emergency department (ED) patients. The impact of non admittance to the ICU is not well studied. We aimed to study the outcomes of critically ill patients not transferred to ICU and remaining at the ED.

**Patients and methods:** We conducted a prospective observational study which included all critically ill patients older than 18 years admitted to the ED. The period of study was 1 year. Data of all patients were collected. Main outcomes were length of stay at the ED and in-hospital mortality.

**Results:** During the study period, a total of 100 patients with a mean age of 67 ± 12 years were included. 60% were male. The main diagnoses were acute respiratory failure in 34%, septic shock in 22% of cases. Among hospital survivors, the median hospital length of stay was 50 h. In-hospital mortality was 19%. Higher SOFA score at admission, advanced age and male gender were associated with lower hospital survival.

**Conclusion:** Critically ill emergency department patients not admitted to the intensive care unit had longer hospital stay period and higher in-hospital mortality. These results unveil the need to identify the factors associated with non transfer to the ICU as well as specific determinants of adverse outcomes.

**Compliance with ethics regulations:** Yes.

### P-055 SIRS is still useful in the sepsis-3 era

#### Abderrahim Achouri, Hadil Mhadhbi, Khedija Zaouche, Hamida Maghraoui, Radhia Boubaker, Kamel Majed

##### University Hospital Center of Rabta in Tunis, Tunis, Tunisia

###### **Correspondence:** Abderrahim Achouri (achouryabderrahim@gmail.com)

*Ann. Intensive Care* 2020, **10(Suppl 1):**P-055

**Rationale:** Sepsis is known for its important mortality in critically ill patients. The last guidelines defined sepsis as life threatening organ dysfunction. It rejected the concept of systemic inflammatory response syndrome (SIRS) associated to suspected or confirmed infection, and considered the concept of dysregulated response to infection. Actual guidelines recommend the quick sequential organ failure assessment score (qSOFA) to identify patients with sepsis especially when outside intensive care unit. Thus, outcomes have mainly to judge the value of SIRS in the sepsis-3 era. The purpose of our study was to compare whereas qSOFA score or the SIRS criterion are superior to predict in-hospital mortality, shock and mechanical ventilation use in sepsis.

**Patients and methods:** Our study includes patients in whom the sepsis-3 definition is met. Therefore, this inclusion was retrospectively performed throughout emergency department (ED) admission cases for clinically suspected infection.

**Results:** We collected 93 patients admitted to ED for sepsis. Mean age was 65 years ± 14 with bornes of 24 and 92. Men were 57% of the patients. Death occurs in 29.6% of patients, sepstic shock in 25% and the use of mechanical ventilation in 5.6%. qSOFA ≥ 2 has a significant association with in-hospital mortality (p < 0.001) but not SIRS ≥ 2 (0.208). Neither qSOFA ≥ 2 nor SIRS ≥ 2 has association with the use of mechanical ventilation (p = 0.104 vs. p = 1). Whereas, both have a significant association for prediction of septic shock.

**Discussion:** The absolute sensitivity and negative predictive value in our study can be explained by the small size of our sample. This needs confirmation with literature data about the fact that SIRS criterion are superior in term of sensitivity and NPV than qSOFA to predict septic shock. Despite the weak odds ratio (OR) of SIRS before that of qSOFA and the poor specificity and Positive predictive value (PPV), we can conclude that SIRS according to its sensitivity and NPV, seems to persist useful in the sepsis-3 era as a reliable prognostic tool in the ED. This may need more large studies for confirmation.

**Conclusion:** Despite SIRS has no significant association with mortality in sepsis, it has largely higher sensitivity and superior NPV to predict septic shock than qSOFA in ED.

**Compliance with ethics regulations:** Yes.

**Table 1:** Comparison between qSOFA and SIRS in prediction of septic shock

	qSOFA ≥ 2	SIRS ≥ 2
Septic shock (%); p value	24% (< 0.001)	29% (0.02)
Odds ratio [95% IC]	15.143 [4.191–54.715]	1.409 [1.237–1.605]
AUC [95% IC]	0.772 [0.676–0.868]	0.593 [0.478–0.707]
Sensitivity	0.89	1
Specificity	0.65	0.19
Positive predictive value (PPV)	0.46	0.29
Negative predictive value (NPV)	0.94	1

### P-056 Predictors of mortality in septic shock in intensive care

#### Mohamed Lazraq, Sabah Benhamza, Youssef Miloudi, Abdelhak Bensaid, Najib Elharrar

##### Hôpital 20 aout 1953, CHU ibn rochd, Casablanca, Morocco

###### **Correspondence:** Mohamed Lazraq (mohamed_lz@hotmail.com)

*Ann. Intensive Care* 2020, **10(Suppl 1):**P-056

**Rationale:** The mortality from septic shock in intensive care remains high despite the evolution of diagnostic and therapeutic techniques. Our study aimed to determine the predictive factors of mortality in our patients.

**Patients and methods:** Retrospective study over 2 years in the intensive care unit of the hospital 20 August. All patients with septic shock were included. A p value < 0.05 was considered significant.

**Results:** 81 patients were collected. The age ranged from 11 to 94 years old. The average duration of hospitalization in pre-intensive care was 5 days. The reasons for admission: (febrile respiratory distress: 31% of cases, polytrauma: 14% and 23% for sepsis), the most frequent infections: pulmonary (33%) and blood (32%). 38% received prior antibiotic therapy and 35% were immunocompromised. The overall mortality was 48%. The analytical study of the data shows that the age, the length of stay before admission in intensive care and that in intensive care, fever, hypothermia, slimming, hypotension, collapse, failures (respiratory, hematological, renal, hepatic and neurological) and the use of catecholamines are correlated with mortality, whereas sex, chest pain, tachycardia or bradycardia and mottling are not predictive of mortality.

**Conclusion:** Despite improved techniques for the diagnosis and treatment of patients with septic shock, mortality remains high, especially in the presence of certain risk factors, hence the value of prevention in immunocompromised patients and the reduction in their length of stay in a hospital setting.

**Compliance with ethics regulations:** Yes.

### P-057 Molecular characterization of carbapenemase-producing Enterbacteriaceae in trauma and burn center in Tunisia

#### Mehdi Gaddas, Sarra Dhraief, Karim Mechri, Imen Jami, Amenallah Messaadi, Lamia Thabet

##### ^1^Centre de Traumatologie et des Grands Brûlés de Ben Arous-Laboratoire de biologie médicale et banque du sang, Ben Arous, Tunisia; ^2^Centre de Traumatologie et des Grands Brûlés de Ben Arous-Service de réanimation des brûlés, Ben Arous, Tunisia

###### **Correspondence:** Mehdi Gaddas (gaddasmehdi@icloud.com)

*Ann. Intensive Care* 2020, **10(Suppl 1):**P-057

**Rationale:** The emergence of carbapenemase-producing Enterbacteriaceae (CPE) is an increasingly serious worldwide problem associated with high rate of therapeutic failure and mortality. Thus, early detection of CPE and rapid application of infection control measures is of a paramount importance. We conducted a prospective study to characterize the molecular mechanisms and to determine the antimicrobial susceptibility profiles of CPE isolated in trauma and burn center in Tunisia.

**Patients and methods:** We examinated 148 strains with reduced susceptibility to carbapenems among 978 Enterobacteriaceae clinical isolated collected between January 2018 and December 2018. Sixty-three strains were selected for the study (one strain per species and per patient). The susceptibility of each strain was determined for a range of antibiotics involved, according to CA-SFM guidelines. Minimum inhibitory concentration (MIC) of carbapenem were determined using the E-test^®^ method (bioMérieux). Multiplex real-time PCR was performed with Cepheid’s GeneXpert^®^, allowing detection of the most prevalent carbapenemase gene families (blaVIM, blaNDM, blaIMP, blaOXA-48 and blaKPC).

**Results:** Of the 63 selected bacteria, *Klebsiella pneumoniae* was the main isolated one (74.6%) followed by *Enterobacter cloacae* (11.1%). Selected bacteria were mainly isolated from burn intensive care unit (74.6%). PCR was positive for 57 isolates (90.5%). Thirty-one bacteria (54.4%) expressed the blaNDM gene. The blaoxa-48 gene was found in 15 strains (26.3%) and 11 strains carried both genes. Of the 57 CPE, 89.5% revealed ertapenem MIC > 1 mg/l, whereas only 7% showed imipenem MIC > 2 mg/l. The antibiotics showing the highest resistant rates were cefotaxime (100%), piperacillin-tazobactam (94.7%), aztreonam (93.9%), ciprofloxacin (89.4%) and amikacin (52.6%). The most active agents were colistin, tigecycline (excepting with Proteae) and fosfomycin with 94.7%, 61.4% and 91.2% of susceptibility, respectively.

**Conclusion:** The spread of CPE is an alarming problem in our center. Among carbapenemase encoding gene, the blaNDM was predominant. Detection of CPE by GeneXpert^®^ carba-R was established in the center with a whole protocol of prevention.

**Compliance with ethics regulations:** Yes.

### P-058 Proteus mirabilis infections in intensive care unit

#### Sabrine Bradai, Karama Bouchaala, Fatma Zouari, Basma Mnif, Kamilia Chtara, Mounir Bouaziz

##### University of Sfax, Sfax, Tunisia

###### **Correspondence:** Sabrine Bradai (sabrine.bradai2@gmail.com)

*Ann. Intensive Care* 2020, **10(Suppl 1):**P-058

**Rationale:** Proteus mirabilis, a Gram-negative rod-shaped bacterium most noted for its swarming motility and urease activity, frequently causes nosocomial infections especially catheter-associated urinary tract infections. The aim of our study was to point out the incidence of Proteus mirabilis infection in ICU patients, its clinical presentation and course.

**Patients and methods:** We conducted a retrospective descriptive study. All patients hospitalized in the ICU of our University Hospital who developed Proteus Mirabilis infections from January 01, 2017 to June 30, 2019 were included.

**Results:** During the study period, 36 patients were included. The mean age was 49.4 ± 19.5 years. Sex ratio (M/F) was 3.5. Trauma was the major cause of hospitalization in 20 cases (55.6%). Length of hospital stay prior to ICU admission was 5 ± 9.4 days. SAPSII was 44.9 ± 13.3, mean SOFA was 10.4 ± 3.1 and mean APACHE II was 18.1 ± 5.4. All patients required invasive mechanical ventilation, had a central venous catheter (CVC) and indwelling urinary catheter in place. Nine patients (25%) presented acute kidney failure and 6 (16.7%) needed dialysis. Before the isolation of *P. mirabilis*, 10 patients (27.8%) hadn’t any infections and 5 patients (13.9%) didn’t received any antibiotics. Concerning the other 31 patients, antibiotics prescribed were: amoxicillin/clavulanic acid in 17 patients (47.2%), carbapenems in 17 patients (47.2%), aminosids in 13 patients (36.1%), glycopeptides in 10 patients (27.8%), colistin in 9 patients (25%), fluoroquinolones in 7 patients (19.4%), cephalosporins in 6 patients (16.6%), tigecycline in 4 patients (11.1%). The most common infection site of P.mirabilis was the urinary tract in 22 patients (61.1%). After *P. mirabilis* infection, 18 patients (50%) had septic shock. Antibiotics used to treat *P. mirabilis* infection were resumed in Table 1. The mean length of ICU stay was 38.4 ± 18.4 days. Out of the 36 included patients, 9 patients died (the mortality rate was 25%). Death was not related to *P. mirabilis* infections.

**Conclusion:** *P. mirabilis* is among the leading bacteria responsible for nosocomial infections in ICU. They are emerging highly drug resistant pathogens whose incidence is rapidly increasing in ICU. So that, it early identification with in vitro testing is of paramount importance to the success of infection control efforts.

**Compliance with ethics regulations:** Not applicable.

**Table 1:** antibiotics used to treat *Proteus mirabilis* infections

Empiric antibiotic	Number	Percentage (%)	Adapted antibiotic	Number	Percentage (%)
Imipenem Colistin Gentamicin Cefotaxime	8 1 1 3	22.2 2.8 2.8 8.3	Imipenem Cefotaxime Colistin Piperacillin/tazobactam Amikacin Gentamycin	2 2 2 2 1 1	5.6 5.6 5.6 5.6 2.8 2.8
Imipenem + gentamicin Imipenem + amikacin Imipenem + colistin Tigecycline + colistin Amoxicillin/clavulanic acid + amikacin Ciprofloxacin + colistin Ciprofloxacin + rifampicin Imipenem + bactrim Cefotaxime + metronidazol Cefotaxime + ciprofloxacin	4 3 2 2 1 1 1 1 1 1	11.1 8.3 5.6 5.6 2.8 2.8 2.8 2.8 2.8 2.8	Imipenem + gentamicin Imipenem +amikacin Imipenem + tygecycline Imipenem + colistin Tygecycline + colistin Ciprofloxacin + gentamicin Ciprofloxacin + tygecycline Ciprofloxacin + imipenem Cefotaxime + gentamicin Colistine + Amikacin Ertapenem + gentamycin Piperacillin/tazobactam + fosfomycin	5 4 2 1 1 1 1 1 1 1 1 1	13.9 11.1 8.3 5.6 5.6 5.6 2.8 2.8 2.8 2.8 2.8 2.8
Colistin + rifampicin + Ciprofloxacin Colistin + vancomycin + metronidazol Imipenem + gentamicin + tygecycline	1 1 1	2.8 2.8 2.8	Ceftazidime + amikacin + colistine Piperacillin/tazobactam + ciprofloxacin + colistin Piperacillin/tazobactam + Imipenem + levofloxacine	1 1 1	2.8 2.8 2.8
No use of empiric antibiotic	3	8.3	*P. mirabilis* not treated	3	8.3

### P-059 Eosinopenia in sepsis

#### Kaoutar El Fakhr, Habibou Hassane Mahamane Rabiou, Abdeljabbar Marhfour, Boubaker Charra

##### CHU Ibn rochd, Casablanca, Morocco

###### **Correspondence:** Kaoutar El Fakhr (kaoutarelfakhr2013@gmail.com)

*Ann. Intensive Care* 2020, **10(Suppl 1):**P-059

**Rationale:** Eosinopenia is a reliable marker of sepsis. The aim of this study was to test the value of eosinopenia in the diagnosis of sepsis.

**Patients and methods:** This prospective study included 30 patients with sepsis documented on a bacteriological specimen on admission or during hospitalization in the medical resuscitation department of Ibn Rochd Casablanca University Hospital, from January 2018 to April 2019.

**Results:** Comparison between infected and non-infected studied patients was statistically significant as regard variables of SOFA score, APACHE II score at admission, and eosinophil count at admission. Multivariate regression analysis showed statistically significant differences and was independent predictors for infection as follow: total leucocytic count, eosinophil count at admission and SOFA score. The AUC for eosinophil count to predict was 95% with optimal cut off value was 50 cells/mm^3^ with for admission diagnoses. Eight patients (26.7%) were in renal impairment, with white blood cell count > 10,000/mm^3^, eosinophils counts was under 50 cells/mm^3^, CRP > 40 mg/L, PCT > 100 ng/ml. 22 patients (73.3%) had correct renal function, 6 patients (20%) had whiteblood cell count > 10,000/mm^3^ eosinophils counts was under 50 cells/mm^3^ CRP > 40 mg/L PCT > 0.5 ng/ml.

**Conclusion:** The result of the present study revealed that eosinophil counts ˂50 cells/mm^3^ at admission time was a predictor for diagnosis of sepsis in critically ill patients. However, eosinophil counts at admission time were not a specific indicator of mortality.

**Compliance with ethics regulations:** Yes.

### P-060 Impact of multiplex PCR on antibiotic prescription in ICU patients with community acquired-pneumonia

#### Marion Giry, Déborah Boyer, Manuel Etienne, Dorothée Carpentier, Steven Grange, Christophe Girault, Fabienne Tamion, Gaetan Beduneau

##### ^1^Réanimation médicale, CHU de Rouen, Rouen, France; ^2^Maladies infectieuses et tropicales, CHU de Rouen, Rouen, France

###### **Correspondence:** Marion Giry (marion.giry7@gmail.com)

*Ann. Intensive Care* 2020, **10(Suppl 1):**P-060

**Rationale:** Pneumonia is the most frequent community-acquired infection responsible for ICU admission. Multiplex PCR enables early diagnosis of viral infection in daily practice. Few series have described the impact of this technique on the antibiotic duration in ICU. Our principal objective was to evaluate the impact of multiplex PCR on the duration of antibiotic therapy in ICU patients with community-acquired pneumonia (CAP). Our secondary objectives were to evaluate the practices and the respect of French recommendations in the prescription of antibiotics for ICU patient with CAP and to evaluate the potential undesirable effects of overprescribing antibiotics.

**Patients and methods:** Retrospective analysis of the consecutive viral multiplex PCR (Eplex™, Genmark) between November 2016 and October 2017 in a French 21 bed medical ICU admitting around 1000 patients per year. Patients’ nasopharynx was sampled within 72 h following their ICU admission. We identified patient with a viral CAP (group 1) and patient with other microbiological finding (bacterial, mixed, no finding) CAP (group 2). Comparisons were made with non-parametric Welch t-test and Fischer’s exact tests.

**Results:** 223 patients were sampled, of whom 75 had CAP, 27 aspiration or opportunistic pneumonia, 25 non-pulmonary infections, 21 exacerbations of chronic lung disease, 16 pulmonary edema, 10 bronchitis, 6 pleural empyema and 43 other diagnoses. Patients with CAP had the following characteristics: age 60 ± 17 years, male sex 52%, SAPS 2 score 41 ± 19, ICU length of stay 9.1 ± 8.7 days, mortality 15%. There were 16 patients (21%) in the group 1 and 59 patients (79%) in the group 2. The main bacteria were *S. pneumonia* (40.4%) and H. influenza (9.3%). The main viruses were Influenzae A (26.5%), Rhinovirus (23.5%) and Parainfluenzae (17.6%). The antibiotic duration was shorter in group 1 than in group 2 (4.1 days vs. 6.4 days p = 0.007) for the ICU stay. No statistical difference was found on the antibiotic duration for the whole hospitalization (8.1 days vs. 11.1 days, p = 0.07). French recommendations (duration of antibiotic treatment, choice of initial therapy, de-escalation at 48–72 h) were followed in 36% cases. 48% of patients had an antibiotic treatment for more than 7 days. Adverse events were found in 5% of patients.

**Conclusion:** In our study, respiratory viruses were present in 45% of CAP. Their diagnosis with multiplex PCR allows shorter ICU antibiotic treatment duration, no statistical difference was found for the whole hospitalization. A multicentric prospective study needs to validate these results.

**Compliance with ethics regulations:** Yes.

### P-061 Discrepancies exist between medical and paramedical ICU health workers’ opinions on a mandatory vaccination for influenza

#### Renaud Prével^1^, Raphaël Enaud^2^, Erwan Begot^1^, Alexandre Massri^3^, Chloé Gisbert-Mora^4^, Patrick Berger^5^, Cédric Carrié^6^, Hugues De Courson^7^, Alexandre Boyer^1^, Didier Gruson^1^

##### ^1^CHU Bordeaux, Medical Intensive Care Unit, Bordeaux, France; ^2^Inserm U1045 Centre de Recherche Cardio-thoracique, Bordeaux, France; ^3^Intensive Care Unit, CH Francois Mitterrand, Pau, France; ^4^Intensive Care Unit, CH Cote Basque, Bayonne, France; ^5^Inserm U1045 Centre de Recherche Cardio-thoracique, Bordeaux, France; ^6^CHU Bordeaux, Surgical and Trauma Intensive Care Unit, Anesthesiology and Critical Care Department, Bordeaux, France; ^7^CHU Bordeaux, Neuro Intensive Care Unit, Anesthesiology and Critical Care Department, Bordeaux, France

###### **Correspondence:** Renaud Prével (renaud.prevel@hotmail.fr)

*Ann. Intensive Care* 2020, **10(Suppl 1):**P-061

**Rationale:** Influenza is a potential lethal disease causing dozens of thousands excess deaths per year both in Europe and in the United states. Besides hygiene procedures, vaccination is a cornerstone of influenza prevention and guidelines recommend for vaccination among health workers (HW), especially if they are in close contact with frail people. Despite these recommendations, the vaccination coverage is low among health workers both in Europe and in the US. The relevance of a mandatory vaccination for health workers is currently a hot topic but data are scarce regarding intensive care unit health workers’ opinion.

**Patients and methods:** Health workers from 2 medical, 6 surgical and 2 polyvalent ICUs received a link to the electronic record of the survey.

**Results:** Among the 10 ICUs, 1637 ICU health workers (HW) (medical: 185 and paramedical: 1452) were questioned. Three hundred and forty-one ICU (21%) answered, 107 (58%) medical health workers (MHW) and 234 (16%) paramedical health workers (PHW) (p < 0.0001). Among MHW 94/107 (88%) were vaccinated vs only 119/234 (51%) PHW (p < 0.0001). Discrepancies exist between medical and paramedical ICU health workers’ opinions and beliefs about vaccination for influenza and its acceptance. Medical health workers were more prone to consider influenza as a potentially lethal disease occurring not only among frail people but also in healthy people, to consider the vaccine efficient and safe. To agree with “Vaccination for influenza is mostly related with gain for pharmaceutical industry” (OR: 11 [2.3–50]) and to disagree with “The risk of Guillain-Barré syndrome is higher after an episode of influenza than after vaccination for influenza” (OR: 4.2 [1.2–14]) were independently associated to the disagreement with a mandatory vaccination for ICU HW.

**Conclusion:** Vaccination for influenza should be strongly recommended as a tool of individual protection for ICU health workers as for general population. As confidence in vaccine efficacy and concerns about vaccine side-effects impact the vaccination rate, objective information should be provided to ICU health workers about the efficacy and the side effects of vaccination for influenza.

**Compliance with ethics regulations:** Yes.

### P-062 Predictive mortality factors of severe influenza A in intensive care: experience of a tunisian referral center

#### Amira Jamoussi^1^, Samia Ayed^1^, Hamdi Doghri^1^, Takoua Merhabene^1^, Amine Slim^2^, Jalila Ben Khelil^1^, Mohamed Besbes^1^

##### ^1^Medical Intensive Care Unit, Abderrahmen Mami Pneumology Hospital, Ariana, Tunis, Tunisia; ^2^Virology Unit, Microbiology Laboratory, National Influenza Centre, Charles Nicolle’s Hospital, Tunis, Tunisia

###### **Correspondence:** Amira Jamoussi (dr.amira.jamoussi@gmail.com)

*Ann. Intensive Care* 2020, **10(Suppl 1):**P-062

**Rationale:** Influenza A virus infection is a contagious acute respiratory infection with possible severe clinical form. In Tunisia, surveillance of influenzae was initiated in 1989 and our unit is referral center of surveillance of severe cases since 2009. The aim of this study was to describe the epidemiological and clinical aspects of influenza A, and to determine independent predictive factors of ICU mortality.

**Patients and methods:** It was an observational cohort study over an 11-year period (June 2009–June 2019). We prospectively collected data (demographic, clinical and biological data, evolving features) of all consecutive patients diagnosed with influenza A. Multivariate analysis of the predictive factors of ICU mortality was realized.

**Results:** During the study period, 120 patients with influenza A were admitted, 14 (11.7%) were pregnant women. Mean age was 48 ± 16 years and a sex-ratio of 1.14. The mean scores of SAPS II, APACHE II and SOFA were respectively 30, 12 and 4.5. A history of hypertension, diabetes, chronic obstructive pulmonary disease and obesity were recorded in respectively 25, 20.8, 12.5 and 7.5% of cases. Only 7 (5.8%) patients were vaccinated. Acute respiratory failure was the main reason for admission in 115 patients (95.8%). Mean first PaO_2_/FiO_2_ was 181 ± 100 mmHg [45–457]. Acute respiratory distress syndrome (ARDS) was diagnosed in 82 patients, it was severe (n = 44; 54%), moderate (n = 27; 33%) and mild (n = 11; 13%). At admission, lymphopenia was present in 86 patients (72%) and rhabdomyolysis in 19 (15.8%). Virus strains identified with PCR were H1N1 pdm09 (84.2%) and H3N2 (15.8%). A Co-infection with bacteria was documented in 16 cases (13.3%) and aspergillosis in 3 cases. The most frequent complications were acute kidney injury (n = 50), shock (n = 48) and hospital-acquired infections (n = 46). Antiviral therapy oseltamivir was prescribed in 88 patients. Non-invasive ventilation (NIV) was used in 72 patients with success in 35 cases (48.6%). Endotracheal intubation was performed in 59 patients (37 after NIV failure). Mean ICU length of stay was 12 ± 12.8 days and the overall ICU mortality was 31.6%. Independent predictive factors of ICU mortality were: severe ARDS (OR = 4.7; IC95% [1–20.9]; p = 0.040), acute kidney failure (OR = 5.28; IC95% [1.2–23.3]; p < 0.001) and shock (OR = 29; IC95% [5.3–157.5]; p < 0.001). An age under 50 years was protective against ICU mortality (OR = 0.129; IC95% [0.027–0.606]; p = 0.009).

**Conclusion:** Influenza A in ICU characterized by high morbi-mortality, especially in patients with severe ARDS or shock.

**Compliance with ethics regulations:** Yes.

### P-063 Antibiotherapy of peritonitis in intensive care: impact of the 2015 SFAR recommendations and interest of an adapted antibiotherapy

#### Adrien Krings, Hugues Georges, Pierre-Yves Delannoy, Olivier Leroy

##### Centre hospitalier Gustave Dron, Tourcoing, France

###### **Correspondence:** Adrien Krings (adrienkrings@yahoo.fr)

*Ann. Intensive Care* 2020, **10(Suppl 1):**P-063

**Rationale:** Intra-abdominal infections are a major cause of morbidity and mortality. SFAR recommendations on this topic were published in February 2015. The purpose of this work was to evaluate whether our antibiotic therapy was adequate for these recommendations and whether they were adapted to our unit. The secondary objectives were to look for different risk factors for mortality, to evaluate the impact of inappropriate antibiotic therapy, to evaluate the relevance of carbapenem prescription.

**Patients and methods:** This is a single-center retrospective observational study of secondary peritonitis in the Tourcoing intensive care unit. For each peritonitis, the epidemiological data and the co-morbidities of the patients were collected. Bacteriology and anti-infectious therapies were described to determine the rates of adaptation of our antibiotic therapy and that recommended by SFAR. The adequacy of our treatments to the recommendations was also quantifiable. The description of the stay, the occurrence of a death was specified.

**Results:** 131 peritonitis were included. The rate of adaptation of the SFAR antibiotic therapy was 80%. The rate of adaptation of our antibiotic therapy was 73% and its adequacy rate of 24%. The main differences in prescriptions concerned over-prescription of antifungals, molecule against gram positive bacillus and a sub-prescription of aminoglycosides and beta-lactams, in particular carbapenems. The different mortality risk factors found were SOFA score > 8 (OR 5.4 95% CI 2.15–13.7), the Charlson score > 3 (OR 5.2 95% CI 1.52–17.9), the hollow organ perforation (OR 4.2 95% CI 1.63–10.59). A comparison of the appropriate or not antibiotic groups did not reveal a significant difference in mortality, number of surgical revision and length of stay. In 55% of nosocomial peritonitis, antibiotic therapy with carbapenem was recommended. After recovery of microbiological data, it was only necessary for 7.7% of cases.

**Conclusion:** Our work showed a low rate of compliance with SFAR recommendations. These recommendations are applicable to our service by providing a particular reflection for fungal infections. Our study does not show a correlation between mortality and inadequate antibiotic therapy, surgery remaining the major treatment.

**Compliance with ethics regulations:** Yes.

### P-064 Epidemiological profile and antibiotic susceptibility of *Acinetobacter baumannii* isolates in a trauma and burn center in Tunisia

#### Emna Hammami, Sarra Dhraief, Mehdi Gaddas, Sonia Ben Behi, Amenallah Messaadi, Lamia Thabet

##### Centre de Traumatologie et des Grands Brûlés de Ben Arous-Laboratoire de biologie médicale et banque du sang, Ben Arous, Tunisia

###### **Correspondence:** Emna Hammami (emnahammami1993@gmail.com)

*Ann. Intensive Care* 2020, **10(Suppl 1):**P-064

**Rationale:** *Acinetobacter baumannii* is a gram-negative opportunistic bacteria that has gained several drug resistance mechanisms over the last decades. Analysis of *A. baumanii’s* resistance profile helps to establish a prompt control and a prevention program. The aim of this study was to evaluate the epidemiology and antimicrobial resistance of *A. baumannii* isolates in a trauma and burn center in Tunisia.

**Patients and methods:** Retrospectively, we studied all strains of *Acinetobacter baumannii* isolated over a 7-year period (from January 2012 to December 2018). Conventional methods were used for identification. Antimicrobial susceptibility testing was performed with the disk diffusion method, and susceptibility results were interpreted using clinical breakpoints according to CA-SFM guidelines. Data were analyzed using the SIR-system. Minimum inhibitory concentration (MIC) of colistin was determined using the E-test^®^ method (bioMérieux), then using the EUCAST broth micro-dilution method (UMIC, Biocentric^®^) since May 2017.

**Results:** During the study period, 1248 non-repetitive strains of *Acinetobacter baumannii* were isolated representing 9.9% of all isolates, 14% of gram-negative bacilli (GNB) and 40.6% of non-fermenting GNB. In our center, infections due to *A. baumannii* were endemic with epidemic peaks. *A. baumannii* was mainly isolated from burn intensive care unit (67%) and anesthesiology department (22.6%). The most frequent sites of isolation were blood cultures (34.3%), catheters (20%), respiratory specimens (12.5%) and skin samples (10%). The survey of antibiotic susceptibility showed high percentages of resistance to the different antibiotics: 84% to ceftazidime, 93% to imipenem, 86% to amikacin and 91.5% to ciprofloxacin. From 2012 to 2018, the imipenem resistance rate was stable but high (over 90% of resistant strains). Amikacin resistance rate was stable all over the study period. Whereas ceftazidime resistance rate was high and fluctuating (57% in 2016, 89% in 2013 and 2017). A. baumannii strains gained resistance to ciprofloxacin over the years (from 86% in 2012, 92.3% in 2018). Twenty-two strains were resistant to colistin. These strains were isolated mainly from burn patients (72.7%).

**Conclusion:** The dissemination of *A. baumannii* multidrug-resistant strains in our center must be contained by the implementation of strict isolation methods and better hygienic procedures.

**Compliance with ethics regulations:** Yes.

### P-065 Multicentric satisfaction survey of aScope Bronchosampler™, a new sampling accessory for aScope™4 Broncho

#### Emmanuel Novy^1^, Claude Meistelman^1^, Clement Fournier^2^, Gilles Dhonneur^3^, Julien Pottecher^4^

##### ^1^CHRU NANCY, Vandoeuvre Les Nancy, France; ^2^CHRU Lille, Lille, France; ^3^Institut Curie, Paris, France; ^4^HUS Strasbourg, Strasbourg, France

###### **Correspondence:** Emmanuel Novy (e.novy@chru-nancy.fr)

*Ann. Intensive Care* 2020, **10(Suppl 1):**P-065

**Rationale:** aScope BronchoSampler is a small sterile kit to be clicked on aScope 4 Broncho simplifying and securing the sampling technique using a closed process (no suction tube switch). This prospective multicentric observational satisfaction survey had for main objective to collect the user point of view regarding BronchoSampler especially its functionality in clinical practice, such as bronchial sampling in ICU Units/Pulmonology Departments with aScope 4 Broncho versus standard sampling technique, in order to assess its potential adoption.

**Patients and methods:** BronchoSampler was evaluated in 4 hospitals (Lille, Nancy, Strasbourg, Curie Institute) for 4 months. Ambu provided the units free of charge to the different sites. 48 evaluation forms have been collected and consolidated involving 23 operators already using aScope 4 Broncho in their practice. BronchoSampler regardless of the item, was evaluated in comparison to the standard sampling method used by the operators. Results are expressed in absolute value and percentage; the sum might differ from 48 due to lack of answer from evaluator.

**Results:** BronchoSampler was mainly used for the following procedures: Bronchial Wash—BW: 8 (17%) Bronchial Alveolar Lavage—BAL: 36 (75%). Usually, those procedures were done as follows: wall suction/specimen trap: 40 (84%), manual pull/syringe: 2 (4%), material preparation traditionally requires two dedicated persons 32 (67%) versus one operator only for BronchoSampler usage 27 (56%). It frees assistant time and enable the clinician to perform the sampling alone more often. Details of the different evaluated items are listed in the grid. Sampling duration is also reduced, improving workflow. Evaluators consider that BronchoSampler rationalizes the cumbersome sampling process and that the closed system design reduces the risk of losing sample or sample contamination. The set-up, the suction capacity, the sampling quality and quantity have all been evaluated better or far better than that usually observed with usual sampling techniques and devices. Finally, 36 (75%) of users prefer BronchoSampler to commonly used method.

**Conclusion:** This satisfaction survey shows that with its simple but revolutionary design, BronchoSampler brings a real effective benefit in sampling procedure enabling the clinician to perform it alone, and 39 (81%) of the survey evaluators consider that BronchoSampler should replace their current practice.

**Compliance with ethics regulations:** Yes.

**Table 1:** Results of the different questions asked to the users in comparison to usual sampling techniques and devices. Number (%)

In comparison to existing method how do you rate aScope BronchoSampler	−	−	Equal/acceptable	+	++
Device assembly	0 (0%)	2 (4%)	5 (11%)	15 (31%)	26 (54%)
Suction capacity	0 (0%)	3 (6%)	19 (40%)	16 (33%)	10 (21%)
Sampling quality	0 (0%)	1 (2%)	19 (40%)	17 (35%)	11 (23%)
Sampling volume	0 (0%)	3 (6%)	23 (48%)	13 (27%)	7 (15%)
Sampling process simplification	1 (2%)	5 (11%)	2 (4%)	25 (52%)	15 (31%)
Reducing risk of loss or contamination sample	0 (0%)	6 (13%)	3 (6%)	25 (52%)	14 (29%)
Overall perception: the quality and functionality	0 (0%)	0 (0%)	8 (17%)	19 (39%)	21 (44%)

### P-066 Epidemiological profile and antibiotic susceptibility of *Staphylococcus aureus* isolates in a trauma and burn center in Tunisia

#### Karim Mechri^1^, Sarra Dhraief^1^, Hana Fredj^2^, Kawther Faleh^2^, Amenallah Messaadi^2^, Lamia Thabet^1^

##### ^1^Centre de Traumatologie et des Grands Brûlés de Ben Arous-Laboratoire de biologie médicale et banque du sang, Ben Arous, Tunisia; ^2^Centre de Traumatologie et des Grands Brûlés de Ben Arous-Service de réanimation des brûlés, Ben Arous, Tunisia

###### **Correspondence:** Karim Mechri (karimmechri91@gmail.com)

*Ann. Intensive Care* 2020, **10(Suppl 1):**P-066

**Rationale:** *Staphylococcus aureus* is one of the main bacteria involved in nosocomial infections. The spread of methicillin-resistant *S. aureus* (MSRA) has led to considerable difficulties in the treatment of infections due to this pathogen.

**Patients and methods:** The aim of our study was to evaluate epidemiological profile and antibiotic resistance of *S. aureus* isolates in a trauma and burn center in Tunisia. Retrospectively, we studied all strains of *S. aureus* isolated over a 7-year period (from January 2012 to December 2018). Conventional methods were used for identification. Antimicrobial susceptibility testing was performed with disk diffusion method and susceptibility results were interpreted using clinical breakpoints according to CA-SFM guidelines. Data were analyzed using the SIR-system.

**Results:** During study period, 1875 non-repetitive strains of *S. aureus* were isolated, representing 14.8% of all isolates. *S. aureus* was mainly isolated from burn intensive care unit (ICU) (56%), orthopedics (14.4%) and anesthesiology department (12.3%). The most frequent sites of isolation were blood cultures (35.8%) and skin samples (24.4%). The survey of antibiotic susceptibilty showed that MRSA rate was variable depending on the ward involved : The highest rates were observed in burn ICU (61%) and anesthesiology department (48.7%). The lowest rate was observed in orthopedics (22.4%). The overall resistance to methicillin was 47.5%. Evolution of MRSA rate was marked by a decrease from 55% in 2012 to 30.2% in 2018. Regarding other families of antibiotics, the resistance rates have also decreased during the study period: from 53.6% in 2012 to 25.3% in 2018 for gentamicin, from 45.3% and 16.7% in 2012 to 11% and 1.7% in 2018 for erythromycin and clindamycin, respectively, and from 44% in 2012 to 21.8% in 2018 concerning ciprofloxacin. Resistance to tigecycline fell from 20.5% in 2012 to 0.52% in 2018. Resistance to linezolid was rare. It concerned only one strain. All strains were susceptible to glycopeptides.

**Conclusion:** The decrease in MRSA rate and other associated resistances could be explained by a strengthening of hygiene measures and a rationalization of antibiotics use in our center.

**Compliance with ethics regulations:** Yes.

### P-067 Interest of procalcitonin in intensive care (about 120 cases)

#### Amine Raja, Mohamed Elaiassi, Boubaker Charra

##### Medical Resuscitation Department of the University Hospital of Casablanca, Casablanca, Morocco

###### **Correspondence:** Amine Raja (raja.amine2@gmail.com)

*Ann. Intensive Care* 2020, **10(Suppl 1):**P-067

**Rationale:** The possibility of having a sensitive, specific and prognostic biological marker for bacterial infections is a considerable challenge. A step was taken with the discovery of pracalcitonin.

**Patients and methods:** This is a prospective observational cohort study of 120 patients in the Medical Resuscitation Department of the University Hospital of Casablanca during the 6-month period, including patients in whom the PCT was dosed. The data collected allowed us to form two groups according to the PCT value: PCT+ group with PCT > 2 ng/ml and PCT− group with PCT < 2 ng/ml. The statistical analysis of these different data was carried out using Epi Info software version 3.5.3.

**Results:** 60% of our patients had a bacterial infection and 40% did not have one. We also distinguished community infections (42% of I+ patients) and nosocomial infections (58% of I+ patients). We found that the highest rates of PCT were in nosocomial infections and the lowest PCT rates were found in community-acquired infections. Then, in each type of organ involvement we tried to vary the PCT thresholds to 0.5–2 and 10 ng/ml in order to find the best threshold for which PCT allowed to diagnose bacterial infection, justifying our choice of departure. We concluded that the best PCT cut-off value in general was 2 ng/ml, because it gave us the best sensitivity/specificity ratio (67% and 50% respectively) with a positive predictive value of 65% and a negative predictive value of 52%. The link between PCT and bacterial infection was moderate (Yule Q-factor at 0.34). By analyzing the different therapeutic aspects, we showed that 71% of our patients had been treated with ATB before the PCT assay and that the broadest spectrum antibiotics available to our service were used in patients with PCT levels the highest. Finally, concerning the evolution, the higher the rate of PCT, the higher the death rate, especially since 100% of patients with PCT > 10 ng/ml died.

**Conclusion:** Procalcitonin is considered to be one of the best markers of systemic bacterial infection. Indeed, its elevation is earlier than that of CRP and its specificity is better compared to IL-6 and IL-8. The rate of procalcitonin remains low in the presence of viral infection. Procalcitonin is also a prognostic marker, its elevation is correlated with the severity of the infection, and its decrease is a good indicator of the effectiveness of antibiotic therapy.

**Compliance with ethics regulations:** Not applicable.

### P-068 Worldwide clinical practices in perioperative antibiotic therapy for lung transplantation

#### Benjamin Coiffard^1^, Eloi Prud’homme^2^, Sami Hraiech^2^, Nadim Cassir^3^, Laurent Papazian^2^

##### ^1^Assistance Publique-Hôpitaux de Marseille, Hôpital Nord, Médecine Intensive Réanimation, Marseille, France; ^2^APHM, Hôpital Nord, Médecine Intensive Réanimation, Marseille, France; ^3^Aix Marseille Univ, APHM, IHU-Méditerranée Infection, Marseille, France, Marseille, France

###### **Correspondence:** Benjamin Coiffard (bcoiffard.aphm@gmail.com)

*Ann. Intensive Care* 2020, **10(Suppl 1):**P-068

**Rationale:** Due to induction immunosuppression infection is the most common cause of mortality within the first year after lung transplantation (LTx). The management of perioperative antibiotic therapy is a major issue, but little is known about worldwide practices.

**Patients and methods:** We sent by email a survey to 180 LTx centers around the world dealing with 5 daily clinical vignettes concerning perioperative antibiotic therapy. We considered perioperative period as the period of the transplant surgery (per operative) and the post-surgery time before any infection occurrence (postoperative). After general questions on local practices, we asked each center for colonization definition and their diagnostic methods for microbial screening in recipients and donors. The clinical cases were related to specific issues concerning the management of antibiotic therapy in different clinical situations, including no prior colonization, prior colonization with sensitive or multi-drug resistant (MDR) microorganisms including prior colonization with MDR bacteria not sensitive to beta-lactams. The invitation and a weekly reminder were sent to lung transplant specialists for a single consensus answer per center between June and September 2018.

**Results:** We received a total of 99 responses from 24 countries, mostly from Western Europe (n = 46) and the USA (n = 34), (Fig. 1). Systematic screening for bronchial colonization before LTx was mostly performed with sputum samples (72%), regardless of the underlying lung disease. Definition of colonization was very heterogeneous and the delay between the last bacterial isolation in pre-transplant and the LTx to consider if the therapy should target these bacteria varied between 1 week and more than 1 year. In recipients without colonization, antibiotics with activity against gram-negative bacteria resistant strains (piperacillin/tazobactam, cefepime, ceftazidime, carbapenems) were reported in 72% of the centers, and antibiotics with activity against methicillin-resistant Staphylococcus aureus (mainly vancomycin) were reported in 38% of the centers. For these recipients, the duration of antibiotics reported was 7 days (33%) or less (26%) or stopped when cultures of donor and recipients were reported negatives (12%). In recipients with pre-transplant colonization, antibiotics were adapted to the susceptibility of the most resistant strain isolated in pre-transplant samples and given for at least 14 days (67%).

**Conclusion:** Practices vary widely around the world, but resistant bacterial strains are mostly targeted even if no colonization occurs. The antibiotic duration reported was longer for colonized recipients.

**Compliance with ethics regulations:** Not applicable.

**Fig. 1:**
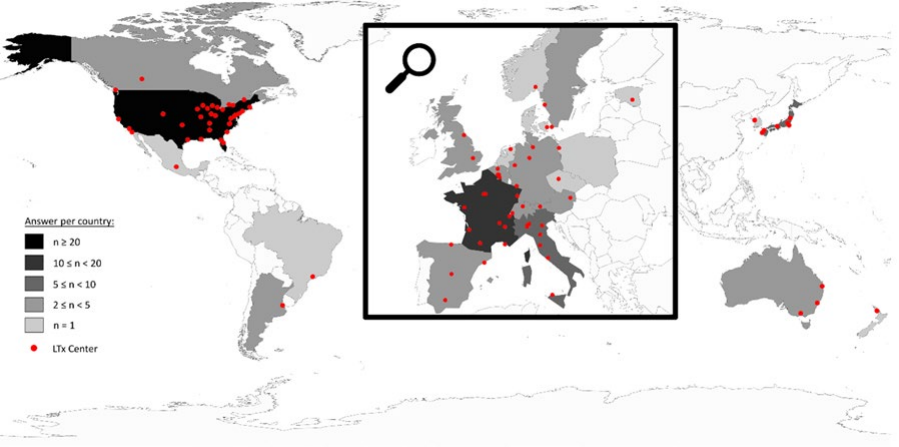
.

### P-069 Use of aminoglycosided in a French pediatric hospital: a retrospective study

#### Séverine Manen^1^, Cécile Bost-Bru^1^, Sébastien Chanoine^2^, Guillaume Mortamet^3^

##### ^1^Service de pédiatrie, CHU de Grenoble, Grenoble, France; ^2^Département de pharmacie, CHU de Grenoble, Grenoble, France; ^3^Service de réanimation pédiatrique, CHU de Grenoble, Grenoble, France

###### **Correspondence:** Séverine Manen (smanen@chu-grenoble.fr)

*Ann. Intensive Care* 2020, **10(Suppl 1):**P-069

**Rationale:** Since aminoglycosides are well-known for their synergic action with betalactams and their narrow spectrum, they are largely used in acute settings. However, due to the emergence of multi resistance drug bacteria, their use should be limited to specific indications. Since the publication of French recommendations in 2011, some adult studies revealed that aminoglycosides are still misused. Despite the pharmacological and physiological specificities in the pediatric population, there is a lack of data in this field in children. This study aimed to describe the use of aminoglycoside prescriptions in a pediatric center.

**Patients and methods:** A single center retrospective study performed in a tertiary hospital. All patients under 18 years old who received at least one intravenous dose of aminoglycosides (amikacin or gentamicin) at the Pediatric Intensive Care Unit (PICU) or Emergency Department (ED) were included. Patients with cystic fibrosis were excluded. The compliance in terms of dosage, duration of treatment and indication was assessed.

**Results:** A total of 149 patients (median age 10 months, 66 males) received aminoglycosides (33 amikacin and 116 gentamicin) during their stay, including 36 (24%) in PICU. In patients admitted in PICU, the administered dose of amikacin and gentamicin were 15.9 mg/kg/day and 4.1 mg/kg, respectively, for a mean duration of 2 days. Underdosing was found in a total of 8 patients (22% of patients in PICU). Ten patients (28%) presented a septic shock but peak serum concentrations were never dosed. Regarding patients admitted in ED, the main indication was pyelonephritis. Monotherapy was never prescribed. No patient experienced nephrotoxicity in our study. Overall, 77 prescriptions (48%) were considered as non appropriate according to the French recommendations. The main reason for misuse was the indication (n = 60, 78%), followed by the underdosing.

**Conclusion:** The most of aminoglycosides prescriptions did not follow the French recommendations in our study. Despite some studies suggest a higher dose or a use in monotherapy, such practices were not widespread.

**Compliance with ethics regulations:** Yes.

### P-070 Blood exchange transfusion in severe pertussis: mortality risk factors

#### Ahmed Ayari, Rim Ouerfelli, Ahmed Hajji, Shatila Hadj Hassine, Assaad Louati, Asma Bouziri, Khaled Menif, Aïda Borgi, Nejla Ben Jaballah

##### Service de Réanimation Pédiatrique Polyvalente/ Bechir Hamza Children’s Hospital of Tunis, Tunis, Tunisia

###### **Correspondence:** Ahmed Ayari (Ahmed.alayari@gmail.com)

*Ann. Intensive Care* 2020, **10(Suppl 1):**P-070

**Rationale:** Severe *Bordetella pertussis* infection is associated with a poor outcome in young infants. Recent data have suggested that blood exchange transfusion (BET) may be helpful for children with pertussis and leucocytosis. We aimed to identify risk factors for mortality in patients with severe pertussis who have had BET.

**Patients and methods:** We collected over a period of 6 years (2013–2018) demographic and clinical data of children hospitalized in the paediatric intensive care unit for severe pertussis and undergone BET.

**Results:** Twenty-six infants were admitted (sex ratio 1.3, mean age: 49 days (± 23)). Of these patients, 11.5% were former premature babies and 50% were hypotrophic. At admission the mean respiratory rate was 61 c.p.m (± 17). The average heart rate was 197 b.p.m (± 27). The mean white blood cell count was 80,369/mm^3^ (± 28,000), the mean lymphocyte count was 36,160/mm^3^ and 63% of our patients had pulmonary hypertension (PH). All patients were intubated ventilated and 61.5% have had a haemodynamic support. All our patients have had a BET. Fifty percent of the patients died. Mortality risk factors were: white blood cell count at admission (p = 0.02), pH (p = 0.002), and the use of norepinephrine (p = 0.02).

**Conclusion:** Identifying risk factors might allow for implementation of more rapid intervention.

**Compliance with ethics regulations:** Yes.

### P-071 Pneumocystis pneumonia in a pediatric intensive care unit: a retrospective study from 2006 to 2019

#### Arielle Maroni, Stéphane Dauger, Camille Dollat, Jérôme Naudin, Anna Deho, Géraldine Poncelet, Michael Levy, Fleur Le Bourgeois, Guillaume Geslain, Maryline Chomton

##### Hospital Robert DEBRE-Pediatric Intensive Care Unit, Paris, France

###### **Correspondence:** Arielle Maroni (arielle.maroni@aphp.fr)

*Ann. Intensive Care* 2020, **10(Suppl 1):**P-071

**Rationale:** *Pneumocystis jirovecii* is a ubiquitous fungus, which causes severe pneumonia in immunocompromised children and required frequent admission in the Pediactric Intensive Care Unit (PICU). Data on their management are poor in pediatric literature. The aim of our study was to describe the clinical characteristics and the management of *Pneumocystis jirovecii* Pneumonia (PJP) in our PICU.

**Patients and methods:** This retrospective observational study included children aged less than 18 years old who were admitted to the PICU and with a discharge diagnosis of PJP between 2006 and 2019. Age, underlying diseases, clinical signs, co-infections, supportive care, treatment response and clinical course were collected.

**Results:** Nineteen children, aged from 2 months to 16 years, were included and all presented acute respiratory failure rapidly progressing to a severe acute respiratory distress syndrome (ARDS) for 14 patients (74%). Underlying disease was known for 6 patients and PJP revealed immunodeficiency state for 13 patients: 10 cases of severe combined immunodeficiency (SCID) and 3 cases of mother-to-child transmission of HIV. Median time to start trimethoprim-sulfamethoxazole was 2 days (min: 0–max: 25) after PICU admission. Until November 2012, diagnosis performed on broncho-alveolar lavage fluid; thereafter we used polymerase chain reaction (PCR) on nasopharyngeal or tracheal aspirate. Fourteen patients (74%) were intubated at day 1 post admission (min: 0–max: 13) for a median duration of 9 days (min: 1–max: 27). Among the 5 patients who were not intubated, 4 needed non-invasive ventilation. Adjuvant therapeutic steroids were added for 12 patients (63%) at 4.5 days (min: 0–max: 25). Viral co-infections were frequent: 9 patients (47%) presented CMV blood co-infection and 8 patients (42%) presented viral respiratory co-infection (Rhinovirus, RSV and CMV). Three patients (16%) died: 2 deaths were caused by refractory hypoxemia related to PJP (both suffered from SCID), the third patient died from broncho-pulmonary dysplasia after recovery from PJP. Among the 16 survivors, 13 (81%) required supplementary oxygen therapy at the PICU discharge. Median length of stay in PICU was 14 days (min: 1–max: 47).

**Conclusion:** PJP is responsible of life-threatening ARDS. Mechanical ventilation is required in most cases. Now, diagnosis is preferentially achieved by PCR. Viral co-infections would be systematically researched and treated. PJP is responsible of prolonged hospitalizations and supplementary oxygen therapies after PICU stay.

**Compliance with ethics regulations:** Not applicable.

### P-072 Prevalence of Staphylococcus aureus health care-associated infections among carriers and associated risk factors in a pediatric intensive care unit

#### Perrine See^1^, Stéphane Bonacorsi^2^, Jérôme Naudin^1^, Anna Deho^1^, Géraldine Poncelet^1^, Catherine Doit^2^, Fleur Le Bourgeois^1^, Maryline Chomton^1^, Stéphane Dauger^1^, Michael Levy^1^

##### ^1^Réanimation et surveillance continu pédiatriques, Hôpital Robert-Debré, APHP, Paris, France; ^2^Microbiologie, Hôpital Robert-Debré, APHP, Paris, France

###### **Correspondence:** Perrine See (perrinesee@aol.com)

*Ann. Intensive Care* 2020, **10(Suppl 1):**P-072

**Rationale:** 20 to 30% of the paediatric population is colonized with nasal Staphylococcus aureus (SA) and this germ is currently one of the leading causes of health care-associated infections (HCAI) in neonatal and paediatric intensive care units (PICU). It is known that the carriage of SA increases the risk of HCAI caused by SA but there is a lack of data regarding the incidence of HCAI in colonized patients and the associated risk factors.

**Patients and methods:** We performed a retrospective single-center descriptive study including all patients hospitalized in Robert-Debré University Hospital PICU and colonized with SA between the 1st of January 2011 and the 31st of December 2013. Data were collected using the microbiology department’s prospective database, hospital records, and biological results reporting software. The results of the descriptive analysis were expressed as numbers and percentages for qualitative variables and as mean and standard deviation for quantitative variables. Two-tailed Fisher’s exact test for quantitative variables and Chi-square test for qualitative variable were used. Risk of infection was modeled using a multivariate logistical regression analysis.

**Results:** Among the 2383 patients hospitalized during the studied period, 573 patients were colonized with SA. Among them, 20 patients developed 21 episodes of HCAI caused by SA with an incidence of 3.7%. These HCAI were mainly respiratory infections (67%) followed by osteoarticular infections (9%), bacteremia (9%) and skin infections (9%). Patients with an HCAI caused by SA had a significantly longer stay duration and a higher mortality rate than the rest of the population. Multivariate analysis showed that antibiotic therapy in the 2 months prior to the hospitalization and hyponatremia during the hospitalization were significantly associated with the occurrence of HCAI caused by SA. On the other hand, an antibiotic therapy performed for another infection during the stay appeared to be a protective factor.

**Conclusion:** SA infection occurs in 3.7% of colonized patients and has a significant impact on their length of stay and their mortality. Our results suggest that previous antibiotic therapy in the last 2 months before the hospitalizations, as well as the occurrence of hyponatremia during the stay represent significant risk factors for developing HCAI.

**Compliance with ethics regulations:** Yes.

### P-073 Acinetobacter baumannii nosocomial infections in pediatric intensive care unit at Casablanca Ibn Rochd Hospital

#### Samira Kalouch, Khalid Yakini, Wissal Aissaoui, Abdelaziz Chlilek

##### CHU ibn rochd casablanca, Casablanca, Morocco

###### **Correspondence:** Samira Kalouch (dr.kalouch@gmail.com)

*Ann. Intensive Care* 2020, **10(Suppl 1):**P-073

**Rationale:** *Acinetobacter baumannii* is a ubiquitous pathogen, resistant to desiccation, responsible for care associated infections, especially in intensive care.

**Patients and methods:** Our work is a retrospective descriptive study over a period of 3 years (December 2015, December 2017) on patients who presented a nosocomial infection with *Acinetobacter baumannii* during their hospitalization in pediatric intensive care unit.

**Results:** In our study the majority of patients were ventilated/intubated (92%), with bladder catheter port (40.30%). While (92.90%) of the patients were tracheotomized. Infectious site was mainly: pulmonary (82%), associated with bacteremia (18%) while (16%) was due to infections in the catheter. The average time of onset of infection was 10 days with extremes of 2 days to 67 days. We noted a very important resistance to the majority of antibiotics: gentamicin 99%, ceftazidime 97%, ciprofloxacin 93%, imipenem 85% and amikacin 77%. While 19% of the strains were sensitive to rifampicin. Resistance to tigecycline and colimycin was 1%. There was a high mortality rate of 63% with male predominance.

**Conclusion:** It appears from our results that nosocomial infections with *Acinetobacter baumannii* and resistance of this germ to antibiotics are worring. The judicious use of antibiotics, hand washing and the use of sterile equipment are essential to reduce the incidence of nosocomial infection with *Acinetobacter baumannii*. Epidemiological surveillance of infections in intensive care and adherence to hygiene measures are priorities to be included in any program of nosocomial infection control and prevention.

**Compliance with ethics regulations:** Not applicable.

### P-074: Postoperative treatment with vancomycin in children with liver or combined liver-kidney transplantation

#### Mehdi Oualha^1^, Hélène Yager^1^, Delphine Callot^2^, Mathieu Genuini^3^, Julie Toubiana^2^, Agathe Béranger^1^, Florence Lacaille^1^, Romain Berthaud^1^, Christophe Chardot^1^, Carole Hennequin^1^, Anais Brassier^1^, Sihem Benaboud^1^, Sylvain Renolleau^1^

##### ^1^Hôpital Necker enfants malades, Paris, France; ^2^Hôpital Cochin, Paris, France; ^3^Hôpital Robert Debré, Paris, France

###### **Correspondence:** Mehdi Oualha (mehdi.oualha@aphp.fr)

*Ann. Intensive Care* 2020, **10(Suppl 1):**P-074

**Rationale:** Children with liver or combined liver-kidney transplantation may receive vancomycin during the early phase of postoperative stage for suspected infections related to gram positive agents. To describe the use of vancomycin and associated exposure and nephrotoxicity.

**Patients and methods:** This single center and retrospective study was conducted in pediatric intensive and intermediate care units at Necker hospital from January 2015 to June 2019. We included all children (< 18 years old) with liver or combined liver-kidney transplantation who received continuous vancomycin infusion during the postoperative stage for at least 24 h. Nephrotoxicity related to vancomycin was assessed using the pRiFLE classification and by the expert advice of the local pharmacovigilance unit. Supratherapeutic exposure to vancomycin was defined when the vancomycin plasmatic concentration at steady state was > 30 mg/L. Catheter related infection was defined using the IDSA recommendations. A posteriori analysis of appropriateness of vancomycin use was assessed by an infectious disease expert. The vancomycin was therefore considered as justified or not and appropriate or not. Occurrence of nephrotoxicity and supratherapeutic exposure in this study group was compared to critically ill children control group.

**Results:** Thirty one children receiving 43 vancomycin lines of treatment whose 13 (26%) observed a risk of acute kidney injury (AKI) (n = 8) and an AKI (n = 5) during the vancomycin treatment period were included. There was a trend to inversed relationship between plasmatic concentrations of vancomycin and estimated creatinine clearance (r2 = 0.2019). Seven patients observed a nephrotoxicity related to vancomycin, they had a higher plasmatic concentration of vancomycin (p = 0.0009). Seven patients (22%) had a supratherapeutic exposure to vancomycin. Nephrotoxicity and supratherapeutic exposure were higher in children with or combined liver-kidney transplantation than in comparative critically ill children group. We found 1 blood stream infection due to the central catheter and 7 blood stream infections probably due to the central catheter. One hundred thirty-five bacteria were identified of which 108 (80%) were Staphylococcus coagulase negative. Nineteen (44%) lines of vancomycin were appropriate and 31 (72%) were justified.

**Conclusion:** Vancomycin could have been avoided in one third of children with liver or combined liver-kidney transplantation during the early phase of postoperative stage. Vancomycin is associated with a risk of both nephrotoxicity and supratherapeuric exposure. Vancomycin should be used with caution, appropriate indications and dosing in this vulnerable population.

**Compliance with ethics regulations:** Yes.

### P-075 Early bacterial infections after pediatric liver transplantation in the era of multiresistant bugs: a 9-year retrospective experience

#### Agathe Béranger^1^, Carmen Capito^2^, Florence Lacaille^3^, Agnès Ferroni^4^, Naïm Bouazza^5^, Muriel Girard^3^, Mehdi Oualha^1^, Sylvain Renolleau^1^, Dominique Debray^3^, Christophe Chardot^2^, Pierre Frange^4^, Florence Moulin^1^

##### ^1^Réanimation pédiatrique, Necker Enfants Malades, Paris, France; ^2^Chirurgie viscérale pédiatrique, Necker Enfants Malades, Paris, France; ^3^Hépatologie pédiatrique, Necker Enfants Malades, Paris, France; ^4^Microbiologie clinique, Necker Enfants Malades, Paris, France; ^5^URC, Cochin-Necker Enfants Malades, Paris, France

###### **Correspondence:** Agathe Béranger (agathe.beranger@gmail.com)

*Ann. Intensive Care* 2020, **10(Suppl 1):**P-075

**Rationale:** Early bacterial infection is a major and severe complication occurring within the first month after pediatric liver transplantation (LT). The rise of antimicrobial resistance, especially extended-spectrum beta lactamase producing Enterobacteriaceae (ESBL-PE), is henceforth a concern for these patients. This study aimed to assess the epidemiology of early bacterial infections, including those caused by multidrug-resistant (MDR) pathogens, and to identify the risk factors for infection.

**Patients and methods:** We conducted a monocentric retrospective study including 142 consecutive LTs in 137 children from 2009 to 2017.

**Results:** Ninety-three bacterial infections occurred after 67 (47%) LTs. Among the 82 isolated pathogens, the most common were *Klebsiella pneumoniae* (n = 19, 23%) and coagulase negative Staphylococcus (n = 16, 20%). Independent risk factors for early bacterial infection were a low weight (OR = 0.96, 95% CI (0.92–0.99), p = 0.03) and the presence of a prosthetic mesh (OR = 2.4, 95% CI (1.1–5.4), p = 0.046). Sixty-one (45%) children carried MDR bacteria and 16 infections were caused by MDR pathogens, especially ESBL producing *K. pneumoniae*. ESBL-PE carriage was associated with ESBL-PE infection (OR = 4.5, 95% CI (1.4–17.4), p = 0.02). Four children died from an early bacterial infection, three of which were caused by ESBL producing *K. pneumoniae*.

**Conclusion:** Early bacterial infection is a known complication after pediatric LT, with a shift toward a predominance of Gram-negative bacteria infections. The most important risk factors were low weight and presence of a prosthetic mesh. Stool ESBL-PE carriage was highly prevalent and associated with ESBL-PE infection. An adapted antimicrobial prophylaxis and personalized antibiotherapy are mandatory to reduce the infection prevalence and mortality.

**Compliance with ethics regulations:** Yes.

**Table 1:** Risk factors for early bacterial infections after liver transplant

Variables, n (%) or median [IQR]	Infected patients, n = 67 (47%)	Non-infected patients, n = 75 (53%)	Univariate p	Multivariate analysis	
				OR (95% CI)	p
Preoperative data					
Age (years)	1.75 [0.9–6.3]	3.5 [1.3–9.7]	**0.02**		
Weight (kg)	11 [8-19]	14.4 [9–28.3]	**0.009**	**0.96 (0.9**–**0.99)**	**0.03**
Male gender	37 (55%)	37 (49%)	NS		
Malnutrition	22 (33%)	28 (37%)	NS		
Biliary atresia	37 (55%)	39 (52%)	NS		
Previous abdominal surgeries	49 (73%)	47 (63%)	NS		
High-emergency waiting list	25 (37%)	24 (32%)	NS		
Graft/recipient weight ratio (%)	2.9 [1.6-4]	2.7 [1.8-3.3]	NS		
Intra-operative data					
Serum lactate (mmol.L^−1^)	3.8 [3–5.5]	3.9 [3.1–4.9]	NS		
Transfusion volume (TBVR*)	2.3 [1.3–3.9]	1.4 [0.8–2.2]	**0.001**	1 (0.9–1.2)	NS
Cold ischemia time (hours)	8.3 [7.3–9.3]	8.1 [6.5–9.1]	NS		
Presence of prosthetic mesh	30 (45%)	16 (21%)	**0.005**	**2.4 (1.1**–**5.4)**	**0.046**
Postoperative data					
Surgical complications	23 (34%)	18 (24%)	NS		
Acute rejection	30 (45%)	30 (40%)	NS		

*TBVR : transfusion/blood volume ratio, estimated as transfusion volume of blood products/total circulating blood volume = transfusion volume (mL)/[80 mL kg^−1^ × weight (kg)]

NS : non-significant and p > 0.1

### P-076 B-lactams in critically ill children with renal failure and continuous renal replacement therapy: dosing and exposure

#### Mehdi Oualha, Benjamin Prim, Déborah Hirt, Saoussen Krid, Inès Gana, Fabrice Lesage, Romain Berthaud, Sihem Benaboud, Jean-Marc Tréluyer, Sylvain Renolleau

##### Necker Hospital, Paris, France

###### **Correspondence:** Mehdi Oualha (mehdi.oualha@aphp.fr)

*Ann. Intensive Care* 2020, **10(Suppl 1):**P-076

**Rationale:** Renal failure (RF) occurs in 1/3 of critically ill children with sometimes need of continuous renal replacement therapy (CRRT). In sepsis, appropriate antibiotic plasmatic concentrations are associated with better clinical outcomes. This study aims to describe the dosing of β-lactams and plasmatic concentrations in critically ill children with RF and/or CRRT and to identify clinical and biological variables associated with β-lactams sub optimal exposure.

**Patients and methods:** We included all critically ill children with RF and/or CRRT receiving β-lactams from January 2016 to December 2017

**Results:** 39 critically ill children were included, 24 and 22 during RF and CRRT period, respectively. Initial dosing adjustment was done in 5 children (18%) with RF and in 9 children (32%) under CRRT. According to the guidelines, appropriate adjustments were observed in 10 (37%) children with RF. At 1st sample, 8 patients (32%) with RF had insufficient β-lactams concentration whereas 6 patients (24%) had supra therapeutic exposure. 9 children with RF observed at least one insufficient β-lactams concentration during the treatment. For children under CRRT, 13 (56%) had insufficient β-lactams concentration and one had supra therapeutic exposure. 17 children (73%) under CRRT observed at least one insufficient β-lactams concentration during the treatment. High dialysate flow rate, 113 ml/kg/h (77–174) was associated with insufficient β-lactams concentrations (p = 0.018).

**Conclusion:** More than half of critically ill children with renal failure or CRRT had inadequate β-lactams concentration. Optimizing dosing regimen of β-lactams and therapeutic drug monitoring of concentrations are mandatory in this population.

**Compliance with ethics regulations:** Yes.

### P-077 Oncological digestive emergencies profile and become

#### Khalid Khaleq, Wafaa Eddouissi, Fatimazahra Bensardi, Rachid Al Harrar

##### Faculté de médecine et de pharmacie/ CHU ibnou Rochd, Casablanca, MOROCCO

###### **Correspondence:** Khalid Khaleq (khaleq20@gmx.fr)

*Ann. Intensive Care* 2020, **10(Suppl 1):**P-077

**Rationale:** The number of cancer patients admitted to emergencies is clearly increasing and digestive oncology is the leading cause of consultation. The aim of this work is to identify the epidemiological factors, the therapeutic modalities as well as the predictive factors of mortality and to compare them with the data of the literature.

**Patients and methods:** 81 patients admitted to visceral emergencies for an urgent syndrome revealing or complicating a primary or secondary digestive cancer, and who required immediate medical and/or surgical intervention and who had stayed at the surgical resuscitation level in our hospital center for a duration of 2 years. Several data were entered on Excel and analyzed using the SPSS version 20 software.—Epidemiological, concerning age and sex; —Clinics including risk factors, history, general condition of the patient and clinical examination data; —Para-clinical, interesting biological assessments, and morphological examinations—Medical and surgical therapeutics; —Postoperative follow-up—Treatment results.

**Results:** The three most frequent sites were rated in order of increasing frequency: colo-rectum (49%), pancreas (17%), and stomach (12%). The age group most found was age over 60 years with 48% of cases, 12% of patients had under 40 years. This series includes 46 men and 35 women with a sex ratio of 1, 31. The installation method was mostly gradual with 62% of cases. Our patients have consulted for urgent clinical presentations mainly occlusive syndrome noted in 47% of patients. Abdominal CT was the first examination performed, followed by abdominal ultrasonography in 73% and 22%, respectively. The therapeutic management was medico-surgical. The surgery done in 96% of patients, 74% for palliative indication: 40% were operated for an ostomy discharge, 24% for a digestive bypass, 24% for a palliative resection and 12% for a stoma feeding. Postoperative outcomes were 44% morbidity and 43% mortality. The main cause of death was septic shock in 80% of cases, thanks to multivariate statistical analysis three factors were deduced significantly related to mortality: The ASA score: p = 0.004; OR = 2.520; IC: [1.33; 4.74], vasoactive drugs: p  =  0.037; OR = 3.868; IC: [1.08; 13.82] and the experience of the anesthetist (junior): p < 0.001; OR = 17.678; IC: [4.20; 74.28].

**Conclusion:** In our context, digestive cancers are a frequent reason for emergency consultation. The majority of surgical or medical interventions were palliative and the postoperative outcome is marked by high morbidity and mortality.

**Compliance with ethics regulations:** Not applicable.

### P-078 Outcome of intracranial hemorrhage in patients with acute leukemia: results of a retrospective cohort study

#### Yannick Hourmant, Sandrine Valade, Adrien Mirouse, Jean Jacques Tudesq, Asma Mabrouki, Eric Mariotte, Virginie Lemiale, Lara Zafrani, Michaël Darmon, Elie Azoulay

##### Hôpital Saint Louis -APHP-Médecine intensive et réanimation, Paris, France

###### **Correspondence:** Yannick Hourmant (yannick.hourmant@aphp.fr)

*Ann. Intensive Care* 2020, **10(Suppl 1):**P-078

**Rationale:** Acute leukemia (AL) is a life threatening hematological disease. ICU is frequently required for life threatening complications including disseminated intravascular coagulation, primary fibrinolysis and severe hemorrhages. Primary objective was to assess survival and neurological outcome in critically ill AL patients with an intracranial hemorrhage. Secondary outcome was to describe characteristics of these patients.

**Patients and methods:** Single center retrospective cohort including adult critically ill patients with AL who experienced intracranial hemorrhage between 2002 and 2019. Type of leukemia was classified according to FAB classification. Brain imaging (CT or MRI) was available for all the patients. Stupor was defined by Glasgow coma scale ≤ 14. Results are reported as median [IQR] or number (%). Adjusted analysis was performed using Cox Model.

**Results:** Thirty-five AL patients, aged of 59 [36–66] years, were included. Acute myeloid leukemia (AML) was the most frequent underlying condition in 29 patients (83%), including 12 patients with acute promyelocytic leukemia (APL). Median white blood cell count was 106 G/L [23.1–26.6] and thrombocytopenia was constant with a median platelet count at 42 G/L [22–81]. At ICU admission median SOFA score was 5 [3–9]. Multiple organ failure was frequent, 24 patients (68.6%) requiring invasive mechanical ventilation (IMV), 10 (28.6%) vasopressors, and 5 (14.2%) renal replacement therapy. Time between AL onset and intracranial hemorrhage was 2 [0–9.5] days. Brain imaging was performed in relation with several neurological symptoms including headaches (31%), delirium (25%) or stupor (68.6%). Disseminated intravascular coagulation (DIC) was present in half of the patients (48.5%): median fibrinogen level was 1 [0.85–1.37] g/L. ICU and hospital mortality rates were 60% (n = 21) and 65.7% (n = 23), respectively. Ten patients were alive 6 months after with a median Rankin score at 0 [0–1]. More than half of the patients without stupor had a favorable neurological outcome (Fig. 1). In univariate analysis, mechanical ventilation and stupor were correlated with mortality, whereas DIC and APL were not. By multivariate analysis stupor was the only factor significantly associated with a higher mortality (HR: 8.56 [2.4–30.4]).

**Conclusion:** Intracranial hemorrhage is associated with a high mortality rate in AL patients, stupor at the onset of intracranial bleeding being independently associated with poor outcome. Up to one third of patients will nevertheless survive and experience a favorable neurological outcome.

**Compliance with ethics regulations:** Yes.

**Fig. 1:**
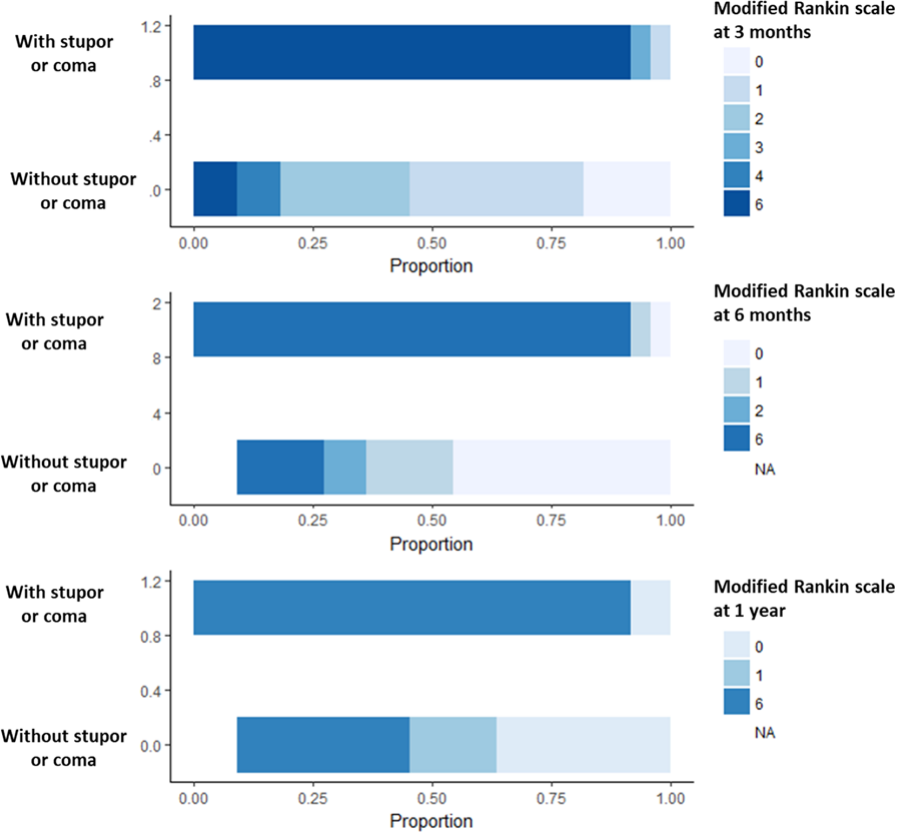
Neurological outcome assessing by modified Rankin scale according to stupor or coma at intracranial hemorrhage diagnosis (blank reflect missing data)

### P-079 Sinusoidal obstruction syndrome in critically ill patients in the era of defibrotide (ISOS study): results of a retrospective cohort study

#### Pierre-Edouard Debureaux^1^, Michaël Darmon^1^, Naike Bige^2^, Anne-Sophie Moreau^3^, Djamel Mokart^4^, Guillaume Morel^5^, Claire Lacan^5^, Pierre Perez^6^, Frédéric Pene^7^, Achille Kouatchet^8^, Muriel Picard^9^, Martin Murgier^10^, Julien Mayaux^11^, Emmanuel Canet^12^, Elie Azoulay^1^, Sandrine Valade^1^

##### ^1^Medical Intensive Care Unit, Saint Louis Hospital, Paris, France; ^2^Medical Intensive Care Unit, Saint Antoine Hospital, Paris, France; ^3^Critical Care Center, CHU de Lille, Lille, France; ^4^Critical Care Center, IPC, Marseille, France; ^5^Intensive Care Unit, CHU Strasbourg, Strasbourg, France; ^6^Medical Intensive Care Unit, Brabois University Hospital, Nancy, France; ^7^Medical Intensive Care Unit, Cochin Teaching Hospital, Paris, France; ^8^Medical Intensive Care Unit, Angers Teaching hospital, Angers, France; ^9^Department of Intensive Care, IUCT-Oncopôle, Toulouse, France; ^10^Medical-Surgical Intensive Care Unit, Saint-Priest-en-Jarez Hospital, Saint Etienne, France; ^11^Medical ICU, Pitié-Salpêtrière Hospital, Paris, France; ^12^Médical Intensive Care Unit, Centre Hospitalier Universitaire-Nantes, Nantes, France

###### **Correspondence:** Pierre-Edouard Debureaux (hemato.debureaux@gmail.com)

*Ann. Intensive Care* 2020, **10(Suppl 1):**P-079

**Rationale:** Sinusoidal obstruction syndrome (SOS, previously known as veno-occlusive disease) is a complication of high dose chemotherapy, frequently occurring during bone marrow transplantation (BMT). Severe SOS is associated with a high mortality rate, related to multi-organ failure (MOF). Defibrotide being the only available option for prevention and treatment. Prognosis of patients with SOS requiring intensive care unit (ICU) admission remains unknown. The primary objective was to assess the outcome of these patients. Secondary objective was to assess risk factors associated with hospital mortality.

**Patients and methods:** Retrospective study conducted between January 2007 and July 2019 in 12 French ICUs. Critically ill adult patients with SOS (according to EBMT classification) who received defibrotide were included. Results are reported as median [IQR] or number (%). Adjusted analysis was performed using Cox Model.

**Results:** Seventy-one patients were included with a median age of 47 years [IQR 32–57]. Underlying hematologic diseases were acute myeloid leukemia (41%), lymphoma (21%), myelodysplasia/myeloproliferative neoplasm (18%) or acute lymphoid leukemia (15%). SOS occurred during myeloablative allogeneic BMT (34%), reduced conditioning allogeneic BMT (41%), autologous BMT (11%) or chemotherapy (14%, including Gemtuzumab Ozogamycin in 7 patients). Median SOFA score at ICU admission was 11 [IQR 7.5–12.5]. EBMT prognostic score was often “very severe” (69%). Main reasons for ICU admission were respiratory failure (n = 26), acute renal injury (n = 17), shock (n = 11), liver failure (n = 8), coma (n = 6) and monitoring (n = 3). Median bilirubin level at ICU admission was 51 µmol/L [IQR 36–90] and platelets count 24 G/L [IQR 15–44]. Mechanical ventilation (MV), vasopressors, and renal replacement therapy (RRT) were required in 59% (n = 42), 52% (n = 37) and 49% (n = 34) of patients, respectively. Sixteen patients receiving defibrotide experienced bleeding events. ICU and hospital mortality rates were 39% and 54% respectively, mainly related to organ dysfunction. In univariate analysis, delayed defibrotide initiation, bilirubin level, organ supports, SOFA, and EBMT scores were associated with hospital mortality. Cox model identified older age (HR 1.02, 95% CI 1.00-1.04), MV (HR 1.99, 95% CI 1.00–3.99), RRT (HR 2.55, 95% CI 1.32–4.91), as associated with mortality. Prophylactic Defibrotide was correlated with a better outcome (HR 0.35, 95% CI 0.13–0.92). Similar results were observed after adjustment for center effect.

**Conclusion:** When organ support is required, ICU management is associated with high mortality. Organ support (namely RRT and MV) and older age were associated with poor outcome. Prophylactic Defibrotide was associated with survival either due to selection process or to efficacy in this setting. Additional studies are needed to confirm these results.

**Compliance with ethics regulations:** Yes.

**Fig. 1:**
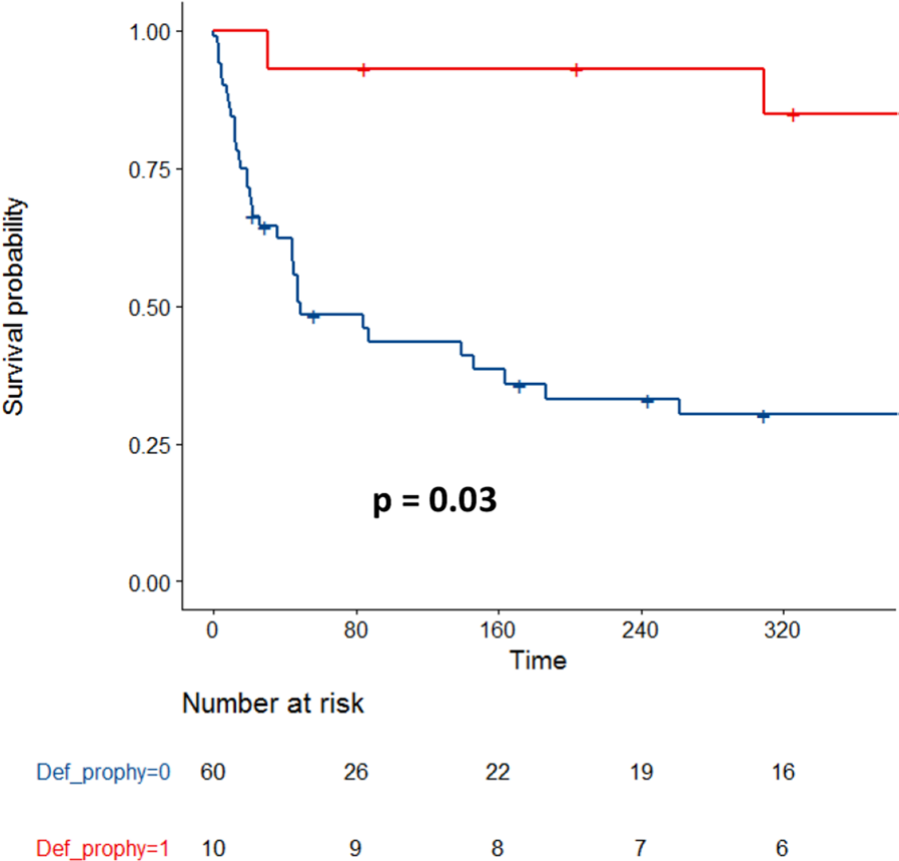
Survival curve according to defibrotide treatment after adjustment of confounding factors

### P-080 Etiology and outcome of acute respiratory failure in critically Ill immunocompromised and neutropenia: Retrospective analysis of a prospective multicenter multinational dataset

#### Djamel Mokart^1^, Michaël Darmon^2^, Achille Kouatchet^3^, Massimo Antonelli^4^, Martin Balik^5^, Andreas Barratt-Due^6^, Philippe Bauer^7^, Gaston Burghi^8^, Alexandre Demoule^9^, David Grimaldi^10^, Ignacio Martin Loeches^11^, Geeta Mehta^12^, Victoria Metaxa^13^, Saad Nseir^14^, Anders Perner^15^, Peter Pickkers^16^, Jean Reignier^17^, Jordi Rello^18^, Peter Schellongowski^19^, Marcio Soares^20^, Miia Valkonen^21^, Andry Van De Louw^22^, Virginie Lemiale^2^, Elie Azoulay^2^

##### ^1^Réanimation, Institut Paoli-Calmettes, Marseille, France; ^2^Réanimation médicale Hôpital Saint Louis, Paris, France; ^3^Réanimation médicale CHU Angers, Angers, France; ^4^Agostino Gemelli University Hospital, Università Cattolica del Sacro Cuore, Rome, Italy; ^5^Department of Anesthesiology and Intensive Care Medicine, Charles University in Prague and General University Hospital, Prague, Republic Czech; ^6^Department of Emergencies and Critical Care, Oslo University Hospital, Oslo, Norway; ^7^Pulmonary and Critical Care Medicine, Mayo Clinic, Rochester, USA; ^8^Terapia Intensiva, Hospital Maciel, Montevideo, Uruguay; ^9^Réanimation médicale Hôpital Pitié Salpetrière, Paris, France; ^10^Department of Intensive Care, Hôpital Erasme, Université Libre de Bruxelles, Brussels, Belgium; ^11^Department of Intensive Care Medicine, Trinity College, Dublin, Ireland; ^12^Department of Medicine and Interdepartmental Division of Critical Care Medicine, Sinai Health System, University of Toronto, Toronto, Canada; ^13^King’s College Hospital, London, UK; ^14^Critical Care Center, CHU Lille, School of Medicine, University of Lille, Lille, France; ^15^Department of Intensive Care, Rigshospitalet, University of Copenhagen, Copenhagen, Danmark; ^16^The Department of Intensive Care Medicine (710), Radboud University Medical Center, Nijmegen, Netherlands; ^17^Medical Intensive Care Unit, Hôtel Dieu-HME University Hospital of Nantes, Nantes, France, Nantes, France; ^18^CIBERES, Universitat Autonòma de Barcelona, European Study Group of Infections in Critically Ill Patients (ESGCIP), Barcelona, Spain; ^19^Department of Medicine I, Medical University of Vienna, Vienna, Austria; ^20^The Department of Critical Care and Graduate Program in Translational Medicine, Rio De Janerio, Brazil; ^21^Division of Intensive Care Medicine, Department of Anesthesiology, Intensive Care and Pain Medicine, University of Helsinki and Helsinki University Hospital, Helsinki, Finland; ^22^Division of Pulmonary and Critical Care, Penn State University College of Medicine, Hershey, USA

###### **Correspondence:** Djamel Mokart (mokartd@ipc.unicancer.fr)

*Ann. Intensive Care* 2020, **10(Suppl 1):**P-080

**Rationale:** Prognosis of critically ill immunocompromised patients (CIIP) has improved over time. Neutropenia is common and is found in one third of these patients. Prognostic impact of neutropenia remains controversial and little data focus on CIIP admitted in a context of acute respiratory failure (ARF). Primary objective was to assess prognostic impact of neutropenia on outcome of these patients. Secondary objective was to assess etiology of ARF according to neutropenia.

**Patients and methods:** Retrospective analysis of prospective multicenter multinational dataset. Adults immunocompromized patients with ARF were included. Adjusted analyses included (1) a hierarchical model with center as random effect; (2) propensity score (PS) matched cohort; and (3) adjusted analysis in the matched cohort.

**Results:** Overall, 1481 patients were included in this study. Median age was 64 [IQR 55–72] and 613 patients (39.7%) were of female gender. Median SOFA score was 7 [4–10] and PS was 1 [0–3]. Main immune defect were hematological malignancy in 533 patients (36%), solid tumor in 473 (32%), systemic disease in 155 (10.5%), and other immunosuppressive drugs in 117 (8%). Neutropenia at admission was observed in 165 patients (11%). Initial oxygenation strategy was Oxygen in 755 patients (51%), High flow nasal oxygen in 165 (11%), non-invasive ventilation in 202 (14%) and invasive mechanical ventilation in 359 (24%). Before adjustment, hospital mortality was significantly higher in neutropenic patients (54% vs. 42% in non-neutropenic patients; p = 0.006). After adjustment for confounder in a mixed model, neutropenia was no longer associated with outcome (OR 1.40, 95% CI 0.93–2.11). After PS matching, 148 neutropenic and non-neutropenic patients were compared. Hospital mortality was similar in both groups (52% vs. 46% respectively; p = 0.35). After adjustment for variables associated with mortality, neutropenia was not associated with hospital mortality (OR 1.04, 95% CI 0.63–1.72). ARF etiologies were distributed similarly in both neutropenic and non-neutropenic patients (Fig. 1), main etiologies being bacterial pneumonia (49% vs. 46%), invasive fungal infection (11% vs. 10%), pneumocystis jiroveci pneumonia (2% vs. 0.7%), and undetermined etiology (8% vs. 10%) (p = 0.815).

**Conclusion:** Neutropenia at ICU admission is not associated with hospital mortality in this cohort of CIIP admitted for ARF. Surprisingly, ARF etiology did not differ despite the multiplicity of observed immune defects.

**Compliance with ethics regulations:** Yes.

**Fig. 1:**
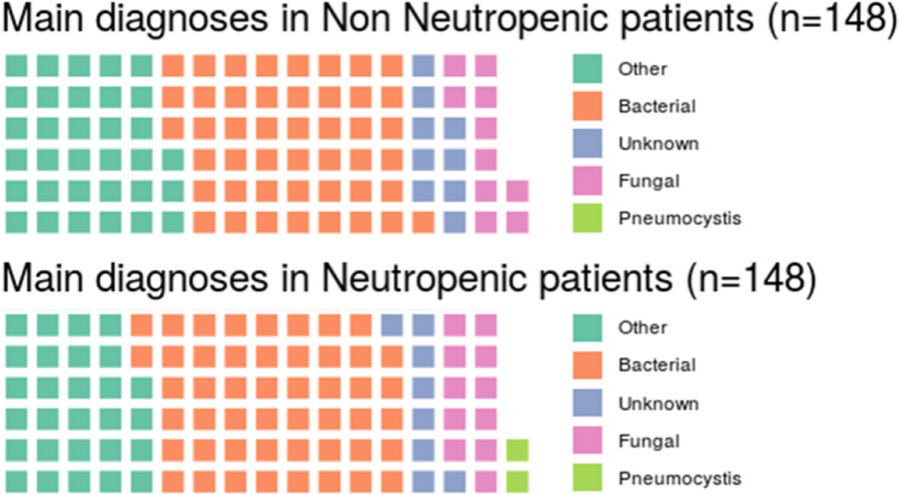
Main diagnoses according to neutropenia in the matched cohort

### P-081 Hepatic dysfunction in critically ill patients with hematologic malignancies: an underestimated life-threatening situation

#### Magali Bisbal^1^, Michaël Darmon^2^, Colombe Saillard^3^, Virginie Lemiale^2^, Frédéric Pene^4^, Achille Kouatchet^5^, Vincent Mallet^6^, Charlotte Mouliade^6^, Alexandre Demoule^7^, Francois Vincent^8^, Martine Nyunga^9^, Fabrice Bruneel^10^, Anne Renault^11^, Christine Lebert^12^, Anne-Pascale Meert^13^, Dominique Benoit^14^, Rebecca Hamidfar^15^, Merce Jourdain^16^, Elie Azoulay^2^, Djamel Mokart^1^

##### ^1^Réanimation, Institut Paoli-Calmettes, Marseille, France; ^2^Réanimation médicale, Hôpital Saint Louis, Paris, France; ^3^Hématologie, Institut Paoli-Calmettes, Marseille, France; ^4^Médecine intensive Réanimation, Hôpital Cochin, Paris, France; ^5^Réanimation, CHU Angers, Angers, France; ^6^Hépatologie médicale, Hôpital Cochin, Paris, France; ^7^Médecine intensive Réanimation, hôpital Pitié Salpêtrière, Paris, France; ^8^Réanimation, Hôpital Avicenne, Paris, France; ^9^Réanimation, CH ROUBAIX, Roubaix, France; ^10^Réanimation, CH Versailles, Versailles, France; ^11^Réanimation médicale, CHU Brest, Brest, France; ^12^Réanimation médicale, CH La Roche-sur-Yon, La Roche-Sur-Yon, France; ^13^Institut Jules Bordet, Bruxelles, Belgium; ^14^Ghent University Hospital, Gent, Belgium; ^15^Réanimation, CHU Grenoble, Grenoble, France; ^16^Réanimation, Hôpital Roger Salengro, CHU Lille, Lille, France

###### **Correspondence:** Magali Bisbal (bisbalm@ipc.unicancer.fr)

*Ann. Intensive Care* 2020, **10(Suppl 1):**P-081

**Rationale:** Hepatic dysfunction (HD) is commonly observed in patients with hematologic malignancies and associated with an increased mortality in allogeneic hematopoietic stem cell transplantation patients. We aimed to assess incidence, risk factors and prognostic impact of HD in a large multicenter cohort study of critically ill patients with hematologic malignancies.

**Patients and methods:** This research was a post hoc analysis of a Franco-Belgian multicenter prospective study assessing the prognosis of patients with hematologic malignancies admitted to intensive care unit (ICU) between January 2010 and May 2011. HD was defined as serum total bilirubin ≥ 33 µmol/L at ICU admission. For patients with HD, a review of medical hospital records was performed by an expert panel to assess management of HD by attending physicians.

**Results:** Among the 1011 patients with hematologic malignancies admitted to ICU, 893 were included in the study, mainly patients with non-Hodgkin lymphoma (31.5%) or acute myeloid leukemia (27.1%). HD at ICU admission occurred in 183 patients (20.7%). Factors independently associated with HD were the use of cyclosporine (OR = 3.357, 95% CI 1.926–5.851, p < 0.0001) and antimicrobial treatment (OR = 1.578, 95% CI 1.038–2.401, p = 0.033) before ICU admission, abdominal symptoms at ICU admission (OR = 2.182, 95% CI 1.461–3.258, p < 0.0001), ascites (OR = 2.562, 95% CI 1.059–6.196, p = 0.037), hepatic Charlson comorbidity (OR = 2.228, 95% CI 1.057–4.696, p = 0.035), increased creatinine at ICU admission (OR = 1.001, 95% CI 1–1.003, p = 0.02), neutropenia (OR = 1.465, 95% CI 1.001–2.144, p = 0.049) and myeloma (OR = 0.378, 95% CI 0.188–0.761, p = 0.006). Hospital mortality was 56.3% and 36.3% in patients with HD and patients with no HD respectively (p < 0.0001). HD appeared as an independent factor of hospital mortality after adjustment with other organ failure (ORadj = 1.861, 95% CI 1.275–2.717, p = 0.001). Factors independently associated with hospital mortality among patients with HD at ICU admission are reported in Table 1. Etiologic diagnoses for HD by physicians were undetermined for 125 patients (72.2%) including 92 (53.2%) for whom the existence of HD has not even been mentioned in the medical record. Investigations were performed in 29% and only 19% of patients received a specific treatment for HD.

**Conclusion:** HD at ICU admission is common, underestimated, poorly investigated, and impairs outcome in critically ill patients with hematologic malignancies. HD should be considered and managed as other organ dysfunctions. It raises the importance of an early severity assessment of HD and a development of diagnosis strategies to get therapeutic options, in close collaboration between hematologists and intensivists.

**Compliance with ethics regulations:** Yes.

**Table 1:** Factors independently associated with hospital mortality in hematological patients with hepatic dysfunction at ICU admission

	OR	95% CI	p
Hepatic Charlson comorbidity Performance status > 1 Acute respiratory failure at ICU admission Mechanical ventilation during ICU stay Renal replacement therapy during ICU stay Chemotherapy initiated at ICU admission	11.669 2.138 2.227 4.836 2.341 0.225	1.366–99.719 1.036–4.412 1.029–4.822 2.235–10.463 1.082–5.067 0.064–0.791	0.025 0.040 0.042 < 0.0001 0.031 0.020

### P-082 CT findings and association with outcome of critically ill patients with hematological malignancies, acute respiratory failure and respiratory virus detection

#### Amazigh Aguersif^1^, Marion Jaffro^1^, Audrey Fernandez^1^, Alexandre Nguyen^1^, Emilie Berard^1^, Sihem Bouharaoua^1^, Jean Michel Mansuy^1^, Catherine Mengelle^1^, Guillaume Morel^2^, Cyril Cadoz^3^, Laura Platon^4^, Alexis Ferre^5^, Camille Verlhac^6^, Nahema Issa^7^, Laurent Argaud^8^, Guillaume Geri^9^, Danielle Reuter^10^, Stein Silva^1^, Muriel Picard^11^

##### ^1^CHU DE TOULOUSE, Toulouse, France; ^2^CHU DE STRASBOURG, Strasbourg, France; ^3^CHU DE METZ, Metz, France; ^4^CHU DE MONTPELLIER, Montpellier, France; ^5^CH DE VERSAILLES, Versailles, France; ^6^CHU DE CLERMONT-FERRAND, Clermont-Ferrand, France; ^7^CHU DE BORDEAUX, Bordeaux, France; ^8^HOSPICES CIVILS DE LYON, Lyon, France; ^9^APHP, AMBROISE PARE, Paris, France; ^10^Ch Sud Francilien, Corbeil-Essonnes, France; ^11^IUCT-ONCOPOLE, Toulouse, France

###### **Correspondence:** Amazigh Aguersif (aguersif.a@chu-toulouse.fr)

*Ann. Intensive Care* 2020, **10(Suppl 1):**P-082

**Rationale:** Acute respiratory failure (ARF) is the main cause for admission to the ICU for patients with hematological malignancies (HM). Viral pneumonia is poorly described in this population. Respiratory viruses PCR is a rapid and sensitive diagnostic tool. Thoracic CT allows to guide the diagnosis but is also poorly described. The primary objective was to describe CT features suggesting viral pathogenicity. Secondary objectives were to assess risk factors associated with the use of invasive mechanical ventilation (IMV) and ICU mortality.

**Patients and methods:** Retrospective study conducted from January 1, 2008 to April 30, 2018 in 10 French ICUs. Adult patients with HM admitted to the ICU for ARF with a positive respiratory viruses PCR and a thoracic CT performed. Results are reported as median [IQR] or number (%). Multivariate analysis was performed using logistic regression.

**Results:** One hundred and four patients have been included, 60 of whom had viral infections alone (57.7%). Median age was 61 years [IQR 53.5–70.5]. Acute myeloid leukemia (26.0%) and multiple myeloma (22.1%) were the most frequent malignancies; 22.1% were allogenic HSCT recipients. Median IGSII and SOFA score at ICU admission were 56 [IQR 44–67] and 8 [IQR 5–11]. IMV, catecholamines and RRT were required in 67.3% (n = 70), 64.4% (n = 67) and 25.0% (n = 26) of patients. ARDS criteria were met in 54.8% (n = 57) of patients. ICU and hospital mortality rates were 40.4% and 46.2%. Influenza was the most frequently documented virus (41.3%), followed by Respiratory Syncytial Virus (22.1%). Fungal co-infections (25.0%) were more frequent than bacterial coinfections (23.1%). The distribution of bronchial, interstitial and alveolar syndromes was similar. The Orthomyxovirus group had more bronchoalveolar association than the Paramyxovirus group (52.4% versus 15.4%, p < 0.05). In multivariate analysis, an alveolar syndrome was a risk factor for the use of IMV (OR = 2.94, 95% CI 1.00–8.67), in contrast to a bronchial syndrome (OR = 0.27, 95% CI 0.09–0.82). Fungal co-infections were associated with ICU mortality (OR = 3.92, 95% CI 1.07–14.40). The lesional intensity on CT was associated with both the use of IMV (OR = 1.88, 95% CI 1.27–2.80) and death in the ICU (OR = 1.64, 95% CI 1.10–2.44).

**Conclusion:** Bronchoalveolar syndrome was suggestive of Orthomyxovirus infection in our population. Several CT features were associated with the use of IMV and ICU mortality. Prospective studies are needed to confirm these results.

**Compliance with ethics regulations:** Yes.

### P-083 High-dose methotrexate in ICU patients: a retrospective study

#### Sandrine Valade^1^, Lara Zafrani^1^, Eric Mariotte^1^, Virginie Lemiale^1^, Guillaume Dumas^1^, Lauriane Goldwirt^2^, Adrien Mirouse^1^, Yannick Hourmant^1^, Jean Jacques Tudesq^1^, Asma Mabrouki^1^, Elie Azoulay^1^, Michaël Darmon^1^

##### ^1^Medical ICU, Saint Louis hospital, Paris, France; ^2^Department of Pharmacology, Saint Louis hospital, Paris, France

###### **Correspondence:** Sandrine Valade (sandrine.valade@aphp.fr)

*Ann. Intensive Care* 2020, **10(Suppl 1):**P-083

**Rationale:** High-dose methotrexate (HD-MTX) is commonly used in the treatment of solid tumours and hematological malignancies. Severe toxicities are frequent, leading to organ dysfunction, multiple organ failure and death. Outcome of these patients when critical illness occurs is poorly studied. This study aims to describe MTX-induced toxicities and to assess outcome in critically ill patients.

**Patients and methods:** In this retrospective study conducted in the ICU of one university hospital between January 2002 and December 2018, all the patients who were given HD-MTX (single dose greater than 500 mg/m^2^) in the ICU were included. Results are presented as median [interquartile range] and number (percent).

**Results:** 33 patients (24 men and 9 women) aged 48 years [34–63], were included. B-cell lymphoma had been diagnosed in 31 patients (Burkitt, n = 14; diffuse large B cell lymphoma with CNS (central nervous system) involvement, n = 9; primary CNS lymphoma, n = 5) and T-cell lymphoma in two patients. Patients were mainly admitted for coma (n = 14; 42%) or acute kidney injury (n = 8; 24%). MTX was administered at a median dose of 6.1 g [5–14]. Fourteen patients had concomitant medication interacting with MTX. Median MTX clearance was 4 days [4–5]. Frequent MTX-related complication were mucositis (n = 21, 64%), diarrhea (n = 14, 44%) or hepatic failure (n = 15, 45%). During ICU stay, 11 patients experienced acute kidney injury (KDIGO stage 2.5 [2–3]). Two patients received carboxypeptidase and three underwent dialysis. Overall, 19 patients (57%) required mechanical ventilation, 10 (30%) vasopressors. Hospital mortality was 33% (n = 11). Cox model identified MTX concentration 24 h after administration higher than 4.6 µmol/L as associated with hospital mortality (HR 6.7, 95% CI 1.6–27.3) (Fig. 1).

**Conclusion:** To our knowledge this is the first study assessing characteristics and outcome of critically ill patients receiving HD-MTX. MTX concentration at H24 was associated with hospital mortality. Despite underlying malignancy, ICU support of these patients was associated with a meaningful survival.

**Compliance with ethics regulations:** Yes.

**Fig. 1:**
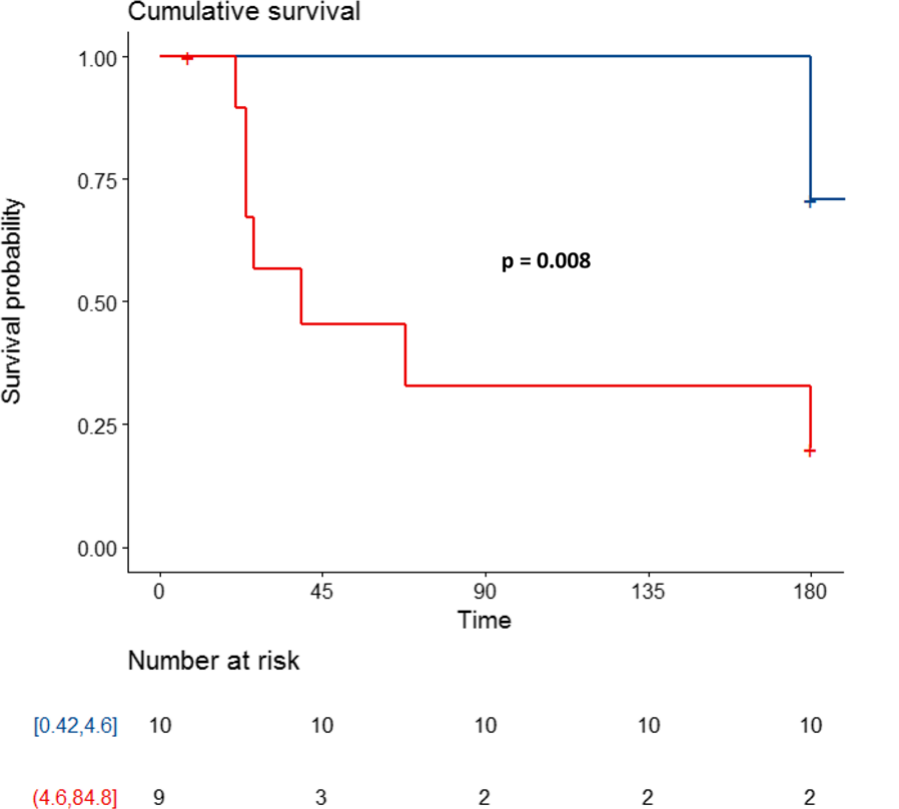
Adjusted influence of MTX dosage at H24

### P-084 Life-threatening complications following high-dose methotrexate and role of glucarpidase

#### Chloé Medrano^1^, Lucie Oberic^2^, Suzanne Tavitian^2^, Florent Puisset^3^, Caroline Protin^4^, Sophie Perriat^3^, Muriel Picard^4^, Francoise Huguet^2^, Sarah Bertoli^2^, Christian Recher^2^, Stanislas Faguer^1^

##### ^1^Department of Nephrology and Organ Transplantation-Intensive Care Unit, University Hospital of Toulouse, Toulouse, France; ^2^Service d’Hématologie, Institut Universitaire du Cancer de Toulouse-Oncopole, Toulouse, France; ^3^Service de Pharmacologie, Institut Universitaire du Cancer de Toulouse-Oncopole, Toulouse, France; ^4^Service de Réanimation, Institut Universitaire du Cancer de Toulouse-Oncopole, Toulouse, France

###### **Correspondence:** Chloé Medrano (chloemedrano@hotmail.fr)

*Ann. Intensive Care* 2020, **10(Suppl 1):**P-084

**Rationale:** High-dose methotrexate (1 g/m^2^; hdMTX) is the cornerstone of chemotherapy in acute lymphoblastic leukemia (ALL) and several high-grade non-hodgkin lymphoma (hNHL). Despite standardized prevention, acute kidney injury (AKI) and other life-threatening complications still occur. Given the cost of glucarpidase, an enzyme that metabolizes MTX in few minutes, and the complexity of hematological patients admitted to the ICU, a better comprehensive view of the factors that predict hdMTX toxicity, as well as the role of glucarpidase as rescue therapy in patients with organ failure, is mandatory.

**Patients and methods:** Retrospective monocenter study including all the adult patients referred for ALL or hNHL in a French university hospital, and who received hdMTX. AKI was defined according to the KDIGO classification. Univariate analysis (Fischer exact or Mann–Withney tests) followed by multivariate analysis (stepwise logistic regression) were used to identify before hdMTX the clinical and biological predictive factors of AKI. Outcomes following glucarpidase were also addressed.

**Results:** From Dec-2011 to Sept-2018, 468 patients received HdMTX (median dose 3 g/m^2^; ALL n = 126, hNHL n = 342), totalizing 1264 hdMTX pulses. Sixty-nine patients (14.7%) developed AKI after a median time of 2 days (stage 1 n = 35, stage 2 n = 18, stage 3 n = 16 including one requiring dialysis in the first week). By multivariate analysis, only age, body mass index and a diagnosis of ALL were significantly and independently associated with the risk to develop AKI. MTX exposure (maximal serum concentration at H24-48) was also associated with AKI (AUC 0.85, p < 0.01). Glucarpidase was used in 42 patients (9%) that differed by a higher age and BMI, and a lower basal eGFR. Glucarpidase was followed by a rapid renal improvement but serum creatinine did not return to baseline (120 vs. 92 micromol/L). Thirty patients with AKI or delayed MTX elimination did not receive glucarpidase but none required renal replacement therapy and eGFR was only slightly but not significantly reduced at the end of follow-up. Extra-renal adverse-events (RBC and platelets transfusions, neutropenia, hepatitis, severe diarrhea, mucitis) were more frequent in patients that developed AKI. Eighteen patients were admitted to the ICU, including 9 and 6 that required mechanical ventilation or vasopressor drugs, respectively.

**Conclusion:** Few actionable factors predict the development of AKI after HdMTX, suggesting additional genetic factors. AKI was reversed by glucarpidase but progression toward CKD was the rule. Further studies will have to identify patients that will actually beneficiate from glucarpidase.

**Compliance with ethics regulations:** Yes.

### P-085 Impact of infection on hepatic encephalopathy during cirrhosis

#### Khaoula Ben Ismail, Sana Khedher, Ameni Khaled, Nassereddine Foudhaili, Mohamed Salem

##### USI Digestif-service de gastroenterologie-EPS Charles Nicolles.Tunis–Tunisie., Tunisia, Tunisia

###### **Correspondence:** Khaoula Ben Ismail (khaoula87@hotmail.fr)

*Ann. Intensive Care* 2020, **10(Suppl 1):**P-085

**Rationale:** Infection is common and accounts for major morbidity and mortality in cirrhosis. Patients with cirrhosis are immunocompromised and have increased susceptibility to develop spontaneous bacterial infections, hospital-acquired infections, and a variety of infections from uncommon pathogens. We aimed to evaluate the impact of infection on hepatic encephalopathy.

**Patients and methods:** This is a prospective study, conducted over a period of 3 years from January 2016 to December 2018. Consecutive patients with approved decompensated cirrhosis admitted to our department are included. All clinical and biological data were collected from the medical records. Univariate and multivariate analysis were used to identify the impact of infection on hepatic encephalopathy.

**Results:** A total of 110 patients diagnosed with decompensated cirrhosis were enrolled in this study. Mean of age was 62 years (18–88). Sex ratio was 1.2. HCV (39%) was the main etiology of cirrhosis. The reasons of hospitalization were: oedema with ascitic syndrome (44% of cases), digestive bleeding (21% of cases), fever (16% of cases), and encephalopathy (36% of cases). Patients with infection seemed to have a high incidence of hepatic encephalopathy with 64% versus 36% when the patients are none infections. The results also showed that in those with hepatic encephalopathy, an effective antibiotic treatment accelerates significantly wakefulness under 48 h with a rate of 76% vs. 24% (p < 0.03) . In addition, the infection does not influence mortality or length of stay compared to other complications such as digestive bleeding.

**Conclusion:** We found that infection caused more episodic hepatic encephalopathy than other complication and an effective antibiotherapy accelerate wakefulness.

**Compliance with ethics regulations:** Yes.

### P-086 Lactulose vs. polyethylene glycol electrolyte solution for treatment of hepatic encephalopathy

#### Khaoula Ben Ismail, Sana Khedher, Ameni Khaled, Nassereddine Foudhaili, Mohamed Salem

##### USI Digestif-service de gastroenterologie-EPS Charles Nicolles.Tunis –Tunisie., Tunisia, Tunisia

###### **Correspondence:** Khaoula Ben Ismail (khaoula87@hotmail.fr)

*Ann. Intensive Care* 2020, **10(Suppl 1):**P-086

**Rationale:** Hepatic encephalopathy (HE) is a common cause of hospitalization in patients with cirrhosis. Pharmacologic treatment for acute (overt) HE has remained the same for decades. To compare polyethylene glycol electrolyte solution (PEG) and lactulose treatments in patients with cirrhosis admitted to the hospital for HE. We hypothesized that rapid catharsis of the gut using PEG may resolve HE more effectively than lactulose.

**Patients and methods:** This is a prospective study, conducted over a period of 3 years. From Janury 2016 to December 2018, we have been interested in cirrhotic patients with hepatic encephalopathy. All clinical and biological data were collected from the medical records. Univariate and multivariate analysis were used to identify the difference beteween PEG and lactulose in the treatement of hepatic encephalopathy.

**Results:** A total of 110 patients diagnosed with decompation of cirrhosis were enrolled in this study. Mean of age was 62 years (18–88). Sex ratio was 1.2. HCV (39%) was the main etiology of cirrhosis. The hospitalization reasons were: edematous-ascitic syndrome in 44%, gastro-intestinal bleeding 21%, fever in 16%, and encephalopathy was present in 36% of cases. A total of 40 patients were randomized to each treatment arm. Baseline clinical features at admission were similar in the groups. Twelve of 20 patients in the standard therapy arm (55%) had an improvement of 1 or more in HESA score, thus meeting the primary outcome measure, compared with 17 of 20 evaluated patients receiving PEG (85%) (p < 0.01). The mean ± SD HESA score at 24 h for patients receiving standard therapy changed from 2.3 ± 0.9 to 1.6 ± 0.9 compared with a change from 2.3 ± 0.9 to 0.9 ± 1.0 for the PEG-treated groups (p = 0.002). The median time for HE resolution was 2 days for standard therapy and 1 day for PEG (p = 0.01). Adverse events were uncommon, and none was definitely study related.

**Conclusion:** We found that PEG led to more rapid HE resolution than standard therapy, suggesting that PEG may be superior to standard lactulose therapy in patients with cirrhosis hospitalized for acute HE.

**Compliance with ethics regulations:** Yes.

### P-087 Acute pancreatitis and pregnancy

#### Janati Adnane, Lina Berrada

##### Obstetric intensive care unit, Casablanca, Morocco

###### **Correspondence:** Janati Adnane (adnanejanati@gmail.com)

*Ann. Intensive Care* 2020, **10(Suppl 1):**P-087

**Rationale:** The association of acute pancreatitis and pregnancy is rare but not negligible, it often cause a diagnostic problem given the gravidal context that can lead to serious repercussions. The objective of our study is to assess the particularities in the diagnosis, management and prognosis of acute pancreatitis during pregnancy

**Patients and methods:** This is a retrospective study about cases of acute pancreatitis occurred during pregnancy over a 3-year period (2017–2019) at the obstetric intensive care unit of the MERIEM maternity hospital in the CHU IBN ROCHD CASABLANCA. A retrospective analysis of the medical files of these patients was carried out, considering epidemiological and etiological criteria, the treatments administered and maternal/fetal fate.

**Results:** We found 10 cases during this period, with an incidence of 1/3000. The average age of onset was 28 years, 90% of cases occurred in the 3^rd^ trimester. Epigastric pain and vomiting were the common symptomatology. Ultrasound showed biliary lithiasis in 70% of cases with increased pancreas size in 30% of cases. Maternal mortality was zero. Uncomplicated benign forms are the most common (80%). Severe hypokalemia was found in 40% of patients. Neonatal morbidity was marked by six premature deliveries. Among them, a newborn died at day-1 of life

**Discussion:** The association of acute pancreatitis and pregnancy is rare, more frequent during the 3^rd^ trimester, it mainly affects the young woman. Lithiasic biliary pathology remains by far the most frequent etiology. The diagnosis is clinical most often represented by epigastralgia with vomiting and biological via lipasemia and amylasemia dosage. Uncomplicated benign forms are the most common. Hydroelectrolytic disorders are often found. Abdominal ultrasound allows the etiological diagnosis. The treatment is above all symptomatic whose objective is the digestive rest, the correction of the hydroelectrolyte disorders but first of all relieve the pain.

**Conclusion:** Acute pancreatitis is a rare event in pregnant women, but can have a maternal and fetal prognosis. It must be systematically evoked in front of the acute abdominal pains of the pregnant woman because the confirmation of the diagnosis is easy and the maternal results depend mainly on therapeutic management. Prematurity remains the predominant factor in neonatal morbidity.

**Compliance with ethics regulations:** Not applicable.

### P-088 Selecting patients with decompensated cirrhosis and acute-on-chronic liver failure (ACLF) admitted to the intensive care unit for liver transplantation

#### Sophie-Caroline Sacleux, Elise Lemaitre, Marc Boudon, Philippe Ichaï, Didier Samuel, Faouzi Saliba

##### AP-HP Hôpital Paul-Brousse, Centre Hépato-Biliaire, Liver Intensive Care Unit, Villejuif, France

###### **Correspondence:** Sophie-Caroline Sacleux (sophie-caroline.sacleux@aphp.fr)

*Ann. Intensive Care* 2020, **10(Suppl 1):**P-088

**Rationale:** ACLF is a clinical concept defined in patients with chronic liver disease who presented organ failure(s) secondary to an acute decompensated event. Liver transplantation in this indication showed good results in selected patients. The aim of this prospective study was to evaluate the outcome and the factors associated with a favorable selection to liver transplantation in this population.

**Patients and methods:** All consecutive patients admitted to the ICU with cirrhosis and ACLF, were recruited. Patient with age < 18 years or with fulminant hepatitis were excluded.

**Results:** Between July 2017 and February 2019, 155 cirrhotic patients were admitted to ICU. Mean age was 55.6 ± 11.3 years (71.6% Male). Cirrhosis was due to alcohol in 78.1% of the patients. ACLF grading at admission was: 44.5% ACLF3 (n = 60), 21.3% ACLF2 (n = 33), 14.8% ACLF1 (n = 23), and 19.4% ACLF0 (n = 30). Of the 155 patients, 46.5% (n = 72) were considered to be eligible for a transplant project and were assessed for liver transplantation. The main reasons were alcohol abuse (66.3%, n = 55), death within 7 days after admission (32.5%, n = 27) and rapid improvement of the liver disease. Of the eligible patients, 50% were transplanted with a mean time between admission to ICU and liver transplantation of 68.9 ± 105.1 days. Twelve patients died on the waiting list (24% of the listed patients), mainly of septic shock. Among those who were assessed for liver transplantation but not listed (n = 21), 76.2% died before the listing (n = 16) and 23.8% were not listed because of severe comorbidities (n = 5). The global mortality rate was 56.8% (n = 88). The 28 and 90 days rate mortality were respectively 42.9% and 56.2%. The overall 3-month patient survival was respectively 97% and 26% in the transplant and non-transplant group (p < 0.001) for the entire cohort. Among eligible patients, factors associated with the absence of liver transplantation, in the multivariate analyses, were mechanical ventilation (HR 8.95, 95% CI 2.75–29.06, p < 0.001) and age over 60 years (HR 3.32, 95% CI 1.04–10.63, p < 0.001).

**Conclusion:** Cirrhotic patients admitted to the ICU should be evaluated for eligibility to liver transplantation. Merely half of the patients were eligible/assessed and 22% patients were transplanted. Among those eligible, patients over 60 years and under mechanical ventilation during early ICU stay would less likely to survive and be selected for liver transplantation.

**Compliance with ethics regulations:** Not applicable.

### P-089 Interest of body composition analysis in CT in cirrhotic patients with septic shock

#### Caroline Lemaitre^1^, Steven Grange^2^, Mathieu Devilder^3^, Dorothée Carpentier^2^, Gaetan Beduneau^2^, Christophe Girault^2^, Fabienne Tamion^2^, Celine Savoye Collet^3^

##### ^1^Hepato-gastroenterology Unit, CHU ROUEN, Rouen, France; ^2^Medical Intensive Care Unit, CHU ROUEN, Rouen, France; ^3^Radiology Unit, CHU ROUEN, Rouen, France

###### **Correspondence:** Caroline Lemaitre (caro.lemaitre@wanadoo.fr)

*Ann. Intensive Care* 2020, **10(Suppl 1):**P-089

**Rationale:** Body composition is known to be a prognostic factor in cirrhotic patients. However, the link between this and the prognosis of patients in intensive care unit (ICU) is unknown. The computed tomography offer accurate estimations of muscle mass by analysing a cross-section usually going through the third lumbar vertebrae. This retrospective study aimed to assess the feasibility of body composition (BC) analysis in cirrhotic patients with septic shock, using Computed Tomography (CT) and evaluate the impact of BC (muscle mass, subcutaneous and visceral fat) on outcome.

**Patients and methods:** This retrospective study included 36 cirrhotic patients with septic shock hospitalized in ICU who underwent an abdomino pelvic CT scan within 48 h of admission. We collected the surface areas of muscle mass and adipose tissue on the CT scans. We compared BC data with mortality and with the number of organ failures.

**Results:** The average age was 60 years [42–73]. The average Child and MELD scores were respectively 10.8 [8–14] and 28.7 [15–54]. The prevalence of sarcopenia was 50%. It was not associated with a higher mortality rate at day 28 (p = 0.31) or with a higher number of organ failures at day 28 (p = 0.55). We observed a higher subcutaneous adiposity index in patients who died at day 28 (p = 0.03) and in patients with renal insufficiency at admission (p = 0.019). There was a trend (p = 0.057) towards more visceral fat in patients who died in ICU.

**Conclusion:** The assessment by CT of body composition reveal evaluation of BC using CT is feasible and reproducible and may constitute a promising tool to evaluate in cirrhosis critically ill patients. Visceral fat mass seems associated with poor outcome in cirrhotic patients with septic shock

**Compliance with ethics regulations:** Yes.

### P-090 Comparative study between superinfection of necrosis lapses in post-ERCP pancreatitis and other etiologies of pancreatitis

#### Rachid Jabi, Mohammed Bouziane

##### CHU Mohammed VI, Oujda, Morocco

###### **Correspondence:** Rachid Jabi (jabirachid@gmail.com)

*Ann. Intensive Care* 2020, **10(Suppl 1):**P-090

**Rationale:** The infection of the necrosis constitutes a pejorative element in the management of the necrotico-haemorrhagic pancreatitis, in the absence of the drainage the mortality approaches 100%. The morbidity and mortality of surgery can be avoided with minimally invasive treatments.

**Purpose:** to compare the morbidity and mortality of the two groups of post-ERCP pancreatitis and the other etiologies.

**Patients and methods:** A retrospective study over 4 years between 2016 and 2019 and a comparison between pancreatitis secondary to post-ERCP and other etiologies of pancreatitis. A P value of 0.05 is considered significant.

**Results:** The surgical treatment used in 10 cases of superinfection post ERCP against seven cases of other etiologies of pancreatitis. High mortality in post-ERCP pancreatic arm 88% vs. 50% (p = 0.09). High morbidity in the operated group 66% vs. 28% (p = 0.04) represented mainly digestive haemorrhages. Duration of stay was significantly longer in the operated group 36 vs. 12 days (p = 0.04). Thrombocytopenia and beta-lactamase-producing enterobacteria have further complicated management in the post-ERCP infected pancreatitis arm. The antibiotic resistance of infected pancreatitis in post-ERCP patients is 86.65% for ciprofloxacin, 19.17% for imipenem and 65% for amikacin.

**Conclusion:** Pancreatitis the most common adverse effect of ERCP with significant morbidity and mortality. The collaboration between the intensive care unit gastroenterologist and the surgeon improves management since the risk factors are mainly related to the patient and can not be modified.

**Compliance with ethics regulations:** Yes.

### P-091 Determinants of early morbidity and mortality after liver transplantation from a retrospective cohort of 229 patients

#### Gautier Nitel, Aghiles Hamroun, Anne Bignon, Gilles Lebuffe

##### CHRU Lille, Lille, France

###### **Correspondence:** Gautier Nitel (gautier.nitel@gmail.com)

*Ann. Intensive Care* 2020, **10(Suppl 1):**P-091

**Rationale:** Liver transplantation (LT) has been recently experiencing an expansion of its indications, allowing patients with potentially more co-morbidities to access to transplantation. In our era of graft shortage, we should focus on the identification of the best LT candidates. The aim of our work is to study the determinants of early morbidity and mortality after LT from three angles: occurrence of a major cardiovascular event (MACE) or acute renal failure (KDIGO stage 2–3 AKI) in the first 30 days postoperative, and death in the year following LT.

**Patients and methods:** Retrospective study investigating the occurrence of MACE or AKI (KDIGO 2–3) within 30 days post-operative and mortality at 1 year after LT, including patients who received a first LT between January 2014 and December 2017 in our center. Analysis of risk factors by a multivariate step-by-step analysis. Statistical significance for p < 0.05. Data presented in Odds ratio (OR) or Hazard ratio (HR) and 95% confidence interval (OR/HR [95% CI]).

**Results:** Among 229 LT patients, 12.7% presented a MACE and 47% experienced an AKI within 30 days postoperative, with 11% of deaths at 1 year after LT. The MELD score is associated with postoperative morbidity with an increased risk of stage 2–3 AKI (OR 1.06 [1.03–1.10] p < 0.001) and MACE (OR 1.06 [1.01–1.11] p = 0.023). Mortality in the first year after LT appeared to be strongly impacted by the occurrence of stage 2–3 AKI in the first postoperative month (HR 6.95 [2.30–20.99] p < 0.001), with a trend, yet not significant, for the occurrence of a MACE (HR 2.17 [0.86–5.46] p = 0.10).

**Conclusion:** The preoperative MELD score is associated with early postoperative morbidity including 30d-AKI and MACE, which significantly influences mortality in the first year after LT.

**Compliance with ethics regulations:** Yes

### P-092 Usefulness of systematic blood culture under veno-arterial ECMO

#### Quentin De Roux^1^, Marie Renaudier^1^, Wulfran Bougouin^2^, Johanna Boccara^1^, Baptiste Dubost^1^, Vincent Fihman^3^, Raphael Lepeule^3^, Antonio Fiore^4^, Jean-Winoc Decousser^3^, Olivier Langeron^1^, Nicolas Mongardon^1^

##### ^1^Service d’anesthésie-réanimation chirurgicale, réanimation chirurgicale cardio-vasculaire, DMU CARE, Assistance Publique des Hôpitaux de Paris (APHP), Hôpitaux Universitaires Henri Mondor, Créteil, France; ^2^Réanimation polyvalente, Hôpital Privé Jacques Cartier, Massy, France; ^3^Service de microbiologie-bactériologie-hygiène, Assistance Publique des Hôpitaux de Paris, Centre Hospitalier Universitaire Henri Mondor, Créteil, France; ^4^Service de chirurgie cardiaque, Assistance Publique des Hôpitaux de Paris, Centre Hospitalier Universitaire Henri Mondor, Créteil, France

###### **Correspondence:** Quentin De Roux (deroux.q@gmail.com)

*Ann. Intensive Care* 2020, **10(Suppl 1):**P-092

**Rationale:** Infectious complications are frequently reported in critically ill patients supported by Veno-Arterial ExtraCorporeal Membrane Oxygenation (VA-ECMO) for refractory cardiogenic shock, but their diagnosis is challenging. No study has specifically studied bloodstream infection (BSI) in this population and some recommendations suggest performing systematic blood culture (BC). In our unit, systematic BC are daily sampled. We investigated the interest of systematic BC to detect BSI under VA-ECMO.

**Patients and methods:** In a retrospective analysis (2013–2017), and after exclusion of patients dying within 24 h, all adult patients from cardio-vascular intensive care unit supported by VA-ECMO were included. Systematic daily and “on demand” BC (at the physician’s discretion) performed from VA-ECMO implantation to 5 days after withdrawal were analyzed. BSI was defined as at least one BC positive to a pathogen (except for contaminants BSI which required at least two positive BC with the same bacteria in 48 h). Multivariable logistic regression was performed to identify risk factors for positivity of systematic BC.

**Results:** One hundred and fifty VA-ECMO (65 after cardiac surgery and 85 for medical etiology, including 39 after refractory cardiac arrest) were included, representing 1422 ECMO-days. Median age was 58 years [48–69] and SAPS II was 54 [38–70]. On 2163 BC performed (1162 systematic BC and 984 “on demand” BC), 192 were positive, including 68 BC (35%) with contaminants and seven BC (4%) with yeasts. Regarding systematic BC, 51 revealed BSI (4%); conversely, 71 “on demand” BC revealed BSI (7%). Sixty different BSI episodes were observed, for a BSI episode rate of 43 cases/1.000 days of ECMO support. In multivariable analysis, performing systematic BC was negatively associated with diagnosing BSI (OR 0.55, 95% CI 0.38–0.81, p = 0.002). Independent risk factors for BSI diagnosis thanks to systematic BC were: VA-ECMO for primary graft failure (OR 2.43, 95% CI 1.20–4.92, p = 0.013) and BC performed under antimicrobial therapy or renal replacement therapy (OR 2.15, 95% CI 1.08–4.27, p = 0.029 and OR 2.05, 95% CI 1.10–3.81, p = 0.008, respectively). On the 36 systematic BC positive for contaminants, five led to antimicrobial therapy initiation, thus deemed as inappropriate antibiotherapy. Mortality rate did not differ between patient with or without BSI (60 vs. 54% p = 0.49).

**Conclusion:** Systematic BC are less useful than “on demand” BC. Positive systematic BC for contaminant lead to inappropriate antimicrobial prescription in a significant proportion of samples. This argues for switching toward a reasonable approach of “on demand” BC under VA-ECMO, according to risk factors of BSI.

**Compliance with ethics regulations:** Yes.

### P-093 Catheterization and fungal infection risk

#### Nabil Mosbah, Habiba Hemamid, Abdelmalek Hakimi

##### CHU Sétif, Sétif, ALGERIA

###### **Correspondence:** Nabil Mosbah (mosbahnabil05@gmail.com)

*Ann. Intensive Care* 2020, **10(Suppl 1):**P-093

**Rationale:** Fungal infections are constantly increasing in hospitals. Indeed, the increase in these infections and especially candida yeast infections is almost parallel to the increase in the widespread use of a wide range of implanted medical devices such as catheters. For this reason, we have been investigating, isolating and identifying Candida yeast colonizing vascular catheters and studying the epidemiological and clinical characteristics of patients with colonized catheters.

**Patients and methods:** It is a prospective, transversal study conducted at the intensive care and neurosurgery services of the Sétif University Hospital, evaluating the fungal colonization of vascular catheters. These are collected from hospitalized patients for a period of 5 months. A culture of the distal end of the catheter is performed directly after its ablation.

**Results:** The results obtained showed that among the 30 samples taken, six are colonized by the yeasts, the incidence is 20%. Six yeast of candida spp were isolated, 50% of them were *Candida albicans* species, 33.33% *Candida parapsilosis* and 16.66% were *Candida glabrata*.

**Conclusion:** It appears that colonization of catheters occurs most frequently in patients with the following characteristics: extreme ages of life, male sex, antibiotic therapy and length of hospitalization or prolonged catheterization.

**Compliance with ethics regulations:** Yes.

### P-094 Molecular characterization and resistance profile of emergent extensively drug-resistant bacteria in burn patients

#### Emna Hammami^1^, Sarra Dhraief^1^, Hana Fredj^2^, Sajida Sboui^2^, Amenallah Messaadi^2^, Lamia Thabet^1^

##### ^1^Centre de Traumatologie et des Grands Brûlés de Ben Arous-Laboratoire de biologie médicale et banque du sang, Ben Arous, Tunisia; ^2^Centre de Traumatologie et des Grands Brûlés de Ben Arous-Service de réanimation des brûlés, Ben Arous, Tunisia

###### **Correspondence:** Emna Hammami (emnahammami1993@gmail.com)

*Ann. Intensive Care* 2020, **10(Suppl 1):**P-094

**Rationale:** The threat of emergent extensively drug-resistant bacteria (eXDR) dissemination worldwide is real. It has become a global public health issue. In fact, Glycopeptides-resistant *Enterococcus faecium* (GRE) and carbapenemase-producing *Enterobacteriaceae* (CPE) are the lead microorganisms in the high resistant bacteria category. The aim of our study was to characterize the molecular mechanisms and to determinate the antimicrobial susceptibility profiles of GRE and CPE isolated from burn patients.

**Patients and methods:** Prospectively, we studied all CPE and GRE strains isolated from burn patients between January and December 2018. All isolated microorganisms were identified on the basis of conventional microbiological techniques. Antibiotic susceptibility testing was carried out by the agar disc diffusion method, and susceptibility results were interpreted using clinical breakpoints according to CA-SFM guidelines. Molecular characterization was performed by multiplex real-time PCR (Cepheid, GeneXpert^®^) allowing detection of the most prevalent carbapenemase encoding genes (blaVIM, blaNDM, blaIMP, (blaOxa-48 and blaKPC) as well as the genes VanA and VanB of GRE.

**Results:** During the study period, 53 eXDR were isolated from 42 burn patients. The most frequent sites of isolation were blood cultures (34%) and skin samples (30.2%). CPE represented 86.8% of isolated eXDR (46 strains). Among them, the most frequently identified species was *Klebsiella pneumoniae* (73.9%) followed by *Enterobacter cloacae* (13%). Twenty-four CPE (52.2%) expressed the blaNDM gene. The blaOxa-48 gene was found in 12 strains (26.1%) and ten strains (21.7%) carried both genes. Of the 46 CPE, 89.1% revealed ertapenem MIC > 1 mg/l whereas most strains were susceptible to imipinem and meropenem with 79.5% and 60.9% of susceptibility, respectively. The antibiotics showing the highest resistance rates were cefotaxime (78.3%), piperacillin-tazobactam (97.8%), ciprofloxacin (78.3%) and amikacin (42.9%). The most active agents were colistin and fosfomycin with 6.5% of resistance for each. Seven strains of GRE were isolated (13.2% of eXDR). All of them expressed the VanA gene, with vancomycin MIC > 256 mg/l. However, teicoplanin MICs ranged from 16 to 64 mg/l. All GRE strains were beta-lactam resistant and highly resistant to aminosides. Linezolid and tigecycline were the only active antibiotics.

**Conclusion:** The dissemination of these extensively drug-resistant bacteria must be contained by implementation of strict isolation methods and better hygienic procedures in order to limit their economical and health consequences.

**Compliance with ethics regulations:** Yes.

### P-095 *Stenotrophomonas maltophilia* infections in intensive care unit: clinical features, management and outcome

#### Myriam Kallel, Samia Ayed, Amira Jamoussi, Dhouha Lakhdhar, Jalila Ben Khelil, Mohamed Besbes

##### Abderrahmen Mami Hospital, Ariana, Tunisia

###### **Correspondence:** Myriam Kallel (myriamkallel1991@gmail.com)

*Ann. Intensive Care* 2020, **10(Suppl 1):**P-095

**Rationale:** *Stenotrophomonas maltophilia* has emerged as an important pathogen that induces nosocomial infections. It is a non-fermentative, gram-negative bacillus that causes severe infectious diseases, particularly bacteremia in the hospital setting. Morbidity and mortality due to *Stenotrophomonas maltophilia* seems to be high, particularly in critically ill patient. The aim of this study was to describe the clinical features, management and outcome of patients with *Stenotrophomonas maltophilia* infections.

**Patients and methods:** This was a retrospective analysis of prospectively collected data of patients hospitalized in intensive care unit (ICU) between January 2010 and December 2018. Collected data were: age, gender, comorbidities, severity scores on admission, prior infections, use of antibiotics, use of invasive devices (urinary tract catheter, or mechanical ventilation), microbiological data, and antimicrobial therapy and outcome.

**Results:** During the study period, 27 patients with *Stenotrophomonas maltophilia* infection were included, with a mean age of 51 ± 17 years. The simplified acute physiology score II and acute physiology and chronic health evaluation II on admission were respectively 35 ± 15 and 20 ± 8. Bacteremia caused by *Stenotrophomonas maltophilia* was observed in 25 patients (92%) and ventilator acquired pneumonia in two patients (8%). Twenty four episodes were classified as primary bacteraemia and only one as secondary bacteraemia due to urinary infection. Four patients (15%) developed septic shock. Mean SOFA on the day of *Stenotrophomonas maltophilia* infection was 6 ± 2. Prior antibiotic use was observed in 85% including an antipseudomonal agent in 26% of cases. Infection due to *Stenotrophomonas maltophilia* was considered in 26 cases. Empiric antibiotic therapy was administered to 13 patients (48%) and had included an appropriate agent in only five cases (38%). After adapting antibiotics, monotherapy was the choice for six (25%) patients while a combination of two antibiotics was indicated in the 20 others (75%). The most used antibiotic was the colistin in 11 episodes (46%). Intensive care mortality was 37%. Univariate comparison between dead and survivors showed a significant difference in prior nosocomial infection and respiratory comorbidities. No independent risk factor of mortality was found in multivariate analysis.

**Conclusion:** Management of infection caused by *Stenotrophomonas maltophilia* is a difficult challenge for physicians. Our data showed that prior infections and use of antibiotics may considerably increase mortality in infection with *Stenotrophomonas maltophilia*. Further prospective studies with larger number of patients should be undertaken for definitive conclusions.

**Compliance with ethics regulations:** Yes.

### P-096 Incidence and prognostic impact of thrombocytopenia in septic shock: a prospective study

#### Rania Ammar Zayani, Karama Bouchaala, Sabrine Bradai, Chokri Ben Hamida, Mabrouk Bahloul, Mounir Bouaziz

##### Habib bourguiba university hospital, Sfax, Tunisia

###### **Correspondence:** Rania Ammar Zayani (rania.ammarzayani@gmail.com)

*Ann. Intensive Care* 2020, **10(Suppl 1):**P-096

**Rationale:** Thrombocytopenia is a frequent disorder in critically ill patients, and several studies have reported its correlation with poor prognosis. Considering the major role of platelets in hemostasis, a significant drop in platelet count is an alarming sign in septic patients. The aim of this study was to show the relationship between thrombocytopenia and platelet level changes and mortality in septic patients.

**Patients and methods:** Patients with criteria for septic shock (based on the Third International Consensus definitions for Sepsis and Septic Shock) at admission or at any time during hospitalization were included in a prospective study conducted for a period of 8 months (January 1–August 31, 2018) in a medical surgical intensive care unit. Patients hospitalized for less than 24 h were excluded. Thrombocytopenia was defined as a platelet count less than 150.000/mm^3^, and recovery was defined as returning to levels more than 150.000/mm^3^ after presenting thrombocytopenia. We assessed the platelet count during the hospitalization and its outcomes.

**Results:** We included 82 patients. The mean ± SD age was 56.54 ± 18.45 years. Sex ratio was 1.92. Thrombocytopenia during sepsis (Group 1) was found in 46 patients (56%) with a mortality rate at 85%. The mortality rate among patients not showing thrombocytopenia (Group 2) was significantly lower 26% (p = 0.01). The receiver operating characteristic showed that in (Group 1), a drop in the platelet count (from admission to septic shock day) more than 45% was associated with poor outcome (sensibility = 56%, specificity = 88%, AUC = 0.82). Among the (Group1), 30% showed recovered platelet counts. The mortality was significantly higher in the patients with uncovered thrombocytopenia (90% vs. 28%, p = 0.001).

**Conclusion:** Thrombocytopenia was shown to be an indicator of poor prognosis in our study. In addition, drops of > 45% and failure to recover the platelet counts were further determinants of unfavorable outcomes.

**Compliance with ethics regulations:** Yes.

### P-097 Epidemiological profile and antibiotic susceptibility of *Pseudomonas aeruginosa* isolates in a trauma and burn center in Tunisia

#### Mehdi Gaddas^1^, Sarra Dhraief^1^, Karim Mechri^1^, Imen Jami^2^, Amenallah Messaadi^2^, Lamia Thabet^1^

##### ^1^Centre de Traumatologie et des Grands Brûlés de Ben Arous-Laboratoire de biologie médicale et banque du sang, Ben Arous, Tunisia; ^2^Centre de Traumatologie et des Grands Brûlés de Ben Arous-Service de réanimation des brûlés, Ben Arous, Tunisia

###### **Correspondence:** Mehdi Gaddas (gaddasmehdi@icloud.com)

*Ann. Intensive Care* 2020, **10(Suppl 1):**P-097

**Rationale:** *Pseudomonas aeruginosa* is known as an opportunistic pathogen frequently causing serious infections. Multidrug resistance in this bacterium is increasing worldwide and poses a major problem in the treatment of infections due to this microorganism. Analysis of resistance profile to antibiotics of *P. aeruginosa* helps to establish a prompt control and prevention program. The aim of this study was to evaluate epidemiological profile and antimicrobial resistance of *P. aeruginosa* isolates in a trauma and burn center.

**Patients and methods:** Retrospectively, we studied all *P. aeruginosa* isolates over a 7-year period (from January 2012 to December 2018). Conventional methods were used for identification. Antimicrobial susceptibility testing was performed with disk diffusion method and susceptibility results were interpreted using clinical breakpoints according to CA-SFM guidelines. Data were analyzed using the SIR-system. Minimum inhibitory concentration of colistin was determined using the E-Test^®^ method (bioMérieux), then using the EUCAST broth micro-dilution method (UMIC, Biocentric^®^) since May 2017.

**Results:** During study period, 1499 non-repetitive strains of *P. aeruginosa* were isolated, representing 12% of all isolates. In our center, infections due to *P. aeruginosa* were endemic with epidemic peaks. *P. aeruginosa* was mainly isolated from burn intensive care unit (73.4%) and anesthesiology department (11.1%). The most frequent sites of isolation were skin samples (35.1%), blood cultures (19.7%), catheters (12.5%) and urines (10.3%). The survey of antibiotic susceptibility showed high percentage of resistance to the different antibiotics: 33.8% of strains were resistant to ceftazidime, 65% to ticarcillin, 57.5% to pipercaillin-tazobactam, 57% to imipenem, 56.8% to ciprofloxacin and 68% to gentamicin. Resistance to colistin was rare. It concerned only four strains, isolated from burn patients. The survey of antibiotic susceptibility evolution have shown a global increase of resistance to commonly prescribed antibiotics between 2012 and 2018: from 48% to 57.2% to imipenem, from 47.5 to 61.8% to ticarcillin-clavulanate, from 7.3% to 49% to ceftazidime and from 59.2 to 65% to gentamicin. Whereas ciprofloxacin resistance rate have decreased from 60.9 to 55%. Antibiotic resistant strains were mainly isolated from burn intensive care unit, with 66% of resistance to imipenem and 40.2% to ceftazidime.

**Conclusion:** The dissemination of multidrug-resistant strains of *P. aeruginosa* in our center must be contained by the implementation of strict isolation methods and better hygienic procedures.

**Compliance with ethics regulations:** Yes.

### P-098 *Acinetobacter baumanii*: therapeutic impasse

#### Sabah Benhamza, Mohamed Lazraq, Abdelhak Bensaid, Youssef Miloudi, Najib El Harrar

##### Réanimation de l’hôpital du 20 Août, Casablanca, Morocco

###### **Correspondence:** Sabah Benhamza (benhamzasabah5@gmail.com)

*Ann. Intensive Care* 2020, **10(Suppl 1):**P-098

**Rationale:** *Acinetobacter baumanii* (AB) is frequently responsible for nosocomial infection in the intensive care units, and its resistance to antibiotics continues to increase. The objective of our study is to determine the epidemiological profile and antibiotic sensitivity of isolated bacteria in the intensive care unit August 20, in order to optimize the probabilistic antibiotherapy of bacteremia in intensive care.

**Patients and methods:** This is a retrospective study performed in the intensive care unit of the hospital August 20, 1953, spread over a period of 3 years from January 2016 to January 2019.

**Results:** The incidence of AB infection in our department was 4.77% for all patients admitted to intensive care. The average age was 45 years ± 20, male predominance (sex ratio 2.42). The average time to onset of infection was 11 days. During the study period, 41 AB strains were isolated, 70% of which were pulmonary, 20% blood, and 8% urinary. Resistance to C3G reached 96% in 2016, 98% in 2017 and 99% in 2018. For imipenem resistance was 81% in 2016, 85% in 2017, 76% in 2018. For amikacin, resistance was 41% in 2016, 43% in 2017, and 35% in 2018. For fluoroquinolones resistance was 75% in 2016, 68% in 2017 and 74% in 2018. Cotrimoxazole resistance was around 30% in the last 3 years

**Conclusion:** The resistance of AB to antibiotics has reached very alarming levels, especially for carbapenems. This requires resuscitators to change their antibiotic prescription behavior and to invest in the prevention of nosocomial infections.

**Compliance with ethics regulations:** Yes.

### P-099 Clinical variables predicting intensive care unit transfer and death in patients with suspected infection managed at the emergency department

#### Hadil Mhadhbi, Khedija Zaouche, Abdelwahab Mghirbi, Radhia Boubaker, Kamel Majed

##### Emergency Department la Rabta, Tunis, Tunisia

###### **Correspondence:** Hadil Mhadhbi (hadil.mhadhbi@gmail.com)

*Ann. Intensive Care* 2020, **10(Suppl 1):**P-099

**Rationale:** Determining which patients with suspected infection will deteriorate is a challenge faced by emergency physicians. We aimed to study the factors associated with poor outcomes in patients with suspected infection.

**Patients and methods:** This is a prospective observational study conducted at the ED during the period of 1 year. Data of all patients admitted with suspected infection of any cause were collected. Poor outcomes were defined as death and transfer to an ICU within 48 h.

**Results:** During the study period, a total of 200 patients with a mean age of 68 ± 11 were included. 55% were male. Within 48 h of management in the ED, 10% of patients were transferred to the ICU and 5% died. Independent predictors of ICU-transfer and death included low systolic blood pressure, fever and tachycardia. A prediction model containing these independent predictors had a good predictive accuracy with an area under the curve of 0.89 (95% CI 0.8403–0.9296). Sensitivity was 63%, specificity 93%, positive predictive value 72% and negative predictive value 90%.

**Conclusion:** Assessing readily available clinical variables at arrival to the ED can aid in predicting poor outcomes.

**Compliance with ethics regulations:** Yes.

### P-100 Diaphragmatic contractile function between supine position versus prone position in ventilated patients

#### Ahlem Trifi, Cyrine Abdennebi, Foued Daly, Asma Mehdi, Yosr Touil, Sami Abdellatif, Salah Ben Lakhal

##### Medical ICU la Rabta hospital, Tunis, Tunisia

###### **Correspondence:** Ahlem Trifi (trifiahlem2@gmail.com)

*Ann. Intensive Care* 2020, **10(Suppl 1):**P-100

**Rationale:** To evaluate the effect of the positioning from the supine position (SP) to the prone position (PP) on the contractile function of the diaphragm in ventilated patients; using the ultrasound (US) imaging.

**Patients and methods:** Comparative prospective study of paired series. The consent and approval were obtained by the patient’s parents and the local ethics committee. Were included 40 ICU patients receiving invasive ventilation (for any reason and for at least 48 h). US diaphragmatic assessment was performed in two stages: at SP and at 60 min of positioning in PP. The US measurements were obtained at the zone of apposition of the right hemithorax. The Teleinspiratory and telexpiratory diameters (TID/TED) were taken on the 3 medio-axillary lines: posterior, median and anterior and their means were considered. Thus, the Diaphragmatic thickening fraction (DTF) was calculated as: DTF = (TID − TED /TED) × 100. All these values were compared by pairing.

**Results:** The diaphragmatic contractile function was explored in 40 patients with the following basic characteristics: sex-ratio = 2.07, median age (years) = 42 [30–60], median BMI = 23.6 [22–28], SAPS II = 42 [29–51], SOFA = 5 [3–8]. The most common co-morbidities were chronic respiratory failure (CRF, n = 11) and hypertension (n = 11). Respiratory distress (n = 19) and coma (n = 18) were the major indications for IV. US diaphragmatic exploration was performed at a median delay of IV at 4 days [2–7]. 95% of patients received sedation and 27.5% received neuromuscular blockers. The ventilator mode was control volume in 35 patients via endotracheal tube (n = 33) and tracheostomy cannula (n = 7). No major incident was detected during the turning of patients. Both TID and TED decreased from the SP to the PP (Fig. 1): TID (mm) (28 in SP vs. 24.5 in PP, p = 0.001), TED (mm) (18.7 in SP vs. 18 in PP, p = 0.037). The observed DTF was lower in the PP but without significance (37.4 vs. 42.05%, p = 0.36). No difference was showed when the comparison between SP-DTF and PP-DTF was adjusted on the ventilator mode, obesity, neuromuscular blockers and CRF.

**Conclusion:** The positioning in PP in ventilated patients reduces both tele-inspiratory and tele-expiratory diameters of the diaphragm but not altered its contractile function.

**Compliance with ethics regulations:** Yes.

**Fig. 1:**
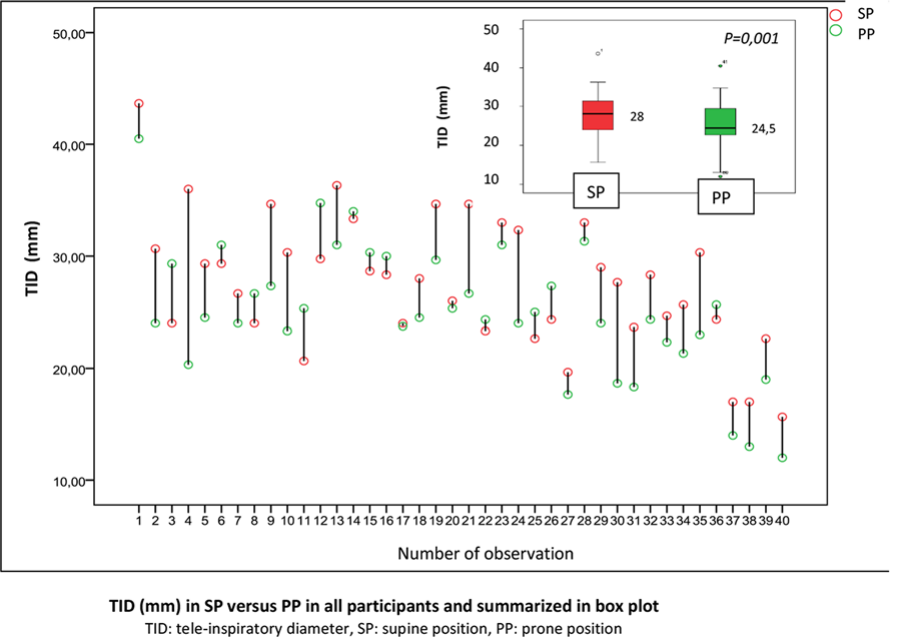
TID (mm) in SP versus PP in all participants and summarized in box plot

### P-101 Assessment of aerosol delivery during mechanical ventilation of a simulated adult tracheostomy patient

#### Barry Murphy, Andrew O’sullivan, Mary Joyce, Gavin Bennett, Elena Fernandez Fernandez, Amy Tatham, Ronan Macloughlin

##### Aerogen Ltd., Galway, Ireland

###### **Correspondence:** Barry Murphy (Bmurphy@aerogen.com)

*Ann. Intensive Care* 2020, **10(Suppl 1):**P-101

**Rationale:** Tracheostomy is a method used for the artificial airway management of patients with respiratory illnesses. The efficiency of aerosol therapy during mechanical ventilation is influenced by the choice of nebuliser and the location of the nebuliser within the circuit. This study assesses the aerosol delivery efficiency of a vibrating mesh nebuliser (VMN) and a jet nebuliser (JN) at two circuit positions during mechanical ventilation in a simulated adult tracheostomy patient.

**Patients and methods:** The aerosolised drug dose was assessed by nebulising a 3 mL dose of 1 mg/mL Salbutamol using a VMN (Aerogen Solo, Aerogen, Ireland) or a JN (Cirrus 2, Intersurgical, UK) during mechanical ventilation (Servo-U, Maquet, Sweden) of a simulated adult (Vt 500 mL, 15 BPM, I:E 1:1) tracheostomy patient. The nebuliser was placed on the inspiratory limb or at the dry side of the humidifier within a dual limb circuit (RT200, Fisher & Paykel, New Zealand). A filter (Respigard, Baxter, Ireland) was placed proximal to the tracheostomy tube (TT) (adult ID 6.4 mm, Shiley, Medtronic, Ireland). The mass of drug was determined using UV spectrophotometry at 276 nm. Results, indicating the dose delivered to the patient, are expressed as the percentage of the nominal dose placed in the nebuliser’s medication cup. Significance was considered at p < 0.05.

**Results:** Results are presented in the table below.

**Discussion:** Nebuliser type influences the efficiency of aerosol delivery, with the VMN delivering a significantly higher % aerosol dose than the JN at the two circuit positions (p = 0.0037 on inspiratory limb; p = 0.0004 at the dry side of humidifier). In agreement with previous reports using bias flow, for both nebulisers, the location within the circuit has a significant effect, with the nebuliser on the dry side of the humidifier delivering more aerosol than on the inspiratory limb (p = 0.0100 for VMN; p = 0.0127 for JN).

**Conclusion:** For a mechanically ventilated adult tracheotomy patient, the type of nebuliser and the location of the nebuliser within the circuit influences aerosol delivery.

**Conflicts of interest:** All authors are employees of Aerogen. 1Ari, A, Respir Care 2015 2Ari A, Respir Care 2010.

**Compliance with ethics regulations:** Yes.

**Table 1:** Results

	% Aerosol dose Average ± SD		
	VMN	JN	P-value
On inspiratory limb	24.52 ± 1.03	12.95 ± 0.21	0.0037
On dry side of humidifier	32.62 ± 1.10	20.97 ± 1.51	0.0004
P-value	0.0100	0.0127	

### P-102 An international survey on practice in use of automatic tube compensation mode during mechanical ventilation in Intensive Care units

#### Emanuele Turbil^1^, Louis-Marie Galerneau^2^, Nicolas Terzi^2^, Claude Guérin^3^

##### ^1^University of Sassaria, Sassaria, Italy; ^2^Medecine Intensive Reanimation, Grenoble, France; ^3^Medecine Intensive Reanimation, Lyon, France

###### **Correspondence:** Emanuele Turbil (emanuele.turbil@gmail.com)

*Ann. Intensive Care* 2020, **10(Suppl 1):**P-102

**Rationale:** Automatic tube compensation (ATC) is a mode available in most ICU ventilators. It compensates for the resistive pressure into endotracheal tube/tracheostomy canula by continuously providing a pressure assistance based on internal diameter of a new endotracheal tube/tracheostomy tube. Its use in ICU is unclear. We designed a survey to further explore this.

**Patients and methods:** The survey was endorsed by the acute respiratory failure section and the clinicaltrials group of the European Society of Intensive Care Medicine (ESICM). The pool was sent out via an email on June 27 2019 to the ESICM members worldwide. The following closed questions were: country, years in ICU, kind of ICU, kind of hospitals, kind of respirators, ATC use (never, always or in some patients), reasons to or not to use ATC, ventilatory mode in which ATC was used. The database was frozen on August 1^st^ 2019 after two reminders. We used the Gross National Income per capita (USD) provided by the world bank 2016 to transform the respondent’s country into a geographical-economical variable with 3 levels: High-Europe, high-nonEurope and middle (1). ATC use was coded as yes or no. The primary end-point was ATC rate of use and the hypothesis was that less than 50% of the respondents do use it. Variables were expressed as counts. Groups were compared by Chi square test. A logistic regression analysis was performed to explore the contributing factors to ATC use.

**Results:** We received 644 responses without any doublons, of which six were empty, from 72 countries. Four-hundred and nine respondents used ATC always or in some patients (64% ATC rate of use). This rate was not different between Economical-geographical regions, ICU, hospitals and years in ICU. For those respondents who did not use ATC the reasons were: ATC mode not available in ICU ventilators (41.9%), ATC not helpful mode (36.7%), ATC not known (18.8%) and ATC provides too much pressure assistance (5.7%). For those respondents who used ATC the reasons were: helpful in weaning (68.2%), set by default (30.5%) and physiological benefit (1.2%). They used ATC during spontaneous breathing trial (30.4%), with any assisted mode (27.9%) and with specific modes (11.7%). We found no risk factor for ATC use in the logistic regression model (Fig. 1).

**Conclusion:** The ATC rate of use was unexpectedly high in this survey. This may result from respondents selection bias or from an a priori underestimation of its use.

**Compliance with ethics regulations:** Yes.

**Fig. 1:**
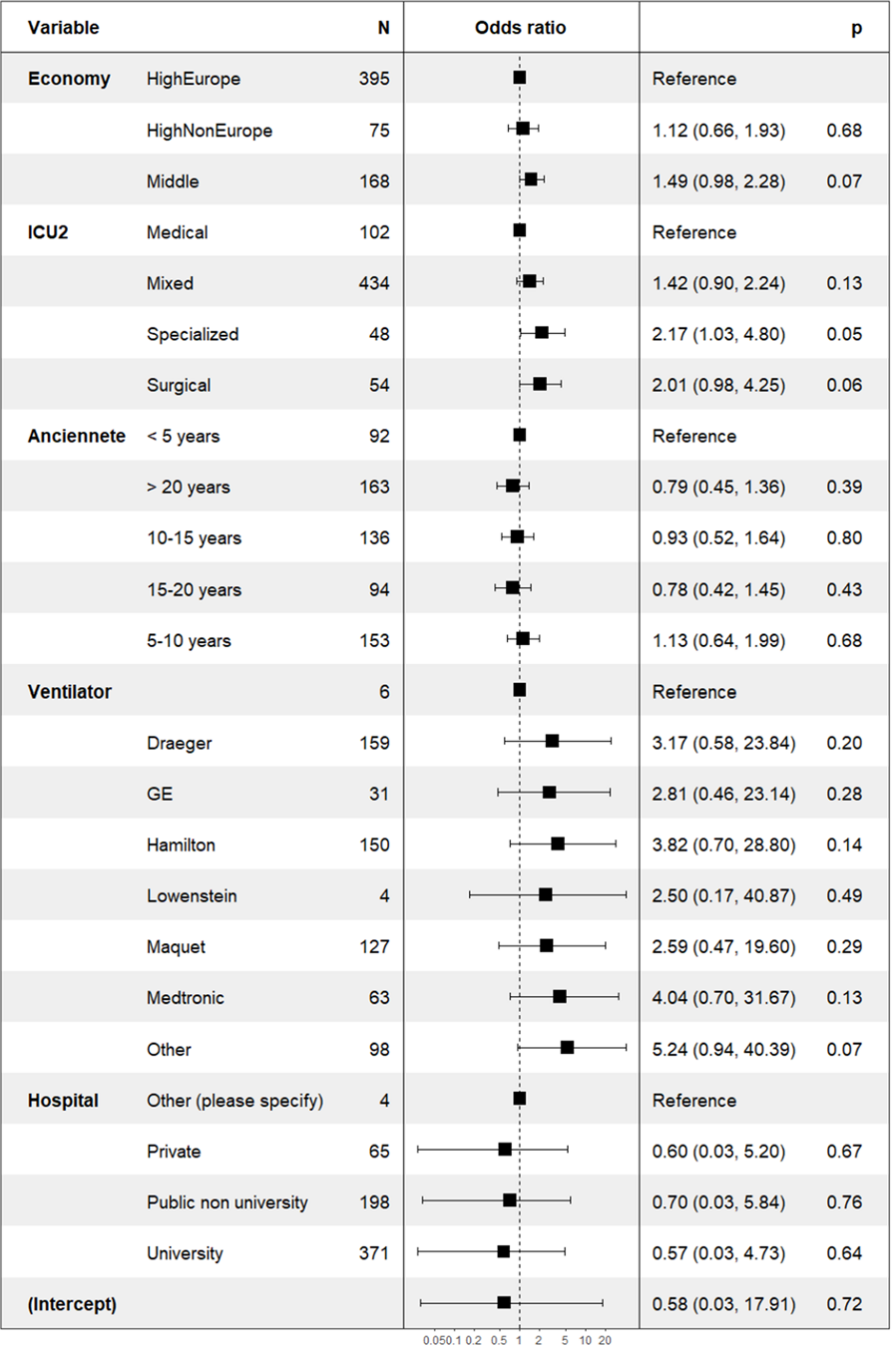
.

**Reference**

1. Laffey et al. Lancet Respiratory Medicine 2017.

### P-103 Assessment of respiratory effort based on the analysis of the ventilator waveforms: a machine learning approach

#### Samuel Tuffet^1^, Bruno Louis^2^, Yvon Deplante^3^, François Perier^1^, Anne-Fleur Haudebourg^1^, Nicolas De Prost^1^, Keyvan Razazi^1^, Armand Mekontso Dessap^1^, Guillaume Carteaux^1^

##### ^1^Réanimation Médicale CHU Henri Mondor, Créteil, France; ^2^INSERM U955 Equipe 13, Créteil, France; ^3^GRC CARMAS UPEC, Créteil, France

###### **Correspondence:** Samuel Tuffet (samuel.tuffet@hotmail.fr)

*Ann. Intensive Care* 2020, **10(Suppl 1):**P-103

**Rationale:** During pressure support ventilation (PSV), adjusting the level of assistance mainly aims at maintaining the patient’s respiratory effort within a normal range. However, respiratory effort measurement is impeded in clinical routine by the need of esophageal pressure recording. In this study, we evaluated the accuracy of assessing the respiratory effort from the flow and airway pressure signals using several machine learning algorithms based on the equation of motion of the respiratory system.

**Patients and methods:** Using the ASL5000 Simulator (IngMar Medical) connected to a PB980 ventilator (Medtronic) set in PSV, we simulated a massive number of different respiratory cycles. Each simulated cycle represented a unique combination of compliance and resistance of the respiratory system, duration and intensity of the muscle pressure (Pmus), positive end-expiratory pressure (PEEP) and pressure support levels. Using least squares regression methods, the flow waveform was fitted according to the equation of motion of the respiratory system to determine the compliance and resistance of the respiratory system, and the Pmus. The hypothesis used (alone or in combination) to constrain the system were: linearity of Pmus at the onset of the inspiratory effort, nullity of Pmus at the end of insufflation, and nullity of Pmus during expiration. Thus, nine methods were built and tested. Calculated and actual peak Pmus values were compared using the Bland-Altman method.

**Results:** The nine methods of Pmus assessment were evaluated using 34 550 different simulated cycles. By limiting the analysis to selected cycles with a predefined applicability criterion (intrinsic PEEP less than 1 cmH_2_O), a limited inspiratory effort (peak Pmus less than 15 cmH_2_O) and a high quality of fitting (R^2^ > 0.99), the method using the three hypothesis together to constrain the system was characterized by a bias of 0.34 cmH_2_O and limits of agreement of -0.44 and 1.12 cmH_2_O. However, when widening the analysis to all the simulated conditions, no method allowed an accurate estimation of the peak Pmus : the best one exhibited a bias of -3.38 cmH_2_O and limits of agreement of − 15.18 and 8.42 cmH_2_O.

**Conclusion:** Among the nine machine learning methods tested, some provided an accurate estimate of the respiratory effort in selected cycles but none allowed such accuracy across all simulated conditions. This incites to assess automated methods using a more complex physiological and physical model.

**Compliance with ethics regulations:** Not applicable.

### P-104 Oesophageal pressure measurement in intubated patients by intensive care units nurses: the ON educational study

#### Maëva Campfort, Flavie Laurioux, Pierre-Yves Olivier, Lise Piquilloud, Alain Mercat, Francois Beloncle

##### MIR, CHU d’Angers, Angers, France

###### **Correspondence:** Maëva Campfort (maevacampfort@aol.com)

*Ann. Intensive Care* 2020, **10(Suppl 1):**P-104

**Rationale:** There is a growing interest in esophageal pressure monitoring in mechanically ventilated patients. Esophageal pressure can be measured with a specific nasogastric catheter equipped with esophageal balloon and connected to a pressure transducer. It is used as a surrogate for pleural pressure and may be considered as a corner stone in advanced care of ventilated patients to better assess lung and chest wall mechanics and easily detect patient-ventilator asynchronies. However, this promising technique is still seldom used in clinical practice. Trained ICU nurses may perform oesophageal pressure measurements which may help facilitate its implementation in the usual patient care. This study aimed at assessing whether a specific educational program to train nurses to perform esophageal pressure monitoring allowed reliable measurements.

**Patients and methods:** This was a prospective monocenter study performed in an academic ICU. Written informed consent was obtained from the nurses before inclusion in the study. The specific educational program consisted of a 30-min online theoretical course, a 1-h group theoretical teaching and a 30-min simulation training on a mannequin. Then each participating nurse performed three esophageal pressure measurements (using Nutrivent^®^ catheters and an ICU monitor connected to arterial line pressure transducers system) on three different mechanically ventilated paralysed patients under supervision. A knowledge assessment was performed with a short written MCQ test. The skill evaluation was by two trained experts. Concretely the trained nurses performed an esophageal pressure measurement without assistance. Their ability to control the esophageal balloon position by an occlusion test, to measure the inspiratory and expiratory airway and transpulmonary pressures and to calculate of respiratory system, lung and chest wall compliances was assessed at the bedside using a standardized evaluation form.

**Results:** We present here the preliminary results of the first nine included nurses. The written knowledge assessment was considered as good (median MCQ test score 34/50, range 32–40/50). The nine trained nurses (100%) succeeded their practical evaluation at first attempt.

**Conclusion:** These preliminary results suggest that trained ICU nurses are able to adequately perform esophageal pressure monitoring. The implementation of this measurements by ICU nurses could allow to facilitate the implantation of this technique in the ICUs

**Compliance with ethics regulations:** Yes.

### P-105 Weaning from mechanical ventilation in burns: Which weaning test in clinical practice?

#### Kawther Faleh, Hana Fredj, Houda Maayoufi, Amel Mokline, Imen Jami, Manel Ben Saad, Amenallah Messaadi

##### Intensive Burn Care Departement, Burn and Trauma Center, Ben Arous, Tunisia

###### **Correspondence:** Kawther Faleh (kaouther_1@yahoo.fr)

*Ann. Intensive Care* 2020, **10(Suppl 1):**P-105

**Rationale:** Several modalities of ventilatory support have been proposed to gradually withdraw patients from mechanical ventilation. We conducted this study to compare T-piece and Pressure support ventilation (PSV) (8 cmH_2_0 and Peep0) in the process of weaning of mechanical ventilation in burns.

**Patients and methods:** It was a prospective randomized trial in burn ICU in Tunisia during 9 months. Mechanically ventilated patients who met standard weaning criteria were included [1]. Patients were randomized into two groups: group1 under T-piece and group 2 under PSV. Duration of the test: 30–120 min. The tolerance of the VS test should be judged on clinical criteria. Stopping the test if occurred: agitation, tachypnea > 35 cycles/ min, tachycardia > 140 / min, SpO_2_ < 90%. Successful withdrawal was defined as the ability to maintain spontaneous respiration for 48 h after extubation.

**Results:** Thirty patients were included, randomized into two groups. The mean age was 29 ± 11 years with a ratio sex of 4. The average TBSA was 31 ± 18%. The cause of mechanical ventilation was essentially a face neck burned (79%). The following table shows the weaning outcome of both modalities. Eighty percent of succeeded extubation for both groups (N = 12/15). The cause of failure of extubation was secretion retention and clutter in majority of cases followed by neurological and cardiac distress. The duration of mechanical ventilation does not influence the outcome of the weaning test (p < 0.005), with a mean of duration of 16 ± 10 days.

**Conclusion:** Our study did not show any difference between the two weaning modalities in the matter of outcome of extubation. The choice of weaning test of mechanical ventilation is to be judged by the clinician according of the state of his patient.

**Compliance with ethics regulations:** Not applicable.

**Table 1:**
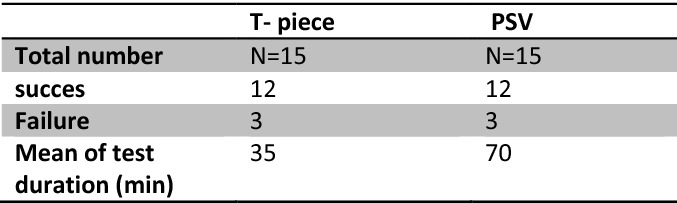
Comparative table between the two groups

### P-106 Role of chest wall expiratory flow limitation and airway closure in patients with acute respiratory distress syndrome

#### Nicolas Terzi^1^, Louis-Marie Galerneau^1^, Mehdi Mezidi^2^, Emanuele Turbil^3^, Hodane Yonis^2^, Loredana Baboi^2^, Bruno Louis^4^, Carole Schwebel^1^, Claude Guérin^2^

##### ^1^Médecine Intensive Réanimation, CHU Grenoble Alpes, Grenoble, France; ^2^Médecine Intensive Réanimation, CHU de Lyon, Lyon, France; ^3^University of Sassaria, Sassaria, Italy; ^4^INSERM 955, Créteil, France

###### **Correspondence:** Nicolas Terzi (nterzi@chu-grenoble.fr)

*Ann. Intensive Care* 2020, **10(Suppl 1):**P-106

**Rationale:** When expiratory tidal flow does not go up after increasing expiratory driving pressure expiratory flow limitation (EFL) occurs. It is thought that EFL heralds airway closure (AC). We investigated the role of chest wall elastance (Ecw) in both EFL and AC in acute respiratory distress syndrome (ARDS) patients. Our hypothesis was that the lower the Ecw to lung elastance (EL) ratio the higher the likelihood of EFL and AC.

**Patients and methods:** Twenty-five moderate to severe ARDS patients were prospectively included in two centers. Mechanical ventilation was delivered in volume-controlled mode with tidal volume 6 ml/kg predicted body weight at positive end-expiratory pressure 5 cmH_2_O in semi-recumbent position. Airway (Paw) and esophageal (Pes) pressures and flow were continuously recorded during 2 min by a data logger (Biopac 150). Then, end-expiratory and end-inspiratory occlusions were performed for 5 s, then respiratory system was slowly inflated at constant flow. Finally, patient was allowed to breathe out freely to atmosphere by using a three-way stop lock by-passing expiratory valve. AC and airway opening pressure (AOP) were determined according to Chen et al. (1). EFL was assessed by the atmospheric method (2). Dynamic elastance of chest wall (Edyn,cw) and lung (Edyn,L) were obtained from least square linear regression method over 10 consecutive breaths. Static elastance (Est,cw and Est,L) were determined by classic formulas and also by taking into account AOP (Est,cw_aop and Est,L_aop, respectively). The performance of Ecw/EL ratio to predict EFL and AC was assessed by the area under receiver operating characteristic (AUCROC) curve.

**Results:** EFL was observed in 9 patients (36%) and AC in 14 (56%). Median AOP was 8.2 cmH2O (95% CI 7.6–12.8). AUCROCs for Ecw/EL ratios to detect EFL and AC are shown in Table 1. Edyn,cw/Edyn,L ratio was better to detect EFL than Est,cw/Est,L ratio with Edyn,cw/Edyn,L ≤ 0.16 100% sensitivity and 84% specificity. Correction for AOP made the performance of Est,cw/Est,L ratio as good as that of the Edyn ratio. AC was poorly predicted by Edyn and Est ratios but its prediction greatly improved with AOP correction. However, with the Est,cw/Est,L_aop the critical ratio was 1.81 (sensitivity 75%, specificity 100%) and 0.60 (sensitivity and specificity 100%) for predicting EFL and AC, respectively.

**Conclusion:** EFL and AC are frequent in ARDS at PEEP 5 cmH_2_O. Edyn,cw/Edyn,L ratio lower than 1 best predicted EFL occurrence. Once AC is taken into account Est,cw/Est,L ratio greater than 1 accurately predicts AC. EFL and AC are two distinct phenomena.

**Compliance with ethics regulations:** Yes.

**Table 1:** Area under ROC curve (95% confidence intervals) for chest wall to lung elastance ratios to predict expiratory flow limitation (EFL) and airway closure (AC) as classification variables

	EFL	P value (versus 0.50)	AC	P value (versus 0.50)
Edyn,cw/Edyn,L	0.90 (0.85–0.94)	< 0.001	0.61 (0.54–0.68)	0.013
Est,cw/Est,L	0.75 (0.54–0.90)	0.011	0.55 (0.34–0.75)	0.75
Est,cw/Est,L_aop	0.90 (0.62–0.99)	< 0.001	1.00 (0.86–1.00)	< 0.001

### P-107 Anesthesia outside operating room in pediatrics

#### Ihssan Mehrez, Adnane Berdai, Said Belmekadem, Mustapha Harandou

##### CHU HASSAN II, Fes, Morocco

###### **Correspondence:** Ihssan Mehrez (mehrez.ihssan@gmail.com)

*Ann. Intensive Care* 2020, **10(Suppl 1):**P-107

**Rationale:** Anesthesia outside the operating room (AOOR) in a pediatric environment was giving increasingly increasing indications and a lot of progress because of its interest in carrying out diagnostic and/or therapeutic explorations: 20% of the acts of anesthesia are performed outside the operating room. The objective of our study is: to clarify the importance and the frequency of the practice of the AHBO, to define its particularities, as well as an evaluation of the ratio: benefit/risk in order to reduce the morbidity and mortality.

**Patients and methods:** We report in this study the experience of the service of the resuscitation mother–child on the gestures of AOOR. This is a prospective observational study, spread over a period of 2 months: from 1/09/17 to 31/10/17, dealing with 251 acts performed for endoscopic digestive and bronchial procedures, cures in dermatology and radiotherapy, and medical imaging (CT and MRI).

**Results:** Of the 251 procedures performed: 91 were performed for CT, 66 for MRI, 7 for arteriography and 50 for endoscopic digestive procedures, 14 for bronchoscopies, 6 for radiotherapy treatments, 17 for laser treatments in Dermatology. Anesthesia techniques use intravenous induction in 70% of cases using: hypnotics (propofol, midazolam, ketamine), morphine (remifentanyl, fentanyl), inhalation induction in 30% of cases (sevoflurane, halothane) and curare for 7 cases of bronchoscopy (rocuronium). This anesthesia was marked by the occurrence of accidents in order of frequency: cardiac in 15% of cases (tachycardia, hypotension and rhythm disorders), and then respiratory in 10% of cases. The most serious accidents were admitted in reality and are represented by 8 cases, of which 3 required an intubation (bronchoscopy), a case of cardiorespiratory arrest recovered, 2 cases of severe hypoxia associated with bradycardia and which required the ventilation with the mask (radiotherapy), and 2 cases of bronchospasm requiring the deepening of the anesthesia (absence of TCI).

**Conclusion:** A good knowledge of the patient and the intervention, and difficulties specific to each specialty is necessary, as well as a pre-anesthetic consultation. The AOOR must obey the same safety rules as in the operating theater and that in terms of: equipment, monitoring (Integrate the capnograph to respiratory monitoring whenever deep sedation and when the continuous control of VAS is difficult), anesthetic technique (TCBI) and post-procedure wakefulness management that must meet the same requirements as the SSPI, especially for prolonged sedation.

**Compliance with ethics regulations:** Yes.

### P-108 Umbilical vein catheterization through Wharton’s jelly: a possibility for a fast and safe way to deliver treatments in the delivery room?

#### Suzanne Borrhomée

##### Hôpital René Dubos, France

###### **Correspondence:** Suzanne Borrhomée (suzanne.borrhomee@gmail.com)

*Ann. Intensive Care* 2020, **10(Suppl 1):**P-108

**Rationale:** A fast and safe venous access can be a critical issue in the delivery room during neonatal cardiopulmonary resuscitation, or before endotracheal intubation. Here, we describe a new method to inject drugs using the umbilical vein, directly punctured through Wharton’s jelly.

**Patients and methods:** This method was performed in 10 newborns between November 2016 and May 2018. Umbilical vein was identified and punctured easily and a reflux was obtained in all patients. The first step was antisepsis, and then the umbilical vein was punctured. The puncture was made approximately 2 to 3 cm above the navel. After checking for blood reflux, the nurse injected the treatment. The cannula was left in the vein during the injection and removed as soon as the intervention was over (intubation was performed, or the heart rate had increased).

**Results:** Here, we report ten cases of emergency injection in the delivery room using this method: —four cases of cardiopulmonary resuscitation using this method to deliver epinephrine. Cardiac massage was performed on all patients.—six cases of intubations in the delivery room using this method to administer the premedication. In all patients, the umbilical vein was identified easily. The equipment was the one usually used for venous injection in our unit and was manipulated and handled with ease. Venous access was obtained in a matter of seconds, and blood reflux was observed in all patients. The treatments were efficient in all but two patients, which was imputable to the method in one patient.

**Discussion:** Although this method has been known in our NICU for several years, there has been no publication regarding this method in neonates. Inserting an umbilical vein catheter in the delivery room has been validated for resuscitation but this technique is lengthy and requires some sterility conditions that makes it even longer, and thus non-fitting for an emergency tracheal intubation. Our method is fast and can be performed easily with no specific training. The whole manipulation procedure, from the beginning of the puncture to the end of the flush- out takes 15 to 20 s. We only identified few specific risks related to this method, mostly infectious, and the risk of drug diffusion.

**Conclusion:** We describe a new route for administration of drugs in the delivery room that was successfully used in 9 nine neonates. Umbilical vein needle catheterization is not only safe and efficient, but is also fast and easy to perform without any special training.

**Compliance with ethics regulations:** Not applicable.

### P-109 Cerebral Vascular Stroke (CVA) in children

#### Choubeila Guetteche

##### Universitary hospital center, Constantine, ALGERIA

###### **Correspondence:** Choubeila Guetteche (cguetteche@gmail.com)

*Ann. Intensive Care* 2020, **10(Suppl 1):**P-109

**Rationale:** Stroke is a rare event in children with slurred morbimortality and risk of serious sequelae, the objective of our study was to determine the para-clinical and progressive clinical characteristics of children admitted for stroke.

**Patients and methods:** Retrospective study including children aged less than 15 years admitted to pediatric intensive care between 2014 and 2018 for ischemic or haemorrhagic stroke, with collection of epidemiologic clinical paraclinical and evolutionary data.

**Results:** Twenty four children, the average time of diagnosis and management 48 h the revealing signs are not specific seizures 75% of cases the alteration of the state of consciousness more headache, the diagnosis is confirmed by cerebral computed tomography, stroke is associated with viral or bacterial meningo-encephalitis, but a large number remain without etiology.

**Conclusion:** A peculiarity of stroke in pediatrics occurring in a being in full development thunderstorm and intellectual, despite the diagnostic progress especially radiological care time is still long

**Compliance with ethics regulations:** Not applicable.

### P-110 High glucose intake and glycemic level in postoperative liver transplantation for children with inherited metabolic disorders of protein intoxication

#### Mehdi Oualha, Victoire Thenot, Marion Grimaud, Anais Brassier, Pascale De Lonlay, Fabrice Lesage, Florence Lacaille, Christophe Chardot, Laurent Dupic, Sylvain Renolleau

##### Necker Hospital, Paris, France

###### **Correspondence:** Mehdi Ouahla (mehdi.oualha@aphp.fr)

*Ann. Intensive Care* 2020, **10(Suppl 1):**P-110

**Rationale:** High glucose intake is recommended in postoperative liver transplantation for children with inherited metabolic disorders of protein intoxication. The significant glucose intake coupled with stress increases the risk of prolonged hyperglycemia. We aimed to assess glucose intake and associated glycemic tolerance the first 72 h following liver or liver/kidney transplantation for inherited metabolic disorders of protein intoxication.

**Patients and methods:** We performed a single case-control study, between January 2011 and December 2018 in the pediatric intensive care unit (PICU) of the Necker-Enfants Malades Hospital of Paris, which was constituted of 26 patients, divided into three different groups : 10 patients with organic acidemias, 6 patients with urea cycle disorders and 10 patients in the control group. Severe hyperglycemia was defined as > 12 mmol/L.

**Results:** During the first 3 days, the median glucose intake rate was 4.4 mg/kg/min (2.5–8.3), 6.5 mg/kg/min (4.9–11.3) and 1.8 mg/kg/min (0.9–3.3) respectively for organic acidemias, urea cycle disorders and control groups, respectively (p = 0.011). Ten (100%) patients with organic acidemias, 5 (83%) patients with urea cycle disorders and 2 (20%) control patients developed severe hyperglycaemia. The average hours spent in severe hyperglycemia was 18.1 h (± 15.5), 5.3 h (± 7.1) and 1.7 h (1.5), respectively for organic acidemias, urea cycle disorders and control groups (p = 0.0009). Insulin was required for 7 patients, organic acidemia, n = 5 and urea cycle disorders, n = 2 (p = 0.04). The “glycemic level/glucose intake” ratio was 4 (0.6–36.6); 1.7 (0.7–5.1) and 2.6 (0.1–12.5) respectively for organic acidemias, urea cycle disorders and control groups (p = 0.0089).

**Conclusion:** Hyperglycemia is common in critically ill transplanted children with inherited metabolic disorders of protein intoxication who receive high glucose intake. The tolerance of glucose intake varied between the two groups of patients with inherited metabolic disorders. Decreasing the glucose intake, especially in patients of organic acidemias, should be suggested to ensure more effective glycemic control.

**Compliance with ethics regulations:** Yes.

### P-111 Acute cellular rejection in first month after pediatric liver transplantation: rate and risk factors

#### Pauline Adnot, Laure Cohen, Florence Moulin, Mehdi Oualha, Muriel Girard, Stephanie Chhun, Christophe Chardot, Sylvain Renolleau, Marion Grimaud

##### Hôpital Necker Enfants Malades, Paris, France

###### **Correspondence:** Pauline Adnot (pauline.adn@gmail.com)

*Ann. Intensive Care* 2020, **10(Suppl 1):**P-111

**Rationale:** Liver transplantation (LT) is the only option for children with end stage liver disease. Recent advances in surgical procedure and immunosuppression have permitted a better patient and long term graft survival. However, acute cellular rejection remains a frequent complication occuring in 20 to 50% of the cases according to different studies. It is more likely to occur during the 4 first weeks post LT. Many predictive factors of acute rejection have been described in litterature and results differ from one study to another. Pediatric studies regarding this topic are few. The aim of this work is to study acute cellular rejection prevalence in the 30 days following LT and to determine predictive factors.

**Patients and methods:** This monocentric retrospective study included patients under 18 years who received a liver transplantation at Necker-Enfants-Malades University Hospital between January 1^st^ 2013 and December 31^st^ 2018. Data regarding receiver, donor, immunosuppression and pre-operative data have been recorded during 30 days post LT. The main event was the occurrence of acute cellular rejection histologically assessed. Survival without reject at 30 days was studied by Kaplan Meier method. Univariate and multivariate Cox model was used to determine predictive factors of acute rejection.

**Results:** 102 patients were included. Median receiver age was 2.69 years [0.94–7.43]. Main LT indication was biliary atresia (57.8%). Median donor age was 22 years [17–35] and donor was mainly deceased (91.3% of cases). Probability of survival at 30 days without rejection was 29.2%. Acute rejection occured during the 30 days for 53 patients (51.9%) with a median time of occurrence of 11 days [9.0–15.5] and was mainly treated by corticosteroids boluses (86.7%) with efficiency. The only statistically significant risk factor of acute rejection was biliary complication (HR = 2.28 [1.01–5.16], p = 0.049). Donor age > 50 years was also found to slightly increase risk of acute rejection (HR = 2.30 [0.88–5.96], p = 0.088). No data regarding immunosuppression was found statistically significant.

**Conclusion:** The prevalence of acute cellular rejection during the first month post LT was high in our population. Occurrence of biliary complication and higher donor age were two risk factors of acute rejection. These datas suggest a closer monitoring of the patients at risk in order to take preventive mesures to maintain patient and long term graft survival.

**Compliance with ethics regulations:** Yes.

### P-112 Dexmedetomidine sedation for pediatric MRI: a retrospective cohort from a French pediatric intensive care unit

#### Hélène Lepeltier^1^, Maxime Gauberti^2^, Arnaud Lepetit^3^, Charlotte Roulland^1^, Alexandre Frugier^3^, David Brossier^4^, Isabelle Goyer^5^

##### ^1^CHU CAEN, Département de pédiatrie, Caen, France; ^2^CHU CAEN, Département de radiologie, Caen, France; ^3^CHU CAEN, Département d’anesthésie, Caen, France; ^4^CHU CAEN, Département de réanimation pédiatrique, Caen, France; ^5^CHU CAEN, Département de pharmacie, Caen, France

###### **Correspondence:** Hélène Lepeltier (helene.lepeltier@orange.fr)

*Ann. Intensive Care* 2020, **10(Suppl 1):**P-112

**Rationale:** Sedation practices for pediatric magnetic resonance imaging (MRI) are highly heterogenous. The main challenge is to keep children immobile while being alone in a traumatizing environment for a long time. Clinicians have to ensure hemodynamic and respiratory stability in this isolated environment while minimizing sedation neurologic adverse effects. In this series, we report the potential usefulness, feasibility, efficacy and safety of dexmedetomidine sedation for pediatric MRI.

**Patients and methods:** A single center retrospective review of six children sedated with dexmedetomidine for MRI in an emergency context. All children were hospitalized in the pediatric intensive care unit of a University Hospital at the time of MRI.

**Results:** Data on six patients aged 8 months to 4 years is reported. Five patients received dexmedetomidine by intravenous route (bolus of 1–2 µg/kg over 10 min, followed by a continuous infusion of 1 µg/kg/h). One child received dexmedetomidine by intranasal route (4 µg/kg with atomization device). One child experienced bradycardia that did not require any intervention. Very few movements were recorded during the MRIs for which images were rated as good quality.

**Conclusion:** Dexmedetomidine seems a promisingly useful sedation agent for pediatric MRI, thanks to its efficient sedative properties and good tolerability without respiratory compromise.

**Compliance with ethics regulations:** Yes.

**Table 1:**
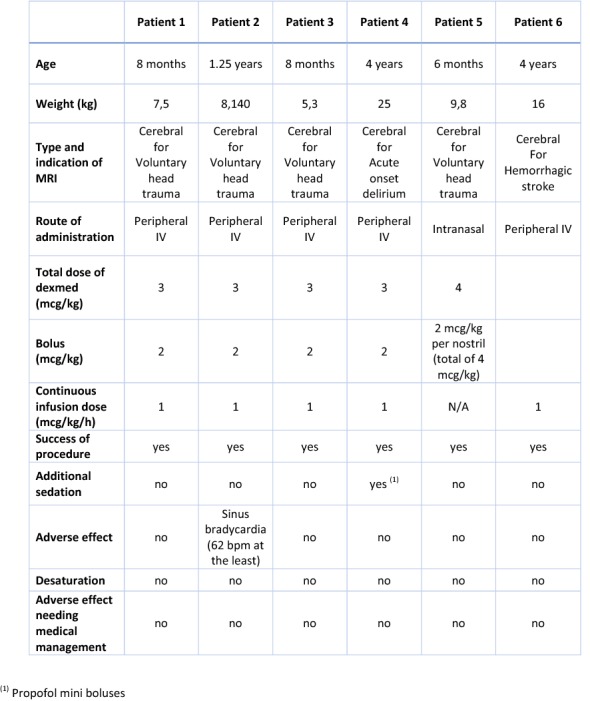
Patient cases description and dexmedetomidine use data

### P-113 Evaluation of simulresp©: a simulation software of child and teenager cardiorespiratory physiology

#### David Brossier^1^, Olivier Flechelles^2^, Michael Sauthier^1^, Guillaume Emeriaud^1^, Bernard Guillois^3^, Philippe Jouvet^1^

##### CHU Sainte Justine, Montréal, Canada; ^2^CHU de Martinique, Fort De France, France; ^3^CHU de Caen, Caen, France

###### **Correspondence:** David Brossier (david_brossier@yahoo.fr)

*Ann. Intensive Care* 2020, **10(Suppl 1):**P-113

**Rationale:** Computational models, or virtual patients, could be used to teach cardiorespiratory physiology and ventilation, determine optimal ventilation management as well as forecast the effect of various ventilatory support strategies. Currently, there is no virtual patient specifically designed for modelling children cardiorespiratory system. Thus, our research team has developed a cardiorespiratory simulator for children called “SimulResp©”. According to Summers et al., the quality of a physiologic model is evaluated by three specific criteria: qualitatively, which relates to the model’s ability to provide directionally appropriate predictions; quantitatively in steady states and in dynamics, which is the ability of the model to provide accurate predictions in steady state situations as well as dynamic transitions. The purpose of this study was to evaluate the quality of SimulResp© according to these criteria.

**Patients and methods:** This study consisted in a prospective evaluation of the SimulResp©’s predictions with simulated healthy subjects. The tests were performed with patients from 8 to 18 years old (8, 10, 12, 14, 16, 18 years), with different characteristics; gender (M, F) and weight (10th, 50th and 90th percentile). Blood gas values (pH, PCO_2_, PO_2_ and SpO_2_) were simulated for several virtual healthy patients with different characteristics. This study was conducted for both spontaneously breathing and mechanically ventilated patients. SimulResp©’s quality and reliability were evaluated in terms of accuracy, robustness, repeatability and reproducibility.

**Results:** Simulresp©’s validation procedures are ongoing. We intend Simulresp© to be accurate when simulating healthy spontaneously breathing patients. But we hypothezised that Simulresp© would not be able to simulate accurate blood gas values of mechanically ventilated patients

**Conclusion:** Simulresp© is a promising computational model that will serve to perform calibration and validation procedures of clinical decision support systems and help clinican to determine optimal respiratory support strategies at bedside. Further calibration procedures are yet required.

**Compliance with ethics regulations:** Yes.

### P-114 Place of tracheotomy in pediatric resuscitation

#### Samira Kalouch, Khalid Yakini, Wissal Aissaoui, Abdelaziz Chlilek

##### CHU IBN ROCHD Casablanca, Casablanca, Morocco

###### **Correspondence:** Samira Kalouch (dr.kalouch@gmail.com)

*Ann. Intensive Care* 2020, **10(Suppl 1):**P-114

**Rationale:** The tracheostomy procedure dates back to ancient times. Its use has been adapted in the neonatal and pediatric population during the last half-century. It consists of creating a small hole in the trachea with placement of a cannula. Tracheostomy also referred to as a stoma, is the hole in the neck that the tube passes through. The aim of this report is to study the indications, the techniques and the complications of this intervention, as well as to specify the prognostic factors of the tracheotomy.

**Patients and methods:** This study consists of 40 tracheotomized patients managed in the intensive care unit. The study was carried out for a period of 4 years, from January 2014 to December 2017.

**Results:** The work involved 25 boys and 15 girls whose ages ranged from 6 months to 14 years with an average age of 7 years and a standard deviation of 4 years. Head trauma (22.5%) and Guillain-Barré syndrome (17%) were the main reasons for admission to the intensive care. Up to 97.5% of tracheotomies were performed for prolonged tracheal intubation. While tracheotomies performed for emergency intubation cases failed by 2.5% of the time. The technique used was the isthmic surgical tracheostomy, which was performed in the operating room by otolaryngologist under general anesthesia. The cutaneous incision was transversal in all cases.The choice of the cannula was adapted to the age, and the decanulation was carried out according to the evolution of the underlying disease. Complications associated with tracheotomy are diverse, and common complications are such as care-associated pneumonia (22.5%), tracheostomy tube obstruction (7.5%), accidental decannulation (2.5%), pneumothorax (2.5%) and cases of tracheal stenosis (2.5%). The mortality rate amounted to 27.5%, where in most cases was due to the poor prognosis of the underlying diseases. The main factors of evolution are the patient’s previous condition, cranial trauma, Guillain-Barré syndrome, tracheostomy time, prolonged tracheal intubation and the presence of complications.

**Conclusion:** Regardless of the indication, the tracheotomy is an act of survival whose usefulness and effectiveness are certain. The mastery of technique, proper selection of equipment, full knowledge of the anatomic relationships of the trachea, careful monitoring and postoperative care are the main conditions to minimize the risk of occurrence of complications.

**Compliance with ethics regulations:** Not applicable.

### P-115 Aspiration pneumonia and drug overdose requiring invasive ventilation: impact of a care protocol on the antibiotic prescription

#### Gurvan Le Bouar^1^, Pierre-Louis Declercq^2^, Medhi Bousta^3^, Jean-Baptiste Michot^4^, Jean-Philippe Rigaud^2^, Laurie Lagache^3^, Olivier Delastre^4^, Déborah Boyer^5^, Samia Boubeche^5^, Dorothée Carpentier^5^, Thomas Clavier^5^, Elsa Demarest^1^, Christophe Girault^5^, Jean Glenisson^5^, Elise Godeau^5^, Steven Grange^5^, Damiano Cerasuolo^6^, Fabienne Tamion^5^, Gaetan Beduneau^5^

##### ^1^Medical-Surgical Intensive Care Unit, District Hospital Center, Évreux, France; ^2^Medical-Surgical Intensive Care Unit, District Hospital Center, Dieppe, France; ^3^Medical-Surgical Intensive Care Unit, District Hospital Center, Le Havre, France; ^4^Medical-Surgical Intensive Care Unit, District Hospital Center, Elbeuf, France; ^5^Medical Intensive Care Unit, Rouen University Hospital, Rouen, France; ^6^Unit of biostatistics, Rouen University Hospital, Rouen, France

###### **Correspondence:** Gurvan Le Bouar (gurvan.lbk@hotmail.fr)

*Ann. Intensive Care* 2020, **10(Suppl 1):**P-115

**Rationale:** Aspiration pneumonia (AP) is a frequently suspected complication of drug overdose requiring mechanical ventilation (MV) and admission to intensive care unit (ICU). In the absence of reliable biomarkers for distinguishing between aspiration pneumonia and aspiration pneumonitis, antibiotic therapy is frequently prescribed. Latest studies suggest that a care protocol could better select patients requiring antibiotic therapy.

**Patients and methods:** The objective was to determine the impact of a care protocol on the antibiotic prescription among patient admitted to ICU for toxic coma with MV. We conducted a prospective observational cohort study in four ICU. We included all patients admitted for toxic coma with MV. In the University-affiliated ICU, a care protocol was applied. In the three others ICU, physicians declared that they did not follow formalized conduct within the service and did as usual.

**Results:** We included 43 patients in care protocol group and 42 in control group. The mean SAPS II was 43.3 (± 15.3) with a mean Glasgow Coma Scale score at 4.9 (± 2.1) before intubation. Within the total population, 40 patients (47%) had a pulmonary bacteriologic sample (PBS), mostly because purulent tracheobronchial aspirate and new infiltrates on the chest X-ray (respectively 36.4% and 29.4% of the population with a bacteriological sample). Among the patients with a bacteriological sample, 34 (85%) were culture positive. The incidence of probabilistic antibiotherapy did not differ between the care protocol group (n = 16) and the control group (n = 16) . There was no difference for the incidence of PBS (20 in each group). The others secondary outcomes did not differ either (Table 1).

**Conclusion:** Our study does not show that a care protocol allows a reduction of antibiotic prescription among patient admitted to ICU for toxic coma with MV. Our incidence of antibiotic prescription is lower than the previous studies. The absence of difference can be explain by two reasons: some of the physicians of the control group had been trained in the university-affiliated ICU in the last years and may follow a management approach similar to that of the control group; despite our precautions, the existence of the study could have modify the practices in the control group.

**Compliance with ethics regulations:** Yes.

**Table 1:**
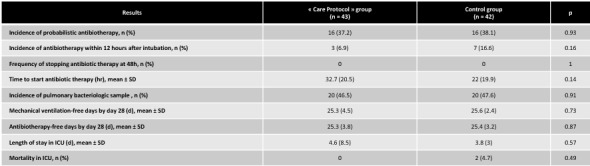
Outcome in patients included

### P-116 Impact of hyperoxemia on mortality during septic shock: retrospective study on the MIMIC-III database

#### Benjamin Popoff, Bertrand Dureuil, Benoit Veber, Thomas Clavier

##### Département d’Anesthésie Réanimation, CHU de Rouen, Université de Rouen Normandie, Rouen, France

###### **Correspondence:** Benjamin Popoff (benjamin.popoff@chu-rouen.fr)

*Ann. Intensive Care* 2020, **10(Suppl 1):**P-116

**Rationale:** Hyperoxemia is associated with excess mortality in intensive care unit (ICU) patients, particularly after stroke, myocardial infarction or cardiac arrest. The effect on septic shock is unknown and few studies have been conducted on the subject. The objective of our study was to evaluate the impact of hyperoxemia on mortality during septic shock.

**Patients and methods:** Design of a retrospective study using the Medical Information Mart for Intensive Care III (MIMIC-III) database provided by the Massachusetts Institute of Technology (MIT) with the approval of the MIT Ethics Committee and the Beth Israel Deaconess Medical Center in Boston. The inclusion criteria were: age above 18 years-old, SEPSIS 3 septic shock criteria and ICU stay with invasive ventilation at least during 24 h after admission. O_2_ exposure was defined by the time weighted average PaO_2_ over the first 24 h of resuscitation with distinction between two groups: normoxemia (PaO_2_ 70–120 mmHg) and hyperoxemia (PaO_2_ > 120 mmHg). The primary endpoint was ICU mortality. The variables are reported as mean (standard deviation) or median [interquartile space]. Univariate analysis was performed by Student or Wilcoxon tests for continuous variables and Pearson Chi^2^ tests for categorical variables. A multivariate analysis was performed by logistic regression adjusting for age, gender, type of ICU (medical or surgical), SOFA score, liver disease and hyperoxemia. Crude and adjusted odds ratios with their 95% confidence intervals could thus be calculated. The analyses were carried out bilaterally, taking a significance threshold of p < 0.05.

**Results:** Between 2001 and 2012, 613 patients were included, 252 in the normoxemia group and 361 in the hyperoxemia group. The demographics data and main results are presented in Table 1. The mean PaO_2_ in the normoxemia group was 98.23 ± 12.62 mmHg compared to 166.1 ± 43.81 mmHg in the hyperoxemia group. No difference of ICU mortality between the two groups were shown in univariate (41.3% vs. 33.2%, OR 0.71 [0.50–1.01], p = 0.052) and multivariate analysis (OR 0.93 [0.65–1.32], p = 0.67).

**Conclusion:** In this population of patients with septic shock, hyperoxemia was not an independent factor of mortality. These data are in contradiction with those previously published in other ICU patient populations. Thus, a prospective study in septic patients with a direct analysis of the impact of hyperoxemia appears necessary.

**Compliance with ethics regulations:** Not applicable.

**Table 1:**
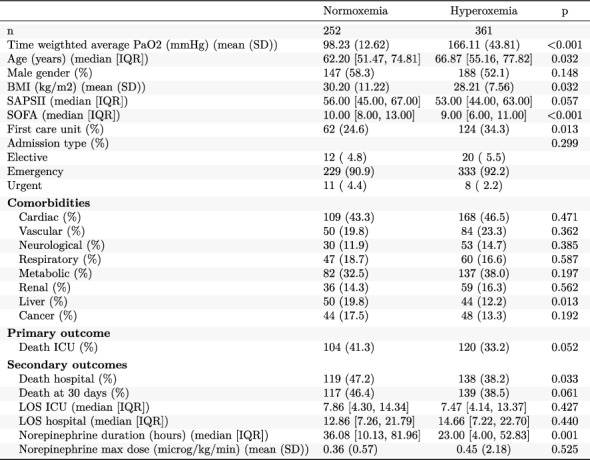
Demographic characteristics of the population and main results

*BMI* body mass index, *SOFA* sepsis-related organ failure assessment score, *SAPSII* Simplified Acute Physiology Score II, *LOS* length of stay, ICU intensive care unit

### P-117 Impact of multiplex PCR (Filmarray TM) on the diagnosis and adequacy of initial therapy in HCAP/HAP/VAP hospitalized in ICU: a pilot monocenter study

#### Jonathan Pehlivan^1^, Camille D’humieres^1^, Juliette Patrier^1^, Lila Bouadma^1^, Manon Lejeune^2^, Clement Lebihan^1^, Laurence Armand-Lefrevre^2^, Jean-François Timsit^3^

##### ^1^Bichat RMI, Paris, France; ^2^Bichat microbiologie, Paris, France; ^3^APHP Bichat Paris, Paris, France

###### **Correspondence:** Jonathan Pehlivan (jonathan.pehlivan@gmail.com)

*Ann. Intensive Care* 2020, **10(Suppl 1):**P-117

**Rationale:** Little is known about the accuracy and impact of the extended multiplex PCR respiratory panel Filmarray biofire (mPCR) on the diagnosis and immediate adequacy of antimicrobial therapy in HCAP/HAP/VAP hospitalized in ICU. Indeed the impact depend on careful analysis of risk factors, previous antimicrobial therapy, Gram stain result, and previous knowledge about the spectrum of the multiplex PCR and its interpretation.

**Patients and methods:** Using results of multiplex PCR not given to the intensivists, we aimed to evaluate the accuracy of mPCR, and the impact of mPCR on the adequacy of empirical antimicrobial therapy. Methods: a microbiological/intensivist expert panel with a previous experience in interpreting mPCR were asked to propose empirical therapy with the case report file including gram stain examination with or without mPCR results. The narrowest spectrum were based on the definitions from Weiss et al [1].

**Results:** 21 episodes (2 HCAP,4 HAP, 15 VAP), with 35 microorganisms, occurring 27 days and 7.9 days in mean after hospital and ICU admission, were included. The SAPSII was 49 in mean, 8 patients were immunocompromized. Quantitative correlation between mPCR and cultures from 11 bronchalveolar lavage (BAL), 7 plugged telescoping catheters (PTC) and 3 endotracheal aspirates (ETA) are on Fig. 1. *S. maltophilia*, *Enterococcus sp.* and *Morganella morganii* were not in the mPCR panel. CTX-M detection was positive for the 4 cases of Extended spectrum beta-lactamases pneumonia. The expert panel proposed an empirical therapy inadequate for 3 cases with or without mPCR. The therapy was narrower when using mPCR (12 vs 9 cases) (Table 1).

**Conclusion:** Even in experienced hand the interpretation of mPCR is complex. The absence of some important gram negative organisms induced inadequate empirical therapy. mPCR allow a minor decrease in the broad spectrum empirical therapy.

**Compliance with ethics regulations:** Yes.

**Table 1:**
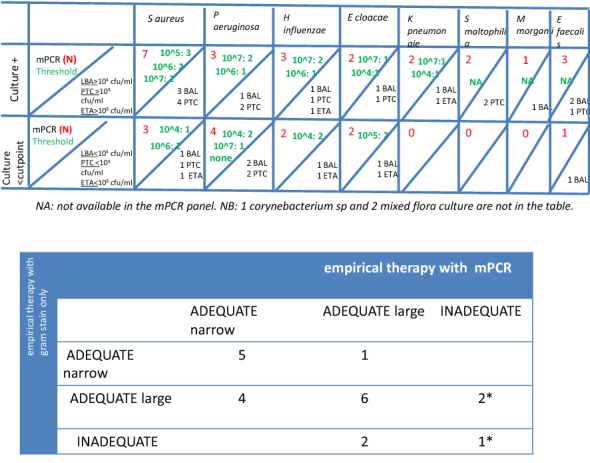
.

### P-118 How to improve the post-operative antibiotics prescription after major pancreatic surgery? A retrospective cohort study

#### Matthieu Petit, Guillaume Geri, Elsa Salomon, Frédérique Peschaud, Antoine Vieillard-Baron, Xavier Repessé

##### APHP-hôpital Ambroise Paré, Boulogne-Billancourt, France

###### **Correspondence:** Matthieu Petit (matthieu-petit@hotmail.fr)

*Ann. Intensive Care* 2020, **10(Suppl 1):**P-118

**Rationale:** Pancreatic surgery is associated with high morbidity, mostly due to infectious complications, so that many centers introduce post-operative antibiotics for all patients. Such systematic prescriptions are not consensual and often rely on local practices. The aims of the study were to describe the occurrence of surgical site infection (SSI) and the antibiotic (ATB) prescription after pancreatic surgery, and to determine the risk factors of post-operative surgical site infection, in order to better define the clinical indications for the prescription of antibiotics after major pancreatic surgery.

**Patients and methods:** All patients undergoing a scheduled major pancreatic surgery from January 2007 to November 2018 were included in the study. Patients were classified in four groups according to the occurrence of a surgical site infection and to the post-operative antibiotic prescription as follows (SSI+/ATB+; SSI-/ATB+; SSI+/ATB-, SSI-/ATB-). In addition, risk factors (fever and pre-operative biliary prosthesis) associated with the occurrence of a surgical site infection and with the antibiotic prescription, were analyzed using a logistic regression model.

**Results:** Data from 149 patients (115 pancreaticoduodenectomies and 34 splenopancreatectomies) were analyzed and classified as presented in the table. Thirty patients (20.1%) experienced a surgical site infection and 42 (28.2%) received post-operative antibiotics. We did not find any difference on post-operative antibiotic prescriptions (26.7% versus 28.6%, p = 0.9) between patients who developed a surgical site infection and those who did not. Amongst the 107 patients who were not prescribed antibiotics post-operatively, 85 (79.4%) did not develop a surgical site infection while 22 (20.6%) did. In-ICU mortality did not differ between infected and non-infected patients (7 versus 2%, p = 0.13). Post-operative fever was different between SSI+ and SSI- (73.3 versus 34.2%, p < 0.001), while the prevalence of pre-operative biliary prosthesis was similar (37.9 versus 26.7%, p = 0.3). Amongst patients who did not develop a surgical site infection, antibiotic prescription was not associated with fever (p = 1), but associated with a higher prevalence of pre-operative biliary prosthesis (15.6 versus 52.9%, p = 0.0001).

**Conclusion:** Non-systematic antibiotic prescription after major pancreatic surgery allowed to appropriately spare antibiotics in 85 (56%) patients at the cost of under prescription in 22 (14.8%) patients. These results suggest that systematic post-operative antibiotic prescription could be excessive. Fever appears to be a relevant clinical sign for individual-based prescription, whereas the presence of a biliary prosthesis does not.

**Compliance with ethics regulations:** Yes.

**Table 1:**
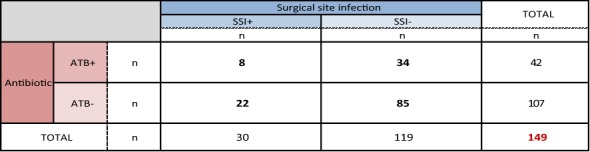
Contingency of the 149 patients according to the occurrence or not of a surgical site infection and to the presence or not of systematic post-operative antibiotics

Table of contingency of the 149 patients according to the occurrence or not of a surgical site infection (SSI+/SSI-) and to the presence or not of systematic post-operative antibiotics (ATB+/ATB-)

### P-119 Care paths of pneumococcal meningitis with sepsis in France: a retrospective analysis of a French medico-administrative (PMSI) database

#### Michael Thy^1^, Claire Dupuis^1^, Bruno Mourvillier^2^, Lila Bouadma^1^, Stephane Ruckly^3^, Anne Perozziello^3^, Damien Van-Gysel^4^, Etienne De Montmollin^1^, Romain Sonneville^1^, Jean-François Timsit^1^

##### ^1^Réanimation médicale et infectieuse, Paris, France; ^2^Réanimation médicale, CHU Reims, Reims, France; ^3^UMR 1137, IAME, équipe Descide, Université de Paris, Paris, France; ^4^Département d’informations médicales, CHU Bichat, APHP, Paris, France

###### **Correspondence:** Michael Thy (michael245thy@gmail.com)

*Ann. Intensive Care* 2020, **10(Suppl 1):**P-119

**Rationale:** Pneumococcal meningitis (PM) in adult patients is associated with substantial morbidity and mortality (1). Current guidelines recommend initial management of PM patients in intermediate or intensive care units (ICU) (2, 3). However, there is little evidence to support those recommendations (1). We aimed to describe care paths of PM with sepsis in French hospitals and to assess outcomes depending on their hospital trajectory.

**Patients and methods:** We conducted a retrospective analysis of the French medico administrative (PMSI) database of consecutive patients with PM and sepsis admitted to French hospitals, between 2010 and 2015. Only the first hospital admission was considered. Cases were identified using a combination of a diagnosis code for PM plus a diagnosis code for organ failure or a procedure code for organ support. Hospital trajectories were determined from the first admission to death or discharge, taking into account all potential transfers. Costs and endpoints were determined at the end of patients’ trajectories. Five groups of patients were defined, according to care pathways: Direct ICU admission (1stICU); Secondary ICU admission, after initial admission to another unit including wards (ward2ndICU) or intermediate care (Interm2ndICU); Admission to an intermediate care unit only (IntermOnly); No ICU admission (noICU). The association between trajectory group and hospital mortality was investigated using logistic regression analysis, after adjustment for confounders.

**Results:** From 2010 to 2015, 1236 patients were hospitalized in France for a first episode of PM with sepsis. NoICU was a small (n = 24, 1.9%) group of elederly patients [80 years (58; 85)], with a high mortality rate (50%) that may correspond to premortem misdiagnosis or early limitation of care. Main characteristics of the remaining four groups are reported in Table 1. IntermOnly were less severe with less organ failures, had a shorter LOS, and a lower death rate and cost of care. After adjustment on age, chronic illness, severity of organ failures and septic shock, compared to 1stICU, only ward2ndICU was associated with an increased risk of death (OR = 1.83[1.38; 2.44]).

**Conclusion:** In France, from 2010 to 2015, most of PM with sepsis were admitted in ICU or intermediate care unit. A delayed ICU or intermediate care unit admission seemed to be harmful.

**Compliance with ethics regulations:** Yes.

**Table 1:** Comparison of the care paths of Pneumococcal Meningitis with sepsis in France

	1stICU	Interm2ndICU	ward2ndICU	IntermOnly	Pval (all)	Pval 1stICU versus ward2ndICU
Cases	636	153	362	61	.	
Characteristics of the patient						
Age > 65 y.o.	244 (38.36)	76 (49.67)	171 (47.24)	26 (42.62)	0.01	< 0.01
Hospital stay characteristics						
Academic institution	292 (45.91)	81 (52.94)	189 (52.21)	22 (36.07)	0.03	0.06
Severity of the patients						
Kidney organ failure	259 (40.72)	62 (40.52)	139 (38.4)	16 (26.23)	0.16	0,47
Respiratory organ failure	614 (96.54)	152 (99.35)	352 (97.24)	22 (36.07)	< 0.01	0.55
Septic shock	620 (97.48)	149 (97.39)	350 (96.69)	36 (59.02)	< 0.01	0.46
Coma	400 (62.89)	89 (58.17)	217 (59.94)	13 (21.31)	< 0.01	0.36
Purpura	49 (7.7)	11 (7.19)	19 (5.25)	5 (8.2)	0.50	0.14
Codes for identification of pneumococcal infection						
Pneumococcal pneumonia (J13)	151 (23.74)	34 (22.22)	81 (22.38)	9 (14.75)	0.45	0.62
Pneumococcal septicemia(A403)	173 (27.2)	51 (33.33)	111 (30.66)	22 (36.07)	0.24	0.24
Outcomes						
Hospital length of stay, median (IQR)	21 [9; 39]	26 [15; 43]	23 [10; 46]	16 [11; 28]	< 0.01	0.22
ICU length of stay, median (IQR)	11 [5; 21]	12 [6; 21]	10 [4; 22]	1 [0; 4]	< 0.01	0.29
Discharge to home	184 (28.93)	34 (22.22)	86 (23.76)	32 (52.46)	< 0.01	0.08
Hospital mortality	253 (39.78)	67 (43.79)	186 (51.38)	8 (13.11)	< 0.01	< 0.01
Cost(€), median (IQR)	21,783 [13,197; 33,774]	27,653 [17,262; 41,659]	22,584 [14,013; 37,245]	12,067 [8398; 18,355]	< 0.01	0,19

### P-120 Protective effects of landiolol against sepsis-induced atrial fibrillation in mice: preliminary results

#### Romaric Larcher^1^, Jerome Thireau^2^, Jeremy Fauconnier^2^, Sarah Colombani^2^, Alain Lacampagne^2^, Kada Klouche^1^

##### ^1^Médecine intensive-Réanimation, CHU de Montpellier INSERM, CNRS, PhyMedEx, Université de Montpellier, Montpellier, France; ^2^INSERM, CNRS, PhyMedEx, Université de Montpellier, Montpellier, France

###### **Correspondence:** Romaric Larcher (r-larcher@chu-montpellier.fr)

*Ann. Intensive Care* 2020, **10(Suppl 1):**P-120

**Rationale:** New-onset atrial fibrillation (AF) is a common complication in patients with sepsis and is associated with increased mortality and morbidity rates. This condition results from a complex chain of events in response to infection, involving immunologic, humoral and cellular process and sympathetic overactivity. Landiolol, the new injectable beta-blocker, with high beta1 selectivity and minimal impact on arterial blood pressure, may have beneficial effects in such a context. In this study, we aimed to investigate whether landiolol decrease the new-onset of atrial fibrillation in a mice model of endotoxin-induced sepsis.

**Patients and methods:** Thirty C57BL/6 male mice were randomly allocated to the following groups: sham (administration of 500 µL of isotonic saline intraperitoneally—IP), septic (administration of 500 µL of isotonic saline with 20 mg/kg of lipopolysaccharide—LPS—of *E. coli* O55:B5 IP) and septic + landiolol (administration of isotonic saline with 20 mg/kg of LPS and, two hours later 25 mg/kg of landiolol IP). Four hours later, an attempt of AF occurrence was triggered by a transesophageal electric pacing at fixed rate (as previously reported) in all mice previously anesthetized by isoflurane 2%. EKG was continuously recorded.

**Results:** Ten mice per group (mean weight: 22 ± 2 g) have been included and analyzed. Among the sham group the mean heart rate was at 362 bpm versus 502 bpm in the septic group. Among the septic + landiolol group the mean heart rate was at 465 bpm (p < 0,001). After transesophageal stimulation, none mice in the Sham group had AF, seven mice (70%) in the septic group had an AF, and 2 mice (20%) in the septic + landiolol group had an AF. Landiolol decreased the incidence of new-onset, sepsis-induced atrial fibrillation in mice (p = 0.025).

**Conclusion:** Landiolol seems to have a protective effect against sepsis-induced AF in mice. However, the mechanisms, including sympathetic activation and inflammasome pathways, should be investigated before drawn definitive conclusion regarding to efficiency of landiolol to prevent New-onset AF during sepsis.

**Compliance with ethics regulations:** Yes.

**Table 1:** EKG recording before and after transesophageal electric pacing

	Sham (n = 10)	Septic (n = 10)	Septic + landiolol (n = 10)
EKG recording before transesophageal stimulation			
Heart rate (bpm), mean ± SD	362 ± 27	502 ± 30	465 ± 21
Sinus cycle length (ms), mean ± SD	167 ± 12	118 ± 9	130 ± 7
P wave (ms), mean ± SD	11 ± 2	17 ± 2	8 ± 2
PR interval (ms), mean ± SD	40 ± 5	43 ± 6	42 ± 5
QRS duration (ms), mean ± SD	10 ± 1	9 ± 1	9 ± 1
QT interval (ms), mean ± SD	15 ± 2	15 ± 2	15 ± 3
QTc (ms), mean ± SD	37 ± 4	43 ± 8	40 ± 13
EKG recording after transesophageal stimulation			
Atrial fibrillation, n (%)	0 (0%)	7 (70%)	2 (20%)

### P-121 Quantifying carriage of extended-spectrum beta-lactamase-producing enterobacteriaceae and subsequent ICU acquired infection in French Guiana

#### Hatem Kallel^1^, Claire Mayence^1^, Caroline Dupuy^1^, Cyrille Mathien^1^, Stephanie Houcke^1^, Dabor Resiere^2^, Hommel Didier^1^

##### ^1^CH Cayenne, Cayenne, France; ^2^CHU Martinique, Fort De France, France

###### **Correspondence:** Hatem Kallel (hatem.kallel@ch-cayenne.fr)

*Ann. Intensive Care* 2020, **10(Suppl 1):**P-121

**Rationale:** Carriage of ESBL-Producing Enterobacteriaceae (ESBL-PE) in ICU is responsible of increased mortality and morbidity. In this study, we were interested in quantifying ICU carriage and ICU-AI caused by ESBL-PE and whether carriage of ESBL-PE had an impact on ICU-AI.

**Patients and methods:** Our study is a prospective observational non-interventional work. It was conducted over 5 years period (Jan 2013–Dec 2017) in the medical-surgical intensive care unit of the Cayenne General Hospital.

**Results:** During the study period 1698 patients were admitted in our ICU. The median age of our patients was 44 years [28–60]. One or more comorbidity was recorded in 45.6% of patients. Immunodeficiency was recorded in 30.8% of patients. One hundred fifty-nine patients (9.3%) were ESBL-PE carriers at admission to ICU and 208 patients acquired ESBL-PE carriage during ICU stay (13.5% of non ESBL carriers at admission). Among the 367 ESBL-PE carriers, 137 patients (37.3%) developed ICU-AI. By multivariable analysis, independent factors associated to the occurrence of ICU-AI were Trauma admission (p: 0.029; OR: 2.5 [1.1–5.7]), Mechanical Ventilation > 48 h (p: 0.006; OR:17.5 [2.2–136.7]), Tracheostomy (p: 0.006; OR: 4.6 [1.5 -13.9]), Renal Replacement Therapy (p: 0.030, OR: 2.5 [1.1–5.9]), Arterial catheterization (p: 0.033, OR: 4.1 [1.1–14.8]), Exposure to : AMX-Clav (p: 0.014; OR: 2.8 [1.2–6.3]), to PIP-TAZ (p: 0.007; OR: 2.5 [1.3–4.8]), to 3rd GC (p: 0.000; OR: 3.6 [1.8–7.3]), and to Imipenem (p: 0.000; OR: 6.1 [2.8–13.2]) during hospitalization. ICU-AI in ESBL-PE carriers was caused by an ESBL-PE in 39 cases (28.4%). Factors associated with ICU-AI caused by ESBL-PE in ESBL-PE carriers were trauma admission (p: 0.001; OR: 0.1 [0–0.5]), Hemodynamic failure at admission (p: 0.005; OR: 4.2 [1.5–11.4]), and Imipenem exposure prior to infection (p: 0.025; OR: 3.2 [1.2–8.9]). The positive predictive value of ESBL-PE carriage to predict ESBL-PE as the causative germ of ICUAI was 28.5%, and the negative predictive value was 84.3%.

**Conclusion:** ESBL-PE carriage is frequently associated to ICU-AI, but, does not predict ESBL-PE as responsible germ of ICU-AI. Antibiotic exposure mainly to carbapenems is independent risk factor exposing to ICU-AI and to ICU-AI caused by ESBL-PE.

**Compliance with ethics regulations:** Yes.

### P-122 Vancomycin serum concentration after 48 h of administration: a 3 years survey in an intensive care unit

#### Nicolas Perin^1^, Claire Roger^1^, Laurent Muller^1^, Jean-Yves Lefrant^1^, Jason A. Roberts^2^

##### ^1^CHU Caremeau Nîmes, Nîmes, France; ^2^CHU Caremeau Nîmes, Nîmes, France

###### **Correspondence:** Nicolas Perin (perinnico.1@gmail.com)

*Ann. Intensive Care* 2020, **10(Suppl 1):**P-122

**Rationale:** Vancomycin dosing protocol in intensive care still remain heterogenous and unreliable. The present study assessed the rate of patients with adequate concentration (20–25 mg/L) after 24–48 h vancomycin continuous intravenous administration, and determined factors associated with a fail of this objective.

**Patients and methods:** In this prospective observational study performed from 2016 to 2018, we analysed the patterns of vancomycin administration in patients admitted in 31 beds Intensive Care Unit (ICU) without previous renal replacement therapy. Main objective assessed the rate of patient with first available vancomycin serum concentration between 20–25 mg/L at 24 or 48 h. Secondary objectives were factors associated with a vancomycin serum concentration out of 20–25 mg/L at 24 or 48 h, proportion of patients with a vancomycin serum concentration < 10 mg/L, previously associated with resistance emergence and assessment of mortality and test of cure.

**Results:** A serum vancomycin concentration between 20–25 mg/L was reported in 43 out of 179 included patients (24%). A serum vancomycin concentration < 20 ml/L and > 25 mg/L were reported in 89 patients (51%) and 44 patients (25%), respectively. Vancomycin serum concentrations during follow-up are shown in Fig. 1. In multivariate regression analysis, a longer time between admission and initiation of vancomycin was the only parameter associated with a serum vancomycin out of this target, while acute kidney injury (AKI) was associated with a lower incidence of subtherapeutic concentration. Acute kidney injury rate was significantly higher in patients with a serum vancomycin concentration > 25 mg/L.

**Discussion:** An adequate therapeutic target of serum vancomycin concentration was reached in 25% patients with nearly 50% < 20 mg/L, which was similar to previous studies. AKI and RRT requirement were higher in patients with serum vancomycin concentration > 25 mg/L, whereas it is hardly to know whether it is a cause or a consequence.

**Conclusion:** These findings highlight the importance of a larger loading dose, vancomycin monitoring and measured creatinin clearance to improve vancomycin dosing protocol.

**Compliance with ethics regulations:** Yes.

**Fig. 1:**
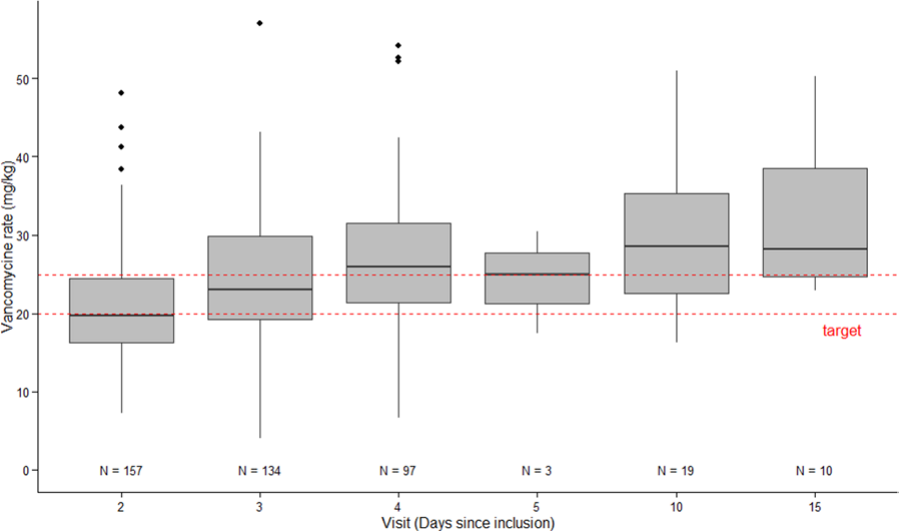
Vancomycin serum concentration throughout the follow-up (24 h of administration was defined as D2; D3 to D15 were the following days)

### P-123 Psychiatric profile of patients hospitalized in intensive care unit for suicide attempt

#### Jihene Guissouma^1^, Meriem Ksouri^1^, Hatem Ghadhoune^1^, Habib Brahmi^1^, Yesmine Garbaa^1^, Hana Benali^1^, Rawnak Houli^1^

##### Hôpital Habib Bougatfa, Bizerte, Tunisia

###### **Correspondence:** Jihene Guissouma (bahri.jihene@yahoo.fr)

*Ann. Intensive Care* 2020, **10(Suppl 1):**P-123

**Rationale:** Suicide is a global phenomenon and one of the leading causes of death in the world. Tunisia ranks second in the suicide rate in the Maghreb, with 5.5 cases of suicide per 100,000 inhabitants. The aim of this study was to reconstruct the state of suicidal subjects before the act in order to identify their psychiatric profile.

**Patients and methods:** A 3-year prospective observational single-center (6-bed intensive care unit) study including all patients hospitalized for suicide attempt (SA). Psychiatric evaluation of patients and contact with their families were done before intensive care unit discharge.

**Results:** Seventy-one patients were enrolled with female predominance (sex ratio 0.42). Mean age was 30 ± 13 years. Familial or personal history of mental illness were found in 8 (11%) and 30 cases (42%) respectively. Personal mental disorders were depression (17%), bipolar disorder (10%), schizophrenia (8%) and border line personality disorder (7%). Twenty-five per cent had prior SA. Sixty-three per cent were single, 30% married and 7% divorced. The Common methods of suicide included drug (52%), chloralose (34%) and pesticide (13%) poisoning. Mean IGS II and APACHE II scores were 30 ± 19 and 14 ± 9 respectively. On admission, 59% of all patients were in coma, 11% had shock and 48% developed aspiration pneumonia. Mechanically ventilation was done in 59% of all cases with mean duration of 3 days. The mean length of stay in intensive care unit was 4 days. Mortality rate was 7%. Psychiatric evaluation and contact with families deduced that the main precipitating factors for suicide were traumatic events. In fact: relationship problems (familial, marital or breakups), school failure and mourning were found in 66%, 6% and 1% of all cases respectively. Reactional SA accounted for 61%. Besides; SA was a sign of a psychiatric pathology collapse in 28% of all cases. Otherwise; it inaugurated a mental illness which was not yet diagnosed in 11% of all cases. Twenty four patients (34%) required psychiatric inpatient admission; whereas 42 (59%) have had psychiatric follow-up on external consultation.

**Conclusion:** In this study, the main risk factors for suicide were traumatic events. Analyzing the psychiatric profile of our population revealed essentially reactional SA, psychiatric pathology collapse or an entry form to a mental illness which was not yet diagnosed. This emphasizes the importance of a regular follow up of suicide attempters in order to control known mental disease or to treat a new psychiatric condition so we can prevent recurrent SA.

**Compliance with ethics regulations:** Yes.

### P-124 Impact of hyperglycemia in severe scorpion envenomation

#### Oussama Jaoued, Yosri Ben Ali, Gharbi Rim, Sik Ali Habiba, Fekih Hassen Mohamed, Elatrous Souheil

##### Hôpital Taher Sfar, Mahdia, Tunisia

###### **Correspondence:** Oussama Jaoued (oussamajaoued@gmail.com)

*Ann. Intensive Care* 2020, **10(Suppl 1):**P-124

**Rationale:** Severe forms of scorpion envenomation (SE) require intensive care unit (ICU) hospitalization. Hyperglycemia is often observed in this situation due to a massive release of catecholamine, increased glucagon secretion and decrease in insulin levels. The aim of this study is to determine the impact of hyperglycemia in severe SE.

**Patients and methods:** This is a retrospective study including all patients admitted to our medical ICU for severe SE between 2011 and 2019. Cardiogenic shock, pulmonary edema and stroke were the most frequent clinical manifestations. Diabetics patients and patients who did not have a blood glucose measurement at admission were excluded. Hyperglycemia was defined by a blood glucose level ≥ 1.8 g/dl.

**Results:** Sixty-three patients aged 40 ± 21 years were included. The incidence of hyperglycemia at ICU admission was 35%. Only five patients required invasive mechanical ventilation. Thirty-seven patients presented with a cardiogenic shock. ICU length of stay was 14 days [IQR 10–23]. Only one patient died. The demographic characteristics were similar between patients with and without hyperglycemia. Cardiogenic shock was observed in 55% of hyperglycemia group and 61% in euglycemic group (p = 0.62). Mechanical ventilation, nosocomial infection, and duration of mechanical ventilation were similar between both groups. The length of stay was 22 ± 6 days in hyperglycemic patients and 24 ± 4 days in euglycemic patients. Work load assessed by omega score was similar in both groups.

**Conclusion:** Hyperglycemia is frequent in severe scorpion envenomation, but seems to be without impact on morbidity.

**Compliance with ethics regulations:** Yes.

### P-125 Fatal poisoning in south Tunisia: a 12 years study

#### Wiem Ben Amor, Narjess Karray, Malek Zribi, Karim Derbel, Zouhir Hammami, Mabrouk Bahloul, Mounir Bouaziz, Samir Maatoug

##### ^1^Forensic departement Habib Bourguiba University Hospital, Sfax, Tunisia; ^2^Reanimation departement Habib Bourguiba University Hospital, Sfax, Tunisia

###### **Correspondence:** Wiem Ben Amor (wiembenamar@yahoo.fr)

*Ann. Intensive Care* 2020, **10(Suppl 1):**P-125

**Rationale:** Poisoning is a worldwide problem, associated with high morbidity and moratlity. In Tunisia, the rate of fatal poisoning has been increasing in the last years, with emergence of new toxic substances. Regardless of the toxic, fatal poisining is considered as a non natural death, that requires medico-legal investigation, to assess whether it is suicidial, crimnal or accidental death. This study aimes to determine the epidemiological characteristics of the cases of fatal poisoning in South, to identify the toxics used in oder to deduce the preventive measures.

**Patients and methods:** We conducted a retrospective study of all cases of fatal poisoning recorded in the forensic department of Habib Bourguiba University Hospital in Sfax, Tunisia, over a 12-years period (2006–2017).

**Results:** During the study period, 88 cases of fatal poisoning were autopsied. The number of victims recorded per year varied between 1 and 16 cases with an average of 8 cases per year. The average age was 30 years with extrems ranging from 7 months to 85 years. Nearly half (46.5%) were younger than 40 years. A male predominance was noted with a sex-ratio of 1.04. The majority of victims were single, low-educated and from rural origin. Personal antecedent of psychiatric pathology was found in 9.1% of cases. Psychotic disorders (schizophrenia) and depression were the most common pathologies. In our study we noticed that death occured every weekday without significant difference between days. However, the frequency of fatal poisoning was slightly higher in cold seassons (53.9%). In 71.6% of cases, victims were found dead at home. Accidental fatal poisoning was the most common (56%). No criminal cases have been observed. We noted a male predominance in accidental forms and a female predominance in suicidal forms. Carbon monoxide poisoning was the most common (35 cases) followed by the organophosphorus poisoning which was noted in 15 cases.

**Conclusion:** Decreasing the mortality rate from poison ingestion requires increasing public awareness about poisons and improving emergency service equipment and health personnel training.

**Compliance with ethics regulations:** Yes.

### P-126 Severe acute poisoning by organophosphate pesticides: report of 18 cases at the Oran hospital and university center

#### Mourad Goulmane

##### Hospital and university center of Oran, Oran, Algeria

###### **Correspondence:** Mourad Goulmane (goulmane.mourad@univ-oran1.dz)

*Ann. Intensive Care* 2020, **10(Suppl 1):**P-126

**Rationale:** Organophosphate pesticides are synthetic organic pesticides widely used in agriculture mainly as an insecticide, nemacid or acaricide. These are the agricultural products, the most incriminated in poisoning in our context. The objective of this work was to determine the clinical, paraclinical, and progressive characteristics of this poisoning in a resuscitation environment.

**Patients and methods:** Retrospective study of cases admitted to intensive care (January 2016–December 2018). Inclusion criteria were clinical, para-clinical, therapeutic and progressive.

**Results:** 18 cases were identified: 16 women and 2 men, mean age = 26.3 ± 10 years. The suicide attempt was the main reason for the intoxication (16 cases). The Glasgow coma score averaged 11 ± 4. The central syndrome was present in 80% of our patients, followed by muscarinic syndrome 75% and nicotinic syndrome in 58% of cases. Therapeutic management consisted of mechanical ventilation in 50% of cases, the use of vasoactive drugs in 20% of cases and the administration of antidotal treatment in 100% of cases. The overall mortality was 16.66%.

**Conclusion:** Organophosphate pesticides intoxication is a real health problem in Algeria. It is a serious condition dominated by the respiratory and neurological distress that causes most deaths. It concerns in our context especially young women who ingest the product for the purpose of autolysis. The diagnosis is based on the clinical and dosage of cholinesterase activity in the plasma. Treatment combines symptomatic measures that rely primarily on respiratory and neurological resuscitation to antidotal treatment. The clinical course in this type of intoxication is generally favorable under treatment with regression of signs in a few days. Mortality is high in our context, so it should be considered a diagnostic and therapeutic emergency. The commercial availability of these products is worrisome, justifying the use of a broad prevention program to inform the public and authorities of the danger of Organophosphate pesticides

**Compliance with ethics regulations:** Not applicable.

### P-127 Factors of gravity of ecstasy poisoning : 2 years experience in a toxicology hospital in Tunis

#### Hela Maamouri, Meriem Fatnassi, Hend Allouche, Nozha Brahmi

##### Service de réanimation toxicologique et polyvalente Centre Mahmoud Yaacoub, Tunis, Tunisia

###### **Correspondence:** Hela Maamouri (helamaamouri@yahoo.fr)

*Ann. Intensive Care* 2020, **10(Suppl 1):**P-127

**Rationale:** Ecstasy or MDMA (3,4-methylenedioxy-*N*-methylamphetamine) is a sympathicomimetic amine of the class of amphetamines widely consumed in rave parties. Several varieties of products known as ecstasy have been identified in Tunisia and have led to serious cases of poisoning. The objective of this study was to investigate the severity factors associated with acute ecstasy poisoning.

**Patients and methods:** It was a retrospective descriptive study including all patients hospitalized for acute ecstasy poisoning in intensive care unit of the Mahmoud Yaâcoub Center of Emergency Medical Assistance from January 1, 2016 to June 30, 2018. Poisoning was considered serious if one or more of the following factors were present: Glasgow score ≤ 8, use of mechanical ventilation, occurrence of seizures, hemodynamic failure, organ dysfunction (acute renal failure, liver injury, myocarditis, rabdomyolysis). The statistical analysis was based on the non parametric Mann Withney test.

**Results:** Ten patients were enrolled, aged on average 20 ± 4.9 years [15–30] with a clear male predominance (sex ratio = 9). Four patients were chronic users of psychoactive substances. The mean ingested dose was 2.4 tablets ± 2.87 with extremes of 1 and 10. Mean IGS II and APACHE II were 21.1 ± 16.30 [6–59] and 8.3 ± 9.25 [1–32]. The clinical examination revealed that five patients met the criteria for serious intoxication with the following signs: coma in four patients requiring the use of mechanical ventilation, seizures (n = 1), rhabdomyolysis (n = 3), shock (n = 1), toxic takotsubo (n = 1) and hepatocellular failure (n = 1) leading to patient’s death. The use of mechanical ventilation was necessary in 4 patients. The analysis of the severity factors did not show a statistically significant association between severity, age (p = 0.6), sex (p = 1) and chronic consumption of psychoactive substances (p = 0.52). On the other hand, we did not find a statistically significant association between serious intoxication, the number of tablets ingested (p = 0.7), the APACHEII score (p = 0.11) and the average length of stay (p = 0.11).

**Conclusion:** Ecstasy acute poisoning is becoming more common in our country and can potentially be very serious regardless of age, sex, medical history or number of tablets ingested. On the other hand, the concentration of NMDA could be the only factor to be taken into consideration upon admission.

**Compliance with ethics regulations:** Yes.

### P-128 The viper envenomations

#### Nissrine Tajellijiti, Youssef Bouidir, Fahd Moussaid, Youssef Elouardi, Mohamed Khallouki

##### Surgical resuscitation department, university hospital, Marrakech, Morocco

###### **Correspondence:** Nissrine Tajellijiti (dr.nissrina.taj5555@gmail.com)

*Ann. Intensive Care* 2020, **10(Suppl 1):**P-128

**Rationale:** The viper envenomations are a real public health problem in Morocco more deaths are reported each year. It is a medical-surgical emergency which can be daunting and life-threatening, as well as the patient’s functional prognosis.

**Patients and methods:** We present 25 cases of serious viper envenomation, on a retrospective study extended over a period of 9 years from 2010 to 2019 including one case with cobraic syndrome, and through a literature review we clarify the following aspects: epidemiological, pathophysiological, clinical and therapeutic. Inclusion criteria: the presence of traces of hooks with at least one locoregional and/or general sign of envenomation. The actual presence of the snake in question and/or its description by a witness or the victim.

**Results:** This is a retrospective study that interested 12 men and 13 women, mean age 41 years. The bites were due to vipers, the species was known cerastes cerastes type in two cases, Macrovipéra Mauritanica in one case, cobra was identified in one case. 13 patients had a consumptive coagulopathy with two cases of ischemic stroke, one case of hypovolemic shock and 4 cases of hemorrhagic shock, ten patients had compartment syndrome treated by emergency fasciotomy discharge. Eleven patients received anti venom serum with clinical improvement and reduction of complications.

**Discussion:** The poison of vipers is a chemical proteinaceous with two essential components: the toxins and enzymes. These proteins are responsible for the observed symptoms. The severity of envenomation is related to the plasma concentration of the venom. The definition of early clinical and biological criteria of gravity has envenomation better assess and clarify the therapeutic indications. Processing viper envenomation considerably simplified over the past decade. The medical care is based on a symptomatic therapy component associated with a specific serum therapy. On early treatment with these specific immunoglobulins from the onset of signs of grades II or III envenomation reduces morbidity, sequelae and the total cost of care.

**Conclusion:** Improving the prognosis of envenomation involves information education and good care that can only be achieved through close collaboration between clinicians, herpetologists, epidemiologists, toxicologists and specific serum producers.

**Compliance with ethics regulations:** Yes.

### P-129 Drug-induced hyperammonaemia: data from VigiBase, the WHO database

#### Nicolas Weiss, Joe Elie Salem, Benedicte Lebrun-Vignes, Kevin Bihan, Marika Rudler, Charlotte Bouzbib, Philippe Sultanik, Dominique Thabut

##### Sorbonne Université, Paris, France

###### **Correspondence:** Nicolas Weiss (nicolas.weiss@aphp.fr)

*Ann. Intensive Care* 2020, **10(Suppl 1):**P-129

**Rationale:** Altered consciousness secondary to metabolic encephalopathies represents a major cause of ICU admission. Ammonia dosage is thus recommended in the absence of any diagnosis. Despite hyperammonaemia is most commonly secondary to liver diseases, portosystemic shunts, inborn errors of metabolism, microbial pullulation, drug-induced hyperammonaemia (DIH) is another possible cause. DIH is poorly described. To describe the drugs associated with DIH.

**Patients and methods:** We used VigiBase, the WHO global Individual Case Safety Report (ICSR) database, which contains reports of suspected adverse drug reactions (ADRs) collected by national drug authorities in over 130 countries between 1967 and 8 May 2019. This observational retrospective study included all ADRs reported as “hyperammonaemia” according to the Medical Dictionary for Drug Regulatory Activities (MedDRAv21.1) term (Prefered term [PT] level). The drugs considered in the analysis were those notified as suspected treatments. Drugs used to treat hyperammonaemia or hepatic encephalopathy were excluded as were drugs reported less than 3 times. Drugs with a positive lower end of the 95% credibility interval for the information component (IC025) ≥0, an indicator value for disproportionate Bayesian reporting, was considered as causative of hyperammonaemia.

**Results:** Among 19 438 165 ICSRs, 576 drugs were identified for the term “hyperammonaemia[PT]”. Six were excluded because they were used to treat hyperammonaemia or hepatic encephalopathy. Thus, 73 drugs had a an IC025≥0 and represented 2759 cases (0.014%). Twelve drugs were reported more than thirty times: Valproic acid, Fluorouracil, Topiramate, Oxaliplatin, Asparaginase, Levofolinic acid, Paracetamol, Tacrolimus, Levetiracetam, Carbamazepine, Bevacizumab, Methotrexate.

**Conclusion:** DIH constitute rare causes of hyperammonamia. Nevertheless, these data could help in the etiological work-up of hyperammonemia.

**Compliance with ethics regulations:** Yes.

**Table 1:** Most frequent drugs responsable for hyperammonaemia

Substances	n	IC 0.25	Serious adverse events, n	Serious adverse events, %	Fatal adverse events, n	Fatal adverse events, %
Valproic acid	1260	6.86	768	61	54	4
Fluorouracil	221	4.27	213	96	20	9
Topiramate	96	4.33	65	68	0	0
Oxaliplatin	74	2.53	68	92	12	16
Asparaginase	71	5.09	50	70	6	8
Levofolinic acid	51	5.44	51	100	6	12
Paracetamol	48	0.95	38	79	16	33
Tacrolimus	37	1.80	36	97	25	68
Levetiracetam	35	2.21	25	71	3	9
Carbamazepine	35	1.48	24	69	0	0
Bevacizumab	34	1.43	34	100	3	9
Methotrexate	33	0.43	31	94	11	33

### P-130 Out-of-hospital cardiac arrest due to smoke inhalation: characteristics and outcomes

#### Christophe Allouache, Nicholas Heming, Virginie Maxime, Bernard Clair, Pierre Moine, Djillali Annane

##### Raymond Poincaré hospital, Garches, France

###### **Correspondence:** Christophe Allouache (allouache.christophe@outlook.com)

*Ann. Intensive Care* 2020, **10(Suppl 1):**P-130

**Rationale:** More than half of fire-related deaths are caused by smoke inhalation. We sought to determine the characteristics and outcomes of patients admitted to the ICU following an out-of-hospital cardiac arrest due to smoke inhalation.

**Patients and methods:** Monocenter observational study in the ICU of an academic hospital of the greater Paris area. Retrospective analysis of a prospectively constituted database. We included in the current study all patients suffering from an out-of-hospital cardiac arrest while in a burning building. Smoke inhalation was documented by the presence of soot in the upper and lower airways. All patients were initially treated by first responders and secondarily managed by a physician working with the French medicalized ambulance system, able to provide immediate life support.

**Results:** 36 patients were admitted to our ICU between January 2007 and December 2018, following an out of hospital cardiac arrest due to smoke inhalation. Median [IQR] age was 55 [41; 71] years. 19 (53%) patients were men. Cardiac arrest occurred in front of a witness in 4 (11%) cases. When data were available (n = 20; 55%), the initial rhythm was constantly an asystole. When data were available (n = 9; 25%), median No Flow was 20 [2; 20] min. Median Low flow was 14 [18; 24] min. Patients received a median intravenous dose of 3 [2; 5] mg of epinephrine. 5 (14%) patients suffered from second or third degree burns. 13 (36%) patients suffered from ARDS. 35 (97%) patients received hydroxycobalamin, median administered dose 6.5 [6.5; 10] g. Twenty-six (72%) patients were treated by hyperbaric oxygen therapy. The median length of stay in the ICU was 2 [0.3; 5] days. 5 (14%) patients survived. Among deceased patients, 10 (32%) died of brain death, 21 (68%) died of multiple organ failure.

**Conclusion:** Out of hospital cardiac arrest due to smoke inhalation is associated with major morbidity and mortality. In our cohort, only 14% of patients admitted to the ICU survived. Strikingly, a third of our cohort evolved towards brain death.

**Compliance with ethics regulations:** Yes.

### P-131 Diagnostic and prognosis values of cerebrospinal fluid analysis after cardiac arrest

#### Marine Paul^1^, Sarah Benghanem^2^, Sybille Merceron^1^, Hugo Bellut^1^, Anne Roche^1^, Mikhael Giabicani^1^, Florence Dumas^3^, Amandine Henry^4^, Paul Jaubert^2^, Fabrice Bruneel^1^, Jean Pierre Bedos^1^, Alain Cariou^2^, Stéphane Legriel^1^

##### ^1^Intensive Care Unit, Centre Hospitalier de Versailles-Site André Mignot, Le Chesnay, France; ^2^Intensive Care Unit, Cochin Hospital, Paris, France; ^3^Emergency Department, Cochin Hospital, Paris, France; ^4^Microbiology Department, Centre Hospitalier de Versailles-Site André Mignot, Le Chesnay, France

###### **Correspondence:** Marine Paul (marine.1604@hotmail.fr)

*Ann. Intensive Care* 2020, **10(Suppl 1):**P-131

**Rationale**

Lumbar puncture (LP) is one of the available tools of etiological diagnostic workup and prognostication after cardiac arrest (CA). However, few studies explored its potential interest in this setting. We aimed to report the diagnostic and prognosis values of LP after CA.

**Patients and methods**

Retrospective analysis of all consecutive patients admitted for CA presenting with a sustained return of spontaneous circulation in two French ICUs between 2007 and 2016 who underwent a LP to explore CA etiology. After exclusion of traumatic LP, we compared patients and cerebral spinal fluid (CSF) characteristics according to LP contribution for CA etiological diagnosis, according to LP abnormality, and to vital/functional status at ICU discharge.

**Results:** Among 1982 patients with ROSC, 65 (3.3%) patients who underwent a LP were included. LP provided an etiological diagnosis of CA from neurological cause in 6/65 (9%) cases, mostly in patients with neurologic prodromal symptoms before cardiac arrest (83%), and when performed in the post-mortem period (67%). LP was abnormal in 37/53 (69.8%) patients with LP non-contributory to etiologic diagnosis in which median CSF protein dosage was of 0.62 g/L (IQR, 0.54–0.76) and median CSF white cell count was of 2 elements per mm^3^ (IQR, 0–5). By univariate analysis, shorter time from collapse to CPR [0 min (0–3) versus 4 min (2–10), p = 0.004] and post-resuscitation shock [26 (70%) versus 5 (31%), p < 0.01] were significantly associated with abnormal LP findings. Patients with abnormal LP were more likely to be associated with poor outcome (CPC 3-4-5 in 27 (73%), p = 0.06).

**Conclusion:** LP is an uncommon exploration after CA, whereas it can provide an etiologic diagnosis in about 9% of cases. Non-specific CSF abnormalities can be frequently encountered after cardiac arrest, possibly in relation with blood brain barrier disruption, and may provide prognosis information.

**Compliance with ethics regulations:** Yes.

### P-132 Epidemiological, clinical, prognostic aspects of severe brain injuries in Yaoundé (Cameroon)

#### Roddy Stephan Bengono Bengono, Albert Ludovic Amengle, Junette Arlette Metogo Mbengono, Agnès Esiene, Paul Owono Etoundi, Jacqueline Ze Minkande

##### Faculty of medicine and Biomedical Sciences, University of Yaounde 1, Yaoundé, Cameroon

###### **Correspondence:** Roddy Stephan Bengono Bengono (rodbeng@yahoo.fr)

*Ann. Intensive Care* 2020, **10(Suppl 1):**P-132

**Rationale:** Brain injury is a major public health and socio-economic problem. The main cause is the road traffic accident. The aim of our study was to evaluate the epidemiological, clinical and prognostic aspects of severe brain injuries (SBT) in Yaoundé.

**Patients and methods:** It was a descriptive study with retrospective and prospective collection of data. The retrospective phase concerned the files of patients admitted from January 2013 to November 2018. The prospective phase concerned patients received from December 2018 to May 2019 in the intensive care units of two hospitals. We included all patients over 5 years with brain injury and a Coma Glasgow Score < 9, in whom the consent of a family member was obtained for the prospective phase. Patient’s data collection included: sociodemographic characteristics, clinical data and prognosis. Continuous variables were described with average, median and standard variation. The Chi square test was used for the association of variables. P < 0.05 was considered statistically significant.

**Results:** A total of 338 patients were recruited. The mean age was 37 ± 19 years. The sex-ratio was 3.77. Road accidents were found in 91.4% of cases. Out-of-hospital care is inexistent. Patients were taken to hospital by non-medical means. The majority were polytrauma patients (83%). Maxillo-facial lesions were the most common. The cerebral CT-scan was not available in all patients. The most neurological signs were mydriasis in 201 patients (59.5%) and seizures (22.5%). More than half of the patients, 51.8% of the cases, had a delay of care less than 12 h. Thirty eight patients (11.2%) received a surgical procedure. The infectious complications were the most common complication observed. The mortality rate was 59.2%. Factors associated with death were: age, motorcyclists, hypertension and hyperthermia.

**Conclusion:** SBT are frequent. Road traffic accidents are the main cause. The establishment of protocols and effective out-of-hospital care can improve the management and reduced the mortality of SBT.

**Compliance with ethics regulations:** Yes.

### P-133 Predictors of outcome in ICU patients with severe blunt chest trauma

#### Mariem Dlela^1^, Abir Bouattour^1^, Yassmine Kammoun^2^, Olfa Turki^1^, Mounir Bouaziz^1^

##### ^1^Habib bourguiba hospital, Sfax, Tunisia; ^2^Hedi chaker hospital, Sfax, Tunisia

###### **Correspondence:** Mariem Dlela (mariem241090@gmail.com)

*Ann. Intensive Care* 2020, **10(Suppl 1):**P-133

**Rationale:** Thoracic injury is a common and potentially devastating component of acute trauma care, globally accounting for about 10 to 20% of trauma admissions with mortality rates that could reach the quarter of early trauma-related mortality, in some series. Early identification of poor outcome predictors could be valuable to guide the most appropriate care. We aim to determine factors associated to mortality in patients with severe non-penetrating chest trauma admitted to the ICU.

**Patients and methods:** This is a prospective cohort study, including all patients with isolated severe blunt chest trauma (Abbreviated Injury Scale AIS > 3) admitted to the intensive care unit of a university hospital, over a one-year period. The primary objective was to analyse risk factors associated to death and poor outcome using univariate and multivariate analysis.

**Results:** One hundred-thirty patients were admitted to the ICU for blunt chest trauma among them 72 were diagnosed with severe isolated chest trauma and were included. The mean age was at 33 ± 18, mean ISS at 36 ± 15 and mean TTS at 7 ± 3. Twenty-eight (39%) patients were diagnosed with acute respiratory distress syndrome, 31 (43%) with post-traumatic acute kidney injury and fourteen (19%) with post-traumatic pulmonary embolism. The mean length of ICU stay (LOS) was at 17 ± 13 days and mean number of days on ventilator was at 10 ± 9 days. Thirty-two (44%) patients underwent elective tracheostomy for prolonged intubation. Thirty-seven patients (51%) developed infections, among them thirty (42%) were diagnosed with pulmonary infection and seven (9%) with non-thoracic infections. Overall mortality had an incidence of 16.7% (12 patients). The following variables were associated to ICU mortality on unvariate analysis: older age, higher TTS scoring, hemothorax, hemodynamic instability on admission, low LVEF and pulmonary hypertension on TTE exam and ARDS. On multivariate analysis, independent factors predictive of ICU mortality were ARDS (p = 0.034; OR = 4; CI 1.1–16), a LVEF less than 50% on admission (p = 0.02; OR = 11; CI 1.5–71), and shock state on admission (p = 0.003; OR = 7.7; CI 1.8–31).

**Conclusion:** Trauma with blunt severe thoracic injuries has been shown to be associated with an increased risk of morbidity and mortality in affected individuals. The identification of predictors from the initial onset of trauma could be valuable to guide the most appropriate medical care.

**Compliance with ethics regulations:** Yes.

### P-134 Early hyperglycaemia in traumatic brain injury: about 380 cases

#### Tayssir El Haj, Kamilia Chtara, Rania Ammar Zayani, Sabrine Bradai, Mabrouk Bahloul, Mounir Bouaziz

##### University of Sfax, Sfax, Tunisia

###### **Correspondence:** Tayssir El Haj (tayssir_elhaj@yahoo.com)

*Ann. Intensive Care* 2020, **10(Suppl 1):**P-134

**Rationale:** Early hyperglycaemia in traumatic brain injury (TBI) is a part of the stress response. It is an important indicator of severity and a reliable predictor of prognosis. We aimed to describe the epidemiological, clinical and paraclinical characteristics and to assess the prognostic impact of this hyperglycaemia on the TBI.

**Patients and methods:** We conducted a retrospective study in the intensive care unit (ICU) of our hospital between 2009 and 2012. Were included all patients with TBI and blood glucose > 8 mmol/L at the first 24 h post-trauma.

**Results:** During the study period, 694 patients were hospitalized in our ICU with TBI. Early hyperglycemia (> 8 mmol / l) was found in 380 patients (54.7%). In univariate analysis, glycaemia > 8.5 mmol/l (= 154 mg/dL) at admission was significantly associated with mortality (p = 0.015). We observed that glycaemia > 7.3 mmol/l at H12, > 7.2 mmol/l at H24, > 6.7 mmol/l at H36 and > 6.5 mmol/l at H48 was significantly associated with mortality (p = 0.07; p < 0.0001; p = 0.001 and p = 0.0008, respectively). The risk factors significantly associated with mortality were age > 32 years (p < 0.0001), SAPS II > 40 (p < 0.0001), initial shock (p < 0.0001), Glasgow Coma Scale (GCS) < 7/15 (p < 0.0001), coma period > 7 days (p = 0.023). The CT scan lesions statistically associated with mortality were: subdural hematoma (p < 0.0001), cerebral oedema (p < 0.0001), intra cerebral haemorrhage (p = 0.023), cortical contusion (p = 0.001), contusion of cerebral trunk (p = 0.011), contusion of the corpus callosum (p = 0.003), thalamus contusion (p = 0.004). In multivariate analysis, independent risk factors statistically associated with mortality were age > 32 years old (OR = 2.92 IC [1.52–5.78]; (p = 0.001)), glycaemia > 8.5 mmol/L at admission (OR = 2.25 IC[1.16–4.39]; (p = 0.017)),GCS < 7/15 (OR = 3.40 IC [1.8–6.24]; p < 0.001), intracerebral hematoma (OR = 3.2 IC [1.10–10.18]; p = 0.049).

**Conclusion:** We recommend a mandatory control of the blood glucose levels during a TBI with a target between 6.5 and 7.5 mmol/L in the acute phase.

**Compliance with ethics regulations:** Not applicable.

### P-135 Management of traumatic pneumothorax

#### Dalila Bougdal, Souhila Sadat, Dalila Zeghdoud

##### Hôpital Salim Zemirli, Alger, ALGERIA

###### **Correspondence:** Dalila Bougdal (dalilabougsrlf@gmail.com)

*Ann. Intensive Care* 2020, **10(Suppl 1):**P-135

**Rationale:** Thoracic trauma accounts for 1/3 of trauma admissions. Pneumothorax is a common post-traumatic lesion (10 to 50%). usually the only treatment is pleural drainage. Purpose of the study: to assess the outcome of patients with undrained pneumothorax

**Patients and methods:** Epidemiological prospective study, from January 2015 to December 2015, including all patients admitted to intensive care for closed chest trauma with a pneumothorax. We collected: age, severity scores, mechanism of the accident, physiological state of patients upon arrival in the emergency room, the lesion report and the associated lesions, treatment in ICU (drained and undrained groups), the indications of pleural drainage and the outcome of the monitored patients (clinical and radiological monitoring).

**Results:** Forty patients results were collected, mean age of 46 years, predominantly male, mean Glasgow score of 10/15.Respectively the mechanism of trauma was a public road accident, a traffic accident, a fall, in 52%, 35%, and 17%. Respiratory distress was present in 25% of cases, requiring mechanical ventilation. Pleural drainage was necessary in 32.5% of cases because of respiratory distress or for the purposes of anesthesia. Spontaneous resorption of pneumothorax was observed in 67% of cases. The evolution of patients who had not had pleural drainage was favorable and free of severe complication.

**Conclusion:** Chest drainage is a medical procedure with significant morbidity. The traumatic pneumothorax drainage indication depends on the effusion Importance, its clinical impact and the need for mechanical ventilation.

**Compliance with ethics regulations:** Yes.

### P-136 Management of cervical spine trauma at the CHU Sylvanus Olympio (SO) in Lome

#### Tabana Mouzou, Pilakimwe Egbohou, Pikabalou Tchetike

##### Anesthesia-Reanimation Service of Chu Sylvanus Olympio, Lomé, TOGO

###### **Correspondence:** Tabana Mouzou (essohanam2020@gmail.com)

*Ann. Intensive Care* 2020, **10(Suppl 1):**P-136

**Rationale:** To take stock of the management of CST in multi-function reanimation.

**Patients and methods**

Materials and methods: This was a descriptive and analytical retrospective study on the records of patients admitted to Multifunctional Reanimation at the CHU SO in Lomé from 1st November 2012 to 31th October 2015 for CST. The parameters studied included socio-demographic and diagnostic aspects at intake, associated lesions, therapeutic and developmental aspects.

**Results:** It involved 61 (74.40%) CST patients over a three years period. Younger adults were the most affected (70.37%) with male predominance (81.97%). Non-medical ambulance was the most used means of transportation (63.93%) to hospital. Road accidents were the leading cause (67.21%) of CST. The score of Fränkel A (68.85%) was the most found in terms of high severity. Dislocation fractures were the most frequent lesions (54.10%) associated with cranial-brain trauma, the main associated lesion (48.84%). CT scan was the most performed radiographic survey (90.16%). All the patients had medical treatment. Surgical treatment involved 23 patients (37.70%) and 38 patients (62.30%) had orthopedic treatment. The change was marked by a steady neurological state for 23 (37.70%) patients and 37 (60.60%) deaths. The leading causes of death were respiratory (45.90%), cardiovascular (29.50%) and bedsores (23%).

**Conclusion:** The management of CST at CHU SO was characterized by a very high death rate and inadequacies. However, there is a breakthrough in the specific management of this pathology with the presence of a neurosurgeon since 2008 and the new construction and equipment of multi-functional resuscitation since November 2012. Efforts in terms of strengthening the technical platform and equipment must be made to improve the management.

**Compliance with ethics regulations:** Yes.

### P-137 Fat embolism syndrome post-traumatic report of 24 cases

#### Mediouni Karim, Amine Raja, Aziz Bouhouri, Alharrar Rachid

##### University hospital IBN ROCHD Casablanca, Casablanca, Morocco

###### **Correspondence:** Mediouni Karim (Mediouni_karim@hotmail.com)

*Ann. Intensive Care* 2020, **10(Suppl 1):**P-137

**Rationale:** The syndrome of fat embolism (FES) is a set of clinical, biological, and radiological manifestations following the obstruction of the microcirculatory network by microdroplets of insoluble fats. It is a complication observed most often in the aftermath of polytrauma, including several long bone fractures, but it can also occur outside any traumatic context. The aim of this work is to address the different epidemiological, pathogenic, clinical, paraclinical, therapeutic, and progressive aspects of the fat embolism syndrome.

**Patients and methods:** Our study is based on a retrospective series including all cases of post-traumatic fat embolism collected in the surgical resuscitation department of the IBN ROCHD University Hospital Center of Casablanca, from January 2011 to December 2016. The diagnosis of FES in our patients was established from a bundle of clinical (modified Gurd criteria and Schonfeld index), biological, and radiological criteria.

**Results:** 24 cases of post-traumatic fat embolism were collected from January 2011 to December 2016. Male sex, age < 40 years, and femur fracture were the dominant features of the profile epidemiologic of the traumatized presenting this syndrome occurring mainly within 72 h of the trauma. Clinical-biological presentation were mainly respiratory distress, disturbances of consciousness, anemia, thrombocytopenia, and hypocholesterolemia. Radiologically, diffuse and bilateral alveolar opacities were the most common positive signs; cerebral CT was most often normal. The management of FES was symptomatic, associating early immobilization of fracture sites, optimal analgesia, and the maintenance of effective blood volume. 41.66% of the patients required the use of mechanical ventilation. 75% of our patients underwent osteosynthesis of their fracture centers, of which 62.5% by ECM and 12.5% by other types of osteosynthesis. The incidence of ARDS in our series was 25%. The evolution was not always favorable with a mortality rate of 26.08%.

**Conclusion:** The diagnosis of FES is essentially clinical but often difficult because of its polymorphic presentation and the absence of specific paraclinical examination. In its complete form (Gurd Triad), it associates respiratory, neurological, and mucocutaneous manifestations. These signs are most often not particularly specific in a context of polytrauma, which makes it a diagnosis of exclusion. Management is essentially prophylactic and involves early fixation of fracture sites. The treatment of a declared EG remains symptomatic.

**Compliance with ethics regulations:** Not applicable.

### P-138 Post-trauma fat embolism syndrome

#### Naila Boukoub, Mouna El Idrissi, Taha Hounain, Sara Maaroufi, Youssef Elouardi

##### Surgical ICU Department, Ibn Tofail Hospital, Marrakech, Morocco

###### **Correspondence:** Naila Boukoub (naila.boukoub@edu.uca.ac.ma)

*Ann. Intensive Care* 2020, **10(Suppl 1):**P-138

**Rationale:** The Fat Embolism Syndrome (FES) is a set of clinical, biological and radiological signs resulting in the obstruction of micro-circulation by micro-droplets of insoluble fats.The clinical signs of the FES are not very specific, the diagnosis is difficult and the risk of misunderstanding this syndrome is very real.The FES appears after a trauma, often few days later. However, it sometimes occurs without previous trauma; and it is particularly difficult to recognize in these cases. The aim of this work is to define the epidemiological profile, the clinical and para-clinical features of this syndrome and its therapeutic management.

**Patients and methods:** This is a monocentric retrospective study, performed in the surgical ICU department at Ibn Tofail Hospital in Marrakesh. We included all hospitalized FES patients in our department from January 2016 to July 2019.We studied the epidemiological, clinical, therapeutic characteristics. To study the prognostic factors, we subdivided our sample into survivors and deaths. Qualitative variables were expressed as a percentage and compared by Chi-2 or Fisher test. Quantitative variables were expressed as mean (± standard deviation) or median (percentile), and compared by Student’s t-test or Mann-Whitney test. For multivariate analysis, we used multiple logistic regressions using SPSS version 10 for Windows. A p-value < 0.05 was considered as significant.

**Results:** We collected 9 hospitalized patients for FES during the study period. The mean age was 30 ± 19 with a male predominance of 90%, femur fracture was present in 78% of cases, with bilateral fractures in 1 case.This syndrome occurs in the majority of the cases within 72 h after the trauma, road traffic accidents was the only cause observed in our series. Clinically, 90% of patients were hypoxemic, one of them developed acute respiratory distress syndrome, neurological signs were present in 85% of cases, and petechiae were observed in 7 patients. Management was mainly symptomatic, including in all cases oxygen, vascular filling and adequate analgesia. Intubation and artificial ventilation were performed in 78% of cases. Death occurred in one case following a fulminant form and only one patient kept neurological impairment shown as agitation up to 1 month later.

**Conclusion:** Fat embolism syndrome remains a classic diagnosis but the frequency seems to be decreasing now. The diagnosis remains primarily clinical, based on the history taking and the group of evocative clinical signs. The management remains symptomatic sometimes requiring artificial ventilation. Early treatement of long bone fractures is the best way to prevent this syndrome.

**Compliance with ethics regulations:** Yes.

### P-139 Sedation-analgesia among elderly patients in Intensive care unit: about 30 cases

#### Maha Hammami, Kamilia Chtara, Olfa Turki, Najeh Baccouche, Mabrouk Bahloul, Mounir Bouaziz

##### University of Sfax, Sfax, Tunisia

###### **Correspondence:** Maha Hammami (dr.hammamimaha@yahoo.fr)

*Ann. Intensive Care* 2020, **10(Suppl 1):**P-139

**Rationale:** Sedative and analgesic treatment administered to critically ill patients with mechanical ventilation need to be regularly assessed to ovoid complications of oversedation mainly in elderly patients. Our objective is to evaluate our sedation practice in the elderlyin our unit

**Patients and methods:** It was a prospective observational study, including elderly patients over 65 years of age without acute brain injury requiring sedation more than 48 h of hospitalization in the intensive care unit of our University Hospital between April 2018 and December 2018.

**Results:** Thirty patients were included. The aged was 73.3 years, the sex ratio was 1.7. Respiratory distress was the most common reason for hospitalization 53%. The most accepted diagnoses were the decompensation of COPD in 34% of cases and septic shock in 23% of cases. The SAPS II averaged 58 ± 12 points, SOFA averaged 10 ± 2.4 points. Renal failure was found in 21 patients (70%), hepatic impairment was noted in 7 patients (23%), hypoproteinemia was marked in 17 patients (57%). Midazolam was used in 90% of patients. It was in combination with fentanyl in 63% of cases and remifentanyl in 30% of cases. The median Ramsay score 5.7 ± 0.6 on the first day of sedation and 5.3 ± 0.7 on the second day of sedation. The median RASS scale was − 4.7 ± 0.6 on the first day of sedation and − 4.4 ± 0.8 on the second day of sedation. The median BPS scale 3.6 ± 1.4 on the first day of sedation and 3.6 ± 1.2 on the second day of sedation. The mean wake up time was 6 ± 3, 38 days. Neuromyopathy of resuscitation was suspected in seven patients (20%), withdrawal syndrome was observed in two patients (7%) and acute cognitive dysfunction in two patients (7%). The median duration of sedation was 4.2 days ± 1.9 days, the median duration of mechanical ventilation was 11.1 ± 6.3 days, the median length of stay was 14.3 ± 9.8 days. Ventilator-associated pneumonia was diagnosis among 79% of patients. The mortality in intensive care was 37%.

**Conclusion:** Sedation analgesia in the elderly person should be adapted according to age, ideal weight and renal and hepatic function by decreasing the initial doses. It should be evaluated by the recommended scores by setting a sedation objective according to the pathology.

**Compliance with ethics regulations:** Not applicable.

### P-140 Causes and risk factors of death of patients admitted in two intensive care units in Yaoundé (Cameroon)

#### Roddy Stephan Bengono Bengono, Junette Arlette Metogo Mbengono, Albert Ludovic Amengle, Agnès Esiene, Paul Owono Etoundi, Jacqueline Ze Minkande

##### Faculty of Medicine and Biomedical Sciences, University of Yaoundé 1, Yaoundé, Cameroon

###### **Correspondence:** Roddy Stephan Bengono Bengono (rodbeng@yahoo.fr)

*Ann. Intensive Care* 2020, **10(Suppl 1):**P-140

**Rationale:** Mortality is a major end point in epidemiologic and interventional studies in intensive care units (ICU). It is an Indicator of performance and good practices. The purposes of our study was to determine the causes and risk factors of death of patients admitted in ICU.

**Patients and methods:** We carried out a cross sectional study with retrospective collection of data in two hospital of Yaoundé’s city. Patient’s data collection included socio-demographic characteristics, clinical informations, mortality rate, causes and risk factors of deaths. Continuous variables were described as average, median, standard deviation and interquatile range. Categorical variables have been described as percentages, proportions and/or frequencies. The Chi square test was used for the association of qualitative variables. P < 0.05 was considered statistically significant. The degree of association was measured by calculating the odds ratio with their 95% confidence interval.

**Results:** Our sample consisted of 622 patients admitted to ICU, including 311 deceased patients and 311 surviving patients. The mortality rate was 14.6%. The mean age of the patients was 43.96 ± 25.4 years. The highest mortality rate was in the 60-74 years age group. Female gender accounted for 64% of deaths. The most common reason for admisssion was impaired state of consciousness (48.2%). Medical causes accounted for 81.3% of deaths and surgical causes (18.7%). The main Medical causes of death were represented by sepsis (24.5%) and stroke (17.8%). The main surgical causes were represented by brain injuries (34.5%) and thermal burns (34.5%). The risk factors found were the age (> 60 years), the history of hypertension and diabètes, the occurrence of infectious complications.

**Conclusion:** Mortality remains high in the ICU studied. Medical causes (sepsis and stroke) are the major causes of death. Better organization in care and good practices in hospital can allow the reduction of mortality.

**Compliance with ethics regulations:** Yes.

### P-141 Journal club in a French ICU: a drop in the ocean of medical literature. Description and clinical impact of 1712 articles read over a 12-year period (2007-2019)

#### Damien Contou^1^, Marina Thirion^2^, Olivier Pajot^1^, Gaëtan Plantefeve^4^, Hervé Mentec^1^

##### ^1^Centre Hospitalier Victor Dupouy, Argenteuil, France; ^2^Centre Hospitalier du Bassin de Thau, Sète, France

###### **Correspondence:** Damien Contou (damien.contou@ch-argenteuil.fr)

*Ann. Intensive Care* 2020, **10(Suppl 1):**P-141

**Rationale:** More than 2500 original articles are newly indexed in PubMed every day. Journal Club (JC) is one way to cope with this abyssal amount of medical information. We aimed at (1) describing journals and articles analyzed during our JC sessions (2), reporting the proportion of published articles being analyzed during JC sessions and (3) assessing the clinical impact on our daily practices for each journal.

**Patients and methods:** A retrospective analysis of prospectively collected data over a 12-year period from 2007 to 2019 in a university-affiliated ICU. JC sessions were scheduled weekly and participants were free to choose and expose orally an article recently published in any medical journal (general, ICU or non-ICU specialized). Clinical impact of a journal was retrospectively and independently assessed by two attending intensivists (DC, HM) and was defined by the ratio of articles considered as having a direct impact on our daily practices over the number of articles of the same journal read during the same period.

**Results:** From August 2007 to August 2019, 313 JC sessions were held and 1712 articles—mostly original (n = 1657/1712; 97%)—from 93 journals were analyzed, accounting for 0.01% of the 11869960 articles (0.02% of the 10982188 original articles) referenced in PubMed during the same period. Median number of articles exposed per session was 6 [4–7]. Median number of doctors attending each session was 7 [6–8] (attendings: 2 [2–3], fellows: 1 [1–2], residents: 3 [2–3]). General, ICU and non-ICU specialized journals accounted for 32%, 47% and 21% of the exposed articles, respectively. Most of the reported articles dealt with intensive care (n = 1177, 69%) especially infectious diseases (n = 286/1177; 24%), hemodynamics (n = 123/1177; 10%) or ICU-organization (n = 111/1177; 9%). Compared to general and non-ICU specialized journals, the proportion of read-over-published articles was higher for ICU-specialized journals (0.18% vs. 0.13% vs. 2.61%, respectively; p < 0.0001). Among original articles, only 93 (5.9%) [interventional (n = 61/93; 66%); observational (n = 32/93; 34%) studies] were considered as having a clinical impact on our daily practices. Compared to ICU and non-ICU specialized journals, general journals had a higher clinical impact (4.3% vs. 4.9% vs. 9.1%, respectively; p = 0.001). Data regarding the 3 most read general, ICU and non-ICU specialized journals are detailed in Table 1.

**Conclusion:** In a French university-affiliated ICU with regular JC sessions, the proportion of read-over-published articles and the clinical impact of medical journals appear minor. In the ocean of medical literature, general medical journals appear more worth reading by intensivists than ICU-specialized journals.

**Compliance with ethics regulations:** Yes.

**Table 1:**
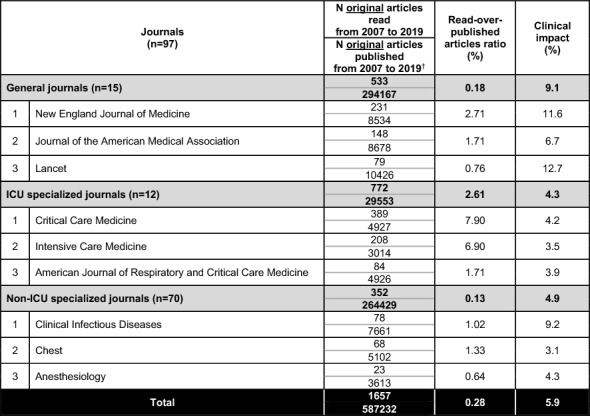
Proportion of read-over-published articles and clinical impact of the 3 most read general, ICU and non-ICU specialized medical journals († number of original articles published from August 2007 to August 2019 only in the 97 journals read during our JC sessions)

### P-142 Evolution and prognostic factors of patients aged 80 and over admitted to surgical ICU

#### Gautier Nitel, Anne Bignon, Eric Kipnis

##### CHU Lille, Lille, France

###### **Correspondence:** Gautier Nitel (gautier.nitel@gmail.com)

*Ann. Intensive Care* 2020, **10(Suppl 1):**P-142

**Rationale:** The world’s population is aging and the 80 and over’s age group is growing fast (+ 3.8% per year). This aging population is impacting Intensive care units with exponential rates of elderly patients (14.4% in 2011, 18% in 2017), associated with significant mortality (from 15% to 50%). The evolution and the prognostic factors of these elderly patients in intensive care are therefore a public health issue for optimal management.

**Patients and methods:** We included all patients aged 80 and over who were operated and admitted to surgical resuscitation in our center, with a duration of stay greater than 24 h, from April 2016 to July 2018. The data collected were: general characteristics of this population, mortality in intensive care, at day 30 and at 6 months and the prognostic factors guiding their evolution in intensive care and at 6 months.

**Results:** Of the 102 patients included in our study, mortality was 31.4% in intensive care, 42.2% at day 30 and 60.8% at 6 months. The prognostic factors in the intensive care unit were the average dose of NORADRENALINE at Day 1 (threshold at 0.65 mg/h), the SOFA score at Day 1 (threshold at 6 points) and the IGS2 score (threshold at 66 points). The prognostic factors at 6 months were ventilatory autonomy on Day 1 (spontaneous ventilation, non-invasive ventilation, invasive ventilation), the reason for admission to intensive care (acute respiratory distress or septic shock) and the fragility score (Clinical Failure Scale with a threshold at 4).

**Conclusion:** The mortality of patients aged 80 and over is influenced by prognostic factors easily obtained daily at patient’s bed. These prognostic factors could be an aid for the resuscitation teams to evaluate the relevance of the care undertaken in elderly or even very elderly patients admitted in an acute situation.

**Compliance with ethics regulations:** Not applicable.

### P-143 Assessing patient safety culture perception in the intensive care unit in Tunisia

#### Oussama Jaoued, Chaoueh Sabrina, Sik Ali Habiba, Wael Chemli, Gharbi Rim, Fekih Hassen Mohamed, Elatrous Souheil

##### Hôpital Taher Sfar, Mahdia, Tunisia

###### **Correspondence:** Oussama Jaoued (oussamajaoued@gmail.com)

*Ann. Intensive Care* 2020, **10(Suppl 1):**P-143

**Rationale:** In Tunisia health care system, patient safety has become a priority of quality assessment. The aim of our study was to describe the safety culture perception of the intensive care unit staff.

**Patients and methods:** The safety attitude questionnaire (SAQ-ICU) was distributed to all intensive care unit staff by email. The questionnaire explores 6 safety culture domains: “team work”, “safety climate”, “job satisfaction”, “stress recognition”, “perception of the hospital and intensive care unit management” and “work condition”.

**Results:** Eighty participants responded to the questionnaire, 61% of them were women. Participants were doctors in 62.5%. The coordination between physicians and nurses was very good only in 21%. Thirty-nine participants thought that the workload was high and 41% like their work. Medical errors are handled appropriately in 26% of cases and it was difficult to discuss errors in 19% of cases. The hospital is a good place to work in 5% of participants, 28% of participants were less effective at work when there were tired. The hospital did a good effort of training new personal in 18% of cases. The number of medical staff was lower than expected in 82% of cases. Half of participants would feel safe being treated as patients in their respective units. All domains explored by SAQ-ICU could be improved according to attendants.

**Conclusion:** Safety culture perception among intensive care unit staff had several deficiencies, mainly the working conditions, the ignorance of medical error reporting procedures and the lack of communication.

**Compliance with ethics regulations:** Yes.

### P-144 Use of the simplified acute physiology score II (SAPS II) at the Emergency Department

#### Hadil Mhadhbi, Khedija Zaouche, Yosra Yahya, Abdelwahab Mghirbi, Kamel Majed

##### Emergency Department la Rabta, Tunis, Tunisia

###### **Correspondence:** Hadil Mhadhbi (hadil.mhadhbi@gmail.com)

*Ann. Intensive Care* 2020, **10(Suppl 1):**P-144

**Rationale:** The Simplified Acute Physiology Score II (SAPS II) is an ICU scoring system used to predict the mortality risk in patients presenting at the ICU. However the majority of critically ill patients present initially at the ED and their transfer to the ICU may be delayed for hours. Therefore, the ability to accurately assess mortality risk at ED may have a great impact. The purpose of this study was to evaluate the performance of SAPS II in predicting early and late mortality in ED patients.

**Patients and methods:** This prospective study was conducted at the ED during a 6-month period. Data for adult ED patients were evaluated. SAPS II score was used to predict early and late mortality rates at 48-h and 30-day respectively. Discrimination was evaluated by calculating the area under the receiver operating characteristic curve (AUROC).

**Results:** During the study period 200 patients were enrolled. The mean age was 65 ± 10 years, 52% of the patients were men. The mean SAPS II was 30. The early mortality rate was 20% and late mortality rate was 15%. SAPS II was efficient in predicting early mortality, with an AUROC of 0.84 (95% CI 0.79–0.89). However, it demonstrated no value in predicting late mortality with an AUROC of 0.44 (95% CI 0.39–0.49)

**Conclusion:** In this study, SAPS II score was accurate in predicting early mortality, however this tool appears less suitable for predicting late mortality.

**Compliance with ethics regulations:** Yes.

### P-145 Outcomes of very elderly (≥ 80 years) critical-ill patients in a tunisian medical intensive care unit

#### Oussama Jaoued, Chaoueh Sabrina, Sik Ali Habiba, Yosri Ben Ali, Fekih Hassen Mohamed, Elatrous Souheil

##### Hôpital Taher Sfar, Mahdia, Tunisia

###### **Correspondence:** Oussama Jaoued (oussamajaoued@gmail.com)

*Ann. Intensive Care* 2020, **10(Suppl 1):**P-145

**Rationale:** The aging of the population increased the number of hospitalizations in ICU. The aim of our study was to determine the impact of hospitalization of patients over the age of 80 on morbi-mortality and consumption of care (omega score).

**Patients and methods:** This is a retrospective study carried out in the ICU in the hospital of Taher Sfar in Mahdia over a period of 17 years. All patients hospitalized in the ICU were included in this study. Two groups of patients were individualized: G1: patients over 80 years old, G2: patients under 80 years old.

**Results:** During the study period, 4053 patients (3664 < 80 years old and 389 ≥ 80 years old) with a mean age 56 ± 20 years and with a mean SAPSII 32 ± 19 were included. The common reason for hospitalization was acute respiratory failure in 53% of cases. Comparing the two groups, the severity score SAPSII was higher among patients older than 80 years (43 ± 16 vs 30 ± 18, p < 0.001). The use of mechanical ventilation was more common in the first group (67% vs. 55%, p < 0.001). The incidence of nosocomial infections was similar in both groups (12% in the group G2 and 14% in group G1, p = 0.2) and the use of renal replacement therapy was also similar in tow groups (7% in the G2 group and 6% in the G2 group, p = 0.3). The duration of mechanical ventilation and length of stay were similar between the two groups. Workload evaluated by the OMEGA score was higher in the first group (57 [IQR 30–131] vs 46 [IQR 21–115], p < 10^−3^). Mortality was 35% in G1 against 19% in G2 (p < 10^−3^). In multivariate analysis age over than 80 years was associated with mortality [OR = 1.4 IC (1.036-1.788), p = 0.02]. The other factors independently associated with mortality were: previous health status (ESA) [OR = 1.8 IC (1.517–2.215); p < 0.001], use of mechanical ventilation [OR = 4.9 CI (3.7–6.5); p < 10^−3^], renal replacement therapy [OR = 3.2 IC (2.4–4.2), p < 0.001] and occurrence of nosocomial infection [OR = 2.2 IC (1.6–2.9); p < 0.001].

**Conclusion:** In our study, age over 80 years was associated with a heavy workload. Age over 80 years was a factor independently associated with mortality.

**Compliance with ethics regulations:** Yes.

### P-146 Assessing the need for a rapid response system: patterns of deteriorating ward patients admitted to the ICU and of Chain of Care

#### Myrtille Gaudel^1^, Mathilde Masson^2^, Johanna Temime^1^, Nicolas Van Grunderbeeck^1^, Clément Delpierre^1^, Charles Detollenaere^1^, Julien Marc^1^, Didier Thevenin^1^

##### Centre Hospitalier de Lens, Lens, France; ^2^Centre Hospitalier d’Arras, Arras, France

###### **Correspondence:** Myrtille Gaudel (myrtille.gaudel@gmail.com)

*Ann. Intensive Care* 2020, **10(Suppl 1):**P-146

**Rationale:** ICU outcome depends on quality of pre-ICU care. We aimed to assess the chain of care of Deteriorating Ward Patients (DWP), through evaluation of preadmission severity and delays before admission, and association with outcome.

**Patients and methods:** Retrospective observational study in a single center (750 beds general hospital) for 1 year-may 15th of 2018 to 2019. All adult patients admitted in the ICU from the wards were included, except for scheduled surgery, or unexpected event in the operative theater. Preadmission severity was assessed through levels of National Early Warning Score 2 (NEWS2): Group 1 with NEWS2 inferior to 5, group 2 with NEWS2 between 5 and 7, and group 3 with NEWS2 superior to 7. These scores were established from vital signs during the 48 h before ICU admission. Patterns of patients, including SOFA and SAPS2, Knaus index, Charlson comorbidity score, cause of admission and technics used in the ICU, length of stay in the ICU and in the hospital, limitations of life-supporting care, and mortality at 30 and 90 days after ICU stay. Satistical analysis was performed through CHi^2^ and Fisher tests on qualitative parameters, and with Kruskal-Wallis, Student and Mann-Whitney tests for quantitave data.

**Results:** Sixty-eight patients were studied: 23 in group 1, 21 in group 2 and 24 in group 3. Most patients (all except 9) had not respiratory rate monitoring before ICU admission. ICU mortality was associated with rising preadmission severity (group 1: 8.7%; group 2: 38.1%; group 3: 45.8%). Base patterns (Charlson comorbidity score, Knaus index) did not differ between the 3 groups, and 61.8% of patients presented with sepsis. Main causes of admission were respiratory (45.5%), hemodynamic (25%) or neurologic (17.7%) failures. All patients admitted after cardiac arrest resuscitation (8 patients) belonged to group 3. Acute severity scores (SOFA and SAPS2) followed preadmission severity. Limitation or withdrawing of life support in the ICU was higher in group 3 (62.5%) than in groups 2 (30%) and 1 (8.7%). Median delay between first NEWS2 equal or superior to 5 and ICU admission was 14 h, and 9 h between NEWS2 equal or superior to 7. Diffrences in delays were not associated with outcome.

**Discussion:** Our study outlines weaknesses in the chain of care of DWP. Emphasis should be put on respiratory rate monitoring and better assessment of severity.

**Conclusion:** Chain of Care of detriorating ward patients must be optimized. This relies on education, better monitoring of vital signs and lower delays of response.

**Compliance with ethics regulations:** Yes.

### P-147 Forty year-old and under critically ill patients dying early after admission despite full intensive care: epidemiology, clinical characteristics and financial impact over 12 years

#### Clémence Perrin, Guillaume Morel, Florence Fagot-Gandet, Valentin Morandeau, Claire Lacan, Pierrick Le Borgne, Vincent Castelain, Francis Schneider

##### Médecine Intensive Réanimation, Hôpital de Hautepierre, Hôpitaux Universitaires de Strasbourg, Strasbourg, France

###### **Correspondence:** Clémence Perrin (clemenceperrin@ymail.com)

*Ann. Intensive Care* 2020, **10(Suppl 1):**P-147

**Rationale:** Access to critical care is controversial in older patients for 2 reasons: lack of available ICU-beds and speculation on induced costs. In contrast, admission of young patients aged 40 or under is infrequently questioned even though they develop catastrophic multiple-organ failure requiring full care. In addition, emotive reaction triggered in staff by these patients often represents a heavy psychological burden when ICU-stay is < 48 h. Information on the epidemiology, clinical information and induced costs regarding such patients is lacking.

**Patients and methods:** This study retrospectively assessed the records of patients aged 40 or under, and admitted from January 2008 to August 2019. Cost-related expenses charged to care-payers were obtained from our Medical Information Department. Data (number, percentages or medians) were reported and discussed by comparison with those of nonagenarians during the same period.

**Results:** Of 10,948 ICU-admissions, 1347 were aged 40 or under (13%), 117 of whom (8.7%) died within the ICU, with 41 (35%) dying within 48 h of admission despite full intensive care. The latter represent our study population (0.4% of the screened population). The median age was 32.8 years [IQR: 17.7-40], male gender was prevalent (61%). Half the patients (n = 20, 48%) were referred from the emergency department, 11 (26.8%) from hematology, 3 from oncology (7.3%), 6 from medical intermediate care units (14.6%), and one from digestive surgery (2.4%). The first diagnosis at admission was septic shock (n = 17, 41.4%), followed by post-anoxic encephalopathy (n = 13, 31.7%), coma (n = 5, 12.2%), acute respiratory failure (n = 4, 9.8%) and cardiogenic shock (n = 2, 4.9%). SAPSII was 71 [IQR: 43–112], and SOFA 12.5 [IQR: 5–20]. All patients were ventilated and infused norepinephrine. Two patients underwent ECMO, and 2 others MARS. Mean (± SEM) retribution per stay was 13,475 ± 3565 €, and mean retribution per “day of stay” 6737 €.

**Discussion:** Full care of these ICU-patients, with early mortality has a financial impact similar to that of nonagenarians at 13,160 ± 11,070 €; the cost per “day of stay” is therefore on average 100% higher than that of nonagerians (mean length of stay: 6.3 days), and, in our experience, 50% higher than that of average patients.

**Conclusion:** ICU-patients aged 40 or under represent a small percentage of admissions and display half our overall mortality: one third of them die within 48 h of admission with a not insignificant financial impact for cost-payers. Septic shock is the first cause of referral, followed by unexpected cardiac arrest.

**Compliance with ethics regulations:** Yes.

### P-148 Blood transfusion: modalities and risks -Intensive care unit A1-

#### Ihssan Mehrez, Abdelkrim Shimi, Ali Derkaoui, Mohhamed Khatouf

##### CHU HASSAN II, Fes, Morocco

###### **Correspondence:** Ihssan Mehrez (mehrez.ihssan@gmail.com)

*Ann. Intensive Care* 2020, **10(Suppl 1):**P-148

**Rationale:** Blood transfusion (BT) is a therapy that consists of administering labiles blood products intravenously: LBP (globular concentrates (CG), fresh frozen plasma (CFP) or platelet concentrates (PC). Many procedures have been established to improve transfusion safety by AFSAPS and ANAES (products, indications and contraindications).

**Patients and methods:** This is a prospective study, analyzing 68 cases of BT either at the Intensive care unit A1,or at the A2 central block of the HASSAN II University Hospital in Fez, over a period of 4 months from November 2018 to March 2019. The objective of the study is to compare the modalities of BT against the latest recommendations and to detect the resulting complications.

**Results:** 68 patients havinfg BT were collected between November and March 2018–2019 in intensive care A1 . The average age is 42 years with extremes ranging from 4 to 86 years. The transfused population with a previous history is 10 cases (14.7%). The indications for transfusion in ICU patients included: 25% post-op bleeding from CVS, 23% with HB objective in CT, 22% anemia poorly tolerated with hypoTA and tachycardia, 13% hemorrhagic EDC, 7% Stress ulcer, 2.5% RD, 2.5% with septic shock objective and 5% by excess. In the operating room, the indications for BT were dominated by: intraoperative bleeding 41%, tachycardia with hypotension 30%, post CEC deglobulization 21%, intraoperative hemorrhagic choc 3.5%, intraoperative cardio respiratory arrest 3.5%, and finally by excess 1%. The average initial Hb is 7.2 g/dl, for a groupage: 50% A+, 36.7% O+, 4.4% B+, 4.4% B-, 2.94% AB-, and 1.47% AB+. The amount of LBP transfused was an average of 2CG /7CQ/3PFC, over a period of 15–40 min. For the modalities of BT: the cross match was done for 7 patients in ICU (18% of cases), and for 2 patients of CVS in block A2 (7% of patients), we had 2 anti-B agglutinations (2.34%): one in ICU patient AB+, and one in patient block A+, for each one blood bags have been changed. For the 68 patients, the BT was marked by the occurrence of 2 incidents or 2.95% of cases; one case of fever and another of hemolysis with hematic urine. The BT was stopped for both accidents.

**Conclusion:** The main complications of BT are non-hemolytic febrile reactions and allergies, with an immunological risk > infectious risk. The transfusion of labile blood products, a highly codified medical procedure, would require the implementation of appropriate procedures.

**Compliance with ethics regulations:** Yes.

### P-149 Value of the CURB-65 score in predicting ICU admission and in-hospital mortality in patients with sepsis

#### Hadil Mhadhbi, Yosra Yahya, Abderrahim Achouri, Khedija Zaouche, Kamel Majed

##### Emergency Department la Rabta, Tunis, Tunisia

###### **Correspondence:** Hadil Mhadhbi (hadil.mhadhbi@gmail.com)

*Ann. Intensive Care* 2020, **10(Suppl 1):**P-149

**Rationale:** Severity scores in patients with sepsis are useful for triaging and predicting mortality. Mortality in Emergency Department Sepsis (MEDS) score is validated in patients with sepsis in the emergency department. CURB-65 is validated in patients with community-acquired pneumonia but not in sepsis. CURB-65 is a simple bedside tool that has many common elements with new sepsis identification score-q SOFA. The study aimed to assess the accuracy of CURB-65 score in predicting ICU admittance and mortality compared to MEDS score.

**Patients and methods:** This prospective study was conducted at the ED during a 6-month period. We enrolled all adult patients with sepsis admitted to the ED. MEDS and the CURB-65 scores were calculated at admission. Patients were studied using CURB-65 score and their ICU admission and in-hospital mortality were ascertained.

**Results:** A total of 120 patients were enrolled. The mean age was 60 ± 10 years. 60% of the patients were men. 30% of patients had a CURB-65 score ≥ 3 points with a mean MEDS score of 13%. Among these patients, 40% were admitted to ICU and 30% died. The CURB-65 score,was efficient in predicting both ICU admittance and in-hospital mortality with an AUROC of 0.84 (95% CI 0.79–0.89) and 0.73 (95% CI 0.67–0.79), respectively.

**Conclusion:** A higher CURB-65 score was correlated with higher rates of ICU admittance and mortality in patients with sepsis due to any cause.

**Compliance with ethics regulations:** Yes.

### P-150 Impact of comorbidities on outcomes in sepsis in emergency department

#### Abderrahim Achouri, Hadil Mhadhbi, Khedija Zaouche, Hamida Maghraoui, Radhia Boubaker, Kamel Majed

##### University Hospital Center Rabta of Tunis, Tunis, Tunisia

###### **Correspondence:** Abderrahim Achouri (achouryabderrahim@gmail.com)

*Ann. Intensive Care* 2020, **10(Suppl 1):**P-150

**Rationale:** Sepsis is a major cause of mortality. In other hand, pre-existent chronic diseases seem to worsen outcomes among critically ill patients. The acknowledgement of this fact may motivate studies in this type of situations in order to improve survival in sepsis. On that purpose, our study tried to check the impact of chronic pre-existent illnesses on outcomes in this type of emergency patients.

**Patients and methods:** We have included patients in whom the sepsis-3 definition was met throughout emergency department admission cases for infection. In this study, considered outcomes were in-hospital mortality, shock occurence and the use of mechanical ventilation.

**Results:** We collected 93 patients admitted to ED for sepsis. Mean age was 65 years ± 14 with bornes of 24 and 92. Men were 57% of the patients. Cormorbidities were: insulin dependent diabetes mellitus in 11.1% of patients, non insulin dependent diabetes mellitus in 42.6%, chronic obstructive lung disease in 20.4%, chronic renal failure in 19.4% with 12% in chronic replacement therapy from total patients, coronary artery disease in 16.7%, with stent in 8.3% and 3.7% with aortic coronary graft from total patients, arterial hypertension in 50%, chronic heart failure in 20.4%, atrial fibrillation in 10.2%,. Death occurs in 29.6% of total patients, septic shock in 25% and the use of mechanical ventilation in 5.6%. We did not find any association between comorbidity and the use of mechanical ventilation, but association with in-hospital mortality was found in pre-existent coronary artery disease (p = 0.001) and in patients with coronary artery stent (p = 0.011). Odds ratio (OR) was respectively 5.2 (95% IC = [1.8–15.0]) and 5.6 (95% IC = [1.3–24.1]). We found significant association between chronic heart failure and shock (p = 0.013) with OR = 3.4 (95% IC = [1.3–9.1]).

**Discussion:** The small size of our sample may enlimit the contibution of other comorbidities on outcomes in sepsis such chronic renal failure, especially with renal replacement therapy and diabetes mellitus. Whereas, we can conclude that cardiac diseases have the most important impact on outcomes in sepsis.

**Conclusion:** Outcomes in sepsis can be affected by comorbidities, especially cardiac diseases. Therefore, that needs large studies to check it.

**Compliance with ethics regulations:** Yes.

### P-151 Micafungin population PK analysis in critically ill patients receiving continuous veno-venous hemofiltration or continuous veno-venous hemodiafiltration

Nicolas Garbez^1^, Litaty Mbatchi^1^, Steven C. Wallis^2^, Laurent Muller^1^, Jeffrey Lipman^2^, Jason A. Roberts^2^, Jean-Yves Lefrant^1^, Claire Roger^1^

#### ^1^CHU Nîmes, Nîmes, France; ^2^University of Queensland, Brisbane, Australia

##### **Correspondence:** Nicolas Garbez (nicolas.garbez@umontpellier.fr)

###### *Ann. Intensive Care* 2020, **10(Suppl 1):**P-151

**Rationale:** To compare the population pharmacokinetics (PK) of micafungin in critically ill patients receiving continuous veno-venous hemofiltration (CVVH, 30 mL/kg/h) to those receiving equidoses of hemodiafiltration (CVVHDF, 15 mL/kg/h + 15 mL/kg/h).

**Patients and methods:** Critically ill patients in septic shock undergoing continuous renal replacement therapy (CRRT) and receiving 100 mg micafungin once daily were eligible for inclusion. Total micafungin plasma concentrations were analyzed using Pmetrics^®^. Probability of target attainment (PTA) was calculated from Monte Carlo simulations using 24-hour area under curve/minimum inhibitory concentration (AUC0-24/MIC) cut-offs 285 (*C. parapsilosis*), 3000 (all Candida species) and 5000 (C. non parapsilosis). Daily dosing regimens of 100, 150 and 200 mg were simulated for the first 2 days of treatment.

**Results:** Eight patients were included in the study. Micafungin concentrations were best described by a two-compartmental PK model. No covariate, including CRRT modality (CVVH and CVVHDF), was retained in the final model, confirmed by internal validation. The mean parameter estimates (standard deviation) were 0.96 (0.32) L/h for clearance, 14.84 (5.33) L for the volume of the central compartment, 0.36 (0.33) 1/h and 0.53 (0.24) 1/h for rate constants. The standard 100 mg daily dosing was unable to reach 90% of PTA for all Candida species except *C. albicans* on the second day of therapy (Fig. 1).

**Conclusion:** There was no difference in micafungin PK between equidoses of CVVH and CVVHDF. A dose escalation to 200 mg is suggested to achieve the PK/PD target of Candida species with MICs exceeding 0.016 mg/L in this population. These “off-label” dosing regimens should be further investigated in clinical trials knowing the favourable toxicity profile and the post-antifungal effect of micafungin in order to ensure efficacy and to prevent the emergence of resistance due to an inadequate initial antifungal dosing regimen.

**Compliance with ethics regulations:** Yes.

**Fig. 1:**
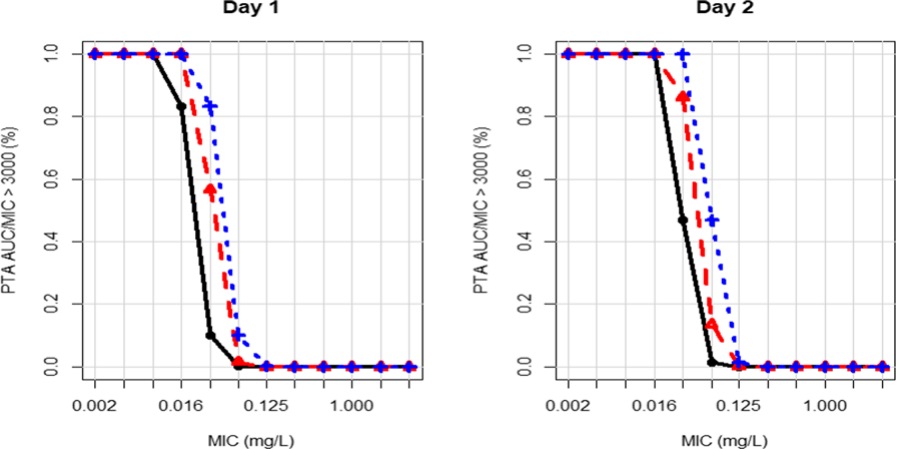
PTA based on AUC0-24/MIC ratios > 3000 for different dosing regimens over 2 days. Black lines with filled circles: 100 mg daily. Red dash lines with triangles: 150 mg daily. Blue dash lines with crosses: 200 mg daily. AUC0-24: Area under curve (0–24 h). MIC: 0.002–4 mg/L

### P-152 Amikacin pharmacokinetic/pharmacodynamic (PK/PD) in intensive care unit: is a 25 mg/kg daily dose really insufficient?

#### Elsa Logre^1^, Maya Enser^1^, Sébastien Tanaka^2^, Marie Dubert^3^, Aurore Claudinon^4^, Nathalie Grall^5^, Hervé Mentec^1^, Philippe Montravers^6^, Olivier Pajot^1^

##### ^1^Réanimation polyvalente, CH Argenteuil, Argenteuil, France; ^2^Réanimation chirurgicale, CHU Bichat et Université de la Réunion, INSERM UMR1188 Diabète, Saint-Denis de la Réunion, Paris, France; ^3^Maladies infectieuses et tropicales, CHU Bichat, Paris, France; ^4^Microbiologie, CH Argenteuil, Argenteuil, France; ^5^Microbiologie, CHU Bichat, Paris, France; ^6^Réanimation chirurgicale, CHU Bichat, Paris, France

###### **Correspondence:** Elsa Logre (elsa.logre@gmail.com)

*Ann. Intensive Care* 2020, **10(Suppl 1):**P-152

**Rationale:** Therapeutic effect of aminoglycosides is obtained when peak plasma concentration (Cmax) reach 8 to tenfold the minimal inhibitory concentration (MIC). For amikacin (AMK), Cmax should thus reach 64 to 80 mg/L, based on clinical breakpoints (defined by EUCAST). Hence, this study aimed to assess the proportion of patients achieving the PK/PD target, Cmax/MIC ≥ 8, using the measured MICs in critically ill patients treated for documented Gram-negative bacilli (GNB) infections. We also evaluated the impact of PK/PD parameters on clinical outcome.

**Patients and methods:** Retrospective study conducted between February 2016 and December 2017 in 2 intensive care units (ICU). All patients receiving a single daily dose of 25 mg/kg AMK for documented severe GNB infections, with MIC and Cmax measurements available on first day of treatment (D1) were included. Results are expressed in n (%) or median [min-max]. Clinical outcome was measured with SOFA score at D8 and mortality.

**Results:** 93 patients, median age 62 years [24–90], accounting for 98 GNB-documented infections were included. SAPS II at admission and SOFA score at D1 were 54 [15–124] and 7 [0–17], respectively. 58 (59.1%) episodes required vasopressors and 62 (63.3%) mechanical ventilation. The leading sources of infection were pulmonary (53%), abdominal (24.5%) and urinary (15.3%) and main causative GNBs were E. coli (31.1%), *P. aeruginosa* (29.5%), *K. pneumoniae* (8.3%). Median dose of AMK was 25 mg/kg [15.6–31.8]. The median Cmax was 55.2 mg/L [12.2–165.7] and the median MIC was 2 mg/L [0.19–16]. The Cmax/MIC ratio ≥8 target was achieved in 87 cases of infections (88.8%) while a Cmax ≥ 64 mg/L was achieved in only 38 cases (38.7%). Overall probability of PK/PD target attainment was 93% considering our MICs and Cmax distributions (Fig. 1). In multivariate analysis, serum creatinine at D1 was the only variable associated with Cmax ≥ 64 mg/L (OR = 1.01 [1.00–1.01]; p = 0.004). Neither Cmax/MIC ratio nor Cmax at D1 were associated with clinical outcome.

**Conclusion:** Studies based on clinical breakpoints may overstate the risk of pharmacodynamic failure. A single daily dose of 25 mg/kg AMK seems appropriate in most critically ill patients treated for severe GNB infections, considering Cmax and MICs observed in our study.

**Compliance with ethics regulations:** Yes.

**Fig. 1:**
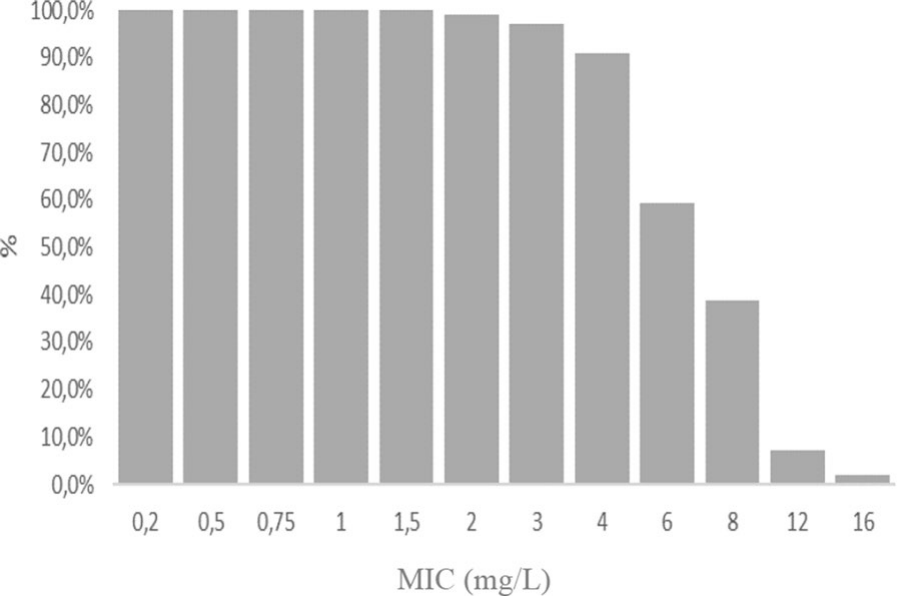
Probability of PK/PD target attainment (Cmax/MIC ≥ 8), according to the MIC

### P-153 Performance of qSOFA for identifying patients with suspected infection at risk of adverse outcomes during a review by an intensivist

#### Maïté Agbakou^1^, Seydou Goro^2^, Axelle Dupont^2^, Jean-Baptiste Lascarrou^1^, Charlotte Garret^1^, Amelie Seguin^1^, Maelle Martin^1^, Arnaud-Felix Miailhe^1^, Helene Migueres^1^, Olivier Zambon^1^, Laura Crosby^1^, Jean Reignier^1^, Jerome Lambert^2^, Emmanuel Canet^1^

##### ^1^Service de médecine intensive réanimation, CHU Nantes, Nantes, France; ^2^Service de biostatistique et information Médicale, Hôpital Saint Louis, APHP, Paris, France

###### **Correspondence:** Maïté Agbakou (maite.agbakou@gmail.com)

*Ann. Intensive Care* 2020, **10(Suppl 1):**P-153

**Rationale:** Sepsis is an important cause of morbidity and mortality in hospitalized patients. Recognizing and responding to patients who experience clinical deterioration remains challenging in daily practice. Our purpose was to assess the ability of the Quick Sequential Organ Failure Assessment (qSOFA) score to identify, among patients reviewed by an intensivist, those at risk of adverse outcomes.

**Patients and methods:** Retrospective cohort of patients with suspected infection reviewed by an intensivist in a university-affiliated hospital between January 2018 and June 2018. Outcomes of interest were hospital mortality and a combined criterion of hospital mortality or ICU stay of 3 days or more.

**Results:** During the study period, 1163 patients were reviewed by an intensivist, of whom 459 (39.6%) had suspected infection according to the Sepsis-3 criteria. At the time of review, 192 (41.8%) patients with suspected infection were qSOFA positive (≥2) and 267 (58.2%) were qSOFA negative (0–1). Following the review, 240 (52.5%) patients were admitted to the ICU, among whom 202 (79.5%) had a prolonged stay (≥3 days). In-hospital mortality was 27.9%, and 79.9% of the patients met the combined criterion of in-hospital mortality or prolonged ICU stay. qSOFA positive patients required more frequently mechanical ventilation (52.9% vs. 36.6%, p = 0.01) and vasopressor support (50.5% vs. 28.8%, p < 0.001) than qSOFA negative patients. Moreover, qSOFA positive patients had higher hospital mortality than qSOFA negative patients (34.5% vs. 23.2%, p = 0.02). For the prediction of in-hospital mortality, a positive qSOFA had a predictive positive value (PPV) of 34%, and a negative predictive value (NPV) of 77%. For the prediction of in-hospital mortality or prolonged ICU stay, a positive qSOFA had a PPV of 83% and a NPV of 24%.

**Conclusion:** Hospitalized patients with suspected infection for whom a review by an intensivist was requested, are at high risk of hospital mortality. Although the accuracy of qSOFA for identifying patients at risk of adverse outcomes is limited, its integration in a multimodal risk assessment approach may help distinguish the subset of patients who will benefit from an escalation of care.

**Compliance with ethics regulations:** Yes.

### P-154 Accuracy of SOFA, qSOFA, SIRS criteria and lactate level in predicting mortality of infected ICU patients

#### Ahlem Trifi^1^, Cyrine Abdennebi^2^, Foued Daly^1^, Yosr Touil^1^, Sami Abdellatif^1^, Salah Ben Lakhal^1^

##### ^1^Medical ICU, la Rabta hospital, Faculty of Medicine of Tunis, Tunis, Tunisia; ^2^Medical ICU la Rabta hospital, Tunis, Tunisia

###### **Correspondence:** Ahlem Trifi (trifiahlem2@gmail.com)

*Ann. Intensive Care* 2020, **10(Suppl 1):**P-154

**Rationale:** According to the Sepsis-3 consensus, sepsis is identified as an increase of at least 2 points in the Sepsis-related Organ Failure Assessment (SOFA) score in patients who presented infection. The quick SOFA or qSOFA is considered as a predictive tool of sepsis and mortality when it is equal to 2 points or more. Systemic Inflammatory Response Syndrome (SIRS) criteria are of limited utility because of their low sensitivity. Hyperlactatemia, as known is a determinant of tissue hypoperfusion. Our objective was to evaluate the prognostic value of SOFA > 2, SIRS > 2, qSOFA > 2 and lactate level > 2 mmol/l in infected patients.

**Patients and methods:** Nine-month prospective cohort study. Patients aged 18 years or older who had a proven or suspected infection were included. SOFA score, SRIS criteria, SOFA q and lactate levels were determined within the first 24 h of infection. The primary endpoint was hospital mortality at 30 days. The predictive power of the studied parameters was determined using using the area under the receiver operating characteristic curve (AUROC).

**Results:** A cohort of 71 cases was studied with mean age at 49.5 years. Bacterial pneumonia was the most common infection site (66%). In the first 24 h of onset of infection the medians [IQR 25–75] of the SOFA, SRIS, and SOFA scores and lactate levels were respectively 6 [3–9], 3 [2–3], 1 [1–2] and 2.04 [0.65–3.4]. The progression to severe septic status was observed in 34 patients (48%) and norepinephrine was introduced in 32 cases. Median length of stay was 11 days [5–18] and mortality was 53%. Overall, the accuracy in predicting mortality of the 4 studied parameters was poor. An increase of SOFA score by at least 2 points had greater accuracy with AUROC = 0.762 [0.647–0.877], sensitivity = 74% and specificity = 79%.

**Conclusion:** In infected patients, the SOFA score had greater prognostic accuracy than the SIRS criteria, the qSOFA score or the lactate level. These results suggest that SIRS, qSOFA, and high lactate level may be useful in screening for sepsis, but this utility is limited in predicting mortality.

**Compliance with ethics regulations:** Yes.

**Fig. 1:**
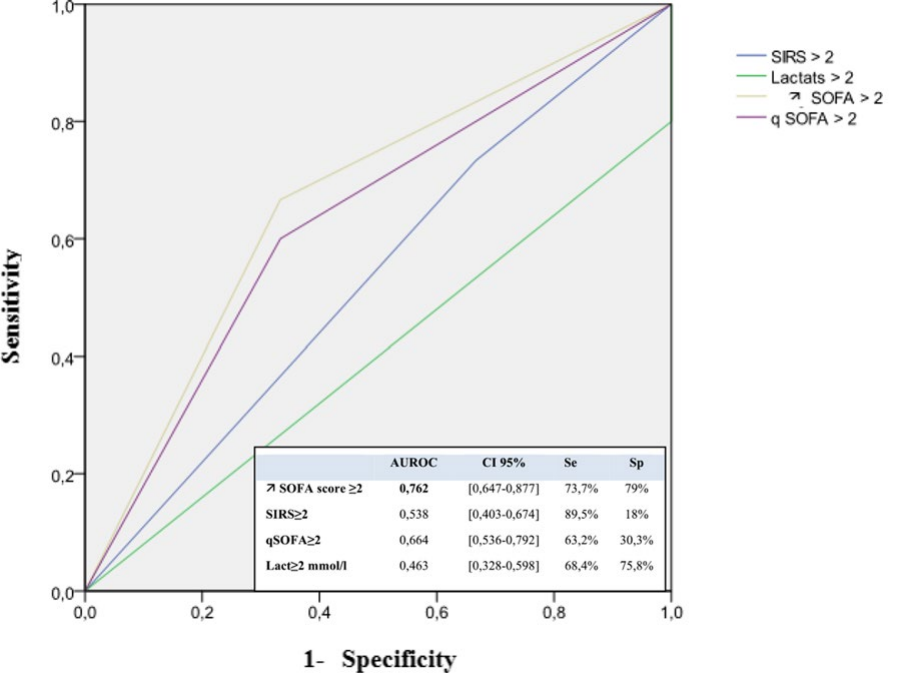
ROC curves of SOFA, qSOFA, SIRS, lactate level to predict mortality. *SOFA* Sequential Organ Failure Assessment, *qSOFA* quick SOFA, *SIRS* systemic inflammatory response syndrome, *AUROC* area under the ROC curve, *Se* sensitivity, *Sp* specificity

### P-155 Use of qSOFA score for predicting prognosis in patients admitted with decompensated liver cirrhosis

#### Khaoula Ben Ismail, Sana Khedher, Ameni Khaled, Nassereddine Foudhaili, Mohamed Salem

##### USI Digestif-service de gastroenterologie-EPS Charles Nicolles.Tunis –Tunisia, Tunisia, Tunisia

###### **Correspondence:** Khaoula Ben Ismail (khaoula87@hotmail.fr)

*Ann. Intensive Care* 2020, **10(Suppl 1):**P-155

**Rationale:** Quick sequential organ failure assessement (qSOFA) has been validated for patients with presumed sepsis and others in general emergency department (ED) population. However, it has not been validated in specific subgroups of patients with a high mortality. The aim of this study is to evaluate the ability of qscore to predict prognosis in patients with decompensated liver cirrhosis.

**Patients and methods:** This is a retrospective study, conducted over a period of 3 years from January 2016 to December 2018. Consecutive patients with decompensated cirrhosis, admitted in our department are included. Data of all patients were collected and the qSOFA score was calculated at admission. The main study endpoints were length of stay, complications and in-hospital mortality.

**Results:** A total of 110 patients diagnosed with decompensated cirrhosis were enrolled. Mean of age was 62 years (18–88). Sex ratio was 1.2. HCV (39%) was the main etiology of cirrhosis. The reasons of hospitalization were: oedema with ascitic syndrome in 44% of cases, digestive haemorrhage (21% of cases), fevers (16% of cases), and hepatic encephalopathy was present in 36% of cases. The mean duration of stay was 10 days ± 7. In-hospital mortality rate was 20% and mean score qSOFA was 1.2.The qSOFA score was significantly correlated with length of stay (p = 0.02) and complications(p = 0.04) but not with in-hospital mortality (p = 0.99).

**Conclusion:** The qSOFA score was not useful for predicting in hospital mortality in patients with decompensated liver cirrhosis but it was significantly correlated to the length of stay and complications.

**Compliance with ethics regulations:** Yes.

### P-156 Angioedema associated with thrombolysis for ischemic stroke: analysis of a case-control study

#### Clara Vigneron^1^, Aldéric Lécluse^2^, Thomas Ronzière^3^, Sonia Alamowitch^4^, Olivier Fain^1^, Nicolas Javaud^5^

##### ^1^Médecine Interne, Centre de Référence associé sur les angiœdèmes à kinines (CRéAk), Hôpital Saint-Antoine, APHP, Paris, France; ^2^Neurologie, CHU Angers, Angers, France; ^3^Neurologie, CHU Pontchaillou, Rennes, France; ^4^Neurologie, Hôpital Saint-Antoine, APHP, Paris, France; ^5^Urgences, Centre de Référence associé sur les angiœdèmes à kinines (CRéAk), Hôpital Louis Mourier, APHP, Colombes, France

###### **Correspondence:** Clara Vigneron (claravigneron@hotmail.fr)

*Ann. Intensive Care* 2020, **10(Suppl 1):**P-156

**Rationale:** Bradykinin-mediated angioedema is a complication associated with thrombolysis for acute ischemic stroke. Risk factors are unknow and management is discussed. The aim of this study was to clarify risk factors associated with bradykinin-mediated angioedema after thrombolysis for acute ischemic stroke.

**Patients and methods:** In a case-control study conducted at a French reference center for bradykinin angiœdema, patients with thrombolysis for acute ischemic stroke and a diagnosis of bradykinin-mediated angiœdema, were compared to controls treated with thrombolysis treatment without angiœdema. Two matched control subjects were analyzed for each case.

**Results:** 53 thrombolysis-related angioedema were matched to 106 control subjects. The sites of attacks following thrombolysis for ischemic stroke mainly included tongue (34/53, 64%) and lips (26/53, 49%). The upper airways were involved in 37 (70%) cases. Three patients required mechanical ventilation. Patients with bradykinin-mediated angiœdema were more frequently women (33 (62%) vs. 44 (42%); p = 0.01), had higher frequency of prior ischemic stroke (12 (23%) vs 9 (8%); p = 0.01), hypertension (46 (87%) vs. 70 (66%); p = 0.005), were more frequently treated with angiotensin-converting enzyme inhibitor (37 (70%) vs. 28 (26%); p < 0.001) and were more frequently hospitalized in intensive care unit (11 (21%) vs. 5 (5%); p = 0.004). In multivariate analysis, factors associated with thrombolysis-related angioedema were female sex (odds ratio [OR], 3.04; 95% confident interval [CI], 1.32–7.01; p = 0.009) and treatment with angiotensin-converting enzyme inhibitors ([OR], 6.08; 95% [CI], 2.17–17.07; p < 0.001).

**Discussion:** Because of the retrospective case-control design and the lack of the total number of thrombolysis for ischemic stroke, the incidence of this complication could not be evaluated in our study. Previous studies reported an incidence of 2.1 to 7.9% of angioedema in patients treated with a thrombolytic therapy for acute ischemic stroke. Our case-control study permits for the first time to analyse more cases to evaluate associated risk factors of this rare complication.

**Conclusion:** This case-control study points out angiotensin-converting enzyme inhibitors and female sex as risk factors of bradykinin-angioedema associated with thrombolysis for ischemic stroke.

**Compliance with ethics regulations:** Yes.

### P-157 Outcome of patients with inflammatory bowel diseases admitted to the intensive care unit: a multicenter retrospective study of 76 patients

#### Romaric Larcher, Fanny Garnier, Matthieu Amalric, Vincent Brunot, Laura Platon, Yassir Aarab, Guillaume Pineton De Chambrun, Jonathan Charbit, Samir Jaber, Boris Jung, Kada Klouche

##### Médecine intensive–Réanimation, Hôpital Lapeyronie, CHU de Montpellier, Montpellier, France

###### **Correspondence:** Romaric Larcher (r-larcher@chu-montpellier.fr)

*Ann. Intensive Care* 2020, **10(Suppl 1):**P-157

**Rationale:** Patients with inflammatory bowel disease (IBD), frequently treated by immunosuppressive drugs, are more susceptible to be admitted to the intensive care unit (ICU). However, outcome and predictive factors of mortality are little known. Therefore, we aimed to assess the outcome and prognostic factors for critically ill IBD patients.

**Patients and methods:** We retrospectively studied data of consecutive IBD (i.e. Crohn’s disease and ulcerative colitis) patients admitted in 3 ICUs between 2007 and 2017. In-ICU and one-year mortalities were estimated and predictive factors of in-ICU mortality were identified by univariate and multivariate analysis.

**Results:** Seventy-six patients (male: 62%, median age: 52.7 [42.1–66.5] years, Charlson index: 2 [3.0-5.0]) entered the study. IBD type was largely represented by Crohn’s disease (67.1%) and its localization was mostly extensive: L3 (58.8% of Crohn’s disease) or E3 (92% of ulcerative colitis) according to the Montreal classification. Twenty-seven patients (35.5%) were treated with corticosteroids and 37 (50%) with immunosuppressive therapy (azathioprine: 26.3% and anti-TNFα: 20%). Reasons for admission were shock/sepsis (61.8%) and acute respiratory failure (19.7%). ICU diagnoses were infection (75%), IBD flare-up (48.7%) or both (32.9%), and pulmonary embolism (5.3%). At admission, SOFA score was 5 [2.0–8.0] and SAPS II 33.0 [24.7–45.2]. Fifty-three patients (56.6%) required mechanical ventilation, 29 (38.2%) vasoactive drugs, and 8 (10.5%) renal replacement therapy. Twenty-three patients underwent emergency surgery (30.2%) and six urgent endoscopic treatment (7.9%). In-ICU and one-year mortality rate were 7.9% and 18.4%, respectively. Prognostic factors of in-ICU mortality were SOFA score (HR 1.31, 95% CI [1.17–1.47], p < 0.01) and azathioprine treatment before ICU admission (HR 4.13, 95% CI [1.56–10.90], p < 0.01) (Fig. 1). Previous immunosuppressive treatment with anti-TNF did not alter the prognosis and even the type of IBD.

**Conclusion:** Our study showed that more than 90% of IBD critically ill patients were discharged alive from the ICU and a majority of them survived after one-year (81.6%). We also found that SOFA score and previous azathioprine immunosuppressive treatment worsened ICU outcome. Higher severity of the acute event affected short-term prognosis and should be taken into account for best ICU triage and management. Intensivists should pay particular attention to patients treated by azathioprine.

**Compliance with ethics regulations:** Yes.

**Fig. 1:**
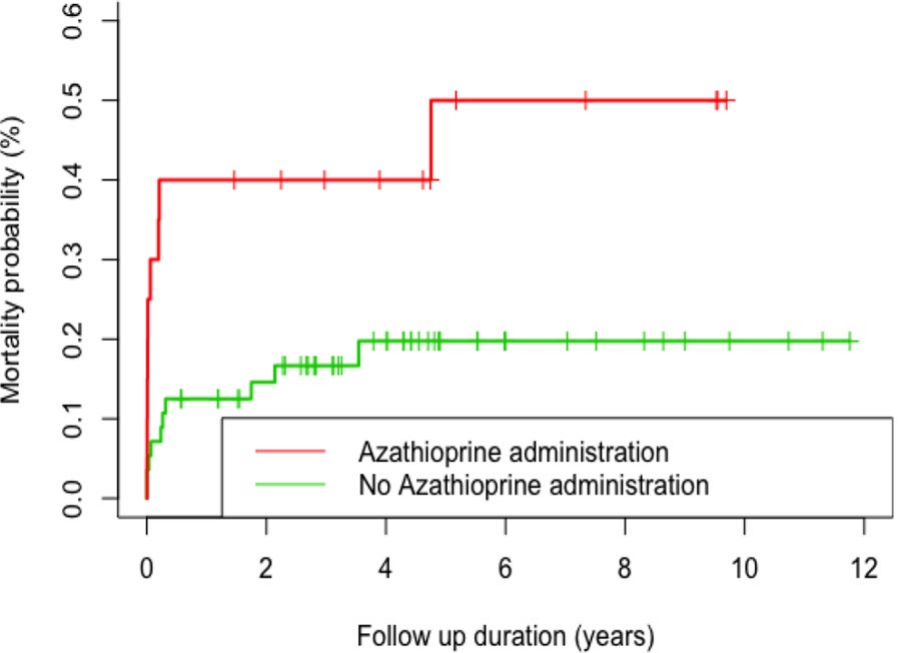
Outcome of IBD patients admitted to the ICU according to precious treatment with azathioprine status

### P-158 Carboxyhemoglobin level is a reliable diagnosis biomarker of hemolytic anemia in intensive care unit

#### Geoffroy Hariri, Kyann Hodjat-Panah, Laurene Blum, Jean-Rémi Lavillegrand, Idriss Razach, Naike Bige, Jean-Luc Baudel, Bertrand Guidet, Eric Maury, Hafid Ait-Oufella

##### Médecine Intensive-Réanimation, Hôpital Saint-Antoine, Paris, France

###### **Correspondence:** Geoffroy Hariri (geoffroyhariri@hotmail.com)

*Ann. Intensive Care* 2020, **10(Suppl 1):**P-158

**Rationale:** Hemolytic anemia (HA) is a common condition in intensive care unit but its diagnosis remains challenging. Free hemoglobin (and heme) degradation leads to CO release that can bind to hemoglobin to form carboxyhemoglobin (HbCO). We hypothesized that HbCO concentration could be used as a reliable diagnosis tool for HA.

**Patients and methods:** We performed a monocentric retrospective study in a 18-bed intensive care unit at St Antoine Hospital, Paris, between 2012 and 2018. All patients hospitalized for HA with arterial HbCO dosage at admission were included. Arterial HbCO was measured in routine in our department with an IL system 1303 pH/blood gas analyzer. Demographic and biological data were collected. A group control of patients with non-hemolytic anemia (Hb < 9 g/dl) (NHA) was also included. Finally, we analyzed patients outcome according to HbCO changes during ICU stay.

**Results:** Between 2012 and 2018, 98 patients with HA were included. 100 NHA patients were included in the control group. Patients with HA were younger than patients with NHA (47 [32; 63] vs. 69 [59; 78] years old, P = 0.001) but admission SOFA was not different between groups (6 [3; 7], vs. 6 [4; 9], P = NS). Among patients with HA, 55% had thrombotic microangiopathy, 25% had autoimmune hemolytic anemia and 20% had sickle cell disease. At ICU admission, HA patients had higher HbCO level than patients with NHA (3.31 [2.3; 3.9] vs. 1.34 [0.9; 1.7] %; p < 0.0001). HbCO was a reliable biomarker of hemolysis (AUC 0.92 (0.88; 0.96) p < 0.0001). An HbCO level threshold at 2.05% identify hemolysis with a sensitivity 87 (79–92) % and a specificity 88 (82–93) %. In HA group, HbCO was negatively correlated to Hb level (R = 0.41; p < 0.0001). In HA patients, changes of HbCO level during ICU management were associated with outcome, decreasing in survivors (2.7 [2; 3.4] vs. 3.1 [2.2; 4.2]; p = 0.018) but not in non-survivors (2.8 [1.9; 3.3] vs. 2.6 [1.3; 3.5] %; p = 0.57).

**Conclusion:** Carboxyhemoglobin is a reliable diagnosis and prognosis biomarker for hemolytic anemia in ICU

**Compliance with ethics regulations:** Yes.

### P-159 Thrombocytopenia in intensive care: still not easy

#### Amine Raja, Mohamed Elaiassi, Maghfour Abdejabbar, Boubaker Charra

##### ^1^The medical resuscitation department (27) of Ibn Rochd University Hospital in Casablanca, Casablanca, MOROCCO

###### **Correspondence:** Amine Raja (raja.amine2@gmail.com)

*Ann. Intensive Care* 2020, **10(Suppl 1):**P-159

**Rationale:** Thrombocytopenia is the most commonly hemostatic disorder encountered in intensive care, present in 41 to 66% of patients. The mortality associated with this thrombocytopenia, the numerous pathological contexts associated with resuscitation and the lack of a recommended management strategy led to the establishment of these guidelines. The aim of our study was to determine the incidence, causes and risk factors associated with the occurrence of thrombocytopenia, as well as the impact of thrombocytopenia on the mortality and length of stay in the ICU Ibn Medical Resuscitation Unit. Rochd de Casablanca, over a period of 12 months.

**Patients and methods:** This was a prospective study, carried out in the medical resuscitation department of Ibn Rochd University Hospital in Casablanca over a period of 12 months. There were two groups: ‘‘sick’’ group with thrombocytopenia with a platelets count < 150,000/mm^3^, and a ‘‘control’’ group without thrombocytopenia. Patients with previous platelet disorders, hematologic malignancies, and patients undergoing chemotherapy were excluded.

**Results:** Of the 317 patients included, 107 episodes of thrombocytopenia were identified, an overall incidence of 33.75%. Sepsis was incriminated 65 times (60.7%), followed by ARDS in 12 patients (11.2%), massive filling in 10 patients (9.3%), disseminated intravascular coagulation in 9 patients (8.4%), and massive transfusion in 6 patients (5.6%). The drug origin was incriminated in 2 patients (1.8%). It was due to quinolones and imipenem. The mortality rate was 54 deaths (50.4%) which was inversely proportional to the lowest platelet count in the thrombocytopenia group, compared to 61 deaths (30%) in the control group. The mean duration of stay in the thrombocytopenia group was 11 ± 10 days with extremes ranging from 3 to 124 days.

**Conclusion:** Thrombocytopenia was a common abnormality in the intensive care system, it occured in many pathological situations and was a factor of morbidity and excess mortality. The most common etiology in this study was sepsis. The diagnostic and therapeutic approach depended on the particular clinical context in which thrombocytopenia occurs. Its onset may constitute a hematological emergency, particularly when there is a major mucocutaneous and / or visceral hemorrhagic syndrome, which necessitates a rapid etiological diagnosis, and the establishment of an effective treatment, both symptomatic and specific.

**Compliance with ethics regulations:** Not applicable.

### P-160 CAPS criteria fail to identify most severely-ill thrombotic antiphospholipid syndrome patients requiring intensive care unit admission

#### Marc Pineton De Chambrun^1^, Romaric Larcher^2^, Frédéric Pene^3^, Laurent Argaud^4^, Alexandre Demoule^5^, Rémi Coudroy^6^, Elie Azoulay^7^, Yacine Tandjaoui-Lambiotte^8^, Stanislas Faguer^9^, Alain Combes^1^, Charles-Edouard Luyt^1^, Zahir Amoura^10^

##### ^1^Sorbonne Université, APHP, Hôpital La Pitié–Salpêtrière, Institut de Cardiométabolisme et Nutrition (ICAN), Service de Médecine Intensive-Réanimation, Paris, Paris, France; ^2^Service de Médecine Intensive-Réanimation, Hôpital Lapeyronie, Centre Hospitalier Universitaire (CHU) de Montpellier PhyMedExp, Université de Montpellier, INSERM, CNRS, Montpellier, France; ^3^Service de Médecine Intensive-Réanimation, Hôpital Cochin, Hôpitaux Universitaires Paris Centre, APHP & Université Paris Descartes, Paris, France; ^4^Service de Médecine Intensive-Réanimation, Hôpital Edouard Herriot, Hospices Civils de Lyon, Lyon, France; ^5^APHP, Hôpital La Pitié–Salpêtrière, Service de Pneumologie, Médecine Intensive et Réanimation Médicale, Département R3S Sorbonne Université, INSERM UMRS1158, Neurophysiologie Respiratoire Expérimentale et Clinique, Paris, France; ^6^Service de Médecine Intensive-Réanimation, INSERM CIC1402, groupe ALIVE, Université de Poitiers, CHU de Poitiers, Poitiers, France; ^7^Service de Médecine Intensive-Réanimation, Hôpital Saint-Louis, APHP, Paris, France; ^8^Service de Réanimation Médico-Chirurgicale, Hôpital Avicenne, APHP, HUPSSD, Bobigny, France; ^9^Département de Néphrologie et Transplantation d’organes-Unité de Réanimation, Centre de Référence des Maladies Rénales Rares, Hôpital Rangueil, CHU de Toulouse, Toulouse, France; ^10^Sorbonne Université, Assistance Publique-Hôpitaux de Paris (APHP), Hôpital La Pitié–Salpêtrière, Institut E3M, Service de Médecine Interne 2, Centre de Référence National Lupus Systémique, Syndrome des Anticorps Anti-phospholipides et autres maladies auto-immunes systémiques rares, Parisf, France

###### **Correspondence:** Marc Pineton De Chambrun (marc.dechambrun@gmail.com)

*Ann. Intensive Care* 2020, **10(Suppl 1):**P-160

**Rationale:** Catastrophic antiphospholipid syndrome (CAPS), the most severe manifestation of antiphospholipid syndrome (APS), is characterised by simultaneous thromboses in multiple organs. Diagnosing CAPS can be challenging but its early recognition and management is crucial for a favourable outcome. This study was undertaken to evaluate the frequencies, distributions and ability to predict mortality of “definite/probable” or “no-CAPS” categories of thrombotic APS patients requiring admission to the intensive care unit (ICU).

**Patients and methods:** This French national multicentre retrospective study, conducted from January 2000 to September 2018, included all APS patients with any new thrombotic manifestation(s) admitted to 24 ICUs.

**Results:** One hundred and thirty-four patients (male/female ratio: 0.4; mean age at admission: 45.4 ± 15.0 years), who experienced 152 CAPS episodes, required ICU admission. The numbers of definite, probable or no-CAPS episodes (Fig. 1), respectively, were: 11 (7.2%), 60 (39.5%) and 81 (53.5%). No histopathological proof of microvascular thrombosis was the most frequent reason for not being classified as definite CAPS. Overall, 35/152 (23.0%) episodes were fatal, with comparable rates for definite/probable CAPS and no CAPS (23% vs. 28.8% respectively, p = 0.4). The Kaplan–Meier curve of estimated probability of survival showed no between-group survival difference (log-rank test p = 0.5).

**Discussion:** Our results suggest that the CAPS criteria do not sufficiently encompass all the parameters responsible for thrombotic APS patients’ disease severity in the ICU. The absence of items referring to organ dysfunction/failure in the CAPS criteria probably limited their ability to predict mortality. Albeit useful for the retrospective classification and comparison of patients, the CAPS criteria may be too stringent and not yet ready-to-use for the management of ICU patients. For physicians outside expert APS centres, the absence of CAPS criteria could be misleading and lead to rejection of the diagnosis for near-CAPS patients, thereby preventing them from receiving the appropriate aggressive treatment they indeed require. We think that, when confronted with a critically-ill thrombotic APS patient, CAPS criteria should be interpreted with caution and should not be the only elements taken into account to decide the intensity of the therapeutic management.

**Conclusion:** In this study, CAPS criteria were not associated with mortality of thrombotic APS patients requiring ICU admission. Further studies are needed to evaluate the adequacy of CAPS criteria for critically-ill APS patients.

**Compliance with ethics regulations:** Yes.

**Fig. 1:**
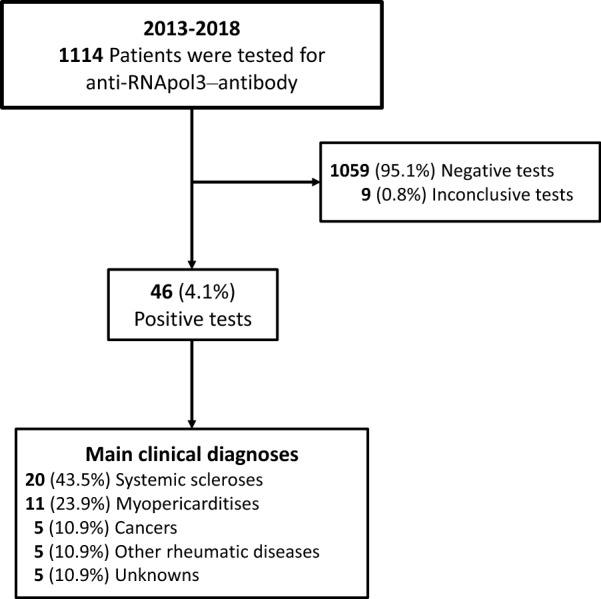
Distributions of the 152 episodes of definite/probable and no catastrophic antiphospholipid syndrome (CAPS) requiring intensive care unit (ICU) admission, diagnosed in 134 antiphospholipid syndrome patients, and their attributed fatalities, with respective reasons for not being classified as definite CAPS

### P-161 Anemia in intensive care unit

#### Sabah Benhamza, Afak Nsiri, Rachid Al Harrar

##### Surgical emergency resuscitation service of Ibn Rochd University Hospital, Casablanca, Morocco

###### **Correspondence:** Sabah Benhamza (benhamzasabah5@gmail.com)

*Ann. Intensive Care* 2020, **10(Suppl 1):**P-161

**Rationale:** 75% of resuscitation patients develop anemia during their stay, it can worsen the prognosis, prolong the length of stay and lead to transfusions that can be the cause of complications. The objective of our work is to specify the incidence of anemia in our unit, its etiologies and its therapeutic management.

**Patients and methods:** We conducted a descriptive and analytical retrospective study within the surgical emergency resuscitation department of Ibn Rochd University Hospital of Casablanca, over a period of 4 years from 2015 to 2018. We included all anemic patients. Statistical analysis was performed with SPSS statistics 20. p < 0.05 was considered significant.

**Results:** We included 361 patients with an estimated incidence of 27%, the average age was 39 years, the sex ratio H / F was 1.93. 47% of admissions were for traumatic pathology and 30% postoperative digestive surgery. 13% had hypotension at admission and the mean temperature was 38.2% .The onset of anemia and its depth were related to length of stay with 99.18% of patients who were anemic beyond the 12th day of hospitalization with a hemoglobin level that became < 8.5 g / dl beyond the 40th day. 60% of the patients had a normochromic normocytic anemia becoming microcytic with the lengthening of the duration of stay. Ferritinemia dosed in 1% of patients and was normal. 48% of our patients had exclusive parenteral nutrition while 26% had an enteral / parenteral combination. 91% were transfused in red blood cells (RBC) and 45% of patients were transfused more than once. 32% received between 3 and 4 RBC units. In 11 patients who received 26 transfusion episodes costing 875 euros, the transfusion was inappropriate. The total cost of the transfusion was estimated at around 33,000 euros. 30% were supplemented with oral iron with an increase in hemoglobin in 6% of them. 52% of the patients came out of the intensive care unit with a hemoglobin level < 10 g/dl/L. The mortality rate of our patients was 14% with as predictive factors in multivariate analysis, hyperthermia, coagulopathy, the transfusion appears as a factor of good prognosis.

**Conclusion:** The prevention of blood spoliation and the fight against inflammation and nosocomial infection remain the pillars of the management of anemia in intensive care but in view of our results and the protective role of transfusion it would be interesting to see again the transfusion thresholds in our context.

**Compliance with ethics regulations:** Yes.

### P-162 Severe viral myopericarditis associated with anti-RNA polymerase III autoantibodies: a new entity

#### Marc Pineton De Chambrun^1^, Jean-Luc Charuel^2^, Guillaume Hekimian^1^, François Lifermann^3^, Isabelle Melki^4^, Charles-Edouard Luyt^1^, Alain Combes^1^, Zahir Amoura^5^

##### ^1^Sorbonne Université, APHP, Hôpital La Pitié–Salpêtrière, Institut de Cardiométabolisme et Nutrition (ICAN), Service de Médecine Intensive-Réanimation, Paris, France; ^2^Sorbonne Université, APHP, Hôpital La Pitié–Salpêtrière, Laboratoire d’Immunochimie, Département d’Immunologie, Paris, France; ^3^Centre Hospitalier de Dax, Service de Médecine Interne, Dax, France; ^4^Université Paris Diderot, APHP, Hôpital Robert-Debré, Service de Pédiatrie Générale, Paris, France; ^5^Sorbonne Université, Assistance Publique–Hôpitaux de Paris (APHP), Hôpital La Pitié–Salpêtrière, Institut E3M, Service de Médecine Interne 2, Centre de Référence National Lupus Systémique et SAPL et autres maladies auto-immunes, Paris, France

###### **Correspondence:** Marc Pineton De Chambrun (marc.dechambrun@gmail.com)

*Ann. Intensive Care* 2020, **10(Suppl 1):**P-162

**Rationale:** Anti-RNA polymerase III (RNApol3) antibodies are rare autoantibodies associated with systemic sclerosis (SSc). Acute myopericarditis, characterized by pericardial and myocardial inflammatory cell infiltrations, and various degrees of myocardial necrosis, is mainly caused by viruses. We identified certain forms of severe viral myopericarditis in the presence of anti-RNApol3 autoantibodies.

**Patients and methods:** We conducted a monocentric, retrospective, observational study (January 2013 to January 2019) including all acute myopericarditis patients with anti-RNApol3 autoantibodies with comparison to RNApol3 negative myopericarditis.

**Results:** Among the 56 viral myocarditis patients hospitalized during the study period, 11 (16 episodes; 34.2 ± 10.9 years old at first antibody detection; female/male sex ratio = 10) were anti-RNApol3 autoantibody-positive. Influenza virus was detected during at least one episode in seven (63.6%) patients. After median [range] follow-up of 48.4 [1.9–71.7] months, only two patients had definite SSc very mild phenotypes, according to the ACR/EULAR 2013 classification. Anti-RNApol3 autoantibodies were never detected in 20 patients with severe influenza virus-related acute respiratory distress syndrome (ARDS). During the 5-year study period, acute myopericarditis accounted for 23.9% of all RNApol3-antibody–positive assays in our immunology laboratory (Fig. 1).

**Discussion:** We described a series of 11 patients with severe acute viral myopericarditises associated with anti-RNApol3 autoantibodies, an association that has never been reported previously. The fortuitous association of these autoantibodies with acute myopericarditis is highly unlikely. Acute myocarditis is a very rare disease with a reported incidence of 22/100,000 inhabitants. Anti-RNApol3–antibody detection is also very rare: 4.6% positive tests (including the 11 patients in this series) out of 1114 samples during a 5-year period in our Immunology Laboratory. This 63% proportion of patients with proven influenza-virus infections suggest that such severe infections could trigger anti-RNApol3 autoantibody production. However, influenza is a common disease and anti-RNApol3 autoantibodies are very rare. Furthermore, no anti-RNApol3 autoantibodies were detected in the 20 patients with severe influenza-related ARDS. Last, anti-RNApol3 autoantibodies remained detectable several months after the viral infection had been cured.

**Conclusion:** This previously unknown association between severe acute viral myopericarditis and anti-RNApol3 autoantibodies is probably not fortuitous. Anti-RNApol3 antibody detection in acute myopericarditis patients could imply individual susceptibility to severe viral infection. Further studies are needed to investigate the pathophysiological mechanisms involved in this entity and potential specific therapeutic strategies.

**Compliance with ethics regulations:** Yes

**Fig. 1:**
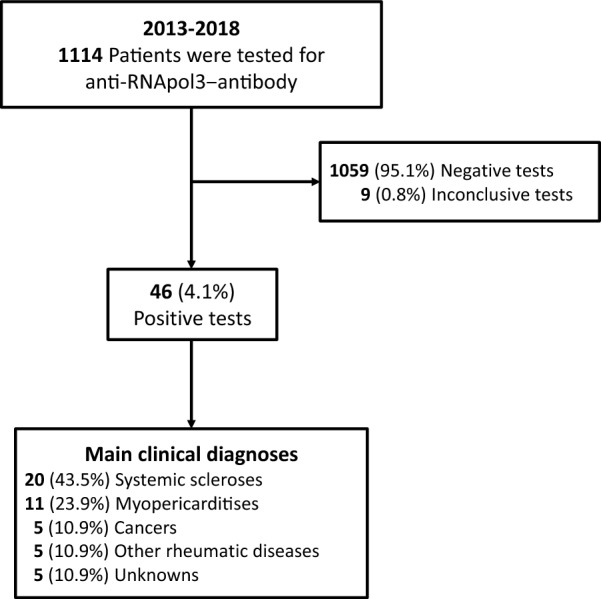
Flow chart of anti-RNA Polymerase III (RNApol3) autoantibody detection in individual patients between 2013 and 2018

### P-163 Gastrointestinal manifestations in severe thrombotic microangiopathies

#### Eric Mariotte^1^, Lara Zafrani^1^, Sandrine Valade^1^, Virginie Lemiale^1^, Adrien Mirouse^1^, Jean Jacques Tudesq^1^, Yannick Hourmant^1^, Agnès Veyradier^2^, Bérangère Joly^2^, Lionel Galicier^1^, Jehane Fadlallah^1^, Michael Darmont^1^, Elie Azoulay^1^

##### ^1^Hôpital Saint Louis, APHP, Paris, France; ^2^Hôpital Lariboisière, APHP, Paris, France

###### **Correspondence:** Eric Mariotte (eric.mariotte@aphp.fr)

*Ann. Intensive Care* 2020, **10(Suppl 1):**P-163

**Rationale:** Thrombotic microangiopathy (TMA) syndrome is characterized by widespread microvascular thrombosis leading to mechanical hemolytic anemia, thrombocytopenia and variable end-organ damage. Digestive tract manifestations during TMA can be due to a direct TMA involvement (like in thrombotic thrombocytopenic purpura (TTP)), or other causes (like in shigatoxin-associated hemolytic uremic syndrome (STEC-HUS)). The goal of this study was to describe the spectrum of gastrointestinal disorders associated with severe TMA and to identify patterns associated with specific TMAs and outcome.

**Patients and methods:** All consecutive patients admitted to a single university hospital ICU with a diagnosis of acute TMA from 2006 to 2018 were included. Demographic, clinical and biological items were abstracted from the patients’ charts. Results are presented as medians (interquartile range) and numbers (%).

**Results:** During the study period, 173 patients presented with acute TMA syndrome, including 107 TTP (61.9%), 28 HUS (16.2% including 15 secondary HUS, 7 STEC-HUS, and 6 atypical HUS) and 41 other TMA (23.7%). They were mostly females (59%) aged 44 (32–56) years old. Gastrointestinal manifestations were observed in 69 (39.9%) patients, consisting of abdominal pain in 35 (20.2%) cases, non-bloody diarrhea in 29 (16.8%), bloody diarrhea in 10 (5.8%), vomiting in 17 (9.8%), liver tests anomalies in 14 (8.1%), pancreas disorders in 7 (4.1%) and bowel obstruction in 3 (1.7%) (Fig. 1). Bloody diarrhea was more frequent in STEC-HUS (57.1% vs. 2.8% of TTP, 6.7% of secondary HUS, 16.7% of atypical HUS and 2.4% of other TMA, p < 0.0001). Bowel obstruction was only seen in 2 HUS and 1 other TMA patients. There was no difference in the relative frequency of other digestive manifestations between the different types of TMA. TMA patients with digestive symptoms presented with more frequent and more severe renal dysfunction (81.2% vs. 59.6%, p = .0016, creatinin 170 [90–396) vs. 102 (576–181) µmol/L, p = 0.0027), reflecting in higher day 1 SOFA scores (7 (6–10) vs. 6 (5–8), p = 0.0031). Gastrointestinal manifestations were associated with lower ICU mortality (2.9% vs. 11.5%, p = 0.0413). Short-term mortality was still lower in TMA patients with digestive symptoms when adjusted on SOFA and types of TMA.

**Conclusion:** Gastrointestinal manifestations are frequent and most of the time aspecific in severe TMA patients. If bloody diarrhea is associated preferentially with STEC-HUS, it may lack in > 40% of the cases and be observed in patients with other types of TMA, including TTP patients. The reason why patients with digestive symptoms seem to display better short-term outcomes deserves further investigations.

**Compliance with ethics regulations:** Yes.

**Fig. 1:**
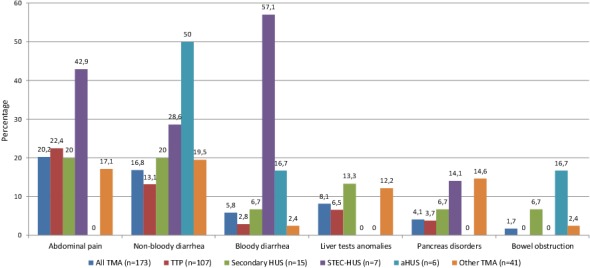
Relative frequencies of digestive manifestations in 173 critically ill TMA patients

### P-164 Study of tolerance and complications of therapeutic plasma exchanges at Rennes University Hospital between 2011 and 2017, the COMPLASMA study

#### Valentin Coirier^1^, Mathieu Lesouhaitier^1^, Florian Reizine^1^, Adel Maamar^1^, Arnaud Gacouin^1^, Yves Le Tulzo^1^, Jean-Marc Tadié^1^, Christophe Camus^1^

##### Rennes University Hospital, Rennes, France

###### **Correspondence:** Valentin Coirier (valentin.coirier@gmail.com)

*Ann. Intensive Care* 2020, **10(Suppl 1):**P-164

**Rationale:** Therapeutic plasma exchanges (TPE) were developed at the end of the 1950’s, and constitute nowadays a major therapy for hematological, neurological, immunological or nephrological diseases. Studies have mainly focused on the efficacy of this therapy, while those dealing with tolerance and complications are relatively scarce and not recent.

**Patients and methods:** We conducted a monocentric retrospective study at Rennes University Hospital, between January 2011 and December 2017. We included all patients who underwent TPE during the study period. Patients under legal protection, minors and patients for whom TPE session monitoring forms were not found were excluded. TPE sessions were performed by nurses, specifically trained to the technique. The primary endpoint was the rate of all types of adverse effects occurring during TPE sessions.

**Results:** 1895 TPE sessions were analyzed (185 patients). The main indication to TPE was thrombotic microangiopathy syndrome. Replacement fluids used were albumin 4% alone (49.2%), fresh frozen plasma alone (FFP) (31.5%) or the combination of albumin 4% and FFP (19.3%). According to past recommendations hydroxyethyl starch (HES) was frequently associated when albumin was used. At least one adverse effect was reported in 805 of 1895 sessions (42.5% [29.9%-70.1%]) and 171/185 patients (92.4%). Hypotension occurred in 288 TPE sessions (15.2%), mostly asymptomatic (96.2% of cases). Hypotension was more frequent with albumin alone (19.9%, versus FFP 8.9%, albumin+FFP 13.7%, p < 0.0001) and associated with the use of HES (OR 2.56 [1.73; 3.79] p < 0.0001). Hypocalcemia occurred in 370 of sessions (19.6%) and was more frequent with the use of FFP: FFP alone 28.0%; albumin + FFP 26.0%; albumin alone 11.7%, p < 0.0001. Allergic reactions occurred in 56 sessions (3%), only with FFP. There was an increased risk of allergic reaction per liter of FFP infused (OR 1.34 [1.11–1.63] p = 0.003). We reported one case of anaphylactic shock, two cases of severe hypocalcemia. There was no death. Increased oxygen requirement was more frequent with the use of FFP.

**Conclusion:** TPE is a safe therapy, when performed by a trained team. Side effects were frequent but mostly not serious. Substitution fluid was the main determinant of the occurrence of complications. Hypotension was more frequent with albumin and associated with the use of HES. Hypocalcemia was more frequent and allergic reactions occurred only with FFP. ClinicalTrials.gov ID:NCT03888417.

**Compliance with ethics regulations:** Yes.

### P-165 Arrhythmia-induced cardiogenic shock on VA-ECMO: reduction or atrio-ventricular node ablation allow recovery

#### Guillaume Hekimian, Nicolas Paulo, Nicolas Brechot, Estelle Gandjbakhch, Xavier Waintraub, Matthieu Schmidt, Ania Nieszkowska, Paul Masi, Guillaume Franchineau, Simon Boursier, Loïc Le Guennec, Guillaume Lebreton, Alain Combes, Charles-Edouard Luyt

##### Hôpital Pitié-Salpêtrière, Paris, France

###### **Correspondence:** Guillaume Hekimian (guillaume.hekimian@aphp.fr)

*Ann. Intensive Care* 2020, **10(Suppl 1):**P-165

**Rationale:** Arrhythmia-induced cardiomyopathy has been recognized for several decades, but most severe forms, i.e. cardiogenic shock and refractory cardiogenic shock requiring mechanical circulatory support, were rarely described in adults.

**Patients and methods:** In this retrospective study, we described patients admitted in our tertiary care center for non-ischemic acute cardiac dysfunction (or worsening of previously known cardiac dysfunction) and recent onset supraventricular arrhythmia who developed cardiogenic shock requiring veno-arterial ECMO (VA-ECMO).

**Results:** In a 10 years period, 35 patients had VA-ECMO for acute non ischemic cardiac dysfunction and recent onset supraventricular arrhythmia (Table 1). Fourteen (40%) patients had known non-ischemic cardiomyopathy and 10 (29%) known paroxystic atrial fibrillation. Cardiogenic shock was the first manifestation of the disease in 21 patients. Atrial fibrillation was the main cause of arrythmia (77% of cases). At ECMO implantation, SOFA score was 10 [7–13], inotropic score 29 [11–80], LVEF 10% [10–15] and lactate level was 8 [4–11] mmol/l. Twelve patients had sustained successful reduction after amiodarone and/or electric shock, all were weaned from ECMO and 11 survived without transplantation nor long term assist device. Among the 21 patients with failure of reduction, 7 underwent an atrio-ventricular ablation while on ECMO and 1 had atrial tachycardia ablation; all were weaned from ECMO and 7 survived. Among the remaining 13 patients without reduction and without ablation procedure, only the 6 patients who were bridged to heart transplantation or left ventricular assist device survived. In univariate analysis, factors associated with unfavorable outcome were previously known heart disease, heart rate, renal replacement therapy, NT-proBNP level, failure of rhythm reduction after amiodarone load and/or electric shock. Among the 18 patients who recovered and survived (11 with successful reduction and 7 with successful ablation), LVEF increased from 10 [10–15]% before ECMO implantation to 50 [45–55]% at long term follow-up.

**Discussion:** This is the largest cohort of arrhythmia induced cardiomyopathies on VA-ECMO and the first description of atrio-ventricular node ablation with favorable outcome in this setting.

**Conclusion:** Arrhythmia induced cardiomyopathy is probably underrecognized and should be considered in any patient with non-ischemic acute cardiac dysfunction and recent onset supraventricular arrhythmia. Recovery is possible in the most severely ill patients on VA-ECMO, even with severe left ventricular dilation. Aggressive rate control by AV-node ablation may be warranted in case of failure of reduction, and may allow recovery and favorable outcome.

**Compliance with ethics regulations:** Yes.

**Table 1:** Characteristics of the patients

	Overall population, n = 35	Non survivors, n = 11	Survivors, n = 24	p
Age–year	48 [39–60]	59 [41–65]	27 [39–53]	0.21
Known non ischemic cardiomyopathy	14 (40)	9 (81.8)	5 (20.8)	0.002
Known paroxystic atrial fibrillation	10 (28.6)	6 (54.5)	4 (16.7)	0.04
Type of supraventricular arrythmia—no. (%)				
Atrial fibrillation	27 (77.1)	8 (72.7)	19 (79.2)	0.69
Atrial flutter	3 (8.6)	2 (18.2)	1 (4.2)	0.23
Atrial tachycardia	2 (5.7)	0	2 (8.3)	0.99
Junctional tachycardia	3 (8.6)	1 (9.1)	2 (3)	0.99
Successful sustained reduction after amiodarone load and/or electric shock	12 (34.3)	1 (9.1)	11 (45.8)	0.003
SOFA score	10 [7–13]	12 [8–14]	10 [7–12]	0.29
Mechanical ventilation—no. (%)	30 (85.7)	10 (90.9)	20 (83.3)	0.99
Renal replacement therapy—no. (%)	15 (42.9)	9 (81.8)	6 (25)	0.003
Inotrope Score	29 [11–80]	67 [11–173]	20 [12–63]	0.29
Heart Rate before ECMO—beats/min	150 [140–168]	140 [120–150]	158 [140–175]	0.01
Lactate—mmol/L	7.9 [4.3–11.3]	7.2 [4.1–10.5]	8 [4.3–11.4]	0.61
NT Pro BNP—pg/mL	8503 [3283–16,899]	20,019 [14,250–34,534]	4741 [2521–8503]	0.0005
Left ventricular ejection fraction—%	10 [10–15]	10 [10–15]	10 [10–15]	0.83
ECMO duration	11 [7–17]	11 [5–12]	12 [8–18]	0.29
Recovery	20 (57)	2 (18.2)	18 (75)	0.003
Bridge to LVAD	2 (5.7)	0	2	0.99
Bridge to transplantation	4 (11.4)	0	4	0.28

### P-166 Transpulmonary thermodilution detects rapid and reversible increases in lung water induced by positive end-expiratory pressure in acute respiratory distress syndrome

#### Francesco Gavelli, Jean-Louis Teboul, Xavier Monnet

##### Service de médecine intensive-réanimation, Hôpital de Bicêtre, Hôpitaux Universitaires Paris-Sud, Le Kremlin-Bicêtre, F-94270 France, Le Kremlin-Bicêtre, France

###### **Correspondence:** Francesco Gavelli (Francesco.gavelli@uniupo.it)

*Ann. Intensive Care* 2020, **10(Suppl 1):**P-166

**Rationale:** It has been suggested that, by recruiting lung regions and enlarging the distribution volume of the cold indicator, increasing the positive end-expiratory pressure (PEEP) may lead to an overestimation of extravascular lung water (EVLW) by transpulmonary thermodilution (TPTD).

**Patients and methods:** In 60 ARDS patients, we measured EVLW (PiCCO2 device) at a PEEP level set to reach a plateau pressure of 30cmH2O (HighPEEPstart) and 15 and 45 min after decreasing PEEP to 5cmH2O (LowPEEP15’ and LowPEEP45’, respectively). Then, we increased PEEP to the baseline level (HighPEEPend). Between HighPEEPstart and LowPEEP15’, we estimated the lung derecruited volume.

**Results:** Reducing PEEP from HighPEEPstart (14 ± 2cmH_2_O) to LowPEEP15’ significantly decreased EVLW from 20 ± 4 to 18 ± 4 mL/kg, central venous pressure (CVP) from 15 ± 4 to 12 ± 4 mmHg, the arterial oxygen tension over inspired oxygen fraction (PaO_2_/FiO_2_) ratio from 184 ± 76 to 150 ± 69 mmHg and lung volume by 144 [68–420]mL. EVLW decrease was similar in high and low derecruiters, defined according to the median value either of the changes in PaO_2_/FiO_2_ or of the lung volume variations. When PEEP was re-increased to HighPEEPend, CVP, PaO_2_/FiO_2_ and EVLW significantly re-increased. EVLW changes were not significantly correlated with the volume derecruited, but were correlated with changes in CVP (ρ = 0.733; p < 0.0001) and in the PaO_2_/FiO_2_ ratio (ρ = 0.478; p < 0.0001). However, at multivariate analysis, the changes in EVLW were independently associated only with the changes in CVP.

**Conclusion:** In ARDS patients, changing the PEEP level induced parallel, small and reversible changes in EVLW. These changes were not due to an artefact of the TPTD technique and are likely due to the PEEP-induced changes in CVP, which is the backward pressure of the lung lymphatic drainage.

**Compliance with ethics regulations:** Yes.

### P-167 Diagnostic and pronostic value of procalcitonin and C-Reactive-Protein in patients undergoing extracorporeal membrane oxygenation support

#### Vincent Liu, Natacha Rousse, Antoine Lamer, Elena Madalina Nodea, Guillaume Gantois, André Vincentelli, Emmanuel Robin, Mouhammed Moussa

##### Réanimation chirurgicale cardiovasculaire, Institut Coeur-Poumon, CHU Lille, Lille, France

###### **Correspondence:** Vincent Liu (vincent.liu@hotmail.fr)

*Ann. Intensive Care* 2020, **10(Suppl 1):**P-167

**Rationale:** Diagnosis of sepsis is a major challenge in intensive care units and is associated with a high morbidity and mortality. Sepsis identification is even more difficult in patients with extracorporeal membrane oxygenation (ECMO) because of many confounding factors. The primary objective was to study the ability of C-reactive protein (CRP) and procalcitonin (PCT) values measured at ECMO support initiation (day 0) to predict the occurrence of early sepsis in patients undergoing venoarterial ECMO (va-ECMO) or venovenous ECMO (vv-ECMO). The secondary objectives were to study the association between these biomarkers and mortality rate during ECMO support and in-hospital mortality rate. Furthermore, we investigated the relationship between early sepsis and mortality.

**Patients and methods:** We performed a retrospective, monocentric study in the cardiovascular Intensive Care Unit of the University Hospitals of Lille, France. Between November 1, 2014 and December 31, 2017, we included patients over 18 years old, who underwent an ECMO support for a medical or surgical indication, and for whom biomarkers (CRP and PCT) levels were available for at least the first 2 days of admission. Biomarkers and blood cultures were daily assessed for the first ECMO support days. Early sepsis was defined by sepsis diagnosis in the first 7 days after circulatory assistance initiation. In-hospital mortality rate was censored at 28 days. After univariate analysis, a Cox multivariate regression model was used to assess if the association between biomarkers levels and early sepsis or mortality rate was independent. A Kaplan–Meier survival plot was used to describe the association between early sepsis and mortality.

**Results:** Among 100 patients included, 68 underwent va-ECMO and 32 underwent vv-ECMO. An early sepsis diagnosis was made in 17.6% of va-ECMO patients and in 100% of vv-ECMO patients. PCT and CRP levels on day 0 were significantly associated with early sepsis diagnosis (Fig. 1). No difference was found between PCT-D0 or CRP-D0 and mortality under ECMO support or in-hospital mortality rates. Following multivariate analysis, PCT-D0 was independently associated with the occurrence of sepsis, HR = 1.04 (95% CI 1.01–1.08, p = 0.04) only in the va-ECMO population (table 1). After ROC curve analysis, the AUC of PCT-D0 was 0.64, (95% CI 0.51–0.77), resulting in respective positive and negative likelihood ratios of 2.47 and 0.48 for the optimal PCT-D0 threshold of 2.45 ng/mL.

**Conclusion:** PCT level on day 0 was independently associated with early sepsis diagnosis in patients undergoing ECMO-va support. Besides, early sepsis was associated with in-hospital mortality.

**Compliance with ethics regulations:** Yes

**Fig. 1:**
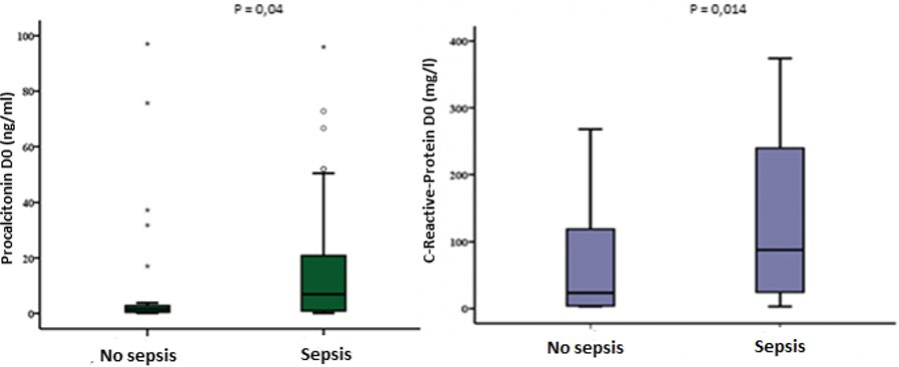
Compared values of PCT and CRP at day 0 according to sepsis

**Table 1:**
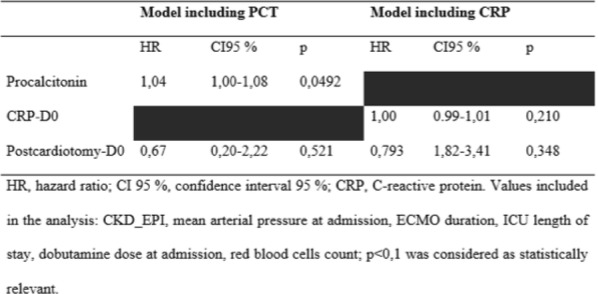
Multivariable analysis of factors associated with sepsis occurrence in the vv-ECMO population

### P-168 Does the infusion speed impact the haemodynamic effect of a fluid bolus in septic shock patients? A pharmacodynamic study

#### Arthur Pavot, Francesco Gavelli, Jean-Louis Teboul, Rui Shi, Nello De Vita, Xavier Monnet

##### Hôpital de Bicêtre, Le Kremlin-Bicêtre, France

###### **Correspondence:** Arthur Pavot, (pavot.arthur@gmail.com)

*Ann. Intensive Care* 2020, **10(Suppl 1):**P-168

**Rationale:** Fluids are one of the most prescribed drug in intensive care, particularly among patient with circulatory failure. Yet, very little is known about their pharmacodynamic properties and this topic has been left largely unexplored. Several factors may impact the haemodynamic efficacy of fluids among which the infusion rate. The aim of this study was to investigate the influence of the rate of fluid administration on the fluid pharmacodynamics, in particular by studying mean systemic pressure (Pms).

**Patients and methods:** We conducted a prospective observational study in 14 patients with septic shock to compare two volume expansion strategies. A fluid bolus, 500 mL of normal saline were administered and several haemodynamic variables were recorded continuously: cardiac output (CO), arterial pressure (AP), mean systemic pressure (Pms, estimated from CI, PVC and MAP). Infusion rate was left at the discretion of the attending physician. A “slow” and a “fast” groups were determined based on the median of the infusion duration. Fluids effect was measured by the area under the curve (AUC), maximal effect (Emax) and time to maximal effect (tmax) for each haemodynamic variable.

**Results:** The effects of fluid on Psm disappeared in one hour on average. Compared to patients of the “slow” group, those of the “fast” group had a shorter tmax and a higher Emax for Pms (p = 0.046 and 0.02 respectively). The AUC for Pms was identical between group, while in case of similar effect of infusion rates, it should be larger in the “slow” group. Regarding CO, tmax was also shorter in the “fast” than in the “slow” group (p = 0.048). The decreasing slope from maximal effect was comparable between groups, for Pms as for CO.

**Conclusion:** The effect of a 500 mL fluid bolus with normal saline in septic shock patients vanished within one hour. A faster infusion rate increased the maximal and total effect of the fluid bolus and shortened the delay to reach the maximal effect. The study is ongoing.

**Compliance with ethics regulations:** Yes.

### P-169 PROtocolized Care to Reduce HYpotension after Spinal Anaesthesia (ProCRHYSA randomized trial): final results

#### Samuele Ceruti^1^, Mathieu Favre^2^, Silvia Bosio^3^, Bruno Minotti^4^, Marco Spagnoletti^5^, Michele Musiari^6^, Andrea Saporito^7^

##### ^1^Hôpitaux Universitaires de Genèvetaux, Genève, Switzerland; ^2^Hôpitaux Universitaires de Genève, Genève, Switzerland; ^3^Clinica Luganese, Lugano, Switzerland; ^4^Kantonsspital St. Gallen, St. Gallen, Switzerland; ^5^Clinica Luganese, Lugano, Switzerland; ^6^Hôpital Fribourgeois Regional (HFR), Fribourg, Switzerland; ^7^Ente Ospedaliero Cantonale, Bellinzona, Switzerland

###### **Correspondence:** Samuele Ceruti (samuele.ceruti@me.com)

*Ann. Intensive Care* 2020, **10(Suppl 1):**P-169

**Rationale:** Significant hypotension following spinal anesthesia is a common issue in everyday clinical practice. To avoid this potentially harming situation, an empirical fluid administration is usually performed before the procedure. Inferior vena cava (IVC) ultrasound has been demonstrated effective in guiding fluid therapy in critical care patients. The purpose of this study was to evaluate the IVC ultrasound guided volemic status optimization in order to decrease post-spinal hypotension rate.

**Patients and methods:** In this prospective, controlled, randomised study, 474 consecutive patients were recruited and 429 patients were randomly assigned to a control group, consisting of pre-anesthesia empirical fluid administration (ITT), an IVC ultrasound group in which fluid management was based on an IVC ultrasound evaluation, and a passive leg raising test (PLRT) group in which volume optimization was performed following the above mentioned test. Primary outcome was the hypotension rate reduction after spinal anaesthesia following fluid optimization therapy between the groups. Secondary outcomes were the total fluid amount administered, the total vasoactive drug amount used and the time needed to realize the whole anaesthetic procedure in all three groups.

**Results:** 11% reduction in hypotension rate (95% CI 1–24%, p = 0.086) was observed between the echocardiography group and the control group, and there was a reduction of hypotension rate by 9% (CI 95% 3–21%, p = 0.154) between the echocardiography group and the PLRT group. The total fluid amount administered was significantly greater in the ultrasound group than in the control group (593 ml; SD 369 ml, versus 453 ml; SD 458 ml, p = 0.01498). The total amine consumption was 36% in control group, 16% in IVC group and 22% in PLRT group. An increased of total study time was observed for the echocardiography group 48 min (SD 10 min) in comparison with the control group 46 min (SD 27 min) and PTLR group 40 min (SD 13 min), (p < 0.001).

**Conclusion:** The study showed a faint but positive trend toward the use of IVC-ultrasound to identify patients in spontaneous breathing needing fluid optimization before spinal anesthesia

**Compliance with ethics regulations:** Yes.

**Fig. 1:**
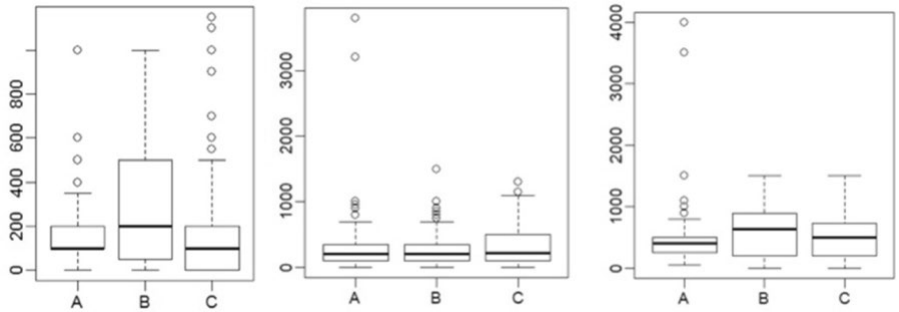
Comparison boxplot between the control group (group A), the echocardiography group (group B), and the PLRT group (group C), concerning the administration of crystalloids. Here are represented data of the pre-anesthesia phase, the post anesthesia phase and globally (pre- and post-anesthesia phase)

### P-170 Does the end-expiratory occlusion test predict preload responsiveness? A systematic review and meta-analysis

#### Francesco Gavelli, Rui Shi, Jean-Louis Teboul, Xavier Monnet

##### Hôpital de Bicêtre, Le Kremlin-Bicêtre, France

###### **Correspondence:** Francesco Gavelli (francesco.gavelli@uniupo.it)

*Ann. Intensive Care* 2020, **10(Suppl 1):**P-170

**Rationale:** We performed a systematic review and a meta-analysis of studies investigating the ability of the end-expiratory occlusion (EEXPO) test to predict preload responsiveness, through the changes in cardiac output (CO) or its surrogates, in adult patients.

**Patients and methods:** This meta-analysis was prospectively registered on PROSPERO (CRD-42019138265). We screened PubMed, EMBASE and Cochrane Database to identify all original articles published between 1960 and 2019 evaluating the ability of the EEXPO test to predict a significant increase in CO or surrogate, compared to the one induced by a subsequent volume expansion or by passive leg raising (PLR). The meta-analysis determined the pooled area under the receiver operating characteristics curve (AUROC) of EEXPO test-induced changes in CO to detect preload responsiveness, as well as pooled sensitivity and specificity and the best diagnostic threshold. Subgroup analysis and sensitivity analysis were planned to investigate potential sources of heterogeneity.

**Results:** Thirteen studies (530 patients) were identified and included in the analysis. Nine studies were performed in the intensive care unit and four in the operating room. Preload responsiveness was defined according to CO changes induced by fluid administration in 12 studies (fluid-induced increase in CO ≥10% or ≥15%) and according to CO changes induced by PLR in one study. The duration of the respiratory hold ranged between 12 and 30 s. For the EEXPO test-induced changes in CO, the pooled sensitivity and specificity were 87 [82-91]% and 91 [87-94]%, respectively, while the pooled AUROC curve was 0.95 ± 0.01 (Fig. 1). The corresponding best diagnostic threshold was 5.1 ± 0.2%. When changes in CO were monitored through pulse contour analysis compared to other methods the accuracy of the test was significantly higher (AUROC: 0.97 ± 0.01 vs. 0.91 ± 0.02, respectively, p < 0.01). When the diagnostic ability of the EEXPO test was evaluated according to the tidal volume (≤7 mL/kg vs. > 7 mL/kg), the PEEP level (≤ 7cmH_2_O vs. > 7cmH_2_O) or the duration of the EEXPO (≤ 15 s vs. > 15 s) no significant difference was observed in terms of AUROC comparison (p > 0.05). However, subgroup analysis identified tidal volume, the method used to monitor CO, the level of PEEP as well as the research team publishing the studies as potential sources of heterogeneity. One study was identified as source of heterogeneity at sensitivity analysis.

**Conclusion:** The changes in CO induced by the EEXPO test reliably detect preload responsiveness. The diagnostic performance is significantly higher when the EEXPO test-induced changes in CO are assessed through the pulse contour analysis method.

**Compliance with ethics regulations:** Not applicable.

### P-171 Acute kidney injury and risk of pulmonary embolism in patients with septic shock

#### Rania Ammar Zayani, Karama Bouchaala, Hela Kallal, Chokri Ben Hamida, Mounir Bouaziz

##### Habib bourguiba university hospital, Sfax, Tunisia

###### **Correspondence:** Rania Ammar Zayani (rania.ammarzayani@gmail.com)

*Ann. Intensive Care* 2020, **10(Suppl 1):**P-171

**Rationale:** Septic acute kidney injury (S-AKI) is a frequent complication in critically ill patients and is associated with high morbidity and mortality. It is well known that chronic kidney disease increases the risk of pulmonary embolism (PE), but few studies have investigated the relationship between acute kidney injury (AKI) and PE occurrence in septic patients. The aim of this study is to determine whether patients with AKI are at increased risk of developing PE.

**Patients and methods:** Were included, in a prospective study conducted over 6 months (January 01–June 30, 2018) in a medical surgical intensive care unit, all the patients older than 18 years with septic shock at admission or during hospitalization. Two groups were compared: patients with kidney injury (AKI+ group) and patients without kidney injury (AKI− group). We studied the occurrence of PE in these two groups.

**Results:** We included 75 patients. The mean (SD) age was 56.43( ± 18) years. Sex ratio was 1.91. Thirty one (41.3%) patients developed PE. The occurrence of PE was significantly higher in (AKI + group) [27 patients (51%) vs. 4 patients (17%); p = 0.006]. The incidence of PE according to kidney injury severity was 2 patients (10%) KDIGO I, 5 patients (50%) KDIGO II, 20 patients (86%) KDIGO III. In the AKI+ group, PE was significantly associated with increased SOFA score at admission (10 points vs. 6 points; p = 0.02), lower platelets count (95,000 vs. 145,000; p = 0.032), higher lacatatemia at septic shock day [6.20 vs. 4.9 mmol/l; p = 0.003] and higher C reactive protein level [223 mg/l vs. 150 mg/l; p = 0.005]. In a multivariate analysis the PE risk factors in (AKI+ group) were thrombopenia (Odds Ratio = 1.01; CI [1.01–1.02], p = 0.01) and C-reactive protein value (Odds Ratio = 1.016; CI[1.005–1.027], p = 0.001).

**Discussion:** The increased risk for PE with AKI may be due to endothelial involvement, vascular injury and the related changes found in procoagulant proteins (increased levels of fibrinogen, factor VII, factor VIII, von Willebrand factor, and plasminogen activator inhibitor-1). In our study, lower platelet and higher C reactive protein level were found in patients with PE, suggesting the participation of disseminated intravascular coagulation. These factors may contribute to increase PE risk.

**Conclusion:** The risk of PE is higher in septic patients with AKI than in those with normal kidney function. Therefore, because of paucity of evidence, larger studies are needed to understand PE pathway in septic AKI and to establish efficient prophylaxis protocols.

**Compliance with ethics regulations:** Yes.

### P-172 Extravascular lung water levels and kinetics are associated with mortality: a systematic review and meta-analysis

#### Francesco Gavelli, Rui Shi, Jean-Louis Teboul, Xavier Monnet

##### Service de médecine intensive-réanimation, Hôpital de Bicêtre, Hôpitaux Universitaires Paris-Sud, Le Kremlin-Bicêtre, F-94270 France, Le Kremlin-Bicetre, France

###### **Correspondence:** Francesco Gavelli (Francesco.gavelli@uniupo.it)

*Ann. Intensive Care* 2020, **10(Suppl 1):**P-172

**Rationale:** Even though many studies have shown that an elevation in extravascular lung water (EVLW) measured by transpulmonary thermodilution (TPTD) is associated to increased mortality in critically ill patients, its role remains a matter of debate. In this systematic review and meta-analysis, we aimed at evaluating the relationship between increased EVLW estimated by TPTD and mortality in critically ill patients.

**Patients and methods:** PubMed, EMBASE and Cochrane Database were screened for original articles published between 1960 and 2019. We performed a meta-analysis using random effects to estimate the pooled relative risk of death associated with elevated EVLW, and the pooled area under the receiver operating characteristics curve (AUROC) of elevated EVLW as a predictor of mortality. Missing data were provided by the authors of the original studies.

**Results:** We included fifteen studies (1086 patients in total). The pooled relative risk of mortality with elevated EVLW levels was 4.10 [2.52–6.68]. The pooled AUROC, estimated from 13 studies, was 0.84 ± 0.04 with a derived threshold of 14.8 ± 1.2 mL/kg. The pooled sensitivity and specificity were 70 [65–74]% and 69 [65–73]%, respectively. Both the baseline and the maximal EVLW values were significantly different among survivors and non-survivors, as well as EVLW variation over time. The results of the analyses comparing indexation of EVLW to actual vs. predicted body weight and ARDS patients vs. non-ARDS patients were robust and consistent with primary analysis. Moreover, neither subgroup nor sensitivity analyses identified potential sources of heterogeneity. According to the multivariable analysis performed in seven studies, the odds ratio of elevated EVLW for mortality ranged from 1.01 to 6.21.

**Conclusion:** The level of EVLW measured by TPTD and its changes over time are associated with mortality in critically ill patients, which may emphasize its clinical value.

**Compliance with ethics regulations:** Yes.

### P-173 The rheumatic mitral stenosis in intensive care: epidemiology, clinical and feature management

#### Fahd Moussaid, Abir Abardazzou, Hamza Elhamzaoui, Taoufik Abouelhassan

##### Emrgency departement, University Hospital, Marrakech, MOROCCO

###### **Correspondence:** Fahd Moussaid (fahdmoussaid5@gmail.com)

*Ann. Intensive Care* 2020, **10(Suppl 1):**P-173

**Rationale:** In Morocco, rheumatic valve disease is a major public health problem affecting children and young adults. Mitral stenosis (MS) is defined as a permanent mechanical obstruction of the mitral valve, interfering with ventricular filling. MS has hemodynamic consequences on the small pulmonary circulation. This pathology of exposure is a problem of complications and the therapeutic decision. The aim of our study is to determine epidemiological, clinical and therapeutic of mitral stenosis in intensive care .

**Patients and methods:** This is a retrospective study over 1 year, within the emergency department of Mohamed VI University Hospital, Marrakech. Inclusion criteria: age of patients over 18 years, echocardiographic diagnosis of mitral narrowing: commissural fusion, calcification, shortened subvalve apparatus, thickened and/or retracted sheet. Exclusion criteria: mechanical or biological prostheses and age over 60 years. The statistical analysis was performed by the SPSS software.

**Results:** Forty patients were admitted to the intensive care unit for mitral stenosis. Average age was 35 years. Sex ratio: 1.3. Clinical picture indicative of MS was dominated by acute lung edema in 40% of cases, rapid atrial fibrillation in 30%, embolic events in 20%, and cardiogenic shock in 10%. Echocardiography diagnosed mitral narrowing as follows: 60% tight, 30% very tight and 10% moderately tight, and 40% of MRs (tight and very tight) were classified as fibrous, and suitable for percutaneous mitral dilation. Initial management was symptomatic, with inotropic positive agents used in 15% of cases, non-invasive ventilation (NIV) in 60% of cases and mechanical ventilation in 10%.

**Conclusion:** Mitral rheumatic stenosis is the prerogative of the young subject and a public health problem in Morocco, a long and silent evolution, complications are often a mode of revelation essentially acute lung edema. Prevention is important through programmes to fight acute rheumatic fever.

**Compliance with ethics regulations:** Yes.

### P-174 Prognostic value of C-reactive protein and quick SOFA in patients with sepsis at the Emergency Department

#### Hadil Mhadhbi, Khedija Zaouche, Abderrahim Achouri, Hamida Maghraoui, Kamel Majed

##### Emergency Department la Rabta, Tunis, Tunisia

###### **Correspondence:** Hadil Mhadhbi (hadil.mhadhbi@gmail.com)

*Ann. Intensive Care* 2020, **10(Suppl 1):**P-174

**Rationale:** Sepsis and septic shock are common causes of mortality in critically ill patients. Rapid identification of sepsis patients at high risk of early mortality either with readily available laboratory parameter or with a clinical assessment is necessary. Various studies have reached contradictory conclusions regarding the prognostic value of C-reactive protein (CRP) in patients with sepsis at the Emergency Department (ED). This study aimed to assess the prognostic value of CRP on sepsis and compare it with quick SOFA (q SOFA).

**Patients and methods:** We conducted a prospective observational study. Adult patients admitted to the ED fulfilling the surviving sepsis campaign third definition of sepsis were enrolled. The main outcome was early (within 48 h) and late (after 30 day) mortality.

**Results:** During the study period, 100 patients were enrolled .The mean age was 65 ± 13 years and 52% of the patients were men. The mean serum CRP was 177 mmol/L. The early mortality rate was 21%.The late mortality rate was 19%. In predicting early mortality, the AUROC was 0.86 (95% CI 0.75–0.97) for CRP and 0.83 (95% CI 0.72–0.95) for q SOFA score. CRP demonstrated superior efficacy in predicting 30-day mortality compared to q SOFA. AUROC was 0.75 for CRP, and 0.52 for qSOFA.

**Conclusion:** This study showed that CRP is a useful predictor of early and late mortality in patients with sepsis at the ED setting.

**Compliance with ethics regulations:** Yes.

### P-175 Usefulness of the C reactive protein in prognosis assessment of patients with sepsis and septic shock at Emergency Department

#### Nadia Zaouak, Yosra Yahya, Khedija Zaouche, Radhia Boubaker, Abdelwahab Mghirbi, Hamida Maghraoui, Kamel Majed

##### Emergency Department of La Rabta Teaching Hospital, Tunis, Tunisia

###### **Correspondence:** Nadia Zaouak (zaouak.nadia@yahoo.fr)

*Ann. Intensive Care* 2020, **10(Suppl 1):**P-175

**Rationale:** Sepsis and septic shock are the most common causes of death in hospitals. Some studies have shown that changes in CRP concentrations are related to the prognosis of patients with sepsis. The aim of this study was to evaluate the clinical significance of initial CRP in patients who suffered from sepsis and septic shock.

**Patients and methods:** This is an observational retrospective study conducted in all patients admitted to Emergency Department (ED) for sepsis or septic shock . The period of study was 8 months. Data of all patients were collected. Patients were divided into sepsis and septic shock groups, and into survivor and non-survivor groups. The statistical analysis concerned the C reactive protein (CRP) at admission.

**Results:** 105 patients were included. The mean age of patients was 74 ± 15 years with a sex ratio of 1.37. The pulmonary sepsis was the most frequent localisation (60%). 9% presented a sepsic shock at admission. The median SOFA score was 2. The mean of CRP at admission was 163 ± 137 mg/L in septic shock group versus 120 ± 130 mg/L in sepsis group (p = 0.00). The levels of CRP were not significantly different between non-survivor and survivor groups: 166 ± 132 mg/L versus 113 ± 131 mg/L (p = 0.08).

**Conclusion:** Our study showed that initial CRP seems to be a predictor biomarker of initial gravity but it wasn’t useful for predicting inhospital mortality in patients admitted to ED for sepsis and septic shock.

**Compliance with ethics regulations:** Yes.

### P-176 Procalcitonin (PCT) kinetics in septic burn patients

#### Sarra Dhraief^1^, Sajida Sboui^2^, Karim Mechri^1^, Amel Mokline^2^, Lamia Thabet^1^, Hana Fredj^2^, Lilya Debbiche^2^, Wael Chemli^2^, Bahija Gasri^2^, Amenallah Messaadi^2^

##### ^1^Departement of Clinical Biology, Burn and Trauma Center–Tunis, Tunisia, Ben Arous, Tunisia; ^2^Intensive Burn Care Departement, Burn and Trauma Center-Tunis, Tunisia, Ben Arous, Tunisia

###### **Correspondence:** Sarra Dhraief (dhraiefsarra@gmail.com)

*Ann. Intensive Care* 2020, **10(Suppl 1):**P-176

**Rationale:** Sepsis still the major cause of mortality in burn patients. Standard clinical and biological parameters lack of sensitivity and specificity. PCT is the most specific.The aim of present study is to evaluate the antibiotic treatment guided by PCT kinetics.

**Patients and methods:** A prospective study in burns ICU in Tunisia was conducted from August 2018 to August 2019. Were included patients with predictive sings of sepsis according to the SFB criteria for the presence of infection. 34 were excluded due to a short course in the ICU. The value of PCT to initiate antibiotic is 0.69 ng/ml (1). Continuing (decrease to 30% of peak level) or modification (decrease < 30%) of antibiotic therapy was guided by a serum PCT assay from the third day of treatment and every 48 h until antibiotic was stopped. This last was stopped when PCT levels had decreased of 80% from the initial value.

**Results:** A total of 106 patients had been diagnosed as sepsis (n = 79, 75%) and septic shoc (n = 27, 25%). Mean age was 32 years ± 14. An average UBS and ABSI score of 40% and 6. The average length of stay in ICU was 13 days. Patients were assigned into two groups: Group A (favorable evolution, n = 76); Group B (unfavorable evolution, n = 30). The therapeutic attitude according to the kinetics of the PCT are presented in the Table 1. We found a significant difference between patients with unfavorable evolution compared to those with a favorable evolution (in whom we stopped antibiotics) (p < 0.005), in terms of hemodynamic state, PCT concentration and renal clearance. PCT-guided antibiotic treatment has been proven to significantly reduce length of antibiotic therapy in our patients. The average duration of antibiotic was 5.6 ± 3 days.

**Conclusion:** PCT measurement may help with the decision to initiate antibiotic therapy in low risk acuity of infection and allows more judicious antibiotic use by reducing antibiotic exposure.

**Compliance with ethics regulations:** Not applicable.

**Table 1:**
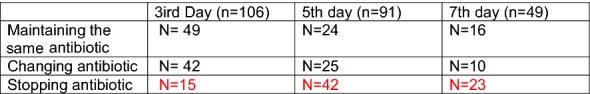
The therapeutic attitude according to the kinetics of the PCT

### P-177 Epidemiological profile and antibiotic susceptibility of blood culture isolates from burn patients in Tunisia

#### Mehdi Gaddas^1^, Sarra Dhraief^1^, Emna Hammami^1^, Amel Mokline^2^, Amenallah Messaadi^2^, Lamia Thabet^1^

##### ^1^Centre de Traumatologie et des Grands Brûlés de Ben Arous-Laboratoire de biologie médicale et banque du sang, Ben Arous, Tunisia; ^2^Centre de Traumatologie et des Grands Brûlés de Ben Arous-Service de réanimation des brûlés, Ben Arous, Tunisia

###### **Correspondence:** Mehdi Gaddas (gaddasmehdi@icloud.com)

*Ann. Intensive Care* 2020, **10(Suppl 1):**P-177

**Rationale:** Intensive care unit (ICU)-acquired bacteraemia is prevalent and poses a severe threat. Providing information about the main causative bacterial agents and determination of their susceptibility to antibiotics may improve empiric therapy and early detection of emerging antimicrobial resistance. The aim of this study was to investigate the species distribution and antibiotic susceptibility of isolated strains from blood cultures in a burn ICU.

**Patients and methods:** We retrospectively studied all strains isolated from positive blood cultures of burn patients during a seven-year period (from January 2012 to December 2018). Bacteriological study of blood was performed on BD BACTECTM blood culture flasks (Becton Dickinson^®^) which were incubated in the BACTECTM system. All isolated microorganisms were identified on the basis of conventional microbiological techniques. Antibiotic susceptibility testing was carried out by the agar disk diffusion method, and susceptibility results were interpreted using clinical breakpoints according to CASFM guidelines. Data were analyzed using the SIR-system.

**Results:** During the study period, 3206 non-repetitive strains were isolated. The most frequently identified species were *Staphylococcus aureus* (16.3%), *Acinetobacter baumannii* (11.1%), *Pseudomonas aeruginosa* (10.3%) and *Klebsiella pneumoniae* (10.2%). The rate of methicillin-resistant *Staphylococcus aureus* (MRSA) was 66.5%. Resistance rate to gentamicin and ciprofloxacin was 59.4% and 52.4%, respectively. Resistance to tigecycline and linezolid were rare, with susceptibility rates of 87.1% and 98.8%, respectively. All *S. aureus* strains were susceptible to glycopeptides. In addition, isolates of *Acinetobacter baumannii* showed high rates of resitance to all tested antibiotics except colistin. Ninety three percent of these strains were resistant to imipenem, 88.7% to amikacin and 45.8% to tigecycline. Similarly high resistance rates were observed among *Klebsiella Pneumoniae* and *Pseudomonas aeruginosa* to ceftazidime (71.4% and 42.5%, respectively), amikacin (39.2% and 76.7%, respectively) and ciprofloxacin (39.2% and 66.7%, respectively). Resistance rates of *Klebsiella Pneumoniae* and *Pseudomonas aeruginosa* to imipenem were 25% and 73%, respectively.

**Conclusion:** Regards to the high antibiotic resistance rates of strains isolated from blood cultures in burn intensive care unit, regular updating of epidemiological date is required. Streamlining antibiotics prescription and better hygiene measures application are also mandatory.

**Compliance with ethics regulations:** Yes.

### P-178 Epidemiological profile and antibiotic susceptibility of blood culture isolates in an intensive care unit in Tunisia

#### Karim Mechri, Sarra Dhraief, Mehdi Gaddas, Emna Hammami, Sonia Ben Behi, Lamia Thabet

##### Centre de Traumatologie et des Grands Brûlés de Ben Arous-Laboratoire de biologie médicale et banque du sang, Ben Arous, Tunisia

###### **Correspondence:** Karim Mechri (karimmechri91@gmail.com)

*Ann. Intensive Care* 2020, **10(Suppl 1):**P-178

**Rationale:** Intensive care unit (ICU)-acquired bacteraemia is prevalent and poses a serious threat. Providing information about the main causative bacterial agents and determination of their susceptibility to antibiotics may improve empiric therapy and early detection of emerging antimicrobial resistance. The aim of this study was to investigate the species distribution and antibiotic susceptibility of isolated strains from blood cultures in an ICU at the trauma and burn center in Tunisia.

**Patients and methods:** This seven-year retrospective study (from January 2012 to December 2018) included all strains isolated from positive blood cultures. Incubation of blood culture vials (BD BACTECTM, Becton Dickinson^®^) and the detection of bacterial growth were performed by the BACTECTM system. All isolated microorganisms were identified on the basis of conventional microbiological techniques. Antibiotic susceptibility testing was carried out by the agar disk diffusion method, and susceptibility results were interpreted using clinical breakpoints according to CA-SFM guidelines. Data were analyzed using the SIR-system. Minimum inhibitory concentrations of colistin (MIC) were determined using the E-Test^®^ method (bioMérieux), then the EUCAST broth micro-dilution method (UMIC, Biocentric^®^) since May 2017.

**Results:** During the study period, 1284 non-repetitive strains were isolated. The most frequently identified species were *Acinetobacter baumannii* (17.8%), *Klebsiella pneumoniae* (15.2%), *Staphylococcus aureus* (14.7%) and *Staphylococcus epidermidis* (7.3%). Isolated *A. baumannii* strains showed high rate of resistance to all tested antibiotics except colistin. Ninety-six percent of these strains were resistant to imipenem, 89.2% to ceftazidime, 86% to amikacin and 97.4% to ciprofloxacin. Three strains were resistant to colistin. Concerning *K. pneumoniae*, 71.2% of the strains were resistant to cefotaxime. Only 1.9% of strains were resistant to imipenem. All strains were susceptible to colistin. The rate of methicillin-resistant *Staphylococcus aureus* (MRSA) was 56%. Resistance rates to gentamicin, ciprofloxacin and tigecycline were 49.3%, 42.2% and 6.2%, respectively. All strains were susceptible to glycopeptides and linezolid.

**Conclusion:** This study investigated on the local distribution patterns of causative miroorganisms of bacteraemia in an ICU at the Trauma and Burn Center in Tunisia, and the correspondant antimicrobial susceptibility profiles. Multridrug-resistant pathogens, especially *Acinetobacter baumannii*, were the most frequently isolated organisms. Hygiene measures should be intensified to prevent the spreading of these resistant strains.

**Compliance with ethics regulations:** Yes.

### P-179 Community infection at *Staphylococcus aureus* with Panton Valentine Leucocidin in new caledonia polyvalent ICU : a descriptive study

#### Florent Vincent, Benoit Marot, Pauline Genieys

##### CHT Gaston Bourret, Noumea, France

###### **Correspondence:** Florent Vincent (vincent.florent.bis@gmail.com)

*Ann. Intensive Care* 2020, **10(Suppl 1):**P-179

**Rationale:** Panton Valentine Leucocidin (PVL) *Staphylococcus aureus* (SA) infections are steadily increasing. This disease, responsible for high mortality, drives patients in ICU, especially in its well-studied necrotizing pneumonia form. But other clinical presentations, also severe, are less well known. The therapeutic emergency must not be delayed, since a wide therapeutic arsenal—antibiotherapy, anti-toxinic and immunoglobulins—exists.

**Patients and methods:** Monocentric retrospective study performed in the only one ICU in New Caledonia. All community patients admitted in ICU between march 2010 and September 2016 with a positive sample at SA PVL+ were included. Clinical, radiological, biological, demographic and therapeutic data were analyzed.

**Results:** 34 patients were included. The mean patient with SA PVL+ admitted to the ICU is a young (32.2 years) male (76.5%), with no past medical history, with symptoms lasting for over 48 h. Clinical presentation is mostly cutaneous, then profound abscess and pulmonary (Table 1). Furuncle is classically found in the preceding 30 days (41.7%). 38.1% of patient had a septic shock with 26.5% requiring intubation for ARDS. MRSA is found in 41.2% of the bacteriological samples, and 72.7% of the patients had a bacteraemia. A precocious targeted antibiotherapy improves the chance of recovery. A septic shock, intubation or multiple visceral failure syndrome are strong mortality risk factors.

**Conclusion:** The continuation of this study with more patients is necessary to confirm identified mortality risk factors (MRSA, lack of anti-toxinic) or protective factors (use of immunoglobulin, antibiotherapy delay) and to precise infection at SA PVL+ risk factors (furuncle?). One proposed improvement will then be to perform subgroup analyzes (necrotizing pneumonia, deep abscess, children).

**Compliance with ethics regulations:** Yes.

**Table 1:**
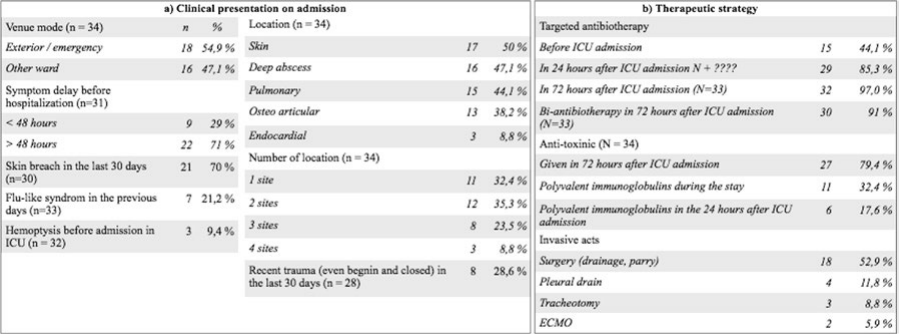
Characteristics of the study: (a) clinical presentation on admission, (b) therapeutic strategy

### P-180 Evaluation of intubating conditions using stylet by conventional through-tube technique and through Murphy’s eye in patients with high Mallampati scores

#### Haider Abbas

##### King George’s Medical University, Lucknow, India

###### **Correspondence:** Haider Abbas (haiderup@gmail.com)

*Ann. Intensive Care* 2020, **10(Suppl 1):**P-180

**Rationale:** Difficult intubation is a nightmare and this study was planned to study the alternative method of use of stylet during difficult oro-tracheal intubation in limited resource situations.The outcome measures assessed were ease of intubation, hemodynamic stability and reducing complications.

**Patients and methods:** After ethical approval, a cohort of 60 patients of Mallampati class III was formed and patients were divided into two groups. In group 1 patients, conventional through tube method was used for inserting stylet, whereas, in group 2 patients, Murphy’s eye was used for inserting malleable flexi tip stylet.

**Results:** Primary outcome measures included mean arterial pressure (MAP), heart rate (HR) and secondary outcome measures were intubation time, saturation drop, ease and number of attempts and pharyngo-laryngeal complications. Hemodynamic stability in terms of MAP and HR was observed in group 2. Intubating time, number of successful intubations and pharyngo-laryngeal (including dislodgement of tube on removal of stylet) complications was also low in group 2.

**Discussion:** Use of stylet through Murphy’s eye showed higher success rate on first intubation attempt, produced attenuated hemodynamic response, better oxygenation, less pharyngo-laryngeal complications as compared to the conventional methods of endotracheal intubation in patients with high Mallampati score.

**Conclusion:** Thus, using Murphy’s eye to slide endotracheal tube over stylet is an effective alternative to conventional rail-road method in these patients especially in developing world and in situations where bougie is not available.

**Compliance with ethics regulations:** Yes.

### P-181 Ultrasound sliding lung in confirming absence of selective endotracheal intubation: An ancillary study from the prospective multicenter trial EPIC

#### Vanessa Jean-Michel^1^, Gwenael Prat^2^, Pierre Bailly^2^, James Benis^3^, Montaine Lefevre^4^, Pierre-Yves Egreteau^4^, Christophe Giacardi^5^, Cécile Aubron^2^, Erwan L’Her^2^

##### ^1^CH de Tourcoing, Tourcoing, France; ^2^CHRU de la Cavale Blanche, Brest, France; ^3^CH de Quimper, Quimper, France; ^4^CH de Morlaix, Morlaix, France; ^5^HIA Clermont Tonnerre, Brest, France

###### **Correspondence:** Vanessa Jean-Michel (vanessa.jeanmichel@gmail.com)

*Ann. Intensive Care* 2020, **10(Suppl 1):**P-181

**Rationale:** Emergency endotracheal intubation in the Intensive Care Unit (ICU) often concerns hypoxemic patients. Unrecognized selective intubation can lead to atelectasis. This study aimed to evaluate the performance of the ultrasound bilateral sliding lung sign as a predictor of proper endotracheal tube placement.

**Patients and methods:** A multicenter prospective study (EPIC) was performed from June 2017 to November 2018 in three ICUs. After intubation, bedside ultrasound was performed with a transducer placed bilaterally on the chest at the mid-axillary time, to identify lung sliding during ventilation. Chest radiography was used as the criterion standard for confirmation of endotracheal tube position

**Results:** Forty-three patients needing endotracheal intubation were included. The sensitivity of ultrasound to confirm proper endotracheal intubation was 98% [95% CI 87–100]. The positive and negative predictive value were 100% and 0%, respectively. Only one case of no one-sided lung sliding was a pneumothorax diagnosed by chest radiography. No selective intubation was found.

**Conclusion:** Ultrasound bilateral lung sliding is an accurate and rapid method to confirm proper endotracheal intubation.

**Compliance with ethics regulations:** Yes.

### P-182 Submandibular sonography in predicting difficult airway in anesthesia

#### Wejden Yacoubi, Faten Haddad, Asma Ben Souissi, Skander Naimi, Sahar Abdallah, M’hamed Sami Mebazaa

##### Mongi Slim teaching hospital, Tunis, Tunisia

###### **Correspondence:** Wejden Yacoubi (wejden.8988@yahoo.com)

*Ann. Intensive Care* 2020, **10(Suppl 1):**P-182

**Rationale:** Difficult airway management is associated with serious morbidity and mortality and cannot always be anticipated using conventional clinical predictors. The aim of our study was to evaluate the effectiveness of submandibular sonography to predict difficult airway.

**Patients and methods:** We conducted a prospective and analytic study in Mongi Slim teaching Hospital. We analyzed 200 patients undergoing orthopedic or visceral surgery under general anesthesia with endotracheal intubation. They were categorized as having easy (grades I and II) or difficult laryngoscopy (DL: grades III or IV) based on the laryngoscopic criteria of Cormack-Lehane. Preoperatively, 4 sonographic parameters including tongue width (TW) and thickness, hyomental distance in neutral and extended position, and anterior neck soft tissue thickness at levels hyoide bone (HB) and thyrohyoid membrane (THM) were evaluated.

**Results:** DL and difficult mask ventilation (DMV) were found in 18% and 12% of cases. Anterior neck soft tissues at the level of HB and THM were the best sonographic parameters significantly associated with DL. Areas under the ROC curves (AUROC) were respectively at 0.813 and 0.928 (p < 10^−3^). In multivariate analysis, a modified Mallampati score > 2 (OR = 5) and soft tissue thickness at THM ≥ 2 cm (OR = 70) were independently associated with DL. A new predicting score was defined (AUROC = 0.885; p < 10^−3^). A value > 2 showed a sensitivity of 94% and a specificity of 74%. For ultrasonography predicting DMV, TW and neck soft tissue at HB showed the best AUROC (0.861, 0.889 and 0.914; p < 10^−3^). Clinical and sonographic parameters independently associated with DMV were: neck circumference≥40 cm (OR = 7), body mass index ≥ 30 kg/m^2^ (OR = 4), TW > 5.1 cm (OR = 15) and soft tissue thickness at HB > 0.98 cm (OR = 5). A derived score > 20 showed the best sensitivity and specificity (92% and 81% respectively). Combined models consisting of sonographic and clinical tests improved the diagnostic value of difficult airway management.

**Conclusion:** We suggest to consider submandibular sonographic examination as a valuable tool to enrich a multimodal preoperative airway assessment.

**Compliance with ethics regulations:** Yes.

### P-183 Transcutaneous PCO_2_ monitoring during intubation in critically ill patients : descriptive study and assessment of the link between PCO_2_ variations and postintubation hypotension

#### Aurélien Frérou, Adel Maamar, Sonia Rafi, Claire Lhommet, Pierre Phelouzat, Emmanuel Pontis, Florian Reizine, Mathieu Lesouhaitier, Christophe Camus, Yves Le Tulzo, Jean-Marc Tadié, Arnaud Gacouin

##### CHU Rennes, Rennes, France

###### **Correspondence:** Aurélien Frérou (aurelien.frerou@chu-rennes.fr)

*Ann. Intensive Care* 2020, **10(Suppl 1):**P-183

**Rationale:** Reducing the risk of severe hypoxemia during endotracheal-intubation (ETI) is a major concern in intensive care unit but little attention was paid to CO_2_ variations during this period. We conducted a prospective observational study to describe transcutaneous CO_2_ (PtcCO_2_) throughout intubation in patients who received preoxygenation with standard oxygen therapy (SOT), non-invasive ventilation (NIV), or high flow nasal cannula oxygen therapy (HFNCOT).

**Patients and methods:** Patients over 18 years undergoing ETI in ICU were continuously monitored for PtcCO_2_ during intubation and the following 3 h under mechanical ventilation (MV). Haemodynamics and respiratory parameters were also recorded as well as arterial partial pressure of CO_2_ (PaCO_2_) to evaluate reliability of the transcutaneous measure.

**Results:** Two hundred and two patients were included in the study. We found a strong correlation between PtcCO_2_ recorded at preoxygenation and the last PaCO_2_ available before intubation (r = 0.87, p < 0.0001). In 75% of patients PtcCO_2_ values recorded at initiation of MV were out of 35–45 mmHg ranges. PtcCO_2_ recorded at ETI, at initiation of MV, 30 min and 1 h of MV were significantly higher than PtcCO_2_ during preoxygenation (p < 0.05 by ANOVA). Variations of PtcCO_2_ were significantly different according to the preoxygenation method (p < 0.001 for interaction in ANOVA). Lastly, a decrease in PtcCO_2_ higher than 5 mmHg within half an hour after the beginning of MV was independently associated with postintubation hypotension (PIH) (Odds Ratio = 2.14, 95% Confident Interval 1.03–4.44, p = 0.04).

**Conclusion:** PtcCO_2_ is a valuable tool to record PaCO_2_ variation in patients requiring invasive mechanical ventilation and could be useful to prevent PIH.

**Compliance with ethics regulations:** Yes.

**Fig. 1:**
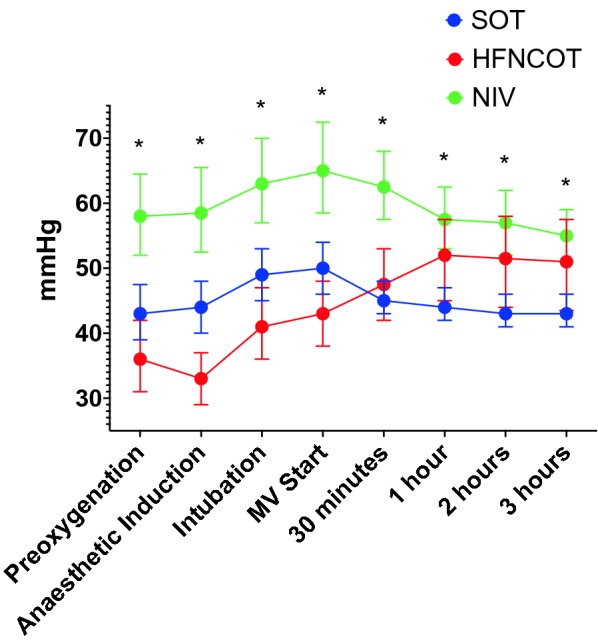
Repeated measures of transcutaneous PCO_2_ (PtcCO_2_) before and after the beginning of the mechanical ventilation (MV) according to the preoxygenation method. SOT: Standard oxygen therapy; HFNCOT: High flow nasal cannula oxygen therapy; NIV: Non invasive ventilation. *p < 0.05

### P-184 National survey of intubation practice in French intensive care units

#### Maelle Martin, Paul Decamps, Jean Reignier, Jean-Baptiste Lascarrou

##### Medecine Intensive Reanimation, CHU Nantes, Nantes, France

###### **Correspondence:** Maelle Martin (maelle.martin@chu-nantes.fr)

*Ann. Intensive Care* 2020, **10(Suppl 1):**P-184

**Rationale:** Intubation in intensive care unit (ICU) is a critical procedure which leads to serious adverse event in 20 to 40% of cases. Several recent trials were conducted to help physicians to choose medications, devices and modality of intubation. Especially, videolaryngoscope (VL) led to several publications in the last few years, with increasing tools marketed and spread use (difficult airway management, routine intubation). We designed an online survey to take a picture of intubation process and devices availability in France.

**Patients and methods:** Prospective, online national survey. Anonymous questionnaire with 26 questions emailed to physicians working in intensive care units in France (one physician per ICU: head or intubation specialist).

**Results:** One hundred and eighty intensivists from 180 different ICUs answered the survey (response rate 180/257: 70%). 43% of French ICUs operators are qualified as non-expert physicians for intubation, among 18.8% of them stay untrained or trained with basic learning process (theory and patient’s room learning). 59.7% access to low fidelity simulation and 34.5% to highest fidelity simulation. 94.4% of participating ICU reports owning a difficult intubation trolley, 74.5% an intubation protocol, 92.2% a capnography with 69.3% systematic use for endotracheal intubation checking, 91.6% a laryngeal mask and 97.2% a front of neck access. Out of predictable difficult intubation, Macintosh laryngoscopy (alone) stays for 83.3% the first line way to intubate in ICU. In case of difficulty for trachea catheterization, 85.6% associated a bougie or 7.8% switched for videolaryngoscopy. 76.6% of participating ICU have a videolaryngoscope, which is statistically associated with owning an intubation protocol (P = 0.043) or using a capnography for intubation (P = 0.02). VL has been acquired during the past 5 years for 67.4% of ICU. The French ICU videolaryngoscope market is dominated by Airtraq^®^ (39.3%), McGrath^®^Mac (36.9%) and Glidescope^®^ (14.5%). 84% of ICU restricted their videolaryngoscopy use for difficult intubation.

**Conclusion:** Nearly half of intensivists are considered as non-expert for airway management with an insufficient access to modern training such as simulation. There is a large implementation of videolaryngoscopy on ICU setting, with however, some use restriction for difficult intubation situation.

**Compliance with ethics regulations:** Yes.

**Fig. 1:**
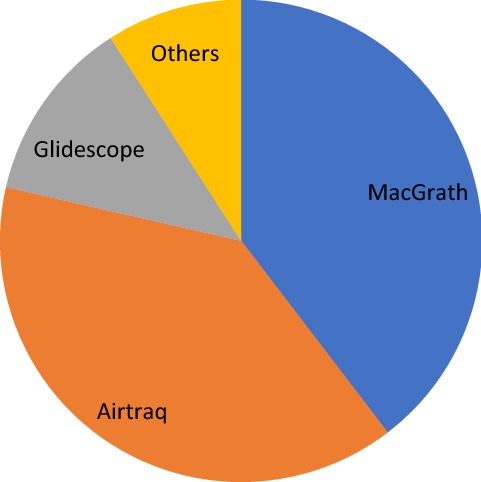
Distribution of videolaryngoscope in French ICUs

### P-185 Impact of videolaryngoscopy expertise on intubation first attempt success in critically ills

#### Matthieu Amalric^1^, Romaric Larcher^1^, Vincent Brunot^1^, Fanny Garnier^1^, Audrey De Jong^2^, Laura Platon^1^, Noémie Besnard^1^, Delphine Daubin^1^, Valérie Moulaire Rigollet^1^, Philippe Corne^1^, Kada Klouche^1^, Boris Jung^1^

##### ^1^Medical Intensive Care Unit, Montpellier University and Montpellier University Health Care Center, Montpellier, France; ^2^Saint Eloi Department of Anesthesiology and Critical Care Medicine, Montpellier University and Montpellier University Health Care Center, Montpellier, France

###### **Correspondence:** Matthieu Amalric (amalric.matthieu@gmail.com)

*Ann. Intensive Care* 2020, **10(Suppl 1):**P-185

**Rationale:** The use of a videolaryngoscope (VL) in the Intensive Care Unit (ICU) on the first endotracheal intubation attempt success rate and intubation related complications is controverted. The objective of this study was to evaluate the first intubation attempt success rate with the McGrath™ MAC VL according to the operators’ VL expertise (defined as the number of videolaryngoscopies already performed by the operators).

**Patients and methods:** As a part of a quality improvement initiative, we implemented the McGrath™ MAC VL in our ICU difficult airway toolbox. It was positioned as a first line laryngoscope for every intubation in critically ill patients to reinforce the VL skill training. Present study was performed using prospectively collected data from a continuous quality improvement database about airway management in a 20-beds French teaching hospital medical ICU. All consecutive intubation procedure performed with VL from September 2018 to June 2019 were included. “First attempt success” group and “first attempt failure” group were compared by univariate and multivariate analysis in order to analyze the first attempt intubation success rate according to the level of operators’ expertise, identify factors associated with first pass intubation failure and describe the intubation related complications.

**Results:** We enrolled 202 consecutive endotracheal intubations. Overall first attempt success rate was 126 (62%). Comorbidities, junior operator, the presence of cardiac arrest and coma were associated with a lower first attempt success rate. The first attempt success rate was less than 50% in novice operators (1-5 previous experiences with VL, independently of airway expertise with direct laryngoscopies) and 87% in expert operators (greater than 15 previous experiences with VL) (Fig. 1). Multivariate analysis confirmed the association between specific skill training with the VL and the first attempt success rate. Severe hypoxemia and overall immediate intubation related complications occurred more frequently in first attempt failure intubations (24/76, 32%) than in first attempt success intubations (14/126, 11%), p < 0.001.

**Conclusion:** We report for the first time in the critically ill that specific videolaryngoscopy skill training, assessed by the number of previous videolaryngoscopies performed, is an independent factor of first attempt intubation success. Also, we observed that specific skill training of the McGrath™ MAC VL was fast. Therefore, future trials evaluating videolaryngoscopy in ICUs should take into account the specific skill training of the operators in videolaryngoscopy.

**Compliance with ethics regulations:** Yes.

**Fig. 1:**
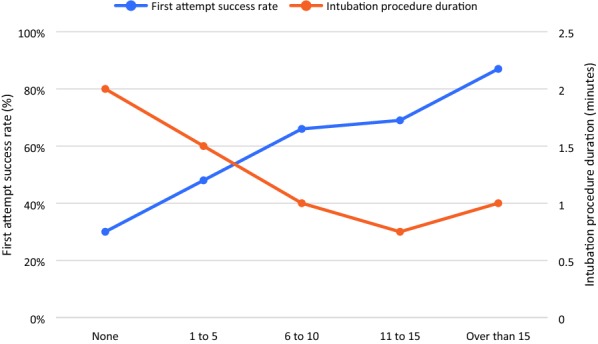
First attempt success rate and intubation procedure duration according to previous experience in videolaryngoscopy

### P-186 Impact of tracheostomy timing on prognosis in intensive care unit

#### Hatem Ghadhoune^1^, Jihene Guissouma^1^, Sourour Belhaj Youssef ^2^

##### ^1^Hospital of Bizerte, University Elmanar Tunis, Faculty of Medicine Tunis, Bizerte, Tunisia; ^2^Faculty of medecine Monastir, Monastir, Tunisia

###### **Correspondence:** Hatem Ghadhoune (ghadhoune@yahoo.fr)

*Ann. Intensive Care* 2020, **10(Suppl 1):**P-186

**Rationale:** Tracheostomy in intensive care unit (ICU) has many advantages. But only patient comfort and shorter ICU and hospital stay were demonstrated. The timing of this procedure is still debated. The aim of this study was to determine the impact of early tracheostomy on prognosis.

**Patients and methods:** We performed a retrospective study in a medical ICU (6 beds unit) from January 2013 to November 2018. The technique of tracheostomy was exclusively surgical in the operating room made by the surgeon. The primary endpoint was mortality in ICU. The secondary outcomes were post-tracheostomy incidence of ventilator acquired pneumonia, duration of mechanical ventilation and length of stay in ICU. These criteria were assessed in relation to timing of the tracheostomy defined as early when performed before day 10 of mechanical ventilation.

**Results:** Forty-two patients were enrolled during the study period. Mean age of patients was 55 ± 19 years. Median length of stay in ICU was of 49 days. Mortality rate was of 60%. Comparing the two groups, early vs late tracheostomy, no difference was found with respect to mortality (62% vs. 59%, p = 0.84), VAP occurrence (12% vs. 35%, p = 0.21), post-tracheostomy duration of mechanical ventilation (52 ± 81d vs. 28 ± 31d, p = 0.17), or length of stay in ICU (64 ± 78d vs. 61 ± 40d, p = 0.84). In multivariate analysis, the only factor independently related to mortality was the SOFA score patient on tracheostomy day with p = 0.041 and OR = 1.88 (CI95% [1.023–3.464]).

**Conclusion:** Tracheostomy in the intensive care unit remains a justified alternative despite the discordant data in the literature. In our study, the delay of the procedure didn’t interfere with the evolution. However, the patient severity as attested by SOFA score at the day of tracheostomy, was the only independent prognostic factor. Those results should be confirmed by other large prospective studies.

**Compliance with ethics regulations:** Not applicable.

### P-187 Tracheotomy in intensive care

#### Sabah Benhamza, Mohamed Lazraq, Youssef Miloudi, Abdelhak Bensaid, Najib El Harrar

##### Réanimation de l’Hôpital du 20 Août, Casablanca, Morocco

###### **Correspondence:** Sabah Benhamza (benhamzasabah5@gmail.com)

*Ann. Intensive Care* 2020, **10(Suppl 1):**P-187

**Rationale:** Many unknowns remain as to the place of tracheostomy in intensive care. Reluctance to perform a tracheotomy is numerous, especially when pre-exists chronic respiratory failure, but some data suggest benefits. We report in this work our experience in tracheotomy in the intensive care unit of the 20 August hospital, Casablanca.

**Patients and methods:** This is a retrospective descreptive study over 2 years (January 2016 to January 2018) including all patients that have been tracheostomized in the intensive care unit of the 20 August hospital 1953.

**Results:** During the study period, 50 patients were tracheostomized with a prevalence of 9.4% in 2 years, the predominance was male (sex ratio 2.3). The average age was 44 ± 18 years old. The indication for tracheostomy was prolonged ventilation in 76% of cases, extubation failure in 20% of cases, and intubation failure in 8% of cases. Tracheostomy was performed on average on the 12th day of intubation. All patients were tracheostomized in the operating room by ENT surgeons. The main complications attributable to tracheotomy were hemorrhage of the tracheostomy orifice in 2 patients (4%) immediately resumed, 2 cases of subcutaneous emphysema (4%), 1 case of pneumothorax (2%), 3 cases of orifice infection (6%). No patient died of a tracheostomy related cause.

**Conclusion:** The tracheotomy in intensive care is still a subject of debate especially concerning the time of its realization. However it seems to reduce the duration of mechanical ventilation, facilitates the care and also the ventilatory weaning.

**Compliance with ethics regulations:** Yes.

### P-188 High flow nasal cannula oxygenation (HFNCO) effects assessed by electric impedance tomography: a physiologic observation in healthy subjects

#### Charlotte Porte, Geoffroy Hariri, Jean-Rémi Lavillegrand, Naike Bige, Jean-Luc Baudel, Hafid Ait-Oufella, Eric Maury

##### Hôpital Saint-Antoine, Paris, France

###### **Correspondence:** Charlotte Porte (charlotte.porte@hotmail.fr)

*Ann. Intensive Care* 2020, **10(Suppl 1):**P-188

**Rationale:** HFNCO is a frequently used device providing heated and humidified high flow oxygen with several advantages: decreased work of breathing, decreased dead space, increased end expiratory lung volume (EELV), more stable FiO_2_. The increase in EELV is relying of the positive expiratory effect generated by the device. The level of generated PEP seems however to largely depend on whether the mouth is open or not. This study was aimed to assess the impact of mouth opening on EELV increase induced by HFNCO using electric impedance tomography.

**Patients and methods:** The following HFNCO trial was proposed to healthy subjects who used HFNCO on a regular basis for patients care. Oxygen flow was set successively during 10 min periods at 0, 35 and 70 L/min (OptiflowTM; Fisher & Paykel Healthcare, Auckland, NZ). These three conditions were tested in semi recumbent and supine position chosen at random. Measurement started in supine position with no flow (baseline) and each period was separated from the following by a wash out period on 2 min during which the subject could breath normally with no supplemental oxygen. Electric Impedance Tomography (Pulmovista^®^ 500, Dräger Medical GmbH, Lündbeck, Germany) was performed applying a 16 electrodes belt placed between the 4th and 5th intercostal space, including a reference electrode located on the abdomen. As no spirometer was used, the data of EELV computed on the EIT device were expressed as percentage of variation of the value measured in supine or semi recumbent position with no flow. Demographic data were expressed as median and extreme values. Comparisons were performed using U Mann Whitney test.

**Results:** 21 healthy subjects (12 women, age 28 years [20–43], weight 68 kgs [55–80], height 171 cm [164–183] BMI 22.6[19.4–29.6] accepted to participate to the study. When subjects received HFNCO with open mouth (whatever position) no modification of EELV was observed (Table 1). Conversely, a significant increase in EELV was noted with closed mouth, whatever position. In the semi recumbent position the increase in EELV was even more important with 70L/min.

**Conclusion:** Electrical impedance tomography illustrates the impact of mouth closure on EELV increase among healthy subjects receiving HFNCO.

**Compliance with ethics regulations:** Yes.

**Table 1:** Variation in EELV according to mouth closure and position

	0L/min	35L/min	70L/min
Supine	100		
Open mouth		+ 1.2	− 0.6
Closed mouth		+ 12.5*	+ 18 *
Semi recumbent	+7		
Open mouth		+ 2.1	− 3.8
Closed mouth		+ 9.3*	+ 25*^$^

EELV values are expressed as percentages of variation of EELV measured in supine position with no flow

* p < .05 for comparison between open and closed mouth in same position with similar flow

^$^p < 0.5 for comparison between 35L/min and 70L/min in same position

### P-189 Nasal high flow oxygen can be used in hypercapnic respiratory failure due to COPD exacerbation without increase in PaCO_2_

#### Lise Piquilloud^1^, Francois Beloncle^2^, Pierre-Yves Olivier^2^, Alain Mercat^2^

##### ^1^Adult Intensive Care and Burn Unit, University Hospital and University of Lausanne, Lausanne, Switzerland; ^2^Medical Intensive Care Department, University Hospital of Angers, Angers, France

###### **Correspondence:** Lise Piquilloud (lise.piquilloud@chuv.ch)

*Ann. Intensive Care* 2020, **10(Suppl 1):**P-189

**Rationale:** In stable COPD patients, nasal high flow oxygen (NHF) use can be associated with reduction in respiratory rate (RR) and minute ventilation (MV). In these patients, PaCO_2_ remains stable or decreases under NHF. This suggests a possible dead space reduction related to a washout effect of NHF. The aim of this study was to assess the physiological effects of NHF in hypercapnic patients with acute COPD exacerbation.

**Patients and methods:** Crossover study in hypercapnic patients suffering from acute COPD exacerbation and treated with intermittent non-invasive ventilation (NIV). NHF 40L/min or standard oxygenotherapy (STAND O2) were randomly administered during 1 h between NIV treatments. RR, tidal volumes (VT), MV and corrected MV (corMV = MV x PaCO2/40) variations were recorded during the last 10 min of each study period using a respiratory inductive plethysmography vest. Blood gas analysis was performed at the end of each oxygen administration period. Visual analogic dyspnea score (VAS) quoted from 0 to 10 was assessed by the patient after 30 and 60 min. Results given as median [IQR]. Wilcoxon tests were used to compare data between STAND O2 and NHF.

**Results:** Twelve patients were included and data could be recorded in 10 (8 males/2 females, 63 [60–78] years old, SAPS II 30 [24–38]. Median PaCO_2_ at inclusion was 58 [54–66] mmHg. Respiratory rate was lower (22 [20–23] vs. 25 [23–27] breaths/minute, p = 0.049) during NHF than during STAND O_2_. PaCO_2_ was also lower during NHF (48.7 [46.4–58.1] vs. 50.7 [48.4–57.5] mmHg, p = 0.049). PaCO_2_ decreased during NHF in 9/10 patients. MV and corMV were lower in NHF compared to STAND O_2_ (Fig. 1). Dyspnea scores were not different between the 2 modalities.

**Conclusion:** In case of acute COPD exacerbation, using NHF between NIV treatments was associated with PaCO_2_ and RR decrease. MV concomitantly decreased suggesting a deadspace volume reduction related to a washout effect of NHF. Corrected MV decreased in all the patients except one. These results suggest that NHF could be used to deliver oxygen between NIV treatments to COPD patients suffering from acute exacerbation and could contribute reducing PaCO_2_.

**Compliance with ethics regulations:** Yes.

**Fig. 1:**
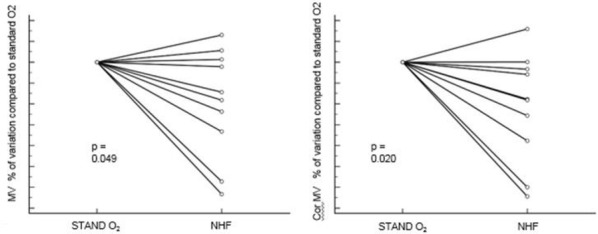
Minute ventilation (MV) and corrected minute ventilation (corMV) variations during standard oxygenotherapy (STANDO2) and nasal high flow oxygen (NHF)

### P-190 Atypical micro-organisms in acute exacerbation of chronic obstructive pulmonary disease: incidence and outcomes

#### Gharbi Rim, Sioud Imen, Oussama Jaoued, Sik Ali Habiba, Kheder Mohamed, Ben Haj Khalifa Anis, Fekih Hassen Mohamed, Elatrous Souheil

##### ^1^Hôpital Taher Sfar, Mahdia, Tunisia

###### **Correspondence:** Gharbi Rim (rimgharbi2008@gmail.com)

*Ann. Intensive Care* 2020, **10(Suppl 1):**P-190

**Rationale:** The role of atypical micro-organisms in acute exacerbation of chronic obstructive pulmonary disease (COPD) that require mechanical ventilation is poorly none. The aim of this study was to determine the role of atypical pathogens in severe acute exacerbation of COPD.

**Patients and methods:** In this prospective study we included all patients admitted for acute exacerbation of COPD requiring mechanical ventilation. Atypical pathogens (chlamydophila pneumoniae and mycoplasma pneumoniae) were searched by serological diagnosis and by culture of sputum samples.

**Results:** In this study we included 58 patients aged 65 ± 13 years. Sixty-eight percent of sputum culture were considered significant. Six cultures were positive with 6 different microorganisms. Neither chlamydophila pneumoniae nor mycoplasma pneumoniae were found. The prevalence of chlamydophila pneumoniae was 56.9% (positive IgG serum). The demographic characteristics was similar between patients with and without positive culture. The rate of noninvasive ventilation (NIV) failure was 18% in positive serology group versus 44% in negative serology group (p = 0.032). The mortality was similar in both groups. In multivariate logistic regression analysis only positive serology (OR = 4.326; 95% IC [1.110–16.790], p = 0.034) was an independent factor of NIV failure.

**Conclusion:** A positive serology of chlamydophila pneumoniae was a predictive factor of NIV failure without an impact on the morbidity and mortality of COPD patient treated with mechanical ventilation.

**Compliance with ethics regulations:** Yes.

### P-191 Non-invasive ventilation use at Emergency Department during acute exacerbations of chronic obstructive pulmonary disease: Clinical outcomes and predictors of failure

#### Hadil Mhadhbi, Yosra Yahya, Khedija Zaouche, Abdelwahab Mghirbi, Kamel Majed

##### Emergency Department la Rabta, Tunis, Tunisia

###### **Correspondence:** Hadil Mhadhbi (hadil.mhadhbi@gmail.com)

*Ann. Intensive Care* 2020, **10(Suppl 1):**P-191

**Rationale:** Emergency departments (ED) receive a growing up number of patients with acute exacerbation of chronic obstructive pulmonary disease (COPD) .Non-invasive ventilation (NIV) could be a good alternative to achieve a respiratory support, avoiding as much as possible the complications of invasive ventilation. The study aimed to assess the clinical outcomes of using NIV in acute exacerbation of COPD at ED and to identify whether clinical variables present at admission are predictive of NIV failure.

**Patients and methods:** We conducted a prospective study conducted at the ED over a period of one year. Data of all patients admitted for acute exacerbation of COPD for all causes and requiring non-invasive ventilation were collected. NIV failure was defined as need for endotracheal intubation or death.

**Results:** During the study period, a total of 70 patients with a mean age of 65 years (± 12) were included. Acute exacerbation of COPD was due to bronchitis in 46%, to pneumonia in 30% of cases. 18% of patients had no apparent etiology of acute exacerbation of COPD. Bilevel Positive Airway Pressure was performed on all patients, during a mean period of 10 h (± 6). Clinical NIV success was observed in 50 patients (71%). The predictors of NIV failure were advanced age, tachycardia, and hypercapnia.

**Conclusion:** The efficiency of NIV in the management of acute exacerbations of COPD at ED is well documented. This is further supported by our study which showed a clinical success in 71% of patients with acute exacerbation of COPD.

**Compliance with ethics regulations:** Yes.

### P-192 Predictive factors of non-invasive ventilation failure in elderly patients with acute respiratory failure at Emergency Department

#### Yosra Yahya, Abdelwahab Mghirbi, Khedija Zaouche, Ramla Baccouche, Hadil Mhadhbi, Nadia Zaouak, Hamida Maghraoui, Kamel Majed

##### Emergency Department of La Rabta teaching Hospital, Tunis, Tunisia

###### **Correspondence:** Yosra Yahya (yosrayahia@gmail.com)

*Ann. Intensive Care* 2020, **10(Suppl 1):**P-192

**Rationale:** Non invasive ventilation (NIV) is often performed in elderly patients with acute respiratory failure (ARF) at Emergency Department (ED). This technique may be subject to many difficulties, due to the presence of frequent co-morbidities. The aim of this study was to identify the predictive factors of NIV failure in elderly patients with ARF at ED.

**Patients and methods:** This was a retrospective study conducted at ED on 1 year and 4 months including patients aged more than 65 years and who required the use of NIV for an ARF. All data were collected and analyzed using the SPSS 22 software. Patients were divided into two groups: NIV failure and NIV success. NIV failure was defined by inhospital mortality, requirement of intubation or hospitalization at intensive care unit.

**Results:** During the study period, a total of 75 elderly patients that required NIV for ARF were included. Median age was 74 years (min = 65, max = 88) and sex ratio was 2.57. The median Charlson index was 5 (min = 3, max = 9). The etiological diagnoses of ARF were acute decompensation of chronic obstructive pulmonary disease (72%), acute heart failure (61%), pneumonia (37%) and pulmonary embolism (1%). The ARF was hypercapnic in 71% of cases and non-hypercapnic in 39%. NIV failure concerned 32%. Predictive factors of NIV failure were clinical signs of right heart dysfunction (p < 0.05), C reactive protein (p = 0.004), initial pH (p = 0.044) and kidney dysfunction (p < 0.05).

**Conclusion:** In our study, NIV failure in elderly patients with ARF at ED was influenced by clinical signs of right heart dysfunction, C reactive protein, initial pH and kidney dysfunction. These clinical and biological factors could be useful to identify the most critical elderly patients and to better guide therapeutic decisions.

**Compliance with ethics regulations:** Yes.

### P-193 ECCO2R as salvage therapy in acute severe asthma: insights from the great Paris area register

#### Jean-Loup Augy^1^, Nadia Aissaoui^1^, Yves Cohen^2^, Muriel Fartoukh^3^, Nicholas Heming^4^, Eric Maury^5^, Johanna Oziel^2^, Guillaume Voiriot^6^, Jean-Luc Diehl^1^

##### ^1^AP-HP, Hôpital Européen Georges Pompidou, Service de Médecine Intensive Réanimation, Paris, France; ^2^AP-HP, Hôpital Avicenne, Service de Réanimation Polyvalente, Bobigny, France; ^3^AP-HP, Hôpital Tenon, Service de Réanimation Polyvalente, Paris, France; ^4^AP-HP, Hôpital Raymond Poincaré, Service de Réanimation Polyvalente, Garches, France; ^5^AP-HP, Hôpital Saint-Antoine, Service de Médecine Intensive Réanimation, Paris, France; ^6^AP-HP, Hôpital Tenon, Service de Réanimation Polyvalente, Paris, France

###### **Correspondence:** Jean-Loup Augy (augyjeanloup@hotmail.com)

*Ann. Intensive Care* 2020, **10(Suppl 1):**P-193

**Rationale:** The interest of ECCO2R in the management of very severe acute asthma exacerbations is still unclear. Since it could help to control respiratory acidosis and /or to limit dynamic hyperinflation, its clinical benefits are uncertain, even in mechanically ventilated patients.

**Patients and methods:** The REXECOR observatory is a prospective ECCO2R cohort in the great Paris area. Ten cases of severe asthma treated by ECCO2R were retrospectively reviewed. Mainly, arterial blood gases (ABG), duration of ECCO2R and IMV were collected and in-ICU mortality were assessed. Data are reported as median (IQR).

**Results:** Ten patients (7 men, age: 58 (IC: 41–61) years, BMI: 24.7 (IC: 19.3–25.9) kg/m^2^, FEV-1: 1.76 (IC: 1.27–2.57) L, (68 (IC: 60–73) %), SAPS2: 26.5 (IC: 23.5–39.7) points) were included. One patient suffered from cardiac arrest before admission and one had pneumothorax at ICU admission. Nine patients were under IMV (started on the day of admission for 8). Before ECCO2R, 10 patients received systemic corticosteroids, 8 paralyzing agents, 1 epinephrine and 10 salbutamol. Two patients suffered from pneumonia. ECCO2R was started 2 (IC: 1–3.75) days after intubation. Venous vascular access was achieved via the right internal jugular route in 8 patients and via the femoral route in 2. The Hemolung device was used in 4 patients, the iLa Activve in 5 and the Prismalung in 1. ABG before and after 1 day of ECCO2R are reported in Table 1. Duration of ECCO2R was 8 (IC: 5.25–11) days and 3 patients were weaned from IMV under ECCO2R. For the remaining 6 patients, duration of IMV after ECCO2R was 21 (IC: 6–34.5) days. ICU stay was 28.5 (IC: 15–48.75) days. The only one NIV patient was not intubated. ECCO2R as stopped in 3 patients because of complications (one hemolysis, one internal bleeding and one membrane clotting). One patient died in ICU after limitation of life-sustaining therapy decision.

**Conclusion:** We report a preferential use of ECCO2R in IMV patients, contrasting with a marginal use in only one NIV patient to prevent intubation. The mortality rate was low, in line with previous case series of severe acute asthma with ECMO or ECCO2R support. More studies are needed (1) to better delineate the pathophysiological benefits of ECCO2R in asthma patients and (2) to confirm strong clinical benefits.

**Compliance with ethics regulations:** Not applicable.

**Table 1:** Evolution after 1 day of treatment by ECCO_2_R

	Before ECCO_2_R	One day after ECCO_2_R initiation	p
pH PaCO_2_ (mmHg) PaO_2_ (mmHg)	7.26 (IC: 7.20–7.27) 68.5 (IC: 63–71.5) 73 (IC: 68–94)	7.4 (IC: 7.37–7.41) 47.5 (IC: 45–59) 82 (IC: 58.5–102)	0.009 0.30 1

Results are expressed in median (interquartile). Comparisons of continuous variables before and after ECCO_2_R were performed using the Wilcoxon signed rank test

ECCO_2_R : extracorporeal CO_2_ removal

### P-194 Acute exacerbations of chronic obstructive pulmonary disease requiring invasive ventilation: factors predicting mortality

#### Jihene Guissouma, Hatem Ghadhoune, Habib Brahmi, Sourour Belhaj Youssef, Meriem Ksouri, Hana Benali, Yesmine Garbaa

##### Hôpital Bougatfa, Bizerte, Tunisia

###### **Correspondence:** Jihene Guissouma (bahri.jihene@yahoo.fr)

*Ann. Intensive Care* 2020, **10(Suppl 1):**P-194

**Rationale:** Acute exacerbations of chronic obstructive pulmonary disease (AECOPD) are the most important events characterizing respiratory illness progression. Their management often needs noninvasive or invasive ventilation (IV). Data of literature confirm that the mortality of AECOPD requiring IV is high but are discordant about prognostic factors. The aim of our study was to describe the epidemiologic and clinical features of patients admitted for AECOPD requiring IV, the treatment and the evolution in intensive care unit in order to deduce the independent factors of mortality.

**Patients and methods:** A 4-year retrospective analytic observational single-center study including patients hospitalized for AECOPD requiring IV.

**Results:** Fifty-eight patients were enrolled. Mean age was 68 ± 9 years with sex-ratio of 4.8. Eighty one percent were smokers and 55% were classified GOLD stage 3. History of intensive care hospitalization and prior IV were found in 43% and 34% of all cases respectively. Mean APACHE II score was 21 ± 9. The predominant precipitating factor for AECOPD was respiratory tract infection (65% of all cases). Twenty two percent of all patients presented septic shock. IV was initiated on admission in 40% of all cases and after noninvasive ventilation failure in 60% of all cases. Forty-eight per cent of all patients developed septic shock as evolutionary complication. Mortality rate was 62%. In univariate analysis: male gender (p = 0.022), duration of respiratory disease progression (p = 0.04), annual exacerbations frequency (p < 10^−3^), GOLD 3 stage (p = 0.008), prior IV (p < 10^−3^), duration of symptoms before hospitalization (p = 0.03), APACHE II score (p = 0.04), pH (p = 0.004), shock on admission (p = 0.01) and septic shock as evolutionary complication (p = 0.01) were predictors of mortality in our study. Besides; shock on admission (p = 0.001) and as evolutionary complication (p = 0.017) were the two independent prognostic factors in multivariate analysis.

**Conclusion:** Vital and functional prognosis of AECOPD requiring IV depends on the severity of the underlying respiratory illness, the severity of the exacerbation and the quality of an early management. This emphasizes the importance of controlling modifiable risk factors including smoking cessation, basic treatment improvement and early appropriate treatment of these exacerbations.

**Compliance with ethics regulations:** Yes.

### P-195 Infections due to hypermucoviscous *Klebsiella pneumoniae* in intensive care : clinical présentation in New Calédonia, 2017–2019

#### Pierre Tailpied^1^, Julien Colot^2^, Mathieu Série^2^

##### ^1^CHT, Noumea, France; ^2^Institut Pasteur de Nouvelle Calédonie, Nouméa, France

###### **Correspondence:** Pierre Tailpied (pierre.tailpied@gmail.com)

*Ann. Intensive Care* 2020, **10(Suppl 1):**P-195

**Rationale:** Since 1990’s, a particular phenotype of *Klebsiella pneumoniae* has emerged in Asia and now spreads to America and Europe. This hypermucoviscous phenotype is characterized by an increase of capsule production. Hypermucoviscous *Klebsiella pneumoniae* (HmKP) strains are responsible for complicated bacteremia with multiple septic sites (liver, central nervous system, muscles). In New Caledonia about one third of *Klebsiella pneumoniae* have hypermucoviscous capsules. The aim of this study was to describe intensive care patients with hypermucoviscous klebsiella pneumoniae infection in New Caledonia.

**Patients and methods:** In an observational retrospective study we have successively included all intensive care patients with documented HmKP infections from November 2017 to August 2019 at the only tertiary medical center of New Caledonia. The hypermucoviscous strains was defined by a positive string test results. We also analyzed risk factors for mortality. This study was approved by local ethic committee.

**Results:** Between November 2017 and August 2019, 163 patients were hospitalized for Klebsiella pneumoniae infections. Among this patients, hypermucoviscous phenotype was found in 96 patients (58.8%) and 33 of these patients (33.3%) required intensive care. The lasted were 16 males (48%) and a majority (72%) were younger than 65 years of age. In intensive care patients, only 6 (18.8%) had nosocomial infection, majority were community acquired infections (81.2%) with 16 (48%) pneumoniae, 4 (12.5%) profound abscess, 2 pyelonephritis (6.2%), 1 (3%) meningitidis. 27 patients (87%) required mechanical ventilation for 8 days (95% CI 4–12), length of stay in ICU was 15 days (95% CI 7–22) and mortality rate was 55%.

**Conclusion:** HmKP infections lead young patients in intensive care unit in one third of case with a majority of pneumoniae requiring mechanical ventilation and with a high rate of mortality. Furthers studies are needed to investigate the role of this particular strain in severity.

**Compliance with ethics regulations:** Yes.

### P-196 Infectious complications following snakebite by bothrops lanceolatus in martinique: a retrospective case series

#### Dabor Resiere^1^, Hossein Mehdaoui^1^, André Cabie^2^, Remi Neviere^1^, Bruno Mégarbane^3^, Hatem Kallel^4^

##### ^1^Department of Critical Care, University Hospital of Martinique, Fort-de-France, France; ^2^Department of Infectious Diseases, University Hospital of Martinique, INSERM CIC 1424, Antilles University EA4537, F-97200, Fort-de-France, France; ^3^Department of Medical and Toxicological Critical Care, Lariboisière Hospital, Paris-Diderot University, INSERM UMRS1144, Paris, France; ^4^Intensive Care Unit, Cayenne General Hospital, Cayenne, 97300 French Guiana, France, Cayenne, GUYANA

###### **Correspondence:** Dabor Resiere (dabor.resiere@chu-martinique.fr)

*Ann. Intensive Care* 2020, **10(Suppl 1):**P-196

**Rationale:** Infections secondary to snakebite occur in a number of patients, and are potentially life-threatening. Bothrops lanceolatus bites in Martinique average thirty cases per year and may result in severe thrombotic and infectious complications. We aimed to investigate the infectious complications related to Bothrops lanceolatus bite.

**Patients and methods:** A retrospective single-center observational study over seven years (2011-2018) was carried out, including all patients admitted to the hospital due to Bothrops lanceolatus bite. Clinical and biological data were reported using the Dx Care, x-plore et cyberlab softwares of the Emergency Medicine and analyzed.

**Results:** One hundred and seventy snake-bitten patients (121 males and 49 females) were included. Thirty-nine patients (23%) presented grade 3 or 4 envenoming. Twenty patients (12%) developed wound infections. The isolated bacteria were Aeromonas hydrophila (3 cases), Morganella morganii (2 cases), group A Streptococuss, and group B Streptococcus (one case each). Patients were treated empirically with third-generation cephalosporin (or amoxicillin/clavulanate), aminoglycoside and metronidazole combinations. Outcome was favorable. The main factor significantly associated with the occurrence of infection following snakebite was the severity of envenoming (P < 0.05). Our findings clearly point towards the frequent onset of infectious complications in *B. lanceolatus*-bitten patients presenting with grade 3 and 4 envenoming.

**Conclusion:** Infectious bite-related complications of *Bothrops lanceolatus* account for approximately 10% of the cases, with a strong predominance for grade III and IV. Thus, based on the bacteria identified in the wounds; we suggest that empiric antibiotic therapy including third-generation cephalosporin should be administered to those patients on hospital admission.

**Compliance with ethics regulations:** Yes.

**Table 1:**
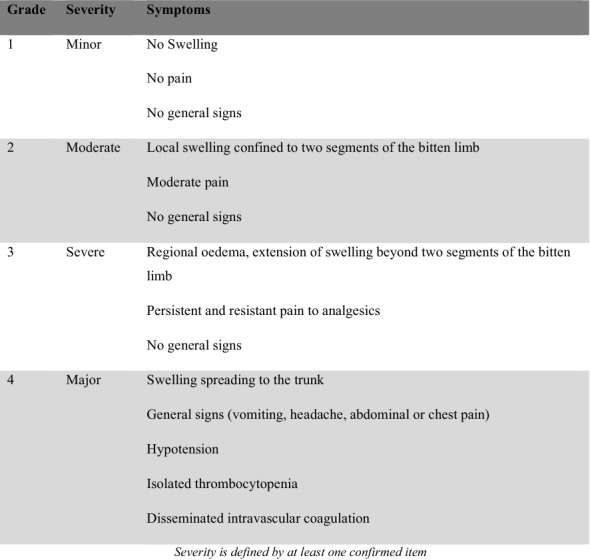
Severity score of envenoming after Bothrops lanceolatus bite

### P-197 Hemophagocytic lymphohistiocytosis secondary to sepsis: a monocentric retrospective study in medical intensive care unit; evolution comparison with a control cohort of sepsis

#### Diane Ducloux, Maximilien Grall, Steven Grange, Fabienne Tamion

##### CHU Rouen, Rouen, France

###### **Correspondence:** Diane Ducloux (diane.ducloux@gmail.com)

*Ann. Intensive Care* 2020, **10(Suppl 1):**P-197

**Rationale:** Hemophagocytic lymphohistiocytosis (HLH) is a rare, serious and usually deadly disease and the result of a secondary hyperinflammation to a dysregulation of the immune system. Secondary HLH to sepsis in intensive care unit are rarely studied in the literature and their treatments are not standardized. The goal of the study was to compare clinical and biological specifications and the change in patients in complicated or not septic shock of HLH.

**Patients and methods:** We realized an observational and retrospective study in medical intensive care unit in the CHU of Rouen between 2000 and 2015. This study compared every patient with a complicated septic shock of HLH according to the HScore (“HLH” group) to a comparison group (“Not HLH” group) paired with age and SOFA score. Medical background, biological parameters, death-rate and outcome of patients have been compared.

**Results:** In total, 37 patients have been included in the “HLH” population. Death-rate in intensive care unit was 59% in the “HLH” group compared to 51% in the “Not HLH” group (p = 0.48). We used more extrarenal cleansing in the “HLH” group (67% vs. 30%, p < 0.02), the duration of assisted ventilation was longer (17.5 days vs. 7.5 days, p < 0.02), as well as the duration of extrarenal cleansing (5.1 days vs. 1.6 days, p < 0.05) and those of amines (9.9 days vs. 4.5 days, p = 0.008). The average time of hospitalization was significantly longer in the “HLH” group (17.9 days vs. 9.17 days, p < 0.01).

**Conclusion:** The secondary HLH to sepsis in intensive care unit, not well known and understudied, seems to have a different profile and a more serious outcome but no change in death-rate has been found considering the pairing with the SOFA. Further studies are needed to plan a better therapeutic strategy within this population.

**Compliance with ethics regulations:** Not applicable.

### P-198 Serum and peritoneal exudate concentrations after high doses of ß-lactams in critically ill patients with severe intra-abdominal infections: an observational prospective study

#### Lisa Leon, Philippe Guerci, Elise Pape, Nathalie Thilly, Amandine Luc, Adeline Germain, Anne-Lise Butin-Druoton, Marie-Reine Losser, Julien Birckener, Julien Scala Bertola, Emmanuel Novy

##### CHRU Nancy, Vandoeuvre Les Nancy, France

###### **Correspondence:** Lisa Leon (lisaleon1307@gmail.com)

*Ann. Intensive Care* 2020, **10(Suppl 1):**P-198

**Rationale:** Critically ill patients with severe intra-abdominal infections (IAIs) requiring urgent surgery may undergo several pharmacokinetic alterations that can lead to ß-lactam under dosage. The aim of this study is to measure serum and peritoneal exudate concentrations of ß-lactams after high doses and optimal administration schemes.

**Patients and methods:** This observational prospective study included critically ill patients with suspicion of IAI who required surgery and a ß-lactam antibiotic as empirical therapy. Serum and peritoneal exudate concentrations were measured during surgery and after a 24 h steady-state period. The pharmacokinetic/pharmacodynamic (PK/PD) target was to obtain ß-lactam concentrations of 100% ƒT>4x MIC (Minimum Inhibitory Concentration) based on a worst-case scenario (highest ECOFF value) before bacterial documentation (a priori) and redefined on the MIC of the isolated bacteria (a posteriori).

**Results:** Forty-eight patients were included with a median [IQR] age of 64 [53-74] and a SAPS II score of 40 [31-65]. Septic shock occurred in 23% of cases. The main diagnosis was secondary nosocomial peritonitis. Piperacillin/tazobactam was the most administered ß-lactam antibiotic (75%). Prior to bacterial documentation, 16 patients (33.3%) achieved the a priori PK/PD target. IAI was documented in 34 patients (70%). Enterobacteriaceae were the most isolated bacteria. Based on the MIC (n = 23) of isolated bacteria, 78% of the patients achieved the PK/PD target (100% ƒT>4xMIC). In the Fig. 1 we presented serum ß-lactams PK/PD target attainment and observed total concentrations of Piperacillin-tazobactam at each timepoint in serum and peritoneal exudate.

**Conclusion:** In critically ill patients with severe IAIs, high doses of ß-lactams ensured 100% ƒT>4xMIC in 78% of critically ill patients with severe IAIs within the first 24 h. A personalized ß-lactam therapeutic scheme with a PK/PD target based on local ecology should be warranted.

**Compliance with ethics regulations:** Yes.

**Fig. 1:**
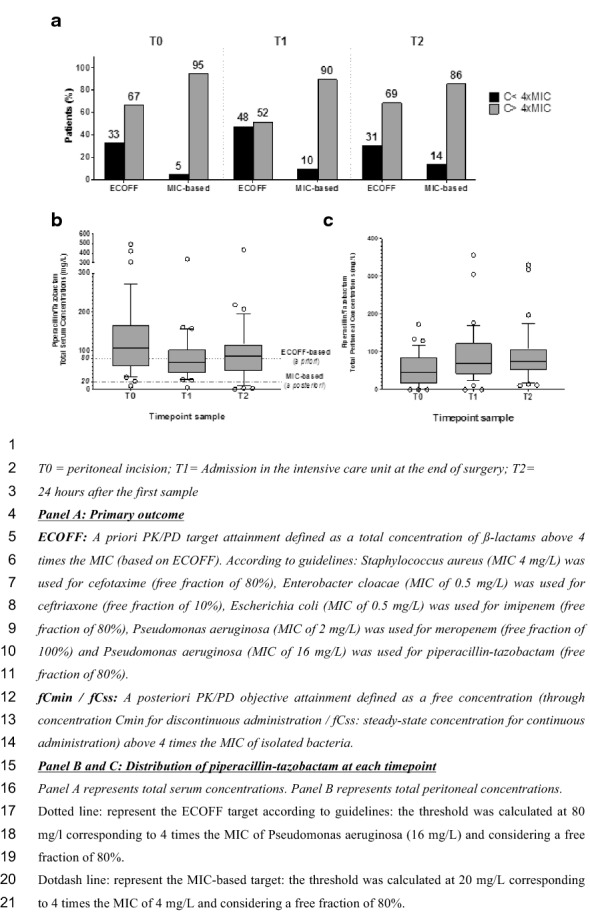
Serum ß-lactam PK/PD target attainment and observed total concentrations of Piperacillin-tazobactam at each timepoint

### P-199 Epidemiology and prognosis of intensive care unit acquired bacteremia in French Guiana

#### Hatem Kallel^1^, Roy Michaela^1^, Claire Mayence^1^, Cyrille Mathien^1^, Stephanie Houcke^1^, Dabor Resiere^2^, Hommel Didier^1^

##### CH Cayenne, Cayenne, France; ^2^CHU martinique, Fort De France, France

###### **Correspondence:** Hatem Kallel (hatem.kallel@ch-cayenne.fr)

*Ann. Intensive Care* 2020, **10(Suppl 1):**P-199

**Rationale:** Intensive care unit acquired bloodstream infections (ICU-BSI) are frequent, and associated with high morbidity and mortality rates. The objective of our study was to describe the epidemiology and the prognosis of ICU-BSI in our ICU (Cayenne General Hospital). Secondary objectives were to search for factors associated to ICU-BSI caused by ESBL-PE, and those associated with mortality at 28 days.

**Patients and methods:** We retrospectively studied ICU-BSI in the medical-surgical intensive care unit of the Cayenne General Hospital, during 78 months (January 2013 to June 2019). We assessed survival at 28 days from the diagnosis of ICU-BSI.

**Results:** ICU-BSI was diagnosed in 9.5% of admissions giving a density incidence of 10.3 ICU-BSI/1000 days. The median delay to the first positive bacteraemia was 9 days. The ICU-BSI was primitive in 65% of cases and secondary to ventilator acquired pneumonia in 15% of cases. One microorganism was isolated in 82.1% and two in 17.9% of cases. The main isolated microorganism were Enterobacteriaceae in 67.7% of patients. They were ESBL-producers in 27.6% of cases. Initial antibiotic therapy was appropriate in 65.1% of cases. Factors independently associated with ESBL-PE as the causative microorganism of ICU-BSI were ESBL-PE carriage prior to ICU-BSI (OR: 7.273; 95% CI 2.876–18.392; p < 0.000) and prior exposure to fluoroquinolones (OR: 4.327; 95% CI 1.120–16.728; p = 0.034). The sensitivity of ESBL-PE carriage to predict ESBL-PE as the causative microorganism of ICU-BSI was 64.9%, and specificity was 81.2%. Mortality at 28 days was 20.6% in the general population. In ESBL-PE carriers, it was 19.4% in ICU-BSI caused ESBL-PE and 14.1% in ICU-BSI caused by non ESBL-PE (p = ns). The median ICU length of stay was 26 days (15–49). It was 35 days (20–61) in ESBL-PE carriers, 38 days (20–60) in ESBL-PE carriers with ICU-BSI caused by ESBL-PE vs 35 days (21–64) in ESBL-PE carriers with ICU-BSI caused by non ESBL-PE (p = ns). In multivariable analysis, there was no parameter which was independently associated to mortality at day 28 from the occurrence of ICU-BSI.

**Conclusion:** ICU-BSI complicates 9.5% of admission to ICU and was associated with 25% in-hospital mortality. No associated prognosis factors were found probably due to multiple confounding factors.

**Compliance with ethics regulations:** Yes.

### P-200 Assessing and applying individualized treatment for group A streptococcal necrotizing soft-tissue infection is possible

#### Tomas Urbina^1^, Camille Hua^2^, Romain Bosc^3^, Françoise Tomberli^4^, Raphael Lepeule^5^, Jean-Winoc Decousser^6^, Armand Mekontso Dessap^1^, Olivier Chosidow^2^, Nicolas De Prost^1^

##### ^1^Service de réanimation médicale, Hôpital Henri Mondor, APHP, Paris, France; ^2^Service de Dermatologie, Hôpital Henri Mondor, APHP, Paris, France; ^3^Service de chirurgie plastique, Hôpital Henri Mondor, APHP, Paris, France; ^4^Service de réanimation chirurgicale, Hôpital Henri Mondor, APHP, Paris, France; ^5^Unité transverale de traitement des infections, Labratoire de bactériologie-virologie, Hôpital Henri Mondor, APHP, Paris, France; ^6^Labratoire de bactériologie-virologie, Hôpital Henri Mondor, APHP, Paris, France

###### **Correspondence:** Tomas Urbina (tomasurbina75@hotmail.com)

*Ann. Intensive Care* 2020, **10(Suppl 1):**P-200

**Rationale:** Necrotizing soft tissue infections (NSTI) are a heterogenous group of severe infections. Among them, group A streptococcal (GAS) infection represent a subgroup that could benefit from specific therapies targeting the toxinic pathway, such as intravenous immunoglobulins or clindamycin. Nevertheless, previous trials evaluating these treatments suffered from a low rate of GAS infection among the study population. Early identification of patients at high risk of GAS infection would allow for assessing targeted treatment strategies.

**Patients and methods:** We conducted a secondary analysis of a previously published cohort of 224 patients admitted to our tertiary center for surgically proven NSTI between 2006 and 2017. Admission characteristics and microbiological documentation based on surgical samples, blood cultures or subcutaneous puncture were recorded. We compared patients with a documented GAS infection to all other patients regarding admission characteristics. A generalized linear regression model was used to identify admission characteristics associated with a subsequent documentation of GAS infection.

**Results:** Among 224 patients, 60 (27%) had a GAS infection, which was monomicrobial in 39 (17%) cases. Admission characteristics associated with GAS infections by univariate analysis were NSAID treatment before admission (19 (31.7%) for GAS infections vs 27 (16.7%) for others, p = 0.004) and leukocytosis as a continuous variable (17,200 /mm^3^ [12,350–22,500] vs. 13,600 [9000–21,000], p = 0.016). Those inversely correlated with GAS infections were immunodeficiency (6 (10%) vs. 52 (31.7%), p = 0.002), and an abdominoperineal topography (1 (1.7%) vs. 37 (22.7%), p > 0.001). After multivariate analysis only immunodeficiency (OR = 0.29 [0.10–0.74], p = 0.015) and an abdominoperineal infection (OR = 0.06 [0.00–0.30], p = 0.007) remained associated with the absence of GAS infection. Using these criteria allowed for identifying subgroups of patients with increased likelihood of GAS infections: from 27% overall (n = 224) to 32% for non-abdominoperineal infections (n = 186), 33% for patients without immunodeficiency (n = 166) and 38% for both non abdominoperineal infections in patients without immunodeficiency (n = 138). A sensitivity analysis for monomicrobial GAS infections yielded similar results with the addition of younger age and non-nosocomial infections as predictors.

**Conclusion:** Upon admission, the absence of immunodeficiency and of an abdominoperineal infection in NSTI patients were covariables associated with GAS infection.

**Compliance with ethics regulations: **Yes.

**Table 1:** Admission characteristics associated with group A streptococcal documentation for 224 patients admitted for necrotizing soft tissue infection

	Available data	Overall (n = 224)	Gas (n = 60)	Others (n = 164)	p (univariate)^a^	Adjusted OR^b^	p (multivariate)^b^
Demographical data							
Age, years, median (IQR)	224	64.00 [53.00–74.25]	60.00 [50.00–72.00]	65.00 [55.50–75.00]	0.083		
Male gender, n (%)	224	127 (56.7)	31 (51.7)	96 (58.5)	0.443		
Comorbidities, n (%)							
Diabetes mellitus	224	83 (37.1)	18 (30.0)	65 (39.6)	0.244		
Immunodeficiency	224	58 (25.9)	6 (10.0)	52 (31.7)	**0.002**	0.29 [0.10–0.74]	< 0.015
HIV^c^ infection	224	2 (0.9)	0 (0.0)	2 (1.2)	0.954		
Cancer	224	21 (9.4)	0 (0.0)	21 (12.8)	**0.008**		
Corticosteroids	224	36 (16.1)	6 (10.0)	30 (18.3)	0.197		
Obliterating arteritis of the lower limbs	224	24 (10.7)	5 (8.3)	19 (11.6)	0.651		
Liver cirrhosis	224	9 (4.0)	0 (0.0)	9 (5.5)	0.142		
Chronic kidney disease	224	25 (11.2)	4 (6.7)	21 (12.8)	0.293		
Chronic alcohol consumption	224	27 (12.1)	5 (8.3)	22 (13.4)	0.422		
Obesity	224	57 (25.4)	13 (21.7)	44 (26.8)	0.54		
Prior to admission							
Time from first symptom, days, median (IQR)	224	5.00 [2.00–9.75]	5.00 [2.00–7.25]	5.00 [2.00–10.00]	0.599		
Antibiotic treatment, n (%)	221	137 (61.2)	30 (50.8)	107 (66.0)	0.057		
NSAID^d^ use, n (%)	222	46 (20.5)	19 (31.7)	27 (16.7)	**0.024**	–	0.122
Presentation upon admission							
Nosocomial infection, n (%)	222	45 (20.1)	4 (6.7)	41 (25.3)	**0.004**	–	0.197
Abdominoperineal infection, n (%)	223	38 (17.0)	1 (1.7)	37 (22.7)	**<** **0.001**	0.06 [0.00–0.30]	0.007
Shock, n (%)	220	91 (40.6)	21 (35.6)	70 (43.5)	0.369		
Creatininemia, µmol/L, median [IQR]	210	112.50 [69.00–171.25]	123.00 [71.25–187.25]	109.50 [67.25–167.75]	0.571		
Uremia, mmol/L, median [IQR]	207	78.00 [40.50–109.00]	10.25 [5.45–18.02]	9.80 [5.20–19.10]	0.966		
Plasma bicarbonate, mmol/L, median [IQR]	193	57.00 [34.00–86.00]	22.70 [20.10–26.00]	23.00 [19.50–27.05]	0.943		
Blood leucocytes 10^3^/mm^3^, median [IQR]	219	14 .4 [9.5, 21.6]	17.2 [12.4, 22.5]	13.6 [9–21]	**0.016**	–	0.067
Platelets 10^3^/mm^3^, median [IQR]	189	217 [153–329]	224 [181–313]	207 [144–344]	0.45		
Hemoglobinemia, g/dL, median [IQR]	215	30.00 [12.00–62.00]	11.05 [10.15–12.50]	10.60 [9.35–12.10]	0.171		
Arterial lactates-mmol/L-median [IQR]	146	20.50 [9.00–34.75]	2.10 [1.50–3.60]	2.00 [1.20–3.40]	0.677		

^a^p values for univariate comparison of documented group A streptococcal infection vs others; Chi squared test or Fisher’s exact test were used for categorical data according to sample size, Mann-Whitney’s test were used for continuous variables due to non-parametrical distribution

^b^p values and adjusted ors from a generalized linear regression model assessing the relationship between admission characteristics and group A streptococcal documentation. The model included all variables with a p value < 0.05 in univariate analysis. Analysis regarding 213 patients (11 patients excluded for missing data on one of the variables of the model)

^c^hiv: Human immunodeficiency virus

^d^nsaid: non-steroidal anti-inflammatory drug

### P-201 Dosage of cefotaxim in patients with sickle cell patients and acute chest syndrome receiving continuous infusion versus intermittent bolus administration

#### Keyvan Razazi^1^, Enora Berti^1^, Jerome Cecchini^2^, Guillaume Carteaux^1^, Nicolas De Prost^1^, Anoosha Habibi^3^, Anne Hulin^4^, Armand Mekontso Dessap^1^

##### ^1^Service de réanimation médicale, Hôpital Henri Mondor, Créteil, France; ^2^Service de réanimation, centre hospitalier intercommunal de Créteil, Créteil, France; ^3^Unité des Maladies Génétiques du Globule Rouge, Hôpital Henri Mondor, Créteil, France; ^4^Laboratoire de Pharmacologie-Toxicologie, Hôpital Henri Mondor, Créteil, France

###### **Correspondence:** Keyvan Razazi (keyvan.razazi@aphp.fr)

*Ann. Intensive Care* 2020, **10(Suppl 1):**P-201

**Rationale:** Sickle-cell disease is the most common genetic disorder in the world. A complication of this disease is the acute chest syndrome (ACS) which is associated with a high risk of death. Respiratory tract infections are often mixed up and the introduction of betalactam antibiotics is recommended. Glomerular hyperfiltration is common and responsible of a high risk of underdosing. This study compares Cefotaxim continuous infusion to intermittent bolus in adult patients with ACS.

**Patients and methods:** This observational retrospective monocentric study included ACS admitted in intensive care unit and treated by Cefotaxim with at least one plasmatic dosing between May 2016 and August 2019.

**Results:** Thirty patients received bolus administration while the 30 others received continuous infusion. We observed 5 patients (16%) and 28 patients (93%) with a Cefotaxim trough level ≥ 2 mg/L in the bolus and continuous group, respectively (p < 0.001). The median residual concentration was 0 mg/L [0–0] and 10.5 mg/L [7.4–13.3] in the bolus and continuous group, respectively (p < 0.001). There was no toxic effect induced by overdosing of Cefotaxim.

**Conclusion:** Compared to intermittent bolus infusion, continuous Cefotaxim administration maximizes the pharmacokinetics parameters by obtaining a plasmatic concentration 5 times above the minimal inhibitory concentration of usual germs associated with ACS. Continuous infusion of time-dependant antibiotics seems to decrease the risk of underdosing in patients with sickle cell disease.

**Compliance with ethics regulations:** Not applicable.

### P-202 The outcome of TBI patients with acute diabetes insipidus in the ICU

#### Mariem Dlela, Olfa Turki, Rezk Gorbel, Najeh Baccouche, Mounir Bouaziz

##### Habib Bourguiba Hospital, Sfax, Tunisia

###### **Correspondence:** Mariem Dlela (mariem241090@gmail.com)

*Ann. Intensive Care* 2020, **10(Suppl 1):**P-202

**Rationale:** Acute diabetes insipidus following head injury and its effect on patients outcome have not been sufficiently evaluated in large prospective studies. The aim of this study was to determine the incidence of acute CDI, delay of onset predictive factors and its impact on TBI patients.

**Patients and methods:** We conducted a prospective cohort, including all patients admitted to ICU with moderate to severe TBI, defined as a Glasgow coma scale (GCS) below twelve. For each TBI patient plasma sodium was measured daily, and if abnormally high, urine specific gravity and osmolality were measured. CDI was diagnosed using the Seckl and Dunger criteria. Acute CDI was defined as CDI diagnosed in the first week following injury. All patients were screened with a brain MRI.

**Results:** During our study’s period, 451 trauma patients were admitted to our ICU, 158 presented with moderate to severe TBI and were included. On admission, our patients had a mean age at 36.8 ± 19, a mean injury severity score (ISS) at 35 ± 11, a mean Abbreviated Injury Severity (AIS) of the Head at 4.5 ± 0.7 and a mean GCS at 7 ± 3. Twenty-three percents (37 patients) of the patients developed hypernatremia and 15% (24 patients) were diagnosed with acute CDI. In 16 of 24 (66%), the CDI was nonrecovering. The median delay to develop transient CDI was 28 h and for non-recoviring CDI was 68 h (p = 0.045). None of the patients with acute CDI had direct hypothalamic or pituitary lesions. Factors associated to the occurrence of acute CDI were: younger age (29 ± 19 vs 38 ± 19, p = 0.039), neuro-surgery (22% vs. 9%, p < 0.001), hemorrhagic shock (24% vs. 7%), p < 0.001), cerebral edema (25% vs. 10%), p < 0.019), and fractures to the base of the skull (20% vs. 8%, p = 0.022). Patients who developed CDI had a significantly higher mortality than those who did not (15 of 24 (62%) vs. 17 of 134 (12%), p < 0.001). There were no difference in terms of mortality between non-recovering and transient CDI (68% vs. 50%, p = 0.1), similarly the onset of CDI did not affect mortality (32 h vs. 35 h, p = 0.5). Patients with acute CDI had poor Glasgow Outcome Scale (2 ± 1.5 vs. 3.4 ± 1.3, p < 0.001) and longer ICU LOS (23 ± 14 vs. 12 ± 11, p = 0.001).

**Conclusion:** Acute CDI is associated with higher mortality and poor outcome. Therefore it is essential to diagnose and treat it promptly and correctly.

**Compliance with ethics regulations:** Yes.

### P-203 Acute glucocorticoid deficiency following traumatic brain injury

#### Mariem Dlela, Rania Ammar Zayani, Abir Bouattour, Najeh Baccouche, Mounir Bouaziz

##### Habib bourguiba hospital, Sfax, Tunisia

###### **Correspondence:** Mariem Dlela (mariem241090@gmail.com)

*Ann. Intensive Care* 2020, **10(Suppl 1):**P-203

**Rationale:** Published data demonstrates that long-term hypopituitarism could be common after traumatic brain injury (TBI).However, few studies focused on radiological, clinical, and repetitive endocrine assessment in the acute phase. The aim of the study was to evaluate the early changes in the adrenal axis following (TBI) and to evaluate whether hormone changes affect patient’s outcome.

**Patients and methods:** We conducted a prospective study, including all patients admitted to a university hospital ICU with moderate to severe traumatic brain injury (TBI), defined as a Glasgow coma scale below twelve (GCS < 12). Each patient underwent sequential measurement of plasma cortisol (PC) on days 1, 3, 7 and 10 after TBI. We defined adrenal insufficiency as PC less than 100 ng/mL. Patients who received glucocorticosteroid therapy were excluded. Outcome was measured by incidence of death, and Glasgow outcome scale (GOS) on day thirty.

**Results:** During our study period’s, 162 were included. On admission, patients had a mean age at 36.8 ± 19, a mean injury severity score (ISS) at 35 ± 11 and a mean GCS at 7 ± 3. None had major abdominal trauma. According to our analysis 47 TBI patients (33%) had at least 1 PC measurement of less than 100 ng/mL. The median delay to develop adrenal insufficiency was 3 days with recovery occurring in 75% of the cases (35 patients) by day 10. Lower PC measurements on day 1 were associated to higher mortality rates (51 ± 11 vs. 126 ± 39, p = 0.004), and poor outcome: GOS (pearson’s R = 0.72, p < 0.001) and ong-term sequelae (47 ± 20 vs. 157 ± 52, p < 0.001). Patients with persistent cortisol deficiency (lasting beyond 10 days) had a higher mortality than those with transient cortisol deficiency (50% vs. 10%, p < 0.001).

**Conclusion:** Our study showed the presence of low plasma cortisol concentrations, inappropriate for the acutely sick, in TBI patients with a clear association between acute cortisol deficiency and subsequent mortality and GOS. Unfortunately, no strict criteria define a normal response of the corticotropic system after TBI. Thus, an ongoing debate exists about supportive cortisol replacement in patients requiring intensive care treatment with presumed relative adrenal insufficiency.

**Compliance with ethics regulations:** Yes.

### P-204 Thyrotropic dysfunction following traumatic brain injury

#### Mariem Dlela, Rezk Gorbel, Olfa Turki, Najeh Baccouche, Mounir Bouaziz

##### Habib Bourguiba Hospital, Sfax, Tunisia

###### **Correspondence:** Mariem Dlela (mariem241090@gmail.com)

*Ann. Intensive Care* 2020, **10(Suppl 1):**P-204

**Rationale:** Endocrine abnormalities have been reported with varying frequencies, following traumatic brain injury (TBI). Few studies have examined the clinical features and outcomes of isolated acute thyrotropic hormone deficiencies after TBI. The aim of the study was to evaluate the early changes in thyrotropic hormone levels after traumatic brain injury (TBI) and to evaluate whether hormone changes are related to outcome

**Patients and methods:** We conducted a 12 months long prospective cohort, including all patients admitted to a university hospital ICU with moderate to severe traumatic brain injury (TBI), defined as a Glasgow coma scale below twelve (GCS < 12). Blood samples for basal hormone values of thyroid-stimulating hormone (TSH) and free thyroxine (fT4) were obtained on days 1, 3, 7 and 10. TSH serum concentrations were considered normal at > 0.27 Mu/L; fT4 at > 12 pmol/L. A thyrotropic insufficiency was defined as low fT4 and low TSH plasma levels. All patients were screened with a brain MRI. Patients were also monitored for neurological deterioration, including cognitive decline, convulsive seizures, increase in cerebral edema and brain herniation that were simultaneous to the diagnosis.

**Results:** During our study period’s, 465 trauma patients were admitted to our ICU and 134 met the inclusion criteria. On admission, our patients had a mean age at 36.8 ± 19, a mean injury severity score (ISS) at 35 ± 11, a mean Abbreviated Injury Severity (AIS) of the Head at 4.5 ± 0.7 and a mean GCS at 7 ± 3. Of the 134 patients a thyrotropic insufficiency was diagnosed in 26 patients (20%) during the first 10 days. The median delay to thyrotropic insufficiency diagnosis was 3 days. In three of 26 (11%), the thyrotropic insufficiency was nonrecovering during the patient’s ICU stay and was transient for the rest. None of the patients with acute thyrotropic insufficiency had direct hypothalamic or pituitary lesions on the brain MRI. Factors associated to the occurrence of acute thyrotropic insufficiency were: the AIS of the head (4.8 ± 0.4 vs. 4 ± 0.7, p = 0.001), cerebral contusions (95% vs. 65%, p = 0.008), subarachnoid haemorrhage (85% vs. 60%, p = 0.037) and subdural haematoma (57% vs. 30%, p = 0.012). Thyrotropic insufficiency was associated to neurological deterioration (p = 0.001) on the day of diagnosis but did not affect ICU mortality (12% vs. 11%, p = 0.6).

**Conclusion:** In this study, low pituitary-thyrotropic axis hormone levels were found in the acute phase of TBI and were associated to neurological deterioration but with no perceived effect on ICU mortality.

**Compliance with ethics regulations:** Yes.

### P-205 Transcranial Doppler (DTC) predictive of complication after a serious cranial trauma (TCG)

#### Souhila Sadat, Dalila Zeghdoud, Dalila Bougdal, Kamel Guenane

##### EHS salim zemirli, Alger, Algeria

###### **Correspondence:** Souhila Sadat (sadatsouhila@hotmail.fr)

*Ann. Intensive Care* 2020, **10(Suppl 1):**P-205

**Rationale:** The renewed interest in the pathophysiology of severe traumatic brain injury (TCG), allowed the understanding of the pathophysiological mechanisms leading to neuronal death.The non-invasive, easy, patient-based technical DTC allows evaluation of cerebral blood flow. Purpose of the study: to determine the contribution of transcranial doppler (DTP) in the prevention of post-traumatic ischemia.

**Patients and methods:** A monocentric, observational, prospective study over a period of 2 years, including 100 TCG in the monitoring of cerebral blood flow (DSC) was provided by the DTC. We collected the following data: age, gender, lesion mechanism, lesion association, GLASGOW score at admission, time to perform the initial scan, time to perform the initial Doppler, various abnormalities found at the initial DTP, the analysis of the level of MAP according to each situation of cerebral blood flow, the proposed therapies, the time to obtain a correct DTC.

**Results:** The mean age of the patients was 34 ± 16.4 years, with a clear male predominance (sex ratio = 7.33 M/1F). Secondary to road accidents in 64% of cases, polytraumatized in 75% of cases with an average GLASGOW score of 6.73 with 95% CI [6.46–7], average ISS of 39, 7 ± 17, the average delay of the initial DTP was 6.68 h with 95% CI [6.06 h–7.35 h], it was correct in (41%), hypohemic in (59%), the statistical analysis showed no difference between the delay in setting up a hypohemia and the presence of a correct cerebral blood flow (P = 1.000), the statistical analysis of the MAP in the DTC group hypohemia compared to the correct DTC group objectified the absence Significant difference between the two groups. The realization of DTP allowed therapeutic prioritization, the introduction of norepinephrine was in 100% of cases, osmotherapy in 100% of cases, optimization of sedation in 29.34% of cases, the introduction of penthotal in 13.54% of cases and the completion of decompressive in 8.82% of cases. Statistical analysis of mortality showed a significant difference in mortality (P = 0.07) in the hypohemic DTC group compared with the correct doppler .

**Conclusion:** TTC is an essential monitoring tool of cerebral hemodynamics, which may in prove the neurologic outiome of TCG.

**Compliance with ethics regulations:** Yes.

### P-206 Prevalence, characteristics, risk factors, and short-term consequences of hyponatremia in intensive care unit patients with post-traumatic brain injury

#### Olfa Turki, Amal Triki, Najeh Baccouche, Karama Bouchaala, Yousfi Mounir, Mabrouk Bahloul, Mounir Bouaziz

##### Habib Bourguiba University Hospital, Sfax, Tunisia

###### **Correspondence:** Olfa Turki (olfa.turki.rea@gmail.com)

*Ann. Intensive Care* 2020, **10(Suppl 1):**P-206

**Rationale:** Hyponatremia is a frequent electrolyte disturbance in hospitalized patients. It is particularly common in brain-injured patients with significantly elevated morbidity and mortality. The aim was to study the prevalence of hyponatremia in the acute phase of post-traumatic cerebral aggression, its degree of severity, its predictive factors as well as its prognostic impact in the population of post-traumatic brain injury.

**Patients and methods:** This is a retrospective study, carried out over a period of 4 years about all traumatized head patients who developed hyponatremia during the first 36 h of their stay. The descriptive part treated all patients who developed hyponatremia by detailing its different stages of severity.The analytical part treated the patients who developed a hypo-osmolar hyponatremia with a threshold of 130 mmol/L retained to define the severity.

**Results:** During the study period, the incidence of hyponatremia in head trauma patients was 30.8%. The occurrence of hyponatremia was associated only with the occurrence of early seizures (p = 0.006). Severe hyponatraemia was associated with paroxysmal occurrence (p = 0.002), mass effect (p = 0.01), and hemostasis disorders. The multivariate study revealed that severe hyponatremia was associated with the Glasgow score (p < 0.001) and pupillary changes (p = 0.048). On the other hand, it is the initial variation in serum sodium that was associated with both the severity of the initial neurological examination; Glasgow (p < 0.001), SAPS2 (p = 0.001), PTS (p = 0.049) and PRISM scores (p = 0.007), haemodynamic instability (p = 0.07) and neurovegetative disorders (p = 0.04). Lesional features have also been found. Regarding the prognosis, the occurrence of initial hyponatremia had a protective effect: a more favorable GOS score p = 0.051 and a lower mortality (p = 0.045). A poor neurologic prognosis as well as a high mortality were associated with the most severe hyponatraemia and particularly with the initial variation of the sodium level (p = 0.002;). The mortality was 24.9%. It was also particularly related to the initial change in sodium levels (p < 0.001, 0.002).

**Conclusion:** We concluded that there is no association between post traumatic early hyponatremia and the severity of the initial clinical presentation. However, the depth of hyponatremia and especially the initial change in sodium levels have been associated with more severe clinical pictures and a more limited prognosis.

**Compliance with ethics regulations:** Yes.

### P-207 Post traumatic epilepsy: about 32 cases

#### Kamilia Chtara, Hasna Bouchiira, Wiem Ben Amor, Rania Ammar Zayani, Sabrine Bradai, Mabrouk Bahloul, Mounir Bouaziz

##### University of Sfax, Sfax, Tunisia

###### **Correspondence:** Kamilia Chtara (kamilia.chtaraelaoud@gmail.com)

*Ann. Intensive Care* 2020, **10(Suppl 1):**P-207

**Rationale:** Post-traumatic epilepsy (PTE) is one of the complications described in the aftermath of head trauma. Its incidence is variable in the literature because of its clinical polymorphism. Objectives of the study was to analyze the epidemiological profile (clinico-biological, radiological, therapeutic and evolutionary) of the patients having presented PTE and to determine the risk factors for this pathology by comparing them with the rest of the traumatized brain patients.

**Patients and methods:** Our study was retrospective. It was conducted in the intensive care unit (ICU) of our University Hospital between 2009 and 2012. Were included in our study all patients admitted to the service with brain injury and a glycaemia above 8 mmol/L during the first 24 h post-trauma.

**Results:** The incidence of PTE was 4.6%. (32 among 694) The average age was 29.5 ± 17.1 years. The sex ratio was 5.4. The average of GCS was 7.6 ± 3.6. Three (9.4%) patients had initial motor impairment. Seizures were observed in 3 (9.3%) patients during the first 24 h of hospitalization. The mean delay of occurrence of PTE was 17 ± 17.7 months. PTE was diagnosed before the end of the first post-traumatic year in 13 patients (54% of cases). The most commonly observed brain lesions were cortical brain contusions (93.8%), subdural hematoma (71.9%). Diffuse axonal lesions were observed among 12 patients. EEG was performed in 9 patients (28.1%). It objectified paroxysms in 3 cases.The final outcome was marked by the death of 9 (28.1%) patients. Recovery was achieved in 90.6% of comatose patients (29 patients) within an average of 18.3 ± 18.8 days (extremes 1 to 90 days). Overall, the final outcome was considered unfavorable in 27 patients (84.3%) and favorable in only 5 patients (15.6%). In univariate analysis, the risk factors associated with PTE were neuro-psychiatric antecedents and alcoholism,a duration of coma greater than 8 days immediate convulsions (< 24 h), intracranial hypertension, hypercapnia, extra-dural hematoma (p = 0.009), intracerebral hemorrhage (p = 0.001), subdural hematoma (p = 0.006), pneumocephaly (p = 0.001), cerebral edema (p = 0.019), intracranial mass effect (p = 0.022) and cerebral involvement (p = 0.048). In multivariate analysis, the independent factors correlated with PTE were neuropsychiatric antecedents, immediate seizures, pneumocephaly, deep contusion of the cerebral peduncle and cerebral edema.

**Conclusion:** PTE is a leading cause of disability for the Trauma Brain Injury. So, we should define patients with risk of PTE to prevent this complication.

**Compliance with ethics regulations:** Not applicable.

### P-208 Hydroelectrolytic disorders in brain injury -Intensive care Unit A1-

#### Ihssan Mehrez, Abdelkrim Shimi, Ali Derkaoui, Mohammed Khatouf

##### CHU HASSAN II FES-intensive care A1, Fes, Morocco

###### **Correspondence:** Ihssan Mehrez (mehrez.ihssan@gmail.com)

*Ann. Intensive Care* 2020, **10(Suppl 1):**P-208

**Rationale:** Electrolytic disorders are common in neuro-resuscitation, especially dysnatremias and dyskalemias. Hyponatremias are the most frequent, including the 2 main etiologies: the syndrome of inappropriate secretion of antidiuretic hormone (SIADH) and the “cerebral salt wasting” syndrome (CSW). Diabetes insipude of central origin secondary to a lack of DHA secretion is the second most common disorder.

**Patients and methods:** It is a prospective study, analysing all the brains injured admitted to the A1 intensive care unit of CHU HASSAN in Fez, Morocco. Study spread over a 5-month period from 01/08/2018 to 31/12/2018. The objective of the study is to detect the most frequent hydro-electrolytic disorders and to evaluate the therapeutic effectiveness of the service protocols.

**Results:** All these brains injured have caused HE disorders over a period of time varying between D2 and D5: *18 cases of hyponatremia (30%)/12 cases of hypernatremia (19%), *19 cases of hypokaliemia (32%)/ 11 cases of hyperkaliemia (18%), *25 cases of hyperchloremia, or 41%/ 5 cases of hypochloremia (8%). *6 cases of diabetes insipidus, or 9.8%. *7 cases without HE disorder (11.4%). The treatment for these disorders was: *for hypoNa; it reached 118 mmol/L, initially corrected by a 24-hour water restriction, followed by an increase in the Basic Ration and furosemide boluses according to the ECV, even sodium loads for a single case of salt loss syndrome, while the main etiology remains the SIADH. *for HyperNa, it has reached 178 mmol/L, evaluated by the extracellular volume, corrected by enteral tap water after calculation of the hydric deficit. If HperNa is associated with polyuria greater than 2 cc/Kg/H; we speak of: *insipude diabetes, with polyuria up to 5 cc/Kg/H, compensated with potassium-containing solutions and blood ionogram monitored every 6 h. Desmopressin was used in titration, by bolus of 0.5 μg, with a diuresis objective between 1 and 1.5 ml/kg/h. *for hypokalemia, up to 2.2 g/dl, observed mainly in the acute phase of brain aggression, corrected by increase in BR for a K between 2.5 and 3 g/l, and by potassium loads if K below 2.5 g/L. The evolution: 5 deaths or 8.2% (2 cases of uncorrected diabetes insipidus), the restriction of disorders were corrected.

**Conclusion:** A knowledge of the hydroelectrolytic disorders encountered in this context is essential, as well as the implementation of a diagnostic and therapeutic protocol, which will reduce the time required to correct these disorders.

**Compliance with ethics regulations:** Yes.

### P-209 Decompressive craniectomy in traumatic brain injury: about 147 cases

#### Karama Bouchaala, Rania Ammar Zayani, Sabrine Bradai, Kamilia Chtara, Chokri Ben Hamida, Mounir Bouaziz

##### Faculty of Medecine,University of Sfax, Sfax, Tunisia

###### **Correspondence:** Karama Bouchaala (karamamnif@gmail.com)

*Ann. Intensive Care* 2020, **10(Suppl 1):**P-209

**Rationale:** Decompression craniectomy (DC) is indicated for intracranien hypertension (ICHT) refractory to medical treatment in traumatic brain injury (TBI).o study outcomes of DC in TBI.

**Patients and methods:** A 9-year retrospective study (January 1, 2009 to December 31, 2017) was conducted in a Tunisian intensive care unit (ICU). We included patients who underwent DC following TBI. The functional prognosis was assessed by Glasgow outcome scale (GOS).

**Results:** We included 147 patients. The majority was younger between 16 and 45 years (66%). Sex ratio of 8.8. The mean (SD) length of stay in ICU was 22.9 ± 21.8 days. The mean Glasgow Coma Score (GCS) (SD) was 7.2 ± 3.6 and GCS ≤ 8 in 68.1%. SOFA score > 5 was found in 71 patients (48.3%) and SAPSII score ≥ 30 in 121 patients (82.3%). The cerebral CTscan at admission showed acute subdural hematoma (ASDH) in (84.4%), cerebral oedema (65.3%) and cerebral contusions (83%). According to MARSHALL’s classification at the initial CTscann 79.6% of patients were Class IV or V. DC was performed within the first 6 h in 63.9% at admission according to pupillary modification (55,1%), mass effect and center line deviation > 5 mm (81,6%). Mortality rate was 42.2% (62 patients). Among the survivors after resuscitation: a good recovery (GOS 4 or 5) was observed in 51.7%, GOS 3 was observed in 36.5%, GOS 2 was observed in 11.8%.GOS at 6 months showed a good recovery (GOS 4 or 5) in 70%, GOS 3 in 22.5%, GOS 2 in 7.5% Cranioplasty was performed in 10 patients (6.8%) within 3 to 9 months. In univariate study, risk factors statistically associated with mortality were age > 65 years (p = 0.001), GCS ≤ 8 (p = 0.001), SOFA > 5 (p = 0.01), SAPSII (p < 0.001), hyperglycemia ≥ 8 mmol/l (p = 0.03), PaCO_2_ > 45 mmHg (p=0.002), brain edema (p=0.022), mass effect > 5 mm (p = 0.001), use of catecholamines (p = 0.001), use of corticosteroids (p = 0.034). Risk factors statistically associated with pour prognosis (GOS2-3) at discharge were GCS ≤ 8 (p = 0.029), SAPSII > 30 (p = 0.035), natremia < 135 mmol/l (p = 0.009), dilation of the ventricles (p = 0.017) and post-traumatic epilepsy (p = 0.038).

**Conclusion:** DC in TBI could be a life-saving procedure that allows for better survival and functional prognosis if its indication is given at time in well selected patients.

**Compliance with ethics regulations:** Not applicable.

### P-210 Gastrointestinal bleeding in immunocompromised patients in the ICU

#### Jennifer Catano, Virginie Lemiale, Eric Mariotte, Sandrine Valade, Michaël Darmon, Elie Azoulay, Lara Zafrani

##### Hôpital Saint Louis, Paris, France

###### **Correspondence:** Jennifer Catano (jcatano@hotmail.fr)

*Ann. Intensive Care* 2020, **10(Suppl 1):**P-210

**Rationale:** Gastrointestinal bleeding (GIB) is a common and potentially life-threatening cause of admission in the intensive care unit (ICU) with an estimated ICU mortality rate of 2 to 10%. Data are scarce on GIB in immunocompromised patients. Thus, we sought to describe etiologies, management and outcomes of GIB in critically ill immunocompromised patients. We aim to identify factors associated with efficacy of therapeutic strategies and mortality.

**Patients and methods:** This is a monocentric, retrospective observational study including all immunocompromised patients admitted to the ICU for GIB from January 2015 to December 2017. Results are reported as median (IQR) or number (%).

**Results:** Forty-six patients were included. Median age at admission was 60 [27–87] years old. Immunosuppression was secondary to hematological malignancies (n = 29, 62%), HIV (human immunodeficiency virus) (n = 6, 13%), chemotherapy for solid tumor (n = 7, 15%), and other immunosuppressive drugs (n = 26, 56%). Four patients (9%) had received bone marrow transplantation. At ICU admission, 12 patients (26%) had anticoagulant therapy, seven (15%) had antiplatelet therapy and 13 (28%) had severe thrombocytopenia (< 50 G/L). Half of the patients presented with hemorrhagic shock (n = 21, 46%). The median SOFA score at admission was 6 [0-16]. During ICU stay, 21 (46%) patients required vasopressors, 32 (70%) patients required invasive mechanical ventilation and 15 (33%) patients received renal replacement therapy. ICU mortality rate was 17% (n = 8). Esophagogastroduodenoscopy was performed in almost all patients (n = 42, 91%). Eighteen (43%) endoscopic therapies designed for hemostasis were performed (adrenaline injection, clips or thermal devices, esophageal varices ligation). Sixteen digestive biopsies (35%) were performed. Eighteen (39%) colonoscopies were performed. Twenty-three recurrences were recorded. Despite endoscopic management, two patients (4%) required embolization by interventional radiology and three patients required surgical hemostasis (6%) because of refractory GIB. Most of GIB were due to specific malignant lesions (n = 10, 22%), followed by esophageal varices rupture (n = 7, 15%), ulcer bleeding (n = 8, 17%) and diverticular hemorrhage (n = 7, 15%). Infectious diseases were diagnosed in three patients (7%), including one Clostridium colitis, one erosive gastritis with Helicobacter pylori and one esophageal candidiasis.

**Conclusion:** GIB is associated with a high mortality rate in immunocompromised patients, especially in patients with hematological malignancies. Specific malignant lesions were the main etiology and may be difficult to treat. Comparison with critically ill non-immunocompromised patients with GIB will help physicians to provide specific therapeutic strategies in this population.

**Compliance with ethics regulations:** Yes.

### P-211 Risk factors for delayed defecation and impact on outcome in critically ill patients: a multicenter prospective non-interventional study

#### Benoît Painvin^1,*^, Arnaud Gacouin^2^, Antoine Roquilly^3^, Claire Dahyot-Fizelier^4^, Sigsimond Lasocki^5^, Chloe Rousseau^6^, Denis Frasca^7^, Philippe Seguin^8^

##### ^1^Anesthésie-Réanimation/CHU RENNES, Rennes, France; ^2^Réanimation Médicale/CHU RENNES, Rennes, France; ^3^Réanimation Chirurgicale/CHU NANTES, Nantes, France; ^4^Réanimation chirurgicale/CHU POITIERS, Poitiers, France; ^5^Anesthésie-Réanimation/CHU ANGERS, Angers, France; ^6^Centre investigation clinique/CHU RENNES, Rennes, France; ^7^Anesthésie-Réanimation/CHU POITIERS, Poitiers, France; ^8^Réanimation chirurgicale/CHU RENNES, Rennes, France

###### **Correspondence:** Benoît Painvin (painvinbe@gmail.com)

*Ann. Intensive Care* 2020, **10(Suppl 1):**P-211

**Rationale:** Delayed defecation is very common in Intensive Care Units (ICU) and it increases length of mechanical ventilation (MV), ICU length of stay (LOS) and possibly mortality. The objective of this prospective multicenter study was to determine risks factors for constipation in ICU and to evaluate their impact on mortality.

**Patients and methods:** It was a prospective multicenter non-interventional trial performed in 5 university ICUs in France from January 2017 to October 2017. All patients ≥ 18 years old who had an expected LOS of 3 days and mechanically ventilated for at least 2 days were eligible. Defecation was defined as the time of the first stool passage.

**Results:** 396 patients were included in the analysis. A stool passage was observed in 84% of the patients during their ICU stay with a mean delay of 7 ± 3 days. In multivariate analysis, risk factors for delayed passage of stool were non-invasive ventilation use and time spent under invasive ventilation whereas alcoholism, laxative treatment (before and after ICU admission) and nutrition ≤ 48 h favoured passage of stool (Table 1). No relations between constipation and mortality were found.

**Conclusion:** We highlighted new and important independent factors for constipation in critically ill patients leading to a better prevention of this phenomenon..

**Compliance with ethics regulations:** Yes.

**Table 1:**
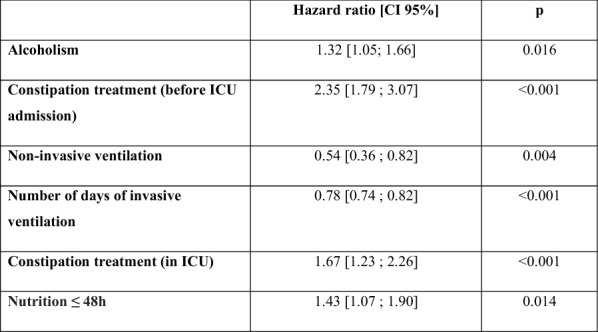
Multivariate analysis

### P-212 Bacterial ecology of intra-abdominal sepsis

#### Khalid Khaleq, Asmaa Ouchane, Aziz Bouhouri, Khalid Zerouali

##### Faculté de médecine et de pharmacie / CHU Ibnou Rochd, Casablanca, Morocco

###### **Correspondence:** Khalid Khaleq (khaleq20@gmx.fr)

*Ann. Intensive Care* 2020, **10(Suppl 1):**P-212

**Rationale:** Community peritonitis is a frequent medical-surgical emergency of the adult, acquired by the patient in a non-hospital setting. Careful multidisciplinary care is essential, involving surgeons, anesthetists, microbiologists and radiologists. The objective of our study is to determine the bacteriological aspects of intra-abdominal sepsis, to describe their sensitivity profiles and to propose treatment regimens for the management of community peritonitis.

**Patients and methods:** We conducted a descriptive retrospective study spanning a period of two years from January 2017 to January 2019 involving 312 cases of community abdominal sepsis operated in the operating room of surgical emergencies of our hospital. We included in our study adult patients admitted for suspected or confirmed abdominal sepsis who had undergone bacteriological examinations on the abdominal collections. Samples taken are sent directly to the bacteriology laboratory for bacteriological analysis of the results.

**Results:** The studies showed the mean age is 45.36 years old, with a sex ratio of 2.76. We found 215 positive results mainly of peritoneal origin with a percentage of 59.53% peritonitis, dominate by intestinal peritonitis 41.40% followed by the appendicular origin 33.59% then peritonitis by perforation of ulcer. The most incriminated organism in intraabdominal sepsis is *E. coli* with a percentage of 44.33% of the total germs found, followed by streptococcus spp 11.14%, enterococci 5.32%, non-fermenting BGN composed mainly of pseudomonas aeruginosa 5.66%, staphylococci 4.66% and *Acinetobacter baumanii* 3.33%. Note also the presence of Bacteroides fragilis is 1%. *E. coli* had a very low sensitivity profile for amoxicillin/clavulanic acid (59.4%), unlike ceftriaxone, gentamicin, amikacin and ertapenem, which had a sensitivity of 77.44%, respectively. 90.22%, 100%, 90.22%.

**Conclusion:** Knowledge of the bacterial ecology of intraabdominal sepsis is important in the choice of probabilistic antibiotherapy, pending bacteriological findings.

**Compliance with ethics regulations:** Not applicable.

### P-213 Nutritional management in medical intensive care unit: a Tunisian national survey

#### Samia Ayed^1^, Amira Jamoussi^1^, Rania Ammar Zayani^2^, Mounir Bouaziz^2^, Amel Mokline^3^, Amenallah Messaadi^3^, Dhouha Lakhdhar^1^, Oussema Jaoued^4^, Souheil Elatrous^4^, Hela Maamouri^5^, Nozha Brahmi^5^, Imen Ben Saida^6^, Mohamed Boussarsar^6^, Ines Fathallah^7^, Nedia Kouraichi^7^, Jihene Guissouma^8^, Hatem Ghadhoune^8^, Dorra Sakis^9^, Foued Daly^9^, Salah Ben Lakhal^9^, Kais Ben Romdhane^10^, Takoua Merhabene^11^, Houda Mateur^12^, Youssef Zied Elhechmi^13^, Zouheir Jerbi^13^, Jalila Ben Khelil^1^, Mohamed Besbes^1^

##### ^1^Abderrahmen Mami Hospital, Ariana, Tunisia; ^2^Habib Bourguiba Hospital, Sfax, Tunisia; ^3^Burns And Traumatology Hospital, Ben Arous, Tunisia; ^4^Taher Sfar Hospital, Mahdia, Tunisia; ^5^Department Of Intensive Care And Toxicology, Tunis, Tunisia; ^6^Farhat Hached Hospital, Sousse, Tunisia; ^7^Yassminet Regional Hospital, Ben Arous, Tunisia; ^8^Habib Bougatfa Regional Hospital, Bizerte, Tunisia; ^9^Larabta Hospital, Tunis, Tunisia; ^10^Carthagene Private Hospital, Tunis, Tunisia; ^11^Regional Hospital Of Zaghouan, Zaghouan, Tunisia; ^12^Regional Hospital Of Tozeur, Tozeur, Tunisia; ^13^Habib Thameur Hospital, Tunis, Tunisia

###### **Correspondence:** Samia Ayed (samia.ayed@yahoo.fr)

*Ann. Intensive Care* 2020, **10(Suppl 1):**P-213

**Rationale:** Nutritional support is an integral part of critical care management. Malnutrition is a high risk situation of severe complications, longer intensive care and hospital length of stay and higher mortality. No data are yet available about nutritional management and risk of malnutrition in Tunisian medical intensive care units (ICU). The purpose of this study was to describe nutritional management in medical intensive care patients and to evaluate the risk of malnutrition.

**Patients and methods:** We conducted a prospective observational cross-sectional study in medical ICUs all around the Tunisian country on the 18th September 2018. All participant units received a questionary form about routine nutritional management and data of all patients hospitalized in ICU on the study day. Collected data were: demographic characteristics, reason for admission, severity scores and subjective evaluation of nutritional status on admission, type and volume of nutritional support on the study day and the day before, nutritional status, nutric score and biological data on the study day, reasons for nutritional interruption and other supports prescribed.

**Results:** Thirteen ICU all around Tunisia participated to the study. No ICU had a nutrition team and only one had a written nutrition protocol. Four ICUs evaluated systematically the nutritional status on admission. All ICUs were aware and practiced early enteral nutrition in patients unable to maintain oral intake with a systematic supplementation of oligoelements and minerals. Neither target energy nor protein intake were calculated. On the study day, 89 patients were hospitalized with an occupation rate of 58%. Mean age was 51 ± 28 years. Mean body mass index was 22 ± 9 and 69% of patients were judged well nourished. Enteral nutrition support was prescribed on admission in 66% of cases with a mean caloric intake of 1100 ± 614 kcal/day. The mean caloric target on the study day was 1721 ± 637 kcal/day with a mean caloric intake of 1677 ± 594 kcal/day and a mean caloric gap of 44 ± 43 kcal/day. The mean nutric score and body mass index on the study day were 3 ± 2 and 24 ± 6 respectively. Twenty patients were judged malnourished by the nutric score and twenty two by clinical evaluation. A good correlation was found between nutric score and clinical evaluation of nutritional status (k = 0.773).

**Conclusion:** Tunisian ICUs don’t have nutrition team or nutritional written protocol. Early enteral feeding and supplementation is common. A good correlation exists between nutric score and clinical nutrition status evaluation.

**Compliance with ethics regulations:** Yes.

### P-214 Stochastic Targeted (STAR) glycemic control: improved performances and safety for all

#### Vincent Uyttendaele^1^, Jennifer L. Knopp^2^, Marc Pirotte^3^, Philippe Morimont^3^, Geoffrey M. Shaw^4^, J. Geoffrey Chase^2^, Thomas Desaive^1^, Benoit Misset^3^, Bernard Lambermont^3^

##### ^1^GIGA-In silico Medicine, University of Liège, Liège, Belgium; ^2^Department of Mechanical Engineering, University of Canterbury, Christchurch, New-Zealand; ^3^Department of Intensive Care, University Hospital of Liège, Liège, Belgium; ^4^Department of Intensive Care, Christchurch Hospital, Christchurch, New-Zealand

###### **Correspondence:** Vincent Uyttendaele (vincent.uyttendaele@uliege.be)

*Ann. Intensive Care* 2020, **10(Suppl 1):**P-214

**Rationale:** Whether more intensive glycemic control (GC) is beneficial or harmful for critically ill patient has been debated over the last decades. GC has been shown hard to achieve safely and effectively in intensive care. The associated increased hypoglycemia and glycemic variability is associated with worsened outcomes. However, model-based risk-based dosing approach have recently shown potential benefits, improving significantly GC safety and performances. The Stochastic TARgeted (STAR) GC framework is a model-based controller using a unique risk-based dosing approach. STAR identifies model-based patient-specific insulin sensitivity and assesses its potential variability over the next hours. These predictions are used to assess hypoglycemic risks associated with a specific insulin and/or nutrition intervention to reach a specific target band. This study analyzes preliminary clinical trial results of STAR in a Belgian ICU compared to the local standard protocol (SP).

**Patients and methods:** Ethics approval was granted by the local University Hospital Ethics Committee. Patient are included if two BG measurements > 145 mg/dL. STAR target band is 80–145 mg/dL compared to 100–150 mg/dL for the SP. Nutrition is administered enterally, and insulin infusion intravenously. GC is stopped if BG is stable (6 h in target band) or after 72 h of control. Safety is assessed by %BG < 80 mg/dL and %BG > 180 mg/dL. Performance is assessed by %BG in target band. Clinical data from 10 patients is used and compared to 20 retrospective patients under the SP.

**Results:** STAR outperformed the SP. Results summary is presented in Table 1. Despite the lower BG target, STAR safety was improved with lower %BG < 80 mg/dL (0.5% vs. 1%), and significantly lower %BG > 145 mg/dL (11% vs. 44%) and %BG > 180 mg/dL (2% vs 13%). STAR was highly effective with 89% BG in target band compared to 54% for the SP. Median [IQR] BG and nutrition rates achieved were lower for STAR (118 [109 129] vs. 139 [117 160] mg/dL and 7.0 [4.7 8.2] vs. 9.8 [8.6 11.5] g/h), while higher insulin rates were administered in STAR (3.0 [2.0 4.0] vs. 2.5 [2.0 3.0] U/h). However, workload was increased under STAR (12 vs. 7 measurements per day), as expected from measurement interval difference between STAR (3-hourly) and the SP (4-hourly).

**Conclusion:** This unique patient-specific risk-based dosing approach GC framework was successful in controlling all patients safely and effectively. These preliminary results are encouraging and show GC can be achieved safely and effectively at lower target bands. In turns, these improved GC outcomes could improve patient outcomes.

**Compliance with ethics regulations:** Yes.

**Table 1:** Results summary for performance, safety and compliance to protocol comparison between STAR and the Standard Protocol (SP)

	STAR	SP
#Patients	10	20
Total hours	455	5006
Average measurements per day	12	7
Median [IQR] BG (mg/dL)	118 [109 129]	139 [117 160]
Median [IQR] insulin rate (U/h)	3.0 [2.0 4.0]	2.5 [2.0 3.0]
Median [IQR] dextrose rate (g/h)	7.0 [4.7 8.2]	9.8 [8.6 11.5]
%BG in 80–145 mg/dL	89	55
%BG in 145–180 mg/dL	9	31
%BG in 100–150 mg/dL	83	54
%BG > 180 mg/dL	2	13
%BG < 80 mg/dL	0.5	1
%BG < 72 mg/dL	0.5	0.5
%BG < 40 mg/dL	0	0

Statistics are computed on hourly resampled BG where appropriate

### P-215 Gastrectomy in intensive care: prognostic factors

#### Adnane Janati, Amine Raja, Aziz Bouhouri

##### CHU IBN Rochd, Casablanca, Morocco

###### **Correspondence:** Adnane Janati (adnanjanati@gmail.com)

*Ann. Intensive Care* 2020, **10(Suppl 1):**P-215

**Rationale:** Although its incidence has declined in recent years, gastric cancer remains common worldwide and is the leading cause of gastrectomy. His treatment is mainly surgical, but his prognosis remains poor. Many studies on survival and prognostic factors have been carried out in foreign series.

**Patients and methods:** This is a retrospective study covering a period of three years from January 2014 to December 2016 interesting patients who had a gastrectomy and hospitalized in emergency resuscitation department surgical UHC Ibnou Rochd from Casablanca. The statistical analysis of the different clinical, paraclinical and therapeutic data was carried out thanks to an exploitation sheet.

**Results:** The mean age in our series was 54.09 years with a male predominance (sex ratio = 2.14). The main revealing symptoms were epigastralgia, weight loss and vomiting. Subtotal gastrectomy was performed in 40.9% of cases and total gastrectomy in 45.45% of cases. Curative resection could only be performed in 86.36% of cases. Operative mortality was 31.81% and morbidity was 22.71%. The main factor influencing operative mortality was age greater than 70 years. In univariate analysis the main prognostic factors; tumor size, degree of parietal invasion, presence of ganglionic invasion, presence of more than 3 ganglia invaded, presence of metastases, locally advanced tumor, tumor stage and curative nature of resection. Patient-related factors such as age associated blemishes and biological factors have a significant influence on the patient’s prognosis.

**Conclusion:** The prognosis of gastrectomies, although it has improved overall, remains mediocre. The only way to improve the prognosis remains the early diagnosis with an effective surgical management and the introduction of an adapted resuscitation.

**Compliance with ethics regulations:** Yes.

### P-216 Efficacy of multiple second line agents in refractory status epilepticus in a pediatric intensive care unit

#### Lea Savary, Claire Le Reun

##### CHU Tours, Tours, France

###### **Correspondence:** Lea Savary (lea.savary@hotmail.com)

*Ann. Intensive Care* 2020, **10(Suppl 1):**P-216

**Rationale:** Convulsive status epilepticus (CSE) is the most common neurological emergency in children. Refractory status epilepticus (RSE) occurs when seizures are not controlled with first- and second-line agents. In adults, RSE requires pharmacological induced coma. In pediatric patients, association of second line treatment is often used to avoid general anesthesia although there is currently no data on the efficacy of this association.

**Patients and methods:** We performed a monocentric retrospective study to assess the efficacy of multiple second line agents in pediatric RSE. All children admitted to Clocheville hospital (Tours) between January 2013 and December 2017 with a diagnosis of RSE were included. Our population was divided into two groups: need of general anesthesia (midazolam+) or not (midazolam-).

**Results:** 55 children were included (30 in group midazolam+, 25 in group midazolam−) during the study period. Among the 48 patients with multiple second line agents, 52% did not need general anesthesia (n = 25). In group midazolam+, CSE was 20% longer in patients treated with multiple second line agents (34.5 h vs. 27.8 h).

**Conclusion:** Multiple second line agent treatment is efficient to avoid general anesthesia in 50% of children. It would be interesting to identify predictors of general anesthesia.

**Compliance with ethics regulations:** Yes.

### P-217 Management of drowning in pediatric intensive care

#### Samira Kalouch, Wissal Aissaoui, Khalid Yakini, Abdelaziz Chlilek

##### Chu Ibn Rochd Casablanca, Casablanca, Morocco

###### **Correspondence:** Samira Kalouch (dr.kalouch@gmail.com)

*Ann. Intensive Care* 2020, **10(Suppl 1):**P-217

**Rationale:** Drowning is an acute respiratory failure resulting from immersion or submersion in a liquid.

**Patients and methods:** We report 8 cases of drowning collated in the pediatric reanimation department during a period from 2010 to 2016. The aim of our retrospective study was to analyze and compare the different epidemiological, clinical, parcalinical, therapeutic and evolutionary of drowning in our study.

**Results:** Our study contains 6 boys and 2 girls, with a sex ratio (M/F) of 3, in an age between 9 months and 8 years. For cases studied, no one was classified stage I, 37.5% classified stage II, 25% stage III, and 37.5% stage IV. All cases collected by ou service were victim of accidental drowning, 87.5% were secondary to the lack of parental supervision. Among 8 cases, 6 had respiratory complications, 4 cases of hydroelectrolytic disorders, 1 case with infectious complications, 3 cases of neurological and cases of cardiac or hypothermic complication. In our study, 5 cases recovered well and 3 cases died.

**Conclusion:** The survival of the drowned person depends on the speed and efficiency of the intervention, which in the first place is prehospital, thus ensuring the first actions at the scene of the accident, which will have repercussions on the hospital care. This has an equal share in the improvement of the victim’s prognosis.

**Compliance with ethics regulations:** Not applicable.

### P-218 Epidemiology of severe pediatric trauma following winter sport accidents in the Northern French Alps

#### Emilien Maisonneuve^1^, Nadia Roumeliotis^2^, Pierre Bouzat^1^, Guillaume Mortamet^1^

##### ^1^CHU Grenoble, Grenoble, France; ^2^CHU Sainte-Justine, Montréal, Canada

###### **Correspondence:** Emilien Maisonneuve (emilienmaisonneuve@orange.fr)

*Ann. Intensive Care* 2020, **10(Suppl 1):**P-218

**Rationale:** This study describes the epidemiology of severe injuries related to winter sports (skiing, snowboarding and sledding) in children, and assesses potential preventive actions.

**Patients and methods:** We did a single-center retrospective study in our pediatric Intensive Care Unit in the French Alps. We include all patients less than 15 years old, admitted to the Intensive Care Unit following a skiing, snowboarding or sledding accident from 2011 to 2018.

**Results:** We included 186 patients (mean age 10.5 years and 68% were male); of which 136 (73%), 21 (11%) and 29 (16%) had skiing, snowboarding and sledding accidents, respectively. The average ISS (injury severity score) was 16. The major lesions were head (n = 94 patients, 51%) and intra-abdominal (n = 56 patients, 30%) injuries. Compared to skiing and snowboarding, sledding accidents affected younger children (7 vs. 11 years, p < 0.001); most of whom did not wear a helmet (90% vs. 8%, p < 0.001). Severity scores were similar amongst winter sports (ISS = 17 for skiing, 11 for snowboarding and 17 for sledding accident, p = 0.02).

**Conclusion:** Winter sports can cause severe trauma in children. Sledding accidents affect younger children that may benefit from wearing protective equipment.

**Compliance with ethics regulations:** Yes.

### P-219 Severe traumatic brain injury in pediatric intensive care unit: study of management practices in French-speaking pediatric intensive care units. PTBIS Protocol-Pediatric Traumatic Brain Injury Survey

#### Manon Denis^1^, Benjamin Lauzier^2^, Etienne Javouhey^3^, David Brossier^4^

##### ^1^Department of Pediatrics, Caen University Hospital, Caen, France; ^2^L’institut du thorax, INSERM, CNRS, UNIV Nantes, Nantes, France; ^3^Pediatric Intensive Care Unit, Lyon University Hospital, Bron, Lyon, France; ^4^Pediatric Intensive Care Unit, Caen University Hospital, Caen, France

###### **Correspondence:** Manon Denis (denis.manon89@gmail.com)

*Ann. Intensive Care* 2020, **10(Suppl 1):**P-219

**Rationale:** Best strategies for the management of severe pediatric traumatic brain injury (TBI) are still not clearly established and wide variations among professional practices have been reported in the literature. Unfortunately, these variations in practice have an impact on the patient’s outcome. The objectives of this work were to assess the adequacy of professional practices to the guidelines for the management of severe head injury 2019 and to assess the level of agreement of respondents in the absence of guideline.

**Patients and methods:** A practice survey was conducted in French-speaking hospitals in Canada, Belgium, Switzerland and France from April 1st to June 30th, 2019. The survey was conducted as a progressive clinical case with 70 questions based on guidelines 2012 and the literature from 2012 to 2019. The questions related to the assessment and management of TBI during the acute and intensive care phase.

**Results:** Seventy-eight questionnaires were included. The adherence to guidelines 2019 was good, with 11 items out of 15 obtaining an adherence rate of more than 60% regardless of the annual number of TBI managed by the centre. There was strong agreement among clinicians on the intracranial pressure (PIC) (> 80%) and cerebral perfusion pressure (> 70%) thresholds used according to age. Guidelines for indication of PIC monitoring were almost perfectly followed in the case of Glasgow score < 8 and abnormal brain CT scan (n = 73, 93%). On the other hand, the natremia and glycemia thresholds and the role of transcranial doppler were not consistent. Strong adherence to recent recommendations was achieved: seizure prophylaxis with levitracetam (n = 21/33, 64%) and capnia threshold (n = 52, 67%). Assessment of O_2_ pressure in brain tissue (n = 12, 16%) and autoregulation (n = 35; 45%) was not a common practice.

**Conclusion:** Overall, practices for the management of TBI appear to be standardised. Variations persist in areas where there is a lack of literature and guidelines in paediatrics, so clinicians seem to refer to adult guidelines.

**Compliance with ethics regulations:** Yes.

### P-220 Ingestion of a button cell in children

#### Choubeila Guetteche

##### CHU Constantine, Constantine, Algeria

###### **Correspondence:** Choubeila Guetteche (cguetteche@gmail.com)

*Ann. Intensive Care* 2020, **10(Suppl 1):**P-220

**Rationale:** Ingesting a coin cell is a common household accident in children, which can have serious consequences. The goal is to determine prognostic factors to improve management and reduce complications.

**Patients and methods:** We conducted a retrospective study including children under 15 admitted in pediatric intensive care between January 2014 and May 2019 for ingestion of button cells, with epidemiological, clinical and paraclinical data collection.

**Results:** Twenty-six children 17 boys (65%), and 9 girls (35%) were included, with an average age of 28 months (10–120), increased incidence in recent years. Clinical signs indicative were dysphasia with hyper-sialorrhea in 24 cases, cervical pain in one case, respiratory distress in one case, the cell was located in the upper third of the esophagus in 18 cases, third average in 6 cases, third inferior in 2 cases, the mean time before extraction was 20 h. Complications: 2 cases of mediastinitis, 2 cases of oesotracheal fistula, a case of perforation.

**Conclusion:** The young age of the child, the diameter of the battery, and especially the time of care are risk factors for the occurrence of complications, the prevention passes through the education of the general public and creation of channel of taking into account fast charge.

**Compliance with ethics regulations:** Not applicable.

### P-221 Anesthesia for extraction of foreign bodies from the airways in children

#### Yacine Benhocine

##### University Hospital Center Nedir Mohamed, Tizi-Ouzou, Algeria

###### **Correspondence:** Yacine Benhocine (yacine001@yahoo.fr)

*Ann. Intensive Care* 2020, **10(Suppl 1):**P-221

**Rationale:** Inhalation of foreign bodies is a common and serious accident in children, especially between 1 and 3 years old. At this age, children use their mouth to explore their environment. Asphyxia is the immediate risk and respiratory sequelae may appear secondarily. The severity of this incident has been considerably reduced due to the progress of the instrumentation and anesthesia which condition the smooth running of the therapeutic act. Aim: to evaluate the anesthetic modalities of the extraction of the foreign bodies of the airways in children, in order to optimize our care with a maximum of security.

**Patients and methods:** A prospective, mono-centric, descriptive study from January 2011 to November 2013 of 128 patients treated for inhalation of foreign bodies in the airways. Study population wasdefined by: age, sex, hospitalization context, physical and radiological examination data, anesthetic management.

**Results:** The average age of the patients was 39.4 months, the male predominated (81%), and the hospitalization context was polymorphic. General anesthesia was necessary in all cases, sevoflurane mainly for narcosis; the combination of an opioid in 70.8% of cases and a curare in 8.6%. Spontaneous ventilation is desirable, but 79% was manually broken down intermittently between extraction attempts. Cases of desaturation, bronchospasm, bradycardia, and pneumothorax have been reported. 86.71% had a good evolution.

**Discussion:** The results of the epidemiological data are consistent with those of the literature. The penetration syndrome is very revealing. The chest x-ray is the key examination, the diagnosis is often based on indirect signs. In case of asphyxia by foreign body enclosed above or between the vocal cords, laryngoscopy and oxygenation is the first step to perform. In other cases, a rigid bronchoscopy is performed under general anesthesia; Inhalation induction with sevoflurane is the technique of choice for many experienced authors. Controlled ventilation is used in the majority of cases because spontaneous ventilation is not often not possible. The heterogeneity of anesthetic practices accounts for the multiplicity of clinical situations.

**Conclusion:** The inhalation of a foreign body is a diagnostic and therapeutic emergency. Extraction of the foreign body takes place under general anesthesia, which is difficult and at risk.

**Compliance with ethics regulations:** Yes.

### P-222 Non-invasive neurally adjusted ventilatory assist (NAVA) in infants with bronchiolitis: a retrospective cohort study

#### Alex Lepage-Farrell, Sally Al Omar, Atsushi Kawaguchi, Sandrine Essouri, Philippe Jouvet, Guillaume Emeriaud

##### CHU Sainte Justine, Université de Montréal, Montréal, Canada

###### **Correspondence:** Alex Lepage-Farrell (alex.lepage-farrell@umontreal.ca)

*Ann. Intensive Care* 2020, **10(Suppl 1):**P-222

**Rationale:** Bronchiolitis is one main reason for admission to pediatric intensive care unit. Most infants are successfully managed with nasal CPAP or high-flow nasal cannula, but about a third of these patients are not sufficiently supported and require an alternative support. Non-invasive neurally adjusted ventilatory assist (NIV-NAVA) improves patient-ventilator interactions and could therefore improve the effectiveness of non-invasive support. Our hypothesis is that NIV-NAVA is feasible in infants with bronchiolitis and that it reduces the respiratory effort.

**Patients and methods:** We retrospectively studied all patients under 2 years of age with a clinical diagnosis of bronchiolitis ventilated with NIV-NAVA in our pediatric intensive care unit, between October 2016 and June 2018. Patients characteristics, respiratory and physiologic parameters, including diaphragmatic electrical activity (Edi) were extracted from an electronic medical database (data collected every 30 s). Respiratory effort was estimated using the modified Wood Clinical Score for Asthma (mWCAS) and the inspiratory peak Edi, and 2-h periods before and after NIV-NAVA initiation were compared (Wilcoxon rank test). The study was approved by the local research ethics committee.

**Results:** During the study period, 205 patients were admitted with bronchiolitis; 64 infants (36 boys) with a median (25th–75th percentile) age of 52 (32–92) days were treated with NIV-NAVA after a failure of other non-invasive support methods, and all were included. Twenty-five subjects (39%) had at least one comorbidity. The interfaces used were predominantly face masks (92%). The maximum ventilatory settings were NAVA level of 1.0 (0.8–1.0), PEEP of 7 (7–8) cmH_2_O, FiO_2_ of 60% (39–100) and maximal pressure of 20 (20–22) cmH_2_O. Total duration of non-invasive ventilation was 70 (53–133) hours, including 48 (29–76) hours in NIV-NAVA. As detailed in the Table 1, mWCAS significantly decreased after NIV-NAVA initiation, from 3.0 (2.5–3.5) to 2.5 (2.0–3.0), p < 0.01. A decrease in inspiratory peak Edi was also observed, which was particularly clinically relevant in infants with high baseline Edi (> 20mcV). Capillary blood pH and pCO2 also significantly improved after NIV-NAVA introduction. Six patients (9%) needed escalation to endotracheal intubation.

**Conclusion:** This study confirms the feasibility of NIV-NAVA in infants with bronchiolitis after failure of first line non-invasive support, with a low failure rate. NIV-NAVA initiation was followed by a decrease in respiratory effort and an improvement in blood gases. This observational study supports the needs for prospective interventional trial.

**Compliance with ethics regulations:** Yes.

**Table 1:** Cardio-respiratory variables of 64 infants with bronchiolitis in the two-hour periods preceding and following NIV-NAVA initiation

Variables	*n*	2 h before NIV-NAVA	2 h after NIV-NAVA	*P*
mWCAS				
All patients	47	3.0 (2.75–3.5)	2.5 (2.0–3.0)	< 0.001
When baseline Edi < 20	20	3.0 (2.5–3.25)	2.5 (2.0–2.5)	< 0.01
When baseline Edi > 20	21	3.5 (2.5–3.6)	2.5 (2.0–3.0)	< 0.01
Inspiratory Edi, μV				
All patients	57	21.4 (9.9–45.9)	20.1 (16.6–32.3)	< 0.05
When baseline Edi < 20	27	9.8 (5.1–14.8)	11.6 (6.8–19.2)	0.18
When baseline Edi > 20	30	43.7 (32.1–54.6)	29.1 (19.8–36.8)	< 0.01
Vital signs				
Heart rate	62	149 (141–160)	144 (133–157)	< 0.001
Respiratory rate	62	43 (35–48)	41 (33–46)	0.05
Oxygenation				
FiO_2_, %	59	35 (25–40)	30 (28–45)	0.22
SpO_2_, %	62	98 (96–100)	98 (97–99)	0.15
SpO_2_/FiO_2_ index	59	2.8 (2.0–3.8)	3.2 (2.2–3.5)	0.06
Capillary blood gas				
pH	24	7.27 (7.24–7.30)	7.32 (7.29–7.34)	< 0.001
CO_2_	24	63 (53–69)	57 (47–64)	0.002
HCO_3_	24	28 (24–31)	28 (26–30)	0.59
FLACC Score	18	1.5 (0.0–3.0)	0 (0.0–2.0)	0.61

### P-223 State of transfusion practices at the pediatric intensive care unit Canastel, Oran, Algeria

#### Amel Zerhouni^1^, Wahiba Djebbari^2^, Lahcen Senhadji^1^, Nabil Tabet-Aoul^3^, Nabil Aouffen^3^, Amine Benhamed^1^

##### ^1^CHUO, Oran, Algeria; ^2^Faculté de médecine Sidi Bel Abbes, Sidi Bel Abbes, Algeria; ^3^Ehs CANASTEL, Oran, Algeria

###### **Correspondence:** Amel Zerhouni (azerhouni2000@yahoo.fr)

*Ann. Intensive Care* 2020, **10(Suppl 1):**P-223

**Rationale:** The use of blood transfusion is frequent in pediatric intensive care units and has increased significantly since 2002. Considered as therapeutic, it requires an assessment of the benefit / risk balance before making the transfusion decision. The aim of our study is to describe the transfusion practices in the Pediatric Resuscitation Department of the EHS Canastel, Algeria.

**Patients and methods:** A retrospective observational study over a 6-month period from January 2018 of any blood transfusion performed in hospitalized patients, in the pediatric intensive care unit. We studied : the age, the sex, the history of blood transfusion, the indication of transfusion, the haemodynamic and respiratory parameters, the transfusional accidents, the length of stay in intensive care, the evolution after a blood transfusion.

**Results:** These included 45 transfusion patients out of 135 hospitalizations during the 6-month period, mean age was 38 months.All patients had no transfusion history, 30% of patients had their anemia admission and 70% developed it during their stay. the reason for hospitalization was respiratory distress in 40%, convulsive condition in 30%, polytrauma in 10%, and head trauma in 20%. The indication of the transfusion was placed on a Hb inferior or equal to 7 g / dl in 50% of cases, in 30% on an Hb superior to 7 g / dl in addition to the clinical criteria of intolerance to anemia; in 20% of the cases no clinical or biological criteria found, the nature of the blood products was of the red cell in 77% of the cases and of the plasma concentrate in 13/100 of the cases and PFC in 10%. 33% received A+, 6% of A-, 15% of B+, 35% of O+ and 9% of O-. 20% of the patients had a transfusion-like reaction at 5 min after the start of the transfusion; 51% of the patients were under artificial ventilation and 29% were under hemodynamic support, 13% under diuretic.The average length of stay was 33 days; the favorable outcome was 35% of the patients after the transfusion with an increase in the Hb level beginning, 29% of the patients had complications of their pathology and the death in 33% of the cases.

**Conclusion:** Current transfusion practices in children often do not reflect the implementation of our current knowledge of the need for transfusion. Hence the need to review the protocols and practice other transfusion alternatives to avoid complications and improve the quality of care.

**Compliance with ethics regulations:** Not applicable.

### P-224 Antibiotic resistance of nosocomial infections in an intensive care unit

#### Sabah Benhamza, Mohamed Lazraq, Youssef Miloudi, Abdelhak Bensaid, Najib El Harrar

##### Réanimation de l’hôpital 20 Août, CHU Ibn Rochd, Casablanca, Morocco

###### **Correspondence:** Sabah Benhamza (benhamzasabah5@gmail.com)

*Ann. Intensive Care* 2020, **10(Suppl 1):**P-224

**Rationale:** Bacterial multi drug resistance is medical actuality nowadays, because of its morbidity and mortality especially in intensive care, it constitutes a real problem in our hospitals.

**Patients and methods:** We conducted a retrospective descriptive study, to identify bacterial drug resistance profile of patients with cross infections in the department of intensive care in 20 August hospital. This study included patients hospitalized between 1st January and 31st December 2017. The data was collected from medical records of this unit as from the register of the bacteriology service of Ibn Rochd university hospital.

**Results:** 468 patients were hospitalized in the resuscitation service, of which 98 had nosocomial infection, an incidence of 20.9%. The mean age of the patients was 51 years with male predominance (sex ratio 2.5), the average stay in intensive care was 17 days. The site of infection was pulmonary in 34% of cases, blood in 20% of cases, urinary in 18% of cases, central catheter in 13%, neuro-meningeal in 5.5% of cases. The germs isolated were: Acinetobacter baumanii in 30.5% of cases, Pseudomonas aeroginosa in 17.8% of cases, Klebsiella pneumonia in 12.9% of cases, Enterococcus feacalis in 9.4% of cases, E.coli in 8.1% of cases and Staphylococcus aureus in 7% of cases. Acinteobacter baumanii showed resistance rates of up to 90% for the impenem and 77% for amikacin. Regarding Pseudomonas, it was resistant to impenem in 57% of cases and in 46% of cases to amikacin. Compared to Klebsiella, resistance to imipenem was 52% and 2% for amikacin. The mortality rate of infected patients was 59%

**Conclusion:** In the light of this work, we found that important emergence of multidrug resistance bacteria in intensive care unit is related to not only the immunocompomised state of patients but also to daily bad practices of health professionals such as the misuse of antibiotics.

**Compliance with ethics regulations:** Yes.

### P-225 Impact of the Accelerate Pheno™ system for rapid antimicrobial susceptibility testing in intensive-care patients with Gram-negative bloodstream infections

#### Nabil Gastli^1^, Guillaume Savary^2^, Paul Jaubert^3^, Déborah Hirt^4^, Claire Poyart^1^, Jean-Paul Mira^3^, Helene Poupet^1^, Solen Kerneis^5^

##### ^1^Service de Bactériologie, APHP.CUP, Site Cochin, Paris, France; ^2^Intensive, Paris, France; ^3^Medical Intensive Care Unit, Cochin Hospital, APHP.Centre–Université de Paris, Paris, France; ^4^Service de Pharmacologie, APHP.CUP, Site Cochin, Paris, France; ^5^Equipe Mobile d’Infectiologie, APHP.CUP, Site Cochin, Paris, France

###### **Correspondence:** Nabil Gastli (nabil.gastli@aphp.fr)

*Ann. Intensive Care* 2020, **10(Suppl 1):**P-225

**Rationale:** Gram-negative bloodstream infections (GNBSI) require timely appropriate antimicrobial therapy in intensive care units (ICU) patients. Conventional techniques usually take 24–72 h for antimicrobial susceptibility testing (AST). Innovative approaches (Accelerate Pheno™ system) provide pathogen identification in ~2 h and AST including Minimal Inhibitory Concentrations (MICs) in ~7 h. We report, in ICU patients with GNBSI, results of implementation of the Accelerate Pheno™ in our laboratory.

**Patients and methods:** We prospectively screened all GNBSI episodes reported in adult ICU patients between September 2018 and September 2019. To allow integration into the laboratory workflow, the Accelerate Pheno™ was run on blood bottles positive before 10 am (Day 0), in parallel with routine procedures: MALDI-TOF identification after short incubation on solid media (Day 0), β LACTA (Bio-Rad^®^) test (Day 0) and disk diffusion method for AST (Day+1). For each episode, antimicrobial regimen was reassessed by a multidisciplinary team of bacteriologists, infectious diseases and ICU physicians by the end of Day 0. We measured: (i) Concordance of Accelerate Pheno™ results with conventional techniques, (ii) Number of antibiotic adaptations on Day 0 and (iii) Number of patients within the therapeutic range (free fraction over 4 x MIC and below concentration at risk of adverse events), based on real-time measurement of beta-lactams concentrations.

**Results:** Of 67 patients reported with GNBSI over the study period, 26 were included. Mean age was of 60 ± 16.5 years, 15/26 were males. Main sources of GNBSI were pulmonary (n = 8) and digestive (n = 7). Bacterial identification of the Accelerate Pheno™ was concordant with standard techniques in 22 (85%): Enterobacteriacae (n = 18), Pseudomonas aeruginosa (n = 4). Overall categorical agreement for AST was of 96% (15 errors including 6 very major errors). By the end of Day 0, the antibiotic regimen was de-escalated in 7 (27%) patients, which was appropriate in 6 (23%). In 2 cases, de-escalation was possible, but not fulfilled by ICU physicians. Twenty patients had beta-lactams concentrations measurements: 12 were in the therapeutic range, 2 below and 6 over.

**Conclusion:** Accelerate Pheno™ provided rapid and accurate results for most microorganisms isolated in blood cultures of ICU patients with GNBSI. However, in a laboratory with routine MALDI-TOF early identification and β LACTA test performed on Day 0, the impact on early adaptation of the antibiotic regimen was evident in around 1 patient over 4.

**Compliance with ethics regulations:** Not applicable.

### P-226 Use of Accelerate PhenoTest Blood Culture Kit for rapid microbiological documentation (90 min) and antibiotic susceptibility testing (+5 h) of positive blood cultures in the ICU and hematology units : an observational study

#### Jean-Luc Baudel^1^, Jacques Tankovic^1^, Redouane Dahoumane^1^, Jean-Remy Lavillegrand^2^, Razach Abdallah^2^, Geoffroy Hariri^2^, Naike Bige^2^, Hafid Ait-Oufella^2^, Nicolas Veziris^1^, Eric Maury^2^, Bertrand Guidet^2^

##### ^1^Service Bactériologie, Hôpital Saint-Antoine, Paris, France; ^2^Service Réanimation Médicale, Hôpital Saint-Antoine, Paris, France

###### **Correspondence:** Jean-Luc Baudel (jean-luc.baudel@aphp.fr)

*Ann. Intensive Care* 2020, **10(Suppl 1):**P-226

**Rationale:** Evaluation of the accurateness of the Accelerate PhenoTest BC Kit for rapid analysis (1.5 h for microorganism identification and 5 additional hours for antibiotic susceptibility testing) of positive blood cultures from ICU and hematology patients.

**Patients and methods:** From February to August 2019, we included patients from the ICU and hematology units with positive blood cultures. The following informations were collected : gender, age, duration of prior antibiotherapy, source of the infection, results obtained by conventional microbiological methods and by PhenoTest (data obtained and time to obtention of results). Informed consent was obtained from all patients.

**Results:** 33 blood cultures were analyzed in 31 patients (m/f ratio 1.07, age 59.3±, 16 from the ICU and 15 from hematology). 57% of the patients were receiving antibiotics at the time of blood culture collection (mean duration : 7.9 days). The source of infection was unknown in 59% of cases, urinary in 21%, catheter-related in 10%, ascites in 6%, pneumonia in 4%. In 17 cases (51%), there was a perfect match between PhenoTest and conventional results (identification and antibiotic susceptibility testing). In 5 cases (15%), the bacterium responsible was not present in the PhenoTest panel. In 3 cases (9%), PhenoTest identification was correct, but some discrepancies were observed regarding antibiogram. In 4 cases (12%) PhenoTest identification was again correct but no antibiogram was available. In 2 cases (6%), where two bacteria were present, PhenoTest could not identify one of them. In 2 cases, PhenoTest did not provide bacterial identification because too few bacteria were present in the blood culture bottle.

**Conclusion:** The PhenoTest panel covered 85% of the bacteria implicated in this study. When the bacterium responsible was present in the panel, the results given by the PhenoTest correlated in 70% of cases with those of conventional methods. Some rare discrepancies were observed regarding antibiotic susceptibility testing that have to be analyzed further. In the remaining 30% of cases, where too few bacteria or two different bacteria were present in the blood culture bottle, technical limitations did not permit to correctly identify microorganism(s) present or to obtain an antibiogram.

**Compliance with ethics regulations:** Yes.

### P-227 Bacterial lung microbiota under mechanical ventilation a pilot study

#### Mélanie Fromentin, Antoine Bridier-Nahmias, Constance Vuillard, Jean-Damien Ricard, Damien Roux

##### INSERM UMR 1137 IAME Infection Antimicrobials Modelling Evolution, Paris, France

###### **Correspondence:** Mélanie Fromentin (mel.fromentin@wanadoo.fr)

*Ann. Intensive Care* 2020, **10(Suppl 1):**P-227

**Rationale:** Studying human lower respiratory tract microbiota by using NGS (new generation sequencing) method is complex because of many unexpected biases due to DNA extraction and amplification procedures. Lung microbiota evolution under mechanical ventilation evolution may be highly informative to evaluate the actual risk of VAP (ventilator-associated pneumonia) development. Before starting a large study on the lung microbiome of ventilated ICU patients, a methodological study was mandatory.

**Patients and methods:** Five control and three VAP patients were selected. Endotrachealaspirate (ETA) and oropharyngeal swab (OS) were collected at ICU admission for control patients and, 6 days before and on the day of VAP diagnosis for VAP patients. After automated extraction of total DNA, hypervariable region V4 of the 16S rDNA genes was amplified with two different pairs of primers 515F-806R: oligonucleotides from the Earth microbiome project (earth primer pair) and from the Gut microbiome project (gut primer pair), followed by sequencing on Illumina MiSeq plateform. After bioinformatics analysis with Mothur^®^ software, we compared the performance of NGS alongside conventional bacterial culture. Differences in alpha diversity (microbial diversity in a sample), expressed as the Shannon index, across respiratory tract site (upper or lower) and across time (before and at VAP time) has been investigated. A positive control (PC), overnight culture of *Escherichia coli*, *Klebsiella pneumoniae*, *Staphylococcus aureus* and *Pseudomonas aeruginosa*, was also sequenced.

**Results:** Twenty-four samples and the PC were analyzed. Amplicon sequence analyses found similar results with the two primer pairs in 58% of cases. Cultured pathogen was found in 89% (8/9) for Human primer pair and in 44% (4/9) for Earth primer pair. For each ETA, NGS revealed bacteria unknown as pathogen globally identified as oropharyngeal flora in conventional microbiology (Table 1). Alpha diversity decreased for all VAP patients overtime, average Shannon 2.5 (2; 2.8) versus 2 (1.6; 2.6), and was higher in upper respiratory tract (OS) versus lower respiratory tract (ETA): average Shannon 2.5 (1.7; 2.7) vs. 1.8 (0.7; 2.6) (NS).

**Conclusion:** This pilot study highlights the impact of 16S rDNA amplification procedures (especially oligonucleotide sequences) used on the results in microbiome research. Concordance between NGS and bacterial culture, as well as similar evolution of the alpha diversity than previously described (1), enables us to validate our methodology using the “gut primers” pair 515F-806R. These findings allow furthers major studies on the pulmonary microbiome of ICU ventilated patients including comparison according to the occurrence of a VAP or not.

**Compliance with ethics regulations:** Yes.

**Table 1:**
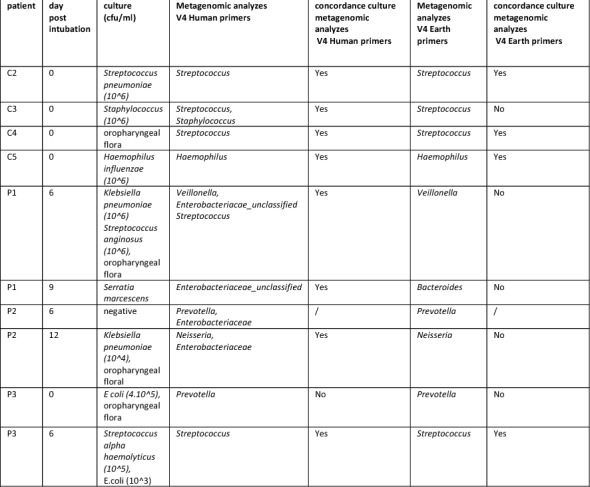
Comparison between NGS and culture results for endotracheal aspirates

### P-228 Lung bacterial microbiome and virome in intensive care a pilot study

#### Mélanie Fromentin^1^, Jean-Damien Ricard^1^, Julien Do Vale^1^, Antoine Bridier-Nahmias^1^, Severine Mercier^2^, Noemie Ranger^3^, Antonio Alberdi^4^, Damien Roux^1^

##### ^1^Inserm UMR 1137 Iame “Infection Antimicrobials Modelling Evolution” University of Paris, Paris, France; ^2^”INSERM U 976 HIPI “Human Immunology, Paris, France; ^3^Inserm UMR 976 HIPI “Human Immunology, Pathophysiology & Immunotherapy” University of Paris, Paris, France; ^4^Plateforme technologique Institut de Recherche Saint Louis, Université de Paris, Paris, France

###### **Correspondence:** Mélanie Fromentin (mel.fromentin@wanadoo.fr)

*Ann. Intensive Care* 2020, **10(Suppl 1):**P-228

**Rationale:** In the field of intensive care only few studies have explored bacterial microbiota whereas virome remained hardly considered. It appears essential to describe both evolution in mechanically-ventilated patients to improve the pathophysiological understanding of ventilator-associated pneumonia (VAP) development. To date no study had been simultaneously conducted on lower respiratory tract with a single nucleic acid extraction before metagenomics analysis of bacterial microbiota and virome. We conducted a preliminary study to validate our methodology based on a common automated extraction of nucleic acids.

**Patients and methods:** Twelve mechanically ventilated patients were selected: five who developped (VAP) and seven controls (C) who did not. Endotracheal aspirate (ETA) were collected between intubation and day 12 (or DVAP for VAP patients). Conventional bacterial microbiology and multiplex respiratory viruses PCR were also performed. Total nucleic acids were extracted using NucliSENS easyMag extractor. For the bacterial microbiota, region V4 of the 16S rRNA genes was amplified. For the virome, the Nextera DNA XT kit (Illumina) and RNA Seq Trio kit (Nugen) protocols were used to prepare viral DNA and RNA libraries. Libraries underwent paired-end sequencing on the Illumina Miseq (bacteria) or NextSeq-500 (virus) platform. After bioinformatics analysis we compared the performance of metagenomics analysis with conventional bacterial culture and other common viral detection methods.

**Results:** For culturable bacteria, concordance between conventional microbiology and sequencing was found in 89% (8/9). After 16S sequencing 73,046 (36,243; 154,186) median reads per sample were analyzed. 16s sequencing also identified bacteria defined as oropharyngeal flora in conventional microbiology. Two predominant phylum, Bacteroidetes and Firmicutes, and three genus Streptococcus, Veillonella, Prevotella were identified. After viral DNA and RNA libraries sequencing, 55,316,004 (52,726,119; 68,629,611) median reads per sample were analyzed. Virome analysis identified three major virus families: Paramyxoviridae, Orthomyxoviridae and Herpesviridae. Results of NGS had 83,3% concordance with multiplex PCRFilmarray or targeted PCR. This method revealed viruses unexplored by targeted PCR, especially HSV1 and bacteriophages. Concordance results are presented in Table 1.

**Conclusion:** Our preliminary results confirm the feasability of exploring both bacterial microbiota and virome on the same sample using a common extraction method. Data from metagenomics were highly concordant with conventionnal detection methods for known pathogenic viruses and bacteria in lower tract respiratory sample and enables identification of other microorganisms. This is the first step for a large cohort study that aims to compare evolution of global lung microbiome in patients at risk of VAP and assess how bacteria and virus interplay.

**Compliance with ethics regulations:** Yes.

**Table 1:**
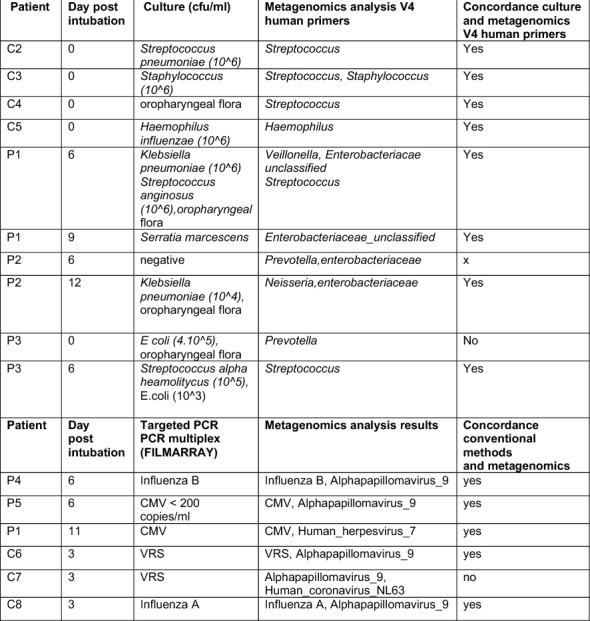
Concordance between conventional methods and NGS

### P-229 Current state of colistin resistance in a burn intensive care unit in Tunisia

#### Sonia Ben Behi^1^, Sarra Dhraief^1^, Amel Mokline^2^, Lilya Debbiche^2^, Amenallah Messaadi^2^, Lamia Thabet^1^

##### ^1^Centre de Traumatologie et des Grands Brûlés de Ben Arous-Laboratoire de biologie médicale et banque du sang, Ben Arous, Tunisia; ^2^Centre de Traumatologie et des Grands Brûlés de Ben Arous-Service de réanimation des brûlés, Ben Arous, Tunisia

###### **Correspondence:** Sonia Ben Behi (benbehi.sonya@gmail.com)

*Ann. Intensive Care* 2020, **10(Suppl 1):**P-229

**Rationale:** Colistin is used as a last-line treatment to combat multidrug-resistant (MDR) Gram-negative bacilli (GNB). Worryingly, colistin resistance in *Klebsiella pneumoniae*, *Pseudomonas aeruginosa* and *Acinetobacter baumannii* is increasingly reported worldwide. We hereby report the prevalence of colistin resistance among GNB isolated from burn patients in Tunisia.

**Patients and methods:** The study was carried out on 225 strains of GNB isolated from microbiological samples of burn patients hospitalized in the intensive care unit between October 2017 and December 2018. Identification was performed by conventional methods. Antimicrobial susceptibility was tested by disk diffusion method and the results were interpreted according to CA-SFM guidelines. Minimum inhibitory concentration (MIC) of colistin was determined using the EUCAST broth micro-dilution method (UMIC, Biocentric^®^)

**Results:** *Pseudomonas aeruginosa* was the most frequently isolated bacteria (111 strains), followed by *Acinetobacter baumannii* (99 strains) and *Klebsiella pneumoniae* (15 strains). The most common sites of isolation were blood cultures (45%), catheters (22%) and skin samples (21%). Most of *P. aeruginosa* isolates were multidrug-resistant with high levels of resistance to imipenem (71.7%), ceftazidime (63%) and ciprofloxacin (74.5%). However, all of them were susceptible to colistin. In fact, MICs of colistin against all *P.aeruginosa* isolates were less than or equal to 0.5 mg/L. *A. baumannii* strains had high resistance rates to beta-lactams : 100% to ceftazidime and 94% to imipenem. Only one strain was resistant to colistin with a MIC equal to 32 mg/L. All *K. pneumoniae* isolates were resistant to extended-spectrum cephalosporins. One third of these strains were resistant to imipenem and more than half (58.3%) were resistant to amikacin. Two strains were resistant to colistin with high MICs (> 64 mg/L). Both were carbapenemase-producers, carrying OXA-48 and NDM carbapenemase encoding genes.

**Conclusion:** These data suggest that colistin-resistant or pan-drug resistant GNB clinical isolates are still relatively rare. However, they have important global public health implications because of the therapeutic problems they present, especially for vulnerable populations such as severely burned patients. Hence the need to test colistin regularly in the laboratory and to set up a monitoring program for MDR pathogens.

**Compliance with ethics regulations:** Yes.

### P-230 Mediastinitis complicating cervico-facial cellulitis

#### Mohamed Lazraq, Sabah Benhamza, Youssef Miloudi, Abdelhak Bensaid, Najib Elharrar

##### Réanimation Hôpital 20 Août 1953, Casablanca, Morocco

###### **Correspondence:** Mohamed Lazraq (mohamed_lz@hotmail.com)

*Ann. Intensive Care* 2020, **10(Suppl 1):**P-230

**Rationale:** Descending necrotizing mediastinitis (DNM) are medico-surgical emergencies whose forecast is closely related to the precocity of the therapeutic assumption. The purpose of our work is to profile these patients as well as the therapeutic and evolutionary aspects.

**Patients and methods:** Retrospective study over 5 years in the intensive care unit of the hospital 20 August. All patients with DNM on cervicofacial cellulitis were included.

**Results:** 12 cases were collected, 17% of cellulitis, incidence of 2.4 patients / year. Average age 39, sex ratio of 1.4. Smoking, chronic alcoholism and diabetes are the most common antecedents. The favoring factors were: (poor dental conditions: 100% of cases, non steroidien anti-inflammatory drugs: 33%, diabetes: 25%). In 67% of cases the front door was dental. Average time taken to take care of 10 days. C-reactive protein and procalcitonin were positive in all patients. In 33% the chest x-ray was normal. All patients received tri-antibiotic therapy. Intubation were difficult in all patients, we used nasofibroscope in 25% of cases and a rescue tracheotomy in one patient. Only one patient had a cervico-thoracic surgical approach; for all the others she was cervical alone. Streptococcus was the most isolated germ. The complications were (septic shock: 33%, ARDS: 8%). The average hospital stay was 7 days with a mortality rate of 42%.

**Conclusion:** DNMs are poorly prognostic. The best treatment remains prevention by better management of dental abscesses and tonsillar phlegmons.

**Compliance with ethics regulations:** Yes.

### P-231 Early- and late-onset pneumonia: myth or reality?

#### Sana Kharrat, Hatem Ghadhoune, Jihene Guissouma, Sadok Bougharriou, Habib Brahmi, Meriem Ksouri

##### Hospital of Bizerte, University Elmanar Tunis, Faculty of Medecine Tunis, Bizerte, Tunisia

###### **Correspondence:** Sana Kharrat (sanakharrat15@hotmail.com)

*Ann. Intensive Care* 2020, **10(Suppl 1):**P-231

**Rationale:** The initial, empirical antibiotic therapy of ventilator-associated pneumonia (VAP) is often based on timing of its occurrence in relation to the onset of mechanical ventilation. This is due to reported differences between causal pathogens associated with early-onset (E-VAP < 5–7 days of mechanical ventilation) compared to late-onset VAP (L-VAP ≥ 5–7 days of MV). E-VAP is most often reported to be due to antibiotic-sensitive pathogens while L-VAP is frequently attributed to antibiotic-resistant pathogens. However, there is emerging evidence that the isolated microorganisms may be similar regardless of onset time. The aim of our study was to compare the clinical outcomes of critically ill patients developing E-VAP and L-VAP and to compare the causative pathogens of the two groups.

**Patients and methods:** All the patients with the diagnosis of VAP admitted between January 2014 and December 2018 were retrospectively included. VAP was suspected on the basis of clinical and chest x-ray findings. The identification of the causative organisms was performed with endotracheal aspirate (ETA) cultures.

**Results:** Ninety patients developed VAP. E-VAP was observed in 30 patients (33,3%), whereas 60 patients (66,6%) developed L-VAP. Among patients with early-onset VAP, 53% received antibiotics prior to the development of pneumonia, compared to 88% with late-onset VAP (p = 0.001). Otherwise, no differences (sociodemographic factors, antecedents, severity score, length of stay, length of MV) between the two groups were observed. The most common pathogens associated with E-VAP were Enterobacter species (26.6%), Pseudomonas aeruginosa (23.3%) and oxacillin-resistant *Staphylococcus aureus* (ORSA 13,3%). Enterobacter species (36.6%), *Acinetobacter baumannii* (26.6%) and *Pseudomonas aeruginosa* (25%) were the most common pathogens associated with L-VAP. No difference was noted in the contribution of multidrug resistant bacteria MDR (50% vs. 70%). Hospital mortality was significantly greater for patients with L-VAP caused by MDR (73%) compared to patients with E-VAP (50%) (p = 0.04).

**Conclusion:** This classification is no longer helpful for empirical antibiotic therapy, since both early-onset and late-onset VAP were caused by MDR bacteria. This justifies the need of intensive care unit-specific knowledge of causal agents associated with VAP to reduce the rate of administration of inadequate antimicrobial therapy.

**Compliance with ethics regulations:** Yes.

## Disclosure of conflicts of interest

- AARAB Yassir: Research support/Scientific studies : SuperSonic Imagine S.A. (Aix-en-Provence, France)
- ABARDAZZOU Abir: No disclosure
- ABBAS Haider: No conflict of interest
- ABDALLAH Razach: No disclosure
- ABDALLAH Sahar: No disclosure
- ABDEJABBAR Maghfour: No disclosure
- ABDEL-NABEY Moustafa: No conflict of interest
- ABDELLATIF Sami: No conflict of interest
- ABDENNEBI Cyrine: No conflict of interest
- ABID Selma: No conflict of interest
- ABIDI Ameni: No conflict of interest
- ABOAB Jérôme: No conflict of interest
- ABOUELHASSAN Taoufik: No conflict of interest
- ABOUQAL Redouane: No conflict of interest
- ABRAHAM Paul: No conflict of interest
- ABROUG Fekri: No conflict of interest
- ACHOURI Abderrahim: No conflict of interest
- ACHREF Laajili: No disclosure
- ADDA Melanie: No disclosure
- ADDA Mireille: No disclosure
- ADNANE Janati: No disclosure
- ADNOT Pauline: No conflict of interest
- ADRIE Christophe: No disclosure
- AEGERTER Philippe: No conflict of interest
- AGBAKOU Maïté: No conflict of interest
- AGERON Francois-Xavier: No conflict of interest
- AGUERSIF Amazigh: No conflict of interest
- AHO Serge: No disclosure
- AISSAOUI Nadia: Trainings, Teaching: ASTRA-ZENECA, MEDTRONIC; Invitation to national or international congresses: ASTRA-ZENECA, ABIOMED, THORATEC, MEDTRONIC
- AISSAOUI Wissal: No conflict of interest
- AIT-HAMOU Zakaria: No conflict of interest
- AIT-OUFELLA Hafid: No conflict of interest
- AKROUR Yanis: No conflict of interest
- AL HARRAR Rachid: No disclosure
- AL OMAR Sally: No conflict of interest
- ALAMOWITCH Sonia: No disclosure
- ALBERDI Antonio: No conflict of interest
- ALBUISSON Eliane: No conflict of interest
- ALI Deeba: No conflict of interest
- ALLALI William: No conflict of interest
- ALLOUACHE Christophe: No disclosure
- ALLOUCHE Hend: No conflict of interest
- ALVES Barbara: No conflict of interest
- AMALRIC Matthieu: Invitation to national or international congresses: Sanofi
- AMENGLE Albert Ludovic: No disclosure
- AMEYE Lieveke: No conflict of interest
- AMIEL Jean-Bernard: No conflict of interest
- AMIOT Aurélien: No conflict of interest
- AMMAR Wessem: No disclosure
- AMMAR ZAYANI Rania: No conflict of interest
- AMOURA Zahir: No disclosure
- AMOUYAL Elsa: No conflict of interest
- ANDRE Cécile: No disclosure
- ANGUEL Nadia: No disclosure
- ANIS Ben Haj Khalifa: No conflict of interest
- ANNANE Djillali: No conflict of interest
- ANOTA Amélie: Consultancy, Expert: Roche, Astrazeneca; Trainings, Teaching: BMS
- ANSELMI Luciano: No conflict of interest
- ANTONELLI Massimo: No disclosure
- AOUFFEN Nabil: No disclosure
- APPERT-DECLE Alexandra: No conflict of interest
- ARAFA Meher: No conflict of interest
- ARGAUD Laurent: No conflict of interest
- ARMAND-LEFREVRE Laurence: Trainings, Teaching: Biomerieux-non-paid internal symposium
- ARNAL Jean-Michel: Research support/Scientific studies: Hamilton Medical; Consultancy, Expert: Hamilton Medical, Resmed, Breas Medical
- AROICHANE Nadine: No conflict of interest
- ARRESTIER Romain: No conflict of interest
- ARTRU Florent: No disclosure
- ARTZNER Thierry: No conflict of interest
- ASEHNOUNE Karim: No disclosure
- ASFAR Pierre: Consultancy, Expert: Expert activity for Banque Publique d’Investissement (French government Agency)
- ASLANIAN Pierre: No conflict of interest
- AUBART Melodie: No conflict of interest
- AUBRON Cécile: Invitation to national or international congresses: Pfizer
- AUDIBERT Juliette: No conflict of interest
- AUGUSTO Jean-François: No conflict of interest
- AUGY Jean-Loup: No conflict of interest
- AXUS Marina: No disclosure
- AYARI Ahmed: No conflict of interest
- AYED Samia: No conflict of interest
- AZOULAY Elie: Research support/Scientific studies: Pfizer, Ablynx; Consultancy, Expert: Lectures for Baxter, Pfizer and Gilead; Invitation to national or international congresses: Gilead 2018
- BABOI Loredana: No disclosure
- BACCARI Amal: No conflict of interest
- BACCOUCHE Najeh: No conflict of interest
- BACCOUCHE Ramla: No conflict of interest
- BACHASSON Damien: Research support/Scientific studies: Fondation EDF, Association Française Contre les Myopathies; Patent or product inventor: Institut de Myologie, Paris, France
- BACHELLIER Philippe: No disclosure
- BADAOUI Karim: No conflict of interest
- BADER-MEUNIER Brigitte: Research support/Scientific studies: Novartis, Roche, Pfizer; Invitation to national or international congresses: Novartis
- BAGATE François: Invitation to national or international congresses: Orkyn
- BAHLOUL Mabrouk: No conflict of interest
- BAILLEUL Clotilde: No disclosure
- BAILLY Pierre: No conflict of interest
- BAISSE Arthur: No conflict of interest
- BALIK Martin: Trainings, Teaching: Fresenius Medical Care; Patent or product inventor: GML Czech Republic
- BANYDEEN Rishika: No conflict of interest
- BAPTISTE Amandine: No conflict of interest
- BAPTISTE Olivier: No conflict of interest
- BARBAR Saber Davide: No disclosure
- BARBIER François: No disclosure
- BARBIER Louise: Trainings, Teaching: Ethicon, Astellas; Invitation to national or international congresses: Sandoz, Astellas
- BARNERIAS Christine: No disclosure
- BARON Aurore: No disclosure
- BARON Elodie: No conflict of interest
- BARRATT-DUE Andreas: No disclosure
- BARRAU Stephanie: No disclosure
- BARRAUD Damien: No disclosure
- BARRAUD Helene: No disclosure
- BARROIS Brigitte: No conflict of interest
- BARUCHEL André: No disclosure
- BASTIDE Marie Anaïs: No conflict of interest
- BAUDEL Jean-Luc: No conflict of interest
- BAUDIN Florent: Invitation to national or international congresses: Dr Baudin has received speaking fees from Maquet Critical Care (EPNV, Montreux May 2016)
- BAUDRY Thomas: Trainings, Teaching: Gilead, Pfizer; Invitation to national or international congresses: Pfizer
- BAUER Philippe: No conflict of interest
- BAYAT Sam: Research support/Scientific studies: Fisher & Paykel
- BEAUCOURT Camille: Trainings, Teaching: Gallia 100 euros
- BEAUFILS Fabien: Consultancy, Expert: Astra Zeneca; Invitation to national or international congresses: Astra Zeneca
- BEDHAIFI Amir: No disclosure
- BEDOS Jean-Pierre: Consultancy, Expert: Pfizer/Correvio; Invitation to national or international congresses: Correvio
- BEDUNEAU Gaetan: Research support/Scientific studies: SRLF; Association Départementale d’Aide aux Insuffisants Respiratoires (76); Trainings, Teaching: Drager; Invitation to national or international congresses: MSD; Hill Rom
- BEGANTON Frankie: No conflict of interest
- BEGOT Erwan: No disclosure
- BEINSE Guillaume: Research support/Scientific studies: Association pour la Recherche contre le Cancer; La Ligue contre le Cancer; LeoPharma; Invitation to national or international congresses: Pharmamar
- BELAID Imtinene: No conflict of interest
- BELAYACHI Jihane: No conflict of interest
- BELE Nicolas: No conflict of interest
- BELHAJ YOUSSEF Sourour: No conflict of interest
- BELLIEN Jeremy: No disclosure
- BELLUT Hugo: No conflict of interest
- BELMEKADEM Said: No conflict of interest
- BELONCLE Francois: No conflict of interest
- BEN AHMED Hedia: No disclosure
- BEN AHMED Tarek: No conflict of interest
- BEN ALI Yosri: No conflict of interest
- BEN AMOR Wiem: No conflict of interest
- BEN BEHI Sonia: No conflict of interest
- BEN HADJ SALEM Omar: No conflict of interest
- BEN HAMIDA Chokri: No conflict of interest
- BEN ISMAIL Khaoula: No conflict of interest
- BEN JABALLAH Nejla: No conflict of interest
- BEN KHELIL Jalila: No conflict of interest
- BEN LAKHAL Salah: No conflict of interest
- BEN LAMINE Farah: No conflict of interest
- BEN ROMDHANE Kais: No disclosure
- BEN SAAD Manel: No conflict of interest
- BEN SAIDA Imen: No conflict of interest
- BEN SALAH Adel: No disclosure
- BEN SALAH Amina: No conflict of interest
- BEN SOUISSI Asma: No conflict of interest
- BEN ZARROUK Sarra: No disclosure
- BENABOUD Sihem: No disclosure
- BENAIS Morgan: No conflict of interest
- BENALI Hana: No conflict of interest
- BENARD Matthieu: No conflict of interest
- BENBERNOU Soumia: No conflict of interest
- BENETAZZO Lucie: No conflict of interest
- BENGHANEM Sarah: No conflict of interest
- BENGONO BENGONO Roddy Stephan: No conflict of interest
- BENHAMED Amine: No disclosure
- BENHAMZA Sabah: No conflict of interest
- BENHOCINE Yacine: No conflict of interest
- BENICHOU Nicolas: Research support/Scientific studies: SFNDT (société francophone de néphrologie dialyse et transplantation)—Master 2 scholarship
- BENIS James: No conflict of interest
- BENNETT Gavin: No disclosure
- BENOIT Dominique: Research support/Scientific studies: Gilead, Astellas, Fisher-Paykel, Baxter, Alexion and Fresenius Kabi
- BENSAID Abdelhak: No disclosure
- BENSARDI Fatimazahra: No disclosure
- BENYAMINA Mourad: No disclosure
- BENZERARA Laurent: Patent or product inventor: aphp
- BENZERDJEB Nazim: Research support/Scientific studies: AMARAPE, iCAP; Consultancy, Expert: ALPHASIGHTS, MSD; Trainings, Teaching: MSD
- BEQIRI Erta: No disclosure
- BÉRANGER Agathe: No conflict of interest
- BERARD Emilie: No conflict of interest
- BERDAI Adnane: No disclosure
- BERGER Patrick: No disclosure
- BERNAL William: No disclosure
- BERNARDIN Gilles: No disclosure
- BERRADA Lina: No conflict of interest
- BERTHAUD Romain: No conflict of interest
- BERTHET Guillaume: No conflict of interest
- BERTI Enora: No conflict of interest
- BERTOLI Sarah: No disclosure
- BERTRAND Pierre-Marie No conflict of interest
- BESBES Lamia: No disclosure
- BESBES Mohamed: No conflict of interest
- BESCH Camille: Invitation to national or international congresses: Abbvie, Novartis
- BESCH Guillaume: No conflict of interest
- BESNARD Noémie: No conflict of interest
- BESSET Sebastien: No disclosure
- BETTON Delphine: No conflict of interest
- BEURET Pascal: No conflict of interest
- BEURTON Alexandra: No conflict of interest
- BEUVON Clément: No conflict of interest
- BIEDERMANN Sebastien: Invitation to national or international congresses: Vifor Pharma
- BIGE Naike: No conflict of interest
- BIGNON Anne: No conflict of interest
- BIGRAT Vincent: No conflict of interest
- BIHAN Kevin: Research support/Scientific studies: Pitié-Salpêtrière hospital; Trainings, Teaching: Sorbonne University
- BILLE Emmanuelle: No disclosure
- BIRCKENER Julien: No conflict of interest
- BIRONNEAU Vanessa: Trainings, Teaching: ARAIR; Invitation to national or international congresses: Alize Santé, SOS oxygène, ANTADIR
- BISBAL Magali: No conflict of interest
- BISTON Patrick: No disclosure
- BITKER Laurent: No conflict of interest
- BLANC-BRUDE Olivier: No disclosure
- BLATEAU Alain: No disclosure
- BLOCH-LAINE Emmanuel: No conflict of interest
- BLUM Laurene: No disclosure
- BOCCARA Johanna: No conflict of interest
- BOCCI Maria grazia: Trainings, Teaching: Catholic University of Rome
- BOCHUD Pierre-Yves: No disclosure
- BODEMER Christine: No disclosure
- BODENES Laetitia: No conflict of interest
- BODET-CONTENTIN Laetitia: No conflict of interest
- BOGGIAN Katia: No conflict of interest
- BOIGE Valérie: Research support/Scientific studies: Merck Serono; Consultancy, Expert: Bayer, Merck Serono, Eisai, Ipsen; Trainings, Teaching: Ipsen, Amgen; Invitation to national or international congresses: Roche, Sanofi, Merck Serono, Bayer
- BOILÈVE Alice: No conflict of interest
- BOISSEAU Chloé: No disclosure
- BOISSEL Nicolas: No disclosure
- BOISSIER Florence: No conflict of interest
- BOIVIN Alexandra: No conflict of interest
- BONACORSI Stéphane: No conflict of interest
- BONGIOVANNI Filippo: No conflict of interest
- BONNARDEL Eline: No conflict of interest
- BONNEFOY-CUDRAZ Eric: No disclosure
- BONNET Sixtine: No conflict of interest
- BONNEVIE Tristan: Research support/Scientific studies: Fisher & Paykel
- BONTEN Marc: No disclosure
- BORCOMAN Edith: No disclosure
- BORDES Julien: No conflict of interest
- BORDET Fabienne: No conflict of interest
- BORDET Jeanne: No conflict of interest
- BORGI Aïda: No conflict of interest
- BORIES Phuong-Nhi: No conflict of interest
- BORRHOMÉE Suzanne: No conflict of interest
- BORTOLOTTI Perrine: No conflict of interest
- BOSC Romain: No disclosure
- BOSIO Silvia: No conflict of interest
- BOST-BRU Cécile: No disclosure
- BOTTE Astrid: No conflict of interest
- BOUADMA Lila: Research support/Scientific studies: Thermo Fisher Scientific
- BOUATTOUR Abir: No conflict of interest
- BOUAZIZ Mounir: No conflict of interest
- BOUAZZA Naïm: No conflict of interest
- BOUBAKER Ghalia: No conflict of interest
- BOUBAKER Radhia: No conflict of interest
- BOUBECHE Samia: No disclosure
- BOUCHAALA Karama: No conflict of interest
- BOUCHIIRA Hasna: No conflict of interest
- BOUDON Marc: Invitation to national or international congresses: EUMEDICA
- BOUGDAL Dalila: No conflict of interest
- BOUGHANMI Hatem: No conflict of interest
- BOUGHARRIOU Sadok: No conflict of interest
- BOUGOUIN Wulfran: Consultancy, Expert: Withings
- BOUHARAOUA Sihem: No disclosure
- BOUHEMAD Belaid: No conflict of interest
- BOUHOURI Aziz: No disclosure
- BOUIDIR Youssef: No conflict of interest
- BOUKARI Madjid: No disclosure
- BOUKOUB Naila: No conflict of interest
- BOULAFTALI Yacine: No conflict of interest
- BOULAIN Thierry: Invitation to national or international congresses: Pfizer
- BOULANGER Eric: Consultancy, Expert: Roquette SA; Invitation to national or international congresses: SFGG and EUGMS
- BOULARAN Josiane: No disclosure
- BOUNES Vincent: No disclosure
- BOURCIER Simon: No disclosure
- BOURDIN Gaël: No conflict of interest
- BOUREL Claire: No conflict of interest
- BOURENNE Jeremy: No disclosure
- BOURGOIN Pierre: Research support/Scientific studies: ORIONPAHARMA in 2018 and MEDTRONIC in 2017
- BOURMAUD Aurelie: No conflict of interest
- BOURSIER Simon: No conflict of interest
- BOUSBIA Soulef: No conflict of interest
- BOUSSARSAR Mohamed: No disclosure
- BOUSTA Medhi: No disclosure
- BOUTIN Emmanuelle: No disclosure
- BOUTONNET Mathieu: No conflict of interest
- BOUZAT Pierre: No conflict of interest
- BOUZBIB Charlotte: Consultancy, Expert: Gilead; Invitation to national or international congresses: Gilead
- BOUZIANE Mohammed: No disclosure
- BOUZIRI Asma: No conflict of interest
- BOYER Alexandre: Trainings, Teaching: Basilea
- BOYER Déborah: No conflict of interest
- BRADAI Sabrine: No conflict of interest
- BRAHMI Habib: No conflict of interest
- BRAHMI Nozha: No disclosure
- BRASSART Benoit: No conflict of interest
- BRASSIER Anais: No disclosure
- BREBION Amélie: Invitation to national or international congresses: bioMérieux, Hologic, Janssen
- BRECHOT Nicolas: No conflict of interest
- BREDIN Swann: No conflict of interest
- BRETAGNOL Anne: No disclosure
- BREUREC Sébastien: No conflict of interest
- BRIDIER-NAHMIAS Antoine: No conflict of interest
- BRISARD Laurent: Research support/Scientific studies: BAXTER (Grant Research)
- BROCHARD Laurent: No disclosure
- BROCHON Sandie: No disclosure
- BROSSIER David: No conflict of interest
- BROWN Delphine: No conflict of interest
- BRUEL Cedric: No disclosure
- BRULE Noelle: No disclosure
- BRUNEEL Fabrice: Invitation to national or international congresses: PFIZER, GILEAD, MSD
- BRUNOT Vincent: Invitation to national or international congresses: Fresenius Kabi and Fresenius Medical Care
- BUCUR Petru: No disclosure
- BUETTI Niccolo: Research support/Scientific studies: Swiss National Science foundation research grant and bangerter rhyner foundation supporting my postdoc
- BUI Hoang-Nam: No disclosure
- BURELLI Gabrielle: No conflict of interest
- BURGEL Pierre-Régis: No disclosure
- BURGHI G: No conflict of interest
- BUSTARRET Olivier: No conflict of interest
- BUTIN-DRUOTON Anne-Lise: Invitation to national or international congresses: Astellas
- CABIE André: No disclosure
- CABRE Philippe: No conflict of interest
- CABRIO Davy: No conflict of interest
- CADOZ Cyril: No disclosure
- CALANDRA Thierry: No disclosure
- CALLOT Delphine: No conflict of interest
- CALLY Radj: No conflict of interest
- CALVAT Sylvie: No conflict of interest
- CAMPFORT Maëva: No conflict of interest
- CAMUS Christophe: No conflict of interest
- CANET Emmanuel: Consultancy, Expert: Gilead
- CANNET Floriane: No conflict of interest
- CANOUI-POITRINE Florence: Research support/Scientific studies: Abbvie
- CAPDEVILA Xavier: No conflict of interest
- CAPELLIER Gilles: Consultancy, Expert: Baxter; Invitation to national or international congresses: Asten
- CAPITO Carmen: No conflict of interest
- CAPLAN Morgan: No conflict of interest
- CAPLIN Cecile: No disclosure
- CARFANTAN Cyril: No conflict of interest
- CARIOU Alain: Consultancy, Expert: Bard
- CARLES Michel: Invitation to national or international congresses: SOS Oxygène
- CARPENTIER Dorothée: No disclosure
- CARRIÉ Cédric: No disclosure
- CARTEAUX Guillaume: Consultancy, Expert: Air Liquide Medical Systems; Trainings, Teaching: Air Liquide Medical Systems
- CARVAL Thibaut: No conflict of interest
- CASSIBBA Julie: No conflict of interest
- CASSINA Tiziano: No conflict of interest
- CASSIR Nadim: Invitation to national or international congresses: Pfizer, MSD, Eumedica
- CASTANARES Diego: No conflict of interest
- CASTELAIN Vincent: Invitation to national or international congresses: Pfizer; Astellas; Philips
- CASTILLO Jean-Marie: No disclosure
- CATANO Jennifer: No conflict of interest
- CAVAILLE Guilhem: No disclosure
- CAVAILLON Jean-Marc: No disclosure
- CECCHINI Jerome: No conflict of interest
- CERASUOLO Damiano: No conflict of interest
- CERUTI Samuele: No conflict of interest
- CHABARTIER Cyrille: Invitation to national or international congresses: FRESENIUS
- CHABERT Paul: No conflict of interest
- CHAFFAUT Cendrine: No conflict of interest
- CHAILLOU Delphine: Consultancy, Expert: Novartis; Invitation to national or international congresses: Novartis
- CHAMPION Suzanne: No disclosure
- CHAMPY Pauline: No disclosure
- CHANOINE Sébastien: Consultancy, Expert: AstraZeneca; Invitation to national or international congresses: Astellas, LFB, Novartis, MSD, Actelion
- CHAPALAIN Xavier: No conflict of interest
- CHAPEAU David: No conflict of interest
- CHAPLAIN Agathe: No disclosure
- CHARBIT Jonathan: No disclosure
- CHARDOT Christophe: No conflict of interest
- CHAREYRE Judith: No conflict of interest
- CHARPENTIER Julien: Trainings, Teaching: MSD; Invitation to national or international congresses: PFIZER; CORREVIO
- CHARRA Boubaker: No disclosure
- CHARRON Cyril: No disclosure
- CHARUE Dominique: No disclosure
- CHARUEL Jean-luc: No disclosure
- CHASE J. Geoffrey: Research support/Scientific studies: Tiro Medical, Medtronic Inc; Consultancy, Expert: Tiro Medical, Medtronic Inc; Stock shareholder: Tiro edical; Patent or product inventor: Tiro Medical, Medtronic Inc
- CHATZIANTONIOU Christos: No conflict of interest
- CHAUVELOT Louis: Invitation to national or international congresses: Laidet médical
- CHAZOT Guillaume: No conflict of interest
- CHELBI Rym: No conflict of interest
- CHELLY Jonathan: Consultancy, Expert: Hamilton Medical; Invitation to national or international congresses: Hamilton medical
- CHEMLI Wael: No conflict of interest
- CHENOUARD Alexis: No conflict of interest
- CHERKAB Rachid: No conflict of interest
- CHEVRET Sylvie: No disclosure
- CHHUN Stephanie: No conflict of interest
- CHICHE Jean-Daniel: No disclosure
- CHICOISNEAU Maxence: No conflict of interest
- CHLILEK Abdelaziz: No disclosure
- CHOCRON Richard: Consultancy, Expert: ASPEN
- CHOMMELOUX Juliette: No conflict of interest
- CHOMTON Maryline: No conflict of interest
- CHOSIDOW Olivier: No disclosure
- CHOUCHANA Laurent: No conflict of interest
- CHOUCHENE Imed: No disclosure
- CHOUKROUN Gérald: No conflict of interest
- CHTARA Kamilia: No conflict of interest
- CICCONE Camille: No conflict of interest
- CINOTTI Raphaël: No conflict of interest
- CLAIR Bernard: No disclosure
- CLAUDINON Aurore: No conflict of interest
- CLAUSSE Darless: No conflict of interest
- CLAVIER Thomas: No disclosure
- CLEMENT Nicolas: No conflict of interest
- CLEUZIOU Pierre: Invitation to national or international congresses: Eisai
- CLOUZEAU Benjamin: No conflict of interest
- COCCINI Coralie: No disclosure
- COGO Adrien: No disclosure
- COHEN Johana: No disclosure
- COHEN Laure: No conflict of interest
- COHEN Yves: No conflict of interest
- COIFFARD Benjamin: No conflict of interest
- COINTAULT Olivier: No disclosure
- COIRIER Valentin: No conflict of interest
- COITEUX Valerie: No disclosure
- COLARDELLE Philippe: No disclosure
- COLIN Gwenhael: Consultancy, Expert: Combioxin
- COLOMB Benoit: No conflict of interest
- COLOMBANI Sarah: No conflict of interest
- COLOMBANI Sylvie: No conflict of interest
- COLOT Julien: No disclosure
- COMBAUX Danièle: No conflict of interest
- COMBES Alain: Research support/Scientific studies: GETINGE; Consultancy, Expert: GETINGE, BAXTER, XENIOS; Trainings, Teaching: GETINGE, BAXTER, XENIOS; Invitation to national or international congresses: GETINGE, BAXTER, XENIOS
- COMBRET Yann: No conflict of interest
- COMMEREUC Morgane: No disclosure
- CONIA Alexandre: No disclosure
- CONSTANTIN Jean-Michel: No disclosure
- CONTAL Olivier: No disclosure
- CONTOU Damien: No conflict of interest
- CORNE Philippe: No disclosure
- CORNET Muriel: Research support/Scientific studies: Pfizer; Consultancy, Expert: Pfizer; Trainings, Teaching: Gilead Science; Invitation to national or international congresses: Gilead Sciences, Pfizer
- COSTE Joël: No disclosure
- COSTES Nicolas: No conflict of interest
- COUDROY Rémi: Research support/Scientific studies: European Respiratory Society, SRLF
- COUPEZ Elisabeth: No disclosure
- COUR Martin: No conflict of interest
- COURCELLE Romain: No conflict of interest
- COURET David: No conflict of interest
- COURTEILLE Benoit: No disclosure
- COUSEIN Etienne: Consultancy, Expert: Becton Dickinson, LFB; Invitation to national or international congresses: Novartis
- COUSIN Nicolas: No conflict of interest
- COUSSEMENT Julien: Consultancy, Expert: SANOFI; Invitation to national or international congresses: EUMEDICA
- COUTANCE Guillaume: Research support/Scientific studies: Biotest; Consultancy, Expert: Biotest; Invitation to national or international congresses: Sanofi, Biotest
- COUTEAU-CHARDON Amélie: No conflict of interest
- CRACCO Christophe: No conflict of interest
- CREMER Olaf: No disclosure
- CRETEUR Jacques: No conflict of interest
- CROISIER Jean-Louis: No conflict of interest
- CROSBY Laura: No conflict of interest
- CRULLI Benjamin: No conflict of interest
- CZOSNYKA Marek: No disclosure
- D’HUMIERES Camille: Research support/Scientific studies: Université de Paris; Trainings, Teaching: Université de Paris
- DA SILVA Daniel: Research support/Scientific studies: Fresenius Medical Care; Consultancy, Expert: Fresenius Medical Care; Invitation to national or international congresses: Xenios Novalung, Heilbronn, Germany
- DACHRAOUI Fahmi: No disclosure
- DAHOUMANE Redouane: No conflict of interest
- DAHYOT-FIZELIER Claire: No disclosure
- DAIX Thomas: No conflict of interest
- DALY Foued: No conflict of interest
- DAMONTI Lauro: No conflict of interest
- DANTAN Etienne: No conflict of interest
- DARMON Michaël: Research support/Scientific studies: MSD, Astute Medical; Consultancy, Expert: Gilead-Kite; Trainings, Teaching: MSD, Astelas, Gilead-Kite
- DARMONT Michael: No disclosure
- DAS Vincent: No disclosure
- DAUBIN Cedric: No conflict of interest
- DAUBIN Delphine: No conflict of interest
- DAUDON Michel: No disclosure
- DAUFRESNE Pierre: No conflict of interest
- DAUGER Stéphane: No conflict of interest
- DAVIET Florence: Invitation to national or international congresses: sandosz
- DE COURSON Hugues: No conflict of interest
- DE JONG Audrey: Trainings, Teaching: Baxter, Medtronic; Invitation to national or international congresses: Astellas
- DE LONLAY Pascale: Research support/Scientific studies: INSERM; Trainings, Teaching: Diplome inter-universitaire maladies héréditaires du métabolisme; Invitation to national or international congresses: Plusieurs congrès; Patent or product inventor: brevet académique
- DE MONTMOLLIN Etienne: No conflict of interest
- DE PROST Nicolas: No conflict of interest
- DE ROUX Quentin: No conflict of interest
- DE SAINT-BLANQUAT Laure: No conflict of interest
- DE VITA Nello: No conflict of interest
- DEBATY Guillaume: Consultancy, Expert: Zoll Medical
- DEBBICHE Lilya: No conflict of interest
- DEBOCK Romain: No disclosure
- DEBRAY Dominique: Consultancy, Expert: ALEXION,; Trainings, Teaching: VERTEX; Invitation to national or international congresses: ASTELLAS and SANOFI
- DEBUREAUX Pierre-Edouard: No conflict of interest
- DECAMPS Paul: No conflict of interest
- DECLERCQ Pierre-Louis: Invitation to national or international congresses: Isis Médical, Pfizer
- DECORMEILLE Guillaume: Trainings, Teaching: CHU TOULOUSE; Invitation to national or international congresses: SFAR-SRLF
- DECOUSSER Jean-Winoc: Invitation to national or international congresses: MOBIDIAG
- DEHO Anna: No conflict of interest
- DEKEYSER Thibault: No conflict of interest
- DEL BELLO Arnaud: No disclosure
- DELANNOY Pierre-Yves: No conflict of interest
- DELASTRE Olivier: No disclosure
- DELEMAZURE Julie: Trainings, Teaching: cardioSleep
- DELHAES Laurence: No disclosure
- DELIGNETTE Marie-charlotte: No conflict of interest
- DELLAMONICA Jean: Trainings, Teaching: Medtronic; Invitation to national or international congresses: MSD, General Electrics
- DELPIERRE Clément: No conflict of interest
- DELVILLE Marianne: No conflict of interest
- DEMAILLY Zoé: Research support/Scientific studies: SRLF
- DEMAREST Elsa: No disclosure
- DEMARET Pierre: No conflict of interest
- DEMISELLE Julien: No conflict of interest
- DEMONDION Pierre: No conflict of interest
- DEMOULE Alexandre: Research support/Scientific studies: Drager, Philips; Consultancy, Expert: Baxter, Respinor, LungPacer; Trainings, Teaching: Fisher & Paykel, Hamilton, Baxter; Invitation to national or international congresses: Fisher & Paykel
- DENIS Manon: No conflict of interest
- DEPEYRE Fanny: Invitation to national or international congresses: Pfizer
- DEPLANTE Yvon: No conflict of interest
- DEQUIN Pierre-François: Research support/Scientific studies: MedImmune Combioxin Ferring Pharmaceuticals A/S Asahi Kasei Pharma America Corporation
- DERAUGLAUDRE Lucie: No conflict of interest
- DERBEL Karim: No disclosure
- DERKAOUI Ali: No disclosure
- DERVIN Krystel: No conflict of interest
- DESAIVE Thomas: No conflict of interest
- DESGUERRE Isabelle: Research support/Scientific studies: Ptc inc, Avexis; Consultancy, Expert: Avexis, PTC inc, Biogene; Trainings, Teaching: roche, PTC inc, Avexis; Invitation to national or international congresses: sarepta, biogen, Avexis, Biomarin
- DESNOS Cyrielle: No conflict of interest
- DESROYS DU ROURE François: No conflict of interest
- DETOLLENAERE Charles: No conflict of interest
- DEVAQUET Jérôme: Invitation to national or international congresses: MSD France
- DEVILDER Mathieu: No conflict of interest
- DEVOS Philippe: No conflict of interest
- DEVROEY Marianne: No conflict of interest
- DEYE Nicolas: Invitation to national or international congresses: Zoll, Bard
- DHONNEUR Gilles: No disclosure
- DHRAIEF Sarra: No conflict of interest
- DI MEGLIO Lucas: Research support/Scientific studies: PhD grant from “la fondation de l’avenir pour la recherche médicale”
- DIANA Jean-Sebastien: Trainings, Teaching: PARIS DESCARTES (PARIS 5); Invitation to national or international congresses: EBMT, ESID workshop, ASCA, SFP, SHIP
- DIDIER Hommel: No conflict of interest
- DIEHL Jean-Luc: Research support/Scientific studies: Alung, General Electric HealthCare, Fresenius Medical Care; Consultancy, Expert: Fresenius Medical Care; Invitation to national or international congresses: Fresenius Medical Care, General Electric HealthCare
- DINA Julia: No disclosure
- DJEBLI Houria: No conflict of interest
- DJIBRE Michel: No conflict of interest
- DLELA Mariem: No conflict of interest
- DO VALE Julien: No conflict of interest
- DOGHRI Hamdi: No disclosure
- DOIT Catherine: No disclosure
- DOLLAT Camille: No conflict of interest
- DOLOU Julien: No conflict of interest
- DONETTI Laurence: No conflict of interest
- DOURTHE Marie-Emilie: Invitation to national or international congresses: Novartis
- DOYEN Denis: No conflict of interest
- DRES Martin: Consultancy, Expert: Lungpacer; Invitation to national or international congresses: Lungpacer
- DREYFUSS Didier: Research support/Scientific studies: Grant from French Ministry of Health
- DROUOT Xavier: No disclosure
- DU CHEYRON Damien: No conflict of interest
- DUBÉ Bruno-Pierre: Consultancy, Expert: Novartis, GSK
- DUBERT Marie: No conflict of interest
- DUBOST Baptiste: No conflict of interest
- DUBOST Jean-Louis: No conflict of interest
- DUBURCQ Thibault: No conflict of interest
- DUCHEMANN Boris: Consultancy, Expert: BMS, MSD, Roche; Invitation to national or international congresses: ROCHE, OXYVIE, PFIZER, Astra zeneca
- DUCLOUX Diane: No conflict of interest
- DUCREUX Michel: No disclosure
- DUFLOT Thomas: No conflict of interest
- DUFOSSEZ Marie-Christine: No conflict of interest
- DUFOUR Nicolas: Consultancy, Expert: Agence Nationale de Sécurité du Médicament et des produits de santé
- DUMAS Florence: No conflict of interest
- DUMAS Guillaume: No conflict of interest
- DUMONTET Erwan: No conflict of interest
- DUPEYRAT Julien: No conflict of interest
- DUPIC Laurent: Invitation to national or international congresses: ESPNIC
- DUPLAND Pierre: No conflict of interest
- DUPONT Axelle: No conflict of interest
- DUPONT Damien: Trainings, Teaching: Pfizer; Invitation to national or international congresses: MSD
- DUPONT Sébastien: No conflict of interest
- DUPONT Thibault: No conflict of interest
- DUPUIS Claire: No conflict of interest
- DUPUY Caroline: No disclosure
- DURAND Francois: No disclosure
- DUREUIL Bertrand: No disclosure
- DURRBACH Antoine: No disclosure
- ECKERT Philippe: No conflict of interest
- ECOLLAN Patrick: Trainings, Teaching: ASTRA ZENECA; LILLY
- EDDOUISSI Wafaa: No disclosure
- EGBOHOU Pilakimwe: No disclosure
- EGO Anne: No disclosure
- EGRETEAU Pierre-Yves: No conflict of interest
- EHRMANN Stephan: Research support/Scientific studies: Aerogen Ltd, Fisher & Paykel, Hamilton; Consultancy, Expert: Aerogen Ltd, La Diffusion Technique Française; Trainings, Teaching: Aerogen Ltd, Fisher & Paykel; Invitation to national or international congresses: Fisher & Paykel, Aerogen Ltd; Patent or product inventor: Brevet européen; Jet aerosol dispenser brevet EP17305015
- EL BEKI Nabil: No conflict of interest
- EL FAKHR Kaoutar: No conflict of interest
- EL HADHRI Asma: No conflict of interest
- EL HAJ Tayssir: No disclosure
- EL HARRAR Najib: No disclosure
- EL HARRAR Rachid: No disclosure
- EL IDRISSI Mouna: No disclosure
- EL KALIOUBIE Ahmed: No conflict of interest
- EL KETTANI Chafik: No disclosure
- EL KOUARI Fadwa: Invitation to national or international congresses: Celgene
- ELAIASSI Mohamed: No disclosure
- ELATROUS Souheil: Invitation to national or international congresses: Medis Tunisie
- ELHADRAMI Reda: No disclosure
- ELHAMMOUDI Driss: No disclosure
- ELHAMZAOUI Hamza: No disclosure
- ELHARRAR Najib: No disclosure
- ELHECHMI Youssef zied: No disclosure
- ELIE Caroline: No conflict of interest
- ELODIE Baron: No disclosure
- ELOUARDI Youssef: No disclosure
- EMERIAUD Guillaume: Research support/Scientific studies: Maquet Critical Care
- EMNA Abid: No conflict of interest
- ENAUD Raphaël: Research support/Scientific studies: VERTEX; Consultancy, Expert: BIOCODEX; Invitation to national or international congresses: NESTLE, NUTRICIA
- ENNOURI Emna: No conflict of interest
- ENSER Maya: No conflict of interest
- EPAILLARD Nicolas: No disclosure
- ERNON Ludovic: No conflict of interest
- ESCALARD Clément: No conflict of interest
- ESIENE Agnès: No disclosure
- ESSOURI Sandrine: No conflict of interest
- ETIENNE Manuel: Invitation to national or international congresses: Gilead (SFLS 2019); Pfizer (ECCMID 2019), MSD (JNI 2019), MSD (JNI 2018), ASTELLAS (ECCMID 2018), CORREVIO (RICAI 2017), GILEAD (JNI 2017)
- ETIENNE Pascal: No disclosure
- EVRARD Bruno: Invitation to national or international congresses: Pfizer
- FADIL Aziz: No disclosure
- FADLALLAH Jehane: No disclosure
- FAGOT-GANDET Florence: No conflict of interest
- FAGUER Stanislas: Research support/Scientific studies: Astellas; Consultancy, Expert: Vifor Pharma; Invitation to national or international congresses: Astellas
- FAIN Olivier: No disclosure
- FAITOT Francois: Invitation to national or international congresses: Astellas, Novartis, Sandoz, Bayer
- FALAHI Fahimeh: No disclosure
- FALEH Kawther: No conflict of interest
- FALQUE Loic: No conflict of interest
- FARTOUKH Muriel: Research support/Scientific studies: Biomérieux, MSD; Consultancy, Expert: Biomérieux; Advertising documents: Biomérieux; Trainings, Teaching: Biomérieux; Invitation to national or international congresses: Biomérieux
- FATHALLAH Ines: No conflict of interest
- FATNASSI Meriem: No conflict of interest
- FAUCONNIER Jeremy: No conflict of interest
- FAVORY Raphael: Research support/Scientific studies: La Jolla (CA) ATHOS 3 study
- FAVRE Mathieu: No conflict of interest
- FEDOU Anne-laure: No conflict of interest
- FELIX Arthur: No conflict of interest
- FERGE Jean-louis: No conflict of interest
- FERNANDEZ Audrey: No conflict of interest
- FERNANDEZ FERNANDEZ Elena: Research support/Scientific studies: Aerogen Ltd.
- FERRE Alexis: No conflict of interest
- FERREIRA Luis: No conflict of interest
- FERRIÈRE Nicolas: Invitation to national or international congresses: ORKYN
- FERRONI Agnès: No disclosure
- FEUILLET Fanny: No conflict of interest
- FEYDEAU Pauline: No conflict of interest
- FIANCETTE Maud: Research support/Scientific studies: AKPA
- FIESCHI Claire: No disclosure
- FIHMAN Vincent: No conflict of interest
- FILLATRE Pierre: Research support/Scientific studies: Agence de Biomédecine
- FINEBERG David: Research support/Scientific studies: I am a full time employee of Asahi Kasei Pharma America, the research sponsor
- FIORE Antonio: No conflict of interest
- FITTING Catherine: No conflict of interest
- FLATRES Aurelien: Research support/Scientific studies: SuperSonic Imagine S.A. (Aix-en-Provence, France)
- FLECHELLES Olivier: No disclosure
- FLECHER Erwan: No disclosure
- FLORENTIN Jonathan: No disclosure
- FOLLIET Laure: No conflict of interest
- FONTAINE Candice: No conflict of interest
- FOREL Jean-Marie: No disclosure
- FORFAIT Carole: No conflict of interest
- FORT Clémentine: No conflict of interest
- FOSSÉ Quentin: No conflict of interest
- FOUCHER Yohann: No conflict of interest
- FOUDHAILI Nassereddine: No conflict of interest
- FOURNIER Alicia: No conflict of interest
- FOURNIER Clement: No disclosure
- FRAJ Nesrine: No conflict of interest
- FRANCHINEAU Guillaume: No conflict of interest
- FRANCOIS Bruno: Research support/Scientific studies: Asahi Kasei Pharma America
- FRANCOZ Claire: Invitation to national or international congresses: Biotest, Chiesi, Gilead
- FRANGE Pierre: Research support/Scientific studies: French Agency for Research on AIDS and Viral Hepatitis (Inserm-ANRS), Shire, Chimerix; Consultancy, Expert: MSD France; Trainings, Teaching: MSD France, Janssen-Cilag, Gilead Sciences, Medtronic; Invitation to national or international congresses: Gilead Sciences, Janssen-Cilag, MSD France, Pfizer, Astellas
- FRASCA Denis: No conflict of interest
- FRAT Jean-Pierre: Research support/Scientific studies: french ministry of health; Consultancy, Expert: Fisher and Paykel Healthcare, SOS oxygène; Invitation to national or international congresses: Fisher and Paykel Healthcare, SOS oxygène
- FREDJ Hana: No conflict of interest
- FRÉROU Aurélien: No conflict of interest
- FRITZ Caroline: No disclosure
- FROMENTIN Mélanie: Research support/Scientific studies: MSD; Invitation to national or international congresses: MSD
- FROUIN Antoine: No conflict of interest
- FRUGIER Alexandre: No disclosure
- GABORIAU Louise: No conflict of interest
- GACI Rostane: Invitation to national or international congresses: BARD
- GACOUIN Arnaud: No disclosure
- GADDAS Mehdi: No conflict of interest
- GAILLARD Arnaud: Trainings, Teaching: Zoll Medical
- GAIMARD Sophie: No conflict of interest
- GAINNIER Marc: No conflict of interest
- GALBOIS Arnaud: No conflict of interest
- GALERNEAU Louis-Marie: Invitation to national or international congresses: Agir À Domicile
- GALICIER Lionel: Consultancy, Expert: NOVARTIS, EUSAPHARMA; Trainings, Teaching: Baxalta, PFIZER; Invitation to national or international congresses: JANSSEN, NOVARTIS, SHIRE
- GALLAH Salah: No conflict of interest
- GAMON Lucie: No conflict of interest
- GANA Inès: No conflict of interest
- GANDJBAKHCH Estelle: No disclosure
- GANSTER Frédérique: No disclosure
- GANTOIS Guillaume: No conflict of interest
- GARBAA Yesmine: No conflict of interest
- GARBEZ Nicolas: No conflict of interest
- GARNIER Fanny: No conflict of interest
- GARRET Charlotte: No disclosure
- GARRIGUES Eve: No conflict of interest
- GARROUSTE Maïté: No conflict of interest
- GASRI Bahija: No conflict of interest
- GASTINNE Thomas: No disclosure
- GASTLI Nabil: No conflict of interest
- GAUBERTI Maxime: No conflict of interest
- GAUDEL Myrtille: Trainings, Teaching: Getinge France; Invitation to national or international congresses: Drager Médical SAS
- GAUDRY Stéphane: Trainings, Teaching: ZAMBON
- GAVAUD Ariane: No conflict of interest
- GAVELLI Francesco: No conflict of interest
- GEERAERTS Thomas: No conflict of interest
- GELEE Bruno: No conflict of interest
- GENEIX Mario: No conflict of interest
- GENIEYS Pauline: No conflict of interest
- GENNISSON Jean-Luc: Consultancy, Expert: supersonic imagine
- GENUINI Mathieu: No conflict of interest
- GEORGES Hugues: No conflict of interest
- GERI Guillaume: No conflict of interest
- GERMAIN Adeline: No conflict of interest
- GESLAIN Guillaume: No conflict of interest
- GETTE Sébastien: No disclosure
- GHADHOUNE Hatem: No conflict of interest
- GHEERBRANT Hubert: No conflict of interest
- GHOMARI Nabil: No conflict of interest
- GHRENASSIA Etienne: No disclosure
- GIABICANI Mikhael: No conflict of interest
- GIACARDI Christophe: No conflict of interest
- GIAJ LEVRA Matteo: No conflict of interest
- GIBELIN Aude: No conflict of interest
- GILIBERTO Jean-Pierre: No disclosure
- GINGUAY Antonin: No conflict of interest
- GIRARD Muriel: No conflict of interest
- GIRAUDEAU Bruno: No conflict of interest
- GIRAULT Christophe: No conflict of interest
- GIRY Marion: No conflict of interest
- GISBERT-MORA Chloé: No conflict of interest
- GLAVNIK Boris: No disclosure
- GLENISSON Jean: No disclosure
- GODEAU Elise: No disclosure
- GOETZ Christophe: No disclosure
- GOLDGRAN-TOLEDANO Dany: No disclosure
- GOLDWIRT Lauriane: No conflict of interest
- GONTIER Olivier: No disclosure
- GONZALEZ Frederic: No conflict of interest
- GORBEL Rezk: No conflict of interest
- GORO Seydou: No conflict of interest
- GOUDELIN Marine: No conflict of interest
- GOULMANE Mourad: Research support/Scientific studies: Oran hospital and university center; Trainings, Teaching: Faculty of Medicine of Oran ALGERIA; Invitation to national or international congresses: french language reanimation company
- GOURSAUD Suzanne: No conflict of interest
- GOUTAY Julien: No conflict of interest
- GOYER Isabelle: No conflict of interest
- GRALL Maximilien: No disclosure
- GRALL Nathalie: Invitation to national or international congresses: Da Volterra
- GRAMMATICO-GUILLON Leslie: No conflict of interest
- GRANDMOUGIN Daniel: No conflict of interest
- GRANGÉ Steven: Consultancy, Expert: Alexion, Sanofi; Invitation to national or international congresses: Alexion, Sanofi, Octapharma
- GRAS-LEGUEN Christèle: No disclosure
- GRAVIER Francis-Edouard: No conflict of interest
- GREGNON Marion: No conflict of interest
- GREGOIRE Murielle: No conflict of interest
- GRIETEN Jef: No conflict of interest
- GRIMALDI David: Trainings, Teaching: Alexion; Invitation to national or international congresses: MSD
- GRIMAUD Marion: No conflict of interest
- GRIMBERT Philippe: No disclosure
- GRISOTTO Coline: No conflict of interest
- GRONDELAERS Jill: No conflict of interest
- GROS Antoine: No disclosure
- GRUSON Didier: No conflict of interest
- GUENANE Kamel: No conflict of interest
- GUERCI Philippe: Invitation to national or international congresses: SFAR, ESICM
- GUERIN Emmanuelle: No conflict of interest
- GUÉRIN Claude: No conflict of interest
- GUÉROT Emmanuel: No conflict of interest
- GUERVILLY Christophe: No disclosure
- GUETTECHE Choubeila: No conflict of interest
- GUEYE Papa: No conflict of interest
- GUIDET Bertrand: Research support/Scientific studies: Grifols; Invitation to national or international congresses: Grifols
- GUILLOIS Bernard: No disclosure
- GUILLON Antoine: Consultancy, Expert: Becton Dickinson
- GUILLON Benoit: No disclosure
- GUILLOT Camille: No conflict of interest
- GUINAULT Damien: Consultancy, Expert: SANOFI, ASTELLAS; Invitation to national or international congresses: NOVARTIS
- GUIOT Julien: No conflict of interest
- GUISSOUMA Jihene: No conflict of interest
- GUITTON Christophe: Invitation to national or international congresses: Fisher & Paykel, Pfizer
- GUNTZ Julien: No conflict of interest
- HABIBA Sik Ali: No conflict of interest
- HABIBI Anoosha: Research support/Scientific studies: addmedica, agios; Consultancy, Expert: Bluebird, Pfizer; Trainings, Teaching: Novartis; Invitation to national or international congresses: Addmedica
- HABRE Walide: No disclosure
- HADCHOUEL Juliette: No conflict of interest
- HADDAD Faten: No conflict of interest
- HADJ Mathilde: No conflict of interest
- HADJ HASSINE Shatila: No conflict of interest
- HAGUI Mounir: No conflict of interest
- HAJJI Ahmed: Invitation to national or international congresses: société tunisienne de pédiatrie
- HAKIMI Abdelmalek: No disclosure
- HAMDI Aicha: No conflict of interest
- HAMIDFAR Rebecca: No conflict of interest
- HAMMAMI Emna: No conflict of interest
- HAMMAMI Maha: No conflict of interest
- HAMMAMI Zouhir: No disclosure
- HAMMOUDA Zeineb: No conflict of interest
- HAMROUN Aghiles: No conflict of interest
- HAMROUNI Mouldi: No disclosure
- HAMZAOUI Olfa: Trainings, Teaching: Cheetah Compagny
- HANTSON Philippe: No conflict of interest
- HARANDOU Mustapha: No disclosure
- HARIRI Geoffroy: No conflict of interest
- HAUDEBOURG Anne-Fleur: No disclosure
- HEATON Nigel: No disclosure
- HEKIMIAN Guillaume: No conflict of interest
- HELMS Julie: Invitation to national or international congresses: MSD France, Pfizer PFE France, Diagnostica Stago
- HEMAMID Habiba: No conflict of interest
- HEMING Nicholas: No conflict of interest
- HENNEQUIN Carole: No conflict of interest
- HENRY Amandine: No conflict of interest
- HERBEZ Emilie: No conflict of interest
- HERBRECHT Jean-Etienne: Trainings, Teaching: Gilead
- HERMANN Bertrand: No conflict of interest
- HERNANDEZ PADILLA Ana catalina: No conflict of interest
- HERRMANN Sofia: Consultancy, Expert: Abbvie; Advertising documents: Humira; Invitation to national or international congresses: Abbvie, Janssen, Fresenius, Kabi, Cook
- HERTIG Alexandre: Consultancy, Expert: SANOFI GENZYME, HANSA; Trainings, Teaching: ASTELLAS; Invitation to national or international congresses: SANOFI GENZYME, ASTELLAS, SHIRE
- HILFIKER Roger: Research support/Scientific studies: Swiss Cancer Research
- HILLAIRE-BUYS Dominique: No conflict of interest
- HINAUT Ronan: No conflict of interest
- HIRT Déborah: No conflict of interest
- HO TIN NOE Benoit: Research support/Scientific studies: INSERM Fondation pour la Recherche Médicale InCa; Invitation to national or international congresses: GRC conferences
- HODJAT-PANAH Kyann: No disclosure
- HOGREL Jean-Yves: Consultancy, Expert: Biogen, Sarepta; Invitation to national or international congresses: Sanofi; Patent or product inventor: Ateliers Laumonier, Valotec
- HOLLEBECQUE Antoine: No disclosure
- HOLLEVILLE Mathilde: No conflict of interest
- HOPPE Marie-Anne: No disclosure
- HOUCKE Stephanie: No conflict of interest
- HOULI Rawnak: No conflict of interest
- HOUNAIN Taha: No disclosure
- HOURCASTAGNOU Edith: Invitation to national or international congresses: MSD, PFIZER
- HOURI Fadoua: No conflict of interest
- HOURIA Djebli: No disclosure
- HOURMANT Yannick: No conflict of interest
- HRAIECH Sami: No conflict of interest
- HUA Camille: No conflict of interest
- HUANG Florent: Invitation to national or international congresses: Bristol Meyers Squibb
- HUET Olivier: Consultancy, Expert: EDWARDS LIFE SCIENCE
- HUGUET Francoise: No disclosure
- HULIN Anne: Research support/Scientific studies: Bayer; Trainings, Teaching: Université Paris Descartes; Université Paris-Est Créteil
- HYVERNAT Hervé: No conflict of interest
- ICHAÏ Philippe: No conflict of interest
- IMEN Sioud: No conflict of interest
- IOOS Vincent: No disclosure
- ISERIN Franck: No disclosure
- ISSA Nahema: No conflict of interest
- JABER Samir: Consultancy, Expert: drager, Fisher-Paykel; Medtronic; Baxter; Xenios Fresenius; Trainings, Teaching: Drager, Fisher-Paykel; Medtronic; Baxter; Xenios Fresenius; Invitation to national or international congresses: Drager, Fisher-Paykel; Medtronic; Baxter; Xenios Fresenius
- JABI Rachid: No conflict of interest
- JACQ Gwenaëlle: No conflict of interest
- JACQUET Emmanuelle: Research support/Scientific studies: Unicancer (ESME and STORM studies); Invitation to national or international congresses: Pfizer; Patent or product inventor: Pfizer, Roche, Lilly
- JACQUIER Sophie: No conflict of interest
- JAFFRO Marion: No disclosure
- JAMI Imen: No conflict of interest
- JAMME Matthieu: Consultancy, Expert: Alexion
- JAMOUSSI Amira: No conflict of interest
- JANATI Adnane: No disclosure
- JANSSEN-LANGENSTEIN Ralf: Invitation to national or international congresses: Pfizer
- JAOUED Oussama: No conflict of interest
- JARRAYA Fatma: No conflict of interest
- JARRIGE Bruno: No conflict of interest
- JAUBERT Paul: No conflict of interest
- JAVAUD Nicolas: No conflict of interest
- JAVOUHEY Etienne: Research support/Scientific studies: CSL Behring; La jolla
- JAY Guillaume: No disclosure
- JEAN-MICHEL Vanessa: No conflict of interest
- JEMMELI Rim: No disclosure
- JENKINS Emily: No conflict of interest
- JERBI Zouheir: No disclosure
- JESTIN Matthieu: No conflict of interest
- JIVANJEE Mufaddal: No conflict of interest
- JOFFRE Jérémie: No conflict of interest
- JOLAINE Valerie: No conflict of interest
- JOLY Bérangère: No conflict of interest
- JONATHAN Florentin: No disclosure
- JORAM Nicolas: No conflict of interest
- JORET Aurelie: No disclosure
- JORIS Jean: No disclosure
- JOSEPH Adrien: No conflict of interest
- JOST Daniel: No conflict of interest
- JOUAN Youenn: No conflict of interest
- JOUFFROY Romain: Invitation to national or international congresses: SRLF 2018
- JOURDAIN Cecile: No disclosure
- JOURDAIN Merce: No disclosure
- JOURNOIS Didier: No disclosure
- JOUVEN Xavier: No disclosure
- JOUVET Philippe: Research support/Scientific studies: Air Liquide Santé; Invitation to national or international congresses: Air Liquide Santé
- JOYCE Mary: Research support/Scientific studies: Aerogen; Advertising documents: Aerogen; Patent or product inventor: Aerogen
- JOZWIAK Mathieu: No conflict of interest
- JUNG Boris: Research support/Scientific studies: APARD; Invitation to national or international congresses: Sedana
- KAIDOMAR Michel: No disclosure
- KALFON Pierre: Consultancy, Expert: GENERAL ELECTRIC HEALTHCARE; Invitation to national or international congresses: Fresenius
- KALLAL Hela: No conflict of interest
- KALLEL Aicha: No conflict of interest
- KALLEL Hatem: No conflict of interest
- KALLEL Kalthoum: No disclosure
- KALLEL Myriam: No disclosure
- KALOUCH Samira: Trainings, Teaching: faculte de médecine et pharmacie casablanca
- KAMAR Nassim: Research support/Scientific studies: Astellas; Consultancy, Expert: Abbvie, Amgen, Astellas, Chiesi, Gilead, Fresenius Medical care, Merck Sharp and Dohme, Neovii, Novartis, Roche, Sanofi, and Shire; Invitation to national or international congresses: Abbvie, Amgen, Astellas, Chiesi, Gilead, Fresenius Medical care, Merck Sharp and Dohme, Neovii, Novartis, Roche, Sanofi, and Shire
- KAMEL Toufik: Research support/Scientific studies: Benefit-to-risk balance of bronchoalveolar lavage in the critically ill. A prospective, multicenter cohort study
- KAMINSKI Dominique: No conflict of interest
- KAMMOUN Yassmine: No conflict of interest
- KARRAY Narjess: No conflict of interest
- KAWAGUCHI Atsushi: Research support/Scientific studies: Fonds de la recherche en sante du Quebec
- KAYANOKI Toshihiko: Research support/Scientific studies: Asahi Kasei Pharma America Corporation
- KELLENS Isabelle: No conflict of interest
- KERNEIS Mathieu: Research support/Scientific studies: Institut Servier, FFC; Consultancy, Expert: Bayer, Sanofi, Servier; Invitation to national or international congresses: Sanofi
- KERNEIS Solen: Research support/Scientific studies: bioMérieux; Consultancy, Expert: Accelerate Diagnostics, bioMérieux
- KHALED Ameni: No conflict of interest
- KHALEQ Khalid: No conflict of interest
- KHALLOUKI Mohamed: No disclosure
- KHANNA Nina: No conflict of interest
- KHARRAT Sana: No conflict of interest
- KHATOUF Mohammed: No disclosure
- KHEDHER Sana: No conflict of interest
- KHZOURI Takoua: No disclosure
- KIPNIS Eric: No disclosure
- KLAII Salma: No conflict of interest
- KLEIN Thomas: No conflict of interest
- KLOUCHE Kada: Invitation to national or international congresses: PFIZER
- KNOPP Jennifer l.: No conflict of interest
- KOSSOROTOFF Manoëlle: No conflict of interest
- KOUATCHET Achille: No disclosure
- KOURAICHI Nedia: Trainings, Teaching: Faculty of medecin Tunis
- KOUTSOUKOU Antonia: No conflict of interest
- KRID Saoussen: No conflict of interest
- KRINGS Adrien: No conflict of interest
- KSOURI Meriem: No conflict of interest
- KUBIS Nathalie: Invitation to national or international congresses: LFB; Patent or product inventor: brevet (22nd March 2018; FR 18 52473), not yet licensed
- L’HER Erwan: Research support/Scientific studies: Oxynov Inc, Québec, QC, CA, General Electrics Healthcare; Consultancy, Expert: Oxynov Inc, Québec, QC, CA, Smiths Medical, General Electrics Healthcare; Invitation to national or international congresses: General Electrics Healthcare; Stock shareholder: Oxynov Inc, Québec, QC, CA; Patent or product inventor: Université de Bretagne Occidentale, Université Laval-Qc-CA
- LABBE Vincent: No disclosure
- LABRO Laura: No disclosure
- LACAILLE Florence: No conflict of interest
- LACAMPAGNE Alain: No disclosure
- LACAN Claire: No conflict of interest
- LACHERADE Jean-Claude: No conflict of interest
- LADJEMI Maha-Zohra: No conflict of interest
- LAFON Charles: No conflict of interest
- LAFON Marie-Edith: No disclosure
- LAFON Thomas: No conflict of interest
- LAGACHE Laurie: Invitation to national or international congresses: ASTEN
- LAHMAR Manel: No disclosure
- LAHMER Basma: No conflict of interest
- LAHOUCINE Barrou: No disclosure
- LAINE Laurent: No conflict of interest
- LAISSI Mohamed: No conflict of interest
- LAKHDHAR Dhouha: No disclosure
- LALLOZ Amandine: Invitation to national or international congresses: Stryker
- LAMBERMONT Bernard: Research support/Scientific studies: Maquet, Cytosorbents; Trainings, Teaching: Drager; Invitation to national or international congresses: Orion, Acertys, Drager
- LAMBERT Jerome: No disclosure
- LAMBIOTTE Fabien: No conflict of interest
- LAMBOTTE Olivier: Research support/Scientific studies: Gilead; Consultancy, Expert: BMS, Astra Zeneca, MSD, Incyte, Janssen; Invitation to national or international congresses: BMS
- LAMER Antoine: No conflict of interest
- LAMHAUT Lionel: Research support/Scientific studies: Getinge; Trainings, Teaching: Stryker,Zoll, Getinge; Invitation to national or international congresses: Zoll, Astra Zeneca, Getinge; Patent or product inventor: Neckpure, SAUV Life
- LAMIA Bouchra: Research support/Scientific studies: Bayer, MSD, Vivisol, Asten, Philips; Consultancy, Expert: Bayer, MSD, Chiesi, Novartis, Lowenstein, Philips,Vivisol/Advertising documents: Philips; Trainings, Teaching: Novartis, GSK, Astra Zeneca, Boeringher; Invitation to national or international congresses: Chiesi, Astra Zeneca, SOS oxygene, Novartis, Boeringher
- LAMOTH Frédéric: Consultancy, Expert: Gilead, MSD, Basilea; Invitation to national or international congresses: MSD, Gilead
- LANCEL Steve: No conflict of interest
- LANCELOT Aymeric: No disclosure
- LANGERON Olivier: No conflict of interest
- LAPIDUS Nathanael: No disclosure
- LARAMAS Mathieu: Consultancy, Expert: Astra-Zeneca; Sanofi; Trainings, Teaching: NOVASRTIS; BAYER; AMGEN; JANSSEN; Invitation to national or international congresses: SANOFI; PFIZER; ASTELLAS
- LARCHER Romaric: Trainings, Teaching: MSD, PFIZER; Invitation to national or international congresses: MSD, PFIZER
- LARDE Julie: No disclosure
- LASCARROU Jean-Baptiste: Consultancy, Expert: Asahi Kasei
- LASOCKI Sigsimond: No disclosure
- LASSALLE Thibault: No conflict of interest
- LATERRE Pierre-François: Consultancy, Expert: Ferring, Adrenomed, Inotrem
- LAUNAY Elise: No disclosure
- LAUNEY Yoann: No conflict of interest
- LAUNOIS Amélie: No conflict of interest
- LAURENT Alexandra: No conflict of interest
- LAURENT Raynaud: No disclosure
- LAURENT Virginie: No conflict of interest
- LAURIOUX Flavie: No disclosure
- LAUTRETTE Alexandre: Invitation to national or international congresses: Hemotec
- LAUZIER Benjamin: No conflict of interest
- LAVAYSSIERE Laurence: No disclosure
- LAVIGNE Flavie: No conflict of interest
- LAVILLEGRAND Jean-Rémi: No conflict of interest
- LAZARESCU Alina: No disclosure
- LAZRAQ Mohamed: No conflict of interest
- LE BARS Didier: Consultancy, Expert: NucAdvisor
- LE BERRE Nicolas: No disclosure
- LE BIHAN Clément: No conflict of interest
- LE BORGNE Pierrick: No disclosure
- LE BOUAR Gurvan: No conflict of interest
- LE BOURGEOIS Amandine: No disclosure
- LE BOURGEOIS Fleur: Invitation to national or international congresses: Fresenius Kabi France
- LE CORRE Marine: No conflict of interest
- LE GOIC Maeva: No conflict of interest
- LE GOUGE Amélie: No conflict of interest
- LE GOUILL Steven: No disclosure
- LE GUENNEC Loïc: No conflict of interest
- LE GUYADER Maxence: No disclosure
- LE NEINDRE Aymeric: Trainings, Teaching: Health Impact; Invitation to national or international congresses: SIFR, SRLF
- LE NIGER Catherine: No conflict of interest
- LE REUN Claire: No conflict of interest
- LE TACON Serge: No disclosure
- LE TERRIER Christophe: No conflict of interest
- LE TULZO Yves: No conflict of interest
- LE-LILLO LOUËT Agnès: No disclosure
- LEBERT Christine: No disclosure
- LEBIHAN Clement: No disclosure
- LEBLANC Sarah: Research support/Scientific studies: Cook; Consultancy, Expert: Norgine; Trainings, Teaching: Fujifilm, Boston Scientific
- LEBRETON Guillaume: No disclosure
- LEBRUN-VIGNES Benedicte: Research support/Scientific studies: NOVARTIS; Consultancy, Expert: ANSM
- LEBUFFE Gilles: No disclosure
- LECLERC Maxime: No conflict of interest
- LÉCLUSE Aldéric: Research support/Scientific studies: PGRX AVC study; Consultancy, Expert: BMS-Pfizer, Boerhinger Ingelheim, Bayer; Invitation to national or international congresses: BMS-Pfizer, Boerhinger Ingelheim
- LEDOUX Didier: No disclosure
- LEFEBVRE Francois: No conflict of interest
- LEFEVRE Montaine: No conflict of interest
- LEFRANT Jean-Yves: Invitation to national or international congresses: PFIZER
- LEFRERE Francois: Consultancy, Expert: NOVARTIS; Trainings, Teaching: NOVARTIS; Invitation to national or international congresses: NOVARTIS
- LEGARCON Vincent: No conflict of interest
- LEGER Julie: No conflict of interest
- LEGER Maxime: No conflict of interest
- LEGER Pierre-Louis: No conflict of interest
- LEGOUIS David: No conflict of interest
- LEGOUY Camille: No conflict of interest
- LEGRAND Matthieu: No disclosure
- LEGRAS Annick: No conflict of interest
- LEGRIEL Stéphane: Invitation to national or international congresses: London-Innsbruck Colloquium on Status Epilepticus and Acute Seizures
- LEJEUNE Manon: No disclosure
- LEMAITRE Caroline: No conflict of interest
- LEMAITRE Elise: No conflict of interest
- LEMAITRE Florian: Research support/Scientific studies: Astellas, MSD, Janssen, Sandoz; Consultancy, Expert: Janssen; Invitation to national or international congresses: Gilead
- LEMARIE Jeremy: No disclosure
- LEMERLE Marie: No conflict of interest
- LEMIALE Virginie: Trainings, Teaching: Gilead, Astellas, Alexion, Pfizer, MSD; Invitation to national or international congresses: Pfizer, Biomerieux
- LEMTIRI Justine: No conflict of interest
- LENGLINE Etienne: Invitation to national or international congresses: Gilead EHA 2019
- LENZOTTI Anne-marine: No disclosure
- LEON Lisa: No conflict of interest
- LEON Rusel: No disclosure
- LEPAGE-FARRELL Alex: No conflict of interest
- LEPELTIER Hélène: No conflict of interest
- LEPETIT Arnaud: No disclosure
- LEPEULE Raphael: Invitation to national or international congresses: MSD, Pfizer, Eumedica
- LEPRETRE Anne-Claire: No conflict of interest
- LERMUZEAUX Mathilde: No conflict of interest
- LEROY Christophe: Invitation to national or international congresses: PFIZER
- LEROY Dorian: No conflict of interest
- LEROY Olivier: No disclosure
- LESAGE Anne: No disclosure
- LESAGE Fabrice: No conflict of interest
- LESENNE Anouk: No conflict of interest
- LESOUHAITIER Mathieu: No conflict of interest
- LETAVERNIER Emmanuel: No disclosure
- LETOCART Philippe: No disclosure
- LEVESQUE Eric: No disclosure
- LEVRAT Albrice: No conflict of interest
- LEVY Michaël: No conflict of interest
- LÉVY Bruno: Research support/Scientific studies: Gettinge, Amomed; Consultancy, Expert: Orion, Amomed, novartis; Invitation to national or international congresses: Pfizer
- LÉVY Raphaël: No conflict of interest
- LHEUREUX Florent: No conflict of interest
- LHOMMET Claire: No disclosure
- LIENARD Hélène: No conflict of interest
- LIET Jean-Michel: No conflict of interest
- LIFERMANN François: No conflict of interest
- LIM Pascal: No disclosure
- LIPMAN Jeffrey: No disclosure
- LIU Vincent: No conflict of interest
- LIU Yihua: No disclosure
- LLITJOS Jean-François: No conflict of interest
- LOGRE Elsa: No conflict of interest
- LORTON Fleur: Research support/Scientific studies: Groupe de pathologie infectieuse pédiatrique; Groupe Francophone de Réanimation et Urgences Pédiatriques; Société Française de Pédiatrie-Pampers
- LOSSER Marie-Reine: Invitation to national or international congresses: Pfizer Esicm 2019
- LOUATI Assaad: No conflict of interest
- LOUIS Bruno: Research support/Scientific studies: Fondation pour la Recherche Médicale; Fédération ANTADIR; Agence Nationale de la Recherche
- LOUIS Guillaume: No conflict of interest
- LOUNDOU Anderson: No disclosure
- LSTIBUREK Laura: No conflict of interest
- LU Chen: No disclosure
- LUC Amandine: No conflict of interest
- LUCET Jean-Christophe: No conflict of interest
- LUFT Antoine: No conflict of interest
- LUIS David: No disclosure
- LUPERTO Marta: No conflict of interest
- LUYT Charles-Edouard: Research support/Scientific studies: Bayer Healthcare, Faron, AstraZenecca; Consultancy, Expert: Bayer Healthcare, Faron, ThermoFischer Brahms, Carmat; Trainings, Teaching: Merck Sharp & Dohme, Biomérieux
- MAAMAR Adel: No conflict of interest
- MAAMOURI Hela: No conflict of interest
- MAAREK Alizee: Invitation to national or international congresses: SANDOZ
- MAAROUFI Sara: No disclosure
- MAATOUG Samir: No conflict of interest
- MAATOUK Syrine: No conflict of interest
- MAATOUK Yed: No disclosure
- MAAYOUFI Houda: No conflict of interest
- MABROUKI Asma: No conflict of interest
- MACLOUGHLIN Ronan: Research support/Scientific studies: Aerogen Ltd; Patent or product inventor: Aerogen Ltd
- MADANI Naoufel: No conflict of interest
- MAGHRAOUI Hamida: No conflict of interest
- MAHAMANE RABIOU Habibou Hassane: No conflict of interest
- MAHAUX Pedro: No conflict of interest
- MAILLOUX Arnaud: No disclosure
- MAISONNEUVE Emilien: No conflict of interest
- MAIZEL Julien: No conflict of interest
- MAJED Kamel: Trainings, Teaching: ERC, faculté de médecine de tunis
- MAKOUDI Sarah: No conflict of interest
- MALACRINO Dominique: No conflict of interest
- MALKA David: No disclosure
- MALLARD Jeremy: No conflict of interest
- MALLET Vincent: No disclosure
- MAMZER Marie france: No disclosure
- MANCEAU Sandra: No conflict of interest
- MANEN Séverine: No disclosure
- MANSUY Jean-Michel: No disclosure
- MARC Julien: No conflict of interest
- MARCHAL Jean-Claude: No disclosure
- MARCHETTI Oscar: No disclosure
- MARCOTTE Guillaume: Consultancy, Expert: LFB Biomédicament; Trainings, Teaching: LFB Biomédicament
- MARHFOUR Abdeljabbar: No conflict of interest
- MARI Arnaud: No conflict of interest
- MARIE Damien: No conflict of interest
- MARIJON Eloi: No disclosure
- MARIOTTE Eric: Consultancy, Expert: Sanofi-aventis
- MARJANOVIC Nicolas: No disclosure
- MARJANOVIC Zora: No disclosure
- MARONI Arielle: No conflict of interest
- MAROT Benoit: No conflict of interest
- MARQUE Sophie: No conflict of interest
- MARTI Pierre-Emmanuel: No conflict of interest
- MARTIN Clémence: Consultancy, Expert: Zambon; Trainings, Teaching: Zambon, Chiesi; Invitation to national or international congresses: Chiesi
- MARTIN Lucile: No conflict of interest
- MARTIN Maelle: No conflict of interest
- MARTIN DELGADO Maria Cruz: No conflict of interest
- MARTIN LOECHES Ignacio: Consultancy, Expert: Msd, gilead
- MARTINO Frédéric: No conflict of interest
- MARTIS Nihal: Trainings, Teaching: Octapharma; Invitation to national or international congresses: Merck, Octapharma, Sanofi, AddMedica, Novartis, LeoPharma; Stock shareholder: Merck
- MARZOUK Mehdi: No conflict of interest
- MASI Paul: No conflict of interest
- MASSION Paul: No disclosure
- MASSON Gabriel: Research support/Scientific studies: SRLF Bourse Mobilité
- MASSON Mathilde: No conflict of interest
- MASSRI Alexandre: No disclosure
- MATECKI Stefan: No disclosure
- MATEUR Houda: No disclosure
- MATHIEN Cyrille: No conflict of interest
- MATHIEU Daniel: No conflict of interest
- MATUSIK Elodie: No conflict of interest
- MAUCHIEN Benedicte: No conflict of interest
- MAURY Eric: Research support/Scientific studies: Doran International, Drager; Trainings, Teaching: Vygon
- MAXIME Virginie: No conflict of interest
- MAYAUX Julien: Invitation to national or international congresses: Gilead
- MAYENCE Claire: No disclosure
- MAYEUR Jeanne: No disclosure
- MAZIGHI Mikael: No disclosure
- MBATCHI Litaty: No conflict of interest
- MCLEAN Anthony: No disclosure
- MEAUDRE Eric: Invitation to national or international congresses: LFB
- MEBAZAA M’hamed Sami: No conflict of interest
- MECHRI Karim: No conflict of interest
- MEDDEB Khaoula: No conflict of interest
- MEDIOUNI Karim: No conflict of interest
- MEDRANO Chloé: No disclosure
- MEDRINAL Clement: Invitation to national or international congresses: ASTEN SANTE
- MEERT Anne-Pascale: No conflict of interest
- MEFFRE Sarah: No conflict of interest
- MEGARBANE Bruno: No conflict of interest
- MEHDAOUI Hossein: No disclosure
- MEHDI Asma: No disclosure
- MEHDI Somai: No disclosure
- MEHREZ Ihssan: Research support/Scientific studies: CHU HASSAN II FES
- MEHTA Geeta: No conflict of interest
- MEISTELMAN Claude: No disclosure
- MEKONTSO DESSAP Armand: Research support/Scientific studies: Fischer and Paykel, Philips, Ferring, GSK; Consultancy, Expert: Baxter, Air Liquide, Amomed; Trainings, Teaching: Getingue, AddMedica
- MELKI Isabelle: No conflict of interest
- MELLATI Nouchan: No conflict of interest
- MELLEBEEK Evi: No conflict of interest
- MEMAIN Nathalie: No conflict of interest
- MENG Paris: No disclosure
- MENGELLE Catherine: No disclosure
- MENIF Khaled: No conflict of interest
- MENTEC Hervé: Invitation to national or international congresses: ISIS Normandie, MSD France; Stock shareholder: Tanderev; Patent or product inventor: Tanderev
- MERCAT Alain: Research support/Scientific studies: Fisher-Paykel, General Electric; Consultancy, Expert: Faron Pharmaceuticals, Air Liquide Medical System; Trainings, Teaching: Medtronic, Fisher-Paykel, Dräger Medical; Patent or product inventor: Covidien
- MERCERON Sybille: No conflict of interest
- MERCIER Emmanuelle: No conflict of interest
- MERCIER Romain: No disclosure
- MERCIER Severine: No conflict of interest
- MERCIER-DES-ROCHETTES Emmanuelle: No disclosure
- MERHABENE Takoua: No conflict of interest
- MERLE Jean-claude: No disclosure
- MESOTTEN Dieter: No conflict of interest
- MESSAADI Amenallah: No conflict of interest
- MESSIKA Jonathan: Invitation to national or international congresses: CSLBehring; Fisher&Paykel
- METAXA Victoria: No disclosure
- METOGO MBENGONO Junette arlette: No conflict of interest
- MEUNIER Anne: No conflict of interest
- MEURICE Jean-claude: No disclosure
- MEYBECK Agnes: Consultancy, Expert: Janssen, Gilead; Invitation to national or international congresses: Janssen, Viiv, Gilead
- MEYER Pascal: No disclosure
- MEZIANI Ferhat: Research support/Scientific studies: STAGO
- MEZIDI Mehdi: Research support/Scientific studies: GE Healthcare
- MGHIRBI Abdelwahab: No conflict of interest
- MHADHBI Hadil: No conflict of interest
- MIAILHE Arnaud-Félix: No conflict of interest
- MICHAELA Roy: No conflict of interest
- MICHARD Baptiste: No disclosure
- MICHEL Philippe: No conflict of interest
- MICHOT Jean-Baptiste: No conflict of interest
- MICHOT Jean-Marie: No disclosure
- MIGUERES Helene: No conflict of interest
- MILOUDI Youssef: No disclosure
- MIMOZ Olivier: Research support/Scientific studies: BD, 3 M; Consultancy, Expert: BD, 3 M, Sanofi; Invitation to national or international congresses: 3 M, BD, Sanofi
- MINOTTI Bruno: No conflict of interest
- MIRA Jean-Paul: Consultancy, Expert: ASAHI KASEI; Trainings, Teaching: MSD; Invitation to national or international congresses: ESTOR
- MIROUSE Adrien: No conflict of interest
- MISSET Benoit: Research support/Scientific studies: Bactiguard; Invitation to national or international congresses: Astellas
- MNIF Basma: No conflict of interest
- MOAL Valérie: No conflict of interest
- MOHAMADOU Inna: No conflict of interest
- MOHAMED Fekih hassen: No conflict of interest
- MOHAMED Kheder: No disclosure
- MOHAMED AMINE Amine: No disclosure
- MOHTY Mohamad: No disclosure
- MOINE Pierre: No disclosure
- MOJOLI Francesco: No disclosure
- MOKART Djamel: No conflict of interest
- MOKLINE Amel: No conflict of interest
- MOLINARI Nicolas: No disclosure
- MOLLIERE Chloe: No conflict of interest
- MONCHI Merad: No disclosure
- MONGARDON Nicolas: No conflict of interest
- MONGODI Silvia: No conflict of interest
- MONNET Xavier: Consultancy, Expert: Pulsion Médical Systems; Advertising documents: Cheetah medical; Trainings, Teaching: Pulsion Medical Systems
- MONTERO Santiago: Trainings, Teaching: ESC Clinical Training Grant in 2016
- MONTINI Florent: No conflict of interest
- MONTRAVERS Philippe: Research support/Scientific studies: Pfizer, MSD; Consultancy, Expert: Pfizer, MSD, Menarini, Tetraphase, Parexel, Bayer
- MORANDEAU Valentin: No conflict of interest
- MORAWIEC Elise: No conflict of interest
- MOREAU Anne-Sophie: No conflict of interest
- MOREL Guillaume: No conflict of interest
- MOREL Jerome: No conflict of interest
- MOREL Johanna: No conflict of interest
- MORIMONT Philippe: No conflict of interest
- MORO-SIBILOT Denis: No disclosure
- MORTAMET Guillaume: No conflict of interest
- MOSBAH Nabil: No conflict of interest
- MOSCHIETTO Sebastien: No conflict of interest
- MOUCADEL Virginie: Research support/Scientific studies: bioMérieux
- MOULAIRE RIGOLLET Valérie: No disclosure
- MOULIADE Charlotte: No conflict of interest
- MOULIN Florence: No disclosure
- MOUNIR Yousfi: No conflict of interest
- MOURABIT Karima: No disclosure
- MOURVILLIER Bruno: Trainings, Teaching: MSD; Invitation to national or international congresses: Pfizer, MSD
- MOUSSA Mouhammed: No disclosure
- MOUSSAFEUR Amina: No disclosure
- MOUSSAID Fahd: No conflict of interest
- MOUZOU Tabana: Invitation to national or international congresses: labo ACUPAN
- MULLER Gregoire: Trainings, Teaching: Astra-Zeneca; Invitation to national or international congresses: Pfizer
- MULLER Laurent: No disclosure
- MULLER Michel: No conflict of interest
- MUNTING Aline: No conflict of interest
- MURGIER Martin: No conflict of interest
- MURPHY Barry: Research support/Scientific studies: Aerogen; Advertising documents: Aerogen; Patent or product inventor: Aerogen
- MUSIARI Michele: No conflict of interest
- N’GUYEN Quang-thang: No conflict of interest
- N’GUYEN Tran: No disclosure
- NABIL Mosbah: No disclosure
- NACCACHE Lionel: No disclosure
- NAIMI Skander: No conflict of interest
- NAKAA Sabrine: No disclosure
- NALLET-AMATE Megan: No conflict of interest
- NATALIS Eloïse: No disclosure
- NAUDIN Jérôme: Invitation to national or international congresses: Novartis
- NAY Mai-Anh: No conflict of interest
- NEMLAGHI Safaa: No conflict of interest
- NEOFYTOS Dionysios: Research support/Scientific studies: MSD; Consultancy, Expert: MSD, Gilead, Pfizer; Invitation to national or international congresses: Gilead, Pfizer
- NESSELER Nicolas: No conflict of interest
- NEVIERE Remi: No disclosure
- NGUYEN Alexandre: No disclosure
- NGUYEN KHOA Thao: No conflict of interest
- NICOLAU-TRAVERS Marie-Laure: No disclosure
- NIÉRAT Marie cécile: No conflict of interest
- NIESZKOWSKA Ania: No disclosure
- NIGEON Olivier: No conflict of interest
- NITEL Gautier: No conflict of interest
- NODEA Elena Madalina: No conflict of interest
- NOEL Marine: No conflict of interest
- NOGIER Marie-béatrice: No disclosure
- NOORAH Zaid: No disclosure
- NOUIRA Wiem: No conflict of interest
- NOUMEIR Rita: Stock shareholder: Softmedical
- NOURY Norbert: No conflict of interest
- NOVY Emmanuel: Research support/Scientific studies: MSD; Invitation to national or international congresses: PFIZER, ASTELLAS
- NSEIR Saad: Consultancy, Expert: MSD; Trainings, Teaching: MSD, Pfizer, Gilead, Biomérieux, Bio-Rad
- NSIRI Afak: No disclosure
- NSSAIR Karim: No conflict of interest
- NYUNGA Martine: No conflict of interest
- O’GRADY John: No disclosure
- O’SULLIVAN Andrew: Research support/Scientific studies: Aeorgen; Advertising documents: Aerogen; Patent or product inventor: Aerogen
- OBERIC Lucie: Consultancy, Expert: Roche, Takeda, Janssen; Trainings, Teaching: Janssen; Invitation to national or international congresses: Roche, Janssen, Servier
- OLIVER Leopold: No disclosure
- OLIVIER Pierre-Yves: Consultancy, Expert: Air Liquide Medical System
- OLLIVIER Veronique: No conflict of interest
- ONIMUS Thierry: No conflict of interest
- OPPENHEIMER Anne: Invitation to national or international congresses: Gedeon Richter
- ORKISZ Maciej: No conflict of interest
- ORLIAGUET Gilles: Research support/Scientific studies: UNIVERSITÉ DE LILLE, EUROPEAN SOCIETY OF ANAESTHESIA, FONDATION ROTHSCHILD; Invitation to national or international congresses: LABORATOIRE AGUETTANT
- ORLOWSKI Sophie: No conflict of interest
- ORVAIN Corentin: Consultancy, Expert: Novartis, Incyte; Invitation to national or international congresses: Pfizer, JAZZ Pharmaceuticals
- OUALHA Mehdi: Invitation to national or international congresses: SRLF
- OUANES Islem: No disclosure
- OUCHANE Asmaa: No disclosure
- OUERFELLI Rim: No conflict of interest
- OUESLATI Meriem: No conflict of interest
- OWONO ETOUNDI Paul: No disclosure
- OZIEL Johanna: No conflict of interest
- OZIER Yves: Consultancy, Expert: LFB
- PAESMANS Marianne: No conflict of interest
- PAGANI Jean-Luc: No conflict of interest
- PAGET Christophe: No conflict of interest
- PAGLIARANI Pablo: Invitation to national or international congresses: Vegenat, Pfizer, Fresenius
- PAINVIN Benoît: No conflict of interest
- PAJOT Olivier: No conflict of interest
- PANIS Elke: No conflict of interest
- PAPAZIAN Laurent: No disclosure
- PAPE Elise: No conflict of interest
- PARLATO Mariana: No conflict of interest
- PARMENTIER-DECRUCQ Erika: No conflict of interest
- PARZIBUT Gilles: No conflict of interest
- PARZY Gabriel: No conflict of interest
- PASQUIER Pierre: No conflict of interest
- PATEAU Victoire: Research support/Scientific studies: OxyNov; Patent or product inventor: OxyNov
- PATRIER Juliette: No conflict of interest
- PAUGAM Catherine: No disclosure
- PAUL Marine: No conflict of interest
- PAUL-BELLON Rachel: No disclosure
- PAULO Nicolas: No conflict of interest
- PAVOT Arthur: Invitation to national or international congresses: Fresenius Medical Care France
- PEHLIVAN Jonathan: No conflict of interest
- PEIGNE Vincent: Invitation to national or international congresses: Air liquide
- PÉJU Edwige: No conflict of interest
- PENE Frédéric: Consultancy, Expert: Alexion
- PÉPIN-LEHALLEUR Adrien: Invitation to national or international congresses: Chiesi
- PERE Morgane: No conflict of interest
- PEREIRA Bruno: No disclosure
- PEREZ Didier: No disclosure
- PEREZ Pierre: No disclosure
- PEREZ Yonatan: No conflict of interest
- PERIER François: No disclosure
- PERIN Nicolas: No conflict of interest
- PERIOT-JARRY Mathilde: No conflict of interest
- PERNER Anders: No disclosure
- PERNY Jessica: No disclosure
- PEROZZIELLO Anne: No conflict of interest
- PERREIN Adeline: No disclosure
- PERRIAT Sophie: No disclosure
- PERRIN Clémence: No conflict of interest
- PERRIN Gilles: No disclosure
- PERSICHINI Romain: No conflict of interest
- PESCHAUD Frédérique: No conflict of interest
- PESKINE Anne: No conflict of interest
- PETERS-SENGERS Hessel: Trainings, Teaching: Amsterdam UMC-masterclass experimental nephropathology; Invitation to national or international congresses: 15th Congress of the International Society for Organ Donation and Procurement (ISODP)
- PETIT Matthieu: No conflict of interest
- PETITDEMANGE Lucie: No conflict of interest
- PEZZATO Stefano: No conflict of interest
- PHELOUZAT Pierre: No conflict of interest
- PHILIPPART Francois: No conflict of interest
- PHILIPPE Goutorbe: No disclosure
- PHILIPPE Pascal: No conflict of interest
- PIAGNERELLI Michael: No conflict of interest
- PICARD Muriel: No conflict of interest
- PICARD Yoann: No disclosure
- PICHEREAU Claire: No conflict of interest
- PICHON Nicolas: No conflict of interest
- PICKKERS Peter: No disclosure
- PIN Isabelle: Advertising documents: GSK, AZ, NOVARTIS; Trainings, Teaching: GSK, AZ, NOVARTIS; Invitation to national or international congresses: AZ, NOVARTIS
- PINETON DE CHAMBRUN Guillaume: Consultancy, Expert: Abbvie, Takeda, Pfizer, Janssen; Trainings, Teaching: Abbvie, MSD, Tillots Pharma, Takeda, Janssen, Pfizer, Hospira; Invitation to national or international congresses: Abbvie, Amgen, Takeda, Pfizer, MSD
- PINETON DE CHAMBRUN Marc: Invitation to national or international congresses: LFB
- PIQUILLOUD Lise: No conflict of interest
- PIRANI Tasneem: No conflict of interest
- PIRIOU Vincent: No disclosure
- PIROTTE Marc: No conflict of interest
- PITON Gaël: Trainings, Teaching: FRESENIUS France
- PLACIER Sandrine: No conflict of interest
- PLANTEFEVE Gaëtan: Consultancy, Expert: Panacea Conseil; Trainings, Teaching: Panacea Conseil
- PLATON Laura: Invitation to national or international congresses: EBMT, SFGMTC
- PODREZ Kevin: No disclosure
- POIROUX Laurent: Trainings, Teaching: Fisher & Paykel, Doran
- POISSY Julien: Research support/Scientific studies: SRLF, CHRU Lille; Invitation to national or international congresses: Pfizer, MSD, Eumédica
- PONCELET Géraldine: No conflict of interest
- PONS Bertrand: No conflict of interest
- PONSIN Pauline: No disclosure
- PONTIS Emmanuel: No conflict of interest
- POPOFF Benjamin: No conflict of interest
- PORTEAUD Jordan: No disclosure
- POTIER Pierre: No disclosure
- POTTECHER Julien: Research support/Scientific studies: Ambu; Invitation to national or international congresses: Amomed
- POULAIN Daniel: No conflict of interest
- POULARD Thomas: No conflict of interest
- POUPET Helene: No conflict of interest
- POYART Claire: No disclosure
- PRADAT-DIEHL Pascale: No conflict of interest
- PRAT Gwenael: No conflict of interest
- PREAU Sebastien: No conflict of interest
- PREDA Gabriel: No conflict of interest
- PREISER Jean-Charles: No conflict of interest
- PRESTIFILIPPO Alessia: No disclosure
- PREVEDELLO Danielle: No conflict of interest
- PRÉVEL Renaud: Invitation to national or international congresses: Pfizer (ECCMID Madrid 2018)
- PRIEUR Guillaume: Trainings, Teaching: Health Impact
- PRIGENT Amélie: No disclosure
- PRIM Benjamin: No disclosure
- PROTIN Caroline: No disclosure
- PRUD’HOMME Eloi: No conflict of interest
- PUISSET Florent: No disclosure
- PUYMIRAT Etienne: No disclosure
- QUENOT Jean-Pierre: No conflict of interest
- QUINTARD Hervé: No disclosure
- RABEONY Tioka: No disclosure
- RABODONIRINA Meja: No disclosure
- RACHID Alharrar: No disclosure
- RADFORD Amanda: Research support/Scientific studies: I am a full time employee at Asahi Kasei Pharma America, the research sponsor
- RAFFOUX Emmanuel: No disclosure
- RAFI Sonia: No disclosure
- RAHMANI Imen: No conflict of interest
- RAIMBAULT Mélina: No conflict of interest
- RAJA Amine: Research support/Scientific studies: A.JANNATI; Consultancy, Expert: A.BOUHOURI; Advertising documents: R.HARRAR; Trainings, Teaching: R.HARRAR; Invitation to national or international congresses: R.HARRAR; Stock shareholder: R.HARRAR; Patent or product inventor: R.HARRAR
- RAMBAUD Jerome: No conflict of interest
- RAMBAUD Thomas: No conflict of interest
- RANGER Noemie: Research support/Scientific studies: Pathoquest (Paris)
- RAUCOULES Marc aimé: No disclosure
- RAULT Christophe: No disclosure
- RAZACH Idriss: No disclosure
- RAZAZI Keyvan: Invitation to national or international congresses: Réanimation 2020
- REBIERE Vincent: No conflict of interest
- RECHER Christian: No disclosure
- REGAIEG Kais: No conflict of interest
- REGNIER Jean: No disclosure
- REHOUMA Haythem: No disclosure
- REIGNIER Jean: No conflict of interest
- REIZINE Florian: No conflict of interest
- RELLO Jordi: No conflict of interest
- RENALDO Florence: No conflict of interest
- RENAUDIER Marie: No conflict of interest
- RENAULT Anne: Trainings, Teaching: Université de Bretagne occidentale; Invitation to national or international congresses: Fresenius
- RENOLLEAU Sylvain: No conflict of interest
- REPESSÉ Xavier: No conflict of interest
- RESCHE-RIGON Matthieu: No conflict of interest
- RESIERE Dabor: No conflict of interest
- REUTER Danielle: No disclosure
- REYNAUD Faustine: No disclosure
- REYNAUD Marie: No conflict of interest
- RHANEM Toufiq: No disclosure
- RIAD Zakaria: No disclosure
- RIAUD Charline: No conflict of interest
- RICARD Cécile: No conflict of interest
- RICARD Jean-Damien: Invitation to national or international congresses: Fisher&Paykel
- RICCI Jean-Ehrland: No conflict of interest
- RICHARD Jean-Christophe: Research support/Scientific studies: Hamilton
- RICHARD Régine: No conflict of interest
- RICHARD Vincent: No disclosure
- RICHECOEUR Jack: Trainings, Teaching: Massimo
- RICOME Sylvie: No conflict of interest
- RIGAUD Jean-Philippe: No conflict of interest
- RIM Gharbi: No conflict of interest
- RIMMELE Thomas: No disclosure
- ROBERT Alexandre: No conflict of interest
- ROBERT Blandine: No disclosure
- ROBERT René: Research support/Scientific studies: Fisher and Paykel; Invitation to national or international congresses: Fresenius Medical Care
- ROBERT Tiphaine: No conflict of interest
- ROBERTS Jason A.: Research support/Scientific studies: MSD, Biomerieux; Consultancy, Expert: MSD, Accelerate diagnostics, Biomerieux
- ROBIN Emmanuel: No conflict of interest
- ROBIN Nicolas: No disclosure
- ROBINEAU Olivier: No disclosure
- ROCH Antoine: No disclosure
- ROCHE Anne: No conflict of interest
- ROGER Claire: Consultancy, Expert: PFIZER, FRESENIUS MEDICAL CARE; Invitation to national or international congresses: MSD,PFIZER
- ROLLE Amélie: No conflict of interest
- RONDEAU Eric: No disclosure
- RONZIÈRE Thomas: No disclosure
- ROQUILLY Antoine: No disclosure
- ROSSELLI Sylvène: No disclosure
- ROUBY Jean-Jacques: No disclosure
- ROUIS Sana: No conflict of interest
- ROULEAU Stéphane: No conflict of interest
- ROULET Sylvie: No disclosure
- ROULLAND Charlotte: No disclosure
- ROUMELIOTIS Nadia: No conflict of interest
- ROUSSE Natacha: No disclosure
- ROUSSEAU Anne-Françoise: Invitation to national or international congresses: Orion
- ROUSSEAU Chloe: No disclosure
- ROUSSEAU Christophe: No conflict of interest
- ROUSSET David: No disclosure
- ROUSTAN Jerome: No disclosure
- ROUX Damien: Consultancy, Expert: Astellas
- ROUZE Anahita: No disclosure
- ROZENBERG Emmanuel: No conflict of interest
- ROZIER Romain: Invitation to national or international congresses: SFAR, ARCOTHOVA
- RUCKLY Stephane: Research support/Scientific studies: Pfizer
- RUDLER Marika: No disclosure
- SAADE Anastasia: No conflict of interest
- SABA Makni: No conflict of interest
- SABIA Marie: No disclosure
- SABRINA Chaoueh: No conflict of interest
- SACCHERI Clément: No disclosure
- SACLEUX Sophie-Caroline: No conflict of interest
- SADAT Souhila: No conflict of interest
- SAGARDOY Thomas: No disclosure
- SAGNIER Anne: No disclosure
- SAILLARD Colombe: Trainings, Teaching: Amgen, Novartis; Invitation to national or international congresses: Amgen
- SAINT-SARDOS Pierre: No disclosure
- SAKIS Dorra: No conflict of interest
- SALAME Ephrem: No disclosure
- SALEM Joe Elie: No conflict of interest
- SALEM Mohamed: No disclosure
- SALIBA Faouzi: Research support/Scientific studies: Novartis, Astellas, Chiesi, Baxter; Consultancy, Expert: Novartis, Baxter, Vital therapies; Invitation to national or international congresses: Gilead, Abbvie, Novartis, Astellas, Pfizer, Chiesi
- SALOMON Elsa: No conflict of interest
- SALOMON Remi: No disclosure
- SALVADOR Elodie: No conflict of interest
- SALVI Nadege: No disclosure
- SAMUEL Didier: Consultancy, Expert: Intercept, Biotest gilead sciences, abbvie
- SAN Sovannarith: No disclosure
- SANGLA Frédéric: No conflict of interest
- SAPORITO Andrea: Consultancy, Expert: BBraun, Sintetica; Invitation to national or international congresses: Sintetica; Patent or product inventor: Lightsens
- SAUNEUF Bertrand: No conflict of interest
- SAUTHIER Michael: Research support/Scientific studies: Fonds de recherche en santé du Québec (gouvernement)
- SAUVAGE Brice: No conflict of interest
- SAVARY Guillaume: No conflict of interest
- SAVARY Lea: No conflict of interest
- SAVOYE COLLET Celine: No conflict of interest
- SAZIO Charline: No disclosure
- SBOUI Sajida: No conflict of interest
- SCALA BERTOLA Julien: No conflict of interest
- SCHELLONGOWSKI Peter: Consultancy, Expert: Getinge, Novartis, Gilead, Astro Pharma, Shire, Astellas, Basilea, Baxter
- SCHENCK Maleka: No conflict of interest
- SCHMIDT Aline: No disclosure
- SCHMIDT Matthieu: Consultancy, Expert: Getinge, Drager
- SCHMITT Johan: Invitation to national or international congresses: Société Francaise d’Anesthésie Réanimation; Congrès Anesthésie Réanimation Urgentistes Militaires
- SCHNEIDER Francis: No conflict of interest
- SCHNELL David: No conflict of interest
- SCHNITZLER Béatrice: No disclosure
- SCHORTGEN Frédérique: Trainings, Teaching: ESICM, SRLF, IHTMS, CREUF, AFIB; Invitation to national or international congresses: ESICM, SRLF, IHTMS, CREUF, AFIB
- SCHUGHART Klaus: No disclosure
- SCHULTZ Marcus: No conflict of interest
- SCHWEBEL Carole: Invitation to national or international congresses: Pfizer
- SCICLUNA Brendon: No disclosure
- SCULIER Jean-Paul: No conflict of interest
- SEE Perrine: No conflict of interest
- SEGHBOYAN Jean-marie: No disclosure
- SEGUIN Amelie: No conflict of interest
- SEGUIN Philippe: Consultancy, Expert: LFB; Invitation to national or international congresses: Astellas
- SEJOURNE Caroline: No conflict of interest
- SELLAMI Walid: No conflict of interest
- SENDID Boualem: Research support/Scientific studies: AllFun project, FP7 European Commission; Invitation to national or international congresses: Pfizer
- SENHADJI Lahcen: No conflict of interest
- SERBOUTI Rita: Research support/Scientific studies: Fresenius Medical Care; Consultancy, Expert: Fresenius Medical Care; Trainings, Teaching: Fresenius Medical Care; Invitation to national or international congresses: Fresenius Medical Care
- SERFATY Lawrence: No disclosure
- SÉRIE Mathieu: No conflict of interest
- SHAW Geoffrey M.: No conflict of interest
- SHI Rui: No conflict of interest
- SHIMI Abdelkrim: No disclosure
- SHOJAEI Maryam: No disclosure
- SI-TAHAR Mustapha: Consultancy, Expert: Cynbiose Respiratory; Stock shareholder: Cynbiose respiratory
- SIAMI Shidasp: No conflict of interest
- SILVA Daniel: Research support/Scientific studies: Fresenius Medical Care France; Consultancy, Expert: Fresenius Medical Care France; Invitation to national or international congresses: Xenios Novalung, Heilbronn, Germany
- SILVA Stein: No disclosure
- SIMILOWSKI Thomas: Research support/Scientific studies: Lungpacer; Consultancy, Expert: ADEP Assistance, AstraZeneca, Chiesi France, GSK France, Lungpacer Inc., KPL consulting, TEVA; Trainings, Teaching: Chiesi France, GSK France, Novartis France; Invitation to national or international congresses: Novartis France; Patent or product inventor: Air Liquide Medical Systems, MyBrain Technology
- SIMON Marie: Trainings, Teaching: Fresenius, Pfizer, Gilead, MSD; Invitation to national or international congresses: Pfizer, MSD
- SIMON-PIMMEL Jeanne: No conflict of interest
- SIRAULT Bruno: No disclosure
- SIRODOT Michel: No disclosure
- SLAMA Michel: No disclosure
- SLIM Amine: No disclosure
- SMIELEWSKI Peter: No disclosure
- SOARES Marcio: Stock shareholder: Epimed Solutions; Patent or product inventor: Epimed Solutions
- SOLO NOMENJANAHARY Mialitiana: No conflict of interest
- SOMMET Julie: No conflict of interest
- SONNEVILLE Romain: Research support/Scientific studies: French ministry of Health, SRLF, ESICM; Consultancy, Expert: Baxter; Trainings, Teaching: Panacea
- SONZINI Roberta: No conflict of interest
- SORTAIS Clara: No conflict of interest
- SOUBRANE Olivier: No disclosure
- SOUHEIL Elatrous: No disclosure
- SOULIGNAC Chloe: No disclosure
- SOUWEINE Bertrand: Consultancy, Expert: MSD; Trainings, Teaching: Gilead; Invitation to national or international congresses: Pfizer
- SPAGNOLETTI Marco: No conflict of interest
- STECKELMACHER Claire: No disclosure
- STOCKX Luc: Research support/Scientific studies: Phenox, Medtronic; Consultancy, Expert: Phenox; Trainings, Teaching: Phenox; Invitation to national or international congresses: Phenox
- STOCLIN Annabelle: No conflict of interest
- SUBRA Gilles: No disclosure
- SULTANIK Philippe: Invitation to national or international congresses: ABBVIE, GILEAD, BIOTEST
- SUZANNE Marie: Invitation to national or international congresses: NOVARTIS PHARMA SAS
- SZTRYMF Benjamin: No conflict of interest
- TABET-AOUL Nabil: No conflict of interest
- TABRA Cécilia: No conflict of interest
- TACCONE Fabio: No disclosure
- TADIÉ Jean-Marc: No conflict of interest
- TAILPIED Pierre: No conflict of interest
- TAJELLIJITI Nissrine: No conflict of interest
- TAMION Fabienne: No conflict of interest
- TANAKA Kosuke: Research support/Scientific studies: I am a full time employee of Asahi Kasei Pharama America, the reserch sponsor.
- TANAKA Sébastien: No conflict of interest
- TANDJAOUI-LAMBIOTTE Yacine: Trainings, Teaching: Gambro
- TANG Benjamin: No disclosure
- TANKOVIC Jacques: No conflict of interest
- TARTE Karin: No disclosure
- TATHAM Amy: No disclosure
- TATTEVIN Pierre: Research support/Scientific studies: Astellas, Biomérieux, Gilead, Pfizer, et MSD; Consultancy, Expert: Gilead, Astellas, Coreviome, MSD, Mylan, Shionogi, et Pfizer,; Invitation to national or international congresses: Mylan, MSD, Pfizer, Biomérieux, Gilead, et Astellas
- TAVITIAN Suzanne: No disclosure
- TCHETIKE Pikabalou: No disclosure
- TEBOUL Jean-Louis: Consultancy, Expert: Getinge
- TEITEN Christelle: No conflict of interest
- TEIXEIRA Luis: No disclosure
- TEMIME Johanna: No conflict of interest
- TERNACLE Julien: No disclosure
- TERREAUX Jeremy: No disclosure
- TERZI Nicolas: Invitation to national or international congresses: Boehringer Ingelheim
- THABET Lamia: No conflict of interest
- THABUT Dominique: No conflict of interest
- THENOT Victoire: No conflict of interest
- THEOCHARIDOU Eleni: No disclosure
- THEODOSE Igor: No conflict of interest
- THEPOT-SEEGERS Valérie: No conflict of interest
- THEVENIN Didier: No conflict of interest
- THIEBLEMONT Catherine: No disclosure
- THIERY Guillaume: Consultancy, Expert: Gilead, advisory board, CAR-T Cells; Trainings, Teaching: Amgen, lecture CAR-T cells
- THILLE Arnaud: Research support/Scientific studies: Fisher&Paykel; Trainings, Teaching: Fisher&Paykel, GE Healthcare, Maquet - Getinge, Covidien; Invitation to national or international congresses: Fisher&Paykel, GE Healthcare, Maquet - Getinge, Covidien
- THILLY Nathalie: No conflict of interest
- THIREAU Jerome: No conflict of interest
- THIRION Marina: Trainings, Teaching: Panacéa Conseil & Formation Santé; Invitation to national or international congresses: Pfizer (congrès SRLF 2019)
- THIRY Eric: No disclosure
- THOMAS Laure: No conflict of interest
- THY Michael: No conflict of interest
- TIMSIT Jean-François: Research support/Scientific studies: Merck, bioMerieux, Pfizer; Consultancy, Expert: Nabriva, Merck, Pfizer, Bayer Pharma, Paratek; Trainings, Teaching: Gilead
- TISSIÈRES Pierre: Research support/Scientific studies: bioMerieux; Consultancy, Expert: Sedana, Baxter
- TOFFART Anne-Claire: Research support/Scientific studies: Roche, BMS, Astra Zeneca; Consultancy, Expert: Roche, BMS, MSD, Astra Zeneca, Vifor PharmaBoehringer Ingelheim, Pfizer; Trainings, Teaching: Roche, MSD, Astra Zeneca; Invitation to national or international congresses: Roche, BMS, MSD, Astra Zeneca, Vifor PharmaBoehringer Ingelheim, Pfizer
- TOMBERLI Françoise: No conflict of interest
- TOUBIANA Julie: No conflict of interest
- TOUIL Yosr: No conflict of interest
- TOUMI Radhouane: No conflict of interest
- TOURTIER Jean-pierre: No disclosure
- TRELUYER Jean-marc: No disclosure
- TRÉLUYER Jean-Marc: Research support/Scientific studies: APHP and Université de Paris
- TREMOLIERES Pierre: No conflict of interest
- TRIDON Chloé: No disclosure
- TRIFI Ahlem: No conflict of interest
- TRIKI Amal: No conflict of interest
- TUDESQ Jean-Jacques: Trainings, Teaching: MSD, Gilead; Invitation to national or international congresses: Pfizer
- TUFFET Samuel: No conflict of interest
- TURBIL Emanuele: No conflict of interest
- TURKI Olfa: No conflict of interest
- UHEL Fabrice: No conflict of interest
- URBINA Tomas: No conflict of interest
- URIEN Saïk: No conflict of interest
- UYTTENDAELE Vincent: Research support/Scientific studies: FNRS FRIA–Fund for Research and Training in Industry and Agriculture (Belgium)
- VALADE Sandrine: Consultancy, Expert: Sanofi; Invitation to national or international congresses: Pfizer
- VALENTE Mélanie: No disclosure
- VALENTINO Ruddy: No disclosure
- VALETTE Xavier: No conflict of interest
- VALKONEN Miia: No disclosure
- VALLEE Barthélémy: No conflict of interest
- VALLY Shazima: No disclosure
- VAN BOXSTAEL Sam: Trainings, Teaching: ALSG; Invitation to national or international congresses: ZOL Genk
- VAN CAUTER Sofie: Consultancy, Expert: Biogen; Invitation to national or international congresses: Biogen
- VAN DE LOUW Andry: No conflict of interest
- VAN DELDEN Christian: Consultancy, Expert: Astellas, Pfizer, MSD, Gilead; Trainings, Teaching: Basilea
- VAN DER POLL Tom: No disclosure
- VAN GRUNDERBEECK Nicolas: Research support/Scientific studies: MSD; Trainings, Teaching: Getinge group; Invitation to national or international congresses: AIr Liquide
- VAN POUCKE Sven: No conflict of interest
- VAN VUGHT Lonneke: No disclosure
- VAN-GYSEL Damien: No conflict of interest
- VANDERMEULEN Elly: No conflict of interest
- VANDROUX David: Invitation to national or international congresses: Nordic pharma
- VANDUEREN Charlotte: No conflict of interest
- VANELDEREN Pascal: Research support/Scientific studies: BBraun
- VARNOUS Shaida: No disclosure
- VEBER Benoit: No disclosure
- VEDRENNE-CLOQUET Meryl: Trainings, Teaching: MSD; Invitation to national or international congresses: Fresenius
- VERGEZ François: No disclosure
- VERLHAC Camille: Research support/Scientific studies: Fresenius kabi; Invitation to national or international congresses: Hospal
- VERRIER Nathalie: No disclosure
- VEYRADIER Agnès: No conflict of interest
- VEZIRIS Nicolas: Research support/Scientific studies: Accelerate Diagnostic et Curetis; Consultancy, Expert: becton dickinson; Invitation to national or international congresses: otsuka
- VIALLARD Marcel Louis: No conflict of interest
- VICAIRE Hugues: No conflict of interest
- VIEILLARD-BARON Antoine: Research support/Scientific studies: GSK
- VIGNERON Clara: No conflict of interest
- VIGNON Philippe: No conflict of interest
- VILLA Elodie: No conflict of interest
- VILLENEUVE Laurent: No disclosure
- VIMEUX Sylvie: No conflict of interest
- VIMPERE Damien: No conflict of interest
- VINCENT Arthur: No conflict of interest
- VINCENT Florent: Trainings, Teaching: SANOFI
- VINCENT Francois: No disclosure
- VINCENT Jean-Louis: No disclosure
- VINCENTELLI André: Consultancy, Expert: Abbott; Medtronic; Trainings, Teaching: Abbott; Invitation to national or international congresses: Abott
- VINETTI Marco: Research support/Scientific studies: Combacte, Adrenomed AG, Inotrem, Artisan Pharma, Sepcell; Consultancy, Expert: Orion
- VINSONNEAU Christophe: No conflict of interest
- VISSAC Camille: No conflict of interest
- VIVIEN Benoit: No conflict of interest
- VIVIER Emmanuel: No conflict of interest
- VOIRIOT Guillaume: Research support/Scientific studies: Biomérieux, SOS oxygène, Janssen; Consultancy, Expert: Biomérieux; Invitation to national or international congresses: Biomérieux
- VON KIETZELL Matthias: Invitation to national or international congresses: Gilead, Viif, MSD, Pfizer
- VUILLARD Constance: No disclosure
- VUNDELINCKX Joris: Research support/Scientific studies: Interreg Euregio Maas-Rijn, European goverment
- VUOTTO Fanny: No conflict of interest
- WAINTRAUB Xavier: Research support/Scientific studies: Medtronic, Abbott, Sorin; Consultancy, Expert: Boston, Abbott, Biosense Webster; Trainings, Teaching: Boston; Invitation to national or international congresses: Medtech
- WALLET Florent: Consultancy, Expert: Novartis Gilead Kite; Invitation to national or international congresses: Gilead Kite
- WALLIS Steven c.: No conflict of interest
- WEISS Emmanuel: Consultancy, Expert: MSD; Invitation to national or international congresses: MSD
- WEISS Nicolas: Consultancy, Expert: Med Day pharmaceuticals
- WIBAIL Alain: No disclosure
- WIESEN Patricia: No conflict of interest
- WITTEBOLE Xavier: Research support/Scientific studies: Investigator fee paid to the hospital by AKPA; Consultancy, Expert: AKPA
- WORMSER Johan: No conflict of interest
- WOUTERS Patrick: Research support/Scientific studies: Fresenius; Consultancy, Expert: aguettant; Invitation to national or international congresses: Vifor
- YACOUBI Wejden: No conflict of interest
- YAGER Hélène: No conflict of interest
- YAHYA Yosra: No conflict of interest
- YAKINI Khalid: No disclosure
- YAKOUBEN Karima: No disclosure
- YONIS Hodane: Invitation to national or international congresses: LVL medical et Pfizer
- YOUNAN Romy: No conflict of interest
- YOUSSOUFA Atika: No disclosure
- ZACHARIA Mahi: No disclosure
- ZAFRANI Lara: Research support/Scientific studies: Jazz Pharmaceuticals
- ZAMBON Olivier: No disclosure
- ZAOUAK Nadia: No conflict of interest
- ZAOUCHE Khedija: No conflict of interest
- ZARROUGUI Wafa: No conflict of interest
- ZE MINKANDE Jacqueline: No disclosure
- ZEGHDOUD Dalila: No disclosure
- ZERBIB Yoann: No conflict of interest
- ZERHOUNI Amel: No conflict of interest
- ZERHOUNI Amine: No conflict of interest
- ZERIMECH Farid: No conflict of interest
- ZEROUALI Khalid: No disclosure
- ZHENG Yi: No conflict of interest
- ZIMMERLI Stefan: Research support/Scientific studies: MSD, Pfizer, Gilead; Consultancy, Expert: MSD, Pfizer; Trainings, Teaching: Gilead; Invitation to national or international congresses: Gilead
- ZOUAOUI BOUDJLETIA Karim: No disclosure
- ZOUARI Farah: No conflict of interest
- ZOUARI Fatma: No conflict of interest
- ZOUARI Hager: No conflict of interest
- ZRIBI Malek: No conflict of interest
- ZUBER Benjamin: Invitation to national or international congresses: Oxyvie

